# Genome-Wide Analysis of APETALA2/Ethylene-Responsive Factor (AP2/ERF) Gene Family in Barley (*Hordeum vulgare* L.)

**DOI:** 10.1371/journal.pone.0161322

**Published:** 2016-09-06

**Authors:** Baojian Guo, Yafeng Wei, Ruibin Xu, Shen Lin, Haiye Luan, Chao Lv, Xinzhong Zhang, Xiyun Song, Rugen Xu

**Affiliations:** 1 Jiangsu Key Laboratory of Crop Genetics and Physiology/Co-Innovation Center for Modern Production Technology of Grain Crops, Key Laboratory of Plant Functional Genomics of the Ministry of Education, Barley Research Institution of Yangzhou University, Yangzhou University, Yangzhou, China; 2 JiangSu Yanjiang Institute of Agricultural Sciences, Nantong, China; 3 College of Agronomy and Plant Protection, Qingdao Agricultural University, Qingdao, China; National Institute of Plant Genome Research, INDIA

## Abstract

APETALA2/Ethylene-Responsive Factor (AP2/ERF) gene family is plant specific transcription factor. It plays critical roles in development process, tolerance to biotic and abiotic stresses, and responses to plant hormones. However, limited data are available on the contributions of *AP2*/*ERF* gene family in barley (*Hordeum vulgare* L.). In the present study, 121 *HvAP2*/*ERF* genes in barley were identified by using bioinformatics methods. A total of 118 *HvAP2/ERF* (97.5%) genes were located on seven chromosomes. According to phylogenetic classification of AP2/ERF family in *Arabidopsis*, HvAP2/ERF proteins were divided into AP2 (APETALA2), RAV (Related to ABI3/VP), DREB (dehydration responsive element binding), ERF (ethylene responsive factors) and soloist sub families. The analysis of duplication events indicated that tandem repeat and segmental duplication contributed to the expansion of the AP2/ERF family in barley. *HvDREB1s*/*2s* genes displayed various expression patterns under abiotic stress and phytohormone. Taken together, the data generated in this study will be useful for genome-wide analysis to determine the precise role of the *HvAP2*/*ERF* gene during barley development, abiotic stress and phytohormone responses with the ultimate goal of improving crop production.

## Introduction

The APETALA2/Ethylene-Responsive Factor (AP2/ERF) gene family is one of largest gene families, encoding plant-specific transcription factors. The AP2/ERF superfamily is defined by the AP2/ERF domain, which consists of about 60 to 70 amino acids and is involved in DNA binding. The AP2/ERF superfamily is divided into AP2 (APETALA2), RAV (Related to ABI3/VP), DREB (dehydration responsive element binding), ERF (ethylene responsive factors) and soloist sub families [1–4]. The AP2 subfamily members contain multiple AP2/ERF domains or lacking a conserved WLG motif within AP2/ERF domain, the RAV subfamily transcription factors include a single AP2/ERF domain and B3 domain, DREB and ERF subfamily possess only one AP2/ERF domain, the remaining genes are defined as soloist [1]. The *AP2*/*ERF* gene members play an important role in the regulation of plant development and tolerance to biotic and abiotic stresses [4–12]. With more extensive plant genome sequences, AP2/ERF gene family have been identified in various plants, such as *Arabidopsis* [3], rice [3], maize [4], sorghum [13], soybean [14], foxtail millet [15]. However, no research has been performed for the identification and characterization of the AP2/ERF family in barley.

Salinity and drought are two of the most serious abiotic stress factors. Plants have evolved on the molecular level to survive these environmental stresses. Plant hormone ABA also plays an important role in improving the tolerance of plants to drought and salinity [16–19]. The DREBs/CBFs (Dehydration Responsive Element Binding proteins/C-repeat CRT binding transcription factors), hereafter referred as DREBs is a major member of AP2/ERF gene family, which binds to DRE (A/GCCGAC) and/or the CRT (TGGCCGAC) core *cis*-acting sequences in promoters of drought and salt responsive genes and regulates stress-responsive genes [20]. The ectopic overexpression of *DREB* genes in plant systems resulted in improved salt stress tolerance as positive regulator [20–22]. Enhanced expression of *OsDREB2A* and *OsDREB1F* results in improved drought and salt stress tolerance in rice and *Arabidopsis* [22]. In rice, *OsDREB1A* and *OsDREB1F* were induced by cold stress. *OsDREB1F* was also induced by drought, salt and ABA treatment. Over-expression of *OsDREB1A* and *OsDREB1F* resulted in with higher tolerance to drought, high-salt stress in *Arabidopsis* [20–21]. In *Arabidopsis*, a *cbf2* mutant was identified by using a reverse genetic approach, in which the *CBF2*/*DREB1C* gene was abnormal. *cbf2* mutant showed increased tolerance to drought and salt stress. Expression analysis indicated that *CBF2*/*DREB1C* negatively regulated the expression of *CBF1*/*DREB1B* and *CBF3*/*DREB1A* [23]. Remarkably, *DREB1*/*2* genes were induced under drought and salt stress, indicating cross-talk between them [17, 21, 24–26]. These results suggest that the functions of *DREB1*s and *DREB2*s genes in combination with ABA are conserved both in monocot and dicot plants, and play an important role in plant drought and salt **s**tress responses [20].

Barley is considered as the most salt and drought tolerant among cereal crops but cultivars show considerable variation to different tolerances [27]. With growing world population, global food production should be meet the demands by improving abiotic tolerance of crops, and has become the focus for enhancing breeding efforts [27]. In the present study, we identified 121 *HvAP2*/*ERF* genes in barley by using bioinformatics methods and constructed a phylogenetic tree. Most of the *HvAP2*/*ERF* genes were localized to chromosomes using drawing tools and duplication events were also analyzed. The expression patterns of one hundred and seven *AP2*/*ERF* genes were detected using published RNA sequencing. Finally, the expression level of twenty one genes in response to drought, high-salt stress and exogenous ABA was performed by Quantitative real-time PCR. These results will be useful in further investigation of the AP2/ERF family in plants.

## Materials and Methods

### Sequence database searches

Multiple database searches were performed to collect all members of the barley *HvAP2*/*ERF* gene. Barley sequence data were sourced from the Morex assembly (http://webblast.ipk-gatersleben.de/barley/) [28], Gramene (http://ensembl.gramene.org/Hordeum_vulgare/Info/Index) and NCBI database (http://www.ncbi.nlm.nih.gov/). We used the BLAST programs (TBLASTN and BLASTN) which is available on the IPK barley genome database and NCBI barely EST database. We used the amino acid sequence of the AP2/ERF domain from *Arabidopsis* (*Arabidopsis thaliana*), rice (*Oryza sativa* L.) and maize (*Zea mays* L.) as a query sequences [3, 4]. To increase the extent of the database search results, we also performed the database searches using amino acid sequences of the *AP2*/*ERF* genes of some members of the Transcription Factor Database (http://planttfdb.cbi.pku.edu.cn/) [29]. Barley *HvAP2*/*ERF* candidate genes with expected (E) values less than 1.0 were retrieved and the non-redundant sequences were examined for the presence of conserved AP2/ERF domain using the domain analysis programs Pfam (Protein family: http://pfam.sanger.ac.uk/) [30], HMMSCAN (https://www.ebi.ac.uk/Tools/hmmer/search/hmmscan) and SMART (Simple Modular Architecture Research Tool: http://smart.embl-heidelberg.de/) with the default cutoff parameters [31]. The isoelectric points and protein molecular weights were obtained with the help of the proteomics and sequence analysis tools on the ExPASy proteomics server (http://expasy.org/) [32]. The gene names of *HvDREB* and *HvERF* was given according to the ascending order of AP2/ERF domain of analysis in DREB and ERF subfamily. The gene names of *HvAP2* and *HvRAV* was given according to the ascending order of the phylogenetic tree in the AP2 and RAV subfamily.

### Chromosomal location, gene structure, promoter and duplication events of *HvAP2/ERF* genes

The chromosomal locations were retrieved from the Gramene (http://ensembl.gramene.org/Hordeum_vulgare/Info/Index). All genes were mapped to the chromosomes with MapDraw software [33]. The exon/intron structures were constructed using GSDS (http://gsds.cbi.pku.edu.cn/) [34]. Maximum 2,000 bp promoter regions were examined to identify *cis*-regulatory element using PlantCARE (http://bioinformatics.psb.ugent.be/webtools/plantcare/html/). Tandem duplication genes were identified manually if they were within 10 predicted genes or within 30 kb apart of each other and marked on the barley physical map [35]. Segmental duplications were identified by BLASTP ten predicted proteins upstream and downstream of each *HvAP2*/*ERF* [36].

### Phylogenetic tree analysis

Full-length amino acid sequences of *HvAP2*/*ERF* genes identified in barley were aligned using the Clustal X 1.83 program with default pairwise and multiple alignment parameters. The phylogenetic tree was constructed based on this alignment result using the neighbor joining (NJ) method in MEGA version 6 with the following parameters [37]: Poisson correction, pairwise deletion, uniform rates and bootstrap (1000 replicates). Conserved motifs were investigated by multiple alignment analyses using MEME version 3.0 [38].

### Comparative genomics analysis among barley, maize, rice, *Brachypodium* and foxtail millet

Comparative genomics analysis was performed according to Lata et al [15]. The protein sequences of HvAP2/ERF proteins were carried out BLASTP search against protein sequences of maize, rice, *Brachypodium* and foxtail millet (http://gramene.org/; www.phytozome.net), orthologous genes was also performed reciprocal BLASTP search to conform relationship among them. Cutoff with E-value≤1e-05 and at least 80% similarity were considered significant. The amino-acid sequence of paralogous and orthologous AP2/ERF proteins combined the corresponding CDS sequences among barley, maize, rice, *Brachypodium* and foxtail millet were aligned using Clustal W, and then analyzed by using PAL2NAL (http://www.bork.embl.de/pal2nal/) for calculating the synonymous (Ks) and non-synonymous (Ka) substitution rates [39].

### Expression profiling analysis of the *HvAP2/ERF* genes

Gene expression data from eight tissues of the cultivar ‘Morex’ were obtained by making use of the barley genome database (http://apex.ipk-gatersleben.de/apex/f?p=284:10: 6281639160219::NO). Eight tissues of the cultivar ‘Morex’ earmarking stages of the barley life cycle, including 4-day embryos dissected from germinating grains, roots and shoot from the seedlings (10 cm shoot stage), young developing inflorescences (5 mm), developing inflorescences (1–1.5 cm), developing tillers at six leaf stage (the third internode), 5 and 15 days post-anthesis developing grain (DPA) (bracts removed), which were selected for deep RNA sequencing (RNA-seq) [28]. The expression patterns are presented as heat maps in green/yellow/red/ coding, which reflected the FPKM (Fragments Per Kilobase of transcript per Million mapped reads) with red indicating high expression level, yellow indicating middle expression level, and green indicating low expression level.

### Plant materials and treatments

Barley Gairdner was a salt-sensitive variety and CM72 was a salt-tolerant variety [40–41]. Whole-plant responses to salinity, polyethylene glycol (PEG) and abscisic acid (ABA) were studied in glasshouse by using a hydroponic culture technique. Seeds of Gairdner and CM72 were sterilized with 5% sodium hypochlorite for 10 min and rinsed with distilled water, then germinated on wet filter paper at 20°Cfor 3 days. The germinated seeds were transferred into 60-well plastic containers (25 L) with aerated hydroponic solution similar to that used by Wu et al [42]. The pH of the hydroponic solution was adjusted to 6.8 using 1 M HCl as required. All solutions were renewed weekly. Plants were grown in a greenhouse, and a temperature of 20°C/day and 15°C/night. Three weeks old seedlings were exposed to 250 mM NaCl, 20% PEG and 100 μM ABA for 0 h, 1 h, 6 h, 12 h, 24 h and 48 h. After treatment, root and leaf were collected and immediately frozen in liquid nitrogen for RNA extraction with three biological replicates. For each replicate, ten plants of each genotype were used for RNA analysis.

### Quantitative real-time PCR analysis of barley *HvDREB1s/2s* genes under salt, drought and phytohormone treatment

Total RNA of each sample was isolated using an RNA extraction kit (TRIzol reagent, Invitrogen, USA) and incubated with RNase-free DNase I (TaKaRa, Japan) for removing DNA contamination. Quality and yields of RNA were analyzed by agarose gels electrophoresis and NanoDrop 1000 Spectrophotometer V 3.7. First strand cDNA was generated from 2 μg total RNA with M-MLV reverse transcriptase (TaKaRa, Japan) by using random primers. Specific primers for quantitative real-time PCR analysis were listed in S1 Table. Reaction was carried out in 20 μl reaction system containing 10 mM Tris-HCl (pH 8.5), 50 mM KCl, 2 mM MgCl_2_, 0.4 μl DMSO, 200 mM dNTPs, specific PCR primers 10 pmol, Taq DNA polymerase 1 U, SYBR GREEN I fluorescence dye 0.5 μl. Quantitative real-time PCR was performed using a ViiA^™^ 7 Real-Time PCR System (Applied Biosystems, USA). The running protocol was as follows: 94°C for 3 min, followed by 40 cycles at 94°C for 30 s, 58°C for 30 s, 72°C for 30 s, and a final extension of 72°C for 5 min. ADP-ribosylation factor 1-like protein(ADP) was used as an internal control [43]. All reactions were run in triplicate. Ct values were determined by the ViiA^™^ 7 software with default settings. The relative expression levels of target genes were determined using 2^-ΔΔCt^ method [44]. For each sample, PCR was performed with three biological replicates.

## Results

### Identification of the AP2/ERF family genes in barley genome

To identify the *AP2*/*ERF* genes in barley, BLAST searches of the barley databases were performed using the AP2/ERF domains of the *Arabidopsis*, rice and maize protein as a query sequences. A total of 121 *HvAP2*/*ERF* genes from the entire barley genome were identified as potential ones encoding AP2/ERF domain (S2 and S3 Tables). Among which 33 *HvAP2*/*ERF* genes (33/121, 27.3%) were found splice variants of primary transcripts. The number of alternate transcript ranged from 2 to 7 (S3 Table). Based upon the phylogenetic classification of AP2/ERF family in *Arabidopsis*, 19 HvAP2/ERF proteins were classified into AP2 subfamily (S4 Table), with containing one AP2/ERF domain without WLG motif, two (HvAP2-1/3/5/6/7/8/9/11/12/14/17) or three (HvAP2-18) AP2/ERF domain, the remaining members only contained (S4 Table). Six of the proteins, containing both AP2 and B3 domains, were classified into the RAV subfamily (S4 Table). Of the remaining 95 proteins that have only one AP2/ERF domain, 41 members were classified into the DREB subfamily and 54 members were placed in the ERF subfamily based on the conserved motif (S4 Table). A soloist gene including an AP2/ERF like domain sequence was assigned due to a lack of homology with the other *HvAP2*/*ERF* genes (S4 Table). All the identified *HvAP2*/*ERF* genes encode proteins ranging from 123 (HvERF3.5) to 659 (HvAP2-1) amino acids along with a protein mass from 13.38 kD to 69.73 kD and protein pI ranging from 3.98 (HvDREB2.11) to 11.87 (HvERF6.5) (S3 Table).

Table 1 shows the comparison of the *AP2*/*ERF* genes from *Arabidopsis*, maize, rice, foxtail millet and barley. In barley, 95 *HvAP2*/*ERF* genes were classified into the DREB/ERF subfamily. The number of the genes in *Arabidopsis*, maize, rice and foxtail millet were 122, 163, 131 and 138, respectively [3–4, 15]. In AP2 subfamily barley, *Arabidopsis*, maize, rice and foxtail millet had 19, 18, 44, 36, and 28 *HvAP2*/*ERF* genes, respectively. Six barley *HvAP2*/*ERF* genes were predicted to encode proteins with an AP2/ERF domains and B3 domains, which were classified into the RAV subfamily. Similar numbers of genes were also found in *Arabidopsis*, maize, rice and foxtail millet in RAV subfamily (Table 1).

**Table 1:** Summary of the AP2/ERF superfamily in *Arabidopsis*, rice, maize, barley and foxtail millet.

Classification	Group	Barley^a^	*Arabidopsis*^b^	Rice^b^	Maize^c^	Foxtail Millet^d^
AP2 sub family		19	18	36	44	28
	Multiple AP2/ERF domain	13	14		22	
	Single AP2/ERF domain (without WLG motif)	6	4		22	
DREB/ERF sub family		95	122	131	163	138
	DREB sub family	41	57		65	48
	ERF sub family	54	65		98	90
RAV		6	6	7	3	5
Soloist		1	1	0	0	0
Total		121	147	164	210	171

a) Some members of *HvAP2*/*ERF* genes were derived from the Transcription Factor Database (http://planttfdb.cbi.pku.edu.cn/) [29].

b) Nakano et al., 2006 [3];

c) Liu et al., 2013 [4].

d) Lata et al., 2014[15]

### Chromosomal location and structure of *HvAP2/ERF* genes

**Fig 1:**
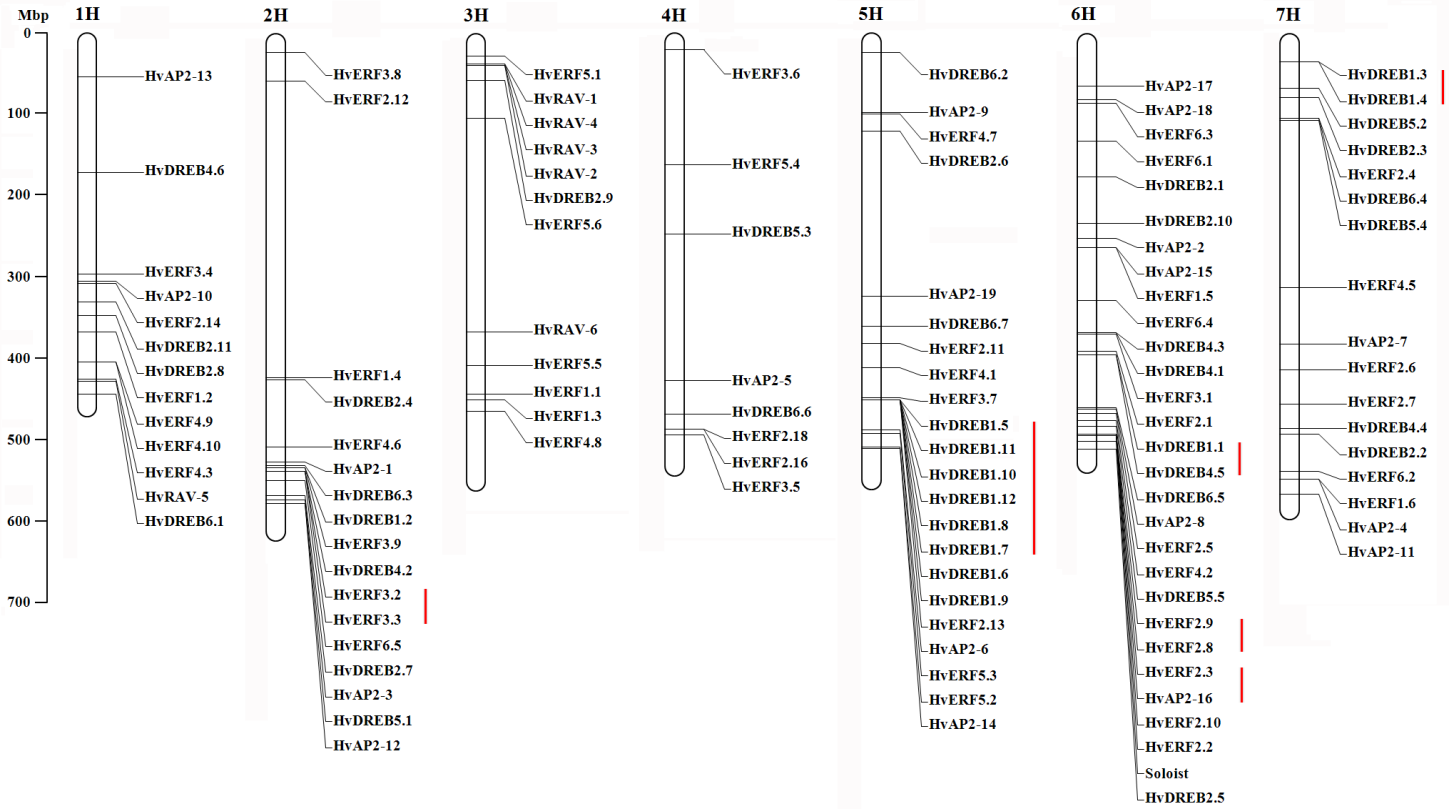
The chromosome location of the *HvAP2*/*ERF* genes in barley. The red lines represent tandemly duplicated gene pairs.

One hundred and eighteen *HvAP2*/*ERF* genes (97.5%, 118/121) were located on 7 chromosomes; three genes (*HvERF2*.*15*, *HvERF2*.*17* and *HvERF4*.*4*) were not found the precise chromosome and physical locations on barley genome (Fig 1, S3 Table). Chromosome 6 had the largest number of *HvAP2/ERF* genes (29 genes) while only eight genes were located on 4H. Other chromosomes contained 13–22 *HvAP2/ERF* genes. Gene structural analyses showed that most *HvAP2/ERF* genes contain one exon (87/121, 71.9%), the remaining *HvAP2/ERF* genes shared 2 (19 genes), 4 (3 genes), 5 (1 gene), 6 (1 gene), 7 (4 genes), 8 (2 genes), 9 (3 genes) and 10 exons (1 gene) (S1 Fig). Duplication events analysis indicated that 22 (22/121, 18.2%) *HvAP2/ERF* genes were tandem repeated (S5 Table) and 6 (6/121, 5.0%) *HvAP2/ERF* genes were segmentally duplicated (S6 Table). The tandem duplicated genes contained six clusters, the largest cluster located on chromosome 5, which including six genes, chromosome 2 and 7 contained one gene pairs, respectively, chromosome 6 including 3 clusters. Remarkably, three *HvAP2/ERF* gene pairs displayed segmentally duplicated, including *HvAP2-5* and *HvAP2-8*, *HvRAV-1* and *HvRAV-2*, *HvERF3*.*6* and *HvERF3*.*9* gene pairs.

### Phylogenetic tree of the HvAP2/ERF proteins in barley

**Fig 2:**
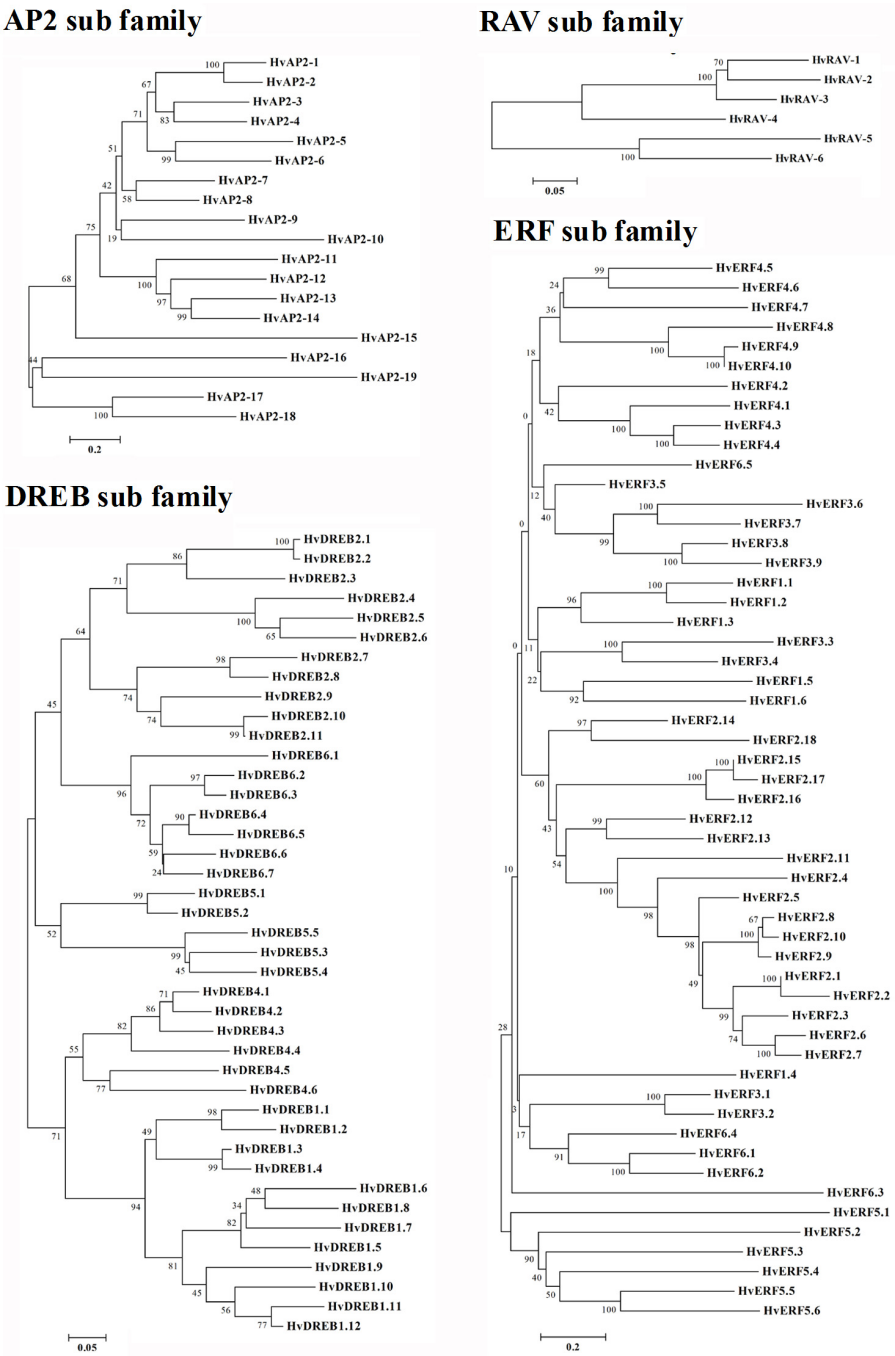
The phylogenetic analysis of *HvAP2*/*ERF* genes in barley.

The AP2/ERF family genes are plant-specific transcription factors. Based on alignment of the HvAP2/ERF domain of barley, 121 barley *HvAP2*/*ERF* genes were classified into the DREB, ERF, AP2 and RAV subfamilies, and one soloist (Fig 2, S3 Table). Phylogenetic trees of the AP2, RAV, DREB and ERF subfamilies in barley were constructed (Fig 2). A total of 41 DREB subfamily genes distributed into the A1, A2, A4, A5 and A6 groups in barley, including 11, 12, 6, 5 and 7 genes, respectively (Fig 2, S2 Fig). Additionally, 54 genes belonging to the ERF subfamily in barley distributed into the B1-B6 groups, including 6, 18, 9, 10, 6 and 5 genes, respectively (Fig 2, S2 Fig). In addition, 19 genes were classified into the AP2 subfamily and six genes into the RAV subfamily (Fig 2).

### Conserved motifs of the HvAP2/ERF proteins

**Fig 3:**
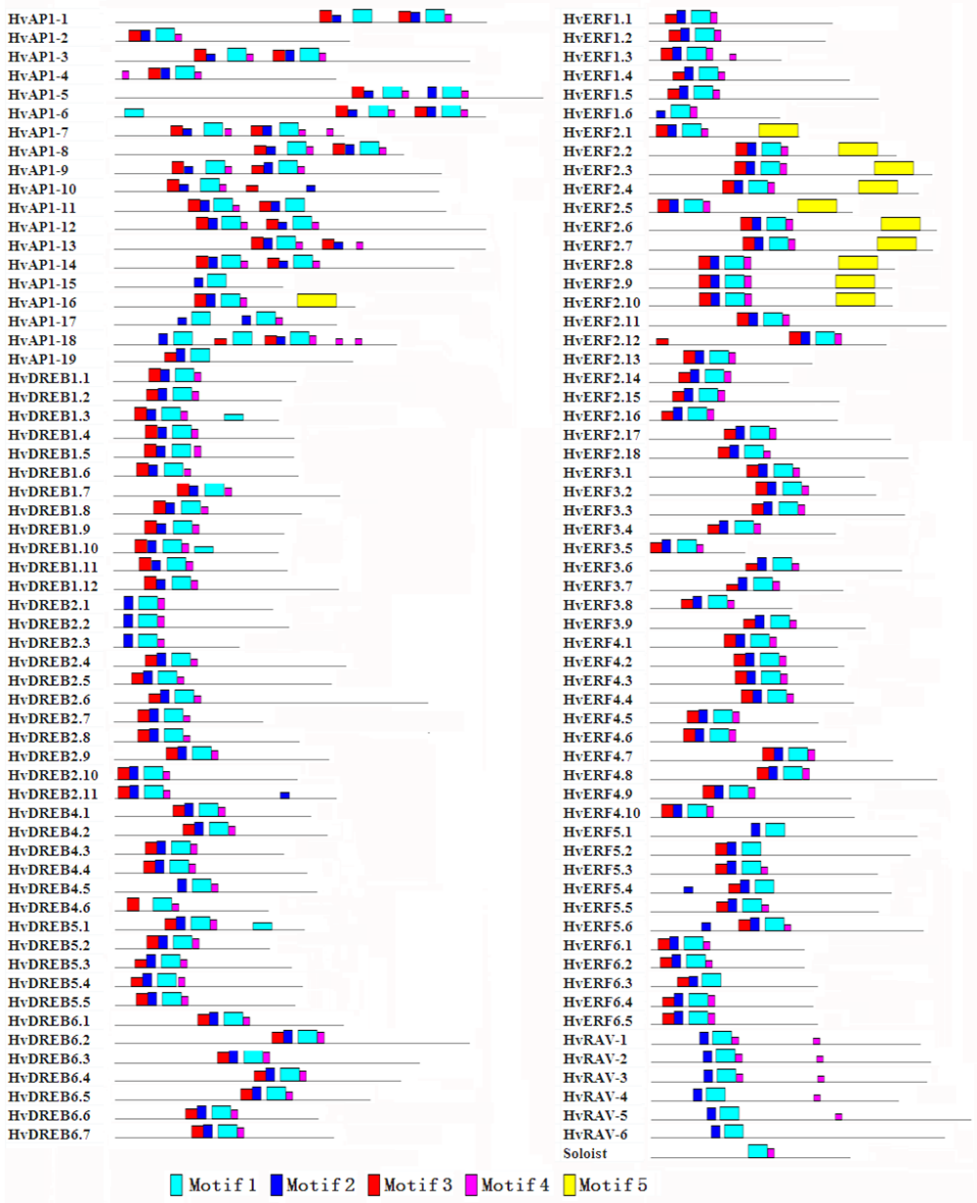
Conserved domains of HvAP2/ERF proteins in barley.

Conserved motifs can provide evidence for further classification as it is likely that identical motifs exhibit similar functions. Five conserved motif (Motif 1–5) were analyzed by using MEME software to identify conserved motifs among proteins in the families and subfamilies. The MEME motif analysis revealed that different HvAP2/ERF proteins had different conserved motifs (Fig 3, S3 Fig). All of the *HvAP2*/*ERF* genes had the full AP2/ERF domain and Motif 1 (S3 Fig, S4 Table). In addition, motif 2, 3 and 4 were detected in most of the *HvAP2/ERF* genes (S2 Fig). Remarkably, motif 5 only observed in HvAP2-16 and HvERF2.1–2.10 (S2 Fig). Two conserved amino acids in the AP2/ERF domains differ between DREB and ERF [45]. In DREB/ERF subfamily, the 14th valine (V14) and the 19th glutamic acid (E19) are conserved in AP2/ERF domain of HvDREB protein, whereas alanine (A) and aspartic acid (D) are conserved in the corresponding positions of the HvERF proteins (S3 Fig).

### Orthologous relationships of *HvAP2/ERF* genes among grass species

To investigate the relationship of *HvAP2/ERF* genes among grass species, comparative genomics was carried out between barley, maize, rice, *Brachypodium* and foxtail millet (S7–S10 Tables). The largest number of orthology of genes was 18 (18/121, 15%) between barley and *Brachypodium* (S10 Table), followed by rice (12/121, 10%) (S8 Table), foxtail millet (9/121, 7%) (S10 Table) and maize (6/121; 5%) (S9 Table). For *HvAP2-10*, barley shared a greater orthology with maize (81%), rice (83%), *Brachypodium* (86%) and foxtail millet (88%), suggesting that *HvAP2-10* shared the similar functions in different species.

### Expression pattern analysis of barley *HvAP2/ERF* genes under different development stage

**Fig 4:**
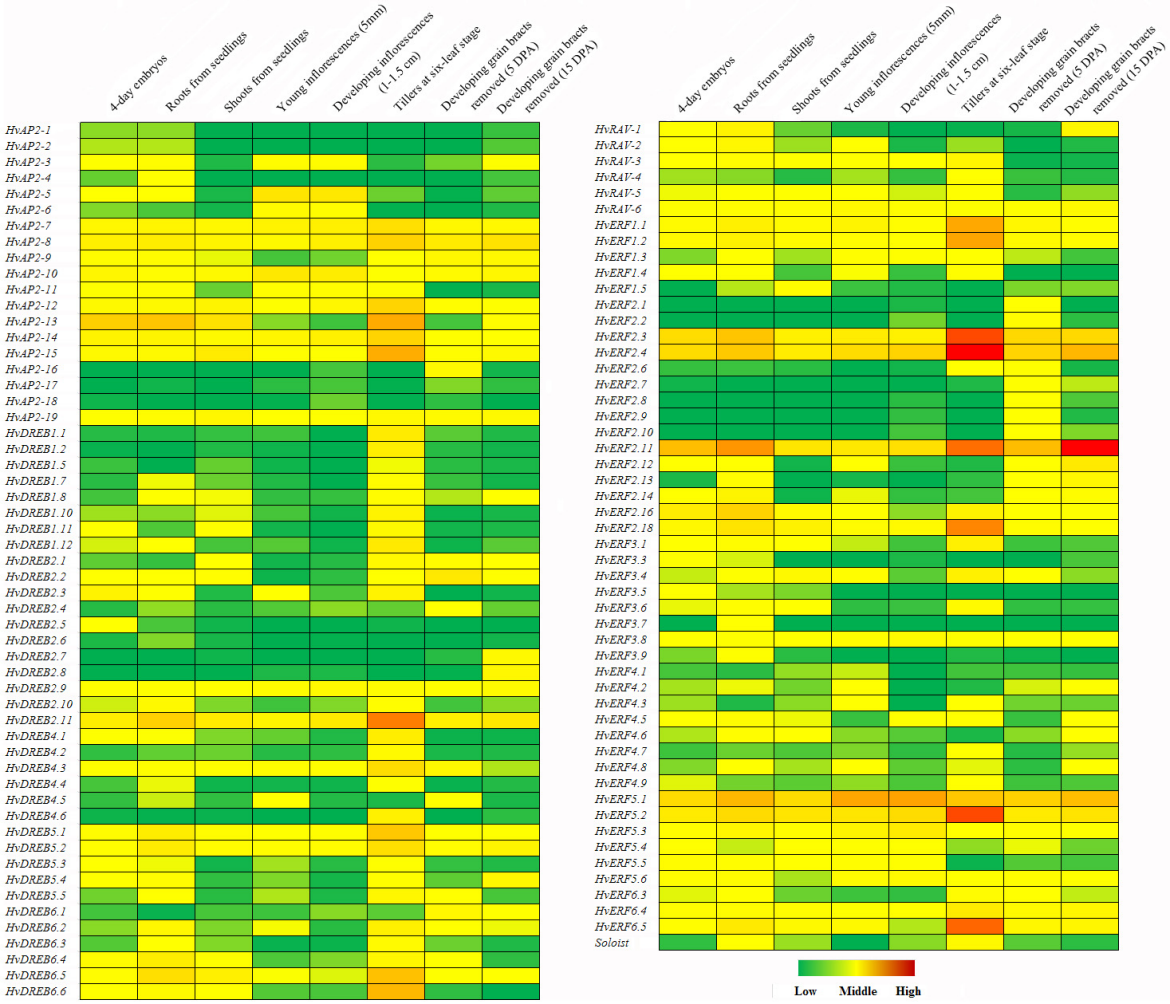
The expression profile of *HvAP2*/*ERF* genes in eight tissues of barley. The expression patterns are presented as heat maps in green/yellow/red/ coding, which reflected the FPKM with red indicating high expression level, yellow indicating middle expression level, and green indicating low expression level.

Expression profiling analysis of the gene family can provide important clues regarding their functions [46]. One hundred and seven *HvAP2/ERF* genes were detected by RNA sequencing, 14 *HvAP2/ERF* genes had no transcript in any tissue (Fig 4). The transcripts of 49 *HvAP2*/*ERF* genes (49/107, 45.8%) were detected in four days embryo after germinating. 40 (40/107, 37.4%) and 68 (68/107, 63.6%) *HvAP2*/*ERF* genes were detected in root and shoot from seedling, respectively. 66 (66/107, 61.7%) *HvAP2*/*ERF* genes were found expressed in developing tillers (six-leaf stage). In addition, 45 (45/107, 42.1%) and 59 (59/107, 55.1%) *HvAP2*/*ERF* genes were expressed in young fluoresce (5 mm and 1–1.5 cm length) and developing grains (5 and 15 DPA), among which *HvERF2*.*11* displayed extremely higher expressed in 15 DPA than 5 DPA developing grains. Remarkably, *HvERF2*.*3* and *HvERF2*.*4* displayed higher expression level in developing tillers than other tissues. Indicating that expression analysis could be contributed to functional analysis of *HvAP2/ERF* genes in barley.

### Expression analysis of *HvDREB1s/2s* genes under abiotic stresses and phytohormone treatments

**Fig 5:**
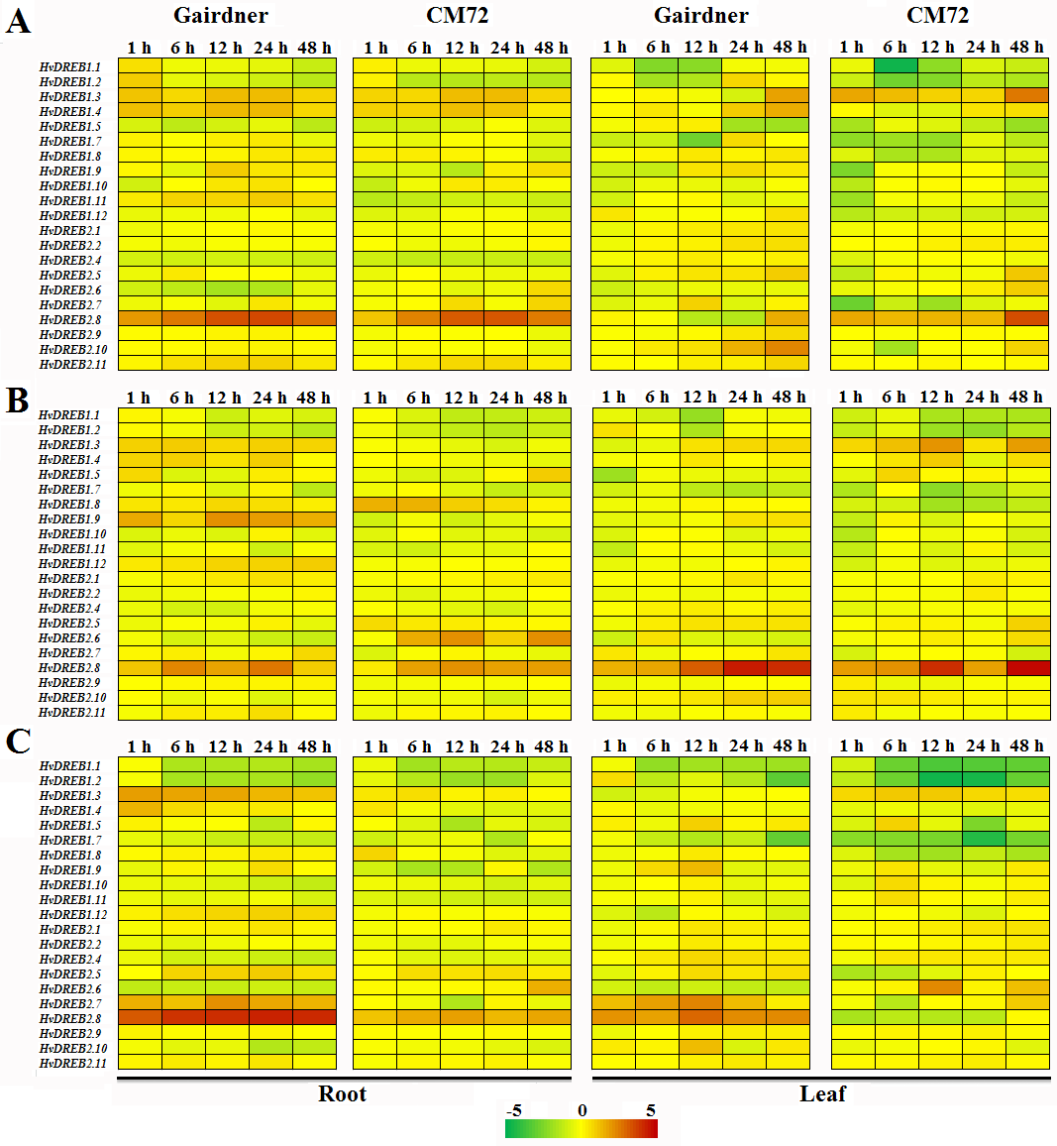
The expression analysis of *HvDREB1s* and *HvDREB2s* genes in response to salinity, dehydration and Abscisic acid (ABA). A, B, and C represent heat maps showing differential gene expression patterns in response to salinity, dehydration and ABA at 0 h, 1 h, 6 h, 12 h, 24 h and 48 h time point.

Plant growth is affected by various abiotic stresses, such as drought, high salinity, and low temperature [21]. Moreover, many *HvDREB1s/2s* genes are induced under stress conditions and phytohormone [4, 11, 17, 21–22, 26]. As shown in Fig 5, the quantitative real-time PCR was performed, including 11 *HvDREB1s* genes and 10 *HvDREB2s* genes. Most of *HvDREB1s* and *HvDREB2s* displayed similar expression patterns between Gairdner and CM72 in root and leaf tissue. Under salt stress, *HvDREB1*.*11* was up-regulated in Gairdner and down-regulated in CM72 in root tissue, the fold change value ranged from 0.41 to 1.00 and 0.79 to 1.35 in Gairdner and CM72, respectively (S11 Table). However, *HvDREB2*.*6* showed the opposite expression pattern. *HvDREB1*.*3 and HvDREB2*.*8* were up-regulated in CM72 but down-regulated in Gairdner in leaf tissue after 12 h treatment. In contrast, *HvDREB1*.*8*, *HvDREB1*.*12*, and *HvDREB2*.*10* were down-regulated in CM72 and up-regulated in Gairdner in leaf tissue (Fig 5A). Remarkably, *HvDREB2*.*8* exhibiting more than a 1-fold change value in Gairdner and CM72 under high salt stress in root tissue (S11 Table). Under drought stress, *HvDREB1*.*8* showed higher expression in CM72 than Gairdner, while *HvDREB1*.*9* showed less expression in CM72 than Gairdner in root tissue. Both genes displayed down-regulated after early treatment (1 h time point) in leaf tissue (Fig 5B). *HvDREB2*.*8* displayed up-regulated expression in both root and leaf tissue in both varieties with the expression level in leaf being higher than that in root tissue (Fig 5B), the average fold change value was 1.72 and 3.00 in root and leaf tissue, respectively (S11 Table).

The plant hormone ABA has important roles in improving the tolerance of plants to drought and salinity stresses [16]. The Quantitative real-time PCR was also carried out to analyze the expression level of selected 21 *HvDREB1s* and *HvDREB2s* genes after ABA treatment (Fig 5C). The heat maps indicated that *HvDREB1*.*3* exhibited higher expressed in Gairdner than in CM72 in root tissue with the average fold change value of 1.61 and 0.37, respectively (S11 Table). On the contrary, *HvDREB1*.*3* was up-regulated expressed in CM72 and down-regulated expressed in Gairdner in leaf tissue. The expression level of *HvDREB2*.*8* was dramatically increased in Gairdner with 3.99-fold change value than CM72 with 1.56-fold change value in both tissues, however, *HvDREB2*.*6* displayed the opposite expression pattern (S11 Table).

Promoter analysis is a vital method to identify regulatory networks between environment stimulation and gene expression. Therefore, a *cis*-element scan was carried out to identify the potential regulatory elements response to abiotic stress and hormones. A total of 82 *cis*-regulatory elements were observed at least in one *HvDREB1s/2s* genes (S12 Table). Several *cis*-regulatory elements were involved in abiotic stress and phytohormone response. For example, ABRE (abscisic acid responsiveness element) and DRE (Dehydration-responsive element) involved in 18 and 1 *HvDREB1s/2s* genes, respectively, both of them were identified as *cis*-regulatory elements that participated in ABA-dependent and ABA-independent response to dehydration-inducible genes [16] (S12 Table). In addition, GARE-motif (gibberellin-responsive element), AuxRR-core (*cis*-acting regulatory element involved in auxin responsiveness) and MBS (MYB binding site involved in drought-inducibility) were also detected in promoter of regions (S12 Table).

## Discussions

### Characterization of the barely AP2/ERF superfamily

AP2/ERF superfamily is one of the largest groups of transcription factor family in plants, which also plays an important role in the transcriptional regulation involving in complicated developmental processes, biotic and abiotic stress, including seed germination, flower development and leaf senescence, fruit ripening, and responses to salt, drought, low temperature and pathogen attack [10, 12, 20, 47–51]. Based on the sequencing of plant genome, the *AP2*/*ERF* gene family was widely analyzed in plants [3–4, 13–15, 52–54]. However, there is still little information about barley *HvAP2*/*ERF* genes. To further investigate the AP2/ERF family in barley, 121 *HvAP2*/*ERF* genes were identified from 79379 (high-confidence and low-confidence genes) annotated genes and genome DNA database [28]. Each of them has notable features with at least one conserved AP2/ERF domain. Remarkably, the number of *HvAP2*/*ERF* gene in barley was less than *Arabidopsis* (147 genes), maize (210 genes) and rice (164 genes) [3, 4]. In addition, the numbers of some subfamilies were similar. For example, the numbers of AP2 subfamily in barley and *Arabidopsis* were 19 and 18, respectively, being half of the number in rice and maize. The numbers of RAV subfamily in barley, *Arabidopsis*, rice and foxtail millet were 6, 6, 7 and 5, respectively. On the contrary, the numbers of other subfamilies were significantly different. For example, 95 genes were identified in the DREB/ERF subfamily in barley. The numbers of genes were 122, 131, 163 and 138 in *Arabidopsis*, rice, maize and foxtail millet, respectively. Recently, it has been reported that segmental duplication events and tandem duplication events in plants were contributed to the expansion of the AP2/ERF family, suggesting that pressure was the predominant force acting on the evolution of the AP2/ERF family [3–4, 15]. In the present study, the Ka/Ks ratio for tandem duplicated and segmental duplication gene-pairs in barley *HvAP2/ERF* genes less than 1.0 expect *HvERF2*.*8* and *HvERF2*.*9* gene pairs, indicating that *HvAP2/ERF* genes undergoing purifying selection in gene expansion (S5 and S6 Tables). The smaller number of genes in *AP2/ERF* family in barley suggested that there may be more other *HvAP2/ERF* genes existing in the unknown genomic regions or chromosome duplication events was restricted in barley evolutionarily expansion.

Conserved motifs in transcription factors play an important role in gene function [1]. A total of fifty conserved motifs outside of the AP2/ERF domain were detected in *Arabidopsis* [3]. In the present study, we analyzed five motifs of AP2/ERF proteins, motif 1 (partial of AP2/ERF domain) was observed in all gene members, other motifs were outside the AP2/ERF domain. Previous studies revealed that DREB and ERF subfamily contained conserved WLG motif in AP2/ERF domain [3–4]. In the present study, WLG motif was highly conserved in DREB and ERF subfamily, as well as in RAV and Solosist subfamily (Table 1, Fig 2). Sequence alignment of ERF subfamily has revealed 14th alanine and the 19th aspartic acid of AP2/ERF domain are conserved, whereas valine and glutamic acid residues are conserved at the corresponding positions of DREB subfamily [3]. The two conserved amino acids are located on the β-sheet in the AP2/ERF domain, which is important for binding to the target DNA sequences [1]. The AP2/ERF domain of DREB and ERF sub family were well-conserved among *Arabidopsis*, rice and maize [3–4]. Remarkably, all DREB and ERF subgroups were completely conserved in Val-14 and Ala-14 acid residues, respectively. However, in the present study, seventeen *DREB* gene members were conserved in Glu-19, meanwhile fifty ERF subfamily gene members were completely conserved in Asp-19, only HvERF1.6/2.12 was not conserved in Asp-19 amino acid residues. These conserved amino acid residues probably indicate crucial roles for DREB/ERF sub family genes involved in different forms of physical interaction with DNA [1].

### Expression analysis indicated *HvAP2/ERF* genes may play important roles during plant growth, abiotic stress and hormone response

Tissue-specific expression data at a given developmental stage is useful for identifying genes involved in defining precise nature of individual tissues. In the present study, the expression pattern of one hundred and seven *HvAP2/ERF* genes were detected by RNA sequencing, it was contribute to investigate the function of the *HvAP2/ERF* genes in barley. Remarkably, *HvAP2-12* gene also named as *Cleistogamy 1* (*Cly1*)/*HvAPETALA2* (*HvAP2*), which was an ortholog of *AP2* (AT4G36920.1), *TOE3* (AT5G67180.1) and rice AP2-like gene Os04g0649100 [55–56]. In situ RNA hybridization indicated that the *Cly1* transcript was detected in the lodicule up to the stamen primordium stage [55]. Another gene *HvDREB2*.*2* also named *Nud* (*Nudum*), which control covered/naked caryopsis in barley and was expressed in the caryopsis at two weeks after anthesis rather than in hulls or leaves [57]. In the present study, *HvAP2-12* displayed relative high expression level in developing tillers at six leaf stage, the transcript of *HvDREB2*.*2* gene was only detected in developing grains (5 DPA). Therefore, more sophisticated specificity expression analysis is helpful to parse the function of *HvAP2/ERF* genes.

Plants were involved in adverse environmental stresses in their natural environments. On the molecular levels, they have evolved a wide range of mechanisms to cope with them. In plants, the genes respond to drought and high-salt stress involved in two ABA dependent and two ABA-independent signal transduction pathways [16–17]. A phylogenetic tree of the *HvDREB1s* and *HvDREB2s* proteins and their orthologs from *Arabidopsis*, maize and rice was constructed (S4 Fig). *HvDREB2*.*9* was an orthology gene of *OsDREB2A* with improving drought and salt stress tolerance in rice and *Arabidopsis*, which had no obvious difference after drought, high-salt and ABA treatment although promoter contained multiple ABREs in barley. The expression level of *HvDREB1*.*1* and *HvDREB1*.*2* were decreased after 6 h, on the contrary, *OsDREB1F* as an orthology gene of *HvDREB1*.*1* and *HvDREB1*.*2* was increased after drought, high-salt and ABA treatment [20]. Remarkably, some *HvDREB1s* and *HvDREB2s* may be displayed different functions in plants. *HvDREB1*.*8* was orthology gene of *OsDREB1A* which was induced by drought, high-salt stress and also ABA treatment in root, the expression level of *HvDREB1*.*8* was higher in CM72 than Gairdner. In addition, *HvDREB1*.*3*/*1*.*4*/*2*.*8* was increased under drought and salt stress, as well as *HvDREB2*.*8* response to ABA treatment. Further analysis revealed that *HvDREB1*.*3* and *HvDREB1*.*4* were identified as homologous to *OsDREB1C* genes in rice whereas *OsDREB1C* showed constitutive expression [21]. *HvDREB2*.*8* was a homologous of maize *ZmDREB2*.*7* which enhanced tolerance to drought stress by overexpressing *ZmDREB2*.*7* in transgenic *Arabidopsis* [4] (S4 Fig). However, *HvDREB2*.*8* displayed higher expression level in Gairdner than CM72 except in leaf under salt stress. An ABRE functions as a *cis*-acting DNA element involved in ABA-regulated gene expression, which was observed in promoter regions of dehydration-inducible genes. In the present study, promoter analysis revealed that *HvDREB1*.*3/1*.*4/1*.*8/2*.*8* contained multiple ABREs, suggesting that these genes are involved in ABA-dependent response under drought and high-salt conditions (S12 Table). Therefore, gene expression analysis of *HvDREB1s* and *HvDREB2s* should help us to investigation the molecular mechanisms of environment adaptability of plant under abiotic stress and hormone response, the function of differentially expressed genes between Gairdner and CM72 should be further investigated in relation to abiotic stress and hormone response.

## Supporting Information

S1 Fig Gene structure analysis of the *HvAP2/ERF* genes. (TIF)

S2 Fig Conserved motifs of HvAP2/ERF proteins in barley. (TIF)

S3 Fig Alignment of conserved motifs of the DREB and ERF sub family in barley. (TIF)

S4 Fig Phylogenetic tree of canonical *DREB1* and *DREB2* genes in *Arabidopsis*, maize, rice and barley. The phylogenetic tree was constructed based on the sequence alignments of seventy-four full-length *DREB 1* and *DREB*2 genes from four species. The gene ID and names are illustrated in red for barley; black for rice; blue for *Arabidopsis*; and green for maize. The gene names were used in the present study according to published data [1, 4, 46, 58]. Bootstrap values from 1,000 replicates were indicated at each node and the scale represents branch lengths.
(TIF)

S1 Table Primers used in this study. (XLS)

S2 Table Putative 121 *HvAP2/ERF* genes in barley. (DOCX)

S3 Table The information of *HvAP2*/*ERF* genes in barley. (XLS)

S4 Table Summary of functional domains present in the HvAP2/ERF proteins. (XLS)

S5 Table The Ka/Ks ratios for tandemly duplicated *HvAP2/ERF* genes. (XLS)

S6 Table The Ka/Ks ratios for segmentally duplicated *HvAP2/ERF* genes. (XLS)

S7 Table The Ka/Ks ratios for orthologous HvAP2/ERF proteins between barley and maize. (XLSX)

S8 Table The Ka/Ks ratios for orthologous HvAP2/ERF proteins between barley and rice. (XLSX)

S9 Table The Ka/Ks ratios for orthologous HvAP2/ERF proteins between barley and *Brachypodium*. (XLSX)

S10 Table The Ka/Ks ratios for orthologous HvAP2/ERF proteins between barley and foxtail millet. (XLSX)

S11 Table The fold change values of *HvDREB1s* and *HvDREB2s* genes in root and leaf tissues between Gairdner and CM72. (XLSX)

S12 Table *Cis*-regulatory elements analysis of the promoter region of *HvDREB1s* and *HvDREB2s* genes (XLSX)
