# Supplementary figures and images for: Genome-Wide Analysis of APETALA2/Ethylene-Responsive Factor (AP2/ERF) Gene Family in Barley (Hordeum vulgare L.)
Source: PLoS One. 2016 Sep 6;11(9):e0161322. doi: 10.1371/journal.pone.0161322 (PMC5012588; doi:10.1371/journal.pone.0161322)

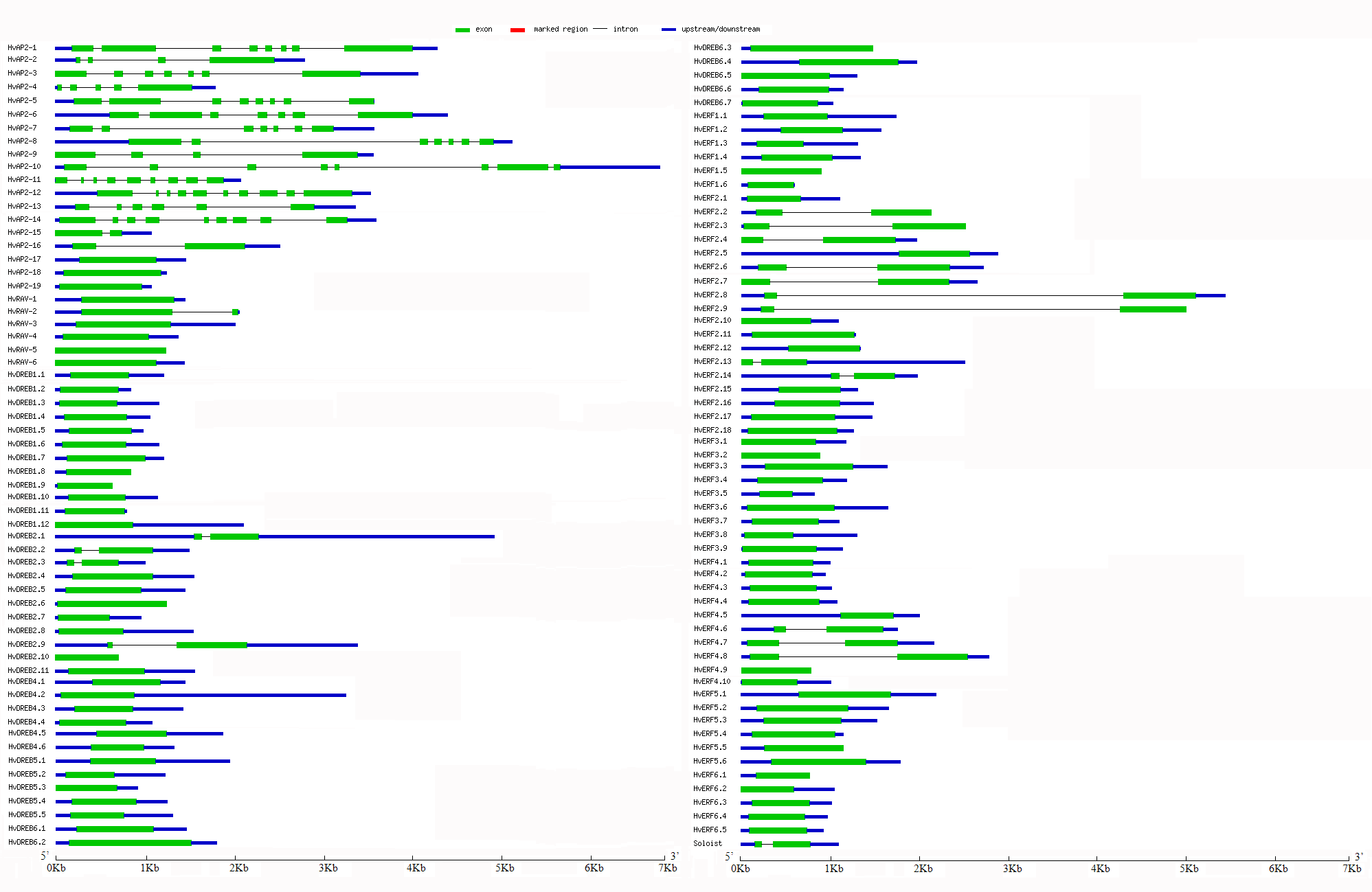

Supplement: S1 Fig — (TIF) [file pone.0161322.s001.tif]

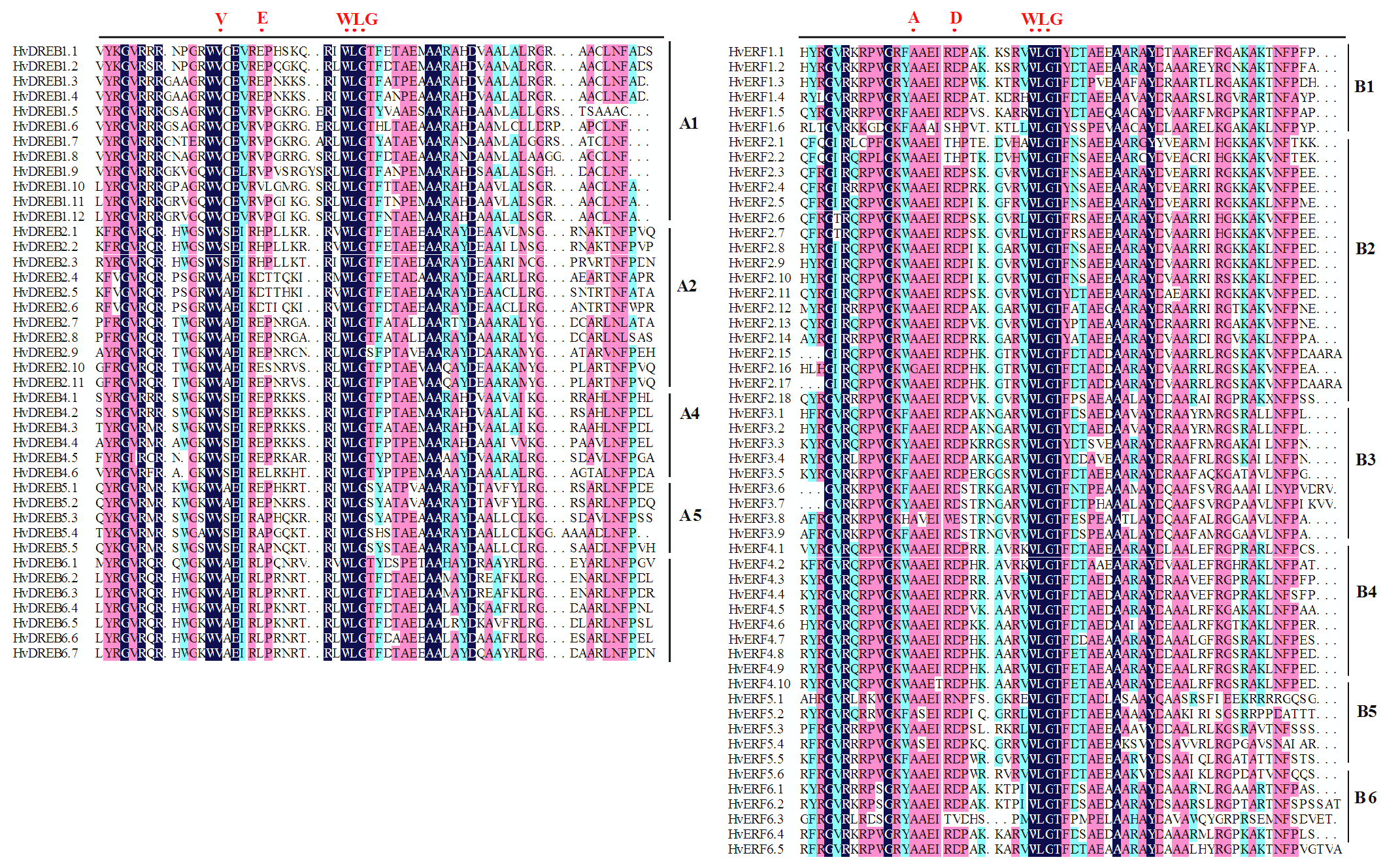

Supplement: S2 Fig — (TIF) [file pone.0161322.s002.tif]

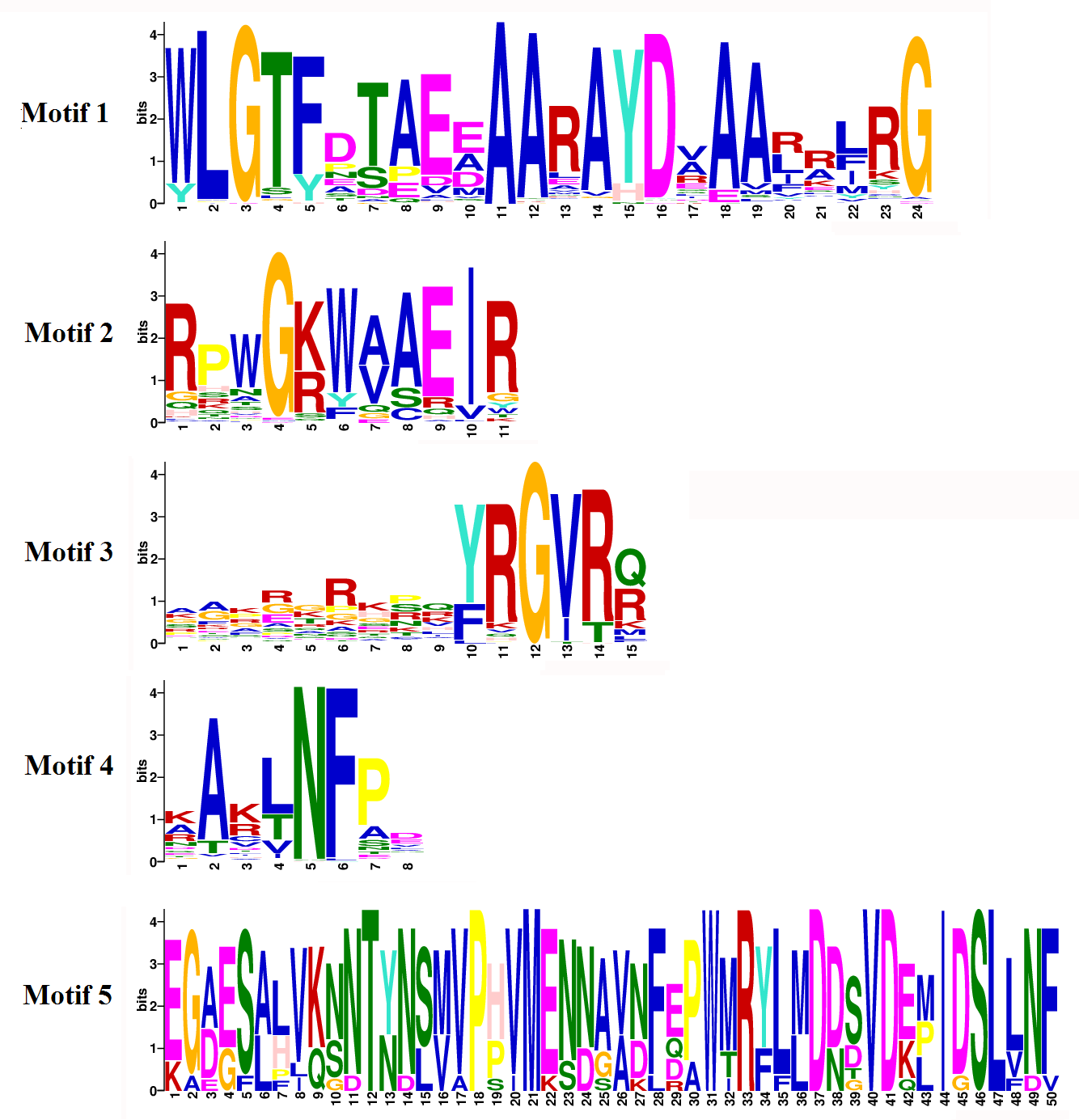

Supplement: S3 Fig — (TIF) [file pone.0161322.s003.tif]

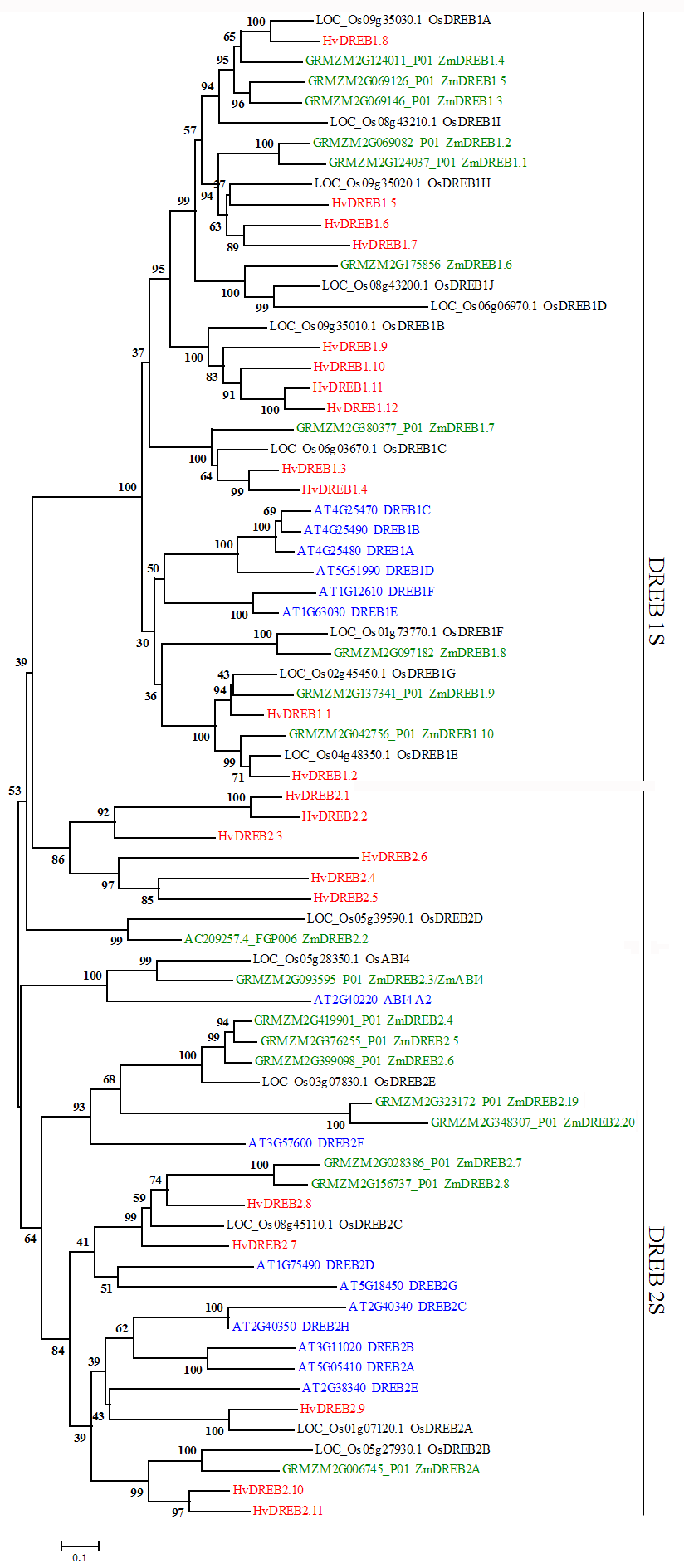

Supplement: S4 Fig — The phylogenetic tree was constructed based on the sequence alignments of seventy-four full-length DREB 1 and DREB2 genes from four species. The gene ID and names are illustrated in red for barley; black for rice; blue for Arabidopsis; and green for maize. The gene names were used in the present study according to published data [1, 4, 46, 58]. Bootstrap values from 1,000 replicates were indicated at each node and the scale represents branch lengths. (TIF) [file pone.0161322.s004.tif]
