# Supplementary material for: Genome-Wide Analysis of APETALA2/Ethylene-Responsive Factor (AP2/ERF) Gene Family in Barley (Hordeum vulgare L.)
Source: PLoS One. 2016 Sep 6;11(9):e0161322. doi: 10.1371/journal.pone.0161322 (PMC5012588; doi:10.1371/journal.pone.0161322)
Supplement: S2 Table — (DOCX) [file pone.0161322.s006.docx]

>HvAP2-1

>Protein

MASANNWLGFSLSGQGNHPQPHQNGSPAAAAIDVSGAGDFYGLQAQTAPDAHLGMSGLRADANYGVMDAFNGGNQETQDWAMRGLDYHGGSSELSMLVGSSGGRMTVDDGEAPKLEDFLGGNSFSDVQDHAGSYLFSSGSAMGSGAAAGSHGVQGRGGSTIELSMIKTWLRNDNNQAQHDQEMSADASATSYACSGAPGSTGNGVGVANSRGQGLALSMSMGSNSHPQMPVVPAAVGTESTSSENKRVDSPSAGTADAVQRKSIDTFGQRTSIYRGVTRHRWTGRYEAHLWDNSCRREGQTRKGKQVYLGGYDKEDKAARAYDLAALKYWGTTTTTNIPISTYEKEIEEMKHMTRQEYIAYLRRNSSGFSRGASKYRGVTRHHQQGRWQARIGRVAGNKDLYLGTFTTEEEAAEAYDIAAIKFRGLNAVTNFEMSRYDVKSILEGSTLPVGGAARRLKEAAELAEAGVWRAEDGSIVSHLQADAMAGYHHGWPTSIAFGSHQQQQSAAQLALHYPYGVGGQARGWCKPEQDAVIEAAHGGQDLQELHLGSGGSTHNFFQPASRTAVYGNGGGGAWYQGLGGNAYMMPVGTVVDADQGHSGSTATTEEGRLVGYGAEAGVDPYAAMRRAYELSQGSSSVSVAKVADGYSNNWSSPFNGMG

>cDNA

TGTCCGGGCGAGAGATAGGATTGTTTTCCCCTGTCCTCACTTCTTCCCAGCCAGCCACGAGCCACCTCCCACAAACCAATGCTAGCTTTGACTGCCCTGATCAGAAGAATCTAGCTAGCGACCAGACCACCACTGCCATCTCTTCCAAATCGACCACTGATCGATAAAAATACACGAGCTCCATCTCCACAATGGCTTCGGCGAACAACTGGCTGGGCTTCTCGCTCTCCGGCCAAGGGAACCACCCACAGCCCCACCAGAATGGCTCACCGGCCGCCGCGGCCATTGACGTCTCCGGTGCAGGCGACTTCTATGGCCTGCAAGCCCAGACGGCGCCGGATGCGCACCTTGGCATGTCTGGCCTCAGGGCCGACGCCAACTACGGCGTCATGGATGCCTTCAACGGAGGCAACCAAGAAACCCAAGATTGGGCAATGAGGGGTTTGGACTACCACGGCGGCTCCTCCGAGCTGTCGATGCTAGTCGGCTCGAGCGGCGGGAGGATGACGGTGGACGACGGCGAGGCGCCGAAGCTCGAGGACTTCCTCGGCGGCAACTCCTTCTCCGACGTGCAGGACCACGCCGGCAGCTACCTGTTCTCGTCAGGGAGCGCGATGGGCAGCGGAGCCGCTGCTGGTTCGCACGGCGTCCAAGGCCGTGGCGGCAGCACCATAGAACTGTCCATGATCAAGACATGGCTCCGGAACGACAATAACCAGGCGCAGCATGACCAGGAGATGAGCGCCGACGCGAGCGCGACCAGCTATGCGTGCTCCGGCGCGCCAGGGAGCACCGGCAACGGCGTGGGCGTGGCGAACTCGCGTGGGCAGGGCCTAGCGCTCTCGATAGCATGGGGTCGAACTCGCACCCGCAGATGCCAGTGGTCCCGGCTGCGGTGGGGACGGAGAGCACGTCATCAGAGAACAAGCGGGTGGATTCGCCGAGCGCCGGCACGGCGGACGCCGTCCAGAGGAAATCCATCGACACCTTCGGGCAAAGGACCTCGATCTACAGAGGTGTAACAAGGCATAGATGGACAGGGCGGTACGAGGCGCATCTGTGGGACAATAGTTGCAGGAGAGAGGGGCAAACTCGCAAGGGGAAACAAGTTTATCTGGGCGGTTATGACAAAGAAGACAAGGCAGCTAGGGCTTATGATTTGGCAGCTCTAAAATATTGGGGCACAACCACAACAACAAATATCCCAATAAGTACTTACGAGAAGGAGATAGAAGAAATGAAACACATGACTAGGCAGGAGTACATTGCGTATCTTAGGAGGAATAGCAGTGGTTTTTCTCGTGGCGCGTCAAAATACCGCGGTGTAACCAGGCATCATCAGCAAGGAAGATGGCAAGCAAGGATAGGGAGAGTCGCGGGCAACAAGGATCTCTACCTCGGCACCTTCACGACCGAGGAGGAGGCCGCGGAGGCGTACGACATCGCCGCCATCAAGTTCCGCGGCCTCAACGCCGTCACCAACTTCGAGATGAGCCGCTACGACGTCAAGAGCATTCTGGAGGGCAGCACGCTGCCGGTCGGCGGAGCGGCCAGGCGCCTCAAGGAGGCGGCCGAGCTCGCCGAGGCCGGCGTGTGGCGGGCGGAGGACGGCAGCATCGTCTCGCACCTGCAGGCCGACGCCATGGCGGGCTACCACCACGGCTGGCCCACGTCCATCGCTTTCGGCAGCCACCAGCAGCAGCAGTCCGCGGCGCAGCTCGCCCTGCACTACCCGTACGGCGTTGGCGGGCAGGCCCGCGGATGGTGTAAGCCGGAGCAGGACGCGGTGATCGAGGCCGCGCACGGCGGGCAGGACCTCCAGGAGCTGCACCTGGGAAGCGGCGGCAGCACCCACAACTTCTTCCAGCCGGCGTCAAGGACAGCGGTCTACGGCAACGGCGGGGGCGGCGCCTGGTACCAAGGCCTCGGCGGCAACGCGTACATGATGCCGGTGGGCACGGTGGTGGACGCCGACCAGGGACACAGCGGCAGCACGGCAACTACGGAGGAGGGGAGGCTCGTGGGCTACGGCGCCGAGGCTGGCGTCGACCCGTACGCGGCCATGAGGCGCGCCTACGAGCTGTCCCAGGGCTCGTCGTCCGTGAGCGTCGCCAAGGTGGCGGACGGCTACTCCAACAACTGGAGCTCGCCGTTCAATGGCATGGGGTGATCGGGCCCCCTATGTGTTACTAATCAACTGCCACACTTGTTGTAACGTACGTTGCGCACGAGTAACCGCAGACCAATTAAGTTCTGTAATTTTAGGATAGTGATCTAGAGCAGTGCATGAGCACGAGCGGTGCCTTCTGAAAGGTAGTAATGGCCAGCGACGGGTGAAGTAGGAGGACTCGTTGTAAATCACCATCGCTGCGCCGTTAGTTTTGGGGATTTCAGAAGGAAGAAATCAAGCTAATCTAGCTATCAAACAAACTTCCATGGCTACTTCATTC

>CDS

ATGGCTTCGGCGAACAACTGGCTGGGCTTCTCGCTCTCCGGCCAAGGGAACCACCCACAGCCCCACCAGAATGGCTCACCGGCCGCCGCGGCCATTGACGTCTCCGGTGCAGGCGACTTCTATGGCCTGCAAGCCCAGACGGCGCCGGATGCGCACCTTGGCATGTCTGGCCTCAGGGCCGACGCCAACTACGGCGTCATGGATGCCTTCAACGGAGGCAACCAAGAAACCCAAGATTGGGCAATGAGGGGTTTGGACTACCACGGCGGCTCCTCCGAGCTGTCGATGCTAGTCGGCTCGAGCGGCGGGAGGATGACGGTGGACGACGGCGAGGCGCCGAAGCTCGAGGACTTCCTCGGCGGCAACTCCTTCTCCGACGTGCAGGACCACGCCGGCAGCTACCTGTTCTCGTCAGGGAGCGCGATGGGCAGCGGAGCCGCTGCTGGTTCGCACGGCGTCCAAGGCCGTGGCGGCAGCACCATAGAACTGTCCATGATCAAGACATGGCTCCGGAACGACAATAACCAGGCGCAGCATGACCAGGAGATGAGCGCCGACGCGAGCGCGACCAGCTATGCGTGCTCCGGCGCGCCAGGGAGCACCGGCAACGGCGTGGGCGTGGCGAACTCGCGTGGGCAGGGCCTAGCGCTCTCGATGAGCATGGGGTCGAACTCGCACCCGCAGATGCCAGTGGTCCCGGCTGCGGTGGGGACGGAGAGCACGTCATCAGAGAACAAGCGGGTGGATTCGCCGAGCGCCGGCACGGCGGACGCCGTCCAGAGGAAATCCATCGACACCTTCGGGCAAAGGACCTCGATCTACAGAGGTGTAACAAGGCATAGATGGACAGGGCGGTACGAGGCGCATCTGTGGGACAATAGTTGCAGGAGAGAGGGGCAAACTCGCAAGGGGAAACAAGTTTATCTGGGCGGTTATGACAAAGAAGACAAGGCAGCTAGGGCTTATGATTTGGCAGCTCTAAAATATTGGGGCACAACCACAACAACAAATATCCCAATAAGTACTTACGAGAAGGAGATAGAAGAAATGAAACACATGACTAGGCAGGAGTACATTGCGTATCTTAGGAGGAATAGCAGTGGTTTTTCTCGTGGCGCGTCAAAATACCGCGGTGTAACCAGGCATCATCAGCAAGGAAGATGGCAAGCAAGGATAGGGAGAGTCGCGGGCAACAAGGATCTCTACCTCGGCACCTTCACGACCGAGGAGGAGGCCGCGGAGGCGTACGACATCGCCGCCATCAAGTTCCGCGGCCTCAACGCCGTCACCAACTTCGAGATGAGCCGCTACGACGTCAAGAGCATTCTGGAGGGCAGCACGCTGCCGGTCGGCGGAGCGGCCAGGCGCCTCAAGGAGGCGGCCGAGCTCGCCGAGGCCGGCGTGTGGCGGGCGGAGGACGGCAGCATCGTCTCGCACCTGCAGGCCGACGCCATGGCGGGCTACCACCACGGCTGGCCCACGTCCATCGCTTTCGGCAGCCACCAGCAGCAGCAGTCCGCGGCGCAGCTCGCCCTGCACTACCCGTACGGCGTTGGCGGGCAGGCCCGCGGATGGTGTAAGCCGGAGCAGGACGCGGTGATCGAGGCCGCGCACGGCGGGCAGGACCTCCAGGAGCTGCACCTGGGAAGCGGCGGCAGCACCCACAACTTCTTCCAGCCGGCGTCAAGGACAGCGGTCTACGGCAACGGCGGGGGCGGCGCCTGGTACCAAGGCCTCGGCGGCAACGCGTACATGATGCCGGTGGGCACGGTGGTGGACGCCGACCAGGGACACAGCGGCAGCACGGCAACTACGGAGGAGGGGAGGCTCGTGGGCTACGGCGCCGAGGCTGGCGTCGACCCGTACGCGGCCATGAGGCGCGCCTACGAGCTGTCCCAGGGCTCGTCGTCCGTGAGCGTCGCCAAGGTGGCGGACGGCTACTCCAACAACTGGAGCTCGCCGTTCAATGGCATGGGGTGA

>DNA

CACGATGAAGGTTAGTTTAGCCCCTTTCATTTGACTTCATATTTGCACTTTCATTTCTTCTGACCTTCGTGTTTGCTTGGTAAACTAACATACAACACGTTTTTGACTCCTTGGTATCCGGCACGGATCCTGTAATGGAACCACCACCACCACCTACTGGACCACAACTAGTGTTGGCTACGTTTGAAGAATTTCTGGCACAACACTGTCCGACTCTGGTTTGTACGTCCCCAAACTATTCAACCACTACTTATCGTTTATTCAACACAATCATATATCTCTTTAACATCTCTTTTCAAACATGCAGGGAACCGGTGGATCATCCGTTGGTCGTGGTGCCAATTATGGCATCACTCCCGAGTCACGGAGCCCGATCAATCCGATCAACCGAGGATGTGGCGATGGCGGTCTTGACGGTAGCGGTGTCGGTAGCAGTGGTGACCTGGGCTTTGGCGGTCTTGGCGGTGACGAACTTGGTGGTGGACGTCATGGCTTAAGCATGTGGGTGGTGGTGGTGGTAACTTCTATCTTTCATGCTTCTCTATTATGGACTTATGTTGTATTGTTCGACTTATGTTGTGTTGTTTTGACTTATGTTGTGTTGATTCGACTTATGTTGTGATGATAATACTTATCTTGTCGTGATAATGATGTGGATATGTGATATGTATGTGATGTGTATATGATATGTGATATATATGTGTTGTGTATTCGAATTCAACATGAGCAGGAAATTAAAAAAACAGGAGATATGTTGACAGCATAGCCGTTGGCGTAGCCATGCGCCAGGAAGCACCAAAAGCCGCCACATGGCAGAGGTACGCCGACGTCCTCCTCGTCGATGCAGCTCTCGATATATGCCGACGGCTAAGTCGTTGGGGTAGCCGCACGCCGGGACAAGTGAGGGACCGCCACATGCCATATTTATGCCAACGACCTCCCCGTTGGCTCAGCTCTGGAGATATGTCGACGTCTATGCCGTCAGCATAGGGGTGCCACGTGGCTGGCTACGGTTAGGTGTCGCCCGTCGAGGGTGCCGTCATCCGCCGTCATGTGGCAAAGGTTGCCGACGGTCTATCTATGTCGACTGCTGTATGGAGGGCGTTGGCGTAGGTACCTATGCCGATGACTTTTCTATGCCGACGAACCTGGCCGGGTACGCCGACGTTCGCGTTCCCGACCGTGCTATGCCGACGGTGGCCGTCGGCATACCCCTACGCTGATGCGGTATGGCCCTACGCTGACGGTCTTCGGCCGTCGGCATATCCCTTCAGTCTGGTAGTGATGATATGTTGCATGTGTTTGGCCGTTTTGGACACCTTCAATATTTTGAACTTTATTGCATCTTTAGGGTATTAAGTTAAATTTCAGGTCTATGAAGTTCTGTAGATTATGCCAAAATATCAAGTGTGTTGCAGTGATAATTTGTTGAATTATAGTACTGCTATGCTAATAAATTGATGGAAATTGCATGCTATGGATCTCAAGCATGCACTTTTGGCATTCAAGAATGGTCAACACATAAGTCTCAGCTTTGTACTACTGACACAATGGAGTACGTATAGTAATATCCCTCAGAAAAGTACTCCTACATACAATCGAATCTACTGTAGGTGCAAAAGAAAATATACAATAGAATCGATTTGCATGCACGTTGGAATTCAGAATGTTTTCAATACTGGAAAAGTCGCTACCTGGAATCACACGTTGCATCAGCAGGTAGCTAGAAGTAGCACCCACTAGGAGGAGGCTAGCTTACCTACTCATGCTGGTATAAGTACAGGGCCTGCTTCCCCAGCATTTTCCTCCTGTCCGGGCGAGAGATAGGATTGTTTTCCCCTGTCCTCACTTCTTCCCAGCCAGCCACGAGCCACCTCCCACAAACCAATGCTAGCTTTGACTGCCCTGATCAGAAGAATCTAGCTAGCGACCAGACCACCACTGCCATCTCTTCCAAATCGACCACTGATCGATAAAAATACACGAGCTCCATCTCCACAATGGCTTCGGCGAACAACTGGCTGGGCTTCTCGCTCTCCGGCCAAGGGAACCACCCACAGCCCCACCAGAATGGCTCACCGGCCGCCGCGGCCATTGACGTCTCCGGTGCAGGCGACTTCTATGGCCTGCAAGCCCAGACGGCGCCGGATGCGCACCTTGGCATGTCTGGCCTCAGGGCCGACGCCAACTACGGCGTCATGGATGCCTTCAACGGAGGCAACCAAGAAACCCAAGGTGACCTGATCCTGATGAGCTACTGTACATGTACTACTTCAGCTTCAGCGTATACCTTTGTCTTGTCTAGCCTCTTATGTTGGATGCCATCATGGGGTTACAGATTGGGCAATGAGGGGTTTGGACTACCACGGCGGCTCCTCCGAGCTGTCGATGCTAGTCGGCTCGAGCGGCGGGAGGATGACGGTGGACGACGGCGAGGCGCCGAAGCTCGAGGACTTCCTCGGCGGCAACTCCTTCTCCGACGTGCAGGACCACGCCGGCAGCTACCTGTTCTCGTCAGGGAGCGCGATGGGCAGCGGAGCCGCTGCTGGTTCGCACGGCGTCCAAGGCCGTGGCGGCAGCACCATAGAACTGTCCATGATCAAGACATGGCTCCGGAACGACAATAACCAGGCGCAGCATGACCAGGAGATGAGCGCCGACGCGAGCGCGACCAGCTATGCGTGCTCCGGCGCGCCAGGGAGCACCGGCAACGGCGTGGGCGTGGCGAACTCGCGTGGGCAGGGCCTAGCGCTCTCGATGAGCATGGGGTCGAACTCGCACCCGCAGATGCCAGTGGTCCCGGCTGCGGTGGGGACGGAGAGCACGTCATCAGAGAACAAGCGGGTGGATTCGCCGAGCGCCGGCACGGCGGACGCCGTCCAGAGGAAATCCATCGACACCTTCGGGCAAAGGACCTCGATCTACAGAGGTGTAACAAGGTTAGCTAGCTTAGATCTTCTTGGCTTCTGCTAGTGACCTAGAGGTGTTCAATTTGCTTCTTTGGCTCCTGCAGATGCCTGATCTTCATTGCCCATTAGTTTCTTTCTTTCTCCCTCTCGATTGCACACTAATCTTTCTTCTTTTCGATGCCATTTTTGAGGGGTCGATCTGTCTGCAACGCATATATACGATTAGAAATAGCTAAGAAAAAAATGGATTTTGAGAACCTGGGGTACACCGTACACTACAGATCGAGTCTAGAACCCACTTTTTAAAACTTGACTATAACTTGTTTATAACTTCTGTAAAATGAGGAACAAAAATCATGATCATTCTAGTATTTTGCGAGCATCATAATGCAAACGAAGAGTTAACTAATTAATAGCAGTATGCTACTTCACTTGTGCTATGCAACTTTCCACTTCACTTAGCCTTTAGGCAACACAATGCTTGTAATATAAGGTGATTCTCACAAAATGCACTAGTGGTACCATAGTTGACTAGGTAGAGGGGGGAAAGGGGGACACATATGCATAGAATTTCCATGATCTGCGTAGGGTTGTTTGGCCAATAGAAATGTCGCTTTGATGCTTCAAAGCTGATTTTACTTTATGTGTAATTTGTTGTTAATTCTACCATCAGGCATAGATGGACAGGGCGGTACGAGGCGCATCTGTGGGACAATAGTTGCAGGAGAGAGGGGCAAACTCGCAAGGGGAAACAAGGTATAATGAGATTGTTCATATATGAGGGGGGTTGTGTTCATGTAGTATGTATATACATATATAGTTCAATCATTAGCTAGATCTTGATTTATTTTTTTGGCAACTTCTATGGGATGTTCTTAAGCATGTGCTAATTAAGCTTCCTTCTTTCTGTTCTGTCAATGCTGACATCTCCTGGGACATGACACTGCAAATACTGATCATGGGAATGCAGTTTATCTGGGTAAGAATTTGTAACTGATCTTTCGTTCAAGTATTTGATGGACAAATGTTCTGATGCTTCCTAGACCTATGTAGCACATTAATGTCTTATCGGTGAACATTTGCGTAGGCGGTTATGACAAAGAAGACAAGGCAGCTAGGGCTTATGATTTGGCAGCTCTAAAATATTGGGGCACAACCACAACAACAAATATCCCAGTAAGGAAAAATCTATAATAGATTGTTCTGTTTCCTTCTGTATGGGCAGCAGCTGATCCTGTTTTCTTCTTTCGCCTATAAAAATTGTGCAGATAAGTACTTACGAGAAGGAGATAGAAGAAATGAAACACATGACTAGGCAGGAGTACATTGCGTATCTTAGGAGGTATGCATTCCAAGTTCTGTTTTACAACATCACATGTACATGTGTTGAAAGATTGCACGGTGGCTAACAATCACATGGATGCATATGCATGATCATTCAACTGCAGGAATAGCAGTGGTTTTTCTCGTGGCGCGTCAAAATACCGCGGTGTAACCAGGTAAAAATGTACTGTTTACATTGCGTAAAAGTTGGTAGCGAAGCGATCTAAATATCTTATGGTTTGACAGGCATCATCAGCAAGGAAGATGGCAAGCAAGGATAGGGAGAGTCGCGGGCAACAAGGATCTCTACCTCGGCACCTTCAGTAAGCCGCATAAACTATCACATCAAAGCGAAATTGCTTTCTCAGCTAGCTGAAGCACTAGTTTAATTACTGTACTGTTATAGTAATCCTACAGTAGTAGTAGATGTGCTGTGCTCTCAAGAACCAAGTCATGAAGCCCTAATTATTTACTGGTAGTACTCCAGCTCACGAAATCTCGTAGGACGCAAGCGTTGCAGTATTTTTATGCGTTCAAATGGCGCTTAAGATAGTGGAGTACGTACACCATTAACTATGTCCGAAATGTTCCTTTCCAAGCTTGTGTCTCGGAGCTGTCTGAGGAAACCGGGGCATGCGGTCAAGAGCCGGTTTGAATATCCCTAGCGCCCTGCTTGAGCTTCGCGCCGTCCCAGCGCGCGTTGCATGCGCCACCGCGCCGTGCGTGCGTGCGTGCGTGCATGCATGCATGCAACGCGCAATGCGCTCCACTGGCTGGTCAGTTATTAGGTAGACACACGCGTTGGTGAGCTAACCCTAATCATGCGCGTGTTGTGCAGCGACCGAGGAGGAGGCCGCGGAGGCGTACGACATCGCCGCCATCAAGTTCCGCGGCCTCAACGCCGTCACCAACTTCGAGATGAGCCGCTACGACGTCAAGAGCATTCTGGAGGGCAGCACGCTGCCGGTCGGCGGAGCGGCCAGGCGCCTCAAGGAGGCGGCCGAGCTCGCCGAGGCCGGCGTGTGGCGGGCGGAGGACGGCAGCATCGTCTCGCACCTGCAGGCCGACGCCATGGCGGGCTACCACCACGGCTGGCCCACGTCCATCGCTTTCGGCAGCCACCAGCAGCAGCAGTCCGCGGCGCAGCTCGCCCTGCACTACCCGTACGGCGTTGGCGGGCAGGCCCGCGGATGGTGTAAGCCGGAGCAGGACGCGGTGATCGAGGCCGCGCACGGCGGGCAGGACCTCCAGGAGCTGCACCTGGGAAGCGGCGGCAGCACCCACAACTTCTTCCAGCCGGCGTCAAGGACAGCGGTCTACGGCAACGGCGGGGGCGGCGCCTGGTACCAAGGCCTCGGCGGCAACGCGTACATGATGCCGGTGGGCACGGTGGTGGACGCCGACCAGGGACACAGCGGCAGCACGGCAACTACGGAGGAGGGGAGGCTCGTGGGCTACGGCGCCGAGGCTGGCGTCGACCCGTACGCGGCCATGAGGCGCGCCTACGAGCTGTCCCAGGGCTCGTCGTCCGTGAGCGTCGCCAAGGTGGCGGACGGCTACTCCAACAACTGGAGCTCGCCGTTCAATGGCATGGGGTGATCGGGCCCCCTATGTGTTACTAATCAACTGCCACACTTGTTGTAACGTACGTTGCGCACGAGTAACCGCAGACCAATTAAGTTCTGTAATTTTAGGATAGTGATCTAGAGCAGTGCATGAGCACGAGCGGTGCCTTCTGAAAGGTAGTAATGGCCAGCGACGGGTGAAGTAGGAGGACTCGTTGTAAATCACCATCGCTGCGCCGTTAGTTTTGGGGATTTCAGAAGGAAGAAATCAAGCTAATCTAGCTATCAAACAAACTTCCATGGCTACTTCATTC

>HvAP2-2

>Protein

MASANNWLGFSLSGQGNHPQPHQNGSPAAAAIDVSGAGDFYGLQAQTAPDAHLGMSGLRADANYGVMDAFNGGNQETQDWAMRGLDYHGGSSELSMLVGSSGGRMTVDDGEAPKLEDFLGGNSFSDVQDHAGSYLFSSGSAMGSGAAAGSHGVQGRGGSTIELSMIKTWLRNDNNQAQHDQEMSADASATSYACSGAPGSTGNGVGVANSRGQGLALSMSMGSNSHPQMPVVPAAVGTESTSSENKRVDSPSAGTADAVQRKSIDTFGQRTSIYRGVTRHRWTGRYEAHLWDNSCRREGQTRKGKQGIMRLFIYEGGCVHVVCIYIYSSIIS

>cDNA

GGTGGCTATGACAAGGAGGACAAGGCAGCAAGGGCTTATGATTTGGCAGCACTCAAGTATTGGGGTACAACAACAACAACAAATTTCCCAATTAATACCTACGAAAAAGAGGTGGATGAAATGAAACATATGACTAGGCAGGAATACATTGCATACTTAAGAAGGAATAGCAGCGGATTTTCTCGCGGTGCGTCAAAATATCGTGGTGTAACTAGGCACCATCAGCATGGGAGATGGCAAGCAAGAATTGGGCGGGTTGCAGGAAACAAGGATCTCTACTTAGGCACCTTCAGCACCGAGGAGGAGGCGGCGGAGGCCTACGATATCGCGGCGATCAAGTTCCGTGGACTCAACGCCGTCACCAACTTCGACATGAGTCGGTACGACGTCAAGACCATCCTCGAGAGCAGCACCCTACCGGTGGGTGGCGCGGCGAGGCGCCTCAAGGAGGCCGCTGATCATCCAGAGGCTGGCGCCACGATCTGGCGGGCCGGCATGCACGGCGGCGTCATCTCCCAGCTGACCGACGTCGGGATGGGCGCGTACGCGTCGTACCCCCACGGCTGGCCGACCATCGCATTTCAGCAGCCGTCGCCGCTCTCGGTGCACTACCCATACGTCGCCCAGCCGGCCCGCGGGTGGTGCAAGCCCGAGCAGGACGCCACCGTCGCCGCGCAAAACCTGCAGGACCTCCAGCAGCTGCACCTCGGGACCGCCGCTCACAACTATTTCCAGGCATCGTCGAGCTCCACGGTGTACAACGGCGGCGGGTACCAGCAGGGCACCGGTGGCAACGCCTTCTTGATGCCGGCTACCGCCGTCATGGATGAACAGGGGCACAGCAGCACTGCCACCAACCTGGGAAGCACCTGCAGCTACGGGGATGAAGAGGGGAAGCTCAATATTGGGTACGATGCCATGGCGGCGATGGCAAGCACCGCAGCCGACCCATACGCGGCGGCGTCAACTGTGAGTATCGCGAGGGCGAACGGGTACTCCAACCACTGGAGCTCGCCGTTCAATGGCATGTGATGATGGTGAAGAGGGCAGAAGCTAGTAATAGCGGGATTACACTTAACTAGCTAGAGCATAATCCAAGTTAGGTCAGGGGTCTTGCTCGTTGTTGCGTTTCAGCTCGGGGTTCCGGCTAGCTCTTCTTGGCCGGGTGAATGACACTCGTAAAGGTGCTGGGCATGCATGCCTTCTGGAAGCTGGTAATGGCAAGTTAGGATAAGTTAAGCTTGTGTTGTTGTAAGTTATCCTAACTTGCTGTTAGTTTTGGGTAGTTTCAGAAGATCAAGTTTAATCTCTAGAGGACAAAAGCTGATCGTGCGATGGCAATAACTTCATAATTCCAG

>CDS

ATGAAACATATGACTAGGCAGGAATACATTGCATACTTAAGAAGGAATAGCAGCGGATTTTCTCGCGGTGCGTCAAAATATCGTGGTGTAACTAGGCACCATCAGCATGGGAGATGGCAAGCAAGAATTGGGCGGGTTGCAGGAAACAAGGATCTCTACTTAGGCACCTTCAGCACCGAGGAGGAGGCGGCGGAGGCCTACGATATCGCGGCGATCAAGTTCCGTGGACTCAACGCCGTCACCAACTTCGACATGAGTCGGTACGACGTCAAGACCATCCTCGAGAGCAGCACCCTACCGGTGGGTGGCGCGGCGAGGCGCCTCAAGGAGGCCGCTGATCATCCAGAGGCTGGCGCCACGATCTGGCGGGCCGGCATGCACGGCGGCGTCATCTCCCAGCTGACCGACGTCGGGATGGGCGCGTACGCGTCGTACCCCCACGGCTGGCCGACCATCGCATTTCAGCAGCCGTCGCCGCTCTCGGTGCACTACCCATACGTCGCCCAGCCGGCCCGCGGGTGGTGCAAGCCCGAGCAGGACGCCACCGTCGCCGCGCAAAACCTGCAGGACCTCCAGCAGCTGCACCTCGGGACCGCCGCTCACAACTATTTCCAGGCATCGTCGAGCTCCACGGTGTACAACGGCGGCGGGTACCAGCAGGGCACCGGTGGCAACGCCTTCTTGATGCCGGCTACCGCCGTCATGGATGAACAGGGGCACAGCAGCACTGCCACCAACCTGGGAAGCACCTGCAGCTACGGGGATGAAGAGGGGAAGCTCAATATTGGGTACGATGCCATGGCGGCGATGGCAAGCACCGCAGCCGACCCATACGCGGCGGCGTCAACTGTGAGTATCGCGAGGGCGAACGGGTACTCCAACCACTGGAGCTCGCCGTTC

>DNA

TTGGACGCGGCGCGAAGCGCGGCTGTTGGAGATGCTCTAAGCGCTGTTCTTCTTCTCTGGAACTCTTACGATATCTTCTACACTAATCTATACCACTATAACCTGAGTGCACCAAAATAAGAGGTATATCTAATTTATCTGTTATAGCTGACCCAACACAAATAGATAGTGTGGATCTTCACTTTTCTCTGGATAAACACAGAAGTTTTTATATAGATGTCTCTTTTATAGACCTCTCAAAACGAATGTGTTTCAACGAATGATGAATACAAAAGTGATACAAAATGGAAACTGGTGCTTTCTTTTCTCTGATCAACATACTCTGGCACATACATGTACGTTAGAAATGGATGTATTTTTCTAACGACAACATGTACATTTTGATTGTGCTAACTATATATATTCTTAAATTCTTCAATATTCCTTTTCAGACATAGATGGACAGGGCGTTATGAGGCTCATCTCTGGGACAATAGCTGCAGAAGAGAAGGGCAGAGTCGCAAGGGTAGGCAAGGTAATAAAAATGGCTATATACTTCCTCTTTCTCTCTCCATTTCACAACAATATATACACCCTTGATGTTCTATTTAAGCATTATATCTTGTGTTGCATCAACACTCTCCATGACTACATCACCATCGCCTTCGGGAATGATACTGTATTTTCCTCGACAATCAGTTTATTTGGGTATGCTTGCCTTACTCTTTGGCCATTTTTATTCATTGCAGTTTACCATTTTAGTTATTCCACCTTATGATTTTGATCATGAAACCTTAGGTGGCTATGACAAGGAGGACAAGGCAGCAAGGGCTTATGATTTGGCAGCACTCAAGTATTGGGGTACAACAACAACAACAAATTTCCCAGTAAGCAAGTTCTAATATTATTTTTATGGATGCTTTTTTCCTTGCATTGCTATTTTTGTTGCTGTAAAACATCTGAAGCTATTTTTATTAGAACGTAATAGTTTTGTACAATGGCAGATTAATACCTACGAAAAAGAGGTGGATGAAATGAAACATATGACTAGGCAGGAATACATTGCATACTTAAGAAGGTACTATTGCTAGCCGACGTTCATCTCATACTGAAGTATCACACAAGTAAATGGAATATTACTCTACCAATAAATTGATCCAACAATTGCAGGAATAGCAGCGGATTTTCTCGCGGTGCGTCAAAATATCGTGGTGTAACTAGGTATGTTACTAGGTAATTCAGTCTGTTCGTTTTTTTTATAAAGGGCTTTGTTATTTAAAAGTTTAAACATTACACCCGGCCTTTTCATAACTAAGATGCACATAACCACAGTTCAAAAGTACGGTAACAGAAAAAAGCAACAGACGTAATACGATCATATGTAGACATAAATCATCGTTGTCTATGAAGAGCCAATCCGTAGATCACACTACCATCCATGTGGGATAAAAATATCCCTCGCCATTCATGTGGGATAAAATTCAGTCTGTTTGTACATATAATAATTTATGTTATGTGGAAGTAGGTAATGTCCGATATGTATTCTTAGAATTAGTAGGCATGCATGCATGCATGTCATGTAAGCAAACTTTTAGAGATTTGACTATAAACTACATACAGAATAAAATAAATGAACCTATTTCCTAAAATATGATTACATACATTCATATGTACTCCCTCCGTTCTTAAATATAAGTCTCTGTAGAGATTTCACTAGTGGACTACATACGATGTATATAGACATACTTTAGAGTATAGATTCAAACATTTTGTTTCGTATGTAGACCCTTAGTGAAATTGCTTAAAAGACTNNNNNNNNGGAACGGAGGGAGTAGTATATAGTGTAATCTTTAAAAGATCTTATATTTAGGAACGGAGGGAGTAAATAGATAGTTATCCAAATGGTTGTTATATCTTGTTTCAGTGTTCTAAGTTCTAACATCGGCTGAGATCTTTACAAGGCACCATCAGCATGGGAGATGGCAAGCAAGAATTGGGCGGGTTGCAGGAAACAAGGATCTCTACTTAGGCACCTTCAGTAAGTTATTGGCCACATATAATTAACCCTCTCTTGGGAGCCTTTCTAATTGCTAGACTAATGAAGATCCTTAATACTAAATTATGACTAGTCATAGTGTTATAGCTCCAAGATCTAAGTCATCGACCAGTACTACTTATTTAGTTATTTTTTTCTTGAACTCTTCGTCTCGTAGAACTGAAGGTTAGATATTCTATATGTTGCCCTATGGGCCAATAAAATAGTGGTCACTGCATTAACTATGTCCCAAATGCACCTTTAAGTTTTTGTCTCAAAGTTGTCTTAGAAAACCGGGGCATAGAGTCAAGGACAAAGTTGAAAACCCCAGTGGTTTGAATTAATCACGCACTTGCATCCTAACCTCCGTATAGCTGCATGCATGCATGCATGAATATGGAACATACATCACACATGCTGCATGCAAATGCAATAGGGGGGAACCAGCCCTAGCTAGCTAATAATGTACTACATCCTAATCGTGCACATCCTCTCGCACGTCCGTAGGCACCGAGGAGGAGGCGGCGGAGGCCTACGATATCGCGGCGATCAAGTTCCGTGGACTCAACGCCGTCACCAACTTCGACATGAGTCGGTACGACGTCAAGACCATCCTCGAGAGCAGCACCCTACCGGTGGGTGGCGCGGCGAGGCGCCTCAAGGAGGCCGCTGATCATCCAGAGGCTGGCGCCACGATCTGGCGGGCCGGCATGCACGGCGGCGTCATCTCCCAGCTGACCGACGTCGGGATGGGCGCGTACGCGTCGTACCCCCACGGCTGGCCGACCATCGCATTTCAGCAGCCGTCGCCGCTCTCGGTGCACTACCCATACGTCGCCCAGCCGGCCCGCGGGTGGTGCAAGCCCGAGCAGGACGCCACCGTCGCCGCGCAAAACCTGCAGGACCTCCAGCAGCTGCACCTCGGGACCGCCGCTCACAACTATTTCCAGGCATCGTCGAGCTCCACGGTGTACAACGGCGGCGGGTACCAGCAGGGCACCGGTGGCAACGCCTTCTTGATGCCGGCTACCGCCGTCATGGATGAACAGGGGCACAGCAGCACTGCCACCAACCTGGGAAGCACCTGCAGCTACGGGGATGAAGAGGGGAAGCTCAATATTGGGTACGATGCCATGGCGGCGATGGCAAGCACCGCAGCCGACCCATACGCGGCGGCGTCAACTGTGAGTATCGCGAGGGCGAACGGGTACTCCAACCACTGGAGCTCGCCGTTCAATGGCATGTGATGATGGTGAAGAGGGCAGAAGCTAGTAATAGCGGGATTACACTTAACTAGCTAGAGCATAATCCAAGTTAGGTCAGGGGTCTTGCTCGTTGTTGCGTTTCAGCTCGGGGTTCCGGCTAGCTCTTCTTGGCCGGGTGAATGACACTCGTAAAGGTGCTGGGCATGCATGCCTTCTGGAAGCTGGTAATGGCAAGTTAGGATAAGTTAAGCTTGTGTTGTTGTAAGTTATCCTAACTTGCTGTTAGTTTTGGGTAGTTTCAGAAGATCAAGTTTAATCTCTAGAGGACAAAAGCTGATCGTGCGATGGCAATAACTTCATAATTCCAG

>HvAP2-3

>Protein

MEEEERGAAAELAAIAGAGPPPKLEDFLGGGANRNGGSNNGADQAVSAATAEMYDSELKFLAAGFLSGSAGTTAPTVSPAAAPQEQADPKMPAPAPEQKKAVDSFGQRTSIYRGVTRHRWTGRYEAHLWDNSCRREGQSRKGRQVYLGGYDKEEKAARAYDLAALKYWGSSTTTNFPVADYEKEVEEMKHMTRQEFVASLRRKSSGFSRGASIYRGVTRHHQHGRWQARIGRVAGNKDLYLGTFSTEEEAAEAYDIAAIKFRGLNAVTNFEIGRYNVESIISSNLPIGNMSGGAGRGSKALESSSPEAAALPVEAPHALAFTALPMKYDQQQQDYLSFLALQHHQQGNLQGLGYGLYSSGVNLDFANAGAGGGTMAPHCYGSNGVDLHLQQHDDQQQQQHEGQEQQQQQHDHHQSMGFGTSTPMAAFSSGGPYESSVTAGSFGYYPNVAAFQTPIFGME

>cDNA

GGCATGGCCACTACCCGTGGCTCAACTTCTCCCTCGCTCACCACTGCGAGATGGAGGAGGAGGAGCGGGGAGCGGCCGCCGAGCTGGCCGCCATCGCCGGGGCCGGGCCGCCGCCCAAGCTCGAGGACTTCCTCGGGGGCGGCGCCAACCGCAACGGGGGGAGTAATAATGGCGCCGACCAGGCTGTTTCTGCAGCGACGGCGGAGATGTACGACTCGGAGCTCAAGTTCTTGGCCGCCGGATTCCTGAGCGGCTCTGCCGGAACCACGGCGCCCACCGTGTCCCCGGCGGCGGCTCCTCAGGAGCAGGCCGACCCGAAGATGCCGGCCCCGGCGCCGGAGCAGAAGAAGGCCGTCGACTCCTTCGGCCAGAGGACCTCCATCTACCGTGGCGTCACGCGGCACCGTTGGACGGGAAGGTATGAGGCGCATCTGTGGGACAACAGCTGCCGGCGTGAAGGCCAGAGCCGCAAGGGCCGGCAAGTGTACCTCGGTGGCTATGATAAGGAGGAGAAGGCGGCAAGGGCGTACGATCTTGCCGCGTTGAAGTACTGGGGGTCTAGCACCACCACCAACTTCCCGGTTGCCGACTATGAAAAGGAGGTGGAGGAGATGAAGCACATGACACGCCAGGAGTTTGTAGCTTCACTTAGGAGGAAGAGTAGTGGATTCTCCCGTGGCGCTTCCATATACAGGGGTGTCACAAGACATCACCAGCATGGTCGATGGCAGGCGAGGATCGGACGGGTTGCCGGCAACAAGGACCTATACCTCGGAACATTCAGCACCGAGGAGGAAGCCGCGGAGGCCTACGACATCGCGGCGATCAAGTTCCGGGGCCTGAACGCGGTGACCAACTTCGAGATCGGCCGGTACAACGTGGAGAGCATCATCAGCAGCAACCTCCCGATCGGCAACATGTCCGGCGGCGCAGGGCGGGGCAGCAAGGCCCTGGAGTCCAGCTCCCCGGAGGCCGCCGCATTGCCCGTGGAAGCGCCGCACGCGCTGGCGTTCACGGCGCTGCCGATGAAGTACGACCAGCAGCAGCAGGACTACCTGTCGTTCCTGGCCCTGCAGCACCACCAGCAGGGCAACCTGCAGGGGCTGGGCTACGGGCTCTACAGCTCCGGCGTCAACCTCGACTTCGCCAACGCCGGCGCAGGCGGCGGCACGATGGCGCCTCACTGCTACGGCAGCAACGGCGTCGACTTGCATCTTCAGCAGCACGATGATCAGCAGCAGCAGCAGCACGAGGGGCAGGAGCAGCAGCAGCAGCAGCATGATCACCACCAGTCCATGGGGTTCGGCACGTCGACGCCCATGGCGGCTTTCAGCAGCGGCGGGCCCTATGAAAGCTCGGTGACGGCAGGGTCCTTTGGATACTACCCAAATGTGGCAGCCTTTCAGACGCCGATCTTTGGAATGGAATGATTCGGGGCGGTGGAGGCCGTGATGTCTTTGATGAGATCAAAGACTGCTTGCTTGCTGACATAGTTCTCACAAGTCACACAACACAACAAGGAGTTAAAGAGGATGAGGAGTTAAAAGAGGATGAGGAGGAAGGGGTGGTGGGTAGGGTACGGCACGACGATATGGTGCAAACATAGGAAGCAGCATGCATGGACGAGACGCTGACAGATGAGACGCAGGGGAGAAGAAGACCCCCGCCGCTGCGCTAACAACAAGCAAAGCAGGCTAGCTAGCGTTTGTTTCTGGTTGTAATTTCTGAACTTTTCTTCTCTTTTTTCCCTTTCGTAGCTTTGGCCACTTTGCGTTTGTGATGGTCCGGGCTTTGTTGTTTGCTGCCCCTTTGAGAGCTTAGCTTTGTCTTCTTTCTGCTGACATGATTCTGCTGGAGCCATCATAGTTTTAGCCAGCCAGCAAGCAAGCCAGCTCTGTGTAATGGAAGCAGAGCAGGGACATCTTTGTAGCTTTTGCTCTTTTGCGTCTTTTCTCTAGCTAGATCGATCGCTTTAAATTAGTGCCCCACCCCCCTGCAATGTACTCCTGGGCTGGCATGGTTGCTCCAGGGTTGTTAGTCAGAGAGGATATATATAAATTCCAAGGTTCTTGTTTACAGTC

>CDS

ATGGAGGAGGAGGAGCGGGGAGCGGCCGCCGAGCTGGCCGCCATCGCCGGGGCCGGGCCGCCGCCCAAGCTCGAGGACTTCCTCGGGGGCGGCGCCAACCGCAACGGGGGGAGTAATAATGGCGCCGACCAGGCTGTTTCTGCAGCGACGGCGGAGATGTACGACTCGGAGCTCAAGTTCTTGGCCGCCGGATTCCTGAGCGGCTCTGCCGGAACCACGGCGCCCACCGTGTCCCCGGCGGCGGCTCCTCAGGAGCAGGCCGACCCGAAGATGCCGGCCCCGGCGCCGGAGCAGAAGAAGGCCGTCGACTCCTTCGGCCAGAGGACCTCCATCTACCGTGGCGTCACGCGGCACCGTTGGACGGGAAGGTATGAGGCGCATCTGTGGGACAACAGCTGCCGGCGTGAAGGCCAGAGCCGCAAGGGCCGGCAAGTGTACCTCGGTGGCTATGATAAGGAGGAGAAGGCGGCAAGGGCGTACGATCTTGCCGCGTTGAAGTACTGGGGGTCTAGCACCACCACCAACTTCCCGGTTGCCGACTATGAAAAGGAGGTGGAGGAGATGAAGCACATGACACGCCAGGAGTTTGTAGCTTCACTTAGGAGGAAGAGTAGTGGATTCTCCCGTGGCGCTTCCATATACAGGGGTGTCACAAGACATCACCAGCATGGTCGATGGCAGGCGAGGATCGGACGGGTTGCCGGCAACAAGGACCTATACCTCGGAACATTCAGCACCGAGGAGGAAGCCGCGGAGGCCTACGACATCGCGGCGATCAAGTTCCGGGGCCTGAACGCGGTGACCAACTTCGAGATCGGCCGGTACAACGTGGAGAGCATCATCAGCAGCAACCTCCCGATCGGCAACATGTCCGGCGGCGCAGGGCGGGGCAGCAAGGCCCTGGAGTCCAGCTCCCCGGAGGCCGCCGCATTGCCCGTGGAAGCGCCGCACGCGCTGGCGTTCACGGCGCTGCCGATGAAGTACGACCAGCAGCAGCAGGACTACCTGTCGTTCCTGGCCCTGCAGCACCACCAGCAGGGCAACCTGCAGGGGCTGGGCTACGGGCTCTACAGCTCCGGCGTCAACCTCGACTTCGCCAACGCCGGCGCAGGCGGCGGCACGATGGCGCCTCACTGCTACGGCAGCAACGGCGTCGACTTGCATCTTCAGCAGCACGATGATCAGCAGCAGCAGCAGCACGAGGGGCAGGAGCAGCAGCAGCAGCAGCATGATCACCACCAGTCCATGGGGTTCGGCACGTCGACGCCCATGGCGGCTTTCAGCAGCGGCGGGCCCTATGAAAGCTCGGTGACGGCAGGGTCCTTTGGATACTACCCAAATGTGGCAGCCTTTCAGACGCCGATCTTTGGAATGGAATGA

>DNA

CTTGCTGGTCGGAGTAGGATTACTCATCATCCTTCTGTTCTGACCATGGACATGGACGCCGCGCATGGCCACTACCCGTGGCTCAACTTCTCCCTCGCTCACCACTGTCAGTACTACTACCATCCCACGATCCCTTCCTTCTCTTGCTCAGTCACCTTCTTGGTCGTCGCGCTGATGGGTATGTATGTGTGTGTATGCATGTGCAGGCGAGATGGAGGAGGAGGAGCGGGGAGCGGCCGCCGAGCTGGCCGCCATCGCCGGGGCCGGGCCGCCGCCCAAGCTCGAGGACTTCCTCGGGGGCGGCGCCAACCGCAACGGGGGGAGTAATAATGGCGCCGACCAGGCTGTTTCTGCAGCGACGGCGGAGATGTACGACTCGGAGCTCAAGTTCTTGGCCGCCGGATTCCTGAGCGGCTCTGCCGGAACCACGGCGCCCACCGTGTCCCCGGCGGCGGCTCCTCAGGAGCAGGCCGACCCGAAGATGCCGGCCCCGGCGCCGGAGCAGAAGAAGGCCGTCGACTCCTTCGGCCAGAGGACCTCCATCTACCGTGGCGTCACGCGGTAAGCCAGCTCTCTATGCATGGCGTTGATCCTTCTAGCTGTTTGGCCAAGAAACCTACCAAGAGGTTGTCCTGTCTGGGTTCTTGCCTTCTTGAGCTCTCTACTGCTTGCATGCAAATATCTGGCCGATATTCGTCTGGATGCAAATAAATACATAAATACATATCACACAAATTAACCACTAACTACATAGAAAAGCAGAAATGATACTAGAACTAATCTCAAGTTGTTCATTCGGCAAAATAAGGAGCTCTTTTCCTATGCTCGATCAAGTTTTAGCCCTCTCGTCTGATTTTACTGCCCGTTTTGGGTGATTTTTAGGCACCGTTGGACGGGAAGGTATGAGGCGCATCTGTGGGACAACAGCTGCCGTCGTGAAGGCCAGAGCCGCAAGGGCCGGCAAGGTGACCACACGATACTACTCACCATGCATACACGTCCGCGAATGATCAAATCCATTCATCATTTTGCTGTTTCTAACTTGTGCTTCTTGATGTTCTTACCATGTCAACTTGATGTGTTATTTCTCCTTCATGGCTGTTATTCCTGGCGTCATCTCCCTCCAGTGTACCTCGGTGAGAAAACGAGAGCCTTCTAGCTCTTCCATTAAAAAAATGTATCTGTGTGGGTGAAGCTAAAGTGTTGTCGATGCATGGAAACAACAGGTGGCTATGATAAGGAGGAGAAGGCGGCAAGGGCGTACGATCTTGCCGCGTTGAAGTACTGGGGGTCTAGCACCACCACCAACTTCCCGGTAAGATGATATATAGACGGATTGCATGATTGCCAAAACTCCTGGATATGTGCAAGAATGTGCAGCAAAGCATGGGCATATCTACCGATGTGCTGGGTGTGTATCTTTATCCGGCAAGTGTTCTTTTTTAGGTTGCCGACTATGAAAAGGAGGTGGAGGAGATGAAGCACATGACACGCCAGGAGTTTGTAGCTTCACTTAGGAGGTACTTGCATACATATGGGCTACTAATCATCACTCCTTCTAGTCTTCTAATGTTGCACTAGCATTGTGTGCATGCTTACATGCATGCATGGACATTCTATATATACTATGCATGTTTGATCACTTATACACTAGCATTTTTTTTATGTTATTCAAGTGATTTTGATGACGAAAAAAACATGTAATCTGGTGCAGGAAGAGTAGTGGATTCTCCCGTGGCGCTTCCATATACAGGGGTGTCACAAGGTTCATATTCTCCTCTCCATTTGTTTGCTTGTGTGTTAACCATGTTTGCCATCTTGCATGTGGTGTCATCTAAAAATGGTATGTTTTGCATTCTATCATCTAGACATCACCAGCATGGTCGATGGCAGGCGAGGATCGGACGGGTTGCCGGCAACAAGGACCTATACCTCGGAACATTCAGTGAGTATATACATCCTTGTGCAAACTATGTTTAATTTGTGTACTCGGATGAAGATATGCATGCATGTTCGGCTACTTTCTCTCACTGCACCCATTGCATACTAAACCAAGACATTTTTATTAAACCAAGATATATCTTTGCTACCTTACATTACCTCTTTTGTTCCCAAAAAGAGATAAGAGATATATGTGGGTAAATTAACTAAGAATTAAGTGAGGGTACAATCTTCCCAAAGCTTAGTTGGGTAATGAAATCTCAAGTAGGAGGGGAGCACGTGTTCGTGTTAGCCCTCCCCTCGAAGCGGGCATTAGTCGCATCTTTCCATCAACAACCACATTACAATACTGTTGCCGTGCACGTAGGTTCATCTTAATTTACAGTCTGTTCGTACAGGTCTTGTAGCTAGATTCGTCGACATATTTTTTATCTAGGTCAAAAACCATCTAGTTTGACCGGATGCACCCATGGACCACGTGTATCTACAGGCGGCATGCCCACACTTGATTCCAGTTTGTCATCTTTTATGATGGTTTTGGGGCTTTGGAAAAAGGAGTGTGTGTAATGCTCAGATGGGAGCCTGAACACTTTCCTTCCTGCATTGCATGTAGCAACCTGTATTTAGAACTTGGAATATAGTTGCTGTTTTCACGGCCAGCTTTCCACAAAGCGTCTGACCGGTGATTATATTAGGAGAGTATCTTACTGAGATGGCCATTATTGCACTCGGTTTAACTAAGCTCATTGCACTCATGGGCTTGTCAGTACTAGCAGGTAATTAGTCAAAAGTCCTAGTGATGCAGCTTGCATTGCTGTCTAGGAGCTGGAAATCCATGGCATTCTTCTGCATGCTTTTGTCTATGTCCTGGTACCATATGTTTCATCTCTGTTTTTCTTTTCAAGCATTTTTTTCCTGCGTTGTCTTTCTTTTTTGTAGTAGTGAAAAATGTATATATGTTGATTTTAATGCAAAATACTTCCTCATGAAGAATATTGCTTGATCTCCAACAACTGACATTTTGATAAAATCTAACAACTGAAATGCTATCTAAAATAGGCACCGAGGAGGAAGCCGCGGAGGCCTACGACATCGCGGCGATCAAGTTCCGGGGCCTGAACGCGGTGACCAACTTCGAGATCGGCCGGTACAACGTGGAGAGCATCATCAGCAGCAACCTCCCGATCGGCAACATGTCCGGCGGCGCAGGGCGGGGCAGCAAGGCCCTGGAGTCCAGCTCCCCGGAGGCCGCCGCATTGCCCGTGGAAGCGCCGCACGCGCTGGCGTTCACGGCGCTGCCGATGAAGTACGACCAGCAGCAGCAGGACTACCTGTCGTTCCTGGCCCTGCAGCACCACCAGCAGGGCAACCTGCAGGGGCTGGGCTACGGGCTCTACAGCTCCGGCGTCAACCTCGACTTCGCCAACGCCGGCGCAGGCGGCGGCGCGATGGCGCCTCACTGCTACGGCAGCAACGGCGTCGACTTGCATCTTCAGCAGCACGATGATCAGCAGCAGCAGCAGCACGAGGGGCAGGAGCAGCAGCAGCAGCAGCATGATCACCACCAGTCCATGGGGTTCGGCACGTCGACGCCCATGGCGGCTTTCAGCAGCGGCGGGCCCTATGAAAGCTCGGTGACGGCAGGGTCCTTTGGATACTACCCAAATGTGGCAGCCTTTCAGACGCCGATCTTTGGAATGGAATGATTCGGGGCGGTGGAGGCCCTGATGTCTTTGATGAGATCAAAGACTGCTTGCTTGCTGACATAGTTCTCACAATTCACACAACACAACAAGGAATTAAAGAGGATGAGGAGTTAAAAGAGGATGAGGAGGAAGGGGTGGTGGGTAGGGTACGGCACGACGATATGGTGCAAACATAGGAAGCAGCATGCATGGACGAGACGCTGACAGATGAGACGCAGGGGAGAAGAAGACCCCCGCCGCTGCGCTAACAACAAGCAAAGCAGGCTAGCTAGCGTTTGTTTCTGGTTGTAATTTCTGAACTTTTCTTCTCTTTTTTCCCTTTCGTAGCTTTGGCCACTTTGCGTTTGTGATGGTCCGGGCTTTGTTGTTTGCTGCCCCTTTGAGAGCTTAGCTTTGTCTTCTTTCTGCTGACATGATTCTGCTGGAGCCATCATAGTTTTAGCCAGCCAGCAAGCAAGCCAGCTCTGTGTAATGGAAGCAGAGCAGGGACATCTTTGTAGCTTTTGCTCTTTTGCGTCTTTTCTCTAGCTAGATCGATCGCTTTAAATTAGTGCCCCACCCCCCTGCAATGTACTCCTGGGCTGGCATGGTTGCTCCAGGGTTGTTAGTCAGAGAGGATATATATAAATTCCAAGGTTCTTGTTTACAGTC

>HvAP2-4

>Protein

MAALKYWGPTTTTNFPVSNYEKELEEMKSMTRQEFIASLRRKSSGFSRGASIYRGVTRHHQHGRWQARIGRVAGNKDLYLGTFSTQEEAAEAYDIAAIKFRGLSAVTNFDMSRYDVESILNSDLPIGAGAAARASKFQPDVLSLPAPNVASPDMLPPAEKDYWSLLAMHYQQQQQQHLQQYPASAFETYGSGVNVDFTMGMGSANNASGGATVWGTTGATGHGDVGSRQSNSYSSNIPYASMVSGSGATATGGYEGSTGNNGTWVTTSNPASTTAPQYYNYLFGME

>cDNA

AGGAGGAGAAGGCGGCGAGGGCCTACGACATGGCTGCGCTGAAGTACTGGGGTCCGACCACCACGACGAACTTCCCGGTGTCCAACTACGAGAAGGAGTTGGAGGAGATGAAGTCGATGACGCGGCAGGAGTTCATCGCTTCCCTTCGCAGGAAGAGCAGCGGCTTCTCACGAGGGGCGTCCATCTACAGAGGCGTAACAAGGCATCATCAGCACGGCCGGTGGCAGGCAAGGATCGGCAGGGTGGCCGGAAACAAGGACCTGTACCTGGGAACTTTCAGCACGCAGGAGGAGGCGGCGGAGGCGTACGATATCGCGGCGATCAAGTTCCGTGGGCTCAGCGCCGTGACCAACTTCGACATGAGCCGCTACGACGTCGAGAGCATCCTCAACAGCGACCTGCCCATCGGTGCCGGGGCGGCCGCCCGCGCCTCCAAGTTCCAACCGGACGTCCTATCGCTGCCGGCGCCAAATGTGGCATCACCGGACATGCTGCCGCCGGCGGAGAAGGACTACTGGTCCCTGCTCGCCATGCACTACCAGCAGCAGCAGCAACAGCACCTGCAGCAGTACCCCGCCTCGGCATTCGAGACCTACGGGTCCGGCGTGAACGTGGACTTCACAATGGGCATGGGCAGCGCCAACAACGCCAGCGGCGGCGCCACCGTGTGGGGCACCACCGGTGCAACAGGACACGGCGACGTCGGCAGCAGGCAGAGCAACAGCTACTCCAGCAACATTCCTTATGCTTCCATGGTGTCTGGATCAGGGGCAACAGCAACCGGGGGTTACGAGGGCTCCACCGGCAACAATGGCACCTGGGTGACGACGAGCAACCCGGCCAGCACGACGGCTCCTCAGTACTACAACTATCTGTTTGGCATGGAGTAGTAGGTGATCGATCACTGACTGCCTAGCTAGCCATAGCTATAGCCATAGCTGAAATTGGGTGATAGGCCTAGGAGGAGGATGGAGAGGGACTAACATGGAACTGATCAAAATTTTGGCTGCTGCTGCGTGCATGGGAACGATGTATGATGATGAATGCGATGCATTCTTCTCCTGTTCTTTTTTACTTCCTGACAAAGGTCCTGTAGCGAGAGAACGCTCGTGTATCATCTGAATTTCTAATTCTGAATTTCTAATCCAG

>CDS

ATGGCTGCGCTGAAGTACTGGGGTCCGACCACCACGACGAACTTCCCGGTGTCCAACTACGAGAAGGAGTTGGAGGAGATGAAGTCGATGACGCGGCAGGAGTTCATCGCTTCCCTTCGCAGGAAGAGCAGCGGCTTCTCACGAGGGGCGTCCATCTACAGAGGCGTAACAAGGCATCATCAGCACGGCCGGTGGCAGGCAAGGATCGGCAGGGTGGCCGGAAACAAGGACCTGTACCTGGGAACTTTCAGCACGCAGGAGGAGGCGGCGGAGGCGTACGATATCGCGGCGATCAAGTTCCGTGGGCTCAGCGCCGTGACCAACTTCGACATGAGCCGCTACGACGTCGAGAGCATCCTCAACAGCGACCTGCCCATCGGTGCCGGGGCGGCCGCCCGCGCCTCCAAGTTCCAACCGGACGTCCTATCGCTGCCGGCGCCAAATGTGGCATCACCGGACATGCTGCCGCCGGCGGAGAAGGACTACTGGTCCCTGCTCGCCATGCACTACCAGCAGCAGCAGCAACAGCACCTGCAGCAGTACCCCGCCTCGGCATTCGAGACCTACGGGTCCGGCGTGAACGTGGACTTCACAATGGGCATGGGCAGCGCCAACAACGCCAGCGGCGGCGCCACCGTGTGGGGCACCACCGGTGCAACAGGACACGGCGACGTCGGCAGCAGGCAGAGCAACAGCTACTCCAGCAACATTCCTTATGCTTCCATGGTGTCTGGATCAGGGGCAACAGCAACCGGGGGTTACGAGGGCTCCACCGGCAACAATGGCACCTGGGTGACGACGAGCAACCCGGCCAGCACGACGGCTCCTCAGTACTACAACTATCTGTTTGGCATGGAGTAG

>DNA

ATACGAAGTTATTACGAGACATAATTTAGAGTGTAGATTCACTCATTTTGTTCCGTATGTAGACTCCTAGTGAAATCGCTTAAAAGACTTATATTTAGGAACGGAGGGAGTATCAAGCACCCTATAAGGACAGCCTGAAAAGCTCATCAGGACGGGTGAACAAAAGCAACGGCACCAGTCCACCCCACACCCTCGCTAGCTATGCTCCACGGGTTTGGCGTAGAGAGCAGCAAACAAAAAGGAAAGGGAAAAAGAAGAAAGTTGCATGCATGGCTAGCGCGCGCGGCAATGGCGGGGGGGCTGCATGCGGGGGAACAGCGGCCGCCATTCCGGTCAAGGAGACCGCCCATGCCACCCCGTCGAGCGCCTCCACGTTGTCGACCACCACCACCATTAGTCCATTACCATTACCACCACGCCCCTTTGGATTCTCGCAAAGCAAGAGGCTCTACTACTAGTACCATGCATTAAACTACTCCACAAATCACTCTCCCTAATTAATCCCTCGCTCTCTCCTTGGCCCGTGTGCACGCCCTCTCCTTGGACGCATTCACATTCTCTCTCTCTAGAGAGTGTGTGTGTGCGTGTGTGAACAGTGCACAGTTCAAACAAGCAAGCTCAAGCGACCGGCCGGAGTTCATTTGCACTGGTTGTGGTTAGTTACTCTATATAAACTACCATGCTTTGACTATTCTCTGCAGAGAGTCCCATTCCCCTCCTTGCACCCGTACCCCTTCTTCTAGCTAGCTATAGTCTTCTTCTTCTCAAGGCAAGGCACACGGCACACGGCCGCCATGGACATGGACATGAGCTCTGCTGCCCACGCTGCTCACCACTGGCTCTCCTTCTCCCTCTCCAACAACTACCACCACGGCCTCCTCGAGGCCCTCTCCAGCTCCTCCTCCGGCCACCACATCGCCGGTAAGGAGGCCAAGGCCAACACCCCTAATCCCTATATCGATCATCATCTGCTAGTGAGTGATCTCTATCTACACATGCATCCTAGCTCGTGACTAACGGTGCATGCTTTCTTGGGCTTTTGTAGGAGAGGAAGGCCCCGTGGACGAGGCGCCTAAGATGGAGGACTTCCTCGGCTGTGCCGGCGGCAGCGCCGGCACGACATCGACGGCTGTGGTAGACCAAGGCGAGCTGGGTAGCATCGCCGCGGGGTTCTTGCATCGGTATCCGGCGCACGACGGTACGCCGGACCAGAACTCCGGCGCGGTGACCGTAGCGGCGACGACCATGGAGGTCGCCGAGTCCGATCAGGCGAGGAGGCCCGCCGAGACGTTTGGCCAGCGGACGTCAATCTACCGCGGCGTCACCAGGTAGGGATTTATACTTGCTAACTAATCACATGCATGCATCTTCCTCTTCCTATCGAGATGACAGAAGAACATATACTAGTTGGTTAAGATGGTTCTTAGTAGTAGCACTTCTTGCTTGGCGAGTGAAGTACACCAGTTGTATACATGTTTTGTTCTATCAACAAGTTCATCTTTGCTAATCCATCTCGTGGTGTTTTGTCGAAGTAATGCATGATCTGGAGTTCTGGACTATGCAGGCACCGGTGGACGGGGAGGTATGAGGCGCACCTGTGGGACAACAGCTGCCGCCGGGAAGGCCAAAGCCGCAAAGGCCGCCAAGGTACACCGTCATGCATGCATGCTTTGCCCAATAATCCTGCAAAAAACAACACATACATAAATTCTGATCACTAACATGTTTTCTCTTTGCTTTTCCCCCACAAATTTCTCTGACTTGTTGGCGCATTGGCGATAATGATCATCAGTCTATTTAGGTATATATGTCACAAAGCCACATCCACTCATTTTACAGTTTTACACATCTTCATCAATGCCATCGCTTCATGATTTGGAGAAGAAGTATTTAAGGCTTACATTAGGAATTTTGGGGGAAATGCAGGAGGCTATGACAAGGAGGAGAAGGCGGCGAGGGCCTACGACATGGCTGCGCTGAAGTACTGGGGTCCGACCACCACGACGAACTTCCCGGTGAGTACCGACTCCGGCAAGAGATCGACATTGATCGACAACTCCATATACATACATGATCTCTGAGCGTGTTCGCCATATCCATATATATAGGTGTCCAACTACGAGAAGGAGTTGGAGGAGATGAAGTCGATGACGCGGCAGGAGTTCATCGCTTCCCTTCGCAGGTTAGCACTTGGCCGGCGACCACCCAGACTGCCCAGATTCACAGGGGCTCGCTGACTTACGCGTCCTGTTCATCTTCTCCACGTACACACCTTGCCGTACGCGTGCACGCACGCACGCATGCACAGCTTCTGCATGTGATCTGCTTTACTCCGGCCGAGTTCTCAGTTCATCACACATGCATTGCATGTTGACCTCAATGCGTGTTCCTCACACAGGAAGAGCAGCGGCTTCTCACGAGGGGCGTCCATCTACAGAGGCGTAACAAGGTAATTAACAATGGTTTCACCTGAATGCATGAGCTTGCCAAAACAAAATGTGGTACCAGTATGAACTTTGAATATGAAAAAAAAGAACACGCAAATGTCTTCTTCTTTCGGTCAAATGTTTGTAACAACCGAGATTTGATTTTATATATTTGATGTCAGGCATCATCAGCACGGCCGGTGGCAGGCAAGGATCGGCAGGGTGGCCGGAAACAAGGACCTGTACCTGGGAACTTTCAGTGAGTAGTCTTCTTGATCCCCCACATTGGCATTTGCCTTCTGATGATGTGCCACCACTGCTATAGGGTCCATTGCATGCATTGCTAAATTATTAATCGAATTTGATGGTACGGCATCTGATGATTAATTTGAGGTGTACGGCATCTGATGATTAATCCGGTGGTCTGACCGTCTCTCCGCACCACAGGCACGCAGGAGGAGGCGGCGGAGGCGTACGATATCGCGGCGATCAAGTTCCGTGGGCTCAGCGCCGTGACCAACTTCGACATGAGCCGCTACGACGTCGAGAGCATCCTCAACAGCGACCTGCCCATCGGTGCCGGGGCGGCCGCCCGCGCCTCCAAGTTCCAACCGGACGTCCTATCGCTGCCGGCGCCAAATGTGGCATCACCGGACATGCTGCCGCCGGCGGAGAAGGACTACTGGTCCCTGCTCGCCATGCACTACCAGCAGCAGCAGCAACAGCACCTGCAGCAGTACCCCGCCTCGGCATTCGAGACCTACGGGTCCGGCGTGAACGTGGACTTCACAATGGGCATGGGCAGCGCCAACAACGCCAGCGGCGGCGCCACCGTGTGGGGCACCACCGGTGCAACAGGACACGGCGACGTCGGCAGCAGGCAGAGCAACAGCTACTCCAGCAACATTCCTTATGCTTCCATGGTGTCTGGATCAGGGGCAACAGCAACCGGGGGTTACGAGGGCTCCACCGGCAACAATGGCACCTGGGTGACGACGAGCAACCCGGCCAGCACGACGGCTCCTCAGTACTACAACTATCTGTTTGGCATGGAGTAGTAGGTGATCGATCACTGACTGCCTAGCTAGCCATAGCTATAGCCATAGCTGAAATTGGGTGATAGGCCTAGGAGGAGGATGGAGAGGGACTAACATGGAACTGATCAAAATTTTGGCTGCTGCTGCGTGCATGGGAACGATGTATGATGATGAATGCGATGCATTCTTCTCCTGTTCTTTTTTACTTCCTGACAAAGGTCCTGTAGCGAGAGAACGCTCGTGTATCATCTGAATTTCTAATTCTGAATTTCTAATCCAG

>HvAP2-5

>Protein

MRAMAGGGTNCLGFSLSPHIDMAMEVPSSSGPDHAQPASASAMSTNAATCNFLFSPPAQMAAPPPGYYYIGGAYGDGTSTAGVYYSHHPVMPVTSDGSLCIMEGMMPSSSPKLEDFLGGGNDGGHDTVTYYSHHHQQQGQQDQEGSRVYQHHQHYQQQQQELAPYNFQHLTEAEAIYQEATAPMDDAMAAAKNLLVTSYGSCYSNAGMQPLSLSMSPSSQSSSCVSAAPQQHQMAAVASAAASMAASQGGSNGGGEQCVGKKRGTGKGGQKQPVHRKSIDTFGQRTSQYRGVTSRHRWTGRYEAHLWDNSCKKDGQTRKGRQVYLGGYDNEDKAARAYDLAALKYWGPSTNTNFPLETYREEVEEMKSMTRQEFIAHLRRRSSGFSRGASIYRGVTRHHQHGKWQARIGRVAGNKDLYLGTFTTQEEAAEAYDVAAIKFRGLNAVTNFDITRYDVDKIMESSSLLPGEEARKVKPIEAANHVPANAMHNGGAELSHAEEGSSGVWKMVLHGTPQQA

>cDNA

CTTTGGCTTGCTTGGCCCCCTCCCTCTCATTCCTTTTGCTCAGTTCACGGGTCCCTCCCGTCTTCCTCGTAGTTCACTTCTCTTTTACTTCTACTACTAGCTCCACCTCCATGTCGTCGCTCGGACAAGGATAGTGCAGTGACGCAGTAGTAGTGGGGCTCAGCTCAGAGTGAAAGCGAAGCAAGAAGCGTTTTCGTCTGTGTTTGTTTGTTGATGAGAGCAATGGCCGGCGGCGGCACCAACTGCTTAGGCTTCTCCCTCTCCCCACACATTGACATGGCCATGGAGGTGCCCTCCTCCTCTGGACCCGACCACGCTCAGCCTGCTAGCGCTAGTGCTATGTCCACCAACGCCGCGACCTGCAACTTCCTCTTCTCCCCTCCCGCGCAAATGGCCGCTCCACCTCCTGGCTACTACTACATCGGCGGCGCCTATGGGGATGGCACCAGCACCGCCGGCGTCTACTACTCCCACCACCCCGTCATGCCCGTCACGTCCGATGGATCCCTGTGCATCATGGAAGGCATGATGCCGTCGTCCTCGCCGAAGCTCGAGGACTTCTTGGGCGGCGGCAATGACGGTGGACACGACACAGTCACCTACTACAGCCACCACCACCAGCAGCAGGGCCAGCAAGACCAGGAGGGAAGCAGAGTCTACCAGCACCATCAGCACTATCAGCAGCAGCAGCAGGAGCTAGCGCCCTACAACTTCCAGCACTTGACGGAAGCAGAGGCGATCTACCAAGAGGCCACGGCGCCGATGGACGATGCAATGGCCGCTGCCAAGAACCTGCTGGTGACAAGCTACGGGTCATGCTACAGCAACGCGGGGATGCAGCCGCTGAGCCTGTCCATGAGCCCCAGTTCCCAGTCCAGCAGCTGCGTCAGCGCAGCTCCTCAGCAGCATCAGATGGCTGCGGTTGCTTCTGCTGCTGCCTCTATGGCAGCTTCCCAGGGAGGCAGTAATGGTGGCGGGGAGCAGTGTGTGGGGAAGAAGAGGGGCACTGGGAAGGGAGGCCAGAAGCAGCCCGTTCACCGCAAGTCCATTGACACGTTTGGGCAGAGGACCTCCCAGTATAGGGGCGTCACCAGCAGGCACAGGTGGACTGGGAGATATGAAGCCCACCTCTGGGACAACAGTTGCAAGAAGGATGGGCAGACGAGGAAAGGGAGGCAAGTTTATCTAGGTGGTTATGACAATGAAGACAAGGCTGCCAGGGCTTATGATCTGGCTGCTCTGAAATATTGGGGGCCGTCGACGAACACCAATTTCCCGCTAGAAACTTATCGAGAGGAGGTCGAGGAGATGAAAAGCATGACAAGGCAGGAATTCATTGCACACTTGAGAAGGAGAAGCAGCGGGTTTTCTCGTGGTGCTTCGATATATCGAGGAGTGACGAGGCATCATCAGCATGGAAAATGGCAAGCTAGGATTGGCAGGGTTGCTGGGAACAAAGACTTGTATCTCGGCACTTTCACCACTCAGGAAGAAGCAGCTGAGGCCTATGACGTAGCCGCGATCAAGTTCCGTGGCCTGAACGCCGTAACCAACTTCGACATAACGAGGTACGACGTGGACAAGATCATGGAGAGCAGCTCTCTGCTGCCCGGGGAGGAAGCGCGCAAGGTCAAGCCGATCGAGGCGGCCAACCACGTGCCTGCCAATGCCATGCACAACGGCGGCGCGGAGCTCAGCCATGCCGAAGAAGGAAGCTCCGGCGTCTGGAAGATGGTGCTCCACGGGACACCACAACAAGCAGC

>CDS

ATGAGAGCAATGGCCGGCGGCGGCACCAACTGCTTAGGCTTCTCCCTCTCCCCACACATTGACATGGCCATGGAGGTGCCCTCCTCCTCTGGACCCGACCACGCTCAGCCTGCTAGCGCTAGTGCTATGTCCACCAACGCCGCGACCTGCAACTTCCTCTTCTCCCCTCCCGCGCAAATGGCCGCTCCACCTCCTGGCTACTACTACATCGGCGGCGCCTATGGGGATGGCACCAGCACCGCCGGCGTCTACTACTCCCACCACCCCGTCATGCCCGTCACGTCCGATGGATCCCTGTGCATCATGGAAGGCATGATGCCGTCGTCCTCGCCGAAGCTCGAGGACTTCTTGGGCGGCGGCAATGACGGTGGACACGACACAGTCACCTACTACAGCCACCACCACCAGCAGCAGGGCCAGCAAGACCAGGAGGGAAGCAGAGTCTACCAGCACCATCAGCACTATCAGCAGCAGCAGCAGGAGCTAGCGCCCTACAACTTCCAGCACTTGACGGAAGCAGAGGCGATCTACCAAGAGGCCACGGCGCCGATGGACGATGCAATGGCCGCTGCCAAGAACCTGCTGGTGACAAGCTACGGGTCATGCTACAGCAACGCGGGGATGCAGCCGCTGAGCCTGTCCATGAGCCCCAGTTCCCAGTCCAGCAGCTGCGTCAGCGCAGCTCCTCAGCAGCATCAGATGGCTGCGGTTGCTTCTGCTGCTGCCTCTATGGCAGCTTCCCAGGGAGGCAGTAATGGTGGCGGGGAGCAGTGTGTGGGGAAGAAGAGGGGCACTGGGAAGGGAGGCCAGAAGCAGCCCGTTCACCGCAAGTCCATTGACACGTTTGGGCAGAGGACCTCCCAGTATAGGGGCGTCACCAGCAGGCACAGGTGGACTGGGAGATATGAAGCCCACCTCTGGGACAACAGTTGCAAGAAGGATGGGCAGACGAGGAAAGGGAGGCAAGTTTATCTAGGTGGTTATGACAATGAAGACAAGGCTGCCAGGGCTTATGATCTGGCTGCTCTGAAATATTGGGGGCCGTCGACGAACACCAATTTCCCGCTAGAAACTTATCGAGAGGAGGTCGAGGAGATGAAAAGCATGACAAGGCAGGAATTCATTGCACACTTGAGAAGGAGAAGCAGCGGGTTTTCTCGTGGTGCTTCGATATATCGAGGAGTGACGAGGCATCATCAGCATGGAAAATGGCAAGCTAGGATTGGCAGGGTTGCTGGGAACAAAGACTTGTATCTCGGCACTTTCACCACTCAGGAAGAAGCAGCTGAGGCCTATGACGTAGCCGCGATCAAGTTCCGTGGCCTGAACGCCGTAACCAACTTCGACATAACGAGGTACGACGTGGACAAGATCATGGAGAGCAGCTCTCTGCTGCCCGGGGAGGAAGCGCGCAAGGTCAAGCCGATCGAGGCGGCCAACCACGTGCCTGCCAATGCCATGCACAACGGCGGCGCGGAGCTCAGCCATGCCGAAGAAGGAAGCTCCGGCGTCTGGAAGATGGTGCTCCACGGGACACCACAACAAGCA

>DNA

ATCGCGTAGGCACGCCTGTACAGCTGCATACGCACGTTCAAAAACCAACAGAGGCCAAGAACAAACAGGCCCCTAGATTTTATATGCCCGCTCTAATGGAATTGCATTAGGGCTCAGGAGAGGGTGGTACTTATCCACCGCTTTGCTTTGGCTTGCTTGGCCCCCTCCCTCTCATTCCTTTTGCTCAGTTCACGGGTCCCTCCCGTCTTCCTCGTAGTTCACTTCTCTTTTACTTCTACTACTAGCTCCACCTCCATGTCGTCGCTCGGACAAGGATAGTGCAGTGACGCAGTAGTAGTGGGGCTCAGCTCAGAGTGAAAGCGAAGCAAGAAGCGTTTTCGTCTGTGTTTGTTTGTTGATGAGAGCAATGGCCGGCGGCGGCACCAACTGCTTAGGCTTCTCCCTCTCCCCACACATTGACATGGCCATGGAGGTGCCCTCCTCCTCTGGACCCGACCACGCTCAGCCTGCTAGCGCTAGTGCTATGTCCACCAACGCCGCGACCTGCAACTTCCTCTTCTCCCCTCCCGCGCAAATGGCCGCTCCACCTCCTGGCTACTACTACATCGGCGGCGCCTATGGGGATGGCACCAGCACCGCCGGCGTCTACTACTCCCACCACCCCGTCATGCCCGTCACGTCCGATGGATCCCTGTGCATCATGGAAGGTGTGCTCACTGTTGCACTTCTCTCTCTCTCTCTCTTGGTTCCCGTTGCTGCAAACGCTGGCATTAATCTTTCGTGCTGCCATCACGTAGGCATGATGCCGTCGTCCTCGCCGAAGCTCGAGGACTTCTTGGGCGGCGGCAATGACGGTGGACACGACACAGTCACCTACTACAGCCACCACCACCAGCAGCAGGGCCAGCAAGACCAGGAGGGAAGCAGAGTCTACCAGCACCATCAGCACTATCAGCAGCAGCAGCAGGAGCTAGCGCCCTACAACTTCCAGCACTTGACGGAAGCAGAGGCGATCTACCAAGAGGCCACGGCGCCGATGGACGATGCAATGGCCGCTGCCAAGAACCTGCTGGTGACAAGCTACGGGTCATGCTACAGCAACGCGGGGATGCAGCCGCTGAGCCTGTCCATGAGCCCCAGTTCCCAGTCCAGCAGCTGCGTCAGCGCAGCTCCTCAGCAGCATCAGATGGCTGCGGTTGCTTCTGCTGCTGCCTCTATGGCAGCTTCCCAGGGAGGCAGTAATGGTGGCGGGGAGCAGTGTGTGGGGAAGAAGAGGGGCACTGGGAAGGGAGGCCAGAAGCAGCCCGTTCACCGCAAGTCCATTGACACGTTTGGGCAGAGGACCTCCCAGTATAGGGGCGTCACCAGGTAAGTTGCTTATACTACAGATACTTAGCTTTTGGATTCACCTATGATCTCAACTTGCTCAATAGTAATACGTACCATCTTTTGTAAGTTCTATTTGATCATTTTACATTGCTGATCTTGTGCAAATAAACTAATGTTTCTAAAATAAATAAATTCTGCTTCATTTAGAACATGTAATGGCAGTACTAGCTACTCCAGTATACTCCCCCCTGTTTGAGCCTTATGTTTTGCCCTGTGATTTTGTCCTAGGTAGCAGCATTGATGTCTGCAGTCTTCCTCTGATGCACATCTGCAGTTCTTGTTATTTAGTAGGGCACCCACCCATATTTCGTTCCAAAACAAATTAATGCCATTGACATGGCCCTCGTTAAGATTTATTCACCCACAACATGCTTTTGTTTCGTTCCATTCCTCAAGATTCAGCCCAATGCACCTGTTGCTTCTGATGATGCATCTTCGGTTCTTTGAATTGATGAGACACCACCCACCTACTCTTTTGTTTTTGACCAAACCACCTGAGTCATCTCGCCTCCATTTTTATATTGTTTATATCGAATGACCAAGGTTTGTGTGTTTGATCCACTGGAGCAGGCACAGGTGGACTGGGAGATATGAAGCCCACCTCTGGGACAACAGTTGCAAGAAGGATGGGCAGACGAGGAAAGGGAGGCAAGGTTGATTACTACTAGCTCAACTTCTAGCACAAATTGTATCAAAAAAATCTTACTCATCACCTTATAACTGCACCTGACATTCTCATCTTGTGTATGCCAACATCTCTGGCTCGAATGGTGTCTTCTTGCAGTTTATCTAGGTAAAGTTGGCTTCATCTATGGGAGTACTTTGCTGCAGCACTTGTACCAAGCCGTGTCCTAGTTCCTGACATTTTCTTAATGTGGGCTAGGTGGTTATGACAATGAAGACAAGGCTGCCAGGGCTTATGATCTGGCTGCTCTGAAATATTGGGGGCCGTCGACGAACACCAATTTCCCGGTATGGATGACAACATGGCTTGGTTCATGGCAACTAAAACATCGATATAGTTCTCTATAATGTGAATTGATGTGTTGCGTTTCACAGCTAGAAACTTATCGAGAGGAGGTCGAGGAGATGAAAAGCATGACAAGGCAGGAATTCATTGCACACTTGAGAAGGTAAAAAAGCAGGAGTCGTTTTTCTCCTACTCTAGATCATTTCTTGATTGGTAGATCATTAATACGTTTGTGATGCCTAAAGGAGAAGCAGCGGGTTTTCTCGTGGTGCTTCGATATATCGAGGAGTGACGAGGTAAGATGCCTCATATTTCCCTCAATTAAGAAAATATTGTACGGGGGAAAAGGTCTGAATACTTTGAGCGGAAAACAATCTCAACACGCCGATGTTGCATTTCAGGCATCATCAGCATGGAAAATGGCAAGCTAGGATTGGCAGGGTTGCTGGGAACAAAGACTTGTATCTCGGCACTTTCAGTAAGAACGACATATCCGCTTGGTTTTATATACTAGTATAACCAAAAGAGTTTTCACAAAGTTATGTGCCATAAGTGAAAGAAGCTTTTAGTGCTAATCTTAAGTAGTACTCCGTCTGATCCAAAATAAGCGTCAGTTTTGAACTGGGTTTAGTTCAAAATGGCGACACTTTTTATGGATCGGAGGGGTGGTAGTAGTAACTACCATTCCTTTAGCGGGTACTATTACCGATGCTATGCTAATTGTCGCGGCAAAGTAGATTTGCCAACCAATAGAAGCTTTGGGATCTACTCAGTTTTACTGTTAACTAACTTTAGTTCGTGGAGTACATCTGCAACTGTAATGCATCATCCTTCCCTGATCATTCTTTGCTTTTCCAGATGTTTTATGCTTTTCATAAGGCTGTGACCTCTGTACAAAGTTATCGGTTGACTGTCTTGAATACTCCCTTGGACTGAAAAATCAATCACACTAGCACGACGCAAAAAGTAATCAGATCGGAAATCGTACTTTTACAATAGGAATCTACCTTGGCTTCCGAATGTATAATCAAGAATGTCATCAACGGCAGCATCTTTTAAAGTAACGTACTTATTATCTTACCATTACGTACGCATGTTTGAAGACCTCTAACATGTACTGAATCTATGTACAGCCACTCAGGAAGAAGCAGCTGAGGCCTATGACGTAGCCGCGATCAAGTTCCGTGGCCTGAACGCCGTAACCAACTTCGACATAACGAGGTACGACGTGGACAAGATCATGGAGAGCAGCTCTCTGCTGCCCGGGGAGGAAGCGCGCAAGGTCAAGCCGATCGAGGCGGCCAACCACGTGCCTGCCAATGCCATGCACAACGGCGGCGCGGAGCTCAGCCATGCCGAAGAAGGAAGCTCCGGCGTCTGGAAGATGGTGCTCCACGGGACACCACAACAAGCAGC

>HvAP2-6

>Protein

MTNGGHSMSGAGGWLGFSLSPQVAAAAMDAAAGSGIVDVAGHHHAHHGGVYYHPDPVASSPMSFYFGGGDNVGAASGGYYSGISALPLRSDGSLCLADALRRSEQKHHGAEVSAPPKLEDFLGAGPAMALSLDNSGYYYGGHSHDNDGAGGGQQPLLYAMMPGSGGHHMYYDAHAALLDEQAAATSAAMEAAGWMARDGDVYDVDAGNGEEGGGAIVPAGPGNPGGYAHPLTLSISSGSQSSCVTVQQAAAQAHAYVGQATAASKKRGAGAGAKQNKQPVVHRKCIDTFGQRTSKYRGVTRHRWTGRYEAHLWDNSCRKEGQTRKGRQVYLGGYDMEEKAGRAYDLAALKYWGASTHINFPVEDYQEELEVMKNMTRLEYVAHIRRKSSGFSRGASMYRGVTRHHQQGRWQARIGRVSGNKDLYLGTFSAEADAAEAYDVAAIKFRGVSAVTNFEISRYDVDKIMESSTLLPADQVRRRKDGPDALVASAAAALVQAGGAADYWRQPVAAASAVTPCGDKQSRHHLDLMSSESLSLLRGVVSMDGDAAGAHGMGNSSSARMSGASSLATSLSNSREQSPDQGGGLAMLFARPAVPKLASSLPMGSWVSSPTPARPGVSVAHMSVFAAWADA

>cDNA

AACTGACGGAAGATCTGATTCATAATCAGTGAGCGGTGAAAGGGACAAGAATGTTAGGATGTAAAATTTGAACTTTTAAAAATTTAAACTAGTGTTGCATGCAGCTATTTAAACGTCTATATATTTTATATGCGACTATTTGACTATACGGACTTGAAATATATCCGACTATGGTTGAATAACGTCCTTCTGTTATGTGGAAGGAAATTTGCGGCAACACCGTTGAAGATGCCCTTAGCGTGTGTGTGGGCAAGACCAAGACAAGAGCAACTCATTCGTTTTGCACACACTTTAAAAGCCAAGCCCAGTACACCCCGTCGACCTCTCCGCCGATCACACCACACCCCGACTCTCCCTCTTCTTCCCCCCATCCCGCTCCCCACGTAGAGTACTACAAGAAGCGCTGAGCCCGGCACATCCCGCACACGCGCACGCACAAACCAAAGTCCCCCTTCAAACCCGCTGAGCCAACAATGGAGAGCAGCCGCATCATTGCGACATGTACCACCCAATGATTGATCCGTCTCATTCCCATCTAAGCTAGATCTTCTTGAATCTTGAGACCACCACAGCCTCATCCAAGCTCGTGCTCGTGCGCCCCTCGCTCCCATCCAATGACCAACGGCGGCCACAGCATGAGCGGCGCGGGCGGCTGGCTGGGGTTCTCGCTCTCGCCTCAAGTCGCCGCCGCGGCCATGGACGCGGCTGCCGGCTCCGGCATCGTCGACGTCGCTGGCCACCACCACGCGCACCACGGCGGGGTCTACTACCACCCCGACCCGGTCGCGTCCTCCCCCATGTCCTTCTACTTCGGGGGCGGCGACAATGTCGGGGCGGCGAGCGGCGGGTACTACTCCGGGATCTCCGCCCTGCCGCTCAGGTCCGACGGCTCGCTCTGTCTCGCCGACGCGCTCCGGAGGAGCGAGCAGAAACACCACGGGGCGGAGGTGTCGGCGCCGCCGAAGCTCGAGGACTTCCTCGGCGCTGGTCCCGCAATGGCGCTGAGCCTGGACAACTCCGGCTACTACTACGGCGGCCACAGCCACGACAACGATGGCGCTGGAGGCGGCCAGCAGCCGCTGCTGTACGCCATGATGCCTGGCTCCGGTGGCCACCACATGTACTACGACGCCCACGCGGCGTTGCTGGACGAGCAGGCTGCAGCCACGTCGGCCGCGATGGAAGCGGCCGGCTGGATGGCGCGTGACGGGGACGTCTACGACGTGGACGCCGGCAACGGCGAGGAGGGCGGGGGCGCCATCGTGCCGGCCGGCCCCGGCAACCCAGGCGGATACGCACACCCGCTGACGCTGTCCATCAGCTCTGGGTCCCAATCCAGCTGCGTCACCGTGCAGCAGGCGGCCGCACAAGCCCACGCCTACGTCGGCCAGGCAACCGCGGCCAGCAAGAAGCGCGGCGCCGGCGCGGGCGCCAAGCAAAACAAGCAGCCGGTGGTCCACCGCAAGTGCATCGACACCTTCGGCCAGCGCACGTCCAAGTACAGGGGCGTCACCAGGCATAGGTGGACGGGGAGGTATGAGGCGCACCTCTGGGACAACAGCTGCCGGAAGGAGGGCCAGACCAGGAAAGGCCGGCAAGTTTATCTTGGTGGGTATGACATGGAGGAGAAGGCGGGGAGGGCGTATGATCTTGCGGCGCTCAAGTACTGGGGCGCGTCCACGCACATCAACTTCCCGGTGGAGGACTACCAGGAGGAGCTGGAGGTGATGAAGAACATGACCAGGCTAGAGTACGTGGCTCACATCAGAAGGAAGAGCAGCGGGTTCTCGCGCGGAGCTTCGATGTACCGGGGAGTCACCAGGCACCACCAGCAGGGGCGGTGGCAGGCGCGCATCGGCCGGGTTTCCGGCAACAAGGACCTCTACCTCGGAACATTCAGCGCGGAGGCGGACGCGGCAGAGGCGTACGACGTGGCGGCGATCAAGTTCCGCGGCGTCAGCGCGGTCACCAACTTCGAAATCAGCCGGTACGACGTGGACAAGATCATGGAGAGCAGCACGCTGCTGCCCGCCGATCAGGTGCGGCGCAGGAAGGACGGCCCCGACGCACTGGTGGCCAGCGCGGCGGCCGCGCTCGTGCAGGCCGGCGGCGCCGCGGACTACTGGAGGCAGCCCGTTGCGGCGGCGTCGGCGGTCACGCCGTGCGGCGACAAGCAGAGCCGCCACCACCTCGACCTCATGTCGAGCGAGTCCTTGTCGCTGCTGCGCGGCGTGGTGTCCATGGACGGCGACGCTGCTGGTGCTCACGGGATGGGAAACTCCAGCAGCGCGCGCATGTCGGGCGCGTCGTCGTTGGCCACGAGCCTGAGCAACTCCCGGGAGCAGAGCCCGGACCAGGGAGGCGGCCTGGCCATGCTGTTCGCCCGGCCCGCGGTGCCGAAGCTGGCGAGCTCGCTGCCCATGGGCTCCTGGGTCTCGTCGCCTACGCCGGCCAGGCCCGGCGTGTCCGTGGCGCACATGTCAGTGTTCGCCGCGTGGGCGGACGCATGAGCAACAACCGCACCGTCATCCTTTTAGGGCTACAACATGCACGGCGGCAAGAAGGTTTAGGGCAAGTAGTACTCAGTAGCCATGGTAGCTATTAGCCTATTACTAGTAGTAAGGAACTAAGGGTTAGTAGTAACCATAGTTAGTAGCTTAGCCGACGCAGGTCGACGGCGAGGGATAAAGCGCGCATGCATGGCTGGGCTCCCGTGGTTCCTTCTTCAGCTGCGTCTGGGACGAAGGGTTTTTGTAGTATCGAGCCTGGCACGGCAACGGCGGCGTCGCCTCCGGCCGAGGGCCGCCGCTGATCGGAGATGGATGGGCAGTAGTAGTTCCTGTCTCCACTCTCCAGACCTCCTAACTTCCATCAAATGAAAATGTGTTCGTCTTGCAACGTTCAGG

>CDS

ATGACCAACGGCGGCCACAGCATGAGCGGCGCGGGCGGCTGGCTGGGGTTCTCGCTCTCGCCTCAAGTCGCCGCCGCGGCCATGGACGCGGCTGCCGGCTCCGGCATCGTCGACGTCGCTGGCCACCACCACGCGCACCACGGCGGGGTCTACTACCACCCCGACCCGGTCGCGTCCTCCCCCATGTCCTTCTACTTCGGGGGCGGCGACAATGTCGGGGCGGCGAGCGGCGGGTACTACTCCGGGATCTCCGCCCTGCCGCTCAGGTCCGACGGCTCGCTCTGTCTCGCCGACGCGCTCCGGAGGAGCGAGCAGAAACACCACGGGGCGGAGGTGTCGGCGCCGCCGAAGCTCGAGGACTTCCTCGGCGCTGGTCCCGCAATGGCGCTGAGCCTGGACAACTCCGGCTACTACTACGGCGGCCACAGCCACGACAACGATGGCGCTGGAGGCGGCCAGCAGCCGCTGCTGTACGCCATGATGCCTGGCTCCGGTGGCCACCACATGTACTACGACGCCCACGCGGCGTTGCTGGACGAGCAGGCTGCAGCCACGTCGGCCGCGATGGAAGCGGCCGGCTGGATGGCGCGTGACGGGGACGTCTACGACGTGGACGCCGGCAACGGCGAGGAGGGCGGGGGCGCCATCGTGCCGGCCGGCCCCGGCAACCCAGGCGGATACGCACACCCGCTGACGCTGTCCATCAGCTCTGGGTCCCAATCCAGCTGCGTCACCGTGCAGCAGGCGGCCGCACAAGCCCACGCCTACGTCGGCCAGGCAACCGCGGCCAGCAAGAAGCGCGGCGCCGGCGCGGGCGCCAAGCAAAACAAGCAGCCGGTGGTCCACCGCAAGTGCATCGACACCTTCGGCCAGCGCACGTCCAAGTACAGGGGCGTCACCAGGCATAGGTGGACGGGGAGGTATGAGGCGCACCTCTGGGACAACAGCTGCCGGAAGGAGGGCCAGACCAGGAAAGGCCGGCAAGTTTATCTTGGTGGGTATGACATGGAGGAGAAGGCGGGGAGGGCGTATGATCTTGCGGCGCTCAAGTACTGGGGCGCGTCCACGCACATCAACTTCCCGGTGGAGGACTACCAGGAGGAGCTGGAGGTGATGAAGAACATGACCAGGCTAGAGTACGTGGCTCACATCAGAAGGAAGAGCAGCGGGTTCTCGCGCGGAGCTTCGATGTACCGGGGAGTCACCAGGCACCACCAGCAGGGGCGGTGGCAGGCGCGCATCGGCCGGGTTTCCGGCAACAAGGACCTCTACCTCGGAACATTCAGCGCGGAGGCGGACGCGGCAGAGGCGTACGACGTGGCGGCGATCAAGTTCCGCGGCGTCAGCGCGGTCACCAACTTCGAAATCAGCCGGTACGACGTGGACAAGATCATGGAGAGCAGCACGCTGCTGCCCGCCGATCAGGTGCGGCGCAGGAAGGACGGCCCCGACGCACTGGTGGCCAGCGCGGCGGCCGCGCTCGTGCAGGCCGGCGGCGCCGCGGACTACTGGAGGCAGCCCGTTGCGGCGGCGTCGGCGGTCACGCCGTGCGGCGACAAGCAGAGCCGCCACCACCTCGACCTCATGTCGAGCGAGTCCTTGTCGCTGCTGCGCGGCGTGGTGTCCATGGACGGCGACGCTGCTGGTGCTCACGGGATGGGAAACTCCAGCAGCGCGCGCATGTCGGGCGCGTCGTCGTTGGCCACGAGCCTGAGCAACTCCCGGGAGCAGAGCCCGGACCAGGGAGGCGGCCTGGCCATGCTGTTCGCCCGGCCCGCGGTGCCGAAGCTGGCGAGCTCGCTGCCCATGGGCTCCTGGGTCTCGTCGCCTACGCCGGCCAGGCCCGGCGTGTCCGTGGCGCACATGTCAGTGTTCGCCGCGTGGGCGGACGCATGA

>DNA

AACTGACGGAAGATCTGATTCATAATCAGTGAGCGGTGAAAGGGACAAGAATGTTAGGATGTAAAATTTGAACTTTTAAAAATTTAAACTAGTGTTGCATGCAGCTATTTAAACGTCTATATATTTTATATGCGACTATTTGACTATACGGACTTGAAATATATCCGACTATGGTTGAATAACGTCCTTCTGTTATGTGGAAGGAAATTTGCGGCAACACCGTTGAAGATGCCCTTAGCGTGTGTGTGGGCAAGACCAAGACAAGAGCAACTCATTCGTTTTGCACACACTTTAAAAGCCAAGCCCAGTACACCCCGTCGACCTCTCCGCCGATCACACCACACCCCGACTCTCCCTCTTCTTCCCCCCATCCCGCTCCCCACGTAGAGTACTACAAGAAGCGCTGAGCCCGGCACATCCCGCACACGCGCACGCACAAACCAAAGTCCCCCTTCAAACCCGCTGAGCCAACAATGGAGAGCAGCCGCATCATTGCGACATGTACCACCCAATGATTGATCCGTCTCATTCCCATCTAAGCTAGATCTTCTTGAATCTTGAGACCACCACAGCCTCATCCAAGCTCGTGCTCGTGCGCCCCTCGCTCCCATCCAATGACCAACGGCGGCCACAGCATGAGCGGCGCGGGCGGCTGGCTGGGGTTCTCGCTCTCGCCTCAAGTCGCCGCCGCGGCCATGGACGCGGCTGCCGGCTCCGGCATCGTCGACGTCGCTGGCCACCACCACGCGCACCACGGCGGGGTCTACTACCACCCCGACCCGGTCGCGTCCTCCCCCATGTCCTTCTACTTCGGGGGCGGCGACAATGTCGGGGCGGCGAGCGGCGGGTACTACTCCGGGATCTCCGCCCTGCCGCTCAGGTCCGACGGCTCGCTCTGTCTCGCCGACGCGCTCCGGAGGAGCGAGCAGAAACACCACGGTAAATATCTGCACAGTACTTACCACGGTTCTTACTTTGCTACAGTTTGGGGTTGCTGAGCTCTTGGCTTCTAGCTAGCTGCGCCTGCGCGTGTCTCTTTATGTTTCTTGGTTGGGCTTGCGGTCTGCGCAGGGGCGGAGGTGTCGGCGCCGCCGAAGCTCGAGGACTTCCTCGGCGCTGGTCCCGCAATGGCGCTGAGCCTGGACAACTCCGGCTACTACTACGGCGGCCACAGCCACGACAACGATGGCGCTGGAGGCGGCCAGCAGCCGCTGCTGTACGCCATGATGCCTGGCTCCGGTGGCCACCACATGTACTACGACGCCCACGCGGCGTTGCTGGACGAGCAGGCTGCAGCCACGTCGGCCGCGATGGAAGCGGCCGGCTGGATGGCGCGTGACGGGGACGTCTACGACGTGGACGCCGGCAACGGCGAGGAGGGCGGGGGCGCCATCGTGCCGGCCGGCCCCGGCAACCCAGGCGGATACGCACACCCGCTGACGCTGTCCATCAGCTCTGGGTCCCAATCCAGCTGCGTCACCGTGCAGCAGGCGGCCGCACAAGCCCACGCCTACGTCGGCCAGGCAACCGCGGCCAGCAAGAAGCGCGGCGCCGGCGCGGGCGCCAAGCAAAACAAGCAGCCGGTGGTCCACCGCAAGTGCATCGACACCTTCGGCCAGCGCACGTCCAAGTACAGGGGCGTCACCAGGTAGCCGCGCGGCACAACCCATAACTCATTGCATGTCTTCCTTGTTTGTCAGTCACCATCGTCTTCCTCCTCGCGCTAACCCCCGCTTGCTAGCAAAAGCAGGCATAGGTGGACGGGGAGGTATGAGGCGCACCTCTGGGACAACAGCTGCCGGAAGGAGGGCCAGACCAGGAAAGGCCGGCAAGGTAGTACTTCCAAGTTCCAACCTTAATCAATCAAGCTCTGTAACTAGTAGTAGAACTTGGCCCCTGGTACCATTTTTGTTTGTTTAGTTCTAGTAGCACTACGTACCACTAGCAGCACTCTCCTAAGTAGTGATGTGAGTGCACTTCCAACTGCTAACTAATGAACTGCTCTTTGCATGTCATTTGCTGGACCTTTTGGCCTGGGATCTATCGTGCTTCTCCTCGTCTCCATCTGCAGTTTATCTTGGTGAGTACCACTGCCACTGGGAGTGATACCTGCTGGGATCAGTCAGTACAAATGTTACTACTACATTTTTGCACATGTGTGTAATCAGTGGGTTCAAGTACTACTGTACTTTAGTTATTTTCTTGTGGGATCAGTCAAACACAAATACAAGTACTCCATTTTTGCGCATGTCTAATCAGACGGAGTGGTGCTTACAGGAGTTTGTTGATTTGTGCAGGTGGGTATGACATGGAGGAGAAGGCGGGGAGGGCGTATGATCTTGCGGCGCTCAAGTACTGGGGCGCGTCCACGCACATCAACTTCCCGGTACGCACGCGATCACCTACTTATCGATCCATCTCCGGTGTCCATTGCTCTACATTGTTTGCTAGCTTGTGATAAAAATGTAGCCACTAGCCAGTGATTTTGACCTTTGTTTGTGTGATTTCGTGCAGGTGGAGGACTACCAGGAGGAGCTGGAGGTGATGAAGAACATGACCAGGCTAGAGTACGTGGCTCACATCAGAAGGTAGATAACCTAGTACTTGCCTGAATCCTGAGAGAAATGTGCACTCGATGTACGCATGGCGGACACTCACCTCTCGGATCGCCATTGTTGCAGGAAGAGCAGCGGGTTCTCGCGCGGAGCTTCGATGTACCGGGGAGTCACCAGGCACCACCAGCAGGGGCGGTGGCAGGCGCGCATCGGCCGGGTTTCCGGCAACAAGGACCTCTACCTCGGAACATTCAGTAAGCGCTCAGCCCCAGCTAGCTCTTCCTCTCCCAACCGCCGCCGTTAATATCGTTTCCCTGAGACTGCAACCTCGCAATCTCGCTACAAGATAGTACTAGTACCTAGTGGACTACAAATCTGCAGCATGCATGCTCCTATCATGCATCACCATGATCACCGATTCACCAAAGTGACAGCAGCAACTAGTTCGTTGTCGCCGCAGCAAAGCAATGGCTGCAGAGGCTTGTCACGGTTTAAGGACGCAGGGCGTCCTGTCACAATGATCATAACCTCTGATGTGGCTCAATGATGCCCCTTGCCCCTCCCGAAAGAATGAACGTAGCATCGCTCTGCCGATTCCTTGCGTGCTTTTGGCCGCACTTTTGCGCGTGCTAGGCCGCGCCCAGTGGCATTGCATCGGCCATCGGCGGTAGTGGGTTTACGTTTGGGTGTTGCTCGCCTTTCTCATAAGGCTGGCAGGCCCTGAGCATGCATGCATGCTCTCGTAGCTTTCGCGCTTGGCTCCTGTTCTGTTGTTTGACCGCCGGGAAAGCCCGGCTGCTGTTTGAGGCTCGGTTTGTGATCGATCGTTGGATGTGTGTTTTCTTTGCGTGCAGGCGCGGAGGCGGACGCGGCAGAGGCGTACGACGTGGCGGCGATCAAGTTCCGCGGCGTCAGCGCGGTCACCAACTTCGAAATCAGCCGGTACGACGTGGACAAGATCATGGAGAGCAGCACGCTGCTGCCCGCCGATCAGGTGCGGCGCAGGAAGGACGGCCCCGACGCACTGGTGGCCAGCGCGGCGGCCGCGCTCGTGCAGGCCGGCGGCGCCGCGGACTACTGGAGGCAGCCCGTTGCGGCGGCGTCGGCGGTCACGCCGTGCGGCGACAAGCAGAGCCGCCACCACCTCGACCTCATGTCGAGCGAGTCCTTGTCGCTGCTGCGCGGCGTGGTGTCCATGGACGGCGACGCTGCTGGTGCTCACGGGATGGGAAACTCCAGCAGCGCGCGCATGTCGGGCGCGTCGTCGTTGGCCACGAGCCTGAGCAACTCCCGGGAGCAGAGCCCGGACCAGGGAGGCGGCCTGGCCATGCTGTTCGCCCGGCCCGCGGTGCCGAAGCTGGCGAGCTCGCTGCCCATGGGCTCCTGGGTCTCGTCGCCTACGCCGGCCAGGCCCGGCGTGTCCGTGGCGCACATGTCAGTGTTCGCCGCGTGGGCGGACGCATGAGCAACAACCGCACCGTCATCCTTTTAGGGCTACAACATGCACGGCGGCAAGAAGGTTTAGGGCAAGTAGTACTCAGTAGCCATGGTAGCTATTAGCCTATTACTAGTAGTAAGGAACTAAGGGTTAGTAGTAACCATAGTTAGTAGCTTAGCCGACGCAGGTCGACGGCGAGGGATAAAGCGCGCATGCATGGCTGGGCTCCCGTGGTTCCTTCTTCAGCTGCGTCTGGGACGAAGGGTTTTTGTAGTATCGAGCCTGGCACGGCAACGGCGGCGTCGCCTCCGGCCGAGGGCCGCCGCTGATCGGAGATGGATGGGCAGTAGTAGTTCCTGTCTCCACTCTCCAGACCTCCTAACTTCCATCAAATGAAAATGTGTTCGTCTTGCAACGTTCAGG

>HvAP2-7

>Protein

MATTVQPHSPDPTAITSPPPPPPPPRQEDLTAAGEGVEIAALDEQPAHVAVAVADKGKTAPGGGKLVAEAMRKCAAPRSSRYHGVTRLKWSGKYEAHLWDNTSQVEGRKRKGKHVYLGSYVTEENAARAHDLAALKYWGISQPTKLNFNISDYVKEIEIMKSMNQDEFVAYIRRQSSCFSRGTSSYRGVTRRKDDKWQARIGRIGESRDTKDIYLGTFETEVEAAEAYDLAAIQLRGVHAVTNFDVSNYSEEGLKKLEGSSVVVNLEDQSEVTKLAVTNFDISKHCEDGLKKLDGAS

>cDNA

CAAACCCTATGATCGGGCCAAGGCAGCGAAGCGAATCAGGCGGAACAGGACGCAGTCACGCAGCTGCTCTTTCCTCCACCCGCCTCCCCCGTAGGGGTAGCTAGGGTTTCTCCGTCCAAATCGTGAATAATAGTCCACTTGAAGATTCGCGCGAAAGCCGCGGCGATGGCCACCACCGTCCAACCCCACTCCCCGGACCCAACAGCCATCACCAGCCCCCCTCCACCTCCTCCTCCACCTCGCCAGGAGGACCTGACCGCCGCGGGGGAGGGCGTAGAGATCGCGGCACTCGATGAGCAGCCTGCCCACGTCGCCGTCGCCGTCGCCGACAAGGGGAAGACGGCTCCCGGCGGCGGGAAGCTGGTGGCGGAGGCCATGCGCAAGTGCGCGGCGCCCCGGTCGTCGCGCTACCACGGCGTGACGAGGCTCAAGTGGAGCGGCAAGTACGAGGCACACCTCTGGGACAACACCAGCCAGGTTGAGGGGCGCAAGCGCAAGGGCAAGCATGTGTACTTGGGAAGCTATGTTACTGAAGAGAATGCTGCAAGGGCGCATGACCTTGCAGCCCTGAAATATTGGGGCATAAGTCAACCCACCAAACTAAACTTCAATATTTCTGATTATGTAAAAGAAATTGAGATCATGAAGAGCATGAATCAAGATGAATTTGTGGCCTACATAAGGAGGCAGAGCAGTTGTTTCTCAAGAGGAACTTCATCATACAGGGGTGTAACAAGACGAAAGGATGATAAATGGCAAGCACGTATTGGTAGGATTGGTGAGAGTAGAGACACTAAAGACATCTATCTTGGGACCTTTGAAACTGAAGTGGAGGCAGCTGAAGCGTATGACCTAGCAGCAATTCAGCTCCGTGGTGTTCATGCTGTGACCAACTTTGATGTCAGCAACTACTCCGAAGAAGGTTTGAAGAAACTAGAAGGCTCATCCGTGGTAGTGAACCTGGAGGACCAATCAGAAGTCACTAAGTTAGCTGTGACCAATTTTGATATTAGCAAACACTGCGAAGATGGTTTGAAGAAACTAGATGGCGCATCCTAGGTAGTGAACCTGGAGGGCCAATCAGAAGTCACCAAGTTATCTGTGACTAACTTTGATATTAGCAACTACCGTGAAGATGGTTTGAAGAAACTAGAAGCCTCATCGGAGATAGCGAACCTGGAGGGCCAATCAGAAGTCACAAAGTTGGCTGGACAATAAATATTAGAATAGCAACATGTAAATTATTTATTTCTTCCTTTTTATCTTTTCTCTGATCATCAATTTTCCCACCTTTTTTCTTTTCCTGATGAGAGTGTCTTTTCTTCGTCCACTTTTTCTGAAGCCCTTCTATTCTCAAGGAATTTCCATGGGCTGGTTGCCAACTCTGGTATTTACCAGCTACAGAGTAGGATCGTATGATGCTGTACTCTGTATGTTCATGTACACTGATTACAAACCGTTGCAAGGCAATGAAAGCTCTATTTTTGCGGAAATTGCATTTGGAATGGAGGCC

>CDS

ATGGCCACCACCGTCCAACCCCACTCCCCGGACCCAACAGCCATCACCAGCCCCCCTCCACCTCCTCCTCCACCTCGCCAGGAGGACCTGACCGCCGCGGGGGAGGGCGTAGAGATCGCGGCACTCGATGAGCAGCCTGCCCACGTCGCCGTCGCCGTCGCCGACAAGGGGAAGACGGCTCCCGGCGGCGGGAAGCTGGTGGCGGAGGCCATGCGCAAGTGCGCGGCGCCCCGGTCGTCGCGCTACCACGGCGTGACGAGGCTCAAGTGGAGCGGCAAGTACGAGGCACACCTCTGGGACAACACCAGCCAGGTTGAGGGGCGCAAGCGCAAGGGCAAGCATGTGTACTTGGGAAGCTATGTTACTGAAGAGAATGCTGCAAGGGCGCATGACCTTGCAGCCCTGAAATATTGGGGCATAAGTCAACCCACCAAACTAAACTTCAATATTTCTGATTATGTAAAAGAAATTGAGATCATGAAGAGCATGAATCAAGATGAATTTGTGGCCTACATAAGGAGGCAGAGCAGTTGTTTCTCAAGAGGAACTTCATCATACAGGGGTGTAACAAGACGAAAGGATGATAAATGGCAAGCACGTATTGGTAGGATTGGTGAGAGTAGAGACACTAAAGACATCTATCTTGGGACCTTTGAAACTGAAGTGGAGGCAGCTGAAGCGTATGACCTAGCAGCAATTCAGCTCCGTGGTGTTCATGCTGTGACCAACTTTGATGTCAGCAACTACTCCGAAGAAGGTTTGAAGAAACTAGAAGGCTCATCCGTGGTAGTGAACCTGGAGGACCAATCAGAAGTCACTAAGTTAGCTGTGACCAATTTTGATATTAGCAAACACTGCGAAGATGGTTTGAAGAAACTAGATGGCGCATCCTAG

>DNA

GCGAGCGCGGAAGAGTCGCACGCGCGGGAGCCGGCGCAACAAATAAGGGGCGCGGGATTGCGTTTCGGCCAGCGCGCTGAAATGAAAATGGCGCGTGCACCGTTTTTGCACGCCTGCTGGAGCTGCCCGCCGCGTTGCGCTCGCGCTAAAACTGACTTTTTTACTGCGCGGCGCTTGTATAGGGCGCGTGCTGGAGATGCTGTGACAAACCCTATGATCGGGCCAAGGCAGCGAAGCGAATCAGGCGGAACAGGACGCAGTCACGCAGCTGCTCTTTCCTCCACCCGCCTCCCCCGTAGGGGTAGCTAGGGTTTCTCCGTCCAAATCGTGAATAATAGTCCACTTGAAGATTCGCGCGAAAGCCGCGGCGATGGCCACCACCGTCCAACCCCACTCCCCGGACCCAACAGCCATCACCAGCCCCCCTCCACCTCCTCCTCCACCTCGCCAGGAGGACCTGACCGCCGCGGGGGAGGGCGTAGAGATCGCGGCACTCGATGAGCAGCCTGCCCACGTCGCCGTCGCCGTCGCCGACAAGGGGAAGACGGCTCCCGGCGGCGGGAAGCTGGTGGCGGAGGCCATGCGCAAGTGCGCGGCGCCCCGGTCGTCGCGCTACCACGGCGTGACGAGGTGGGCAGGCTAGGAAGGACGTTGTTACTGTTCATTGATAGGGCTTGCTGTCCTTGCGTGTTGTTGACTCGATTGTTTGCGTTCGTATGTATATACTGGGACAGGCTCAAGTGGAGCGGCAAGTACGAGGCACACCTCTGGGACAACACCAGCCAGGTTGAGGGGCGCAAGCGCAAGGGCAAGCATGGTGCGTAATTTTGCTGTCCACACTGTAGCCTGCTCATTCAATGGTTTTCAGAGCCCAACGAAACTACCTAGTATGGTTTAACTTGGAATGCTGAGAGGGAACTGATCAGTTATCAGTTGCCCTCGAATAGAAACAGAGGGTTGGTTGCAAGGTGCACATACCGTGTCTTCTAATTTACAATATCATAATATGTGGCACCACAATTCGAGTTACTAAATGCAAACTAGGTGCTAATATAGGATGATTGCTGCTTTGGATTAATAATTATTTGTACGGGAATGCAGGAACATCTTTGTTGGATCAGTACATTGATTATTGATATTCATTCTTAATATGACACCTTGAAACCCTTGGTTGATGTAGGATGATAAATTGTGTGCAAGCGCAATAGAGTACACCTTTTTTTCTTTAATAAGAGGAAAGGGTATTACTAAATATCTCTATTAAGCTTTTCTCTTCTTTGCGGATGATGTGGTGCTAGGTGATGATAGTCGGACGGGGTCAATACGAAGTTAGAGCTATGGAGACAAATCTTGGAATCGAAAGGTTTTAGACTTNNNNNNACTAAAACCGAGTACATGAGGTGCGGTTTCAGTACTACTAGGCACGAGGAGGAGGAGGTTAGCCTTGATGAGCAGGTGGTGCCCCAGAAGGGCACCCTGCAATATTTGGTGTCAGTGTTGTAGAAGGACGGGGATATCGATCAAGATGTTAACCTAACCATCGAATCATTCAGATGGATGAAGTGGTGCCTAGCTTTTGGCGTTCTATGTGACAGGAGAGTGCCAGAAAAGCTAGGCAGACTAAAAGGCAACATGTTCAACAATTAGGTGTGGCGGAGTTGCACATGTTGAGATGGAGATGTGTCGCCACACAAGGAAGGACCGGGTCCGGAATGATAATATATGAGATAGAGTTCGGGTAGCACCAATCGAAGAGAAGTTTGTCCAACGTCGTCTGAGATGGTTTGGGCATATTCAGTGCAGGTCTCCAGAAGTTTCATTGTGTAGCAGACGGCTAAAGTGTGCTGATAATGCCAAGAGAGGTCGGGGTAGGCCGAACTTGACATGGGAGGAGTCTTTAAACAAATAGAGATTTGAGGGACTGGAGTATCGCCAAAGAACTAGCCGTAGACAGGGGTGCACGGAACGTAGCTATCCATGTGCCAGAACCCATGAGTTGGTTGCGAGATCTTATGGGTTTCACCTCTAGCCTACCCCAACTTGTTTCGGACTAAAGGCTTTGTTGTAAGCTTTTCTCTTGTTTCTGTAGGAATTGGGCATTGGAAATTCTACTGTTTTTTGTTATAAAATGGAACGTTTAATTGTGCACTTAGCATCTTCACTCCCCCTAACTGATTTTTTTTGTTTGGATATTCTGCATGGGATCTGTTTCTACTTTTGGCTGCCATTGTGGTTCTTCAGTGTACTTGGGTAACAACTTATACTGCCTGCTATCATAGAGTGAGTTTGGATTGAATATAAAAACAGCTCAATTTGATCTTCATTTTGGCATTACAGGAAGCTATGTTACTGAAGAGAATGCTGCAAGGGCGCATGACCTTGCAGCCCTGAAATATTGGGGCATAAGTCAACCCACCAAACTAAACTTCAATGTATGGAGTGTTTGTTGTTTTGTGTATTTGAGGAACTGGCATCTTGTTATACAGGAAAATAGAATTAACTTCTAATTGTTCTCCAGATTTCTGATTATGTAAAAGAAATTGAGATCATGAAGAGCATGAATCAAGATGAATTTGTGGCCTACATAAGGAGGTCCGTTATATAGTTGAATATTGCAGGTTTGAAACCTATGTACTAGTTTTTCAAAGTTTTTATGATTATTTCAGGCAGAGCAGTTGTTTCTCAAGAGGAACTTCATCATACAGGGGTGTAACAAGGTTCTATTCTGTACTTCAGGAATATATATAAAGAGTTATCTAGTCTAGTGAAATAAATTTGTAGGATTTTGATCCATCTTGTTTCTTTTTGCAACTGAAAATTTTCTGCTGTGTTCAAACTTAAGAATTCCGCATGTTTGGTTCTACAGCAAATTTTAACTTTATCTATTCGCTTGGACTAAACAGACGAAAGGATGATAAATGGCAAGCACGTATTGGTAGGATTGGTGAGAGTAGAGACACTAAAGACATCTATCTTGGGACCTTTGGTGAGTACAAATTGTAACTGATGGCATTAACCTTGTGTTGAAAACTATTTTGAATGAGGAAATCACTATAAGTTGAAACTTGTTGCTAATGAATAAGGAATTATGCTTGGTTCGCAGAAACTGAAGTGGAGGCAGCTGAAGCGTATGACCTAGCAGCAATTCAGCTCCGTGGTGTTCATGCTGTGACCAACTTTGATGTCAGCAACTACTCCGAAGAAGGTTTGAAGAAACTAGAAGGCTCATCCGTGGTAGTGAACCTGGAGGACCAATCAGAAGTCACTAAGTTAGCTGTGACCAATTTTGATATTAGCAAACACTGCGAAGATGGTTTGAAGAAACTAGATGGCGCATCCTAGGTAGTGAACCTGGAGGGCCAATCAGAAGTCACCAAGTTATCTGTGACTAACTTTGATATTAGCAACTACCGTGAAGATGGTTTGAAGAAACTAGAAGCCTCATCGGAGATAGCGAACCTGGAGGGCCAATCAGAAGTCACAAAGTTGGCTGGACAATAAATATTAGAATAGCAACATGTAAATTATTTATTTCTTCCTTTTTATCTTTTCTCTGATCATCAATTTTCCCACCTTTTTTCTTTTCCTGATGAGAGTGTCTTTTCTTCGTCCACTTTTTCTGAAGCCCTTCTATTCTCAAGGAATTTCCATGGGCTGGTTGCCAACTCTGGTATTTACCAGCTACAGAGTAGGATCGTATGATGCTGTACTCTGTATGTTCATGTACACTGATTACAAACCGTTGCAAGGCAATGAAAGCTCTATTTTTGCGGAAATTGCATTTGGAATGGAGGCC

>HvAP2-8

>Protein

MKSGEEVSQGQQMDGFVEEKAAGESRDGRKIERSPSINLNSLPAMAPAAAEIGLLHGAAESEANDASTQKGDESSGTDQKKVPKNEEVDEGEIQGRADMKSDSVDPLNSENHAGEKDALVTVPENEGRADGGDNYKGVQVLSIVKKDEPEEIVDSINPVTVAEYREEKGATSSTSAITAVRAPGSRSSCYHGVTRHRWSGKYEAHLWDSTCRVEGRRRKGKQVYLGSYDTEQKAARAYDVAALKYWGLNTKLNFSISEYEKELADIQDMSPEECVTYLRRRSSCFSRGASIYRGVTRRQKDGRWQARIGLIAGTRDIYLGTFKTEEEAAEAYDIAAIEIRGKNAVTNFDRSNYMDRGMHCIEGAGLKLLATKPE

>cDNA

GGGGCCTCCGAGGCACGGACCGCCAGGGTCCTCCAGCCTGGCACGCATCACGAGGGCGTCGTCCACGAAGCAAAACTCCCACATCACCATTATATTTATTTATATTTATATAAAACGAGCATTTTGTTTACTCCTCCTGATGTATAGTGGTACTCCTACCAGTACCAGTAACCGCTGCAGGTGGCGGCAGTAAAAGATCCAGCAAATATCCGATGGTTTCGGAGCGCCAGTGCGGCGGCGCGAGATAAAATCCGCCCCCCACCCCCACACGCGATTTCCCCCACTCTCCCCGTTTCCTCCCTCGATTTCTCCAAATCTTTTCTTCTCCTTCTCCACCAGCGATTAGTTTGTTGTTTCCGGCATCACTCCGCAATAAGCCGCCCCCGCCCCCGCCTCCGCCGCGCTGGCCCTCGTCGTTTCCTTCCCCAATTCCGCCGCCCCTCCCAGCCCGATATTTATTCCCTGCCTCGGCATCCATTTCAAGTTGGTAGATTTTTCCGGCTCGTCGTTGCTAGTGGTAATATCCGCGCTGGGATATTTCCCCTTTTGCTTTCTTGGCCGCGCGCGTCCAGCCCCCCCGGTGGCCTCCGGATCTTTCGATCGCCGCGAGCAGGCGGCTCAAGGTACCTTCTTGTTTTCCGATTCTGGCAGCTAGAAGAAGAAGAAGAAGAAGAAGAAGAAGAAGTTGTGTTGGGCGATGGAATGATTTCCTGGTTGGGATCTAACCGTTTGATTCTTTTTTCTTGTTTGGGATGCTCTGTCTTTGTCCCCCCTCTTTCAGATAGTTCGTGAATAGGACGGCTGCTAGGCAGGTTAGGTTCGTGATAGACGATGAAATCCGGGGAGGAAGTTAGTCAGGGTCAGCAAATGGACGGTTTTGTGGAGGAGAAAGCTGCTGGGGAGTCGAGGGATGGTCAGAAGATCGAGAGGAGCCCTTCTATCAATCTGAATTCCTTGCCTGCAATGGCCCCTGCCGCTGCGGAGATTGGTGTCTTGCACGGCGCAGCGGAGTCAGAGGCCAACGATGCAAGCACTCAGAAGGGAGATGAGTCCAGTGGCACTGATCAGAAGAAGGTCCCGAAGAATGAGGAAGTTGATGAAGGCGAAATTCAGGGCCGTGCAGACATGAAGAGCGACTCGGTTGACCCTTTGAACAGCGAGAACCATGCCGGGGAGAAGGATGCTTTGGTAACTGTGCCAGAAAATGAGGGGCGTGCGGATGGTGGCGATAATTATAAGGGAGTTCAAGTTCTCAGCGTTGTCAAAAAGGATGAGCCTGAGGAAATTGTTGATTCTATTAATCCTGTAACGGTTGCGGAGTATAGAGAGGAGAAGGGCGCCACCAGTTCTACTTCTGCAATTACTGCGGTGCGAGCACCAGGCTCCCGGTCGTCTTGTTACCATGGTGTGACCAGGCATAGGTGGAGTGGGAAATATGAAGCTCATTTGTGGGACAGTACTTGCAGAGTAGAAGGACGGAGAAGGAAAGGGAAGCAAGGTTATGCAGGAAGTTATGATACTGAGCAAAAAGCTGCCAGGGCATATGATGTTGCAGCTCTTAAATACTGGGGACTAAATACAAAGCTGAACTTTTCGATTTCAGAATATGAAAAGGAACTGGCGGACATACAAGACATGTCTCCAGAGGAATGTGTGACATACTTGCGAAGGAGGAGCAGCTGCTTCTCAAGAGGGGCTTCTATTTACAGAGGAGTTACAAGGAGGCAGAAAGATGGTCGATGGCAGGCCCGCATAGGACTGATTGCTGGAACTAGAGACATTTACCTTGGAACTTTCAAAACCGAGGAAGAAGCTGCAGAAGCATATGATATTGCTGCCATTGAGATACGTGGGAAAAACGCGGTGACCAACTTTGACCGAAGCAACTACATGGACAGGGGCATGCATTGTATAGAAGGGGCAGGGTTGAAGCTGCTTGCAACGAAGCCAGAATGATTTGATATCGTATATTGAACAGATTTGGTAAGCCATATTTTGGGGCTTAGTGGTACATACAAGATAGAAGAACTGGTCGCAGCCTGTCAGTATTTGCTGCTGTATGATTCTTCAGATTATATATAGTTCTTCCAGAGAGAATTTCAGTCATTTAGCAAGCTTTGTGTCCAGGACAAGATTTTGACCATGTATTACTGTTATAGT

>CDS

ATGAAATCCGGGGAGGAAGTTAGTCAGGGTCAGCAAATGGACGGTTTTGTGGAGGAGAAAGCTGCTGGGGAGTCGAGGGATGGTCAGAAGATCGAGAGGAGCCCTTCTATCAATCTGAATTCCTTGCCTGCAATGGCCCCTGCCGCTGCGGAGATTGGTGTCTTGCACGGCGCAGCGGAGTCAGAGGCCAACGATGCAAGCACTCAGAAGGGAGATGAGTCCAGTGGCACTGATCAGAAGAAGGTCCCGAAGAATGAGGAAGTTGATGAAGGCGAAATTCAGGGCCGTGCAGACATGAAGAGCGACTCGGTTGACCCTTTGAACAGCGAGAACCATGCCGGGGAGAAGGATGCTTTGGTAACTGTGCCAGAAAATGAGGGGCGTGCGGATGGTGGCGATAATTATAAGGGAGTTCAAGTTCTCAGCGTTGTCAAAAAGGATGAGCCTGAGGAAATTGTTGATTCTATTAATCCTGTAACGGTTGCGGAGTATAGAGAGGAGAAGGGCGCCACCAGTTCTACTTCTGCAATTACTGCGGTGCGAGCACCAGGCTCCCGGTCGTCTTGTTACCATGGTGTGACCAGGCATAGGTGGAGTGGGAAATATGAAGCTCATTTGTGGGACAGTACTTGCAGAGTAGAAGGACGGAGAAGGAAAGGGAAGCAAGGTTATGCAGGAAGTTATGATACTGAGCAAAAAGCTGCCAGGGCATATGATGTTGCAGCTCTTAAATACTGGGGACTAAATACAAAGCTGAACTTTTCGATTTCAGAATATGAAAAGGAACTGGCGGACATACAAGACATGTCTCCAGAGGAATGTGTGACATACTTGCGAAGGAGGAGCAGCTGCTTCTCAAGAGGGGCTTCTATTTACAGAGGAGTTACAAGGAGGCAGAAAGATGGTCGATGGCAGGCCCGCATAGGACTGATTGCTGGAACTAGAGACATTTACCTTGGAACTTTCAAAACCGAGGAAGAAGCTGCAGAAGCATATGATATTGCTGCCATTGAGATACGTGGGAAAAACGCGGTGACCAACTTTGACCGAAGCAACTACATGGACAGGGGCATGCATTGTATAGAAGGGGCAGGGTTGAAGCTGCTTGCAACGAAGCCAGAATGA

>DNA

AACAAACGCACCATACAAAAGAATTGCATCGGATAGATCAAAGCGAAGATGCTATGACCATTGTATTGAAGATCAAGATATAGGTACTAACTACGGACCCCTAGGTCTGTGGTAAACTACTTACACATCATCATGAGGGCAGCAAGGTTGATGAAGTGGCTCTCCATGATCGATCCCCCTCTCGCAGGGTGCCGGAAGGAGCTCTAGATGGATTCACCGTGGAACAGAGCTTGCGGCCGCAAAAAAAGTGTTTCACGGTCGTCCTTAGGGATTTTAGAATATATGTGAATTTATAAGCCAGAGAACGTAGGTAGAGAGACCACCAGGAAGGGGCAAGCCACCGTTATGGACGTGATGCATATTCATCAATTGTGCAGGCATAGGCGGAGTGGAAAATATGAAGCCTTGTATATTAAAATGAAGGGAGTATACAGATATAGATTTAGGAAATGTAAAATACTAAGATATTGGGAATGCATAAAATCAAGTACGAAATCTGAAGGTCAGTATCCACATCCGATCACGTCCTCACACGTTTGAGGGTTTTGATGTCCGTCCGTAGTGTAGATGCTCTCATCTCCTCCCCGCAGCTCCGAGCCACCGTTACATCCCCTAGAACGTTCCAGCACGCACTCTCAGTTCTCATACCTCGTGAATGGCTCAGATCTTCACCCCCACACTGGCAACACATTTTCTCTAGCCACGTGTACCTGCCACAGAACGGGGCCTCCGAGGCACGGACCGCCAGGGTCCTCCAGCCTGGCACGCATCACGAGGGCGTCGTCCACGAAGCAAAACTCCCACATCACCATTATATTTATTTATATTTATATAAAACGAGCATTTTGTTTACTCCTCCTGATGTATAGTGGTACTCCTACCAGTACCAGTAACCGCTGCAGGTGGCGGCAGTAAAAGATCCAGCAAATATCCGATGGTTTCGGAGCGCCAGTGCGGCGGCGCGAGATAAAATCCGCCCCCCACCCCCACACGCGATTTCCCCCACTCTCCCCGTTTCCTCCCTCGATTTCTCCAAATCTTTTCTTCTCCTTCTCCACCAGCGATTAGTTTGTTGTTTCCGGCATCACTCCGCAATAAGCCGCCCCCGCCCCCGCCTCCGCCGCGCTGGCCCTCGTCGTTTCCTTCCCCAATTCCGCCGCCCCTCCCAGCCCGATATTTATTCCCTGCCTCGGCATCCATTTCAAGTTGGTAGATTTTTCCGGCTCGTCGTTGCTAGTGGTAATATCCGCGCTGGGATATTTCCCCTTTTGCTTTCTTGGCCGCGCGCGTCCAGCCCCCCCGGTGGCCTCCGGATCTTTCGATCGCCGCGAGCAGGCGGCTCAAGGTACCTTCTTGTTTTCCGATTCTGGCAGCTAGAAGAAGAAGAAGAAGAAGAAGAAGAAGAAGTTGTGTTGGGCGATGGAATGATTTCCTGGTTGGGATCTAACCGTTTGATTCTTTTTTCTTGTTTGGGATGCTCTGTCTTTGTCCCCCCTCTTTCAGATAGTTCGTGAATAGGACGGCTGCTAGGCAGGTTAGGTTCGTGATAGACGATGAAATCCGGGGAGGAAGTTAGTCAGGGTCAGCAAATGGACGGTTTTGTGGAGGAGAAAGCTGCTGGGGAGTCGAGGGATGGTCAGAAGATCGAGAGGAGCCCTTCTATCAATCTGAATTCCTTGCCTGCAATGGCCCCTGCCGCTGCGGAGATTGGTGTCTTGCACGGCGCAGCGGAGTCAGAGGCCAACGATGCAAGCACTCAGAAGGGAGATGAGTCCAGTGGCACTGATCAGAAGAAGGTCCCGAAGAATGAGGAAGTTGATGAAGGCGAAATTCAGGGCCGTGCAGACATGAAGAGCGACTCGGTTGACCCTTTGAACAGCGAGAACCATGCCGGGGAGAAGGATGCTTTGGTAACTGTGCCAGAAAATGAGGGGCGTGCGGATGGTGGCGATAATTATAAGGGAGTTCAAGTTCTCAGCGTTGTCAAAAAGGATGAGCCTGAGGAAATTGTTGATTCTATTAATCCTGTAACGGTTGCGGAGTATAGAGAGGAGAAGGGCGCCACCAGTTCTACTTCTGCAATTACTGCGGTGCGAGCACCAGGCTCCCGGTCGTCTTGTTACCATGGTGTGACCAGGTAAGCTTGGTTTTGTGTTCCTATAATTGGTATTTGATCAGAGGAAAGAAACACAACAGTGTGCTTCACTATATTAACTAAACAAATTATTAGATAGAGCTGCTTACCAGACTTCATCGATTGTGCAGGCATAGGTGGAGTGGGAAATATGAAGCTCATTTGTGGGACAGTACTTGCAGAGTAGAAGGACGGAGAAGGAAAGGGAAGCAAGGTATGCGTCTTTTATTTGAAGCTATCTAATGGGCATTTATATACATTTTATCTCTGCGTCACGACCTATTTATAAAGTACGCATTTGTGATAGATGTGGTTTATGCTTATAGCCCCGTTGTCGGGGGTCCATTGTAGAGTGTGTGGCTAGGTCTAGGGCATCATGCGTGCATGTGAGGGCCACAGCCCATGCGTAGGCATAAAAACACGAGAGATAAATAGGTAGATAACTCTTTACTTTCCCCATTGATGGAGACCTTGCTATATGGAGTACAAAGAAAATTTGTGATGTAAAATTCGGCTGGTTAATAGTTAAATAAATAGTACTAGGACAGAGTAGGATGTATAGTTTCATTAGCTCCGGAGGTATCATAAATGCTAGTTGTCAATATATAGTTCAAAGTAAAAAAAAGGAAGATTGTGTCTCGCGTGTTTATTTCCTATGTTAAAATAATGCTAGTTTACCGGCGGTTTCTCTGCAACGTATCTTTTTATCTGTAGTTTCTGTGTCAGCAAGGTAGCACTGTGTTGTTTCTGCATGTCACAAAATTTTATATTTGTCCAAGTTTATCATTTGTATCATCATGCCTTTTTTGCTTTATCTGATTCACTACACTTGGTAGTTAGTACTCCCTTCGTTCCTAAATATTTGTCTTTTTAAAGATTCAACTACGGACTACATAAGGAGCAAGGTAGCACTGTGTCGTTTCTGCATGTCACAAAATTTTATATTGGTCCAAGTTTATCATTTGTATCATCATGCCTTTTTTGCTTTATCTGATTCACTACACTTGGTAGTTAGTACTCCCTCCGTTCCTAAATATTTGTCTTTTTAAAGATTCAACTACGGACTACATATGGAGCAAAATGAGTGAACTTACACTCTAAATTATNNNNNNATACATCCGTATGTAGTTCGTAGTGAAATCTCTAAAAAGACAAATATTTAGGAACGGAGGGAGTAGTGCTGACATAGTGACGTTACATTCTTGAGCTGCATTAATGTTATCCTTTATTTATCCAATCTCCAGCAAAGAGTACCGACTCAACATAGGTCTGTTGCAACTTCTAACTCTACTCTTAAGCAATACACGAGGATCTCTCTCAAATTGTTAAGAATGTCAAAACAATTTGTTATTTCCCTCAACAGGTATAAACAAAGAGACATAGCTTTGAGAGGAATTGTATCCATGAGTAGACTTTCTTGTTTTGGTTTAAGTGAACACTAAGCATCCATGTAGTGTTGTTTGAGATCCTAGCGTCACTGCGCCATGCAGAGCCACCTATTCAATTTGGCTATCTCTTATTTGACATGAGGATAAATATATTGATAAGTCGGCAACGTTACATTCTTGCTGAGTACATGGTATGGGCGACACCTAGAAACAAACCGGACTAGAATGCAAAGAAAATTTAGCAGTCAGATGCGCTGCCAAGTTGGTTTCTCGCTTTACAAAGGTAATATCAAAATCACCAAGCACCATAGTGAGCTCCTGAATTTGCATGAGGACAGGAAGAATCATGGTCCTATTGGTCAACCTGGACTTCCATAAGTTTACCGGAAGAATTTGCATTATGCATTATGATTTTAGGAATTAGGAGTGCTTATTTGGTAACATATAATTCTGGGGTTTTGTGTCCCATAAGGCCGCACTTCTAAAACTCAAACCGAACAATTAGCCGGTCAAATGACCAGTTCACTGATTTGCTGGTCAGAGATCAGTTTAATGGTTGAACCATCGGGCATAAGAATTTCTTTAGCGTATCTTTTAATTAGGAAGCAATTGCTGATCATGTCAAGTAGTAATAGTTGTAACAAGATAATATACAAACTATCTTGCCTCCTAACTAAAAATTAATTCAGCTGTTTTTCTCTTTGCATGATCCACTCCTGTGATCTTAACTGGTTCTGGTACTACTGTGCTACCCTGTCCCTCTCTGTTCTGGCTTTTAAATCTATGTCTTGGACGTTATCTCTTAAAGAGTATCACTCCCTCCGATCCATAACAAGTGTTGTGGTTTACTATGGATCGGAGGGAGTACATACTTAGCTTTTCTTGAATTGTTTCAAAGCAGGCAGAACATTTGATTGCCATAAGTTTGATGTATATCATGTTAACCTTTTCTTGTAGATCTGTTATTTCCTGTTTCTGTAAGATATACTGTTATATTATAGATATGTTTGACTTCTGATTGGTTACTGATGGTTTCTTGCAATGGTGTTCCAATATGGCGGTGCTTCAGTTTATTTAGGTGAGCATTATTTATATAAATCATGACATTATGTCTTTAGATATTCTCAAGTACAAAAAGTGCTAGGCATTAATTGTGCATTTTGCCACCGCCTAGCACCTATCTTGCCTAAGCGCATGCCTAGCATGATTAAACGCTTGGCGATTTGGTGTTCTGTTGTAGCCTAGTGCCAAAGAATCTGTGCTTAGGATCTGATTGTTTTCTCTCCAAATTTTCTACTATGCAGGAAGTTATGATACTGAGCAAAAAGCTGCCAGGGCATATGATGTTGCAGCTCTTAAATACTGGGGACTAAATACAAAGCTGAACTTTTCGGTAAATCTTTCTTGCTTGTTTTTGCTTCCACTGATATAGTTATGGACAAGCGGATCTCATGAGTCAATACTATTCCAGATTTCAGAATATGAAAAGGAACTGGCGGACATACAAGACATGTCTCCAGAGGAATGTGTGACATACTTGCGAAGGTATGTCAAGGGTATATTAATTCAGACTCTTCAGATCGATGTTCCCGTGAGAGGATGAATTTAATAACAAATTCATTTGTCCTGCAGGAGGAGCAGCTGCTTCTCAAGAGGGGCTTCTATTTACAGAGGAGTTACAAGGTGGGCTCTCCTTTTCATTCTGTTCAGCTTGTTCAAAAAAGCTGCCTTTTCCTCTTCAAAACTGCCCAAAACTCACATGTAACCATGTCCCTATGACAGGAGGCAGAAAGATGGTCGATGGCAGGCCCGCATAGGACTGATTGCTGGAACTAGAGACATTTACCTTGGAACTTTCAGTACGATGCACATCCCTTGTTCTGGCCCCTTTCAACTCTCATCAATAGTTCCCATTTTCTTAACTGAAAAACAGTGTATCCCAATTCAATTTCCTGAAGATATGTTTTCGTGTTTGAACTAGAAACCGAGGAAGAAGCTGCAGAAGCATATGATATTGCTGCCATTGAGATACGTGGGAAAAACGCGGTGACCAACTTTGACCGAAGCAACTACATGGACAGGGGCATGCATTGTATAGAAGGGGCAGGGTTGAAGCTGCTTGCAACGAAGCCAGAATGATTTGATATCGTATATTGAACAGATTTGGTAAGCCATATTTTGGGGCTTAGTGGTACATACAAGATAGAAGAACTGGTCGCAGCCTGTCAGTATTTGCTGCTGTATGATTCTTCAGATTATATATAGTTCTTCCAGAGAGAATTTCAGTCATTTAGCAAGCTTTGTGTCCAGGACAAGATTTTGACCATGTATTACTGTTATAGT

>HvAP2-9

>Protein

MKRSPPPQPSPSSSPACSPSPSSPSSSDSSSIAIPRKRARTQKAGSAKAKAAPKRAKKDSGRSTKDSDASANGAAASGKRSSIYRGVTRHRWTGRFEAHLWDKNCFTSIQNKKKGRQVYLGAYDTEEAAARAYDLAALKYWGPETTLNFTVDEYAKERSEMEAVSREEYLAALRRRSSGFSRGVSKYRGVARHHHNGRWEARIGRVLGNKYLYLGTFDTQEEAARAYDLAAIEYRGANAVTNFDISRYLDQPQLLAQLEQGPQVVPALQEELQHDHQSDNAVQELNSGEAQKPGSVSEPIAVDDTDNTGDIGAPLVFDSGVEENLWSPCMDYDVDPIFGPNISSSMNLSEWFNDPAFESNIGYMFEGCSDVDDCSTRHGAGLSALGFLKEGDDKLKDGSDMEAEITPQANDVSCPPKMITVCN

>cDNA

ATGAAGAGATCCCCTCCTCCCCAGCCATCTCCTTCTTCTTCGCCGGCATGCTCGCCCTCGCCGTCTTCTCCTTCCTCGTCCGACTCGTCTTCGATAGCCATTCCCCGCAAGCGAGCGCGGACGCAGAAGGCCGGGAGCGCCAAGGCGAAGGCCGCGCCCAAGCGGGCCAAGAAAGACTCGGGCAGGAGCACCAAGGACTCTGACGCCTCCGCCAATGGCGCCGCCGCGTCTGGGAAGAGAAGCTCCATCTACAGGGGGGTCACAAGGCACAGGTGGACAGGCAGATTTGAGGCACACCTCTGGGACAAGAATTGCTTCACTTCCATCCAGAACAAGAAGAAAGGGAGGCAAGTCTATCTGGGGGCTTATGACACAGAGGAGGCAGCTGCTCGTGCGTATGACCTTGCAGCTCTCAAATATTGGGGGCCTGAAACCACACTGAATTTCACGGTGGATGAGTACGCGAAGGAGAGGTCAGAGATGGAGGCGGTGTCGCGGGAGGAGTACCTCGCCGCGCTCCGCCGCCGGAGCAGCGGCTTCTCCAGGGGGGTCTCCAAGTACAGGGGCGTCGCCAGGCACCACCACAATGGGCGATGGGAGGCGCGAATCGGAAGGGTGCTGGGGAACAAGTACCTGTACCTGGGAACCTTTGATACCCAAGAGGAAGCAGCCAGGGCCTATGATCTTGCTGCCATCGAATACCGGGGCGCAAATGCCGTAACCAATTTTGACATTAGCCGCTACCTGGACCAGCCGCAGTTACTGGCGCAACTGGAGCAGGGGCCACAGGTGGTGCCAGCATTGCAAGAGGAGCTTCAACATGATCACCAAAGTGACAATGCAGTACAAGAGCTCAACTCTGGTGAAGCGCAGAAGCCAGGTAGCGTCAGCGAGCCGATTGCAGTGGATGACACAGACAATACAGGAGACATCGGTGCCCCCCTTGTGTTTGACAGCGGCGTCGAGGAGAACCTGTGGAGCCCTTGCATGGATTATGATGTGGACCCCATCTTTGGACCCAACATCAGCAGCTCGATGAATCTGAGCGAGTGGTTCAACGATCCCGCCTTCGAGAGCAACATCGGGTACATGTTCGAAGGATGTTCGGACGTCGATGACTGCAGCACCAGGCATGGCGCAGGTCTGTCAGCGTTGGGTTTTCTCAAGGAAGGCGACGATAAGCTGAAGGATGGTTCAGACATGGAGGCAGAAATAACTCCTCAGGCAAACGATGTCTCCTGCCCTCCAAAAATGATCACCGTGTGTAATTGAGCATCTCTGTGCTCTCATGTCCAAGGAAGGTTTCAGGTTTTCATGTTGGTTATACCTCTTAGCTAGCTTCTTGTAGGCTGTCTGTCTGTCTGATCAGGTCAGAGAAATTCATGTGTTCATTCCCATCCTTTGAGAAAAAACCCAAAGGCATTCAACTTGGTATGGCAAGAATGCCAATGCTCC

>CDS

ATGAAGAGATCCCCTCCTCCCCAGCCATCTCCTTCTTCTTCGCCGGCATGCTCGCCCTCGCCGTCTTCTCCTTCCTCGTCCGACTCGTCTTCGATAGCCATTCCCCGCAAGCGAGCGCGGACGCAGAAGGCCGGGAGCGCCAAGGCGAAGGCCGCGCCCAAGCGGGCCAAGAAAGACTCGGGCAGGAGCACCAAGGACTCTGACGCCTCCGCCAATGGCGCCGCCGCGTCTGGGAAGAGAAGCTCCATCTACAGGGGGGTCACAAGGCACAGGTGGACAGGCAGATTTGAGGCACACCTCTGGGACAAGAATTGCTTCACTTCCATCCAGAACAAGAAGAAAGGGAGGCAAGTCTATCTGGGGGCTTATGACACAGAGGAGGCAGCTGCTCGTGCGTATGACCTTGCAGCTCTCAAATATTGGGGGCCTGAAACCACACTGAATTTCACGGTGGATGAGTACGCGAAGGAGAGGTCAGAGATGGAGGCGGTGTCGCGGGAGGAGTACCTCGCCGCGCTCCGCCGCCGGAGCAGCGGCTTCTCCAGGGGGGTCTCCAAGTACAGGGGCGTCGCCAGGCACCACCACAATGGGCGATGGGAGGCGCGAATCGGAAGGGTGCTGGGGAACAAGTACCTGTACCTGGGAACCTTTGATACCCAAGAGGAAGCAGCCAGGGCCTATGATCTTGCTGCCATCGAATACCGGGGCGCAAATGCCGTAACCAATTTTGACATTAGCCGCTACCTGGACCAGCCGCAGTTACTGGCGCAACTGGAGCAGGGGCCACAGGTGGTGCCAGCATTGCAAGAGGAGCTTCAACATGATCACCAAAGTGACAATGCAGTACAAGAGCTCAACTCTGGTGAAGCGCAGAAGCCAGGTAGCGTCAGCGAGCCGATTGCAGTGGATGACACAGACAATACAGGAGACATCGGTGCCCCCCTTGTGTTTGACAGCGGCGTCGAGGAGAACCTGTGGAGCCCTTGCATGGATTATGATGTGGACCCCATCTTTGGACCCAACATCAGCAGCTCGATGAATCTGAGCGAGTGGTTCAACGATCCCGCCTTCGAGAGCAACATCGGGTACATGTTCGAAGGATGTTCGGACGTCGATGACTGCAGCACCAGGCATGGCGCAGGTCTGTCAGCGTTGGGTTTTCTCAAGGAAGGCGACGATAAGCTGAAGGATGGTTCAGACATGGAGGCAGAAATAACTCCTCAGGCAAACGATGTCTCCTGCCCTCCAAAAATGATCACCGTGTGTAATTGA

>DNA

TTATGAGCACGCCTTACCCGTTCGCTATGCAGTGGAGCTTCTGAAGACATGCGTTGAATATGCAAAACCATCTCACATGATGTTGAACAAGCTTCTCTTAAATCGGTGCTATCCCAACTTTATCTCTTATATCATCATTTCCAACTCCGTCCTTCCTTGTGCGGTCACACATCCATCTCAACATGCGCATCTCTGCCACACCTAACTCTTGAACATGTAGCCTTTTAGTTGGCGAACACTTAGCGACATACAACATTGCGGGTCGAACCGCCATCTTATGGAATTTGCATTTTAGCTTTTTTGTCACTTTCTTGTCATAGAGAATGCAATAAGGTTGGCGTCAACCCATCCATCTGTCTTTGATACGATGGTTCACATCTTCATCCATATCTCCATCCCTCTACAAGATTGACCCCAAGTATTAAAAGGTGTGCTTTTGAGGCACCACATGCCCATCAAGGCTAACCTTCTCCTCATCGTGCCTAGTGGTACTGAAACTGCACCTTGTTGTCTCGGTTTTAGTTCTGGTAAGTCTAAAACCTTTTTGCTTTCAAGATTTGTCTCCATCGTCCTAACTTCCTATTGACCCTCGTCCAACTACCATCGACTAGCACCACATCATATGCGAAGAGCATACACCATGGGATATCTCCTTGTATATCCCTTGTGAACTCATCGGTCACCAAAGCAAAAAAGATAAGGGCTCAAAGCTGACCTTTGTGTAGTTCTATTTTAATCACGAAGTCATCAGTCGCCATCACTTCTTAGAACACTTTTTCAAAATTTATCGTACATGTCCTTGATGAGGGTAATACTTTGTTAGGACTTTGTATTTCTCCAAGGCCCACCATATGACATTCCGTGATATCTTATCATAGGCCTTCTCTAAGTCAATGAACACCCTATGCAGGTCCATATTTTGCTCCATGCATCTCTCCCTAAGTTGTCGTATCAAGAAAATGGCTTCCATGGTCAACCTCTCACGCATGAAACCAATATGATTTTGGTCATGCTTGCCATTCTTCTTAAGCGGTTCTCAATGACTCTCTCACATAGCTTCATTGTAGGGCTCATCAGCCTAATTCCACGGTAATTAGTTCAACTCTGGACACCCACTTGTTCTCGAAGATTACTACTAATATACTCCGTCTCCATTCTTCTGCCATCTTGTTTGCCCAAAAATAAGGTTGAAGTGCTTGGTTAGCCATACTATCTCTATGTCCCTGAGTCCTCTCCACGCCTCGATTGGGATACAGTAAGGGCATGTCGCCTTGCCTCCTTTTATCCTTTTCAAAGCATCCTTGACCTCAAACTCCTGGATTTTTCGCACAAAACGCTTGCTAGTATCATCAATTGAGTCGTATAGTTCAATGATAGATATTTCATTCTCCTCATTGAACAACTTGTCAAAGTACTCCTGCCATATATGCATAATTTCCTCGTCCTTCATGAGGAGATGGTCTGCTCCGTCCTTCATCCATTTGACTTGGTCCACATCCCTTGTATTCCTCTCATGGATCTTGGCCATTTTATAGATGTACCTTTTGCCTTACGTCATGTATAACCATTAGTAGAGGTCCTCATACACCTGACCCCTTGCTTCAATCACAACTCGCTTTGCGGCCTTCTTTGCCATATTTTACTTCTATATGTTGTCTGCACTCCTACCCAGGTATAGGCTTATACTTGTGTATGATCCGAAGTATGTCTAAAGGGGTGTGTGATTAGACTACTTGACCAAATAAAAACTTAGTCTTTTCCCAATTATAGTTGTGGGCAAGTTTTAGCAGTTGTTGTAAGTCAAGCACACCCTAGACATGAAATTCTAAGAGTATAGCGGCGAAAGTAATGACATGCACATGTAAGTAGATGTTAGGGATAGGAAGATCAAACGCAAAGGTTGACACAGCGATTTTTTGCGTGGTGCCGATAGGTGGTGCTATCATACGTCCACGTTGATGGAGACTTCAGCCCATGGAGGGTAACGGCCGCGCGAGTCCATGAAGAGATCCCCTCCTCCCCAGCCATCTCCTTCTTCTTCGCCGGCATGCTCGCCCTCGCCGTCTTCTCCTTCCTCGTCCGACTCGTCTTCGATAGCCATTCCCCGCAAGCGAGCGCGGACGCAGAAGGCCGGGAGCGCCAAGGCGAAGGCCGCGCCCAAGCGGGCCAAGAAAGACTCGGGCAGGAGCACCAAGGACTCTGACGCCTCCGCCAATGGCGCCGCCGCGTCTGGGAAGAGAAGCTCCATCTACAGGGGGGTCACAAGGCACAGGTGGACAGGCAGATTTGAGGCACACCTCTGGGACAAGAATTGCTTCACTTCCATCCAGAACAAGAAGAAAGGGAGGCAAGTCTATCTGGGGGCTTATGACACAGAGGAGGCAGCTGCTCGTGCGTATGACCTTGCAGCTCTCAAATATTGGGGGCCTGAAACCACACTGAATTTCACGGTACGTTTTGCTGATCTTGGAAATAGAAGAAAAAAAGGAATCATTGATATGAATGTGTACCCAAGTTGCTGCGTCAGTTCCTGTTTGGTACAAGCAGGAGCCAGGGTGTGTCTTGGCAAGCACCGCCATTGTCCATTGACTCGAGAGTTATGGAGGTGACAGCAGAGCAAAAGAGATGAGTCACTGAGTAGTAGTAACGCCTTGACAGTCCATGTTTTAGTGTACAACTTAGATGAGCAAAGCACATAAAAATACAGCCTCTAATGTGCTGATTGTGTAACAACTGTTTGTTTGTCAACAAAGTAAACATAGTTACTTCCTCCATAAACTAGTATAAAAGCGTTTAAATCACTATAAGCACTCTACTATGCAAAAATAACGTTCCTCAAGCATTGTTCATGAACGCAGGTGGATGAGTACGCGAAGGAGAGGTCAGAGATGGAGGCGGTGTCGCGGGAGGAGTACCTCGCCGCGCTCCGCCGCCGGAGCAGCGGCTTCTCCAGGGGGGTCTCCAAGTACAGGGGCGTCGCCAGGTAGGAGTACTATATACACTACTAATACCGCATTAATCAGCAGCTCCTGTTTTGGTTAGCCACAAAACTAGTGCTACTATTCCGAGCCATGCAAAATTCATGAAGAAAGGAGACAGGTGGCATCCGAGTGCCAAGTGTACACTCCGTGGGATCCAGAGATGGTGCTGACTCGGGAGGAGAGACCTTTCTGGTGGACTGACAAAATAGGGGTACGTAGGCAGCTAGGATCCATCTGCACCGTGGAGTCAGTGGCACTGCATGCGTCAGCTGGCAGGCACAAAGTGCAACGTCATACCCCGGCCAGCCATGTCATCTAGAGTGGCCACAGATGGCTCACCTACCTACCTCTTTCTCTCGTCAGCTGTGTGCCCAACAAAGTCCAACCTGGCCGACAGCAGGCGTCCGGTTTGGAATTTCGACTCAAATTCACAAAAAACAGTTCAACGAATGTCGTTGAACTTTTTCTTGCGACTGATTTTTGACAAAACTATACACAGGGATTTACTGAAATTTCAGAGAGAAACACTCAGGATTTGTGTCTGAATATACGTAAATGTTTGTAATTTTGGGTGCAGGCACCACCACAATGGGCGATGGGAGGCGCGAATCGGAAGGGTGCTGGGGAACAAGTACCTGTACCTGGGAACCTTTGGTGAGCAGCCTCCTCCTCCCCCTCCTCCTTTGAGCAAGAAAACAGTTTTAATTAATCGGCATTGAAAGGCTACCATTGTTCGTTGTAGAACCGTCCTGCTTACTAACATTCCGCAAGGGAAGAGCAGCTGCCCACCTAACTTTTACCAGAATTTTATGATCAAACCTAACACCCACATATATTCAATGGCATGCACTTGTTGTTTCCCTTCAATGCACCACACACGGCCTCACCTTAATGCTGCTGCTACCATAATCTCTACTACTACTCCTCCTATGTTAATTACTCCCTCCGTCCCATAATATAAGAACGTTTTTGACACTCTTATATTATGGGACAGAGGGAGTAGTTGTTATTCTCGCTTTAGTTCTACTTTTGTTTTTCATTTTTCTACTTGCATAGTATATCTGAAAAAGAAAGAAAGAAAGAAAGAAAAGGGCTGGCAGGGTCAAGATTCAGGACCATGAGACAGACCAAGATACCATCCTGTGTGCGATCCCAAGAGGATTGCTCAACTTGCCCTAAAAATAACATTAAGCGCACAGACCAAGTGTTGGATCAGATCATACGCAATGCCCTACACTGCTAATTACCAGTAAATACGGCAGGCATGTGAGCGGCACGTGGTGGGAGTGGCAATGCCACCGGCTGGTGCATAGGGATCTTCAATCTCTCCCTGTCTTCTTTTCACTTGCCTCCCTGACTCAGCAGTGAGTGGTCGCCTCGCCCTCACGTGCATGGGGCATGCCTTCCTCTCTGTCAAAAACCAACCAGATCAAGATTCAGGATCCAAACCACCGCACAAATGATATGATTCACCACACACCGCCTGCTAGTAAAGGGCAGATTGAGATAAAAAGGAAAAAAAAATCCCCAAAGGGCGAGAAGATCGCCACTAAGGGCAGGTTGCCACAAGAGCTCTCTAATTAATTACTGCAGTAGCAAAGCACCACAATCAACTCCAGTGACCAGCACATGTGAAAGCAGCAAGCACCCCATCCACTGTCGTCTTTCAGGCAACTAGGATGTGAAATTCAGCATCTTCTGCTCTTAGATACTTGACGCTTACGCTCATTCACTGATATCACCAAACAACTCTGGTAATTCTTTGATTTAGCTCACCATAGTGCATCTACTTTCTTAATGCAGATACCCAAGAGGAAGCAGCCAGGGCCTATGATCTTGCTGCCATCGAATACCGGGGCGCAAATGCCGTAACCAATTTTGACATTAGCCGCTACCTGGACCAGCCGCAGTTACTGGCGCAACTGGAGCAGGGGCCACAGGTGGTGCCAGCATTGCAAGAGGAGCTTCAACATGATCACCAAAGTGACAATGCAGTACAAGAGCTCAACTCTGGTGAAGCGCAGAAGCCAGGTAGCGTCAGCGAGCCGATTGCAGTGGATGACACAGACAATACAGGAGACATCGGTGCCCCCCTTGTGTTTGACAGCGGCGTCGAGGAGAACCTGTGGAGCCCTTGCATGGATTATGATGTGGACCCCATCTTTGGACCCAACATCAGCAGCTCGATGAATCTGAGCGAGTGGTTCAACGATCCCGCCTTCGAGAGCAACATCGGGTACATGTTCGAAGGATGTTCGGACGTCGATGACTGCAGCACCAGGCATGGCGCAGGTCTGTCAGCGTTGGGTTTTCTCAAGGAAGGCGACGATAAGCTGAAGGATGTTTCAGACATGGAGGCAGAAATAACTCCTCAGGCAAACGATGTCTCCTGCCCTCCAAAAATGATCACCGTGTGTAATTGAGCATCTCTGTGCTCTCATGTCCAAGGAAGGTTTCAGGTTTTCATGTTGGTTATACCTCTTAGCTAGCTTCTTGTAGGCTGTCTGTCTGTCTGATCAGGTCAGAGAAATTCATGTGTTCATTCCCATCCTTTGAGAAAAAACCCAAAGGCATTCAACTTGGTATGGCAAGAATGCCAATGCTCC

>HvAP2-10

>Protein

MASPNPAAADAGLQNKLEAAAGGSEGPPPAYGAAVGVAAAEQAPPRRLAPARKERVCTAKDRISRMTPCAAGKRSSIYRGVTRHRWTGRYEAHLWDKSTWNQNQNKKGKQVYLGAYDDEEAAARAYDLAALKYWGAGTQINFPVSDYTRDLEEMQMISKEDYLVSLRRKSSAFSRGLPKYRGLPRQLHNSRWDASLGQLLGNDYMNLSCGKGIALDGKFAGSFGLERKIDLTNYIRWWVPKKARQSDTSKAEEVADEIRAIEGSVQLTEPYKLPSLGLGSHSKPSSAGLSACGILSQSGAFKSFLEKSTKLSEECTFSKEIDEGKVAVSEPTTGHHTSAVDINMNGLLVQRAPYTLAPVMPTMKSTWSPSDPSADHLFWSNFILPSSQPVTMATITTTTFAKNEVSSSDPFKNQEYEHTK

>cDNA

CACGCGTCCGCGGTCACCACTGCGACTCCTCCAAAGGCCGAAGCGCTCGCCCCCGTCCTCCTGCCTCGTCGCGCCGGCTCGACCGATCCGGTGGCGGCGCCATGGCCTCCCCCAACCCCGCCGCGGCCGACGCGGGGCTGCAGAACAAGCTGGAGGCCGCGGCGGGGGGAAGCGAGGGGCCGCCGCCCGCGTACGGCGCGGCGGTGGGGGTAGCCGCCGCGGAGCAGGCGCCGCCCAGGAGGCTCGCCCCGGCGCGGAAGGAGCGCGTCTGCACCGCCAAGGACCGCATCAGCCGCATGACGCCCTGCGCCGCCGGGAAACGCAGCTCCATCTACCGCGGGGTCACCCGGCATAGATGGACAGGGCGATATGAGGCTCACCTTTGGGACAAAAGCACATGGAATCAGAATCAGAATAAAAAAGGGAAACAAGTATATTTAGGCGCATATGATGATGAAGAGGCTGCGGCAAGAGCCTATGATCTTGCTGCATTGAAATACTGGGGAGCTGGAACACAGATAAATTTTCCTGTCTCCGATTATACAAGAGATCTTGAAGAGATGCAGATGATCTCCAAAGAGGACTATCTTGTATCTCTCCGAAGAAAGAGCAGTGCCTTCTCTAGGGGATTACCAAAATATCGTGGGCTTCCCAGGCAGCTCCATAATTCCAGATGGGATGCTTCTTTGGGACAACTGCTTGGCAATGACTACATGAACCTCAGTTGTGGAAAGGGCATTGCACTGGATGGAAAATTTGCGGGAAGCTTTGGGTTAGAGAGGAAAATTGATCTAACAAATTACATTAGGTGGTGGGTACCCAAAAAGGCACGGCAGTCAGATACATCAAAAGCAGAAGAGGTTGCTGATGAAATCCGTGCTATTGAAGGTTCAGTGCAACTGACTGAGCCGTATAAGCTGCCTTCTCTTGGCCTCGGTTCCCATTCAAAACCCTCTTCAGCGGGGCTATCTGCATGCGGTATCTTATCTCAGTCTGGTGCCTTCAAAAGCTTCTTGGAGAAGTCCACAAAATTATCTGAAGAATGCACATTTAGCAAAGAAATAGATGAAGGAAAGGTCGCTGTATCAGAACCTACTACTGGACATCATACATCTGCAGTTGACATTAACATGAATGGGTTGCTAGTACAAAGAGCTCCATACACATTGGCCCCTGTTATGCCTACAATGAAAAGTACCTGGAGTCCTTCTGATCCTTCTGCGGATCATCTATTTTGGAGCAACTTCATCTTGCCATCGAGTCAACCTGTCACAATGGCGACAATAACGACAACAACGTTTGCAAAGAATGAGGTGAGTTCAAGTGATCCATTCAAGAACCAGGAGTATGAACATACCAAATAGCAAGGATATTTTAGCTGGTTGATGATGACAGCGTCGTTCACCTCCAGTGTGTTTGTGCTGCCAATCTTTTGATGTGGTGGCTTGTGAGCAATACTCTTTGTATCCATACTTCACAGAACATGCATAGAAGGTAAGAAAACCATAGTTATGTAATATACAGTGGCTAATGCCCAGGAAACAATGTGTAGCTGTAAAGCCGTCTGTATATATTGCTGTTGTCTACAGATTTGAGGAAGAGAACAGAAATAGGAATGTGATTTTTGT

>CDS

ATGGCCTCCCCCAACCCCGCCGCGGCCGACGCGGGGCTGCAGAACAAGCTGGAGGCCGCGGCGGGGGGAAGCGAGGGGCCGCCGCCCGCGTACGGCGCGGCGGTGGGGGTAGCCGCCGCGGAGCAGGCGCCGCCCAGGAGGCTCGCCCCGGCGCGGAAGGAGCGCGTCTGCACCGCCAAGGACCGCATCAGCCGCATGACGCCCTGCGCCGCCGGGAAACGCAGCTCCATCTACCGCGGGGTCACCCGGCATAGATGGACAGGGCGATATGAGGCTCACCTTTGGGACAAAAGCACATGGAATCAGAATCAGAATAAAAAAGGGAAACAAGTATATTTAGGCGCATATGATGATGAAGAGGCTGCGGCAAGAGCCTATGATCTTGCTGCATTGAAATACTGGGGAGCTGGAACACAGATAAATTTTCCTGTCTCCGATTATACAAGAGATCTTGAAGAGATGCAGATGATCTCCAAAGAGGACTATCTTGTATCTCTCCGAAGAAAGAGCAGTGCCTTCTCTAGGGGATTACCAAAATATCGTGGGCTTCCCAGGCAGCTCCATAATTCCAGATGGGATGCTTCTTTGGGACAACTGCTTGGCAATGACTACATGAACCTCAGTTGTGGAAAGGGCATTGCACTGGATGGAAAATTTGCGGGAAGCTTTGGGTTAGAGAGGAAAATTGATCTAACAAATTACATTAGGTGGTGGGTACCCAAAAAGGCACGGCAGTCAGATACATCAAAAGCAGAAGAGGTTGCTGATGAAATCCGTGCTATTGAAGGTTCAGTGCAACTGACTGAGCCGTATAAGCTGCCTTCTCTTGGCCTCGGTTCCCATTCAAAACCCTCTTCAGCGGGGCTATCTGCATGCGGTATCTTATCTCAGTCTGGTGCCTTCAAAAGCTTCTTGGAGAAGTCCACAAAATTATCTGAAGAATGCACATTTAGCAAAGAAATAGATGAAGGAAAGGTCGCTGTATCAGAACCTACTACTGGACATCATACATCTGCAGTTGACATTAACATGAATGGGTTGCTAGTACAAAGAGCTCCATACACATTGGCCCCTGTTATGCCTACAATGAAAAGTACCTGGAGTCCTTCTGATCCTTCTGCGGATCATCTATTTTGGAGCAACTTCATCTTGCCATCGAGTCAACCTGTCACAATGGCGACAATAACGACAACAACGTTTGCAAAGAATGAGGTGAGTTCAAGTGATCCATTCAAGAACCAGGAGTATGAACATACCAAATAG

>DNA

AAAACTCCAAAAACTAGGAAAAACCAGGCGCAAACCGAAACGCCGGACAAAAATGAAACCCCCAAAATGTTTATTGGAAATAAAATATAAAATTCAGAAAAAGCGCCCAACACGTGACACATAATGGCGGCTGGACGTACCATTTGACGCGCTGTGAGACGATAAAAATGACCCTTGCGGGGCTCTCACAAGAGGTAACCCCTTGACTAGCTACTCTAGGATTTTTCAAGAGTGCATACTCCTTTTTTATGGTGGGGAATGCATACTCCTGCTTTGCGAGGGAGGGCATCCCACTTTGTTGTTGCACGCTCGGCCCAGCATACAAAGGCTCATTGCTCCAGCCTGCTTGCCATCTGCTACGCCTAATAGTGAACCTCTCCTTGTCTTATAGGCGCCATCCACTCAAAATGTTTCGACCAAGGAGGTATGTATCAATGATGTAACTCATGCACTCCTACATATAATTAATGGGACTAAGTCAGCAGAAAGCATAAATGAGACCATCTTGGTATTAATACCAAAGGTAAAGAACCCTATGATGCTCCCTTAGTTCAGGACTATCTCTTTATGTAATGTGCTATACAAAATTGCCTTCAATGTCATATCAGACAGGTTAAAGGTGGTCCTTCCAGAGATTATCTCACACGAGGAGTCTGTCTTTGTGCTTAGTAGGTTGATTGCGGATAACATTATCGCAACCTATGAGTGCCTACATTTTATGAAAAGGAACAAGGCCAAGAGATATCACGACTATGCTTTGAAGCTTGACATGATGAAAGAATATGATAGGGTGGAGTGGGACTATTTACAAGCAATAATGTTAAAATTGGCCTTTACAGAGACATGGGTAAACATCGTCATGGGCCTGGTCAGGACAGTTAAATTCGAAGTTCAGTTCAACGGTAGGAAAGTTTAGGAGTTTAGTCCTTCACATGGGGATTCAACACGGGGACCCAATCTCCCCATACCTCTTGCTAATTGCAGCAGAGCGCCTCTTGTACCTCCTGAAATCTATAGATGAGTCATCCAGCTTGAGCGGGTTGTGTTGGAAATATGACCGATTTACTATGTGATTTTACTAGCAGCAATAGTAGATAAATCATGACTATCATAGCAATTATAAGACAAGTCATGAGATCATACAAATAGTATGCAAGTAGTATCTGAAGTTGTGAGTAGGAACAGAACATATCTAGAATAGGAACTCGAACAAGAATTTGCGGCAGAAAGTCAAACAAGAAGAACACCAACACGTGCTGAGTTGCAGCAACAACAGTAGTGGGATTGGCGTTGATTTCGTCGCCAACCATGTTGCCGAAGAGGTTGTTGATGTCGAGGAAGAAGTCATCGACCGGGAAGTGTTCGTCGGCGTCCATGGTGTCTGTGATGAAGAAGCTAGTAGTCGCGTAGAACACTCCCGAAAATCTTATCACCCTTCTCCTGTATAGGACTCAAAGAGGCGGGGTTTCGTAGGCCTACTATCCCAACCTGCGGTGCATGCTGCATGCCGGGATGGGGAAGAACCTAGCAGCAATTCACAGATTGGGACTCGCTGGCGTGACGAAGATGATGTTCTCGTCTGTTTCTCCGGAGAGGAGCGACCTCTCTTTTATAGGCACAAGAAAGGGAGGCGAACAGGCAGCAAGGGAGGCGAAGCAAAAGAGGGAGACAAAACGAATTGGCAGCGGTCGAGGGGTGCATCGTTCGTATTTAATCTCCACTACAGCAAAACTTTTCAGCTCCCGAGCGGCCTTTGTATACCAGTCGCGCATGGAAAAAAATTAGTCATTAGCCCGGCTCATTCGCCCAACCCGCGACGCGTTGTGGCAAAGCGAGGCGGGCGACGAAGAAGGAGCAGCCCACATGTATGTCTCTCTTGTTCTCATGATCATACATGTGCACGCGTCCGCGGTCACCACTGCGACTCCTCCAAAGGCCGAAGCGCTCGCCCCCGTCCTCCTGCCTCGTCGCGCCGGCTCGACCGATCCGGTGGCGGCGCCATGGCCTCCCCCAACCCCGCCGCGGCCGACGCGGGGCTGCAGAACAAGCTGGAGGCCGCGGCGGGGGGAAGCGAGGGGCCGCCGCCCGCGTACGGCGCGGCGGTGGGGGTAGCCGCCGCGGAGCAGGCGCCGCCCAGGAGGCTCGCCCCGGCGCGGAAGGAGCGCGTCTGCACCGCCAAGGACCGCATCAGCCGCATGACGCCCTGCGCCGCCGGGAAACGCAGCTCCATCTACCGCGGGGTCACCCGGTACGTACCGCCCCTGCCTGGCCCGCTCACGGACTCATCTCCGCTGCTACCTGCTTTTCGTTTCCCGGCGACCATGTCTGGAAGAAGCTCAGCACTACAGATCGCCAATGAGCTTCCGTATGCGTCTCTGTCTCGAGTTATTGGGGGTGGGGAGCGTATCGGAAAGTGACGCAGAGCGTGACTAGCCTGGGAGATCCGATGAGAACTGGCACACGACGAGAATAAGTGAATGTTATAGAGGACTATTGCTGTTAGGTATCTCAACATTGCAGCCAGTGTTTGTCTCTGGTTATTATCACGAAACAGCTGTAAATTAATGTATGAACATCACAGTTAGTACCCTGTTCAAACGATGATCTGCAGGTCAGCACATGCTAATGGTTAATGTGCACGCTCAACCACGAGAAATTACAAGTAGTATTACCTCCGATGTCTGGCCACCTTAATTGTTTACAAATACTTCCAAGTTTGACCTCTTCACCGTGCTTGGCACTTCATGCTTCTCGGTGCGCATTTCATATTTGGTGTCCTCCTTCGCTCCTTCCTTCTCCACTTATGAGTATTATTTTCTGTACTATACGCTATTATTTCTTTTCTTTTTTACTGCCTTCAACAGACATATACTTCCTTAAACTTTTTGTGAGCATAGCATTATCTCCTTTTGGTGTTCCTTATACATGTATTCATTCAATTTATTACTTAGCCCAATGTTGCATCAATAGGCATAGATGGACAGGGCGATATGAGGCTCACCTTTGGGACAAAAGCACATGGAATCAGAATCAGAATAAAAAAGGGAAACAAGGTATGCGACTATTGTGTGCTCAGTACAGAGTTACAGCTGTTGTTAATTTATCATGCTTCTTTAGCAATCTTATTACATTGGTGTATAGGAAACTTGTCCATCTTAACTTCCTTTTATTGCAGAGGTCATTTGGGATTATTGTGTGCTCAGTACAGAGTTACAGCTGTTGTAAATTTATCATGCTTCTTTAGCAATCTTATTACATTGGTGTATAGGAAACTTGTCCATCTTAACTTCCTTTTATTGCAGAGGTCATTTGGGATTATTGTGTGCTCAGTGCAGAGTTACAGCTGTTGTAAATCTAGCATGCTTCTTTAGCAATCTATTACATTGCTGTATAGGAAATTTGTCCATCTTAGCTTCCTTTTATTGCAGAGGCCATTTGGGATATTGTGTGCTAAGTGCAGAGTTACAGCTGTTGTAAATTTATCATGCTTCTTTAGCAATCTTATTACACTGGTGTATAGGAAACTTGTCCATCTTAGCTTCCTTTTATTGCAGAGTCATTTGGTTTAAGGTCTCTAATCCTCAATCATCCATCATCCGATATGGAATCTATGTAGTAATTAAGTAGTTCTTCCGTCTACAAGTTTACACTCAAACAGATCTTTTTCTTTTTTACTATTGCTTGAGTCAGGTATTTTTGACTAGATAATTGTTTGCTGTGCCATAACCACTGCATGCATTTACTGGTTGTAAAGAGTATGCATTCTGCATATCTCAGTTTTTATACTATGTGCATGATTTCTTTCATATCTCCATACGATGACCTGTGTTAAAGTTAACTATATCAGTATGATTTACCATTTAATTTTTATTACTGTAGTTGTGCTTAATGTAATTTAATGCACATAGTGTTAACTTGATAAGATGTTTCTTTTTTACATGGGAAACTGCACGGCGCACCAATCTTGCAGTATATTTAGGTAAGGAGCACCACCGATGCTGACATTAAAATGCACATAGTGTTAACTTGATAAGATGTTTCTAATGCCCACCATTTGATGTGCAACATAGGCGCATATGATGATGAAGAGGCTGCGGCAAGAGCCTATGATCTTGCTGCATTGAAATACTGGGGAGCTGGAACACAGATAAATTTTCCTGTGAGTCGTCTATAAGATTTGCTAATGCCATTCAGCTCTTGTTTTCTAGTCTTTGTTAAATTCTTCCACCTTAAATTATTAAGTATATTTTCCTTTTTTAGTAAGATCGTGGTTTAGCTGAATGCATTATTCATTTATTTCAGGAAGTGTTCCATGGTGAAACTGATACCTCTGTGTGCTAATCAGTCTGTTTGTTATTAATATTGAGCAATGATTTATTTAGAACCCACGATGTAATAAAAATCTTGCACAGAAACCGAAAGCTTCCAAATTTGATCTCTAAATTTCAGAAATATCTAGTGTCTTTTGTCTACTTTACATTATGTATATCAAAATTTGAGATACATGTTACCTCAGTTCAGTTTAAGCCGATGATTCTCTTATCTCGTTTGGCTGGTTTACATATTTACAGATATAAGTGTTGGGACGAAACCCAAAACTGTTTACTATGTAGTGCCTACTTTTCATAAAGCACAATTAAAATTAGGATATATGATGCCCCAGCTCAGCTTCATTATAAGTCTATGATTCTCTCATCTGGTTTTGTTTTTATTATTATTTTAATTTACAATTGTAACTTAAAACAAATAACACAACTGTCTTCTGCAGAGTATTGACGAAACAAAAATTATAATTACTTCATTGTTTTCAATTCCATGTCAACTGTCACAATACCTAATGAAAAATTTGACAATTAGCAAACTTATGTTCATTATGTATCTGCCTATTTAGGTCTCCGATTATACAAGAGATCTTGAAGAGATGCAGATGATCTCCAAAGAGGACTATCTTGTATCTCTCCGAAGGTATATTTGTGTCGTGCATTTATAATGCATATACATTATTTATTTTCTGAAGCAATGACTGGAAGATGCATGCATTGCAGAAAGAGCAGTGCCTTCTCTAGGGGATTACCAAAATATCGTGGGCTTCCCAGGTGCGTTTTAATGATTGAGAAAATATATCCTCTACTATGCAAATTTCTTTTATGTTTGTTGTTTTGTGATAGTTTACTTCTGTAATGCATCCCTGTAAAAGTTTTTCCACATGCAAATCATTTATTCCCAAATTGTTCAAACTGTCGATGCTTTGTTTAATATGATTGTATTGTGCTTTATTGCTACAAATATTTCTGCCACATCAATAATGGGTTGGAAATTTCGCTACAATCTCATATATTAGTTTGTAACTTTCTCTGTTGCTTTGTGTTCATCTTCACAACAGAGCAACACTGCAAAACTAAAATGAGCAAATAAAGTATTCAATAGCTTCTGTAGCCAATTGTGGTTTTGCACCATGACAGCTTAGCAGTTTCCTTAAAAATTAGATTGAAAAAGATTATAGTTCTATACTGGTAGCTTGGGAAATGAACATATCTGCAGCTTGCACTAAACATATATTTCTTCCAAACCCAATCCCTCAATCATACAGTATGTATGCTTCTTGGGCGTCCTATTATACAACAAGTGTTTTTCACTCTGTTCCATGAGTTGGATCTGATCATTGACAGTGTGGGGTCTATCCATATATAGGTAGACATTCGTATTTGGGAAGTGGTATTACAATATTAATTTGTGACAGGTGATGTAATACTATGAGTAGCTTTATTTAGTTCCATTTGTCTCAAATATTGACCAGACATCAGCATTGAAGACTAACCTTTTCAGCTTGCCCTGTTTGATAACAAAATATTTTTGAAGTAAGGTTTTTAAAATGCTGCAGTTTTAATTACTATGAGCTGTTTGGCTATGAGTGAAAATTGTGATAATAATACTGCAGTATTTACAAAACCACAGTATTTTCTCTGTTTTAAAAAAAGAACCCTCGACCTCTTTTTTAAGAACAGAGCACACGAAAGAAATGCCGTCCTCGTTATCTTCTTCCTGCTTCTAAGCTGCAACCACCATGTTGTTTCTGGATATCGTCTCCATCGGGGACAAACCAAGAGATGGCAAAGGAGAGAGGAGCGTGACTATGCTGACCTTTGACCTCCCGCCTCTCGGCGCTGGCCGCTGGCACCTGCCTCTGCGCGGCTGCTTTGTTCCCCTTCTTGCGTTGAGTTGAACTGCCTTCTTTTCTTGTTTGTGCATGGTAGTTCAGTGCATCCGGCTGATTATTATGCATGTCCGTTCAATGCGTCCGGCTAGCTAGCTAGCACCGCACACAGTGCAACTGCTTCATATGCTAGCTAGCTAATTTACGTTGGTGTATACAAGCACTTGCCAAACGGGTCGCAGTATTTTCAAAACTTCAAGATACTTTGGTATGTCAAAGCTGCGGTATCTTGTACCTACTGACTAAAATACTATGGTTTTCAATACTGCGGTTTTTCTAATACTTTGCTGCCAAACATAGCATGTACAAGGCATACACAGTAAAAGTTTTGTAAGATGTGTGCCATGTCAATGTACAGTTTGTAATTATAAATGTTGCAACAAGAATTGTTCAAATTTTCAATTAAGTTTCATGACATAAATGCGTTACTAAAAAGCTGCAAGTGCTTTACTTAAGCTTTATACTAAGTGTACATTGTCATACAGGCAGCTCCATAATTCCAGATGGGATGCTTCTTTGGGACAACTGCTTGGCAATGACTACATGAACCTCAGTTGTGGGCGTCTATGCATACTACTACACTGCTCTTTTGAGTTAAACAAACTAAAGAAGTGGTAACTAGGCTGTGATACCTTGAAAGTTGCAACTTATTTATATTACAAAAGGGCATTGCACTGGATGGAAAATTTGCGGGAAGCTTTGGGTTAGAGAGGAAAATTGATCTAACAAATTACATTAGGTGGTGGGTACCCAAAAAGGCACGGCAGTCAGATACATCAAAAGCAGAAGAGGTTGCTGATGAAATCCGTGCTATTGAAGGTTCAGTGCAACTGACTGAGCCGTATAAGCTGCCTTCTCTTGGCCTCGGTTCCCATTCAAAACCCTCTTCAGCGGGGCTATCTGCATGCGGTATCTTATCTCAGTCTGGTGCCTTCAAAAGCTTCTTGGAGAAGTCCACAAAATTATCTGAAGAATGCACATTTAGCAAAGAAATAGATGAAGGAAAGGTCGCTGTATCAGAACCTACTACTGGACATCATACATCTGCAGTTGACATTAACATGAATGGGTTGCTAGTACAAAGAGCTCCATACACATTGGCCCCTGTTATGCCTACAATGAAAAGTACCTGGAGTCCTTCTGATCCTTCTGCGGATCATCTATTTTGGAGCAACTTCATCTTGCCATCGAGTCAACCTGTCACAATGGCGACAATAACGACAACAACGGTTTGTTTCTCTGCCTGACATATTTTCTACTTCTGGGTTCTGAAATGCTGAATCTGACCATTGATTTGTGACAGTTTGCAAAGAATGAGGTGAGTTCAAGTGATCCATTCAAGAACCAGGAGTATGAACATACCAAATAGCAAGGATATTTTAGCTGGTAATTAAGTAGATCAGATCCTGCATTCTATTCCTGTCACTAAATTTGGTATTTCAGTTTTCTAATTTCTGCGTGTCACCTAAAATGGCTATAAATTTTTTCACATGGGAAGCTTAAGGAATCCTGGGAGTAATCAAAGTTACGATTTTAGAAATGTGTTGTTAACTCAATCAAATCTAAGAATTAGTCACCCAAGAATAAATATACAGACACTAGTTTTTTCCTTAAAAAATGGTGCAAATTTTTAAAGACAAGAGAATATACCTGACAACAAGACACTGCATAGATAGATGGACATATGGAAATGAGTTTCAACTAATTTATGAACTAAAGACAAGATTTATATTTGCTTGTGTTGCATTCCAGTAGGGATCAACACATGGAATGATTGGCACCTGCATATAACTTGTGGATCAACACATGGAATGATTGGCACCTGCATATAACTTGTGTATGGAGTGGTGGGCATGATAAATTAAAACTTCTCATGTGCGTGTGCGTGTGTGTGTGCGCGTTTGGCGTGTGCTCATGCGCGCGCGTGTGTGTTTTACTTTGTGCACAAACCTTCCATTATTGAAAATGTGTGTCCGTCTGTGTTACTCATGGAAACTGGTAGGAGTTTGATTTTCTGTATTAATGGATTCACCAGTGGAGTATCCATTTTGGGTCCCGTTACCGGTAGTAGTTTTATGCTAGTGTAGTTATATGCTCAAGATTGTCTAAAATTTCTACTTGCTTGCTGCACCAGCTAAATTAGTCGTAAAAACAGCCAGTTAAAGTGTCCTAGTTTTTGCTAAGTTGAAGTAGCCCTTTAATTGGTTGCTGAAAGCTAAGCACATTCCAACTGTTCAGCAGGTTGATGATGACAGCGTCGTTCACCTCCAGTGTGTTTGTGCTGCCAATCTTTTGATGTGGTGGCTTGTGAGCAATACTCTTTGTATCCATACTTCACAGAACATGCATAGAAGGTAAGAAAACCATAGTTATGTAATATACAGTGGCTAATGCCCAGGAAACAATGTGTAGCTGTAAAGCCGTCTGTATATATTGCTGTTGTCTACAGATTTGAGGAAGAGAACAGAAATAGGAATGTGATTTTTGT

>HvAP2-11

>Protein

LAGAAGAHWTRPLSRTKSRRGPRSRSSQYRGVTFYRRTGRWESHIWDCGKQVYLGGFDTAQAAARAYDQAAIKFRGVEADINFLLDDYKEDIGKMSLLSKEELVQVLRRQGAGFVRGSSRFRGVTLHKCGKWEARIGQLMGKKFVYLGLYDTEMDAAKAYDKAALDCCGEEAMTNFEPKAAVAACDGDLDLHSWGGEPDLELSLGCSGGGNPSTVTTGDTLRTAAAPGRQRTSLTFEMPEEEEATAPWHPGRNRSIWIRPSPTSITSGLRCPDGDHRPAFTGSSMLHMVCDAVTNL

>cDNA

CCTGGCGGGAGCGGCGGGCGCGCACTGGACCCGGCCGCTGTCGAGGACCAAGAGCCGGCGCGGTCCGCGGTCCCGGAGCTCGCAGTACCGCGGCGTCACCTTCTACCGCCGCACCGGCCGCTGGGAGTCCCACATCTGGGACTGCGGGAAGCAGGTGTACCTGGGAGGATTCGACACAGCACAGGCTGCTGCAAGGGCGTACGACCAGGCGGCGATCAAGTTCCGGGGCGTGGAGGCGGACATCAACTTCCTGCTGGACGACTACAAGGAGGACATCGGCAAGATGAGCCTCTTGAGCAAGGAGGAGCTGGTGCAGGTGCTGCGGCGGCAGGGCGCCGGGTTCGTGAGGGGCAGCTCCCGGTTCCGCGGCGTCACCCTGCACAAGTGCGGCAAGTGGGAGGCCAGGATCGGCCAGCTCATGGGCAAGAAGTTCGTGTACCTTGGCCTCTATGACACGGAGATGGACGCTGCAAAGGCTTACGACAAGGCGGCGCTCGACTGCTGCGGCGAGGAGGCGATGACCAACTTCGAGCCCAAGGCAGCAGTGGCGGCGTGCGACGGCGATCTCGACCTGCACTCTTGGGGCGGCGAGCCTGATCTCGAGCTCAGCCTGGGCTGCTCCGGCGGCGGCAATCCCTCCACGGTCACCACCGGCGACACCCTCCGCACCGCAGCCGCCCCGGGGAGACAGAGGACGAGCCTGACGTTCGAAATGCCGGAGGAGGAGGAGGCGACGGCGCCGTGGCACCCTGGCAGGAACAGGAGCATCTGGATCAGGCCATCGCCGACATCGATAACGTCCGGGCTGAGGTGCCCGGACGGCGATCACCGCCCCGCCTTCACCGGCAGCAGCATGCTCCATATGGTATGTGATGCAGTAACAAACCTGTGACCAATGATCGCTTGACTGAAACCACTTGCTGACCAGCCCTCGCACCATGTCGTGTGACCGTTGAGCAGTGCCAGATCGGCGGAGGCGGCAGGGAAGAGCCACACATGCCCTGGTGGCCCACTGGCTCCGGCGTCGATCGTTGGCAGCCGTACGGCGCCGAGGCAGCAGCAGCAGCATCATCAGGATTCCCGCC

>CDS

CTGGCGGGAGCGGCGGGCGCGCACTGGACCCGGCCGCTGTCGAGGACCAAGAGCCGGCGCGGTCCGCGGTCCCGGAGCTCGCAGTACCGCGGCGTCACCTTCTACCGCCGCACCGGCCGCTGGGAGTCCCACATCTGGGACTGCGGGAAGCAGGTGTACCTGGGAGGATTCGACACAGCACAGGCTGCTGCAAGGGCGTACGACCAGGCGGCGATCAAGTTCCGGGGCGTGGAGGCGGACATCAACTTCCTGCTGGACGACTACAAGGAGGACATCGGCAAGATGAGCCTCTTGAGCAAGGAGGAGCTGGTGCAGGTGCTGCGGCGGCAGGGCGCCGGGTTCGTGAGGGGCAGCTCCCGGTTCCGCGGCGTCACCCTGCACAAGTGCGGCAAGTGGGAGGCCAGGATCGGCCAGCTCATGGGCAAGAAGTTCGTGTACCTTGGCCTCTATGACACGGAGATGGACGCTGCAAAGGCTTACGACAAGGCGGCGCTCGACTGCTGCGGCGAGGAGGCGATGACCAACTTCGAGCCCAAGGCAGCAGTGGCGGCGTGCGACGGCGATCTCGACCTGCACTCTTGGGGCGGCGAGCCTGATCTCGAGCTCAGCCTGGGCTGCTCCGGCGGCGGCAATCCCTCCACGGTCACCACCGGCGACACCCTCCGCACCGCAGCCGCCCCGGGGAGACAGAGGACGAGCCTGACGTTCGAAATGCCGGAGGAGGAGGAGGCGACGGCGCCGTGGCACCCTGGCAGGAACAGGAGCATCTGGATCAGGCCATCGCCGACATCGATAACGTCCGGGCTGAGGTGCCCGGACGGCGATCACCGCCCCGCCTTCACCGGCAGCAGCATGCTCCATATGGTATGTGATGCAGTAACAAACCTGTGA

>DNA

CCTGGCGGGAGCGGCGGGCGCGCACTGGACCCGGCCGCTGTCGAGGACCAAGAGCCGGCGCGGTCCGCGGTCCCGGAGCTCGCAGTACCGCGGCGTCACCTTCTACCGCCGCACCGGCCGCTGGGAGTCCCACATCTGGTCCGTCCGTCACCCTCCCTCCCCTTCCAAAATCCATGGCGACAGCAGAGCTTCGAACACCATCATCACGCAAACCGTCTTCCCTCGATCTTGCATTGCCCTCGTTTATATTTACTTACGCATCTCCGTCTCTCGTCCGTGTGTTCTTCCTATCTGCAGGGACTGCGGGAAGCAGGTGTACCTGGGTGAGTCCTACTTAGCTTTCTTCCTGCGTGGAACTTCATTCCTCACTGGTTCCTCCTAAATGCTCTGCTCATGACTCTCTTCTTGCTTCCCTTTTCCCTTCAATTCTTTCGCAGGAGGATTCGACACAGCACAGGCTGCTGCAAGGTCAGCAAAACCCCACATGGTTTCTTATCTTGAATGGAAAAAAAGAAAAGAAAAAATAAGAGCAGAGAGAAAACACATTTTTGCAAAATGAAACCTGAGGAACAAGAGCTTGTCTGGATTTCAGGGCGTACGACCAGGCGGCGATCAAGTTCCGGGGCGTGGAGGCGGACATCAACTTCCTGCTGGACGACTACAAGGAGGACATCGGCAAGGTGACCACCAACACACAAGAGCTCTCTCTCTCTCTCCCTCCCTCCCCTGTCCATTTCCTGCATCTTCCTCGTCGGCAATGTCTGCTCTGTTTCTGACGACAACAACATGTTGTTCATCTTGGTGACTGCCATGGCAGATGAGCCTCTTGAGCAAGGAGGAGCTGGTGCAGGTGCTGCGGCGGCAGGGCGCCGGGTTCGTGAGGGGCAGCTCCCGGTTCCGCGGCGTCACCCTGCACAAGTGCGGCAAGTGGGAGGCCAGGATCGGCCAGCTCATGGGCAAGAAGTCAGTCATCCACCTGCCTGCCCGTCAGCACAAAATGTGCTCCATTTGCCACACAGATGAACATGAAACTTTTTTTCTTCTTGTTAATATTAATCCTGTGTGATGATGATCGTTGCAGGTTCGTGTACCTTGGCCTCTATGACACGGAGATGGACGCTGCAAAGTTAGTAGACACAATCTCATCCAGTTTCTGCGCTCACTGATGCTCCTTCTGTTTCTGTGCCATGGCCCATGGATGATGATGATCTTACATGCTGTTTTTATGTTTGCTGATGATTTCCTGCCCTGTGCTGTGTGATTGACTGGATGATCAGGGCTTACGACAAGGCGGCGCTCGACTGCTGCGGCGAGGAGGCGATGACCAACTTCGAGCCCAAGGCAGCAGTGGCGGCGTGCGACGGCGATCTCGACCTGCACTGTGAGCTCCCTGACTGACTCATGGCAACAAACTCATTTTGGTTGCGGGCTTGGCATGTTCTGAAAATGGGAATTATTGTTGGCATCCTTTGTTGCAGCTTGGGGCGGCGAGCCTGATCTCGAGCTCAGCCTGGGCTGCTCCGGCGGCGGCAATCCCTCCACGGTCACCACCGGCGACACCCTCCGCACCGCAGCCGCCCCGGGGAGACAGAGGACGAGCCTGACGGTATGTCTAGGACACAATGAATTCTGTTGTTGCAATTTTGTTGCTAGATACACATGACTGAGTTGATTCGTGTGAAATTGACACTGAAACTGTTTGTTCGGGGACCAGTTCGAAATGCCGGAGGAGGAGGAGGCGACGGCGCCGTGGCACCCTGGCAGGAACAGGAGCATCTGGATCAGGCCATCGCCGACATCGATAACGTCCGGGCTGAGGTGCCCGGACGGCGATCACCGCCCCGCCTTCACCGGCAGCAGCATGCTCCATATGGTATGTGATGCAGTAACAAACCTGTGACCAATGATCGCTTGACTGAAACCACTTGCTGACCAGCCCTCGCACCATGTCGTGTGACCGTTGAGCAGTGCCAGATCGGCGGAGGCGGCAGGGAAGAGCCACACATGCCCTGGTGGCCCACTGGCTCCGGCGTCGATCGTTGGCAGCCGTACGGCGCCGAGGCAGCAGCAGCAGCATCATCAGGATTCCCGCC

>HvAP2-12

>Protein

MWDLNDSPAAEGPPLSPSVDDSGASSSSAAAVVEIPDDAEDDSAEAVVTRQFFPPAAPGEGGPGNGNDRAAWLRLAGAPAPTVAAAAGGGTGGPAAASAAAKKSRRGPRSRSSQYRGVTFYRRTGRWESHIWDCGKQVYLGGFDTAHAAARAYDRAAIKFRGMEADINFSLEDYDDIKQMGNLTKEEFVHVLRRQSTGFPRGSSKYRGVTLHKCGRWEARMGQFLGKKYVYLGLFDTEEEAARSYDRAAIKCNGKDAVTNFDPSTYAEEFEPAASTGDAEQQNLDLSLGSSAGSNKRGSLDGGGMDDDGAAGSDQRVPMAFELDWHTAAARSTKAKFDQNSARHQMPPPALQLQAPHMQFSPRHHQFVGNADPGTAGGLSLTVGAGAGGGQWPPPPPPHHYQPPHPQQHHQQQQQRLQHGWGNVVPGTSWQPVQPPPPPHHQAGPAPNNAAAAAAAAAASSRFPPYIATQAQSWLQKNGFHSLARPT

>cDNA

GGGCTCTGCTCTGCTCCCCTTTCCCCCCTCCCTCCCTCCCTCCCTCGTCGGGGCCCTGAGCACTAGCTTAGGTATGTTGGTGCAGCAGCGGAAGCCTTTGCTCTTCCACCACTCCATCTCCATCTCCATCTCCATCCACCACGAGCAGGAGCTGGACCTCTCCCCGTCGCCGCTGCCTTTTCCCTCCTCCTCTGCGCTCCTAGGTTAAGAAGAGGAGGTGCTTTTCAAAGGAGAGAGCAAAAGAGAAAGTCCTTGCATACCCCAACAAGGCAACCCGCCACTGCCTTTTTCTCCCCCTCCCCCCTCTCTCTTCCCTCCTCTGCTTTCTTGTTATCTTCAACCCCAGCCAACGAGAGCAGAGCCGAGCCGAGCCGAGCCGAACAGAGAGAGAGGCCGATAGGGGTGGACCGAAGAACCGGACGGCAAGAAGGTAGGAGGAGTGAGGAAGGGAAGGGAAGGGAAGGACGGCCGGCATGTGGGATCTCAACGACTCGCCGGCGGCCGAGGGGCCGCCGCTGTCCCCGTCCGTGGACGACTCCGGCGCCTCCTCCTCGTCTGCCGCCGCGGTGGTCGAGATACCGGACGACGCCGAGGACGACTCCGCCGAGGCCGTCGTCATGCGCCAGTTCTTCCCCCCGGCCGCCCCGGGCGAGGGCGGCCCCGGCAACGGGAACGACCGCGCCGCGTGGCTCCGCCTGGCCGGCGCCCCCGCGCCCACCGTGGCCGCGGCCGCCGGAGGAGGAACAGGAGGCCCCGCGGCGGCGTCGGCGGCGGCCAAGAAGAGCCGGCGCGGTCCCCGTTCCCGCAGCTCGCAGTACCGCGGCGTCACCTTCTACCGCCGGACGGGCCGGTGGGAGTCGCACATATGGGATTGCGGCAAGCAGGTCTATCTGGGTGGATTCGACACTGCTCATGCGGCGGCTCGGGCGTACGATCGGGCGGCGATCAAGTTCCGCGGCATGGAGGCCGACATCAATTTCAGCCTGGAGGACTACGACGACATCAAGCAGATGGGCAACCTGACCAAGGAGGAGTTCGTCCACGTGCTCCGGCGGCAGAGCACGGGGTTCCCCCGGGGGAGCTCCAAGTACAGGGGCGTCACGCTCCACAAGTGCGGCAGGTGGGAGGCGCGGATGGGCCAGTTCCTCGGCAAGAAGTACGTCTACTTGGGGCTGTTCGATACCGAGGAGGAAGCTGCCAGGTCGTACGACCGCGCTGCCATCAAGTGCAACGGCAAGGATGCGGTCACCAACTTCGATCCCAGCACCTACGCCGAGGAGTTCGAGCCGGCGGCTTCGACCGGCGACGCGGAGCAGCAGAACCTGGACCTGTCGCTGGGGAGCTCGGCGGGGTCGAACAAGAGGGGCAGCCTCGACGGCGGCGGCATGGACGACGACGGCGCGGCGGGGTCCGACCAGCGCGTCCCCATGGCCTTCGAGCTCGACTGGCACACGGCGGCGGCGCGCAGCACCAAGGCCAAGTTCGACCAGAACTCGGCGCGTCATCAGATGCCCCCTCCAGCCCTGCAACTGCAAGCCCCCCACATGCAGTTCAGTCCCAGGCATCACCAATTCGTGGGCAACGCCGATCCGGGGACAGCGGGAGGCCTGTCGCTGACGGTCGGCGCCGGCGCCGGGGGCGGGCAATGGCCTCCGCCTCCGCCTCCCCACCACTACCAGCCGCCGCATCCGCAGCAGCACCACCAGCAGCAGCAACAGAGGCTGCAGCACGGCTGGGGCAACGTCGTCCCCGGCACGAGCTGGCAGCCGGTCCAGCCGCCGCCGCCGCCGCACCACCAGGCGGGGCCGGCGCCGAACAACGCTGCCGCCGCCGCAGCAGCAGCAGCAGCGTCATCACGATTCCCACCCTACATCGCCACGCAGGCGCAGAGCTGGCTCCAGAAGAACGGGTTCCACTCCCTGGCCAGACCCACCTAGCCGCTTGCAGAGACACCATCAAGAGCAGCCAGCCAAGAAGATCTTCTTTCTTCATCCATCATCCATGGCGAAGAAGATGGATCGACAGGTGTGTTCCGCCGAACCAACCGGGCGAAATCACGGAGGAAAAACTCAAGTTTCCTCGTCCCACAGCCATTACCAGCAGAACCGAACTGAACTGAGAGTCTGAGACCGGATGCTAGTATTCTTTTTTACCATTTCATGTTTTCTTTCTTGCTCTTTTTCTTGATTGATTGGGTGAGAGCGAGAGATGGATTTGAGAGGAGGACGGATGCGGTCATGTGTAAATGCAGAGTTCCCCAAGAAGACGAGAGACATTGGTTCTAGCTCTAGCTAGCTTTCAACTCAAGTGTGTGTTTGTGGTGGAGC

>CDS

ATGTGGGATCTCAACGACTCGCCGGCGGCCGAGGGGCCGCCGCTGTCCCCGTCCGTGGACGACTCCGGCGCCTCCTCCTCGTCTGCCGCCGCGGTGGTCGAGATACCGGACGACGCCGAGGACGACTCCGCCGAGGCCGTCGTCATGCGCCAGTTCTTCCCCCCGGCCGCCCCGGGCGAGGGCGGCCCCGGCAACGGGAACGACCGCGCCGCGTGGCTCCGCCTGGCCGGCGCCCCCGCGCCCACCGTGGCCGCGGCCGCCGGAGGAGGAACAGGAGGCCCCGCGGCGGCGTCGGCGGCGGCCAAGAAGAGCCGGCGCGGTCCCCGTTCCCGCAGCTCGCAGTACCGCGGCGTCACCTTCTACCGCCGGACGGGCCGGTGGGAGTCGCACATATGGGATTGCGGCAAGCAGGTCTATCTGGGTGGATTCGACACTGCTCATGCGGCGGCTCGGGCGTACGATCGGGCGGCGATCAAGTTCCGCGGCATGGAGGCCGACATCAATTTCAGCCTGGAGGACTACGACGACATCAAGCAGATGGGCAACCTGACCAAGGAGGAGTTCGTCCACGTGCTCCGGCGGCAGAGCACGGGGTTCCCCCGGGGGAGCTCCAAGTACAGGGGCGTCACGCTCCACAAGTGCGGCAGGTGGGAGGCGCGGATGGGCCAGTTCCTCGGCAAGAAGTACGTCTACTTGGGGCTGTTCGATACCGAGGAGGAAGCTGCCAGGTCGTACGACCGCGCTGCCATCAAGTGCAACGGCAAGGATGCGGTCACCAACTTCGATCCCAGCACCTACGCCGAGGAGTTCGAGCCGGCGGCTTCGACCGGCGACGCGGAGCAGCAGAACCTGGACCTGTCGCTGGGGAGCTCGGCGGGGTCGAACAAGAGGGGCAGCCTCGACGGCGGCGGCATGGACGACGACGGCGCGGCGGGGTCCGACCAGCGCGTCCCCATGGCCTTCGAGCTCGACTGGCACACGGCGGCGGCGCGCAGCACCAAGGCCAAGTTCGACCAGAACTCGGCGCGTCATCAGATGCCCCCTCCAGCCCTGCAACTGCAAGCCCCCCACATGCAGTTCAGTCCCAGGCATCACCAATTCGTGGGCAACGCCGATCCGGGGACAGCGGGAGGCCTGTCGCTGACGGTCGGCGCCGGCGCCGGGGGCGGGCAATGGCCTCCGCCTCCGCCTCCCCACCACTACCAGCCGCCGCATCCGCAGCAGCACCACCAGCAGCAGCAACAGAGGCTGCAGCACGGCTGGGGCAACGTCGTCCCCGGCACGAGCTGGCAGCCGGTCCAGCCGCCGCCGCCGCCGCACCACCAGGCGGGGCCGGCGCCGAACAACGCTGCCGCCGCCGCAGCAGCAGCAGCAGCGTCATCACGATTCCCACCCTACATCGCCACGCAGGCGCAGAGCTGGCTCCAGAAGAACGGGTTCCACTCCCTGGCCAGACCCACCTAGCCGCTTGCAGAGACACCATCAAGAGCAGCCAGCCAAGAAGATCTTCTTTCTTCATCCATCATCCATGGCGAAGAAGATGGATCGACAGGTGTGTTCCGCCGAACCAACCGGGCGAAATCACGGAGGAAAAACTCAAGTTTCCTCGTCCCACAGCCATTACCAGCAGAACCGAACTGA

>DNA

GGGCTCTGCTCTGCTCCCCTTTCCCCCCTCCCTCCCTCCCTCCCTCGTCGGGGCCCTGAGCACTAGCTTAGGTATGTTGGTGCAGCAGCGGAAGCCTTTGCTCTTCCACCACTCCATCTCCATCTCCATCTCCATCCACCACGAGCAGGAGCTGGACCTCTCCCCGTCGCCGCTGCCTTTTCCCTCCTCCTCTGCGCTCCTAGGTTAAGAAGAGGAGGTGCTTTTCAAAGGAGAGAGCAAAAGAGAAAGTCCTTGCATACCCCAACAAGGCAACCCGCCACTGCCTTTTTCTCCCCCTCCCCCCTCTCTCTTCCCTCCTCTGCTTTCTTGTTATCTTCAACCCCAGCCAACGAGAGCAGAGCCGAGCCGAGCCGAGCCGAACAGAGAGAGAGGCCGATAGGGGTGGACCGAAGAACCGGACGGCAAGAAGGTAGGAGGAGTGAGGAAGGGAAGGGAAGGGAAGGACGGCCGGCATGTGGGATCTCAACGACTCGCCGGCGGCCGAGGGGCCGCCGCTGTCCCCGTCCGTGGACGACTCCGGCGCCTCCTCCTCGTCTGCCGCCGCGGTGGTCGAGATACCGGACGACGCCGAGGACGACTCCGCCGAGGCCGTCGTCATGCGCCAGTTCTTCCCCCCGGCCGCCCCGGGCGAGGGCGGCCCCGGCAACGGGAACGACCGCGCCGCGTGGCTCCGCCTGGCCGGCGCCCCCGCGCCCACCGTGGCCGCCGCCGCCGGAGGAGGAACAGGAGGCCCCGCGGCGGCGTCGGCGGCGGCCAAGAAGAGCCGGCGCGGTCCCCGTTCCCGCAGCTCGCAGTACCGCGGCGTCACCTTCTACCGCCGGACGGGCCGGTGGGAGTCGCACATATGGTAAGCTCCCGCCGCCGCTCCGCTCCTTTCCTTTCCTTTCTCGAAAGCTTAAGGAAGGAAGGAGAGAGAGTGCAGCAAAAGAAGAGAAGAGAAGAGAAGATGGATGGATGGATGGGTTTGGTATTGGATTCGTCTTGTTTTGGGCATTCCATGCCTCTGCTCCTTTTACCTTTCATAGCAACATCTTATCAAACTTATTCCCCTTCCTTCCTTGTTTCTCTCTCTTCCCCCCTCTCCTCTCCTCTTCTCACCCCATTCTTTTGTGCAATGCAGGGATTGCGGCAAGCAGGTCTATCTGGGTAAGTTTCCTCCATCCCCGTCTTGTCCACACATCCATACTTGGGGTGCGAAATCGATCGCCAATTGGAGCTCACAATGTCACTGACGGCCTGCAGGTGGATTCGACACTGCTCATGCGGCGGCTCGGTACGATAAAGATCCCATACCAACCGATTTGACAGTACCAATCAAGCTCTCTTCTTTACTGATTTCTTCTTCTTCATCATCATCTGCGGCCGCAGGGCGTACGATCGGGCGGCGATCAAGTTCCGCGGCATGGAGGCCGACATCAATTTCAGCCTGGAGGACTACGACGACATCAAGCAGGTGAGCGACGCCCGATCGATCAACACGAGCTAGCGCTTGTTGCTCGCAGAATCGTAACGTGTTTGCCCTGGTTTGCTTGCAGATGGGCAACCTGACCAAGGAGGAGTTCGTCCACGTGCTCCGGCGGCAGAGCACGGGGTTCCCCCGGGGGAGCTCCAAGTACAGGGGCGTCACGCTCCACAAGTGCGGCAGGTGGGAGGCGCGGATGGGCCAGTTCCTCGGCAAGAAGTATGTGCTCCTCCACCAACCACCATCGCCACCCTCCTTCCTCCCTTCTCCTCCAATCGATGGCTCGAATTTTTGCTCTGCTCGTCTGCCTGCTCCTGTCGTTGCGCTCAAAGTTGCAGCATCTCCCTGCTAACTAGCCTGACGGCATGGCATGGCATGGCAGCGGCACTAATGAGAATCTTTGTGCAACGCAGGTACGTCTACTTGGGGCTGTTCGATACCGAGGAGGAAGCTGCCAGGTAGTAAAAACTGAAAAATTATTGGGCATGCACTGTGCGCGCGTCCTCCATCCATTGCTAGCTTTAGCTTTGCTCGGGCTGGGGTTGGGCGGTGAACTGATTGATGTCTCGTGTGTCTCTGTGTCTGGGCAGGTCGTACGACCGCGCTGCCATCAAGTGCAACGGCAAGGATGCGGTCACCAACTTCGATCCCAGCACCTACGCCGAGGAGTTCGAGCCGGCGGGTCAGTAATAATCTTGTTATATTCATCGCGCACTGTTCATCTGTGATTTTGATTTGGCCACCATAGGATTATGCGGCATACGTATGTTTCTTCCGGTTCGTTCACTGACCTTGATCTTGATCTTGGGTGGGCAATGCTCTGCCAGCTTCGACCGGCGACGCGGAGCAGCAGAACCTGGACCTGTCGCTGGGGAGCTCGGCGGGGTCGAACAAGAGGGGCAGCCTCGACGGCGGCGGCATGGACGACGACGGCGCGGCGGGGTCCGACCAGCGCGTCCCCATGGCCTTCGAGCTCGACTGGCACACGGCGGCGGCGCGCAGCACCAAGGCCAAGGTACAGGCCCCAACTTGACCTTGACCACCCTGCAACAAACTACTGCTCCTGCATCATAGTATAATCAAGCGTTGGAAAAGCTGAGAATGGCGTAATTTACTATGCAGTTCGACCAGAACTCGGCGCGTCATCAGATGCCCCCTCCAGCCCTGCAACTGCAAGCCCCCCACATGCAGTTCAGTCCCAGGCATCACCAAGTGGGTACTTTTGCCGCTCCAAGTTGGAGCTCACGAATTTTTCTATCTCTTCTCTTCTTGGTAGGCGCGAATTAACGGTGGTTGTTTGGTTCAAACTGTGCAGTTCGTGGGCAACGCCGATCCGGGGACAGCGGGAGGCCTGTCGCTGACGGTCGGCGCCGGCGCCGGGGGCGGGCAATGGCCTCCGCCTCCGCCTCCCCACCACTACCAGCCGCCGCATCCGCAGCAGCACCACCAGCAGCAGCAACAGAGGCTGCAGCACGGCTGGGGCAACGTCGTCCCCGGCACGAGCTGGCAGCCGGTCCAGCCGCCGCCGCCGCCGCACCACCAGGCGGGGCCGGTGCCGAACAACGCTGCCGCCGCCGCAGCAGCAGCCGCAGCATCATCACGATTCCCACCCTACATCGCCACGCAGGCGCAGAGCTGGCTCCAGAAGAACGGGTTCCACTCCCTGGCCAGACCCACCTAGCCGCTTGCAGAGACACAATCAAGAGCAGCCAGCCAAGAAGATCTTCTTTCTTCATCCATCATCCATGGCGAAGAAGATGGATCGACAGGTGTGTTCCGCCGAACCAACCGGGCGAAATCACGGAGGAAAAACTCAAGTTTCCTCGTCCCACAGCCATTACCAGCAGAACCGAACTGAACTGAGAGTCTGAGACCGGATGCTAGTATTCTTTTTTACCATTTCATGTTTTCTTTCTTGCTCTTTTTCTTGATTGATTGGGTGAGAGCGAGAGATGGATTTGAGAGGAGGACGGATGCGGTCATGTGTAAATGCAGAGTTCCCCAAGAAGACGAGAGACATTGGTTCTAGCTCTAGCTAGCTTTCAACTCAAGTGTGTGTTTGTGGTGGAGC

>HvAP2-13

>Protein

MKQMKGLSKEEFVHVLRRQSTGFSRGSSKYRGVTLHKCGRWEARMGQFLGKKYIYLGLFDNEVEAARAYDKAAIKCNGREAVTNFEPSTYDAELLNEAAAEGADVDLNLSISQPTSQSPKRDKSTLGLQLHHGSYEGSELKRPKVDAPPEMVAIPHRYPVLTEHPPIWHGQSYPLFLNNEDAARDHSRRPEVATGGVPTWAWRVSHPPPTQPMPLFSSSSSAAASSGFSKTAAAAAPAAPSASFRFDPMAPSSSSSNQHHHHHPR

>cDNA

ACCGGCCGCTGGGAATCCCATATCTGGGATTGCGGCAAGCAGGTGTACTTGGGTGGATTTGACACAGCACATGCTGCTGCAAGGGCGTACGACCGAGCGGCGATCAAGTTCCGCGGCGTCGACGCCGACATAAACTTCAACCTCAGTGACTATGAGGACGACATGAAGCAGATGAAGGGCCTGTCCAAGGAGGAGTTCGTGCACGTGCTGCGGCGGCAGAGCACCGGCTTCTCGCGGGGCAGCTCCAAGTACAGAGGCGTCACCCTGCACAAGTGCGGCCGGTGGGAGGCGCGCATGGGCCAGTTCCTCGGCAAGAAGTACATATATCTTGGGCTATTCGACAATGAAGTAGAGGCTGCAAGGGCTTACGACAAGGCGGCGATCAAATGCAATGGTAGAGAGGCCGTGACGAACTTCGAGCCGAGCACCTATGATGCGGAGCTGCTCAATGAAGCTGCTGCTGAAGGCGCTGATGTCGACCTCAACTTGAGCATATCTCAACCAACTTCACAAAGTCCCAAAAGGGATAAGAGCACCCTTGGCCTGCAGCTCCACCATGGATCATATGAAGGCTCTGAACTAAAGAGACCAAAGGTCGATGCCCCCCCTGAGATGGTCGCAATCCCTCATCGGTACCCCGTTCTGACCGAGCATCCACCAATCTGGCATGGCCAATCATATCCCCTCTTCTTAAATAATGAGGATGCAGCCAGAGATCATAGCAGGAGGCCAGAGGTGGCCACAGGGGGTGTTCCAACCTGGGCATGGAGGGTGAGCCACCCTCCTCCAACACAACCCATGCCACTCTTCTCGTCGTCATCGTCCGCTGCAGCATCATCAGGATTCTCCAAAACAGCCGCGGCAGCTGCCCCCGCCGCCCCATCGGCCTCATTCCGGTTCGACCCGATGGCTCCATCATCGTCGTCAAGCAACCAACACCACCACCACCACCCCCGCTGAAAAAGAAGCCATACTGTAAATGTTCTGGGAAGCCAGTATCTTTTTTGCTCCTCCGGCGTTTCAGCGTTTTCGGTCTTGCGCCGGGGCGGTTTCATGTAGTGGATTGGATTCATGACTGTATTTCCCATGCTGCCCAAGTGAAATGTCCCTTCTTTTTTGCGCTCTCTGCATCGGCGCAATGGTCCCATAATTCTCGCTGTCAGATACTAACCTCAGCTCATCTCACCATCTGAGATGGATTCATACCATTGTTGTAGACAAACTTGTCACTGAAATTCAGCAGTACCGATACCATAAGATAAGAGGGACCTTTACTTTGGGAATTATTCCAGTCCCTTTTCTTGGATGTCACTTGGCTCTTGTTTTTGCTCTAGAGCCAACTACCACTGCTGAATTGTACTGTTGTTTGTTCTTGCACTGTTACAAGATTTGGCTCTAACGGATGGCAAAGCATCTCGTGCAAGA

>CDS

ATGAAGCAGATGAAGGGCCTGTCCAAGGAGGAGTTCGTGCACGTGCTGCGGCGGCAGAGCACCGGCTTCTCGCGGGGCAGCTCCAAGTACAGAGGCGTCACCCTGCACAAGTGCGGCCGGTGGGAGGCGCGCATGGGCCAGTTCCTCGGCAAGAAGTACATATATCTTGGGCTATTCGACAATGAAGTAGAGGCTGCAAGGGCTTACGACAAGGCGGCGATCAAATGCAATGGTAGAGAGGCCGTGACGAACTTCGAGCCGAGCACCTATGATGCGGAGCTGCTCAATGAAGCTGCTGCTGAAGGCGCTGATGTCGACCTCAACTTGAGCATATCTCAACCAACTTCACAAAGTCCCAAAAGGGATAAGAGCACCCTTGGCCTGCAGCTCCACCATGGATCATATGAAGGCTCTGAACTAAAGAGACCAAAGGTCGATGCCCCCCCTGAGATGGTCGCAATCCCTCATCGGTACCCCGTTCTGACCGAGCATCCACCAATCTGGCATGGCCAATCATATCCCCTCTTCTTAAATAATGAGGATGCAGCCAGAGATCATAGCAGGAGGCCAGAGGTGGCCACAGGGGGTGTTCCAACCTGGGCATGGAGGGTGAGCCACCCTCCTCCAACACAACCCATGCCACTCTTCTCGTCGTCATCGTCCGCTGCAGCATCATCAGGATTCTCCAAAACAGCCGCGGCAGCTGCCCCCGCCGCCCCATCGGCCTCATTCCGGTTCGACCCGATGGCTCCATCATCGTCGTCAAGCAACCAACACCACCACCACCACCCCCGCTGA

>DNA

TATATTCCACATGTTTCGGAAGCATCGTGATCTTGCTTGCATCGACTAGAGAATTGCTCTGAACCTTTTGTAAGCATCACGTTCTTGCTTCCATCGACTAGACGACTCCTTTGAACCTGCTTATGACAAAAAGGAAAAATATTCCACATGTTTCGGAAGCATCATAATCTTGCTTCCATCGACTAGACGACTGCTTCGAGCCTTCTTCTCTCGAAAAAGAAATCTTGCTTCCATCGACTAGACAATTGCTTCAAACCTTGTGCCGTCAAAAAGGAAAAATATTCCACATGTTTTAGAAGCATCACGATCTTGCTTCCATCGATTAGACGACTGCTTCAAACCTTCTTTTGTCAAAAATGATAAATATTGCATATGTTTAGGAAACATCGTGATCTTGCTTCCAAAAAGAATATTATTTCACTTCCATTAAAAAGAACAATATTTCATCATAGTTACACTAGTTCATATAAATTCTTAGTTTGTTTACAAAGAGAGAAAAGGTTCTACTGCCAGGCAAAGTCAAATACTCAAATTGCATCCAAGCGTCGAAATCAATGGCTGGTACAAACATTTTCCATAGTGGTTATGGTTGGTAATGGTCATACCATAATAATCAGTAAAACAGTGTGTTGGAGAGGATGGCGATCAAAACTGGTACGTAAGTCAATGCATCTGTCCAAAAACTTGTTCATGTATCAGTAGAAGATTCGTCAGCTACCACATTCTTTTTTGGTTTTTTGAGAAAAAGCTACCACATTCTATTTAACTAGATATGAGCAGAAGGTTGGGTGTTACACAACCCCTTTTATTCATTAGTTACTGGCCTTGAAGGGAGTAGATTGTGAAGCCACTTGGTTTCACAAGAAGGCGCGATGTTCCTCCCTACCCCCTAGAAAAGAAAAATGAAATGAAGCAAAGTCGCACATCGATGCCCAAAAAATAAATCGGCTAAAAATTAAAAGAAAAATCCGAGGCCATCTCGAAAAGAAGATATAGCCATCAGTATCTACGTAATTCTACTGTGAACCCTTCAAGGAAAAAAATCATCTCACGGCCGCCAATGTTGATGGACCCAACTAAAGACTGAACTGAGAATATCATCCCTAAAGCCGAGCTTTCAAGCCACCTCCTTCAACAAGGGCACAATGCACATGCGCTAACATTATCTATTCTAGTTCAGAGAGGTGAGATCATGGTTTTAACTTGGAGTGAGTGCATACTTGTCGTCGAGCCCATAACTGTCAACAACCTCTCTAACGGATGACGATCTCCACTAGCCGGATCTCGACACTTAGAGCATCTCCAGCCGTTCGGCCCATCAAACACGTTCTTAGGTGGAAAAATAGCACCTCTTGGGGGCCATCCGGCGATATTTTCGGCACGGGGGTGCTCTGGCTCCCAGCCCCCCTCCCTCCCCCCCCCCCTTCTACCGCCGCACCGGCCGCTGGGAATCCCATATCTGGTCCGTACACAAATCCTTCCAGTCCCAACCACCCCCCAAAAAGAAACCCAATTTTTTCCTCAAATATCACCAGCCGATTCACAAATTTTCTTGCTCCTGTTCATCGCTTAATGCAGGGATTGCGGCAAGCAGGTGTACTTGGGTGGATTTGACACAGCACATGCTGCTGCAAGGTACTACAAATTTAATTAAGCGCGTAGGAGTGCTACATAATTGTGATGTGATCATCACCTGAGCTGCCTGTACTGAAACTCTGAAGTCATGCCCACCCCGTTCATTCCACCGTGCCAAATTGACCTTGGGATGTTCCGCAGGGCGTACGACCGAGCGGCGATCAAGTTCCGCGGCGTCGACGCCGACATAAACTTCAACCTCAGTGACTATGAGGACGACATGAAGCAGGTGATCACCTGTGCCACCAACCAGTGTTCCTCATCCAACCAAATAATTCAGATTCAGGGAGCATTAGTACTGTTGTTGGGACTGATGAACATAAGGAATTCTGACTTCTGACTGTTTTTGTTGCTTGGTGTATGGTATGAAGCAGATGAAGGGCCTGTCCAAGGAGGAGTTCGTGCACGTGCTGCGGCGGCAGAGCACCGGCTTCTCGCGGGGCAGCTCCAAGTACAGAGGCGTCACCCTGCACAAGTGCGGCCGGTGGGAGGCGCGCATGGGCCAGTTCCTCGGCAAGAAGTAAGAACAATTTCCCCTTCCATTTTGAACCAATTTTACATTTCTTGCTTCATGGCATGTTGCACTGAATGCACTTGTGAAGTTACATATGCATCTCTGGATGTGATTGTGCTGCGGCCTATCTGATCTGAATCTGAATCTCATAACAGAGTCATGATTAGATCAAACGAGATCCCATGATCCATTAGGAATGTATTTTTAAGCAGTATAGTAAGTAGTTCTTAGGTTGAAAATAAGATGCATAGATCAGGAAGAATGAAATGAAATACAGTAGTAGGCAGTGGATAGGGAGTTCCTCAAACGACATGCTTGGGTGCAGGTACATATATCTTGGGCTATTCGACAATGAAGTAGAGGCTGCAAGGTTCCTGAGCTTGGATTCTCCCCATTGATGCACAATAAAAAAGTTGTTTCTTTTTTCTCCATCAACCGAAGTACCGACTCGAATCTCTTCTCTTTGTTTTCCTCCTCCTTTTTCTTGTCCCGGCAAAAATCAGGGCTTACGACAAGGCGGCGATCAAATGCAATGGTAGAGAGGCCGTGACGAACTTCGAGCCGAGCACCTATGATGCGGAGCTGCTCAATGAAGCTGCTGCTGAAGGTAACAACATGACAGCAAATTTGATGGATTATTTTTTCATGTCTGCATCCGTGATTTAGCAAAAAAAATCTGCGTTGGCGATTAGCTGATGAACTTGAATTCAATTGGCAGGCGCTGATGTCGACCTCAACTTGAGCATATCTCAACCAACTTCACAAAGTCCCAAAAGGGATAAGAGCACCCTTGGCCTGCAGCTCCACCATGGATCATATGAAGGCTCTGAACTAAAGAGACCAAAGGCAAGTACAAATGATTTCTGCCTTTCTTTGAAAGATTGCACACCAGTCCAATTTCACTGCTGGATTTTGTATGGCTACCTGATCCACTATGTGACTAATAATTGAGTTTCACTGTCACCATGCTAGTGACTGCAGTGTGATGCCCTATCGTAACAAAGTCTTTTTCCTAGTTTACTTCATAGGAACTTCTACAGCTATTGTCAAAATTAAATTTCACCGTCGCCTGATCCACCGCCCAAATTATGTTCACCACACAGTAACATGCTTACGTTTCCTGCCTGACTATGGTTATCTATTTATTTTTCATTCAGAAATTAAATAGCTTGGGAAGATTATTAGTTGACATCCAACTGGAAAAAATCTTTTGCGTGTTACGCAGGTCGATGCCCCCCCTGAGATGGTCGCAATCCCTCATCGGTACCCCGTTCTGACCGAGCATCCACCAATCTGGCATGGCCAATCATATCCCCTCTTCTTAAATAATGAGGTGAGGTGATGCTAAATTTTAAAGAACCTTTCACCTAAAATTTTCATGATGGCTCTGATGACGCCCGATACGGATCGCACCGTGCATATTGAGCATGTCGACGCCACTCTGTTTTGCAATCTGGACAGGCTGATGCCGACTGACAGTGCCCTTGTAGCTGTTAGTGTTGTACTTAGCGTCAATTAAACATTTTTCTGTGTGGCTGCAGTGATAGCTCAAATCTTTTGAATACATATGCCTGATTTCCATAGCCAATCCATTCCCATGTCGATTTCAGAGTCATTAATTCCTACCATAGTTTTCGTATGTAGGATAAAACTGTCTAGCTAATTGGGGTAGAACTACTACTGGTCATAGGATATATATATATAGAATATGCTTGATTTTTTTTTTCCTCAAGGAACATCCTAGAGAGTTGACGATTCAGATTTCGCCAACTCCAATAATTTGGTCAATATTCCTTCATGTGGCTAAGAACGTTTGGTTGTTCTATTTTTTATGCTATTATTTATAATAGTATGACCACTATTCAGAGGTTTTATTTATTTATTTATAGCGAGTAAAGGGATCTGAATCGTACCAATACTATTTTACTAGTATGAGTACGGCTCACAGCTTTACTTGGAAGCAAAAGAAAGCTAGCAAGTACCTTATAGTAATAGGATTAGTACACAGTTTGCCATTTGTTTTGAAAATCCAAAATTGGTTTGCCATTATCCTCTCAGATTTCTCAACTACTAAACTTCTCGAGTATCTTCACATGTGTCATTTTCTTATTTTATTTTTCCTGGAAAGAAAGTTCAGCTGGACATATCTGCCTCACCTTTATGTCGATCAAAGCGATAGGAATTAGACCAATTCTACAAAAGAAACAATGAACAATTCAATTTCTGTCAATTTTTTTTGAATTTATCCTACATGCGCAACCTAAATTATTCTTCATTTATGCAGGATGCAGCCAGAGATCATAGCAGGAGGCCAGAGGTGGCCACAGGGGGTGTTCCAACCTGGGCATGGAGGGTGAGCCACCCTCCTCCAACACAACCCATGCCACTCTTCTCGTCGTCATCGTCCGCTGCAGCATCATCAGGATTCTCCAAAACAGCCGCGGCAGCTGCCCCCGCCGCCCCATCGGCCTCATTCCGGTTCGACCCGATGGCTCCATCATCGTCGTCAAGCAACCAACACCACCACCACCACCCCCGCTGAAAAAGAAGCCATACTGTAAATGTTCTGGGAAGCCAGTATCTTTTTTGCTCCTCCGGCGTTTCAGCGTTTTCGGTCTTGCGCCGGGGCGGTTTCATGTAGTGGATTGGATTCATGACTGTATTTCCCATGCTGCCCAAGTGAAATGTCCCTTCTTTTTTGCGCTCTCTGCATCGGCGCAATGGTCCCATAATTCTCGCTGTCAGATACTAACCTCAGCTCATCTCACCATCTGAGATGGATTCATACCATTGTTGTAGACAAACTTGTCACTGAAATTCAGCAGTACCGATACCATAAGATAAGAGGGACCTTTACTTTGGGAATTATTCCAGTCCCTTTTCTTGGATGTCACTTGGCTCTTGTTTTTGCTCTAGAGCCAACTACCACTGCTGAATTGTACTGTTGTTTGTTCTTGCACTGTTACAAGATTTGGCTCTAACGGATGGCAAAGCATCTCGTGCAAGA

>HvAP2-14

>Protein

MVLDLNVESPADSGTSSSSVLNSADAAGAFRFGLLGSLDDDDCSGELAPAAASGFVTRQLFPAPPPAPGVMMGQAPAPPPTAPVWQPRRAEELVVAQRVAPKKKTRRGPRSRSSQYRGVTFYRRTGRWESHIWDCGKQVYLGGFDTAHAAARAYDRAAIKFRGLEADINFNLSDYEEDLKQMRNWTKEEFVHILRRQSTGFARGSSKYRGVTLHKCGRWEARMGQLLGKKYIYLGLFDSEVEAARAYDRAAIRFNGRDAVTNFDSSSYNGDATPDVENEAIVDADALDLDLRMSQPTAHDPKRDNIIAGLQLTFDSPESSTTMVSSQPMSSSSQWPVHQHGTAVPPQQHQRLYPSACHGFYPNVQVQVQERPLEPRPPEPSSFPGWGWHAQAVPPGSSHSLLLYAAASSGFSTAAGANPAPPPSYPDHHHRFYFPRPPDN

>cDNA

CTGGGGCTTGGCCACCTCGACGTCCCATCTCGCTTTCTTTCTCTTTCTCTTTCCCTTTCTCCCACTGGCGCAGGGCCCTCTCCTCGTCCTCCTCCAGTCCTCATCCCCATGGCCACCACCACCGCCTCCACCCCCCACCACTACTTCTACTCCTCCCGCCTCGCCCTCGCAGCCCGCGGCCACCGCGCTCCCATGCCATAGACGCCAGCCAGCTGATCGGTCGACCTCGCTCGGACGCGGCACGCGGCGAGCGAGGCGGGCATACCTGCGTTTCATTTGCGAGCTCCCGGCGCGCGGGCCTCGGATCGGAGGACGGAGATGGTGCTGGATCTCAATGTCGAGTCTCCGGCCGACTCCGGCACGTCCAGCTCGTCCGTGCTCAACTCCGCGGACGCCGCCGGCGCCTTCCGGTTCGGCCTGCTCGGGAGCCTCGATGACGACGACTGCTCCGGCGAGCTGGCGCCGGCCGCCGCGTCCGGGTTCGTGACGAGGCAGCTGTTCCCCGCGCCGCCGCCCGCGCCCGGGGTCATGATGGGGCAGGCGCCGGCGCCTCCGCCCACGGCGCCGGTGTGGCAACCTCGGCGCGCCGAGGAGCTGGTCGTGGCGCAGCGGGTGGCCCCCAAGAAGAAGACGCGGCGGGGGCCGAGGTCGCGCAGCTCGCAGTACCGGGGCGTCACCTTCTACCGCAGGACCGGCCGCTGGGAGTCGCACATCTGGGATTGCGGGAAGCAGGTCTACTTGGGTGGTTTCGACACCGCGCACGCGGCCGCAAGGGCCTACGATCGCGCGGCGATCAAGTTCCGGGGGCTGGAGGCCGACATCAACTTCAATCTGAGCGACTACGAGGAGGATTTGAAGCAGATGAGGAACTGGACCAAGGAGGAGTTCGTGCATATCCTCCGCCGCCAGAGCACCGGGTTCGCCAGGGGGAGCTCGAAGTACCGCGGCGTCACGCTCCACAAGTGCGGCCGCTGGGAGGCAAGGATGGGCCAACTGCTCGGCAAGAAGTACATATATCTTGGGCTGTTCGACAGCGAAGTTGAAGCTGCAAGAGCGTACGACAGGGCGGCGATTCGCTTCAATGGGAGGGATGCTGTGACTAACTTTGATAGTAGCTCCTACAATGGAGATGCTACACCTGACGTCGAAAATGAGGCAATTGTTGATGCTGATGCTCTTGACTTGGATCTAAGGATGTCGCAACCTACCGCGCACGATCCCAAGAGGGACAACATCATCGCCGGCCTTCAGTTAACTTTTGATTCCCCTGAGTCGTCAACCACAATGGTCTCCTCTCAGCCAATGAGCTCATCGTCCCAGTGGCCTGTGCATCAACATGGCACAGCAGTACCACCTCAGCAGCACCAGCGTTTGTACCCATCTGCTTGTCATGGCTTCTACCCCAACGTACAGGTGCAGGTGCAGGAGAGGCCCTTGGAGCCAAGGCCCCCTGAGCCGTCCTCCTTCCCCGGCTGGGGGTGGCACGCGCAGGCCGTGCCGCCGGGCTCCTCCCACTCGTTGTTGCTTTATGCTGCAGCATCATCAGGATTCTCTACCGCCGCCGGCGCGAACCCCGCCCCGCCGCCGTCGTACCCCGACCATCACCACCGCTTCTACTTCCCCCGCCCGCCGGACAACTAGCTCTCTGCTTACTCCCATGGACGGTGGTCGACGGGTGTGGGCGTGTGCGCGGGCGCGATCGACGTGTTCGCGCCTTGTCGGTAACAGTTGTTGTGAATTAATCGGAGAGAGATAACATTGCCGAGCCATGTGTCACTGGCTGGTTCCTCTCCCTCGCCATGATCAGAATCACAGGCATCATGGTTTCCCGTTCAGACTATGTATCCATGCTTTCAATCATGATCATGATCATGCGGTTGTCCATTACTAGATTCTCATGTATCCATGTTCAAGTTTGTGAAACAGCTGAAAAACCTTGGAAATTAATGGCGGCAGGTTCATGCG

>CDS

ATGGTGCTGGATCTCAATGTCGAGTCTCCGGCCGACTCCGGCACGTCCAGCTCGTCCGTGCTCAACTCCGCGGACGCCGCCGGCGCCTTCCGGTTCGGCCTGCTCGGGAGCCTCGATGACGACGACTGCTCCGGCGAGCTGGCGCCGGCCGCCGCGTCCGGGTTCGTGACGAGGCAGCTGTTCCCCGCGCCGCCGCCCGCGCCCGGGGTCATGATGGGGCAGGCGCCGGCGCCTCCGCCCACGGCGCCGGTGTGGCAACCTCGGCGCGCCGAGGAGCTGGTCGTGGCGCAGCGGGTGGCCCCCAAGAAGAAGACGCGGCGGGGGCCGAGGTCGCGCAGCTCGCAGTACCGGGGCGTCACCTTCTACCGCAGGACCGGCCGCTGGGAGTCGCACATCTGGGATTGCGGGAAGCAGGTCTACTTGGGTGGTTTCGACACCGCGCACGCGGCCGCAAGGGCCTACGATCGCGCGGCGATCAAGTTCCGGGGGCTGGAGGCCGACATCAACTTCAATCTGAGCGACTACGAGGAGGATTTGAAGCAGATGAGGAACTGGACCAAGGAGGAGTTCGTGCATATCCTCCGCCGCCAGAGCACCGGGTTCGCCAGGGGGAGCTCGAAGTACCGCGGCGTCACGCTCCACAAGTGCGGCCGCTGGGAGGCAAGGATGGGCCAACTGCTCGGCAAGAAGTACATATATCTTGGGCTGTTCGACAGCGAAGTTGAAGCTGCAAGAGCGTACGACAGGGCGGCGATTCGCTTCAATGGGAGGGATGCTGTGACTAACTTTGATAGTAGCTCCTACAATGGAGATGCTACACCTGACGTCGAAAATGAGGCAATTGTTGATGCTGATGCTCTTGACTTGGATCTAAGGATGTCGCAACCTACCGCGCACGATCCCAAGAGGGACAACATCATCGCCGGCCTTCAGTTAACTTTTGATTCCCCTGAGTCGTCAACCACAATGGTCTCCTCTCAGCCAATGAGCTCATCGTCCCAGTGGCCTGTGCATCAACATGGCACAGCAGTACCACCTCAGCAGCACCAGCGTTTGTACCCATCTGCTTGTCATGGCTTCTACCCCAACGTACAGGTGCAGGTGCAGGAGAGGCCCTTGGAGCCAAGGCCCCCTGAGCCGTCCTCCTTCCCCGGCTGGGGGTGGCACGCGCAGGCCGTGCCGCCGGGCTCCTCCCACTCGTTGTTGCTTTATGCTGCAGCATCATCAGGATTCTCTACCGCCGCCGGCGCGAACCCCGCCCCGCCGCCGTCGTACCCCGACCATCACCACCGCTTCTACTTCCCCCGCCCGCCGGACAACTAG

>DNA

CCAACCGGCCCGGCACGTACCTCACCTGCGCCTTTGGCCCTCCCCTTGGTGTCTGTGTAGGTGTAGCAGCAGCCCCGGCCGTGGTCAAGGGGGAGAAGACGCTACTACACACGAGGCCTCCCAGATCGAGCGCGGCATGCATGTATGCATGTGCCGCCGGAGCAAGCGCCGGTACGTATGTACGTATATATAATAGCGGCGCCGATTAATCTAGGGTTTGATTAGACTGAGGGGGGCGTGCAGTGCACTTGGCTAGTGTAATGCGGTCCTGCGAGGAGGGATCCAATCTTAATCTTAGCTCGTACGGCCGATGGCTTATCTTAAGCTCGCTAGCTAGGTGGCTAGATAACAACAACAACATGAGTTGATGGTGCCTCGCGTTAACAATTGCAACGAAAAGCCAACTGCCTGGTGCACACACCGTGGACGGATGCGTGGTGGATGGACACCATCATATACCATGGAGCATGGGTGGTTTTTTTTTTCCTGCTTCCTCTTAAATTAGTTATATGAGCAAAGGGCTTTTGAGTCTTGGTCCATACATGTCGGTCAAATCAAATCTTAAGACAAAAAGTTGCAGCATCATTTGTTCTAGAATGAAGTTGAAGTAAATTATGTGCTGCTCTTACGAGTGTTTATGTATTACTACTTTTTTCTTTAGGATGTACTGCGAGATTTTTTTAATACGTTGTTGCTTAATGTTTGGTTAGATCGATGACACGGTGTCATAAAATAATGCCATACCATCGGAGATCTGTTGCAAAGGCACATTGACATATGTAATTGCACTCTTCGTTGCATTAAAAATATGTTTATGCAGTAATCACGTATTTTTTATAGTTGCATCTTTAAAAACATCCAGAGTCTTCGACGTAAGACCTTTCTAGCTGCAGGCCTGATCCGCATGAGATGCTGCTTGGTTGTTGTATATGAAATACAATATTATAATGCCCTGTCCTTCATAAATTAAATTACTTACATTAGCAAGTTGACATTTATTTATCTTTAGCTTTGAAAACATCCAACGCCTTTGGTGTAAGACCTTTCTAGTCGTGCATCAAATTCAGTCAAGATACTACTTGACTGTTTATAAAATGATAGAATTATAATACATTTTCTACATCAATTAATTATTCAGCATGTCGATATTTTATATATTACTTAGCGTTCGCATCCGATACAATACAAAACTGTATGTCGACATAAAAATAAGTTTCAGTCATTTAACAAGAGGTATATAACACATGATTTGGTACCAAATTCGAGCTCTTGGTCGATCAAACTAGCGGTCTATGGTGGTGTTTTTTTACCCATGGACTAGACTAAAAAGTCCCTTGTAGTCCCTAGGTAAAGAAACAGGAGGGACTTTTCCTTAAAGAACTAGGAAAAGACTTTATTGAAGGGACTTTTTTCTTTAGTCCCTGGGACAAAAAAAAATCTAGTCCCTTGAAAAGAAACATCACCTATATAAGATCATTTACCTATATTTTTCAGAAAAAGGGATTGTGGAGAGGAAGCCGGCCAAAAAACCCACGGGGAACCTCTGTTTGATGTTTGATCCCCTCCGAGAGCGACACGTGGCGTCCGCACCCGGGGCGTGGGTGGCCGTAGCGATTAGCTAGCAGCGAAGCTCTGCTGCAGGAAAATACGTAGTTTACCTCCCTCTGATGATGCGCTTGGGGTTGGGCTTGGCCACCTCGACGTCCCATCTCGCTTTCTTTCTCTTTCTCTTTCCCTTTCTCCCACTGGCGCAGGGCCCTCTCCTCGTCCTCCTCCAGTCCTCATCCCCATGGCCACCACCACCGCCTCCACCCCCCACCACTACTTCTACTCCTCCCGCCTCGCCCTCGCAGCCCGCGGCCACCGCGCTCCCATGCCATAGACGCCAGCCAGCTGATCGGTCGACCTCGCTCGGACGCGGCACCGCGGCGAGCGAGGCGGGCATACCTGCGTTTCATTTGCGAGCTCCCGGCGCGCGGGCCTCGGATCGGAGGACGGAGATGGTGCTGGATCTCAATGTCGAGTCTCCGGCCGACTCCGGCACGTCCAGCTCGTCCGTGCTCAACTCCGCGGACGCCGCCGGCGCCTTCCGGTTCGGCCTGCTCGGGAGCCTCGATGACGACGACTGCTCCGGCGAGCTGGCGCCGGCCGCCGCGTCCGGGTTCGTGACGAGGCAGCTGTTCCCCGCGCCGCCGCCCGCGCCCGGGGTCATGATGGGGCAGGCGCCGGCGCCTCCGCCCACGGCGCCGGTGTGGCAACCTCGGCGCGCCGAGGAGCTGGTCGTGGCGCAGCGGGTGGCCCCCAAGAAGAAGACGCGGCGGGGGCCGAGGTCGCGCAGCTCGCAGTACCGGGGCGTCACCTTCTACCGCAGGACCGGCCGCTGGGAGTCGCACATCTGGTTAGCCATCTCTTCTCGCCATGCCTCTCTACTTACCAAAAGAGTGAACCATTCATTAACTAATCAATCGCTGATTACATCTGGGATTGCGGGAAGCAGGTCTACTTGGGTGAGCTCAATCAAATCCCAGCTCCAGCTCCCATCTTCCCCGTGTTTAATTTCGATTGTCTTTGCTATTATTATCATCTTCTGCTAATTAAAGAAGTTGGTTGATTTCTTCAGGTGGTTTCGACACCGCGCACGCGGCCGCAAGGTGAATAAATCATCATAAATTAATCAGGCTACGCTTGAACTCCTTTTACCCCGATTTGAGATTTGACCATGAACTGTGTACTGAGATGAGACGGTGTGGCGTGGCGCAGGGCCTACGATCGCGCGGCGATCAAGTTCCGGGGGCTGGAGGCCGACATCAACTTCAATCTGAGCGACTACGAGGAGGATTTGAAGCAGGTAATCTTATGAAAGCCAAGCTAGATTGATTGCTGTGCCACCCAGTAGTGCGCCGGATGATGATAATATATGGGTGGTGTTGTCCGATGGTTGATATCCGGTGGGTGGGTGGTGTTTTTTGCCAGATGAGGAACTGGACCAAGGAGGAGTTCGTGCATATCCTCCGCCGCCAGAGCACCGGGTTCGCCAGGGGGAGCTCGAAGTACCGCGGCGTCACGCTCCACAAGTGCGGCCGCTGGGAGGCAAGGATGGGCCAACTGCTCGGCAAGAAGTAAGCAGACTACACACACGCTAAAATTATATTAACTTCATTCACATTATCATAATTTCCTTAATCAATGCCATATACTGCAAGATTAGGCTGAAATGAAATTTCATAAACTGTTCATGAACTTGACACTCATTAGTAGGGTATAGATGTGACAGGGCAGCTGTTCATGCTGTGGACGTTTAATTATCATGTCGTAGGTAATCAATCTTAGATTATCTGTTTTGATTGAAACATAATCTTAGCTGGTTTAGGGTAGGGTCATCAAGCTAATCCATGTTGTTAGCTGTTGGCGCGTTCCTGTGCTGGTGCTGGTGAGACGTCCACTTCCCCCAACACTCTTATCGCAGACATCTATTTGGAGCAACTGTTATAGGTTCCACATATATGATGGAGTTGGCAAAACATGATTAATGGAATCCCTGCACACTATAGTTCAGACCCAGAATTGTCAAAGTGATCAATGTGTAGAAGTGATCTCACAGCTGATATATCTACATTACCATGACACACAGGTACATATATCTTGGGCTGTTCGACAGCGAAGTTGAAGCTGCAAGGTGCTTTGATTCGAGCTGATCACTAACATGCGGGTTCACAAAACTTCCTTAATTTTCGCTCACTCACTCTCTTGTCATGTTGGGATTTGGTAGAGCGTACGACAGGGCGGCGATTCGCTTCAATGGGAGGGATGCTGTGACTAACTTTGATAGTAGCTCCTACAATGGAGATGCTACACCTGACGTCGAAAATGAGGGTACTACTACAATCAGTCTCACCCGCTGAAATTTTCCAATCACACCCAACCTTTATCTCATTGTTATCGTTGTTTTCTCAGCAATTGTTGATGCTGATGCTCTTGACTTGGATCTAAGGATGTCGCAACCTACCGCGCACGATCCCAAGAGGGACAACATCATCGCCGGCCTTCAGTTAACTTTTGATTCCCCTGAGTCGTCAACCACAATGGTCTCCTCTCAGGTAAAGAAATTATTATGTTCCGTACTAGCTAATCCAGTGGTTTGATTGCTCCAAAAGATAATTTCCTGTGGCCCAGTTGGTGTCGGTCGATGAGATATTATTGATCCAGGTCCTATGGCGTATCCTCCTAAACTTACGATGTTTGTGCCTTTTGATATCGCTGTAGCCAATGAGCTCATCGTCCCAGTGGCCTGTGCATCAACATGGCACAGCAGTACCACCTCAGCAGCACCAGCGTTTGTACCCATCTGCTTGTCATGGCTTCTACCCCAACGTACAGGTATCATCACTGCGAGAACGACCCCCCTCCCCCCTCCTCCTCCTCTGTTTGGCGCGCTGCTCCACCTGCTTACTGAAACTGTTGCTGCTGAATAATGTCTCAGAAGAAACTGTTGATAGCATTTCAGTTTTTGTCAATTTCATCCCTGAAACTAAGGTTTCCATGTTTCTTTCCTATATCCAATCATCCTAAAACATCTACAAGTAGCATTTTAGGTGTGGTCATGTGTCTACAGACTGAGCTACTTTGCTTTCTTGAAACGCAAACTTTGATCATTGGCGTCTCTCGATCTCATGGACAGAGAGCATGCTGTGTCGTCGTACCAGAGTACTTACTAGATGTGGGTGCTACTGTTTGCCCTGTGAGCTAGTAGATAGATGATTGATGGTTCAATCTGGGTGGATCCGTGAGCGAGCTTGCACCAGCTTTTGAAAAAACTTGCAGTGCGTCGTCTAGTTCTTACAGTTCCATCCATCCATGACACAGCTTTAGATGCAGCTGCTGCTGAGTAGGAGTAGTACCCTGATCACATGGCCCAGCTTTATTCTTGGTATATACTGTGCATTCACATGAAAGCAATGCTTTGCTGGATGCACGGCCATGACTTGACACTCTCTCTCGGGTTGCAGGTGCAGGTGCAGGAGAGGCCCTTGGAGCCAAGGCCCCCTGAGCCGTCCTCCTTCCCCGGCTGGGGGTGGCACGCGCAGGCCGTGCCGCCGGGCTCCTCCCACTCGTTGTTGCTTTATGCTGCAGCATCATCAGGATTCTCTACCGCCGCCGGCGCGAACCCCGCCCCGCCGCCGTCGTACCCCGACCATCACCACCGCTTCTACTTCCCCCGCCCGCCGGACAACTAGCTCTCTGCTTACTCCCATGGACGGTGGTCGACGGGTGTGGGCGTGTGCGCGGGCGCGATCGACGTGTTCGCGCCTTGTCGGTAACAGTTGTTGTGAATTAATCGGAGAGAGATAACATTGCCGAGCCATGTGTCACTGGCTGGTTCCTCTCCCTCGCCATGATCAGAATCACAGGCATCATGGTTTCCCGTTCAGACTATGTATCCATGCTTTCAATCATGATCATGATCATGCGGTTGTCCATTACTAGATTCTCATGTATCCATGTTCAAGTTTGTGAAACAGCTGAAAAACCTTGGAAATTAATGGCGGCAGGTTCATGCG

>HvAP2-15

>Protein

MVSLRRRRLLGLCSGKDSLPVDLPKPVENEKNGEVEHANVNPLSVHPLPLTRTSDVLPESSNGSDSLKEEKNQYYPGKEIKRRKRHRRKQYVDQEPCIMRGVYFKNMKWQAAIKVDKKQIHLGTVGTQDEAARLYDRAAFMCGREPNFELSEEEKNELVKYTWDDFLAMTRNTITSKKQRKVGSLRHNKADLFIGDTEMVNGGGSSNSDDGDVDTSVS

>cDNA

ATGGTTAGCTTGAGGAGACGTCGACTATTGGGTCTTTGTTCTGGCAAAGATTCATTGCCAGTTGATCTTCCTAAGCCTGTTGAGAATGAAAAAAATGGGGAAGTTGAACACGCAAATGTCAATCCGCTCAGTGTGCACCCACTGCCCTTGACCAGGACTTCTGACGTACTTCCAGAATCCTCAAATGGTTCCGACTCTCTGAAGGAAGAGAAAAACCAATACTATCCAGGTAAGGAGATTAAGCGCAGAAAGCGACATAGAAGAAAGCAGTATGTGGACCAAGAGCCATGCATAATGAGAGGGGTCTATTTCAAAAATATGAAATGGCAAGCTGCTATAAAAGTTGACAAGAAACAAATTCACTTGGGTACTGTTGGAACACAGGATGAGGCAGCCCGGCTATATGATAGGGCTGCTTTTATGTGTGGAAGAGAGCCCAATTTTGAACTTTCTGAGGAAGAGAAAAACGAACTGGTTAAGTACACATGGGACGATTTCCTGGCAATGACACGCAACACCATAACCAGCAAAAAACAAAGAAAGGTTGGGTCGCTAAGGCATAATAAAGCAGACTTATTCATAGGAGATACTGAGATGGTCAATGGTGGCGGGTCTTCGAACTCAGATGATGGAGATGTTGACACATCGGTATCCTAGATCGCCGCCAGGACATGGACACCCAAATGGAGTTGTCCCACAGATTCATTCCGATATAATTTACCTCTAGTTTTTCCCCCCTCTCAGTATTTAAATTCATGTGCATAAGGCCCTGGTGGTCTGATAAAAGGTTAGATAACCATGACATCTCAGTTCAAATTGGAGATGGTCAGTTATAGGAACAAATTAAATTGAAGATGGTCAATTATAGAAATGAATTTAGCCGAGCAATCATTTGAAACGATTCCAGAACTGTGCATATCTGTTTATAGGTTTACATCAACATGTAACTAGTTTAACACAAGTTTTACCTATAGAATAATTTTCATTCCTGACA

>CDS

ATGGTTAGCTTGAGGAGACGTCGACTATTGGGTCTTTGTTCTGGCAAAGATTCATTGCCAGTTGATCTTCCTAAGCCTGTTGAGAATGAAAAAAATGGGGAAGTTGAACACGCAAATGTCAATCCGCTCAGTGTGCACCCACTGCCCTTGACCAGGACTTCTGACGTACTTCCAGAATCCTCAAATGGTTCCGACTCTCTGAAGGAAGAGAAAAACCAATACTATCCAGGTAAGGAGATTAAGCGCAGAAAGCGACATAGAAGAAAGCAGTATGTGGACCAAGAGCCATGCATAATGAGAGGGGTCTATTTCAAAAATATGAAATGGCAAGCTGCTATAAAAGTTGACAAGAAACAAATTCACTTGGGTACTGTTGGAACACAGGATGAGGCAGCCCGGCTATATGATAGGGCTGCTTTTATGTGTGGAAGAGAGCCCAATTTTGAACTTTCTGAGGAAGAGAAAAACGAACTGGTTAAGTACACATGGGACGATTTCCTGGCAATGACACGCAACACCATAACCAGCAAAAAACAAAGAAAGGTTGGGTCGCTAAGGCATAATAAAGCAGACTTATTCATAGGAGATACTGAGATGGTCAATGGTGGCGGGTCTTCGAACTCAGATGATGGAGATGTTGACACATCGGTATCCTAG

>DNA

GGGGGGGGGGGGGGGGGTTAGTGTTGTAAGTATATTTCCCGTGTAATATCCCCGTTATATATATTGGCCCATGGCCACCTGGAAATACAAGTTGCATATTTCTTAATAAAGTGCATCCTCCTTACCTGCTTGATCACCATTCTGCGGTTTCTTATCTTATCATATTAGACTTTTGACATTTTTCATGAGCAGGACAGTCATTATCCCCACAGTATTTTTACGTTTTATTACCTTCGGCCCAAATTACTTGTCTTAGATTTGTCTAGATACGGATGTATCTAACAATAAAACGTGTCTAGGTACATCCGTATGTAGACAAATCTATGACAAGTAATTCGGGATGGAGGGAATATATTAGTATTGCCCATAGCTTGAACATCACTGCCCTGGGTGGTATCCGAAGCAATGAGCTAGTCTAGTTTAGTACTTCTGGCATAGTCTATACTCTATACTACTAAATTCAAAAGGTTAACAATATATTTTAGGATAACCATTTACAAAACAATATACTCCCTCGGTTCCTAAATGAAAGTCCTTTTAGAGATTTCAATATGGATTACATACAAAGCAAAGTGGGTGAATCTACACTCTAAACATGTCTATATACATCTAAAAAGGCTTATACCTAGGAAGGGAGGGAGTACCTGCAAGTAATATGTATAGTTGTGTAATGATTAAACAAAGTTATTCATATATATAATGGTTAGACAGTACTAATATGGTATGGTTAGCTTGAGGAGACGTCGACTATTGGGTCTTTGTTCTGGCAAAGATTCATTGCCAGTTGATCTTCCTAAGCCTGTTGAGAATGAAAAAAATGGGGAAGTTGAACACGCAAATGTCAATCCGCTCAGTGTGCACCCACTGCCCTTGACCAGGACTTCTGACGTACTTCCAGAATCCTCAAATGGTTCCGACTCTCTGAAGGAAGAGAAAAACCAATACTATCCAGGTAAGGAGATTAAGCGCAGAAAGCGACATAGAAGAAAGCAGTATGTGGACCAAGAGCCATGCATAATGAGAGGGGTCTATTTCAAAAATATGAAATGGCAAGCTGCTATAAAAGTTGACAAGAAACAAATTCACTTGGGTACTGTTGGAACACAGGATGAGGCAGCCCGGCTATATGATAGGGCTGCTTTTATGTGTGGAAGAGAGCCCAATTTTGAACTTTCTGAGGAAGAGAAAAACGAACTGGTTAAGTACACATGGGACGATTTCCTGGCAATGACACGCAACACCATAACCAGCAAAAGTGAGACTTGTCGTGAAGTATTTTGTTAGTCATCACTTGGCAAAAATGACTGCAAGAGCATAGCATAATTTGTGTTTGTTTCCTTGTCTCCAGAACAAAGAAAGGTTGGGTCGCTAAGGCATAATAAAGCAGACTTATTCATAGGAGATACTGAGATGGTCAATGGTGGCGGGTCTTCGAACTCAGATGATGGAGATGTTGACACATCGGTATCCTAGATCGCCGCCAGGACATGGACACCCAAATGGAGTTGTCCCACAGATTCATTCCGATATAATTTACCTCTAGTTTTTCCCCCCTCTCAGTATTTAAATTCATGTGCATAAGGCCCTGGTGGTCTGATAAAAGGTTAGATAACCATGACATCTCAGTTCAAATTGGAGATGGTCAGTTATAGGAACAAATTAAATTGAAGATGGTCAATTATAGAAATGAATTTAGCCGAGCAATCATTTGAAACGATTCCAGAACTGTGCATATCTGTTTATAGGTTTACATCAACATGTAACTAGTTTAACACAAGTTTTACCTATAGAATAATTTTCATTCCTGACA

>HvAP2-16

>Protein

MCGKVPLANSKISAVTRNVMEVTVLPEKNKPVWGSRGDRCFTGHGRRKGLRMDNDEEDFEAGFGDSNMELVRGGVAQKDDGNESLSQDDLSIMPTADFDGPSEMPKRRKRKNQFQGIRQRPLGKWAAEITHPTKDLHVWPDTFNSAEEAARGYDAEARRIHGKKAKVNFTEKATRSTDFASNQPLVPAMNSTAPVEAPVMDMYSDQGSNSFGSSDLGWEYDAKTPDISSIAPTSTIAEGEEFALVKNNTYNSMVPHVMENNGVNLQPWMRYLLDDSVDKMIDSLLNFDVPQDTIVNMDLWSFDDMPTGGEFF

>cDNA

CTTCGCTGGTGCGTGGGCCCCATCACATGCCCTCTCACATCATCACACGGCCACCGTGTATATAAGGAGTGGCCTCACACCCATTTGCCGACCCACACCTTCCGTCCTGCTTAATTAAAACCTTCGCCCATTTACCGATTCCAGTGAGGCCTCTCCCCCCGACGACGACTTCAACGAGGTACGTACCGGCGGCCATCATGTGTGGTAAAGTGCCCCTTGCGAACAGCAAGATTTCTGCGGTAACGCGGAACGTGATGGAGGTGACGGTGTTGCCCGAGAAGAACAAGCCCGTGTGGGGCAGCCGCGGGGACCGATGCTTCACTGGGCATGGCCGGCGCAAGGGACTCAGGATGGACAACGACGAGGAGGACTTCGAGGCTGGCTTCGGGGACTCTAACATGGAGCTGGTGCGCGGTGGGGTGGCTCAGAAGGATGACGGCAACGAATCCCTCTCCCAAGATGACTTAAGCATCATGCCTACTGCTGATTTTGATGGTCCTTCAGAAATGCCAAAAAGAAGGAAGAGAAAAAACCAATTCCAGGGTATCCGTCAACGCCCACTGGGTAAGTGGGCTGCTGAAATCACACATCCTACCAAGGATCTCCATGTCTGGCCTGATACTTTCAATAGTGCTGAAGAAGCTGCAAGAGGTTATGATGCTGAAGCACGCAGGATACATGGAAAGAAGGCCAAGGTTAACTTTACAGAGAAAGCAACAAGATCAACTGACTTCGCATCAAACCAACCACTTGTTCCCGCAATGAACTCTACTGCCCCTGTTGAAGCTCCTGTTATGGATATGTACTCTGACCAGGGAAGCAACTCTTTTGGCTCCTCCGACTTGGGCTGGGAGTATGACGCCAAAACTCCAGATATATCATCCATTGCTCCCACTTCTACCATTGCTGAAGGAGAAGAATTTGCGCTCGTCAAGAACAATACCTACAACTCTATGGTTCCTCATGTTATGGAGAATAATGGTGTCAATTTACAACCTTGGATGAGATATCTTCTGGATGACAGCGTGGATAAGATGATTGATAGCCTTCTGAATTTTGACGTGCCCCAGGATACCATTGTCAACATGGACCTTTGGAGCTTCGATGACATGCCCACCGGTGGTGAATTTTTCTGAGGGATTCAAACCTTGTATACAGGGACAAAGAGAATAAAACTACGGGAGATCGAGAAGCGCCCTATTGTCACCTTCGGCTAGTGTGCTCATGTCCAAGCTTAGATGCAAAAAATAGTTGCATCCCGTGTCTCTTTTATAATCGAACCTTTCCCTAGTTGACTGTCTGTGTGGAAGGCATTTATGCTGAACAATTGTCTTTACGTTTTGTGAACTTTTATGTCGTCGTTACCATTTGTGAATGTGAACAACATAGCACCTCGGTGCCTGGGCACCTAGCTGGTTCTTTATTAATGCATCATGGATTCAAACCCAT

>CDS

ATGTGTGGTAAAGTGCCCCTTGCGAACAGCAAGATTTCTGCGGTAACGCGGAACGTGATGGAGGTGACGGTGTTGCCCGAGAAGAACAAGCCCGTGTGGGGCAGCCGCGGGGACCGATGCTTCACTGGGCATGGCCGGCGCAAGGGACTCAGGATGGACAACGACGAGGAGGACTTCGAGGCTGGCTTCGGGGACTCTAACATGGAGCTGGTGCGCGGTGGGGTGGCTCAGAAGGATGACGGCAACGAATCCCTCTCCCAAGATGACTTAAGCATCATGCCTACTGCTGATTTTGATGGTCCTTCAGAAATGCCAAAAAGAAGGAAGAGAAAAAACCAATTCCAGGGTATCCGTCAACGCCCACTGGGTAAGTGGGCTGCTGAAATCACACATCCTACCAAGGATCTCCATGTCTGGCCTGATACTTTCAATAGTGCTGAAGAAGCTGCAAGAGGTTATGATGCTGAAGCACGCAGGATACATGGAAAGAAGGCCAAGGTTAACTTTACAGAGAAAGCAACAAGATCAACTGACTTCGCATCAAACCAACCACTTGTTCCCGCAATGAACTCTACTGCCCCTGTTGAAGCTCCTGTTATGGATATGTACTCTGACCAGGGAAGCAACTCTTTTGGCTCCTCCGACTTGGGCTGGGAGTATGACGCCAAAACTCCAGATATATCATCCATTGCTCCCACTTCTACCATTGCTGAAGGAGAAGAATTTGCGCTCGTCAAGAACAATACCTACAACTCTATGGTTCCTCATGTTATGGAGAATAATGGTGTCAATTTACAACCTTGGATGAGATATCTTCTGGATGACAGCGTGGATAAGATGATTGATAGCCTTCTGAATTTTGACGTGCCCCAGGATACCATTGTCAACATGGACCTTTGGAGCTTCGATGACATGCCCACCGGTGGTGAATTTTTCTGA

>DNA

CTCCCTTGGCGTTATAGGAAGCACCATACCATATAAGTTTCCACGCAAGAGACGTTTTCTATTTTCACGGGGCTTACGCTCTTATTTGCCTTGTTTCATGCAGTTTACGATCGTGCGTTGCCATGTTACTTCTTCTCAGTGTAAGTAAGAGACACAAAGTCACGAATAACATTGCCATCTTAGTTTACATTTTCTCTATATACATGGTTAGATCATGAAAACATTATAATTTTTTTAATGTTCATCTGAATTCATAAACTAACCGTATAATAATGGTAATAATTGCACATACTTGATAGTATGTCAATTGGGGAAAGTAGATATGTATGATTTACAAGGATCATAAGAGTTGCATAGTCATTTATAAGGGGATATTGGCTGAATATTAAGTAAGATACAAGGTCCATGTCATGTTTCTTTTTTAAATAGATGTTAGATCTCCATATACATGGTTAGATCAAGAGTGTGATGAGAAAGTTAGAATGCTAACTTATAATTCATATAATTATTGTATAATAATGTGAAGGATTGCATATAAAGTACATGCTAGGAACTCAATGGAGTAGTTGTATTTTTATTTGGGAACAACGGAGTAAATATCTACGAATGACTTTACAAAAATCCTAGAGTTACGTACAATCATTTATGAGAAAAAAAATTTATCAAACTTTCGGTTGGATGAACGTGTCTTATGACAACAATAGAGACATACTTACAGGTGTGTAGTGCAATCTTGATCTAATAAATGGCATAAACTAGCTATGCGTGATTTTCAAAGATTTTAACATTACACACATCATTTATGGGAAGATTTTTGGTTAAATATTTGGTTGGATGAACCTGTTCAATAAACGAGTACAGATAGAGTATACGGGAGTAGTACTACAGTCAGACTATAGGTGTGTAGTACAATCTTGAGCTAATAAATGTTATAATTAGCTATGAGTGATTTGCAAAGATCTTAGCATTACACATAATCATTTATGAGAGAATTTTTCATAAATGCTCGGTTGGATGAGCCCGTCCAAGAAACCCGACGAAGAGACTACACCAAGAGTAGGCATACTACATGCGTGCAGTATAATCTTGAGATAATTAATGATATAACTAGCTATGAGTGATTTGAAAAGATCCTAGCATTACATACAATCATTTATGAAAAGATTTTTGGTTAAAAATTTGGTAGGATGAAACCGTCCAATAAACTCGAACTCAGAGTGTACAGCGGTAGTACTACAATTATACTACATTGCATGGTACAGTCTTTAACTAATAAATGAAGTAACTAGCTATGATTTATTTGCAAAGATCTTAAAACACATACAATAATTTATGAGAAGATATTGGTCAAACATTTGGTTGGATGAACTCGTCCAATACAATTGAACCGAGAAGTACAACCGTAGTAGTATAGTCATACTAAAGTTTTCTAGTATAATCTTAAGCTAATAGTTGTCCTAACTAGGTGTGAGTGATTCGCAATGATCTTAGCATTTCACACAATCATTTATGAGAAGATTTTTTGCTTAAACGTTCGGTTGGATGAACCTATCCAATAAACCCGATCAAAGAGTGTATGACATTAGTAGTAGACTCGTACTACATATGAGTAGTACAAAAATCTTGAGCGATAAATGTTGGCTTGCCTTCAAAGAAAGAAAGTCAAACATACATCCATCCATTAATTCTCAAGGGGAAATATAAGAAATAATACTTCCCCCGAGGGACAAGAGCGTAAGATTCCCCTGAGTAGAAATGCTTGCTTTCAGAACCTGCCTCGTGCTTTCCTCCAATAGTGGGGCCCTTCGCTGGTGCGTGGGCCCCATCACATGCCCTCTCACATCATCACACGGCCACCGTGTATATAAGGAGTGGCCTCACACCCATTTGCCGACCCACACCTTCCGTCCTGCTTAATTAAAACCTTCGCCCATTTACCGATTCCAGTGAGGCCTCTCCCCCCGACGACGACTTCAACGAGGTACGTACCGGCGGCCATCATGTGTGGTAAAGTGCCCCTTGCGAACAGCAAGATTTCTGCGGTAACGCGGAACGTGATGGAGGTGACGGTGTTGCCCGAGAAGAACAAGCCCGTGTGGGGCAGCCGCGGGGACCGATGCTTCACTGGGCATGGCCGGCGCAAGGGACTCAGGATGGACAACGACGAGGAGGACTTCGAGGCTGGCTTCGGGGACTCTAACATGGAGCTGGTGCGCGGTGGGGTGGCTCAGAAGGATGACGGCAACGAATCCCTCTCCCAAGGTATTGGCCCTGGGTAGCTTGCACTAGTTTTTAATAGATCCCTCTCCCAAGGTATCGGCCCGGGTAGCTTGCACTAGTTTTTAATCTGGGTATCACTACCTTTAGCGGTTAACTGTTGTATTAATACAGTCTTGGTTTAGTAGTCTCCATCGTATTTTAGGTCAAAGTATGACCACATAATTAACTAGTAAAATTTTAATGCATGTCATGAAAAGTTATATTGTTGGATTATTTTTTGAGTGTAATTTTAATGTTATTTTCGCTACATTAACTTATATTTTATTATTTAGTCATGGTAAAAAAACGACCCAAAAATACAAGAAGGACTAGTCAACCATGATGGAGGTAGTAGAAGACAGGAGATTGTGTGAGTGGTCGAAAATGGTTTGGTCGTTCGATTGGGCAGGATGAGAAATTGTGCAGGGGTTGCTATTTGGAAGGCTATGTTTGGGTCGTGTTACTCCTGTTTGCTTAGAGATTTTGGATTGTATGTGCTTTCATGGAGTGATCTGTGTGAATGGTCAGGATTTTTCCATTTTTCCGTTGTGTTTGGTTGGTTGGCTATTTCCGCAAAGGTCTGGGCCTCTCTTGAATTGTTTCCTCACATGGTTGTAAGTGTGCGTGAGGATTGTTCTAGGTTGGTTAATTTATCAGTCTAAATGGTTATTCTGCTAAACATTGAATCCGACTTAAAAACATATCCTTGATTCTTCATCATTTATAATGCAATTTGTAGATTAATAATTGCAGAATAATGTCAAGGTTCTATAACATGATATATGTTTATCAGAACCTTGGATTTGTTTTTATTAACTTGTGATATTTATTTTATTTGATTTATTTTCTGGTGACTTATGCATTCGCTCGAACTTGACACATCATGGTTTAGTGTTCATTACAATCATTATATTTTACATAGAAATTTATGACATATTATTTACTATATGTGAGATAAATTTATTTGTGTTGGTCCCTCTTAGCATTGGCATTCATCTTCCATTGCAGATGACTTAAGCATCATGCCTACTGCTGATTTTGATGGTCCTTCAGAAATGCCAAAAAGAAGGAAGAGAAAAAACCAATTCCAGGGTATCCGTCAACGCCCACTGGGTAAGTGGGCTGCTGAAATCACACATCCTACCAAGGATCTCCATGTCTGGCCTGATACTTTCAATAGTGCTGAAGAAGCTGCAAGAGGTTATGATGCTGAAGCACGCAGGATACATGGAAAGAAGGCCAAGGTTAACTTTACAGAGAAAGCAACAAGATCAACTGACTTCGCATCAAACCAACCACTTGTTCCCGCAATGAACTCTACTGCCCCTGTTGAAGCTCCTGTTATGGATATGTACTCTGACCAGGGAAGCAACTCTTTTGGCTCCTCCGACTTGGGCTGGGAGTATGACGCCAAAACTCCAGATATATCATCCATTGCTCCCACTTCTACCATTGCTGAAGGAGAAGAATTTGCGCTCGTCAAGAACAATACCTACAACTCTATGGTTCCTCATGTTATGGAGAATAATGGTGTCAATTTACAACCTTGGATGAGATATCTTCTGGATGACAGCGTGGATAAGATGATTGATAGCCTTCTGAATTTTGACGTGCCCCAGGATACCATTGTCAACATGGACCTTTGGAGCTTCGATGACATGCCCACCGGTGGTGAATTTTTCTGAGGGATTCAAACCTTGTATACAGGGACAAAGAGTAAGCATTTGCAAAATTTCATGTTCAGTCTGAGGTTTACAGTCTTTTCATTACTCTGTTTATGTTGTTGTTTCAGGAATAAAACTACGGGAGATCGAGAAGCGCCCTATTGTCACCTTCGGCTAGTGTGCTCATGTCCAAGCTTAGATGCAAAAAATAGTTGCATCCCGTGTCTCTTTTATAATCGAACCTTTCCCTAGTTGACTGTCTGTGTGGAAGGCATTTATGCTGAACAATTGTCTTTACGTTTTGTGAACTTTTATGTCGTCGTTACCATTTGTGAATGTGAACAACATAGCACCTCGGTGCCTGGGCACCTAGCTGGTTCTTTATTAATGCATCATGGATTCAAACCCAT

>HvAP2-17

>Protein

MMVATAESVHDQGLKLQGGVDVVAVEHDGAAEAAGGRRKRAQAGSKGSTSEGAGRAYGAGKAAKKRAAPRPESWTEFRGVSRTHTGTYGARIRHSKGQTRWLGTFKAAEEAARAYDEAAVRLHGARAVTNYKQNGVSEPVGRAAAKKKPAAPRPDARTEFVGVSRQPNGKYVAGLWDSGRKQMVKVGRFDTAEEAAGAYDAAAVRVYGAAARTNFERKPTAVATDDGDESSVDLLSDLPELPAGDARSDSIMPGPTLDDLKTDADPTQAEWQQVDEFLIDMDFTDMVD

>cDNA

ATATTACACAACATCCTAAATCCTAATCATGCATTTCCTCTCAGATTCTTGCAATTCAAGAAAGAAAAGAAAAAACTCTAGGTAACCTGACAGCTGTATTGGCTGCCAGCCAGTACAGTGTAGAGCAACTGCAACACGAAATCTTGCAGGCTGCACGAATCACGAACAGCAGATGAAGAAACCAGATCCTGTACGCCCCCCGTCCCTCTAAACCCCACCCAAAAGCTCGCGCATTTCCGCCCCCCCCCCCCCCCCCCTGCCAGTCCAGTGTGATGATGGTGGCGACGGCGGAGAGCGTCCATGATCAAGGCCTGAAGCTGCAGGGTGGCGTGGATGTGGTGGCGGTGGAGCACGACGGGGCCGCGGAAGCTGCGGGTGGGAGGAGGAAGAGGGCCCAGGCGGGGTCGAAGGGGAGCACCTCCGAGGGGGCCGGCAGAGCGTACGGCGCGGGGAAGGCGGCGAAGAAGAGGGCGGCGCCGCGGCCGGAGTCCTGGACCGAGTTCCGCGGCGTGTCTCGGACACATACCGGCACGTACGGGGCGCGGATCAGGCACTCCAAGGGGCAGACGCGGTGGCTCGGCACCTTCAAAGCCGCCGAGGAGGCCGCCAGGGCGTACGACGAGGCGGCCGTCAGGCTGCACGGCGCGCGGGCCGTGACCAACTACAAGCAAAACGGCGTCTCCGAGCCGGTCGGCCGAGCGGCGGCAAAGAAGAAGCCGGCGGCGCCTAGGCCTGACGCCCGGACCGAGTTCGTCGGCGTGTCTCGGCAGCCGAACGGCAAGTACGTTGCGGGGCTATGGGACTCGGGGAGGAAGCAGATGGTGAAGGTCGGAAGGTTCGACACCGCCGAGGAGGCCGCCGGAGCGTACGATGCGGCGGCTGTCCGGGTATACGGCGCCGCGGCCCGGACCAACTTCGAGCGGAAACCCACGGCAGTCGCTACTGATGACGGCGATGAGTCGTCCGTGGACCTCCTCAGCGACCTCCCGGAGCTGCCGGCCGGCGACGCCCGCTCGGACAGCATCATGCCAGGTCCGACGCTCGACGATCTCAAGACTGACGCTGACCCGACGCAGGCTGAGTGGCAGCAGGTGGACGAGTTCCTCATCGACATGGACTTCACCGACATGGTGGATTAGCAGCTGGTGGACGAGCGACTTCCTCAAATGCTCTGCTTTTCTGTTTGTCGGGCGTAACAAGGGTAACACGCGACGAATTAGGGACAATTTTGGTGGGGCGGACTATTGCACTTGCACAGGTGATCAATATCCTCCAATGTTTTTGTTTGCTAGGAAACATGGGCATATATTAAGCTTTTATGCCAGCTTTAGCGTTAGCACATCCATATGTGCAAGAGAAAGGTTCAGACAGAATTATGCTTGCTATCTCTCTACCAAAACAGAACTATGATTTCTACTAGCTTGATGTGGACATATGAATCTAGAGTTTTATGGGAAGCACAATTTTATGG

>CDS

ATGATGGTGGCGACGGCGGAGAGCGTCCATGATCAAGGCCTGAAGCTGCAGGGTGGCGTGGATGTGGTGGCGGTGGAGCACGACGGGGCCGCGGAAGCTGCGGGTGGGAGGAGGAAGAGGGCCCAGGCGGGGTCGAAGGGGAGCACCTCCGAGGGGGCCGGCAGAGCGTACGGCGCGGGGAAGGCGGCGAAGAAGAGGGCGGCGCCGCGGCCGGAGTCCTGGACCGAGTTCCGCGGCGTGTCTCGGACACATACCGGCACGTACGGGGCGCGGATCAGGCACTCCAAGGGGCAGACGCGGTGGCTCGGCACCTTCAAAGCCGCCGAGGAGGCCGCCAGGGCGTACGACGAGGCGGCCGTCAGGCTGCACGGCGCGCGGGCCGTGACCAACTACAAGCAAAACGGCGTCTCCGAGCCGGTCGGCCGAGCGGCGGCAAAGAAGAAGCCGGCGGCGCCTAGGCCTGACGCCCGGACCGAGTTCGTCGGCGTGTCTCGGCAGCCGAACGGCAAGTACGTTGCGGGGCTATGGGACTCGGGGAGGAAGCAGATGGTGAAGGTCGGAAGGTTCGACACCGCCGAGGAGGCCGCCGGAGCGTACGATGCGGCGGCTGTCCGGGTATACGGCGCCGCGGCCCGGACCAACTTCGAGCGGAAACCCACGGCAGTCGCTACTGATGACGGCGATGAGTCGTCCGTGGACCTCCTCAGCGACCTCCCGGAGCTGCCGGCCGGCGACGCCCGCTCGGACAGCATCATGCCAGGTCCGACGCTCGACGATCTCAAGACTGACGCTGACCCGACGCAGGCTGAGTGGCAGCAGGTGGACGAGTTCCTCATCGACATGGACTTCACCGACATGGTGGATTAG

>DNA

TCATTGGAACAGAGACTTGTGGCGGTAGAAAAAATGTTTCGGGTTGCTCTGTGATGTATTCCTAATATTTAAGAATTTATAGGGTGAAATTAGATCATACGGAGCCACAAGGGGCCTAGAAGGTAATAGGGTGTGCGTACCCCCTAGGCACGCCATGTCGCCTTATCGTCTCCGCGTGCAGCTTCTGGCCTCCTTCCGAAACTTCTAGGTCCTCTTTTTTCCAAAAAATAATCATCAAAACCTTTCGTAGTGTTGGACTCCATTTGATACTAGTTTCCTGAAAAGCTAAAAACATGCAGAAAACAACAAGTGGCACTAGGCACTCGGTTAATAGGTTACTTTCCAAAAATAATATAAAGCAACAAATCAATTCATATAAAGCATCTAAGATTCATAATATAATAGCATGGAACAATAAAACGTCATAGATACGTTGGAGACGTATCAGTTTACAACTTCATAGAGTGGCTAGATAACACTTTCTAAATCGGGGGAGTTCCCAAAAGTAGGCGTTGGTTAATCTTTTTGAGGGGAAATTTTCTCCCTCTTTTTCTCTCCCTTTTATTTGGTTTTCTTCCTTTACTCCTCCCTCATGTAACATACTTCCGTTTATACAATGAAAATATATCATAGAATTATTGTTTTACGGGTTGCAGGTTAAAACTTTCTCGATATTAATCTAAGATGCCCTTTGAGTGTCTTTATTAAAAGAAAATCAATCCATAGTTGAGGACACCTTTGAATTGAAGGAACTTCATAGAAATTTTGCAGGAGTCCAATCCATAAGAATATTTTATACGAAAACATTTGGATCAAAGGGATAGGTTCACCAAATTTCTAGGGAATCCATTCATATGCCTCGGTTCCATGGTAATCTCAACATGAGCTCATGATTCATGTGTTTGTTTCCTGAGTAACAACAAAAAGCAGCTGTCATATTCCTGCATTTCTTAAATTATGTGAAAATCCATATTACACAACATCCTAAATCCTAATCATGCATTTCCTCTCAGATTCTTGCAATTCAAGAAAGAAAAGAAAAAACTCTAGGTAACCTGACAGCTGTATTGGCTGCCAGCCAGTACAGTGTAGAGCAACTGCAACACGAAATCTTGCAGGCTGCACGAATCACGAACAGCAGATGAAGAAACCAGATCCTGTACGCCCCCCGTCCCTCTAAACCCCACCCAAAAGCTCGCGCATTTCCGCCCCCCCCCCCCCCCCCCTGCCAGTCCAGTGTGATGATGGTGGCGACGGCGGAGAGCGTCCATGATCAAGGCCTGAAGCTGCAGGGTGGCGTGGATGTGGTGGCGGTGGAGCACGACGGGGCCGCGGAAGCTGCGGGTGGGAGGAGGAAGAGGGCCCAGGCGGGGTCGAAGGGGAGCACCTCCGAGGGGGCCGGCAGAGCGTACGGCGCGGGGAAGGCGGCGAAGAAGAGGGCGGCGCCGCGGCCGGAGTCCTGGACCGAGTTCCGCGGCGTGTCTCGGACACATACCGGCACGTACGGGGCGCGGATCAGGCACTCCAAGGGGCAGACGCGGTGGCTCGGCACCTTCAAAGCCGCCGAGGAGGCCGCCAGGGCGTACGACGAGGCGGCCGTCAGGCTGCACGGCGCGCGGGCCGTGACCAACTACAAGCAAAACGGCGTCTCCGAGCCGGTCGGCCGAGCGGCGGCAAAGAAGAAGCCGGCGGCGCCTAGGCCTGACGCCCGGACCGAGTTCGTCGGCGTGTCTCGGCAGCCGAACGGCAAGTACGTTGCGGGGCTATGGGACTCGGGGAGGAAGCAGATGGTGAAGGTCGGAAGGTTCGACACCGCCGAGGAGGCCGCCGGAGCGTACGATGCGGCGGCTGTCCGGGTATACGGCGCCGCGGCCCGGACCAACTTCGAGCGGAAACCCACGGCAGTCGCTACTGATGACGGCGATGAGTCGTCCGTGGACCTCCTCAGCGACCTCCCGGAGCTGCCGGCCGGCGACGCCCGCTCGGACAGCATCATGCCAGGTCCGACGCTCGACGATCTCAAGACTGACGCTGACCCGACGCAGGCTGAGTGGCAGCAGGTGGACGAGTTCCTCATCGACATGGACTTCACCGACATGGTGGATTAGCAGCTGGTGGACGAGCGACTTCCTCAAATGCTCTGCTTTTCTGTTTGTCGGGCGTAACAAGGGTAACACGCGACGAATTAGGGACAATTTTGGTGGGGCGGACTATTGCACTTGCACAGGTGATCAATATCCTCCAATGTTTTTGTTTGCTAGGAAACATGGGCATATATTAAGCTTTTATGCCAGCTTTAGCGTTAGCACATCCATATGTGCAAGAGAAAGGTTCAGACAGAATTATGCTTGCTATCTCTCTACCAAAACAGAACTATGATTTCTACTAGCTTGATGTGGACATATGAATCTAGAGTTTTATGGGAAGCACAATTTTATGG

>HvAP2-18

>Protein

MDAKADGDSVALLRHVSCVGEDVGAVQSCRGRDAVESTKERKAPLRPVARTVFRRVSKKPTGKCVARIRCPKVGARRYLGGFNTAEEAARAYIASAVKLPGAVGLKKSRAAGEVSFKGQAGGKAAAAAKSRSRASSMPAFHGVRIWDPAQRAKLFLGAFDAAEEADGAFDAEAVKLRGAMAKTNLKRQPTVRKKAATRTDAGTKFRGVHRKPSGKYAAQNRHAGGNSRCLGPFNTAEDAVRAYDAAAVKLHGVKAITNFNNTPMAAAVDDGEESPMDLNDVPEMRGVRAKTNLNKPPLVAGSADDGEESRMDLAHSNFPELPALGLFSGSITADAQLDDMFADLPPLDLQQVGELLKDMDFANMMA

>cDNA

CGACGGGCCGCTCCGGCACGTCTCCTGCGTCGACATAGACGCCAAGGCCGATGACGACGGCGGCGACGCGCCGCTCCGGAACGTCTCCTGCGTCGACATGGACGCCAAGGCCGATGGCGACAGCGTCGCGTTGCTCCGGCATGTCTCCTGCGTGGGCGAGGATGTCGGGGCAGTCCAGTCGTGCCGAGGCCGTGATGCGGTGGAGAGCACCAAGGAGAGGAAGGCGCCTCTCAGGCCGGTCGCCCGGACCGTGTTCCGCCGCGTGAGCAAGAAGCCGACCGGCAAGTGTGTGGCGAGGATCAGGTGCCCTAAGGTGGGAGCTCGGAGGTACCTCGGCGGCTTCAACACCGCCGAGGAGGCCGCCCGAGCCTACATCGCGTCGGCGGTCAAGCTGCCCGGCGCGGTCGGCCTGAAGAAGTCACGCGCGGCGGGGGAGGTCAGCTTCAAAGGTCAGGCCGGAGGGAAGGCGGCGGCGGCGGCTAAGTCGAGGTCGAGGGCGAGCTCCATGCCAGCATTCCACGGCGTGCGCATCTGGGACCCGGCGCAGCGAGCGAAGCTGTTTCTCGGCGCCTTCGACGCGGCCGAGGAGGCGGACGGAGCGTTCGACGCGGAGGCCGTCAAGCTGCGTGGCGCCATGGCCAAAACCAACCTGAAGCGGCAGCCCACGGTGAGGAAGAAGGCGGCGACAAGGACGGACGCCGGGACCAAGTTCCGCGGCGTGCACCGGAAGCCCAGCGGCAAGTACGCGGCGCAGAACAGACACGCCGGGGGGAATTCTCGGTGCCTCGGTCCCTTCAACACCGCCGAGGACGCCGTCAGAGCGTACGATGCCGCGGCCGTCAAGCTGCATGGTGTCAAGGCCATAACCAACTTCAACAACACACCCATGGCTGCCGCCGTTGACGATGGCGAGGAGTCGCCCATGGACCTCAACGACGTCCCGGAGATGCGTGGCGTGAGGGCCAAAACCAACCTCAACAAACCACCCCTGGTCGCCGGCTCTGCTGATGACGGCGAGGAGTCGCGCATGGACCTCGCCCACAGCAACTTCCCGGAGCTGCCGGCGCTCGGCCTCTTCTCGGGCAGCATCACCGCAGACGCACAGCTCGACGATATGTTCGCCGACTTGCCGCCGTTGGATTTGCAGCAGGTGGGCGAGCTCCTCAAGGACATGGACTTCGCCAACATGATGGCATGACCCGTGCGTGTTCGTCCGGCGTAGAGCAGAACAGAGACGAAATGCCGCCGATTTTGTCAG

>CDS

ATGGACGCCAAGGCCGATGGCGACAGCGTCGCGTTGCTCCGGCATGTCTCCTGCGTGGGCGAGGATGTCGGGGCAGTCCAGTCGTGCCGAGGCCGTGATGCGGTGGAGAGCACCAAGGAGAGGAAGGCGCCTCTCAGGCCGGTCGCCCGGACCGTGTTCCGCCGCGTGAGCAAGAAGCCGACCGGCAAGTGTGTGGCGAGGATCAGGTGCCCTAAGGTGGGAGCTCGGAGGTACCTCGGCGGCTTCAACACCGCCGAGGAGGCCGCCCGAGCCTACATCGCGTCGGCGGTCAAGCTGCCCGGCGCGGTCGGCCTGAAGAAGTCACGCGCGGCGGGGGAGGTCAGCTTCAAAGGTCAGGCCGGAGGGAAGGCGGCGGCGGCGGCTAAGTCGAGGTCGAGGGCGAGCTCCATGCCAGCATTCCACGGCGTGCGCATCTGGGACCCGGCGCAGCGAGCGAAGCTGTTTCTCGGCGCCTTCGACGCGGCCGAGGAGGCGGACGGAGCGTTCGACGCGGAGGCCGTCAAGCTGCGTGGCGCCATGGCCAAAACCAACCTGAAGCGGCAGCCCACGGTGAGGAAGAAGGCGGCGACAAGGACGGACGCCGGGACCAAGTTCCGCGGCGTGCACCGGAAGCCCAGCGGCAAGTACGCGGCGCAGAACAGACACGCCGGGGGGAATTCTCGGTGCCTCGGTCCCTTCAACACCGCCGAGGACGCCGTCAGAGCGTACGATGCCGCGGCCGTCAAGCTGCATGGTGTCAAGGCCATAACCAACTTCAACAACACACCCATGGCTGCCGCCGTTGACGATGGCGAGGAGTCGCCCATGGACCTCAACGACGTCCCGGAGATGCGTGGCGTGAGGGCCAAAACCAACCTCAACAAACCACCCCTGGTCGCCGGCTCTGCTGATGACGGCGAGGAGTCGCGCATGGACCTCGCCCACAGCAACTTCCCGGAGCTGCCGGCGCTCGGCCTCTTCTCGGGCAGCATCACCGCAGACGCACAGCTCGACGATATGTTCGCCGACTTGCCGCCGTTGGATTTGCAGCAGGTGGGCGAGCTCCTCAAGGACATGGACTTCGCCAACATGATGGCATGA

>DNA

CGACATGGACGCCAAGGCCGATGGCGGCGACGGCGACGCGCCGCTCCGGCACGTCTCCTGCGTCGACATGGACGAGAAGGCCGATGGCGATGGCGGCGACGCGCCGCTCCGGAACGTCTCCTACGTCGACATGGACGCCAAGGCGTATGGCGACGGCGGCGACGGGCCGCTCCGGCACGTCTCCTGCGTCGACATAGACGCCAAGGCCGATGACGACGGCGGCGACGCGCCGCTCCGGAACGTCTCCTGCGTCGACATGGACGCCAAGGCCGATGGCGACAGCGTCGCGTTGCTCCGGCATGTCTCCTGCGTGGGCGAGGATGTCGGGGCAGTCCAGTCGTGCCGAGGCCGTGATGCGGTGGAGAGCACCAAGGAGAGGAAGGCGCCTCTCAGGCCGGTCGCCCGGACCGTGTTCCGCCGCGTGAGCAAGAAGCCGACCGGCAAGTGTGTGGCGAGGATCAGGTGCCCTAAGGTGGGAGCTCGGAGGTACCTCGGCGGCTTCAACACCGCCGAGGAGGCCGCCCGAGCCTACATCGCGTCGGCGGTCAAGCTGCCCGGCGCGGTCGGCCTGAAGAAGTCACGCGCGGCGGGGGAGGTCAGCTTCAAAGGTCAGGCCGGAGGGAAGGCGGCGGCGGCGGCTAAGTCGAGGTCGAGGGCGAGCTCCATGCCAGCATTCCACGGCGTGCGCATCTGGGACCCGGCGCAGCGAGCGAAGCTGTTTCTCGGCGCCTTCGACGCGGCCGAGGAGGCGGACGGAGCGTTCGACGCGGAGGCCGTCAAGCTGCGTGGCGCCATGGCCAAAACCAACCTGAAGCGGCAGCCCACGGTGAGGAAGAAGGCGGCGACAAGGACGGACGCCGGGACCAAGTTCCGCGGCGTGCACCGGAAGCCCAGCGGCAAGTACGCGGCGCAGAACAGACACGCCGGGGGGAATTCTCGGTGCCTCGGTCCCTTCAACACCGCCGAGGACGCCGTCAGAGCGTACGATGCCGCGGCCGTCAAGCTGCATGGTGTCAAGGCCATAACCAACTTCAACAACACACCCATGGCTGCCGCCGTTGACGATGGCGAGGAGTCGCCCATGGACCTCAACGACGTCCCGGAGATGCGTGGCGTGAGGGCCAAAACCAACCTCAACAAACCACCCCTGGTCGCCGGCTCTGCTGATGACGGCGAGGAGTCGCGCATGGACCTCGCCCACAGCAACTTCCCGGAGCTGCCGGCGCTCGGCCTCTTCTCGGGCAGCATCACCGCAGACGCACAGCTCGACGATATGTTCGCCGACTTGCCGCCGTTGGATTTGCAGCAGGTGGGCGAGCTCCTCAAGGACATGGACTTCGCCAACATGATGGCATGACCCGTGCGTGTTCGTCCGGCGTAGAGCAGAACAGAGACGAAATGCCGCCGATTTTGTCAG

>HvAP2-19

>Protein

MMLPSSRKVRISCCDPDATDSSDEDDRHAKKEKRMTMEVLVPMKSSQVLKSRKTLLPCGTMKSMGTEKKQPTSKYPGVRLRSWGKWAAEIRDPVSKTRKWIGTFTSEEAAAAAYEAERNRVRAEMLAIKSRPSPSEHEALSSEATVSCVSSSVSFGDQKAQEVHKVAPMEIDPDTADESLLHCSPELPGKEIEVDAFLGRMNVDESLVHCSSTPSDEEIPVDAFHSQINELPISDYVCTTDKLSLDDISRLADMFHVNDFVDTTGKPPGDDYIGLADISHLPLPMFELDVGLDWEGFDFASMERELEKL

>cDNA

AAGCCCCCGCCCGATCCGCCGTCTCATTCCCCTCACCGCTTCCCCCGGGGATCGCAGTGGCATCCGCGGCTCCAGCAACCAAAGTTGGAGTAGAGACCGCCAGCCAGCACGAGCGATCGCTCTTGATTTATGTCGCCTGCTCTGCTGCTCCATGGTTCTGAGCTCCCATCACCATCACCTCCTTCTGCTTTTTGCTCCCTTCTCCCACAAGCTCGGCAGAGGGGCGCCAGAACTCAGGATTATCGCTTCAAGATCTCCCGATTTGGATTTGGGGTGAAGTACTCTGGATTTCTGCATTTAGACCAGTGCCCCGTGCACTTGAAGTAATAACGATACAAGAATCCCCCAAATCACGATAAGCTTGGTTTGGTTTGGGTGGATTAAGTTTAGCACTCCTCCTTCATTCTCATTGACATTCCTCAAGTTCATAAACAATCAGCCAGAAAGCAGATTTCTTTTGTGAGTCTTCACACAAAGAGAATAACTCCGTCTTCTCTCCGGTTTGAATCAGAAGTTTGAGTCCATTCGGTTTCTGATTTTGGAGATTGTGTGCTCAGTCTCTAAACCAGGAGTTTGTCCTGGGATTTAATATTTCAGTCGATTGTAGTCAAGGTTAATAATTCAATAATCTCTGGATCTTACATAAGATTAAGGGGCTTGTTGCACAAAGAGATGATGTTGCCTTCTTCGCGGAAGGTCCGCATTTCCTGCTGTGATCCTGACGCCACCGATTCCTCTGATGAAGATGATCGGCATGCGAAGAAGGAAAAGAGAATGACGATGGAAGTACTAGTTCCAATGAAAAGCTCCCAAGTCCTCAAATCTCGAAAGACCCTCTTGCCATGCGGCACAATGAAATCAATGGGTACAGAGAAGAAGCAGCCAACCAGCAAGTACCCTGGTGTGCGCCTGCGGTCATGGGGCAAGTGGGCTGCGGAGATTCGTGATCCCGTGAGCAAGACCCGGAAATGGATTGGCACATTTACTTCTGAGGAGGCGGCAGCTGCAGCATATGAGGCAGAACGGAACCGGGTACGTGCTGAGATGTTGGCCATCAAATCTCGGCCATCTCCATCAGAACATGAAGCTTTGTCCAGCGAAGCTACTGTATCCTGTGTATCCTCTTCTGTGTCGTTTGGCGACCAGAAAGCACAAGAGGTACACAAGGTAGCGCCAATGGAGATAGACCCTGACACTGCTGATGAGAGCTTACTGCATTGCTCACCAGAACTGCCAGGTAAAGAAATTGAGGTAGATGCATTCCTTGGCCGGATGAATGTTGATGAAAGCTTAGTGCACTGCTCATCGACACCTAGCGATGAAGAAATTCCAGTGGATGCATTCCACAGCCAGATAAATGAGCTTCCTATCAGTGACTATGTTTGCACAACTGACAAACTCTCACTGGATGATATTTCAAGGCTGGCAGATATGTTTCATGTCAATGACTTCGTTGACACAACAGGCAAACCGCCTGGCGACGACTACATCGGGCTGGCAGACATCAGCCATCTACCGTTGCCAATGTTTGAGTTGGATGTAGGACTTGATTGGGAAGGTTTTGACTTCGCTTCGATGGAACGTGAACTAGAGAAACTTTGAGGCCTTACCAGCATTGAGGTTTTCATCTTTCTGACGGACGTCAGGGTGAGTTTGTGATCAGGAGATATGTTCATCTCCAATTTTGTCAGAAATTGTACAGTCCTCTCCC

>CDS

ATGATGTTGCCTTCTTCGCGGAAGGTCCGCATTTCCTGCTGTGATCCTGACGCCACCGATTCCTCTGATGAAGATGATCGGCATGCGAAGAAGGAAAAGAGAATGACGATGGAAGTACTAGTTCCAATGAAAAGCTCCCAAGTCCTCAAATCTCGAAAGACCCTCTTGCCATGCGGCACAATGAAATCAATGGGTACAGAGAAGAAGCAGCCAACCAGCAAGTACCCTGGTGTGCGCCTGCGGTCATGGGGCAAGTGGGCTGCGGAGATTCGTGATCCCGTGAGCAAGACCCGGAAATGGATTGGCACATTTACTTCTGAGGAGGCGGCAGCTGCAGCATATGAGGCAGAACGGAACCGGGTACGTGCTGAGATGTTGGCCATCAAATCTCGGCCATCTCCATCAGAACATGAAGCTTTGTCCAGCGAAGCTACTGTATCCTGTGTATCCTCTTCTGTGTCGTTTGGCGACCAGAAAGCACAAGAGGTACACAAGGTAGCGCCAATGGAGATAGACCCTGACACTGCTGATGAGAGCTTACTGCATTGCTCACCAGAACTGCCAGGTAAAGAAATTGAGGTAGATGCATTCCTTGGCCGGATGAATGTTGATGAAAGCTTAGTGCACTGCTCATCGACACCTAGCGATGAAGAAATTCCAGTGGATGCATTCCACAGCCAGATAAATGAGCTTCCTATCAGTGACTATGTTTGCACAACTGACAAACTCTCACTGGATGATATTTCAAGGCTGGCAGATATGTTTCATGTCAATGACTTCGTTGACACAACAGGCAAACCGCCTGGCGACGACTACATCGGGCTGGCAGACATCAGCCATCTACCGTTGCCAATGTTTGAGTTGGATGTAGGACTTGATTGGGAAGGTTTTGACTTCGCTTCGATGGAACGTGAACTAGAGAAACTTTGA

>DNA

CCACAAAGCCCCCGCCCGATCCGCCGTCTCATTCCCCTCACCGCTTCCCCCGGGGATCGCAGTGGCATCCGCGGCTCCAGCAACCAAGTAAGCGGCAGCCGAATCCAATGCTCTCTCTCGCAAGATCGGTCCGGCTGCTCGCCTCCGTCGACGGCATGGATCGGTCTCCCGTCCTCCCCGATTTCTTTCCGAAAGTCCGGTCCTTTTGCGGTGATTCGTTCCTGGTTTGGTAATTGCGGGGATTTCGGTCCGGGTTGTTGCCGAGTTTTTTTCCCCCTCTCTTCGAGGATTTCTCCTCCTTTTTGGTGCCGATCTGGTCGTGCTCTTTCCGTAAATTCATAGGCACAAAATATGGAAACTCTTTTTATCCGATGATTGAAACATGGAAAAAATATATTTGTCCTGGGTGTTTCGTCCTTGCTGCTCCAGTCCTCCTTCTCTCGCCGGTTGATTTGCCTGCCATATAATCTCTCGTGTTTGTTCTTGTTATTTTTTTTCTTCCTTTGTTAATACTTTCCGTTCGTGGTGTTGGTGCGATGATCACAGAGTTGGAGTAGAGACCGCCAGCCAGCACGAGCGATCGCTCTTGATTTATGTCGCCTGCTCTGCTGCTCCATGGTTCTGAGCTCCCATCACCATCACCTCCTTCTGCTTTTTGCTCCCTTCTCCCACAAGCTCGGCAGAGGGGCGCCAGAACTCAGGATTATCGCTTCAAGATCTCCCGGTAAGCACGTCCGCTCAGTGCACTAGTTTCTGGTCTGGTCGCAACAACTGACGACAACAATGTCGAATCACTACTACCTGTTTCCCCTGGTCTAGTTTCTTGTATATTATTACTTGAAAGAGTCAGTATTATCACATCTTGTTATTCTTCAGAAGATGAACTCACAATAGAAAGTTCAGAGATCGTCTTATTTTAATCTTCCCTTAATATTAGTACTTTATTCCCACAAGAATAAACTGCAATAGTTTGTTCATCTAGTACTATATGTAGTACCCGTCCTGTTCTTGGTTTGCTCTGGATTAGCCATGATGAGTGTACCACCAAATGATGTGTTTTTTCTTGGTAACAAATTTGATCGATAGTTTTTTTTCTTCCAATTTAAACAGTTGTCTTGTCCTCGTTCTCATCCGGTCATCTTAATTATTGCTCCTTTAGCAAGTTTGTTCGTACGTGTGCTCCAATCTTGGACTGATTTCAAAGCTGACCTGCACAATAAATGGTGGCATTGCTTTATTTAGTCAAAAGATATGTACATGCATTCAAACATACGTTTCTCACAGCCGAGCCAGTTACAGCCATGTAAATTTGTACTGTAGTGATGAGCTGTGCTTTCTTGCTTGTGCCTCCAGATTTGGATTTGGGGTGAAGTACTCTGGATTTCTGCATTTAGACCAGTGCCCCGTGCACTTGAAGTAATAACGATACAAGAATCCCCCAAATCACGATAAGCTTGGTAAGTATTTACTCTACGCAAGGAAATGTATTTCAGAGGGGAGAAAACAGTGAATATGTCTGATCTCTCCTTTTTACCACTTGCCTAAGAAAGATTGGGCCTCATCGACAGTTTTTGGATGCTTGCAGGTTTGGTTTGGGTGGATTAAGTTTAGCACTCCTCCTTCATTCTCATTGACATTCCTCAAGTTCATAAACAATCAGCCAGAAAGCAGATTTCTTTTGTGAGTCTTCACACAAAGAGAATAACTCCGTCTTCTCTCCGGTTTGAATCAGAAGTTTGAGTCCATTCGGTTTCTGATTTTGGAGATTGTGTGCTCAGTCTCTAAACCAGGAGTTTGTCCTGGGATTTAATATTTCAGTCGGTATGTGCACATCTTATGTTCCATGTCCTTGAAATTTGCGTATGAGTGTATTTTCTTGCGAATGTTTGTATAAGTGTATTTGACACACTCACTTGTCTTCTCTGCAGATTGTAGTCAAGGTTAATAATTCAATAATCTCTGGATCTTACATAAGATTAAGGGGCTTGTTGCACAAAGAGATGATGTTGCCTTCTTCGCGGAAGGTCCGCATTTCCTGCTGTGATCCTGACGCCACCGATTCCTCTGATGAAGATGATCGGCATGCGAAGAAGGAAAAGAGAATGACGATGGAAGTACTAGTTCCAATGAAAAGCTCCCAAGTCCTCAAATCTCGAAAGACCCTCTTGCCATGCGGCACAATGAAATCAATGGGTACAGAGAAGAAGCAGCCAACCAGCAAGTACCCTGGTGTGCGCCTGCGGTCATGGGGCAAGTGGGCTGCGGAGATTCGTGATCCCGTGAGCAAGACCCGGAAATGGATTGGCACATTTACTTCTGAGGAGGCGGCAGCTGCAGCATATGAGGCAGAACGGAACCGGGTACGTGCTGAGATGTTGGCCATCAAATCTCGGCCATCTCCATCAGAACATGAAGCTTTGTCCAGCGAAGCTACTGTATCCTGTGTATCCTCTTCTGTGTCGTTTGGCGACCAGAAAGCACAAGAGGTACACAAGGTAGCGCCAATGGAGATAGACCCTGACACTGCTGATGAGAGCTTACTGCATTGCTCACCAGAACTGCCAGGTAAAGAAATTGAGGTAGATGCATTCCTTGGCCGGATGAATGTTGATGAAAGCTTAGTGCACTGCTCATCGACACCTAGCGATGAAGAAATTCCAGTGGATGCATTCCACAGCCAGATAAATGAGCTTCCTATCAGTGACTATGTTTGCACAACTGACAAACTCTCACTGGATGATATTTCAAGGCTGGCAGATATGTTTCATGTCAATGACTTCGTTGACACAACAGGCAAACCGCCTGGCGACGACTACATCGGGCTGGCAGACATCAGCCATCTACCGTTGCCAATGTTTGAGTTGGATGTAGGACTTGATTGGGAAGGTTTTGACTTCGCTTCGATGGAACGTGAACTAGAGAAACTTTGAGGCCTTACCAGCATTGAGGTTTTCATCTTTCTGACGGACGTCAGGGTGAGTTTGTGATCAGGAGATATGTTCATCTCCAATTTTGTCAGAAATTGTACAGTCCTCTCCC

>HvRAV-1

>Protein

MGVEILSSMVEHSFQYSSGASSATAESGAVGTPPRHLSLPVAIADESLTSRSASSRFKGVVPQPNGRWGAQIYERHARVWLGTFPDQDSAARAYDVASLRYRGGDAAFNFPCVVVEAELAFLAAHSKAEIVDMLRKQTYADELRQGLRRGRGMGVRAQPMPSWARVPLFEKAVTPSDVGKLNRLVVPKQHAEKHFPLKRSPETTTTTGNGVLLNFEDGQGKVWRFRYSYWNSSQSYVLTKGWSRFVREKGLGAGDSIMFSCSAYGQEKQFFIDCKKNTTVNGGKSASPLQVMEITKAEQVRVVRLFGVDIAGVKRERAATAEQGPQGWFKRQCMAHGQHSPALGDFAL

>cDNA

TCTACAGATAACGTGTACACATATAGATGCTTCCAAATAAGAAACGCCACACCATCTATATATACACCAACACCATCTATATGTAGTGTACAAACCACCGCACGGCAACCATCTTGTCTAGCCCTAGCTAGCAAGCTATAGAACACCAACTCATCTGAACCTGTAAACACACATACACACACAAAATTGTTTCTATGTGTACCAAGCTTAGCTAAGATAGTATCACAGCCCACAAAGGCACACAGAAACACACTACGAAGAACACACACGCCAAGAAACCACACAAAAGCAACAAGCCATGGGGGTGGAGATCCTGAGCTCCATGGTGGAGCACTCCTTCCAGTACTCTTCGGGCGCGTCCTCGGCCACCGCGGAGTCAGGCGCCGTCGGAACACCGCCGAGGCATCTGAGCCTACCTGTCGCCATCGCCGACGAGTCCCTGACCTCACGGTCGGCGTCGTCTCGGTTCAAGGGCGTGGTGCCGCAGCCCAACGGGCGGTGGGGCGCCCAGATCTACGAGCGCCACGCTCGCGTCTGGCTCGGCACGTTCCCAGACCAGGACTCGGCGGCGCGCGCCTACGACGTTGCCTCGCTCAGGTACCGCGGCGGCGACGCCGCCTTCAACTTCCCGTGCGTGGTGGTGGAGGCGGAGCTCGCCTTCCTGGCGGCGCACTCCAAGGCTGAGATCGTTGACATGCTCCGGAAGCAGACCTACGCCGATGAACTCCGCCAGGGACTACGGCGCGGCCGTGGCATGGGGGTGCGCGCGCAGCCGATGCCGTCGTGGGCGCGGGTTCCCCTTTTCGAGAAGGCCGTGACCCCTAGCGATGTCGGCAAGCTCAATCGCCTGGTGGTGCCGAAGCAGCACGCCGAGAAGCACTTCCCCCTGAAGCGCAGCCCGGAGACGACGACCACCACCGGCAACGGCGTACTGCTCAACTTTGAGGACGGCCAGGGAAAAGTGTGGAGGTTCCGGTACTCATATTGGAACAGCAGCCAGAGCTACGTGCTCACCAAAGGCTGGAGCCGCTTCGTCCGGGAGAAGGGCCTCGGCGCCGGTGACTCCATCATGTTCTCCTGCTCGGCGTACGGGCAGGAGAAGCAGTTCTTCATCGACTGCAAGAAGAACACGACCGTGAACGGAGGCAAATCGGCGTCGCCGCTGCAGGTGATGGAGATTGCCAAAGCAGAACAAGTCCGCGTCGTTAGACTGTTCGGTGTCGACATCGCCGGGGTGAAGAGGGAGCGAGCGGCGACGGCGGAGCAAGGCCCGCAGGGGTGGTTCAAGAGGCAATGCATGGCACACGGCCAGCACTCTCCTGCCCTAGGTGACTTCGCCTTATAGCATCTGCATTTTTTCGTCCCTCTTCTTGTTGTTGTCAAATGAAATATGTTGATCCATTCGTGGATTAGAACAATTGTGTTCCTCAATTTTGCGTAGAACTCATTCTTAAGTCTGATTGCAA

>CDS

ATGGGGGTGGAGATCCTGAGCTCCATGGTGGAGCACTCCTTCCAGTACTCTTCGGGCGCGTCCTCGGCCACCGCGGAGTCAGGCGCCGTCGGAACACCGCCGAGGCATCTGAGCCTACCTGTCGCCATCGCCGACGAGTCCCTGACCTCACGGTCGGCGTCGTCTCGGTTCAAGGGCGTGGTGCCGCAGCCCAACGGGCGGTGGGGCGCCCAGATCTACGAGCGCCACGCTCGCGTCTGGCTCGGCACGTTCCCAGACCAGGACTCGGCGGCGCGCGCCTACGACGTTGCCTCGCTCAGGTACCGCGGCGGCGACGCCGCCTTCAACTTCCCGTGCGTGGTGGTGGAGGCGGAGCTCGCCTTCCTGGCGGCGCACTCCAAGGCTGAGATCGTTGACATGCTCCGGAAGCAGACCTACGCCGATGAACTCCGCCAGGGACTACGGCGCGGCCGTGGCATGGGGGTGCGCGCGCAGCCGATGCCGTCGTGGGCGCGGGTTCCCCTTTTCGAGAAGGCCGTGACCCCTAGCGATGTCGGCAAGCTCAATCGCCTGGTGGTGCCGAAGCAGCACGCCGAGAAGCACTTCCCCCTGAAGCGCAGCCCGGAGACGACGACCACCACCGGCAACGGCGTACTGCTCAACTTTGAGGACGGCCAGGGAAAAGTGTGGAGGTTCCGGTACTCATATTGGAACAGCAGCCAGAGCTACGTGCTCACCAAAGGCTGGAGCCGCTTCGTCCGGGAGAAGGGCCTCGGCGCCGGTGACTCCATCATGTTCTCCTGCTCGGCGTACGGGCAGGAGAAGCAGTTCTTCATCGACTGCAAGAAGAACACGACCGTGAACGGAGGCAAATCGGCGTCGCCGCTGCAGGTGATGGAGATTGCCAAAGCAGAACAAGTCCGCGTCGTTAGACTGTTCGGTGTCGACATCGCCGGGGTGAAGAGGGAGCGAGCGGCGACGGCGGAGCAAGGCCCGCAGGGGTGGTTCAAGAGGCAATGCATGGCACACGGCCAGCACTCTCCTGCCCTAGGTGACTTCGCCTTATAG

>DNA

ATTTAATAGACTTTTCGGTGTTCAAAATCCTCGGTGCATCTCTCTTTCTAAGAACGTTCTTCCCTCTCATTCACCTGATAATTAGTTAATTCAGTTAGTTGACTCCACTTTGTGAAGTCGTCAAGTCCTACTACTAGTATTGGCCACACAAAAATAAAGCTTCATTTCTTTTGACAAATTAAATATGCTTCCTTCTTATATACTATTAATAATAGTAACATTTTCTTAACATTTCATAATTTTTGTCTCCAAGTGTTTAGTATACCTGCTATCTCATGGGCAGACATTTTTGCAACTTAGTCCATCACTTGTCTGTGAACTTGCTAACTGCAGTTAGTTGCCTATAAAAGGCTGACAATCAAACTCAAGAAAATGGGTGACGTTATATGCATACCTATTCCTCAATGACGACACCGGCCTTGACTTTCTTCATACACGTGCACATCAAATGAGATAGCATATATACCTCCTTGTGTTTGTATCGTCGCGAAGACTGTTAGCTTCTACTGTAATTATATTATGCAGGCAAATCCTATGGGTGGAAAAGGAACAAATATATATCAAATAAACTAATTAGTAGGCTGCTTTTATTTAGGCACGTGTGGCACCCATTTAAGTGCTAAACAAAACGGCGTGTCATGACTCGGAATGTCATACAATTACAAAACACTATCCCTTTGTTAACAAGAGTTTTGGCCCTGAGAGCGATCCATTTAGTTTTGTGATTAAAGCACCATCCAAGATCCACACTCTTCAGAACCACCTATATAGTTGGATTAGGATCAATTAATTCTTGTGTATTGGTCTATACTAAAGATAATAATCCCGTAAGGTCTGAATATGTCGGCCAGATAATTGAATTTCGGTTCTTATCTTAATCGTGCAAAAATAAAGTACATGCGACAAAGCATAACTCAAAGTTACAAAGTTGAATTTCGGTTGTTTGATCTTCAGTTCTTATCAAAGAAAGGTCCTAGTGAGAAAGAACAAAGTACATAGCTAACAAGAGGGAAGGTAACATGAGAAAAGTACCGCGTGCTTACTTAAGCAAAGGATGTTTTGGTTCTTTACATTCTAGAATCAGCGACTTTTGACCAAAAATATTTGTACAAATATCCACATAAATGATAATAATAGACTGTGAAACTTTTGGATTGGAAAGCCTTTTCTTTTGGGTTGCACAAAACTTATTGGTTGTGTGAAATCTATTATATAATTACAGCTAAGAAATCGAAACGTAGTTTTTAGGGTTTCATGGTCAAAGATAAAGAAACTTGACTACACACTATTTTGGGGAATAGAGCTGGTGTATTTTGTGCATTATTTGTCGGAATTTAAACAAGAGTATATTTTTCATGCATATCTTCATGTGGGCAAATATTCAAATGTACCAAAAGTATATTTTTCATGCATATCTTCTAACCAATTTTACGTTATTTTTGCACTATAAGCCAATTTTACTCATTCATGTGGGCAATGAATAGCCATCATAGTGTACTACATCTAAATGCATCAAGTACATTGTACCATAAACCAATACATCAACATCCAAGTTGCCAAAATAAAATAATACCTAGCTCCCACTTGCTACAAGCCAGAAAACCACATGTAATATGGAACTTTCGAGGGTGCAAACATGAAAATCTTCACCATCACTTCAAATTAACCACAAACTGAATATGCCCCAACATATAACTCCACTTCTACAGATAACGTGTACACATATAGATGCTTCCAAATAAGAAACGCCACACCATCTATATATACACCAACACCATCTATATGTAGTGTACAAACCACCGCACGGCAACCATCTTGTCTAGCCCTAGCTAGCAAGCTATAGAACACCAACTCATCTGAACCTGTAAACACACATACACACACAAAATTGTTTCTATGTGTACCAAGCTTAGCTAAGATAGTATCACAGCCCACAAAGGCACACAGAAACACACTACGAAGAACACACACGCCAAGAAACCACACAAAAGCAACAAGCCATGGGGGTGGAGATCCTGAGCTCCATGGTGGAGCACTCCTTCCAGTACTCTTCGGGCGCGTCCTCGGCCACCGCGGAGTCAGGCGCCGTCGGAACACCGCCGAGGCATCTGAGCCTACCTGTCGCCATCGCCGACGAGTCCCTGACCTCACGGTCGGCGTCGTCTCGGTTCAAGGGCGTGGTGCCGCAGCCCAACGGGCGGTGGGGCGCCCAGATCTACGAGCGCCACGCTCGCGTCTGGCTCGGCACGTTCCCAGACCAGGACTCGGCGGCGCGCGCCTACGACGTTGCCTCGCTCAGGTACCGCGGCGGCGACGCCGCCTTCAACTTCCCGTGCGTGGTGGTGGAGGCGGAGCTCGCCTTCCTGGCGGCGCACTCCAAGGCTGAGATCGTTGACATGCTCCGGAAGCAGACCTACGCCGATGAACTCCGCCAGGGACTACGGCGCGGCCGTGGCATGGGGGTGCGCGCGCAGCCGATGCCGTCGTGGGCGCGGGTTCCCCTTTTCGAGAAGGCCGTGACCCCTAGCGATGTCGGCAAGCTCAATCGCCTGGTGGTGCCGAAGCAGCACGCCGAGAAGCACTTCCCCCTGAAGCGCAGCCCGGAGACGACGACCACCACCGGCAACGGCGTACTGCTCAACTTTGAGGACGGCCAGGGAAAAGTGTGGAGGTTCCGGTACTCATATTGGAACAGCAGCCAGAGCTACGTGCTCACCAAAGGCTGGAGCCGCTTCGTCCGGGAGAAGGGCCTCGGCGCCGGTGACTCCATCATGTTCTCCTGCTCGGCGTACGGGCAGGAGAAGCAGTTCTTCATCGACTGCAAGAAGAACACGACCGTGAACGGAGGCAAATCGGCGTCGCCGCTGCAGGTGATGGAGATTGCCAAAGCAGAACAAGTCCGCGTCGTTAGACTGTTCGGTGTCGACATCGCCGGGGTGAAGAGGGAGCGAGCGGCGACGGCGGAGCAAGGCCCGCAGGGGTGGTTCAAGAGGCAATGCATGGCACACGGCCAGCACTCTCCTGCCCTAGGTGACTTCGCCTTATAGCATCTGCATTTTTTCGTCCCTCTTCTTGTTGTTGTCAAATGAAATATGTTGATCCATTCGTGGATTAGAACAATTGTGTTCCTCAATTTTGCGTAGAACTCATTCTTAAGTCTGATTGCAA

>HvRAV-2

>Protein

MGMEILSSTVEHCSQYSSSASTATTESGAAGRSTTALSLPVAITDESVTSRSASAQPASSRFKGVVPQPNGRWGSQIYERHARVWLGTFPDQDSAARAYDVASLRYRGRDAATNFPCAAAEAELAFLTAHSKAEIVDMLRKHTYADELRQGLRRGRGMGARAQPTPSWARVPLFEKAVTPSDVGKLNRLVVPKQHAEKHFPLKCTAETTTTTGNGVLLNFEDGEGKVWRFRYSYWNSSQSYVLTKGWSSFVREKGLGAGDSIVFSSSAYGQEKQLFINCKKNTTMNGGKTALPLPVVETAKGEQDHVVKLFGVDIAGVKRVRAATGELGPPELFKRQSVAHGCGRMNYICYSIGTIGPLMLN

>cDNA

TACGTGTATACATGTAGACGCACCCCTACAAATCCATATCTACAAATATACGCCATCACTTGTGTAGGACTACACACATCCCACAGCTACCACCTCATCATCGTCTAGCCCTAGCTAGAGCAGCAACTCATCTTAATCTGTAAACACACATACACACACAAAAATACCTCTATATGTAGCTAACTTAGCTCAGCTAGTATCGGTTACCCGCTAGCATCACAGCCCACACAAGCGCACATACACACACTCCAAAGAAAACACACGCGCCAAGAAAACAAAGAAAATCAACAAGCCATGGGGATGGAAATCCTGAGCTCCACGGTGGAGCACTGCTCCCAGTACTCTTCCAGCGCGTCCACGGCCACAACGGAGTCAGGCGCCGCCGGAAGATCGACGACGGCTCTGAGCCTACCAGTTGCCATCACCGACGAGTCCGTTACCTCGCGGTCGGCATCGGCGCAGCCGGCGTCATCACGGTTCAAGGGCGTGGTGCCGCAGCCCAACGGGCGGTGGGGCTCCCAGATCTACGAGCGCCACGCTCGCGTCTGGCTCGGCACCTTCCCGGATCAGGACTCGGCGGCGCGTGCCTACGACGTTGCCTCGCTCAGGTACCGGGGCCGCGATGCCGCCACCAACTTCCCGTGCGCCGCTGCGGAAGCGGAGCTCGCCTTCCTGACCGCGCACTCCAAGGCCGAGATCGTCGACATGCTCCGGAAGCACACCTACGCCGACGAACTCCGCCAGGGCCTGCGGCGCGGCCGCGGCATGGGTGCGCGCGCGCAGCCGACGCCGTCGTGGGCGCGGGTTCCCCTTTTCGAGAAGGCTGTGACCCCTAGCGATGTCGGCAAGCTCAATCGCCTGGTGGTGCCGAAGCAGCACGCCGAGAAGCACTTCCCCCTGAAGTGCACCGCAGAGACGACGACCACCACCGGCAACGGCGTGCTGCTAAACTTCGAGGATGGTGAGGGGAAGGTGTGGAGGTTCCGGTACTCGTATTGGAACAGTAGCCAGAGCTACGTGCTCACCAAAGGCTGGAGCAGCTTCGTCCGGGAGAAGGGCCTCGGCGCAGGCGACTCCATCGTCTTCTCCTCCTCGGCGTACGGGCAGGAGAAGCAGTTATTCATCAACTGCAAAAAGAACACGACTATGAACGGCGGCAAAACAGCGTTGCCGCTGCCAGTGGTGGAGACTGCCAAAGGAGAACAAGACCACGTCGTTAAGTTGTTCGGTGTTGACATCGCCGGTGTGAAGAGGGTGCGAGCGGCGACGGGGGAGCTAGGCCCGCCGGAGTTGTTCAAGAGACAATCCGTGGCACACGGATGCGGAAGGATGAACTACATTTGCTACTCCATAGGGACAATAGGACCTCTTATGCTCAACTGAGTAGTAGGGTGACC

>CDS

ATGGGGATGGAAATCCTGAGCTCCACGGTGGAGCACTGCTCCCAGTACTCTTCCAGCGCGTCCACGGCCACAACGGAGTCAGGCGCCGCCGGAAGATCGACGACGGCTCTGAGCCTACCAGTTGCCATCACCGACGAGTCCGTTACCTCGCGGTCGGCATCGGCGCAGCCGGCGTCATCACGGTTCAAGGGCGTGGTGCCGCAGCCCAACGGGCGGTGGGGCTCCCAGATCTACGAGCGCCACGCTCGCGTCTGGCTCGGCACCTTCCCGGATCAGGACTCGGCGGCGCGTGCCTACGACGTTGCCTCGCTCAGGTACCGGGGCCGCGATGCCGCCACCAACTTCCCGTGCGCCGCTGCGGAAGCGGAGCTCGCCTTCCTGACCGCGCACTCCAAGGCCGAGATCGTCGACATGCTCCGGAAGCACACCTACGCCGACGAACTCCGCCAGGGCCTGCGGCGCGGCCGCGGCATGGGTGCGCGCGCGCAGCCGACGCCGTCGTGGGCGCGGGTTCCCCTTTTCGAGAAGGCTGTGACCCCTAGCGATGTCGGCAAGCTCAATCGCCTGGTGGTGCCGAAGCAGCACGCCGAGAAGCACTTCCCCCTGAAGTGCACCGCAGAGACGACGACCACCACCGGCAACGGCGTGCTGCTAAACTTCGAGGATGGTGAGGGGAAGGTGTGGAGGTTCCGGTACTCGTATTGGAACAGTAGCCAGAGCTACGTGCTCACCAAAGGCTGGAGCAGCTTCGTCCGGGAGAAGGGCCTCGGCGCAGGCGACTCCATCGTCTTCTCCTCCTCGGCGTACGGGCAGGAGAAGCAGTTATTCATCAACTGCAAAAAGAACACGACTATGAACGGCGGCAAAACAGCGTTGCCGCTGCCAGTGGTGGAGACTGCCAAAGGAGAACAAGACCACGTCGTTAAGTTGTTCGGTGTTGACATCGCCGGTGTGAAGAGGGTGCGAGCGGCGACGGGGGAGCTAGGCCCGCCGGAGTTGTTCAAGAGACAATCCGTGGCACACGGATGCGGAAGGATGAACTACATTTGCTACTCCATAGGGACAATAGGACCTCTTATGCTCAACTGA

>DNA

AAGTTAAGTGAAGAACAAAATAGTCCTCTATTTGAAAAAAAAAAAAAAAAAAAAAAAAAAAAACCATAAGGTTTCATCTTATGTCAATTTTTCTCTTCACCCTCCTAATCCGTCATCCCACCTCCACCATTTTTTTCCAGTTTTCCACCCCCTTCTGGTTTTCATCAAATCAATATGTGATAACTCAATAAACCAGATCAATTTGTGGTATAACAATAAATTCAACATTGCATAAATAAATTGTTATTTGTGGTAAAATATGGTACTGGTTAGACAGCTTTGCTAACCCAATAAATAGCAGTTAATCTCTATCTTTGGTAATGCAATAAAGCAGATCATACATGGGCGCATACATATTTTGTGAGTTTAAGAAATGGGAGGAAAATATGAATTCCCCACGGAAAGTGCACGATCACATACCTAGTCAAATAAAATAATTAGTAAGCCTATTATTTTTATTTACGCGCGTGTGACACCCTTTTAAGGACCACAAAAATGGTGTATCATGAGTCCGAATGTTATCCCATTAGAGAAAATGCATCTCTGTGTGAACAAAAGTTTTGGCATTTACGATGGTTAATTTAGTTTGGTGATAAAAAAATCCATCCAAGCTTGGATTGGGCTCAATTAGTCGTCCTTGTGCATCGGTATATAATCCCTAACCAAGCGAAAGGATCATCTCCAAAATACGAAAAACTCAAGTTAACAAGGGTTGAACGTGTCTTCTAGATAATTGATTTTTTTTTTATCTTAACCGTGCAAAAATAAATTTACACGTGGCAAGGCTGACTCACACTTATGAAATAGTCCATGAACTAGAACTAATTCTAGTTTTTCATACTCTGTATGATAATTTATACAAACGAGTATGTACCAAAAGTATATTGTTCATATATACCTTCTAGCCAATTTTACTGCTTTTACATTATGCCAATTTTACTGTAGCCAGAATGTTCTACTAGTTACATGCATCAAGTACATTGGATCATAAATCAATACATCAACATCCAAGTTGCTAAAATAATATCTAGCTCCCACTTGCTACACGGCAGAAAACCAAAAATTTGGAGCTTTTGGAGGGTGCAAACATGAAAATATCCACCATCACTTCAAATTAACCAAAAATTGATTATGTCCAAGAACATTAGTCCACTTCCGCACATACGTGTATACATGTAGACGCACCCCTACAAATCCATATCTACAAATATACGCCATCACTTGTGTAGGACTACACACATCCCACAGCTACCACCTCATCATCGTCTAGCCCTAGCTAGAGCAGCAACTCATCTTAATCTGTAAACACACATACACACACAAAAATACCTCTATATGTAGCTAACTTAGCTCAGCTAGTATCGGTTACCCGCTAGCATCACAGCCCACACAAGCGCACATACACACACTCCAAAGAAAACACACGCGCCAAGAAAACAAAGAAAATCAACAAGCCATGGGGATGGAAATCCTGAGCTCCACGGTGGAGCACTGCTCCCAGTACTCTTCCAGCGCGTCCACGGCCACAACGGAGTCAGGCGCCGCCGGAAGATCGACGACGGCTCTGAGCCTACCAGTTGCCATCACCGACGAGTCCGTTACCTCGCGGTCGGCATCGGCGCAGCCGGCGTCATCACGGTTCAAGGGCGTGGTGCCGCAGCCCAACGGGCGGTGGGGCTCCCAGATCTACGAGCGCCACGCTCGCGTCTGGCTCGGCACCTTCCCGGATCAGGACTCGGCGGCGCGTGCCTACGACGTTGCCTCGCTCAGGTACCGGGGCCGCGATGCCGCCACCAACTTCCCGTGCGCCGCTGCGGAAGCGGAGCTCGCCTTCCTGACCGCGCACTCCAAGGCCGAGATCGTCGACATGCTCCGGAAGCACACCTACGCCGACGAACTCCGCCAGGGCCTGCGGCGCGGCCGCGGCATGGGTGCGCGCGCGCAGCCGACGCCGTCGTGGGCGCGGGTTCCCCTTTTCGAGAAGGCTGTGACCCCTAGCGATGTCGGCAAGCTCAATCGCCTGGTGGTGCCGAAGCAGCACGCCGAGAAGCACTTCCCCCTGAAGTGCACCGCAGAGACGACGACCACCACCGGCAACGGCGTGCTGCTAAACTTCGAGGATGGTGAGGGGAAGGTGTGGAGGTTCCGGTACTCGTATTGGAACAGTAGCCAGAGCTACGTGCTCACCAAAGGCTGGAGCAGCTTCGTCCGGGAGAAGGGCCTCGGCGCAGGCGACTCCATCGTCTTCTCCTCCTCGGCGTACGGGCAGGAGAAGCAGTTATTCATCAACTGCAAAAAGAACACGACTATGAACGGCGGCAAAACAGCGTTGCCGCTGCCAGTGGTGGAGACTGCCAAAGGAGAACAAGACCACGTCGTTAAGTTGTTCGGTGTTGACATCGCCGGTGTGAAGAGGGTGCGAGCGGCGACGGGGGAGCTAGGCCCGCCGGAGTTGTTCAAGAGACAATCCGTGGCACACGGTCAGCACTCTCCTTCCCTAGGTGTCTTCGCCTTATAGCATCTGCACATACACCTACATATATTCTTCTCCTCCCTTATATCTTGATCCTGTTGGTGGATTAGATAAATCTGTATAGAACTCAGTTTCAAGTCGGATTGCAAAGTGAGTTGTAGTATATGTAATTATGTGTGTGCACAATTAGGAATTCTAGCTATAGAGATCATAATAATCCTACGATTGTTTGTTGCACATCTATGTATGTACTATCATTTGGCACATCAAAAACTTCCATTCTGCCATCACATCAAGAGATCTGTTTCTGTAAGTTTCGATGAAATTTTGTTCATTGCATAATATATTGGCATTGCTACACCAATTGATATCCGCTCAGCAACTATATAAGCAAGCAGTTAGAAATGTTTATTAAACCAGTATTTTACAACATGCATGCATGAAAAAATTGGGCGCTGAATTGCTTCCCTGCCTTGGATTGTGTTACAGTTGATTTTAATTAGTTCAGTCACATGACTAGATTTATCATTGTCATATTCCCACCGATGTCTAATATCCAAGAGTTATGCATATGCTTCAAAAGATGTTATGTGAACTAATGATTTTCTATAAATGGTCTTCATACAAATAGCATTTTCAGTCAGAAACTTGAACAAAATGCGTAACTATATATCCCTTTCTAGGATGCGGAAGGATGAACTACATTTGCTACTCCATAGGGACAATAGGACCTCTTATGCTCAACTGAGTAGTAGGGTGACC

>HvRAV-3

>Protein

MGVEILSSTGEHSSQYSSGAASTATTESGVGGRPPTAPSLPVSIADESATSRSASAQSTSSRFKGVVPQPNGRWGAQIYERHARVWLGTFPDEDSAARAYDVAALRYRGREAATNFPCAAAEAELAFLAAHSKAEIVDMLRKHTYTDELRQGLRRGRGMGARAQPTPSWAREPLFEKAVTPSDVGKLNRLVVPKQHAEKHFPLKRTPETTTTTGKGVLLNFEDGEGKVWRFRYSYWNSSQSYVLTKGWSRFVREKGLGAGDSIVFSCSAYGQEKQFFIDCKKNKTMTSCPADDRGAATASPPVSEPTKGEQVRVVRLFGVDIAGEKRGRAAPVEQELFKRQCVAHSQHSPALGAFVL

>cDNA

CACATGTGCACACACCCCACATCAACCACCTCATCATATTGTCCCCAACCCTAGCTAGAATACCATCTCATCGATTTTAACCTGTAAACACACACACAACACAAGAAAAACAGCACATCATTATTTGCATATGTACCTGGCTCAGCTTAGCTAGCAGCAAAGCCCACACAAGCACAGAGACACACACTCCAGAGAAACACAGGCCAAGAAACCGCAGAAAAGCAACTAGCCATGGGGGTGGAGATCCTGAGCTCAACGGGGGAACACTCCTCCCAGTACTCTTCCGGAGCCGCGTCCACGGCGACGACGGAGTCAGGCGTGGGCGGACGGCCGCCGACTGCGCCGAGCCTACCTGTTTCCATCGCCGACGAGTCGGCGACCTCGCGGTCGGCATCGGCGCAGTCGACGTCGTCGCGGTTCAAGGGCGTGGTGCCGCAGCCCAACGGGCGGTGGGGCGCCCAGATCTACGAGCGCCACGCCCGCGTCTGGCTCGGCACGTTCCCGGACGAAGACTCTGCGGCGCGCGCCTACGACGTGGCCGCGCTCCGGTACCGGGGCCGCGAGGCCGCCACCAACTTCCCGTGCGCGGCCGCCGAGGCGGAGCTCGCCTTCCTGGCGGCACACTCCAAGGCCGAGATCGTCGACATGCTCCGGAAGCACACCTACACCGACGAGCTCCGCCAGGGCCTGCGGCGCGGCCGCGGCATGGGGGCGCGCGCGCAGCCGACGCCGTCGTGGGCGCGGGAGCCCCTTTTCGAGAAGGCCGTGACCCCGAGCGACGTGGGCAAGCTCAACCGCCTCGTTGTGCCGAAGCAGCACGCCGAGAAGCACTTCCCCCTGAAACGCACGCCGGAGACGACAACGACCACCGGCAAGGGGGTGCTTCTCAACTTCGAGGATGGCGAGGGGAAAGTGTGGAGGTTCCGGTACTCGTATTGGAACAGCAGCCAGAGCTACGTGCTCACCAAGGGATGGAGCCGCTTCGTTCGGGAGAAGGGCCTCGGTGCCGGCGACTCCATCGTGTTCTCCTGCTCGGCGTACGGTCAGGAGAAGCAGTTCTTCATCGACTGCAAGAAGAACAAGACGATGACGAGCTGCCCCGCCGATGACCGCGGCGCCGCAACAGCGTCGCCGCCAGTGTCAGAGCCAACAAAAGGAGAACAAGTCCGTGTTGTGAGGCTGTTCGGCGTCGACATCGCCGGAGAGAAGAGGGGGCGAGCGGCGCCGGTGGAGCAGGAGTTGTTCAAGAGGCAATGCGTGGCACACAGCCAGCACTCTCCAGCCCTAGGTGCCTTCGTCTTATAGCATCTGCACATACAGCTCTATATTCCTCTCCGGTTCTCCTCCCTCTTGTTCTGGTTGTTACATGATAGTATATGTTGATCTGTTTGTGGATTAGAGTAATTTTGTTCCTGAATTTTGCGTAGAACTCGTTCTTATGTCCGATTGCAAAATGAGTTGTATGTAATTATGTGTGTACAGTACACCACTAGGATTCTAGCTAGAGAGATCATAATAATCCTAAGATGTTGTTGCATATATCTATGTATATGTACTACCATCCTGGCACATCAATAAATTAAAATTCCATTCTGCCATCTAATTAAGAGATGTGTTTCCTGTTTGTTAGCATCAAGATTTTGTTCACTAGTACAATTGATCCCTCAGGAACTAAGTAAGCTAGCAAGCAATAAGAAACCTTTGCTAAACCATTATTTCGCATACATGCATGGAAAATTATGTGCGCTGGGATCTTGATATGACAAATAATTGAAATTGGTTGCTGGCCATGGACTGTGTTACACTTACATGGCTAAATTTGCCATTTATCTCCATTTGCGTTCATTGTTGGCATATATTGCTATATCTTGCTTGAGTGTGGATCTCTGTTGCACTATATAGTTGTACATCTCCCTCTCGCTACTTGCTTCGAATTCATCGTCCTTACATTAGCATTACCACCGTTCCAAAACAAGTGATGTTGTCTTAGTTCAAGTTTGAACTAAAACAGCGTCACTTAGTTACGAAG

>CDS

ATGGGGGTGGAGATCCTGAGCTCAACGGGGGAACACTCCTCCCAGTACTCTTCCGGAGCCGCGTCCACGGCGACGACGGAGTCAGGCGTGGGCGGACGGCCGCCGACTGCGCCGAGCCTACCTGTTTCCATCGCCGACGAGTCGGCGACCTCGCGGTCGGCATCGGCGCAGTCGACGTCGTCGCGGTTCAAGGGCGTGGTGCCGCAGCCCAACGGGCGGTGGGGCGCCCAGATCTACGAGCGCCACGCCCGCGTCTGGCTCGGCACGTTCCCGGACGAAGACTCTGCGGCGCGCGCCTACGACGTGGCCGCGCTCCGGTACCGGGGCCGCGAGGCCGCCACCAACTTCCCGTGCGCGGCCGCCGAGGCGGAGCTCGCCTTCCTGGCGGCACACTCCAAGGCCGAGATCGTCGACATGCTCCGGAAGCACACCTACACCGACGAGCTCCGCCAGGGCCTGCGGCGCGGCCGCGGCATGGGGGCGCGCGCGCAGCCGACGCCGTCGTGGGCGCGGGAGCCCCTTTTCGAGAAGGCCGTGACCCCGAGCGACGTGGGCAAGCTCAACCGCCTCGTTGTGCCGAAGCAGCACGCCGAGAAGCACTTCCCCCTGAAACGCACGCCGGAGACGACAACGACCACCGGCAAGGGGGTGCTTCTCAACTTCGAGGATGGCGAGGGGAAAGTGTGGAGGTTCCGGTACTCGTATTGGAACAGCAGCCAGAGCTACGTGCTCACCAAGGGATGGAGCCGCTTCGTTCGGGAGAAGGGCCTCGGTGCCGGCGACTCCATCGTGTTCTCCTGCTCGGCGTACGGTCAGGAGAAGCAGTTCTTCATCGACTGCAAGAAGAACAAGACGATGACGAGCTGCCCCGCCGATGACCGCGGCGCCGCAACAGCGTCGCCGCCAGTGTCAGAGCCAACAAAAGGAGAACAAGTCCGTGTTGTGAGGCTGTTCGGCGTCGACATCGCCGGAGAGAAGAGGGGGCGAGCGGCGCCGGTGGAGCAGGAGTTGTTCAAGAGGCAATGCGTGGCACACAGCCAGCACTCTCCAGCCCTAGGTGCCTTCGTCTTATAG

>DNA

GGAGGGAGTATTAGTTTACAGATGGAGTAACTAGTAAGGCTCCTAGTTTTCTTTAGGTGTTCGTGATACCCATTTAAGTACCAAATAAGGAAAAAGATGCCTAGAGTTTTGCCATTTACATCGACTGATTTGGTTTACTGATTAAAACTCCATCCAAGAATCAAACTATGTGTAACCACTAATATTTTTTAGAGTTAGATTAGGCTCAATTAATCATAGTGCAATGGTGCATAATCCCTAGACAGGTGAAGGGATTGTATCTAATATTGTCAAATCCAAATCAACAAGACATGAACGGGTTGACCAAGTAATTTAATTATGATTTCTATCTTTACCATGAAAAAGTAATTTTATATGCATCAAAGCTGACTTATATTGATGAAATATTGTATAAATTTTATTTAATTCTAGTTTTCATTTATTATTTAGAATATTTTCTTTTCAAAAAATTGAAGTGTTAAATCGTAAGGGACAATGCATTTGCAGTTCGTCGTGCGTGTCTTTTTTGGTGTGCAACCTAATACCCTTTTTCCGAAACTATAATAGAGGAGATAATATGACACTTCACCTAAAATAAAAATGTATGGGAAATCAAGAAAGTAATAATTTAATAATTTAATTAGTAATTTCCAAAACTGAGAATATTACAAAGAGTAACGATATTGCAAGAACTAAATTAGTCCACCGAGAGTATGTATTTCGAAAATTGTTTTATTTAAGAAAAGTCAATATTGTTTGATTTGTTGACGGTATGAAGGTGTGAAAGCATGTTGATTCCAGACCAGGAAGATCAAAGGAAAGCCCTGGTAAGAAAGAACAAAGTGCATGACTAAAAAGAAGGAAGGAAAATGAACAAAACTACTCATTGTTTACTAAAATATACGATATATAGGTTTTGCACATTCTTGAATGAGTGACTATGACTAACAAAATCTGTATGAATTTGCACATAAATAATAAATACATAACAATTGTGAAACTACTATTTCGGATTTGCAAACATTTTCTTTTGGGTTGCACAAAATTATTATTGTAAGAAATTTATAGTGCAATTTGTGTCTAAGTGTCCAAATAATTTTACAGGGTTACGTGGTCAAATATGACCGTTGTTCGACAACATATTATTTTTCTAACAGGGCATTATGTGTGAGAATTTAAACAAAACAACACATTATATATGTACCAAAAGTATGTTCTTCATGCATATCTCCAAGCCAGTTTACACATCTGTGGGTTTGGACATTGTACTACATGCACAGGTACATTGAATTCGTAAAGCAATACATCAGCAACCAAGTTGCTAAAATAAAATAATAGCCAGTTCTCACTTCCTACACGCCAGAAAACCACATGTAATATTGAGCTTTTGGAGGGTGCAAACATGAAAATCTCCACCATCACTTCAAATTAACCACAAACTACTAACTATATACTCCTAGTATGTTCCAGCATATTACTCCACTTCCACAAAATACATGTACACATTTAGATGCATCCAAAATAGAAACACCACAATATCTATATATATACCGCCAACACATGTGCACACACCCCACATCAACCACCTCATCATATTGTCCCCAACCCTAGCTAGAATACCATCTCATCGATTTTAACCTGTAAACACACACACAACACAAGAAAAACAGCACATCATTATTTGCATATGTACCTGGCTCAGCTTAGCTAGCAGCAAAGCCCACACAAGCACAGAGACACACACTCCAGAGAAACACAGGCCAAGAAACCGCAGAAAAGCAACTAGCCATGGGGGTGGAGATCCTGAGCTCAACGGGGGAACACTCCTCCCAGTACTCTTCCGGAGCCGCGTCCACGGCGACGACGGAGTCAGGCGTGGGCGGACGGCCGCCGACTGCGCCGAGCCTACCTGTTTCCATCGCCGACGAGTCGGCGACCTCGCGGTCGGCATCGGCGCAGTCGACGTCGTCGCGGTTCAAGGGCGTGGTGCCGCAGCCCAACGGGCGGTGGGGCGCCCAGATCTACGAGCGCCACGCCCGCGTCTGGCTCGGCACGTTCCCGGACGAAGACTCTGCGGCGCGCGCCTACGACGTGGCCGCGCTCCGGTACCGGGGCCGCGAGGCCGCCACCAACTTCCCGTGCGCGGCCGCCGAGGCGGAGCTCGCCTTCCTGGCGGCACACTCCAAGGCCGAGATCGTCGACATGCTCCGGAAGCACACCTACACCGACGAGCTCCGCCAGGGCCTGCGGCGCGGCCGCGGCATGGGGGCGCGCGCGCAGCCGACGCCGTCGTGGGCGCGGGAGCCCCTTTTCGAGAAGGCCGTGACCCCGAGCGACGTGGGCAAGCTCAACCGCCTCGTTGTGCCGAAGCAGCACGCCGAGAAGCACTTCCCCCTGAAACGCACGCCGGAGACGACAACGACCACCGGCAAGGGGGTGCTTCTCAACTTCGAGGATGGCGAGGGGAAAGTGTGGAGGTTCCGGTACTCGTATTGGAACAGCAGCCAGAGCTACGTGCTCACCAAGGGATGGAGCCGCTTCGTTCGGGAGAAGGGCCTCGGTGCCGGCGACTCCATCGTGTTCTCCTGCTCGGCGTACGGTCAGGAGAAGCAGTTCTTCATCGACTGCAAGAAGAACAAGACGATGACGAGCTGCCCCGCCGATGACCGCGGCGCCGCAACAGCGTCGCCGCCAGTGTCAGAGCCAACAAAAGGAGAACAAGTCCGTGTTGTGAGGCTGTTCGGCGTCGACATCGCCGGAGAGAAGAGGGGGCGAGCGGCGCCGGTGGAGCAGGAGTTGTTCAAGAGGCAATGCGTGGCACACAGCCAGCACTCTCCAGCCCTAGGTGCCTTCGTCTTATAGCATCTGCACATACAGCTCTATATTCCTCTCCGGTTCTCCTCCCTCTTGTTCTGGTTGTTACATGATAGTATATGTTGATCTGTTTGTGGATTAGAGTAATTTTGTTCCTGAATTTTGCGTAGAACTCGTTCTTATGTCCGATTGCAAAATGAGTTGTATGTAATTATGTGTGTACAGTACACCACTAGGATTCTAGCTAGAGAGATCATAATAATCCTAAGATGTTGTTGCATATATCTATGTATATGTACTACCATCCTGGCACATCAATAAATTAAAATTCCATTCTGCCATCTAATTAAGAGATGTGTTTCCTGTTTGTTAGCATCAAGATTTTGTTCACTAGTACAATTGATCCCTCAGGAACTAAGTAAGCTAGCAAGCAATAAGAAACCTTTGCTAAACCATTATTTCGCATACATGCATGGAAAATTATGTGCGCTGGGATCTTGATATGACAAATAATTGAAATTGGTTGCTGGCCATGGACTGTGTTACACTTACATGGCTAAATTTGCCATTTATCTCCATTTGCGTTCATTGTTGGCATATATTGCTATATCTTGCTTGAGTGTGGATCTCTGTTGCACTATATAGTTGTACATCTCCCTCTCGCTACTTGCTTCGAATTCATCGTCCTTACATTAGCATTACCACCGTTCCAAAACAAGTGATGTTGTCTTAGTTCAAGTTTGAACTAAAACAGCGTCACTTAGTTACGAAG

>HvRAV-4

>Protein

MASGKPTNPGMEDDNDMEYSSAESGAEDAAESSSSPVLAPPRAAPSSRFKGVVPQPNGRWGAQIYEKHSRVWLGTFPDEDAAARAYDVAALRFRGPDAVINHQRPTAAEEAGSSSSRSEIDPELGFLADHSKAEIVDMLRKHTYDDELRQGLRRGRGRAQPTPAWARELLFEKAVTPSDVGKLNRLVVPKQHAEKHFPPTTAAATGSNGKGVLLNFEDGEGKVWRFRYSYWNSSQSYVLTKGWSRFVKETGLRAGDTVAFYRSAYGNDTEDQLFIDYRKMNRDDEAADAAISEENETGHVAVKLFGVDIAGGGMAGSSGG

>cDNA

CCAGCGCAAGTGTAAAATTGGAAGCAACCATGCACCAATCTCCATATATACACAAGAAATCACCAACTATGCTCGTACAATAATCCCCACGATGGCATCTGGCAAGCCGACAAACCCCGGTATGGAGGACGACAACGACATGGAGTACTCGTCGGCGGAATCGGGAGCCGAGGACGCGGCGGAGTCGTCATCGTCGCCGGTGCTGGCGCCGCCGCGGGCGGCTCCATCGTCGCGGTTCAAGGGCGTCGTGCCGCAGCCCAATGGGCGGTGGGGCGCGCAGATCTACGAGAAGCACTCGCGGGTGTGGCTCGGCACGTTCCCCGACGAGGACGCTGCCGCGCGCGCCTACGACGTGGCCGCGCTCCGCTTCCGCGGCCCGGACGCCGTCATCAACCACCAGCGCCCGACGGCGGCGGAGGAGGCCGGCTCGTCTTCGTCCAGGAGCGAGATCGATCCCGAGCTCGGCTTCCTCGCCGACCACTCCAAGGCCGAGATCGTGGACATGCTCCGGAAGCACACCTACGACGACGAGCTCCGGCAGGGCCTGCGCCGCGGGCGCGGGCGCGCTCAGCCGACGCCTGCGTGGGCGCGAGAGCTTCTCTTCGAGAAAGCCGTGACCCCGAGCGACGTCGGCAAGCTCAACCGCCTCGTGGTGCCGAAGCAGCACGCCGAGAAGCACTTCCCGCCGACCACTGCGGCGGCCACCGGCAGCAACGGCAAGGGCGTGCTGCTCAACTTCGAGGACGGAGAAGGCAAGGTGTGGCGCTTCCGGTACTCGTACTGGAACAGCAGCCAGAGCTACGTGCTCACCAAGGGCTGGAGCCGCTTCGTCAAAGAGACGGGCCTCCGCGCCGGTGACACGGTGGCCTTCTACCGGTCGGCGTACGGGAATGACACCGAGGATCAGCTCTTTATCGACTACAGAAAGATGAACAGGGATGACGAAGCTGCCGACGCGGCGATTTCGGAAGAGAATGAGACCGGCCATGTCGCCGTCAAGCTCTTCGGCGTTGACATTGCCGGAGGAGGGATGGCGGGATCATCAGGTGGCTGAAGGGCACGGCCGGCCGGTAAGTTCTTGGTCTTCTGCCGTAGTGTGATTGCAATTGCATAGTAGTATATTGGTTGCTGGCTACTCGATCTACATTTCCATTTCGGGAAGCAAGAATAGACAGAAACAATTATTTAATCACATGATCACACGATATAGAAGATGCAGACAGATCGGAGCAAGAGTTGTAAGAGAGGGACAGTTCATTCGCATTTTGTATGCCCGTCTTGTCGAATAGATTCCACTATTCGAGAGGGTTATTAGAAAGTGACAATTCTTTGTGTAGTTTTTATGGTGTTCATGTTGAATGTGCGGTTACCTTTAGCAAGCCAAATCTTCT

>CDS

ATGGCATCTGGCAAGCCGACAAACCCCGGTATGGAGGACGACAACGACATGGAGTACTCGTCGGCGGAATCGGGAGCCGAGGACGCGGCGGAGTCGTCATCGTCGCCGGTGCTGGCGCCGCCGCGGGCGGCTCCATCGTCGCGGTTCAAGGGCGTCGTGCCGCAGCCCAATGGGCGGTGGGGCGCGCAGATCTACGAGAAGCACTCGCGGGTGTGGCTCGGCACGTTCCCCGACGAGGACGCTGCCGCGCGCGCCTACGACGTGGCCGCGCTCCGCTTCCGCGGCCCGGACGCCGTCATCAACCACCAGCGCCCGACGGCGGCGGAGGAGGCCGGCTCGTCTTCGTCCAGGAGCGAGATCGATCCCGAGCTCGGCTTCCTCGCCGACCACTCCAAGGCCGAGATCGTGGACATGCTCCGGAAGCACACCTACGACGACGAGCTCCGGCAGGGCCTGCGCCGCGGGCGCGGGCGCGCTCAGCCGACGCCTGCGTGGGCGCGAGAGCTTCTCTTCGAGAAAGCCGTGACCCCGAGCGACGTCGGCAAGCTCAACCGCCTCGTGGTGCCGAAGCAGCACGCCGAGAAGCACTTCCCGCCGACCACTGCGGCGGCCACCGGCAGCAACGGCAAGGGCGTGCTGCTCAACTTCGAGGACGGAGAAGGCAAGGTGTGGCGCTTCCGGTACTCGTACTGGAACAGCAGCCAGAGCTACGTGCTCACCAAGGGCTGGAGCCGCTTCGTCAAAGAGACGGGCCTCCGCGCCGGTGACACGGTGGCCTTCTACCGGTCGGCGTACGGGAATGACACCGAGGATCAGCTCTTTATCGACTACAGAAAGATGAACAGGGATGACGAAGCTGCCGACGCGGCGATTTCGGAAGAGAATGAGACCGGCCATGTCGCCGTCAAGCTCTTCGGCGTTGACATTGCCGGAGGAGGGATGGCGGGATCATCAGGTGGCTGA

>DNA

AAGATATGTATCATAGTCTGTGAGCATATACATATTCCTTCTTCTGCTTCTTCTCTCTTTCTATATGATTTTTTTCTTTGTTTTTCCCTTGCAATACCTACCAAAACCATTGCTTGACCGCTAAGGAAAAATATATATGTTATTAGTACCAAAATAACCCTAGGGTCAAATTAATTTTTAGGATGAGACATTCATCGCTTACCCTGTTAATTATGTGATGGTGGTAACAAACAAACTTATTGTTCTCCGAACATTCACTTTGGCACATGGGAGCATATGCTCCTGCCATCCAAAAAGTTTAAAAGTGTCCAAAAAATTTGAACAAAAATTTGGCGCATACAAATATATATTCTATGTGCGCACGCCAAGTTCCGCGGAAAACCGATATTTTTTGTGGCTTGTGTAAAAAAGAAAAAACAACTTCTCGTGTAAAGCCTGTTTTAACATTGAGTATTTTCTTTTTCACACGCGACAAAAATATTGCTTTTTTTTTCGCAAAACTACTTTGGGAGCATGTAGAATTTCGAGATGTACGCGTGAAACATTTTTGCAGAATTTTGGGACATTTCATTATATGGTTTAAATGGACTTTGTTCTCCTAATTTTCTGACATTGCCAAAAGGTTTGTTTTCCCACAGGTTACTAAATATGCCGAGATGGACAAGTAGTCGAGTTGCACATTCTCGATGAATGTGCAACTTGAGCAACTACCGTTAAGGCTACTCCCAATGCTCCACCTTGTATAGGTGCTAAGGCTGCTACGTAGGTAAAAAATCTGATGTGACAAGCTAATTAATAGGAAAGAGATGAGTGTGGTGATCCCAGGAAGAAACAATGCTAAGCACGTGAACTTAGGTAAAACAATTAAATGAAAGAAATGCACCAATGCATACACAACTTTAGTTGGAAAAACATTAAATAAGGAGGCTTAGCTACAATAAGCTAAACAACTACTCCCTCCGTCCGGTAATAAGTGTATTTTTTCCTTTTCTGTCCATCAAACTAGCTAAACTTTGACTAACTTTATTGAAAAAGGTGCTAGCATTTATGACATCAAATTAGTATCAATAGATTTATCTCTACATAAAGTTTCCAAATATATCATTTTCATGCCATATATGTTGCTACTATTTTTTATAAAGTTGATCAAATTTTAAAAAGTTTGACTAAATAGAAATCTATATGTACACTTATTTGTGGATGGAGGGAGTATGCATTAGGGACTTTAGTTGCTAAACTATTTAATGTGCTTAACACCTCATCTTAGCACCAATGCATTGAAAGTGGCCTAAATGCCCACAAGTTGTCACCATCCTTGCCATGTTTTTTGTAGCTACACGGTAATTATTCATTTACCTATTTCTTAGGTGTCTGAAAAATATTATTTCCTAAGCTGCCACTAAGAATCATCTAACCCCGACGTCGGGATACATGCAACTACAATAAAATTTGACCAGACGGTTATCAATAGTTGACCGAGACATAACAGTTTCCAGAGCATTTATTCTTAATTTCAGCACTATAGCCTTTGTATCATGCTTTTCTGGTCTTCATGTGATGTTTTCGATAGTTTTTCAGCTAGTCATCGTGGATTAGTGCCTATATTGCAAAATAACACTTCGGATCCCCTTTTTGTTACATATGTTGGTGCGCATGGGTATATTCATGGACAAAGACATTTAATATTAATATGCAAGGGCAAAAATGCAATTTAAGTTAATGACCAGATTCTCACAAGTCACAAGCACGTCTACGGCCAAAGAGCATAATAATGACAACCCTAGTGCCAAAAGGTCACTAGAAGCCAACCACACATGTGTATGTAGCATAAAATAACAGCTTTTAGCAACGTATAATGCTGCAAAGCATACATAAGCACCAACATAAACACAATCACACACTAATCGATCCAGCGCAAGTGTAAAATTGGAAGCAACCATGCACCAATCTCCATATATACACAAGAAATCACCAACTATGCTCGTACAATAATCCCCACGATGGCATCTGGCAAGCCGACAAACCCCGGTATGGAGGACGACAACGACATGGAGTACTCGTCGGCGGAATCGGGAGCCGAGGACGCGGCGGAGTCGTCATCGTCGCCGGTGCTGGCGCCGCCGCGGGCGGCTCCATCGTCGCGGTTCAAGGGCGTCGTGCCGCAGCCCAATGGGCGGTGGGGCGCGCAGATCTACGAGAAGCACTCGCGGGTGTGGCTCGGCACGTTCCCCGACGAGGACGCTGCCGCGCGCGCCTACGACGTGGCCGCGCTCCGCTTCCGCGGCCCGGACGCCGTCATCAACCACCAGCGCCCGACGGCGGCGGAGGAGGCCGGCTCGTCTTCGTCCAGGAGCGAGATCGATCCCGAGCTCGGCTTCCTCGCCGACCACTCCAAGGCCGAGATCGTGGACATGCTCCGGAAGCACACCTACGACGACGAGCTCCGGCAGGGCCTGCGCCGCGGGCGCGGGCGCGCTCAGCCGACGCCTGCGTGGGCGCGAGAGCTTCTCTTCGAGAAAGCCGTGACCCCGAGCGACGTCGGCAAGCTCAACCGCCTCGTGGTGCCGAAGCAGCACGCCGAGAAGCACTTCCCGCCGACCACTGCGGCGGCCACCGGCAGCAACGGCAAGGGCGTGCTGCTCAACTTCGAGGACGGAGAAGGCAAGGTGTGGCGCTTCCGGTACTCGTACTGGAACAGCAGCCAGAGCTACGTGCTCACCAAGGGCTGGAGCCGCTTCGTCAAAGAGACGGGCCTCCGCGCCGGTGACACGGTGGCCTTCTACCGGTCGGCGTACGGGAATGACACCGAGGATCAGCTCTTTATCGACTACAGAAAGATGAACAGGGATGACGAAGCTGCCGACGCGGCGATTTCGGAAGAGAATGAGACCGGCCATGTCGCCGTCAAGCTCTTCGGCGTTGACATTGCCGGAGGAGGGATGGCGGGATCATCAGGTGGCTGAAGGGCACGGCCGGCCGGTAAGTTCTTGGTCTTCTGCCGTAGTGTGATTGCAATTGCATAGTAGTATATTGGTTGCTGGCTACTCGATCTACATTTCCATTTCGGGAAGCAAGAATAGACAGAAACAATTATTTAATCACATGATCACACGATATAGAAGATGCAGACAGATCGGAGCAAGAGTTGTAAGAGAGGGACAGTTCATTCGCATTTTGTATGCCCGTCTTGTCGAATAGATTCCACTATTCGAGAGGGTTATTAGAAAGTGACAATTCTTTGTGTAGTTTTTATGGTGTTCATGTTGAATGTGCGGTTACCTTTAGCAAGCCAAATCTTCT

>HvRAV-5

>Protein

MDSAWSCLVDDVSSGTSTGKKASPSPAAPATKPLQRVGSGASAVMDAPEPGAEADSGRGGRLPSSKYKGVVPQPNGRWGAQIYERHQRVWLGTFTGEAEAGRAYDAAAQRFRGRDAVTNFRPLAESDPDDAAELRFLAARSKAEVVDMLRKHIYPDELAQHKRAFFFAAASSPTSSSSPLASPAPSAAAARREHLFDKTVTPSDVGKLNRLVIPKQHAEKHFPLQLPSASAAVPGECKGVLLNFDDATGKVWRFRYSYWNSSQSYVLTKGWSRFVKEKGLHAGDAVEFYRAASGNNQLFIDCKLRSKSTTTTTSVNSEAAPSPAPVTRTVRLFGVDLLIAPAARHAHEHEDYGMAKTNKRTMEASVAAPTPAHAVWKKRCVDFALTYRLATTPQCPRSRDQLEGVQAAGSTFAL

>cDNA

ATGGACAGCGCGTGGAGCTGTCTCGTGGACGACGTGAGCAGCGGCACGTCCACGGGCAAGAAGGCGTCTCCGTCGCCGGCCGCGCCGGCGACCAAGCCGCTGCAGCGCGTCGGCAGCGGGGCCAGCGCGGTCATGGACGCGCCCGAGCCCGGCGCGGAGGCGGACTCCGGCCGCGGCGGGCGGCTGCCGTCGTCCAAGTACAAGGGCGTGGTGCCGCAGCCCAACGGGCGGTGGGGCGCGCAGATCTACGAGCGCCACCAGCGCGTCTGGCTCGGCACGTTCACGGGCGAGGCCGAGGCGGGCCGCGCCTACGACGCGGCGGCGCAGCGCTTCCGCGGCCGCGACGCCGTCACCAACTTCCGCCCGCTTGCCGAGTCCGACCCCGACGACGCCGCCGAGCTCCGCTTCCTCGCCGCCCGCTCCAAGGCCGAGGTTGTTGACATGCTGCGCAAGCACATCTATCCCGACGAGCTCGCGCAGCACAAGCGCGCCTTCTTCTTCGCCGCGGCGTCGTCCCCTACGTCGTCGTCGTCACCTCTCGCCTCGCCGGCTCCTTCAGCCGCGGCGGCGCGGCGCGAGCACCTGTTCGACAAGACGGTCACGCCCAGCGACGTGGGGAAGCTGAACCGGCTGGTGATCCCCAAGCAGCACGCCGAGAAGCACTTCCCGCTGCAGCTCCCTTCTGCCAGCGCCGCCGTGCCAGGCGAGTGCAAGGGCGTGCTGCTCAACTTCGATGACGCGACCGGCAAGGTGTGGAGGTTCCGGTACTCCTACTGGAACAGCAGCCAGAGCTACGTGCTCACCAAGGGGTGGAGCCGCTTCGTGAAGGAGAAGGGCCTTCACGCCGGCGACGCCGTCGAGTTCTACCGCGCCGCCTCCGGCAACAACCAGCTCTTCATCGACTGCAAGCTCCGGTCCAAGAGCACCACGACGACGACCTCCGTCAACTCGGAGGCCGCCCCATCGCCGGCACCCGTGACGAGGACAGTGCGACTCTTCGGGGTCGACCTTCTCATCGCGCCGGCGGCGAGGCACGCGCATGAGCACGAGGACTACGGCATGGCCAAGACAAACAAGAGAACCATGGAGGCCAGCGTAGCGGCGCCTACTCCGGCGCACGCGGTGTGGAAGAAGCGGTGCGTAGACTTCGCGCTGACCTACCGACTTGCCACCACCCCACAGTGCCCGAGGTCAAGAGATCAACTAGAAGGAGTACAAGCAGCTGGGAGTACATTTGCTCTATAG

>CDS

ATGGACAGCGCGTGGAGCTGTCTCGTGGACGACGTGAGCAGCGGCACGTCCACGGGCAAGAAGGCGTCTCCGTCGCCGGCCGCGCCGGCGACCAAGCCGCTGCAGCGCGTCGGCAGCGGGGCCAGCGCGGTCATGGACGCGCCCGAGCCCGGCGCGGAGGCGGACTCCGGCCGCGGCGGGCGGCTGCCGTCGTCCAAGTACAAGGGCGTGGTGCCGCAGCCCAACGGGCGGTGGGGCGCGCAGATCTACGAGCGCCACCAGCGCGTCTGGCTCGGCACGTTCACGGGCGAGGCCGAGGCGGGCCGCGCCTACGACGCGGCGGCGCAGCGCTTCCGCGGCCGCGACGCCGTCACCAACTTCCGCCCGCTTGCCGAGTCCGACCCCGACGACGCCGCCGAGCTCCGCTTCCTCGCCGCCCGCTCCAAGGCCGAGGTTGTTGACATGCTGCGCAAGCACATCTATCCCGACGAGCTCGCGCAGCACAAGCGCGCCTTCTTCTTCGCCGCGGCGTCGTCCCCTACGTCGTCGTCGTCACCTCTCGCCTCGCCGGCTCCTTCAGCCGCGGCGGCGCGGCGCGAGCACCTGTTCGACAAGACGGTCACGCCCAGCGACGTGGGGAAGCTGAACCGGCTGGTGATCCCCAAGCAGCACGCCGAGAAGCACTTCCCGCTGCAGCTCCCTTCTGCCAGCGCCGCCGTGCCAGGCGAGTGCAAGGGCGTGCTGCTCAACTTCGATGACGCGACCGGCAAGGTGTGGAGGTTCCGGTACTCCTACTGGAACAGCAGCCAGAGCTACGTGCTCACCAAGGGGTGGAGCCGCTTCGTGAAGGAGAAGGGCCTTCACGCCGGCGACGCCGTCGAGTTCTACCGCGCCGCCTCCGGCAACAACCAGCTCTTCATCGACTGCAAGCTCCGGTCCAAGAGCACCACGACGACGACCTCCGTCAACTCGGAGGCCGCCCCATCGCCGGCACCCGTGACGAGGACAGTGCGACTCTTCGGGGTCGACCTTCTCATCGCGCCGGCGGCGAGGCACGCGCATGAGCACGAGGACTACGGCATGGCCAAGACAAACAAGAGAACCATGGAGGCCAGCGTAGCGGCGCCTACTCCGGCGCACGCGGTGTGGAAGAAGCGGTGCGTAGACTTCGCGCTGACCTACCGACTTGCCACCACCCCACAGTGCCCGAGGTCAAGAGATCAACTAGAAGGAGTACAAGCAGCTGGGAGTACATTTGCTCTATAG

>DNA

ATGGACAGCGCGTGGAGCTGTCTCGTGGACGACGTGAGCAGCGGCACGTCCACGGGCAAGAAGGCGTCTCCGTCGCCGGCCGCGCCGGCGACCAAGCCGCTGCAGCGCGTCGGCAGCGGGGCCAGCGCGGTCATGGACGCGCCCGAGCCCGGCGCGGAGGCGGACTCCGGCCGCGGCGGGCGGCTGCCGTCGTCCAAGTACAAGGGCGTGGTGCCGCAGCCCAACGGGCGGTGGGGCGCGCAGATCTACGAGCGCCACCAGCGCGTCTGGCTCGGCACGTTCACGGGCGAGGCCGAGGCGGGCCGCGCCTACGACGCGGCGGCGCAGCGCTTCCGCGGCCGCGACGCCGTCACCAACTTCCGCCCGCTTGCCGAGTCCGACCCCGACGACGCCGCCGAGCTCCGCTTCCTCGCCGCCCGCTCCAAGGCCGAGGTTGTTGACATGCTGCGCAAGCACATCTATCCCGACGAGCTCGCGCAGCACAAGCGCGCCTTCTTCTTCGCCGCGGCGTCGTCCCCTACGTCGTCGTCGTCACCTCTCGCCTCGCCGGCTCCTTCAGCCGCGGCGGCGCGGCGCGAGCACCTGTTCGACAAGACGGTCACGCCCAGCGACGTGGGGAAGCTGAACCGGCTGGTGATCCCCAAGCAGCACGCCGAGAAGCACTTCCCGCTGCAGCTCCCTTCTGCCAGCGCCGCCGTGCCAGGCGAGTGCAAGGGCGTGCTGCTCAACTTCGATGACGCGACCGGCAAGGTGTGGAGGTTCCGGTACTCCTACTGGAACAGCAGCCAGAGCTACGTGCTCACCAAGGGGTGGAGCCGCTTCGTGAAGGAGAAGGGCCTTCACGCCGGCGACGCCGTCGAGTTCTACCGCGCCGCCTCCGGCAACAACCAGCTCTTCATCGACTGCAAGCTCCGGTCCAAGAGCACCACGACGACGACCTCCGTCAACTCGGAGGCCGCCCCATCGCCGGCACCCGTGACGAGGACAGTGCGACTCTTCGGGGTCGACCTTCTCATCGCGCCGGCGGCGAGGCACGCGCATGAGCACGAGGACTACGGCATGGCCAAGACAAACAAGAGAACCATGGAGGCCAGCGTAGCGGCGCCTACTCCGGCGCACGCGGTGTGGAAGAAGCGGTGCGTAGACTTCGCGCTGACCTACCGACTTGCCACCACCCCACAGTGCCCGAGGTCAAGAGATCAACTAGAAGGAGTACAAGCAGCTGGGAGTACATTTGCTCTATAG

>HvRAV-6

>Protein

MDSTSCLADDATTSSGGGGASTDKLKALAAAAAAAAPIERVGSGASAVLDAAEPGSEADSGGGRAGKLPSSRYKGVVPQPNGRWGAQIYERHQRVWLGTFPGEADAARAYDVAAQRFRGRDAVTNFRPLADADPDAAAELRFLAARSKAEVVDMLRKHTYFDELAQSKRAFAASAALSAPTTSGDAGGSASPPSPAAVREHLFDKTVTPSDVGKLNRLVIPKQNAEKHFPLQLPAGGGESKGLLLNFEDDAGKVWRFRYSYWNSSQSYVLTKGWSRFVKEKGLGAGDVVGFYRSAAGRTDEDSKFFIDCRLRPNTNTAAEADPVDQSSAPAQKAVRLFGVDLLAAPEQGMPGGCKRARDLVKPPPPKVAFKKQCIELALA

>cDNA

ATGGACAGCACGAGCTGCCTCGCGGACGACGCCACCACCAGCAGCGGCGGCGGCGGCGCCTCCACGGACAAGCTCAAGGCTTTGGCCGCGGCGGCGGCCGCGGCGGCGCCGATCGAGCGCGTGGGCAGCGGCGCCAGCGCGGTCCTCGACGCGGCCGAGCCGGGCTCCGAGGCCGACTCCGGCGGCGGCCGTGCGGGGAAGCTGCCGTCGTCCAGGTACAAGGGCGTGGTGCCGCAGCCCAACGGGCGGTGGGGCGCGCAGATCTACGAGCGGCACCAGCGCGTGTGGCTTGGCACGTTCCCCGGGGAGGCCGACGCCGCGCGCGCCTACGACGTCGCCGCGCAGCGCTTCCGCGGCCGCGACGCCGTTACCAACTTCCGCCCGCTCGCGGACGCCGACCCCGACGCCGCCGCCGAGCTCCGCTTCCTCGCCGCGCGCTCCAAGGCCGAGGTCGTCGACATGCTCCGCAAGCACACCTACTTCGACGAGCTCGCCCAGAGCAAGCGCGCCTTCGCCGCGTCGGCCGCCCTCTCCGCGCCCACCACCTCGGGCGACGCCGGCGGCAGCGCCTCGCCGCCCTCCCCGGCCGCCGTGCGCGAGCACCTCTTCGACAAGACCGTCACGCCCAGCGACGTCGGCAAGCTGAACAGGCTGGTGATACCGAAGCAGAACGCCGAGAAGCACTTCCCGCTGCAGCTCCCGGCCGGCGGCGGCGAGAGCAAGGGCCTGCTCCTCAACTTCGAGGACGATGCGGGCAAGGTGTGGCGGTTCCGCTACTCGTACTGGAACAGCAGCCAGAGCTACGTCCTCACCAAGGGCTGGAGCCGCTTCGTGAAGGAGAAGGGCCTCGGCGCCGGAGACGTCGTCGGGTTCTACCGCTCCGCCGCCGGGAGGACCGACGAAGACAGCAAGTTCTTCATTGACTGCAGGCTGCGGCCGAACACCAACACCGCCGCCGAAGCAGACCCCGTGGACCAGTCGTCGGCGCCCGCGCAGAAGGCCGTGAGACTCTTCGGCGTCGATCTTCTCGCGGCGCCGGAGCAGGGCATGCCGGGCGGGTGCAAGAGGGCCAGAGACTTGGTGAAGCCGCCGCCTCCGAAAGTGGCGTTCAAGAAGCAATGCATAGAGCTGGCGCTAGCGTAGAGTTGTTACTATTAGCTTGATCTCTCTCTCTCCAGCTAGGCGGCGGTTTTGGCTCGCATAATTCATGTGGTAGAGCTAGCCTAATTAGTCCCTTGTTCTTATCAACTTGGTTTGCTTATTTGTCATGTTCTTGCATGCTCTGATGTACTGTAAATCTTATCCTCCCAAAAGATGTATACTAATTAAGGAAGCCGTATAAGGATGCTAGATTAGATCGTTCAACGGACGACGACGAAGAGCAAGAGATGATAATAACATTTTGAATGTAGCCTAGCTCATCAGTTTCCTCGTCAAATTGAACATATCCTATTTGG

>CDS

ATGGACAGCACGAGCTGCCTCGCGGACGACGCCACCACCAGCAGCGGCGGCGGCGGCGCCTCCACGGACAAGCTCAAGGCTTTGGCCGCGGCGGCGGCCGCGGCGGCGCCGATCGAGCGCGTGGGCAGCGGCGCCAGCGCGGTCCTCGACGCGGCCGAGCCGGGCTCCGAGGCCGACTCCGGCGGCGGCCGTGCGGGGAAGCTGCCGTCGTCCAGGTACAAGGGCGTGGTGCCGCAGCCCAACGGGCGGTGGGGCGCGCAGATCTACGAGCGGCACCAGCGCGTGTGGCTTGGCACGTTCCCCGGGGAGGCCGACGCCGCGCGCGCCTACGACGTCGCCGCGCAGCGCTTCCGCGGCCGCGACGCCGTTACCAACTTCCGCCCGCTCGCGGACGCCGACCCCGACGCCGCCGCCGAGCTCCGCTTCCTCGCCGCGCGCTCCAAGGCCGAGGTCGTCGACATGCTCCGCAAGCACACCTACTTCGACGAGCTCGCCCAGAGCAAGCGCGCCTTCGCCGCGTCGGCCGCCCTCTCCGCGCCCACCACCTCGGGCGACGCCGGCGGCAGCGCCTCGCCGCCCTCCCCGGCCGCCGTGCGCGAGCACCTCTTCGACAAGACCGTCACGCCCAGCGACGTCGGCAAGCTGAACAGGCTGGTGATACCGAAGCAGAACGCCGAGAAGCACTTCCCGCTGCAGCTCCCGGCCGGCGGCGGCGAGAGCAAGGGCCTGCTCCTCAACTTCGAGGACGATGCGGGCAAGGTGTGGCGGTTCCGCTACTCGTACTGGAACAGCAGCCAGAGCTACGTCCTCACCAAGGGCTGGAGCCGCTTCGTGAAGGAGAAGGGCCTCGGCGCCGGAGACGTCGTCGGGTTCTACCGCTCCGCCGCCGGGAGGACCGACGAAGACAGCAAGTTCTTCATTGACTGCAGGCTGCGGCCGAACACCAACACCGCCGCCGAAGCAGACCCCGTGGACCAGTCGTCGGCGCCCGCGCAGAAGGCCGTGAGACTCTTCGGCGTCGATCTTCTCGCGGCGCCGGAGCAGGGCATGCCGGGCGGGTGCAAGAGGGCCAGAGACTTGGTGAAGCCGCCGCCTCCGAAAGTGGCGTTCAAGAAGCAATGCATAGAGCTGGCGCTAGCGTAG

>DNA

ATGGACAGCACGAGCTGCCTCGCGGACGACGCCACCACCAGCAGCGGCGGCGGCGGCGCCTCCACGGACAAGCTCAAGGCTTTGGCCGCGGCGGCGGCCGCGGCGGCGCCGATCGAGCGCGTGGGCAGCGGCGCCAGCGCGGTCCTCGACGCGGCCGAGCCGGGCTCCGAGGCCGACTCCGGCGGCGGCCGTGCGGGGAAGCTGCCGTCGTCCAGGTACAAGGGCGTGGTGCCGCAGCCCAACGGGCGGTGGGGCGCGCAGATCTACGAGCGGCACCAGCGCGTGTGGCTTGGCACGTTCCCCGGGGAGGCCGACGCCGCGCGCGCCTACGACGTCGCCGCGCAGCGCTTCCGCGGCCGCGACGCCGTTACCAACTTCCGCCCGCTCGCGGACGCCGACCCCGACGCCGCCGCCGAGCTCCGCTTCCTCGCCGCGCGCTCCAAGGCCGAGGTCGTCGACATGCTCCGCAAGCACACCTACTTCGACGAGCTCGCCCAGAGCAAGCGCGCCTTCGCCGCGTCGGCCGCGCTCTCCGCGCCCACCACCTCGGGCGACGCCGGCGGCAGCGCCTCGCCGCCCTCCCCGGCCGCCGTGCGCGAGCACCTCTTCGACAAGACCGTCACGCCCAGCGACGTCGGCAAGCTGAACAGGCTGGTGATACCGAAGCAGAACGCCGAGAAGCACTTCCCGCTGCAGCTCCCGGCCGGCGGCGGCGAGAGCAAGGGCCTGCTCCTCAACTTCGAGGACGATGCGGGCAAGGTGTGGCGGTTCCGCTACTCGTACTGGAACAGCAGCCAGAGCTACGTCCTCACCAAGGGCTGGAGCCGCTTCGTGAAGGAGAAGGGCCTCGGCGCCGGAGACGTCGTCGGGTTCTACCGCTCCGCCGCCGGGAGGACCGGCGAAGACAGCAAGTTCTTCATTGACTGCAGGCTGCGGCCGAACACCAACACCGCCGCCGAAGCAGACCCCGTGGACCAGTCGTCGGCGCCCGTGCAGAAGGCCGTGAGACTCTTCGGCGTCGATCTTCTCGCGGCGCCGGAGCAGGGCATGCCGGGCGGGTGCAAGAGGGCCAGAGACTTGGTGAAGCCGCCGCCTCCGAAAGTGGCGTTCAAGAAGCAATGCATAGAGCTGGCGCTAGCGTAGAGTTGTTACTATTAGCTTGATCTCTCTCTCTCCAGCTAGGCGGCGGTTTTGGCTCGCATAATTCAGGTGGTAGAGCTAGCCTAATTAGTCCCTTGTTCTTATCAACTTGGTTTGTTTATTTGTCATGTTCTTGCATGCTCTGATGTACTGTAAATCTTATCCTCCCAAAAGATGTATACTAATTAAGGAAGCCGTAGAAGGATGCTAGATTAGATCGTTCAACGGACGACGACGAAGAGCAAGAGATGATAATAACATTTTGAATGTAGCCTAGCTCATCAGTTTCCTCGTCAAATTGAACATATCCTATTTGG

>HvDREB1.1

>Protein

MDVGALSSDYSSGTPSPVGADGGNSEGFSTYMTVSSAPPKRRAGRTKFKETRHPVYKGVRRRNPGRWVCEVREPHSKQRIWLGTFETAEMAARAHDVAALALRGRAACLNFADSPRRLRVPAVGASPDEIRRAAVEAAEAFLPAPDQSNAPAEEVAAAPTMQFAGDPYYGMDDGMDFGMQGYLDMAQGMLIAPPPLVGPSATAGDGDDDGEVSLWSY

>cDNA

GTCGCGCTACCTGGCTGGCGCCTCCGGTGGCACACCGCGTTGAGCCGTGCACGCGGGGACGTCGCGCTCCACGCGCTAAACTATATAAACCGCCACCCTTCCCTTCCAAATCCAAACCACCTGCTTCCACTCCCTCCCCCTGCTTACACTCCAGCAGCCAGCATAGCCAGGCAGCCATGGACGTTGGTGCCCTCAGCAGCGACTACTCGTCGGGGACGCCGTCCCCGGTGGGCGCGGACGGCGGCAACAGTGAGGGCTTCTCGACGTACATGACGGTGTCTTCGGCTCCGCCGAAGCGGCGCGCGGGGCGGACCAAGTTCAAGGAGACGCGGCACCCGGTCTACAAGGGCGTGCGCCGGAGGAACCCCGGGAGGTGGGTCTGCGAGGTGCGGGAGCCGCACAGCAAGCAGAGGATATGGCTCGGCACGTTCGAGACCGCAGAGATGGCGGCGCGCGCGCACGACGTGGCCGCGCTGGCGCTGCGCGGCCGCGCCGCCTGCCTCAACTTCGCCGACTCTCCTCGTCGGCTCCGGGTCCCGGCCGTGGGTGCCAGCCCTGATGAGATACGGCGGGCGGCGGTAGAGGCGGCTGAGGCATTCCTGCCGGCACCCGACCAGAGCAATGCGCCCGCCGAGGAGGTAGCCGCTGCACCAACGATGCAGTTCGCCGGTGATCCGTACTACGGGATGGACGATGGTATGGACTTCGGGATGCAGGGCTACCTCGACATGGCGCAGGGGATGCTCATTGCCCCTCCTCCGTTGGTAGGTCCGTCAGCGACTGCCGGAGACGGCGACGATGACGGTGAGGTCAGCTTGTGGAGCTACTGATTACGCGCATTTGAGTTATGCCATGTGTGGTGCGACGAACTACTAATGCATGTGCAGCTTTATATAGTAATCGAGTAGAGGAAGTGTACTGCATGGTTGCCCTTTCCTTTTCTCCACTTTGTTCTAAACTTCTCTGGGTAATGTACGTTTGCTCTCTGATGAACTGCGATCAGAAGAAGCAGAGGGCCTGATCTTGAGTGTAGTGTTCGAGCTCCGAAGGAGTGTTGGGGCTAGCTATATGTATCTCCCTTGTTTATTATGCATGAGCGGAGTGTCTTCGTCGGTGTTTGGTTAAGCAAAATGTAAGTTTCTCGGTGTATAATTAGCAATATAGCACGTTGCCTTAGAGCATCTCCAGCCGTTTAGTTCCTAACGCATCCGGCAAATGCCATTTTGGC

>CDS

ATGGACGTTGGTGCCCTCAGCAGCGACTACTCGTCGGGGACGCCGTCCCCGGTGGGCGCGGACGGCGGCAACAGTGAGGGCTTCTCGACGTACATGACGGTGTCTTCGGCTCCGCCGAAGCGGCGCGCGGGGCGGACCAAGTTCAAGGAGACGCGGCACCCGGTCTACAAGGGCGTGCGCCGGAGGAACCCCGGGAGGTGGGTCTGCGAGGTGCGGGAGCCGCACAGCAAGCAGAGGATATGGCTCGGCACGTTCGAGACCGCAGAGATGGCGGCGCGCGCGCACGACGTGGCCGCGCTGGCGCTGCGCGGCCGCGCCGCCTGCCTCAACTTCGCCGACTCTCCTCGTCGGCTCCGGGTCCCGGCCGTGGGTGCCAGCCCTGATGAGATACGGCGGGCGGCGGTAGAGGCGGCTGAGGCATTCCTGCCGGCACCCGACCAGAGCAATGCGCCCGCCGAGGAGGTAGCCGCTGCACCAACGATGCAGTTCGCCGGTGATCCGTACTACGGGATGGACGATGGTATGGACTTCGGGATGCAGGGCTACCTCGACATGGCGCAGGGGATGCTCATTGCCCCTCCTCCGTTGGTAGGTCCGTCAGCGACTGCCGGAGACGGCGACGATGACGGTGAGGTCAGCTTGTGGAGCTACTGA

>DNA

GGCATAAACTATGCCCTAACATCTTGAACTCCCATTTGATCGCTACTCTTTCTTCATCAACTCGATCTTCTCCCTCTCAGATTCCAACTTCTTTTCCACCATATCCCGGTCCCTATACATCATCTATTTTTGCGCATCTAAGATGAGCTTGCACCTCTCCTCTTTTTTGTTCACCCTAACCGAAAATGCCCGTCCATGTGGAGCATATTTTTGCAGCTGCAGCGTCATAGGAGGCCCTCGCTTTCTTCCACTTCTTTCCCATCACTTGCCATACTTTTCCATTCTCCCCTAGAACATCTTCCTCTTCTTCATCTAATCCAATTGGTTGGTGGGAGATTGAGACATCATTCTTTTCTTGCCAGTCCTGAGATCGGCCACAAGTTATGTCCACTTTGCCTTGCCATTTAATATCACCCAACAATGGCTAAAATCAAATGGCTTCTTCTCCATTTCTTGATATATAGTTGCCATGACCACAGTCTAGCAGACAAAATATGTATGAAAATGACAATATGACATAAAACAAGGATATGCAAATGATGATAAGATGATGAAATGTAGCTTACTTGAGTCGCCACTCCCATCCCATTTTGAGTGTGGCCAAGCACTTGTGAATAGTAGCTGTCATACTTGTTCACTTCCCCTTGAATTACCCCATTGGTTTGGAGATATTCCATATTATGGTTGGTGACGATAGGATGTGGCTTCACATATTGCTTTTATTCATGATAATCCTTCCAAATCTTCTCCCAATACTTATTGACCTTTTGCCATCTGCGACATATGGGATCCATTATTGCGGCTAACAAAAAATTAACTAACAAAATATCCTTGAAAGTGAAGAATGCTGGACCTCTTCCTTTGTCATCTTTGGCTTCTTTTTGCTCTTGCTCGGTTCTGGATTTGATGTTGCCCTAACTTGAAATGAAGGGCAAATCGGATCCCCAACTTACAGTCCATTTGGGTCAGATCATGACTATCATTGATCATCTCCGACATGCATGATCATCTCCTAAGTCATCATGGTTTGCAAGCATTAATCATGTCCCAAATGAACTAATAATGGTTTGTGCAAAACATTAATCATGACATATGCAAATTTGACCGGAAAGGAGCAAAAAGAACTCATCGAGTCTTGTTGGGTCACTCCGTTGAACAGGTCGTGCACAACTCGTGCGATGCCGGTCCCCATGCTTGTCCGCGTCTGAACGAGCTTTGACGACTAACATACATCCACCGACGACATCTGCGTTGGCGAAAAGCTCTTCGAAATTCCTCAAACGGCCGTCAGAACCCCCTCTGTCCCAACCTGGTCCAACAATGCGATCCTTGTCAACGGCTTGATGGACGGGGTGCAGGGTCCTGGGTGCGACGACGGGGGAGGGGGGAGGGTCACTTGGAAGCAGTATTTGGGTACAAATAGAATCAACAGTCCTCTTTTATTGACAGAAAATCACACTATCCCACAGCGAGCTTTCTCTTTCAAGGCACGTTGCTGTCACAACTCGCAAGAGAGCCCCGGAGCACAGCAGACTGACCCAACCAGCCGGGCCCTCATCCGTCCCATGCCTGCGCGTCGCGTGGGTCTCCATCCGCACCCTCTCGAGCTCCAGCTGGTTCCGACCCAGTTAACGCCCCCGAGCCCAACACCGTCCCGACCCATCAAGTCGCGCTACCTGGCTGGCGCCTCCGGTGGCACACCGCGTTGAGCCGTGCACGCGGGGACGTCGCGCTCCACGCGCTAAACTATATAAACCGCCACCCTTCCCTTCCAAATCCAAACCACCTGCTTCCACTCCCTCCCCCTGCTTACACTCCAGCAGCCAGCATAGCCAGGCAGCCATGGACGTTGGTGCCCTCAGCAGCGACTACTCGTCGGGGACGCCGTCCCCGGTGGGCGCGGACGGCGGCAACAGTGAGGGCTTCTCGACGTACATGACGGTGTCTTCGGCTCCGCCGAAGCGGCGCGCGGGGCGGACCAAGTTCAAGGAGACGCGGCACCCGGTCTACAAGGGCGTGCGCCGGAGGAACCCCGGGAGGTGGGTCTGCGAGGTGCGGGAGCCGCACAGCAAGCAGAGGATATGGCTCGGCACGTTCGAGACCGCAGAGATGGCGGCGCGCGCGCACGACGTGGCCGCGCTGGCGCTGCGCGGCCGCGCCGCCTGCCTCAACTTCGCCGACTCTCCTCGTCGGCTCCGGGTCCCGGCCGTGGGTGCCAGCCCTGATGAGATACGGCGGGCGGCGGTAGAGGCGGCTGAGGCATTCCTGCCGGCACCCGACCAGAGCAATGCGCCCGCCGAGGAGGTAGCCGCTGCACCAACGATGCAGTTCGCCGGTGATCCGTACTACGGGATGGACGATGGTATGGACTTCGGGATGCAGGGCTACCTCGACATGGCGCAGGGGATGCTCATTGCCCCTCCTCCGTTGGTAGGTCCGTCAGCGACTGCCGGAGACGGCGACGATGACGGTGAGGTCAGCTTGTGGAGCTACTGATTACGCGCATTTGAGTTATGCCATGTGTGGTGCGACGAACTACTAATGCATGTGCAGCTTTATATAGTAATCGAGTAGAGGAAGTGTACTGCATGGTTGCCCTTTCCTTTTCTCCACTTTGTTCTAAACTTCTCTGGGTAATGTACGTTTGCTCTCTGATGAACTGCGATCAGAAGAAGCAGAGGGCCTGATCTTGAGTGTAGTGTTCGAGCTCCGAAGGAGTGTTGGGGCTAGCTATATGTATCTCCCTTGTTTATTATGCATGAGCGGAGTGTCTTCGTCGGTGTTTGGTTAAGCAAAATGTAAGTTTCTCGGTGTATAATTAGCAATATAGCACGTTGCCTTAGAGCATCTCCAGCCGTTTAGTTCCTAACGCATCCGGCAAATGCCATTTTGGC

>HvDREB1.2

>Protein

MEWACCGSGYSSSGTQSPAAGDGEEGSYMTVSSAPPKRRAGRTKVRETRHPVYKGVRSRNPGRWVCEVREPQGKQRLWLGTFDTAEMAARAHDVAAMALRGRAACLNFADSPRRLPVPPQGAGHDEIRRAAVEAAELFRPAPGQRNAATVAAATAPPVALGNAELVADSPYYPMDGLESEMQGYLDMAHGMLIEPPPMAWPSTWIEEDYDCEISLWNY

>cDNA

GAGGTACCAAGGCAGGCATGCAAAAAGTAACGGAAAAACAATCTCCAACGGACTGATGGAGTGGGCGTGCTGCGGCAGCGGCTACTCGTCGTCGGGAACGCAGTCCCCGGCGGCCGGCGACGGGGAGGAGGGCTCGTACATGACGGTGTCGTCGGCGCCGCCCAAGCGGCGGGCCGGGAGGACCAAGGTCAGGGAGACGAGGCACCCGGTGTACAAGGGGGTGCGCAGCAGGAACCCCGGGCGGTGGGTCTGCGAGGTGCGCGAGCCGCAAGGGAAGCAGAGGCTATGGCTCGGCACCTTCGACACCGCCGAGATGGCGGCGCGCGCGCACGACGTCGCCGCCATGGCGCTCCGCGGCCGCGCCGCGTGCCTCAACTTCGCGGACTCGCCGCGGAGGCTCCCCGTGCCACCGCAGGGGGCGGGCCACGACGAGATACGACGAGCCGCTGTCGAAGCGGCCGAACTGTTCCGCCCGGCGCCTGGGCAGCGCAATGCAGCTACCGTGGCGGCAGCGACTGCTCCGCCGGTGGCCTTGGGAAACGCCGAGCTCGTCGCAGACTCTCCTTACTACCCCATGGACGGGTTAGAATCCGAAATGCAGGGCTATCTTGACATGGCGCACGGCATGCTCATCGAGCCACCACCAATGGCGTGGCCGTCGACGTGGATCGAGGAGGACTACGACTGCGAGATCAGCCTGTGGAACTACTGATGGCGCGCACCATAGCCGGCCCGACCCTGGCGTTCACGACTCACTGTACATACATCTTATTATGGTCATCCAGGACAAACCTGTACTTGTGGTGTGATGGTGTGTGATGTTTTCCTGCTATTCTGATGAAAATGTCATTGATAGTG

>CDS

ATGGAGTGGGCGTGCTGCGGCAGCGGCTACTCGTCGTCGGGAACGCAGTCCCCGGCGGCCGGCGACGGGGAGGAGGGCTCGTACATGACGGTGTCGTCGGCGCCGCCCAAGCGGCGGGCCGGGAGGACCAAGGTCAGGGAGACGAGGCACCCGGTGTACAAGGGGGTGCGCAGCAGGAACCCCGGGCGGTGGGTCTGCGAGGTGCGCGAGCCGCAAGGGAAGCAGAGGCTATGGCTCGGCACCTTCGACACCGCCGAGATGGCGGCGCGCGCGCACGACGTCGCCGCCATGGCGCTCCGCGGCCGCGCCGCGTGCCTCAACTTCGCGGACTCGCCGCGGAGGCTCCCCGTGCCACCGCAGGGGGCGGGCCACGACGAGATACGACGAGCCGCTGTCGAAGCGGCCGAACTGTTCCGCCCGGCGCCTGGGCAGCGCAATGCAGCTACCGTGGCGGCAGCGACTGCTCCGCCGGTGGCCTTGGGAAACGCCGAGCTCGTCGCAGACTCTCCTTACTACCCCATGGACGGGTTAGAATCCGAAATGCAGGGCTATCTTGACATGGCGCACGGCATGCTCATCGAGCCACCACCAATGGCGTGGCCGTCGACGTGGATCGAGGAGGACTACGACTGCGAGATCAGCCTGTGGAACTACTGA

>DNA

AGTCTTGAGCCATGTCAAGATGAAGAATGACATGCAAAGCCATGTGAAGCTGAATTTTTTGCATCGGAGAATAGTTGGCTGCTCCAACATTTTAGCATGTGGGCTGTTTTGTGCAGTTTGTGATATAAAATGTGGTAGGGCTCAACATGATCAGTTGTCTTTGTTCAAGCTTCCTGCACTGCCGTCTTGAATCCACGCATTGAAATCCAGAAGTTTTCAAACCGAAGCCACCGCTATTTTTGATGCTCTCGTGCCACCTACCACTAGTAGCCGGCCATCAGTCCTGGCGCTCCTAGTCAGGCGCGTCGTACAAACTGACATGCCCATCTCCAAGCCGAAGAGGTGCATGGCCTCAATAACAAATCGAGGCCGACCAGTTCAGGCCTCTCTGAAAACGTGGGCAGTAGTAGAATCAAGGCCAATGATACTAGTAGTCCAGTACTGCTACTCAGGTCCCGTGTATGGAGTACTTACAACTAATACGGGTCCTGTGTGATGTGCGTTCCGTCCCGAAATACTCCAGGCAACCAGCATGCGAGGTCAGGTGCGACGTTAAACTCACCGAAGCCACCGTCTGTTTCCCCGAGCAGCGGAAGTGCAGCTCAGCTGTATCCATTTTCTCTTGACAGTTACGTGCGCGTGAGACCCAGTCCACACAAGAGTTGCTCTATCCCTCAAAAGCGGGGCATGCACTTGTTAAGGATATTTTTGTAACCAGCTGAAAATGGCGTCACGTCGTCCTTGTCTCCTCAACTGAAAACCGCAAATTCCACCGAGGGGCACGCGCACTCTCCAACTTACACAAGTCCGCATCAGCCACTTTAATGCCAGCTCAAGTTTGGAGAAAGCATCGCAGCCCCTAACCCTTTTTCTCTTCGAGATGTATCGCAGTCCCTCACCGTGCGGTTTCTCTCAGCACACGTGAGTGACACATCCACATCACAACTCCTCAAATAGTGTAGCAGCTCCAAACCCAGAAACGAATAGAAGAGAAAAGCCCAGCAGGCCACAAGCCGAGATGGCCCATTGCAGCGCGTCGCGGGCGTCACCGTCGCCTTCCGTGCAATCTCCAGCTGGTCTGACAAGTAGCTGAAACGCACCCCACCTAACCAACCGCCGGGTCGCACATCGCGCTTCACTTCACTCCGGCTGGCTCCTAGTGTCACCCCGCGTTTGCAGCAGCGCGCCGCACGCGTTCCTCCGCCTCCCGTCCATATGCGTAGCTATATAAATAGCCGCCGGATATCTTCCCTCCCAAACTCGTACCAAGGCAGGCATGCAAAAAGTAACGGAAAAACAATCTCCAACGGACTGATGGAGTGGGCGTGCTGCGGCAGCGGCTACTCGTCGTCGGGAACGCAGTCCCCGGCGGCCGGCGACGGGGAGGAGGGCTCGTACATGACGGTGTCGTCGGCGCCGCCCAAGCGGCGGGCCGGGAGGACCAAGGTCAGGGAGACGAGGCACCCGGTGTACAAGGGGGTGCGCAGCAGGAACCCCGGGCGGTGGGTCTGCGAGGTGCGCGAGCCGCAAGGGAAGCAGAGGCTGTGACTCGGCACCTTCGACACCGCCGAGATGGCGGCGCGCGCGCACGACGTCGCCGCCATGGCGCTCCGCGGCCGCGCCGCGTGCCTCAACTTCGCGGACTCGCCGCGGAGGCTCCCCGTGCCACCGCAGGGGGCGGGCCACGACGAGATACGACGAGCCGCTGTCGAAGCGGCCGAACTGTTCCGCCCGGCGCCTGGGCAGCGCAATGCAGCTACCGTGGCGGCAGCGACTGCTCCGCCGGTGGCCTTGGGAAACGCCGAGCTCGTCGCAGACTCTCCTTACTACCCCATGGACGGGTTAGAATCCGAAATGCAGGGCTATCTTGACATGGCGCACGGCATGCTCATCGAGCCACCACCAATGGCGTGGCCGTCGACGTGGATCGAGGAGGACTACGACTGCGAGATCAGCCTGTGGAACTACTGATGGCGCGCACCATAGCCGGCCCGACCCTGGCGTTCACGACTCACTGTACATACATCTTATTATGGTCATCCAGGACAAACCTGTACTTGTGGTGTGATGGTGTGTGATGTTTTCCTGCTATTCTGATGAAAATGTCATTGATAGTG

>HvDREB1.3

>Protein

MDHCGVGLYGEYATVTSAPPKRPAGRTKFRETRHPVYRGVRRRGAAGRWVCEVREPNKKSRIWLGTFATPEAAARAHDVAALALRGRAACLNFADSAALLRVDPATLRTPEDIRAAAMALAQAACPHDAASSSAPALKAASAPAPAMVMVQEAAAAPYDSYATALYGDLTDLDMHSYYCYDGMSGGGDWQSISRMDGADEDGIYGAGDVALWSY

>cDNA

GCTAGCCAGCTGCAGATAAGCGAATCCATCGACCAAtCGATACCtTACAAGATGGACCACTGCGGCGTGGGCCTCTATGGCGAGTACGCGACGGTGACGTCGGCGCCGCCCAAGCGGCCGGCGGGGCGGACCAAGTTCAGGGAGACGCGGCACCCGGTGTACCGGGGCGTGCGGCGGCGCGGCGCCGCGGGGCGGTGGGTCTGCGAGGTGCGCGAGCCCAACAAGAAGTCCCGCATCTGGCTCGGCACCTTTGCCACGCCCGAGGCCGCCGCGCGCGCCCACGACGTCGCCGCGCTCGCGCTCCGGGGCCGCGCCGCCTGCCTCAACTTCGCCGACTCCGCGGCCCTGCTCCGCGTCGACCCGGCCACGCTCCGCACTCCCGAGGACATCCGAGCAGCCGCCATGGCGCTCGCCCAGGCCGCCTGCCCCCACGACGCCGCGTCCTCCTCTGCCCCCGCCCTGAAGGCGGCCTCTGCGCCGGCGCCAGCGATGGTGATGGTGCAGGAGGCCGCGGCGGCGCCGTACGACAGCTATGCCACGGCCCTGTACGGCGACTTGACGGACCTGGACATGCATTCCTACTACTGCTACGACGGGATGAGCGGCGGCGGCGACTGGCAGAGCATCTCGCGTATGGACGGAGCCGACGAAGACGGCATCTACGGCGCAGGAGACGTCGCGCTCTGGAGCTACTGATTGTCTGAGATTGATCGGCCGGTTTGTTCAGCTACCGGGTCCTGCTCAAGTGCTGCTGCGCAACGTCGTCATACGATGAAGAAGATGAAACAGAGTTCCTGGGGAAGAAAGATCCATGCCAGCCGCAGGACATGTCACAGCACCACCGGCAGGCCGGCGCCGCCTCCTCCGCGTGCCCATGCAATCTGTCGATCGGCAGGGCGTACTTCTTCTGGGAGGTCACGTGGTCAGAACGGACTGAGATTTGAGCGAAGTGTTGCCTGAACGCACGACATACTTGCATCAGCGAGTTTCAAGCACGCCTACTCTGCTTCCCCTGTTCTTCAAAAGTGGCGCTAAAACTGCACAATCCCACATGAGCTAGTTTTCGTAGGGTATAGTGCTAGCAAAAATATGCACACCCAGTTTGCTGAGTGATTATACAACCAGAAGACCTCACTCCAATTTATTTTTCCCTTCTATGTAAATACTCCCTCTG

>CDS

ATGGACCACTGCGGCGTGGGCCTCTATGGCGAGTACGCGACGGTGACGTCGGCGCCGCCCAAGCGGCCGGCGGGGCGGACCAAGTTCAGGGAGACGCGGCACCCGGTGTACCGGGGCGTGCGGCGGCGCGGCGCCGCGGGGCGGTGGGTCTGCGAGGTGCGCGAGCCCAACAAGAAGTCCCGCATCTGGCTCGGCACCTTTGCCACGCCCGAGGCCGCCGCGCGCGCCCACGACGTCGCCGCGCTCGCGCTCCGGGGCCGCGCCGCCTGCCTCAACTTCGCCGACTCCGCGGCCCTGCTCCGCGTCGACCCGGCCACGCTCCGCACTCCCGAGGACATCCGAGCAGCCGCCATGGCGCTCGCCCAGGCCGCCTGCCCCCACGACGCCGCGTCCTCCTCTGCCCCCGCCCTGAAGGCGGCCTCTGCGCCGGCGCCAGCGATGGTGATGGTGCAGGAGGCCGCGGCGGCGCCGTACGACAGCTATGCCACGGCCCTGTACGGCGACTTGACGGACCTGGACATGCATTCCTACTACTGCTACGACGGGATGAGCGGCGGCGGCGACTGGCAGAGCATCTCGCGTATGGACGGAGCCGACGAAGACGGCATCTACGGCGCAGGAGACGTCGCGCTCTGGAGCTACTGA

>DNA

GCCCCACCGCCGTTGATGCCCGCCACGCCACTTGCACTAGTGTGCCGCCCCCGCCACCCGCACGACGCCACTCCGTCGCACCCACCACCGCGTGACGTTCCGGCCCAGGGGAGCACGCCTGCGCATCCCCCCTCGAGCAGGAAGACGCTAGGGCCGCGCCGCTGCCGGCATCGGTCGGGCTATGGCCGACAATGCCCTCTGACGACGGCGAAGCAGGGGGGACGGGACGGGCGCTCGACGCGATCTAGGTCGTCCTCGTGGTTGCCGGGCGACACAGGAGGGGGGAGAAAGTTCTTTCTCTACTTATTTTCCCTCCTAATTGAGGTCTGATCGGGAGAAACTATGTCAAAGCATCAAAACAGTGAAATCAGTTTCCGCGGGGCACCCCGTGTCAGCCCGGCAACCTCGCCACGTATCAGAAAGCACGCAAAATGGGCAAGGCCTGCGCCGCGAGGAGAATCGTCATTATCGCCGGCCGGACACCGTCCCACGCAGCCGCTCTGCGGGACAGAGCGCTAGTACTGTACTAGGATAGCGGCGAGCCGTGGACGCGTCCGGCGTCCGGCGCCAGGGCACGAGCCGAGCCGCCCATCCGGTCCTGCCTCACGTGCTTCCCTTGAGATACGGAGCTCCAGCGTGCTGGTACTAGTATAGTAGTAGGAGTACACCGTAGTGTGACGCACTGCCGCGGTACGGTGCCGGCCGCCCAAGACCGACGGAAAAGCCCGTCTCCACGTGCCTCCTCCCCCTTCCGGTCTCCCTCCTTCCTCCGCTTCGCTTCAGGGAAAGCGTACAAGTCCCAACTCTTCTAGTTGCAGGTCGGGATGAGATTGCGACGTACGGACGACTGCTTTGTCCCGAGCCAAGCAATGGCCACCACGCCAGCACGGCTTCCCCTTCCTTCCGCCACCATGTCCAGCTCCCCCTCCCCGTCTCCCGCCACGCGCCGCCCCTTCCAACCCGCGTCCCTTTCTCTGCCCCCGCCGGCCGCCCGAACCCTATATATACATACATGTCGTCGCTCTCCTTCCTCCCTCCCTCCCGACCATACGACCTCGATCACAAAACCATTGCTAGCCAGCTGCAGATAAGCGAATCCATCGACCAATCGATACCTTACAAGATGGACCACTGCGGCGTGGGCCTCTATGGCGAGTACGCGACGGTGACGTCGGCGCCGCCCAAGCGGCCGGCGGGGCGGACCAAGTTCAGGGAGACGCGGCACCCGGTGTACCGGGGCGTGCGGCGGCGCGGCGCCGCGGGGCGGTGGGTCTGCGAGGTGCGCGAGCCCAACAAGAAGTCCCGCATCTGGCTCGGCACCTTTGCCACGCCCGAGGCCGCCGCGCGCGCCCACGACGTCGCCGCGCTCGCGCTCCGGGGCCGCGCCGCCTGCCTCAACTTCGCCGACTCCGCGGCCCTGCTCCGCGTCGACCCGGCCACGCTCCGCACTCCCGAGGACATCCGAGCAGCCGCCATGGCGCTCGCCCAGGCCGCCTGCCCCCACGACGCCGCGTCCTCCTCTGCCCCCGCCCTGAAGGCGGCCTCTGCGCCGGCGCCAGCGATGGTGATGGTGCAGGAGGCCGCGGCGGCGCCGTACGACAGCTATGCCACGGCCCTGTACGGCGACTTGACGGACCTGGACATGCATTCCTACTACTGCTACGACGGGATGAGCGGCGGCGGCGACTGGCAGAGCATCTCGCGTATGGACGGAGCCGACGAAGACGGCATCTACGGCGCAGGAGACGTCGCGCTCTGGAGCTACTGATTGTCTGAGATTGATCGGCCGGTTTGTTCAGCTACCGGGTCCTGCTCAAGTGCTGCTGCGCAACGTCGTCATACGATGAAGAAGATGAAACAGAGTTCCTGGGGAAGAAAGATCCATGCCAGCCGCAGGACATGTCACAGCACCACCGGCAGGCCGGCGCCGCCTCCTCCGCGTGCCCATGCAATCTGTCGATCGGCAGGGCGTACTTCTTCTGGGAGGTCACGTGGTCAGAACGGACTGAGATTTGAGCGAAGTGTTGCCTGAACGCACGACATACTTGCATCAGCGAGTTTCAAGCACGCCTACTCTGCTTCCCCTGTTCTTCAAAAGTGGCGCTAAAACTGCACAATCCCACATGAGCTAGTTTTCGTAGGGTATAGTGCTAGCAAAAATATGCACACCCAGTTTGCTGAGTGATTATACAACCAGAAGACCTCACTCCAATTTATTTTTCCCTTCTATGTAAATACTCCCTCTG

>HvDREB1.4

>Protein

MDQYNNYGGVAYYAGGGGDDNGQGGYATVTSAPPKRPAGRTKFRETRHPVYRGVRRRGAAGRWVCEVREPNKKSRIWLGTFANPEAAARAHDVAALALRGRAACLNFADSAALLAVDPATLRTPDDIRAAAIALAETACPSAPAPSSSSSPSVAASASATAPAMMAMMQESATLQYDDYAMQYGGIADLDQHSYYYDGMSAGGGDWQSGSHMDGDDDCNGGSGYGAGEVALWSY

>cDNA

AAAAGTCTCACCCAAGCAAGCACCCAGCATCACCTCACTCGCCAGTCACCACCATTGCCACAGACCTTGACACCAGCTAGATACCACCTCGTCTCGTGCCATGGACCAGTACAACAACTACGGCGGCGTGGCCTACTACGCCGGCGGCGGCGGGGACGACAATGGCCAGGGCGGGTACGCGACGGTGACGTCGGCGCCGCCGAAGCGGCCGGCGGGGCGGACCAAGTTCAGGGAGACGCGCCATCCGGTGTACCGCGGCGTGCGCCGGCGCGGCGCCGCGGGGCGGTGGGTCTGCGAGGTGCGCGAGCCCAACAAGAAGTCCCGCATCTGGCTCGGCACCTTCGCCAACCCGGAGGCCGCGGCCCGCGCCCACGACGTCGCCGCGCTCGCGCTCCGTGGCCGCGCCGCATGCCTCAACTTCGCCGACTCGGCCGCCCTGCTCGCCGTCGACCCCGCCACGCTCCGCACCCCCGACGACATCCGCGCGGCCGCCATCGCGCTCGCCGAGACGGCGTGCCCCTCCGCGCCCGCGCCGTCGTCGTCGTCGTCGCCGTCCGTGGCAGCCTCGGCGTCCGCGACGGCGCCCGCGATGATGGCGATGATGCAGGAGTCCGCGACGCTGCAGTACGACGACTACGCGATGCAGTACGGCGGCATTGCGGACCTGGACCAGCATTCCTACTACTACGACGGGATGAGCGCCGGCGGCGGCGACTGGCAGAGCGGCTCGCACATGGACGGAGACGACGACTGCAACGGCGGCTCGGGGTACGGTGCCGGCGAGGTCGCGCTCTGGAGCTACTGATCGAGCTGGTCCATTGGTCGCTCCAGTTAGCGTAGGATTAAGAAGAAGCAAAGCAGAGTTGGCCGGGAGCTTCATCAGTTGTGAAGTAGCACTAGTCGTATTATTTATGTGGCCGTAAAGCAGAGAGAGATAGAGAGAGAGAACGCTATGCAAATTGCATCAGCCTGTTTCCCTCCATAATTTTTTTATTTTCTGATGATTTTTTGGTTGGCTGGAATTTTTGTAACATGTGCTGGAGTAGAGTAATAAGGGACAAACTCTGCTTTATC

>CDS

ATGGACCAGTACAACAACTACGGCGGCGTGGCCTACTACGCCGGCGGCGGCGGGGACGACAATGGCCAGGGCGGGTACGCGACGGTGACGTCGGCGCCGCCGAAGCGGCCGGCGGGGCGGACCAAGTTCAGGGAGACGCGCCATCCGGTGTACCGCGGCGTGCGCCGGCGCGGCGCCGCGGGGCGGTGGGTCTGCGAGGTGCGCGAGCCCAACAAGAAGTCCCGCATCTGGCTCGGCACCTTCGCCAACCCGGAGGCCGCGGCCCGCGCCCACGACGTCGCCGCGCTCGCGCTCCGTGGCCGCGCCGCATGCCTCAACTTCGCCGACTCGGCCGCCCTGCTCGCCGTCGACCCCGCCACGCTCCGCACCCCCGACGACATCCGCGCGGCCGCCATCGCGCTCGCCGAGACGGCGTGCCCCTCCGCGCCCGCGCCGTCGTCGTCGTCGTCGCCGTCCGTGGCAGCCTCGGCGTCCGCGACGGCGCCCGCGATGATGGCGATGATGCAGGAGTCCGCGACGCTGCAGTACGACGACTACGCGATGCAGTACGGCGGCATTGCGGACCTGGACCAGCATTCCTACTACTACGACGGGATGAGCGCCGGCGGCGGCGACTGGCAGAGCGGCTCGCACATGGACGGAGACGACGACTGCAACGGCGGCTCGGGGTACGGTGCCGGCGAGGTCGCGCTCTGGAGCTACTGA

>DNA

TACTTACAGAATGAAATTTTCCATGGCAAACCGGTACCCCCGTTTGATATCTCTTGTTCCTTCCTTGCAAGCTACTATAATTCTTACAAGAACATATCTCTAAGCGTGGAGGATATGGTGAAAGGCAAATCTCCAGTGATTGAGCTAATCCCTCCAAAACCATCATCCATGAGTACTGTGGCTAAGAAGTGACCGGCCCCGGAAGCTAATATGTTGGGCTTATCTACTGATGGCTCTTTCAACCTTGATGATGGCACTGCCGGTTCGGGGATGATCCTTAAGGATAACAAAGGGGATGTCATCTTTGCGTCTTACCAAAATTGTTCCAATGCAACGATGCACTTGAGGCGGAGCTACAAGCAATAAAAGAAGGGCTACAACTGGCTTTCATTCATGCTTCGTTACCTGTGGTGCTGCAATCCGATTCTGTTGTGGCTCTAAAGATGAAAGCTATTAGTAATTATGATAGATCACCTTATGGTGTCCTAGTGATAGAAATTAAGAGTTTACTTTTGAATAGGGANNTTTCTTTCCTTAAGATTAGTCGTGAACAAAATAGAGTTGCACATTGCTTAGCAAACTTTGGTCATAGTGGTGATAGCACTGCATGTTGGTTGGCTAGACCACCTCCATGTATCACTGTGCTGATCGCTGAGGATTGTAACTCTGTTACAGTTTAATAAAATCTCTCTGTTCCTCGTAAAAAAAAGTCTTGCATATTACAAGAGCTTCAACCACTCGTTTGTCTGATCAAGCGCGTGTTTTCTTACGACAAAAATAGAGTAAAAAATAGAACGGCAATCCGCCTTGGGGACCCGCGACCCACCCCCGCAGTGACGAGTGACGCCCCGTTCAATCAAAGGCAGGCTTGAGTCCCGGCACGTGGAGTCCCATCCTAGCAACGCGTGTCTCCTCCTCGCAACAAAAAAAAGCGTGTCCCTTCTTCTTTTTTTCGGGAAAAAGCGTGTCCCTTCAATCGACAACGAATCGGACATACGGACACGTGGTGGATTGGACTGTTTTCCTTTTTTTTTCCCTTTTTGATGATGAAGCTCAGCTGATTGGTTTGTTGACGAGAGGTGATCCTGTGGTGTCAGCTGGGCGAAGCCACGTACGTCCTAGGTGGTCTTCGCCGTGGCTATGGACGAGGCTAGGAGCGATGGAGTCAGTCACCATCCTTTTCAGTTTAGCTATAGGACGCAGGAGCCACCGTCCCACGCAGCTACGAGCGTGGACGCGTCCCAGCCTGCGTTTGCGCGGGCGGCGCGAGGGCACGCGCCGGCCATCCGTCCTGTCCCCGCGCGGTCCGGCGGCGGGTCGCCGCAGCACCGCCTTATCACGTGCCCACGCTCCAGGCATGTGGCTGTGGTGTGAACGGGCGTCCCCCATAAGAGCGAGCGGCCATGCGTCGGGCCTGTCCATGGCGGCAACCATCACGTCATGCTTCGTTTCCAGCGTATACAGCGATTTGGAGGAATTTTAATTTTAATAATAAAGCAATCATAAAACCAGTATACAGAAGCATGACGCACGACTGTGGCAAGGTACGGGCAGCAGCACCGGAAAAGGCAGCCCCCGTCGCCATCTCCACGTGGCCCTCCCCTCCCCCCTTCCCATCTCCTTCCAGAAATAGATATCCACAAGATGTTTTCCCCGACCTCGATGCATCCCTCCCGGGCGAAGCAAACTCCGATGAGGCCAACACGCTTTCCCCATCCATCCATCCATCCATCCATCCGTCCACCCGTCTCGCGGCCTCTGCTCCCCACGCCATGGACACAGGTCCAGCTCACACCTCACTCCCGCCACGCGCCGCCCCCTACCCCCTGATTCAAACCCGCCTTTGATCTCTCTCTCCCCCCGAATCCTATAATACATCCGAACATCCTCGCTCCCATTCCAAAGTCTCACCCAAGCAAGCACCCAGCATCACCTCACTCGCCAGTCACCACCATTGCCACAGACCTTGACACCAGCTAGATACCACCTCGTCTCGTGCCATGGACCAGTACAACAACTACGGCGGCGTGGCCTACTACGCCGGCGGCGGCGGGGACGACAATGGCCAGGGCGGGTACGCGACGGTGACGTCGGCGCCGCCGAAGCGGCCGGCGGGGCGGACCAAGTTCAGGGAGACGCGCCATCCGGTGTACCGCGGCGTGCGCCGGCGCGGCGCCGCGGGGCGGTGGGTCTGCGAGGTGCGCGAGCCCAACAAGAAGTCCCGCATCTGGCTCGGCACCTTCGCCAACCCGGAGGCCGCGGCCCGCGCCCACGACGTCGCCGCGCTCGCGCTCCGTGGCCGCGCCGCATGCCTCAACTTCGCCGACTCGGCCGCCCTGCTCGCCGTCGACCCCGCCACGCTCCGCACCCCCGACGACATCCGCGCGGCCGCCATCGCGCTCGCCGAGACGGCGTGCCCCTCCGCGCCCGCGCCGTCGTCGTCGTCGTCGCCGTCCGTGGCAGCCTCGGCGTCCGCGACGGCGCCCGCGATGATGGCGATGATGCAGGAGTCCGCGACGCTGCAGTACGACGACTACGCGATGCAGTACGGCGGCATTGCGGACCTGGACCAGCATTCCTACTACTACGACGGGATGAGCGCCGGCGGCGGCGACTGGCAGAGCGGCTCGCACATGGACGGAGACGACGACTGCAACGGCGGCTCGGGGTACGGTGCCGGCGAGGTCGCGCTCTGGAGCTACTGATCGAGCTGGTCCATTGGTCGCTCCAGTTAGCGTAGGATTAAGAAGAAGCAAAGCAGAGTTGGCCGGGAGCTTCATCAGTTGTGAAGTAGCACTAGTCGTATTATTTATGTGGCCGTAAAGCAGAGAGAGATAGAGAGAGAGAACGCTATGCAAATTGCATCAGCCTGTTTCCCTCCATAATTTTTTTATTTTCTGATGATTTTTTGGTTGGCTGGAATTTTTGTAACATGTGCTGGAGTAGAGTAATAAGGGACAAACTCTGCTTTATC

>HvDREB1.5

>Protein

MDVGIDCWISSPSSSTSGHELGVAVPVWSPAAKRPAGRTKFKETRHPVYRGVRRRGSAGRWVCEVRVPGKRGERIWLGTYVAAESAARAHDAAMLALLGRSTSAAACLNFPDSAWLLVLPPKLTDLADVRRAATEAVAGFLRMEAAVVPILDEATSPVYLPSSVDNADDVFQVPAFSAQGSDMFELDMSGEMDLDAYYAGFAQGMLLEPPPTPSHWENGECGDGAAAAGLWSY

>cDNA

CTCAACTTACACAACACCCAAGCACACTCCAAGCACCGCACTCAGCTACGCAGCACGTACCTCAAGCTGCTCAGGCTCAGACTCAGGCTCAGACTCAGGCTCAGCTACAGTGACCACATTCCGATTCCACCAAGCGATCGGCACTCCGGCAGCTAGCAATGGACGTGGGTATCGACTGCTGGATCAGCTCCCCTTCCTCATCTACGTCCGGGCACGAGCTCGGGGTGGCGGTGCCTGTGTGGTCGCCGGCGGCGAAGCGGCCCGCGGGGCGCACCAAGTTCAAGGAGACGCGGCACCCGGTGTACCGCGGCGTGCGGCGCCGGGGCAGCGCGGGGCGGTGGGTGTGCGAGGTGCGCGTCCCCGGCAAGCGCGGCGAGCGGATCTGGCTCGGGACCTACGTCGCCGCCGAGTCCGCCGCGCGCGCGCACGACGCCGCCATGCTCGCGCTGCTCGGACGCTCCACCTCCGCCGCGGCGTGCCTCAACTTTCCGGACTCCGCGTGGCTGCTCGTCCTTCCCCCGAAGCTCACCGACCTGGCCGACGTCCGGCGCGCGGCCACCGAGGCCGTCGCGGGCTTCCTGCGCATGGAGGCCGCCGTCGTCCCCATCCTGGACGAGGCCACCTCCCCCGTGTACCTGCCGTCGTCCGTGGACAATGCCGACGACGTGTTTCAGGTTCCGGCTTTCTCCGCGCAGGGCAGCGACATGTTCGAGCTCGACATGTCCGGGGAAATGGACCTGGACGCGTACTACGCGGGCTTTGCCCAGGGGATGCTCCTGGAGCCGCCGCCCACGCCGTCGCACTGGGAGAACGGAGAATGCGGCGACGGCGCAGCGGCCGCCGGCCTCTGGAGCTACTGATGCCTGTAACCTCCAGTGGGTTCACACTGAGGATTTTGCTATCCCGATTGGGCCAACTGAAAACTGCCTCAAGCATATTGTTTGGAACGTCGACCAGTTTTGTCAATTCTCTGTTTGAATCATCGATCAAGTGTTTT

>CDS

ATGGACGTGGGTATCGACTGCTGGATCAGCTCCCCTTCCTCATCTACGTCCGGGCACGAGCTCGGGGTGGCGGTGCCTGTGTGGTCGCCGGCGGCGAAGCGGCCCGCGGGGCGCACCAAGTTCAAGGAGACGCGGCACCCGGTGTACCGCGGCGTGCGGCGCCGGGGCAGCGCGGGGCGGTGGGTGTGCGAGGTGCGCGTCCCCGGCAAGCGCGGCGAGCGGATCTGGCTCGGGACCTACGTCGCCGCCGAGTCCGCCGCGCGCGCGCACGACGCCGCCATGCTCGCGCTGCTCGGACGCTCCACCTCCGCCGCGGCGTGCCTCAACTTTCCGGACTCCGCGTGGCTGCTCGTCCTTCCCCCGAAGCTCACCGACCTGGCCGACGTCCGGCGCGCGGCCACCGAGGCCGTCGCGGGCTTCCTGCGCATGGAGGCCGCCGTCGTCCCCATCCTGGACGAGGCCACCTCCCCCGTGTACCTGCCGTCGTCCGTGGACAATGCCGACGACGTGTTTCAGGTTCCGGCTTTCTCCGCGCAGGGCAGCGACATGTTCGAGCTCGACATGTCCGGGGAAATGGACCTGGACGCGTACTACGCGGGCTTTGCCCAGGGGATGCTCCTGGAGCCGCCGCCCACGCCGTCGCACTGGGAGAACGGAGAATGCGGCGACGGCGCAGCGGCCGCCGGCCTCTGGAGCTACTGA

>DNA

ATTCTCAACCCTTAAAACTGATTTTAGATTTAAGGGTTCGAGGGTTCTGCTAGAGATGCTCTTAGCTCCGTCGACATGTCATCCTTGTACAGAATTCATCGCTGTGGAACCCAAGCGCCGCAGCGCCAATCGTCGATCAGATGCACGAGCGAAAATATCAGGCGCATTCTGATTCCATGTTAGCTGCCGCGTGCCCCCTCGCCGACGAGCGCGCTGCGTCACGCATAAGTATAAATGAGCGCACTCTGCACGCCACACCCTCAACTTACACAACACCCAAGCACACTCCAAGCACCGCACTCAGCTACGCAGCACGTACCTCAAGCTGCTCAGGCTCAGACTCAGGCTCAGACTCAGGCTCAGCTACAGTGACCACATTCCGATTCCACCAAGCGATCGGCACTCCGGCAGCTAGCAATGGACGTGGGTATCGACTGCTGGATCAGCTCCCCTTCCTCATCTACGTCCGGGCACGAGCTCGGGGTGGCGGTGCCTGTGTGGTCGCCGGCGGCGAAGCGGCCCGCGGGGCGCACCAAGTTCAAGGAGACGCGGCACCCGGTGTACCGCGGCGTGCGGCGCCGGGGCAGCGCGGGGCGGTGGGTGTGCGAGGTGCGCGTCCCCGGCAAGCGCGGCGAGCGGATCTGGCTCGGGACCTACGTCGCCGCCGAGTCCGCCGCGCGCGCGCACGACGCCGCCATGCTCGCGCTGCTCGGACGCTCCACCTCCGCCGCGGCGTGCCTCAACTTTCCGGACTCCGCGTGGCTGCTCGTCCTTCCCCCGAAGCTCACCGACCTGGCCGACGTCCGGCGCGCGGCCACCGAGGCCGTCGCGGGCTTCCTGCGCATGGAGGCCGCCGTCGTCCCCATCCTGGACGAGGCCACCTCCCCCGTGTACCTGCCGTCGTCCGTGGACAATGCCGACGACGTGTTTCAGGTTCCGGCTTTCTCCGCGCAGGGCAGCGACATGTTCGAGCTCGACATGTCCGGGGAAATGGACCTGGACGCGTACTACGCGGGCTTTGCCCAGGGGATGCTCCTGGAGCCGCCGCCCACGCCGTCGCACTGGGAGAACGGAGAATGCGGCGACGGCGCAGCGGCCGCCGGCCTCTGGAGCTACTGATGCCTGTAACCTCCAGTGGGTTCACACTGAGGATTTTGCTATCCCGATTGGGCCAACTGAAAACTGCCTCAAGCATATTGTTTGGAACGTCGACCAGTTTTGTCAATTCTCTGTTTGAATCATCGATCAAGTGTTTT

>HvDREB1.6

>Protein

MDMAGSDQQRCSPSSPSLSSHLKRPAGRTKFKETRHPVYRGVRRRGSAGRWVCEVRVPGKRGERLWLGTHLTAEAAARAHDAAMLCLLDRPAPCLNFADSVWLLAVPSALSDLADVRRAALSAVADFQRREAASGAATRAQAAAALIDEGTCSQSAQSSMENTGSSSTSSSLPSADGMLEVPATLGSNMFELDMSGEMDLDTYYAYFAEGLLLEPPQPPAAGACWDIDGGGADAALWSY

>cDNA

CTCAGCCTGCTCTCACACTCCCAGACCTCGGTCCACCGTACGTAAGCTGGAACCAGCACCGACCACCTGCAATAGCAATATGGACATGGCCGGCTCCGATCAGCAGCGGTGCTCCCCTTCCTCGCCGTCGTTGTCCTCTCATCTGAAGCGCCCCGCCGGGCGCACCAAGTTCAAGGAGACGCGTCACCCGGTGTACCGCGGCGTGCGGCGCCGCGGCAGCGCCGGCCGGTGGGTGTGCGAGGTGCGCGTCCCCGGCAAGCGAGGCGAGCGGCTCTGGCTCGGGACGCACCTTACCGCCGAGGCGGCCGCACGTGCGCACGACGCCGCCATGCTCTGCCTGCTCGACCGCCCCGCCCCGTGTCTCAACTTCGCCGATTCCGTCTGGCTCCTCGCCGTGCCGTCCGCGCTCTCCGATCTCGCTGACGTCCGGCGCGCGGCTCTCAGCGCCGTCGCGGATTTCCAGCGCCGGGAGGCCGCAAGCGGCGCCGCCACGAGGGCCCAGGCGGCGGCCGCCCTCATTGACGAGGGAACCTGTAGCCAATCCGCGCAGTCGTCCATGGAAAATACCGGCTCGTCTTCGACGTCGTCATCCCTACCTTCAGCCGACGGAATGCTTGAGGTGCCGGCCACACTGGGCAGCAACATGTTCGAGCTGGACATGTCCGGGGAAATGGACCTGGACACATACTATGCGTACTTCGCGGAGGGGCTTCTCCTGGAGCCGCCGCAACCGCCGGCCGCCGGCGCGTGCTGGGATATCGATGGCGGTGGAGCAGACGCCGCGCTCTGGAGCTACTAAAATACTCCGGCCAGCTGCTTTGACTCTATGCATCCATGGAACAAACCGAAGCTGTTCCCTGTCAGCATAATAAAATTGTGGAACTAAAATCCATCAAATACTGCGACACACCTACAAAAGTGCAAATGCAAACAAACGAAGCTCTAACCTACGCACAGGAGCAAGAAAATGACACATGAAATGACATGACTGCTCGAAGAACAATAATAATAGTCCACAGAAGATCCTAGACTGGTGGCAAAAGACTACCAAAAAAGGTACACTATCACATGATGTGTCATGACTATAACTGGTAATCAGACTAGAAAATCAACGGTTTGTTAATCAGACAGATGTGGTCAATCTACGCCCATTCGGAAGATGTGGCCTAGGCACCACCCAGGTCAG

>CDS

ATGGACATGGCCGGCTCCGATCAGCAGCGGTGCTCCCCTTCCTCGCCGTCGTTGTCCTCTCATCTGAAGCGCCCCGCCGGGCGCACCAAGTTCAAGGAGACGCGTCACCCGGTGTACCGCGGCGTGCGGCGCCGCGGCAGCGCCGGCCGGTGGGTGTGCGAGGTGCGCGTCCCCGGCAAGCGAGGCGAGCGGCTCTGGCTCGGGACGCACCTTACCGCCGAGGCGGCCGCACGTGCGCACGACGCCGCCATGCTCTGCCTGCTCGACCGCCCCGCCCCGTGTCTCAACTTCGCCGATTCCGTCTGGCTCCTCGCCGTGCCGTCCGCGCTCTCCGATCTCGCTGACGTCCGGCGCGCGGCTCTCAGCGCCGTCGCGGATTTCCAGCGCCGGGAGGCCGCAAGCGGCGCCGCCACGAGGGCCCAGGCGGCGGCCGCCCTCATTGACGAGGGAACCTGTAGCCAATCCGCGCAGTCGTCCATGGAAAATACCGGCTCGTCTTCGACGTCGTCATCCCTACCTTCAGCCGACGGAATGCTTGAGGTGCCGGCCACACTGGGCAGCAACATGTTCGAGCTGGACATGTCCGGGGAAATGGACCTGGACACATACTATGCGTACTTCGCGGAGGGGCTTCTCCTGGAGCCGCCGCAACCGCCGGCCGCCGGCGCGTGCTGGGATATCGATGGCGGTGGAGCAGACGCCGCGCTCTGGAGCTACTAA

>DNA

TTGTGTTACTCAATACCTAGAGGTTATATGAAGAACAATATCATAAATCACATTGCTCGAAAACTACTTATCCTCACGGATTATAGAAAAGTGAGGAAATAATTTGCAAACAAAATCATGTGAAAATCCAATAGATTATACACAATCTTATGTTCTGCCAATGAAATTGGTATGAGACATCCTCCATATTTGCAAAGTTCAGGGGGAGTGATCTCCCAAACTATAACCTATTAACAATCATCAAATTGCATATATTGTACTCTTTTTCCCTTGATGAGTTTTTCCCTAATGGTTTCTCATAAAAGGTTTNTAACGAGACAATATAAACACAAGTGTTTGTATATGCCATATCATTTTCTCCTTATATTTTTTCCCACTGGGTTTTAAAGGAGTTTTCAATGGCATGTCAGATGCACTCTTTTCCCTTATGAGTTTTCTCCTGTAGTTTCTCATAATGGTTTTTAATGAGGTAATACCTTCTCAAAGATCGTATGTCATACTTTCTATTTTCCCTATTGGGATTTTTAAAGGAAGTACTCAAGACATATCAATTGTTCTCTAAACTCACCAATGAATTTTCTCCTTCTCCAAAGGTTTTTCTCATATGAGTTATCAAGAGAAAATAATCATTATATGTTGCATCATTTTCTCCTTATTATTTTTCCACTGGGTTTAAAGAAGATTTAGCAACATATCTGCATAATTCTCATATTTTTCCCACACGGTTTTTAGAGGAGACTTCAAGATTATTCAAAGAATTTATCAAGATTATGAATATATCCAAGAAGACGCGTTGTACAAGGGGGAGTGTTAAAAATAGAATAGTTGCTTATTAATTAAGCTAATTAGGATTAAATTAATTGTTAGATAAGTTACTTGGTTCTCAAGTAACCATGTAATTCCTTGACTCTCAAGGCAACTATGTAATTCCTTGACTGTCAAGTAACTATGTGTTTACTTGGCTGCCAAGCAACATGTTTACTTCCGTTTGAATATAAATACCCTACTTCTATCATCAATAAAGACTAATGGATCATCTTTCATTCATCTCTCTTTCTTCTTCTTTTTTACATTTAACATGCATAACGCGGGGTAAAACTGTGCCACGAGTCCAAGGCTGGTCCATGCTTGTGGCCCCAATTATCAGTTGCCAGCAAGACCTTTTTAATGGTGCAAATCCATGACGCGTCACTTGTCCCCTGCCGCTTGCCCACGACGCGTACAGAACGAGCCTCAACAAAAAAGATGTCTTCTTCGTTTTGACGCTGCTATTCCCTGACGCGTCACTTGCCCCAAGCCGCTTCCCCACACCGCGTAAATGGCACACTCCCCAAGACACTCAATCGCTGGAGAACAAGATATACTAACGCGGGAGGAAAATGCGTCACTTGTCCCAGCCTGCTATATGCCATCATTTCAACGCTGCAAATTCCTGACGCGTCTTCTCTCGCTGCCGCTAGTCTGGAACGCGTCAGGCAGAACTATAAAAGACACTCAATCTAACAAACACTCCCCAGCTCAATCTCAGCCTGCTCTCACACTCCCAGACCTCGGTCCACCGTACGTAAGCTGGAACCAGCACCGACCACCTGCAATAGCAATATGGACATGGCCGGCTCCGATCAGCAGCGGTGCTCCCCTTCCTCGCCGTCGTTGTCCTCTCATCTGAAGCGCCCCGCCGGGCGCACCAAGTTCAAGGAGACGCGTCACCCGGTGTACCGCGGCGTGCGGCGCCGCGGCAGCGCCGGCCGGTGGGTGTGCGAGGTGCGCGTCCCCGGCAAGCGAGGCGAGCGGCTCTGGCTCGGGACGCACCTTACCGCCGAGGCGGCCGCACGTGCGCACGACGCCGCCATGCTCTGCCTGCTCGACCGCCCCGCCCCGTGTCTCAACTTCGCCGATTCCGTCTGGCTCCTCGCCGTGCCGTCCGCGCTCTCCGATCTCGCTGACGTCCGGCGCGCGGCTCTCAGCGCCGTCGCGGATTTCCAGCGCCGGGAGGCCGCAAGCGGCGCCGCCACGAGGGCCCAGGCGGCGGCCGCCCTCATTGACGAGGGAACCTGTAGCCAATCCGCGCAGTCGTCCATGGAAAATACCGGCTCGTCTTCGACGTCGTCATCCCTACCTTCAGCCGACGGAATGCTTGAGGTGCCGGCCACACTGGGCAGCAACATGTTCGAGCTGGACATGTCCGGGGAAATGGACCTGGACACATACTATGCGTACTTCGCGGAGGGGCTTCTCCTGGAGCCGCCGCAACCGCCGGCCGCCGGCGCGTGCTGGGATATCGATGGCGGTGGAGCAGACGCCGCGCTCTGGAGCTACTAAAATACTCCGGCCAGCTGCTTTGACTCTATGCATGGAACAAACCGAAGCTGTTCCCTTCCAGCATGATAAAATTGTGGAACTAAAATCCATCAAATACTGCGACACAGCTGCAAATGCAAACAAACGAAGTTCTAACCTAGGCACAGGAGCAAGAAAATGACACATGAAATGACATGACTGCTCGAAGAATAATAATAGTCCACAGAAGATCCTAGACTGGTGGCAAATGACTGCCAAAAAAGGTACACTATCACATGATGTGTCATGACTATAACTGGTAGTCAGACTAGAAAAATCAACGGCTTGTTAATCAGACAGATGTGGTCAGGGCGCCCATTCGGAAGATGTGGCCTAGGCACCACCCAGGTCAG

>HvDREB1.7

>Protein

MSPTLSLKLKKSSHTPQSSVSSSTMLRLFKKEAACQSPSTLPVAMDMGLEVSSSSPSSSSVSSSPEHAARRASPAKRPAGRTKFRETRHPVYRGVRRRGNTERWVCEVRVPGKRGARLWLGTYATAEVAARANDAAMLALGGRSATCLNFADSAWLLAVPSALSDLADVRRAAVEAVADFQRREAADGSLAIAVPKEASSGAPSLSPSSGSDSAGSTGTSEPSANGVFEGPVVMDSEMFRLDLFPEMDLGSYYMSLAEALLMDPPPTATIIHAYEDNGDGGADVRLWSYSVDM

>cDNA

AGCAGGAGAAAAGTCTCATGAACACCACTTGATTTCATCCCATTGTCACCAGCTGTCCGGACACCGCATCCCTACCGCCGTCCCAAGCGCGTTCATACACTTCAACCTCCAGCACCACGCATACCTATAAATATGTCTCCCACACTCTCGCTCAAGCTCAAGAAATCATCTCACACTCCTCAGTCCTCAGTAAGCTCAAGCACCATGCTCAGACTGTTCAAGAAGGAAGCCGCCTGCCAATCACCCAGCACTCTGCCGGTAGCCATGGACATGGGCCTTGAGGTCTCGAGCTCCTCCCCCTCCTCCTCGTCGGTGTCGTCCTCGCCCGAGCACGCGGCGAGGCGGGCGTCGCCGGCGAAGCGCCCCGCTGGGCGCACCAAGTTCCGGGAGACGCGGCACCCGGTGTACCGCGGCGTGCGGCGCCGGGGCAACACCGAACGGTGGGTCTGCGAGGTGCGCGTCCCCGGCAAGCGCGGTGCTCGGCTCTGGCTCGGGACGTACGCCACGGCTGAGGTCGCCGCGCGCGCGAACGACGCTGCCATGCTCGCCCTGGGCGGCCGCTCCGCCACGTGCCTCAACTTCGCCGATTCCGCGTGGCTGCTCGCCGTGCCGTCCGCCCTGTCCGATCTCGCAGACGTCCGGCGCGCGGCTGTCGAGGCCGTCGCGGATTTCCAGCGACGGGAGGCTGCCGATGGCTCCCTCGCCATCGCTGTCCCTAAGGAGGCCTCCTCTGGCGCTCCTTCACTATCTCCGTCGTCTGGGTCCGACAGTGCCGGTTCGACGGGGACGTCGGAACCTTCCGCCAATGGAGTGTTCGAGGGGCCCGTTGTAATGGACAGTGAAATGTTCAGGCTTGACTTGTTCCCGGAAATGGACCTGGGCTCGTACTACATGAGCCTCGCGGAGGCGCTGCTCATGGACCCGCCGCCTACAGCGACCATCATCCACGCGTACGAAGACAACGGCGACGGGGGAGCTGATGTCCGGCTCTGGAGCTATAGTGTCGATATGTGATTTCCCAGATGATTCTGCTCTGTTTTGACTGTGTACTGACTGCTGAGTAGTTTTTTTGTTTCCTTAGGAAAGTTTTCCTCTTTTAGAGTGAAGATGTTGTAGCTAATAAACTGAAGCTGCTTCCAATCCAGCACTGAATGAAACAGTTTTAGTCGCTGCAATTCTTATGCCACCTTTATGACTCCGGCTCTTTTATTCCTGAAACATTTCG

>CDS

ATGTCTCCCACACTCTCGCTCAAGCTCAAGAAATCATCTCACACTCCTCAGTCCTCAGTAAGCTCAAGCACCATGCTCAGACTGTTCAAGAAGGAAGCCGCCTGCCAATCACCCAGCACTCTGCCGGTAGCCATGGACATGGGCCTTGAGGTCTCGAGCTCCTCCCCCTCCTCCTCGTCGGTGTCGTCCTCGCCCGAGCACGCGGCGAGGCGGGCGTCGCCGGCGAAGCGCCCCGCTGGGCGCACCAAGTTCCGGGAGACGCGGCACCCGGTGTACCGCGGCGTGCGGCGCCGGGGCAACACCGAACGGTGGGTCTGCGAGGTGCGCGTCCCCGGCAAGCGCGGTGCTCGGCTCTGGCTCGGGACGTACGCCACGGCTGAGGTCGCCGCGCGCGCGAACGACGCTGCCATGCTCGCCCTGGGCGGCCGCTCCGCCACGTGCCTCAACTTCGCCGATTCCGCGTGGCTGCTCGCCGTGCCGTCCGCCCTGTCCGATCTCGCAGACGTCCGGCGCGCGGCTGTCGAGGCCGTCGCGGATTTCCAGCGACGGGAGGCTGCCGATGGCTCCCTCGCCATCGCTGTCCCTAAGGAGGCCTCCTCTGGCGCTCCTTCACTATCTCCGTCGTCTGGGTCCGACAGTGCCGGTTCGACGGGGACGTCGGAACCTTCCGCCAATGGAGTGTTCGAGGGGCCCGTTGTAATGGACAGTGAAATGTTCAGGCTTGACTTGTTCCCGGAAATGGACCTGGGCTCGTACTACATGAGCCTCGCGGAGGCGCTGCTCATGGACCCGCCGCCTACAGCGACCATCATCCACGCGTACGAAGACAACGGCGACGGGGGAGCTGATGTCCGGCTCTGGAGCTATAGTGTCGATATGTGA

>DNA

AATCCCTAAGCCATGCGACAAGGCTTCTGAACCGATGGGTAGAAAGGAATCGAGGGGGGTTAGAGGGGATCTGCCCATACAAACCCTGCCTATCAAATGGGCCGTCGAGGCTTCGAGAGGTTTGCAGCCCCCGCGAGGATAATCCTCAGCAACCCCCCTCCAAACCCCTAGCGCCAAACGAAGCCTTAGGTGATACCATGATACCATTTCAAAAAATTCAATTTCAAACGTTTCAAAAAATTCTAAAAATATTATGGATTTTCACATCACATGAATGCACAATCTTTAAAAATTTCAGGTCCAAATTTGAAATATACATTGAGAAACATAAAAAGACAAATTCAGCATAAATAGTGTAAAAAAGGCAAAAGTCAAAACGCCACAATTCACATCTAAATTTGTCTTTTCTTGTTTTTCAATGTGTATTTAGAATTTGCATGTGAAATTTTTGGGGGATGTGCATTCATGCGATGTGGACATCCGCAATTATTTTCAGAACTTTTTGAGATGTTTAAAATTAATATTTTTGAATGATATCATGGAAGCATTTGGAAGATGGTATCACCGGATACTCTCCCTCTCGGGGAACAAGACGACCTCATTCAGCAGTGACCGCTGTCTTCTCTTTCTGGCCGATCAGCCGGCGGACCAATCAGGCAAGGCAATCACCGCTGCATTAACACTGTTAAGCCAGAAGAAAGTTCGCTTTTTTTTCTTTTGAGAGGAGCAGGAAGTTGCCTTTTTTGCTTAACACTGCAATGCCAAAAGCCCCCACACGCCCAGCAGGAGAAAAGTCTCATGAACACCACTTGATTTCATCCCATTGTCACCAGCTGTCCGGACACCGCATCCCTACCGCCGTCCCAAGCGCGTTCATACACTTCAACCTCCAGCACCACGCATACCTATAAATATGTCTCCCACACTCTCGCTCAAGCTCAAGAAATCATCTCACACTCCTCAGTCCTCAGTAAGCTCAAGCACCATGCTCAGACTGTTCAAGAAGGAAGCCGCCTGCCAATCACCCAGCACTCTGCCGGTAGCCATGGACATGGGCCTTGAGGTCTCGAGCTCCTCCCCCTCCTCCTCGTCGGTGTCGTCCTCGCCCGAGCACGCGGCGAGGCGGGCGTCGCCGGCGAAGCGCCCCGCTGGGCGCACCAAGTTCCGGGAGACGCGGCACCCGGTGTACCGCGGCGTGCGGCGCCGGGGCAACACCGAACGGTGGGTCTGCGAGGTGCGCGTCCCCGGCAAGCGCGGTGCTCGGCTCTGGCTCGGGACGTACGCCACGGCTGAGGTCGCCGCGCGCGCGAACGACGCTGCCATGCTCGCCCTGGGCGGCCGCTCCGCCACGTGCCTCAACTTCGCCGATTCCGCGTGGCTGCTCGCCGTGCCGTCCGCCCTGTCCGATCTCGCAGACGTCCGGCGCGCGGCTGTCGAGGCCGTCGCGGATTTCCAGCGACGGGAGGCTGCCGATGGCTCCCTCGCCATCGCTGTCCCTAAGGAGGCCTCCTCTGGCGCTCCTTCACTATCTCCGTCGTCTGGGTCCGACAGTGCCGGTTCGACGGGGACGTCGGAACCTTCCGCCAATGGAGTGTTCGAGGGGCCCGTTGTAATGGACAGTGAAATGTTCAGGCTTGACTTGTTCCCGGAAATGGACCTGGGCTCGTACTACATGAGCCTCGCGGAGGCGCTGCTCATGGACCCGCCGCCTACAGCGACCATCATCCACGCGTACGAAGACAACGGCGACGGGGGAGCTGATGTCCGGCTCTGGAGCTATAGTGTCGATATGTGATTTCCCAGATGATTCTGCTCTGTTTTGACTGTGTACTGACTGCTGAGTAGTTTTTTTGTTTCCTTAGGAAAGTTTTCCTCTTTTAGAGTGAAGATGTTGTAGCTAATAAACTGAAGCTGCTTCCAATCCAGCACTGAATGAAACAGTTTTAGTCGCTGCAATTCTTATGCCACCTTTATGACTCCGGCTCTTTTATTCCTGAAACATTTCG

>HvDREB1.8

>Protein

MCQIKKEMSGESGSPCSGENYYYSPSTSPEHQQAKQQAAWTSAPAKRPAGRTKFRETRHPVYRGVRRRGNAGRWVCEVRVPGRRGSRLWLGTFDTAEAAARANDAAMLALAAGGAGCLNFADSAELLAVPAASSYRSLDEVRHAVVEAVEDLLRREAHAEDDALSVSCTSSSAPSSLTDDESSSSPAAEGSPFELDVLSDMGWDLYYASLAQGMLMAPPASLAAALGDYGEAHLADVPLWSYQS

>cDNA

ACTTCACACTCGACTCGAGCAACCATAACAAGGACCTCAAGCCGGCCAAAGAAGAAAGAAGAGAGCCATCCAGAGCAGAGCAAGACTACTCTCTCTTGTTGGACTTTGATCGGAGCAAAGAAGATGTGTCAGATCAAGAAGGAGATGAGCGGCGAGTCGGGCTCTCCCTGCAGCGGGGAGAACTACTACTACTCGCCCTCGACGTCGCCGGAGCATCAGCAGGCGAAGCAGCAGGCGGCGTGGACGTCGGCGCCGGCGAAGCGGCCGGCGGGGCGGACCAAGTTCAGGGAGACGCGCCACCCGGTGTACCGCGGCGTGCGGCGCAGGGGCAATGCCGGGCGGTGGGTGTGCGAGGTGCGCGTGCCCGGCAGGCGCGGGAGCAGGCTCTGGCTCGGTACCTTCGACACCGCCGAGGCCGCCGCGCGCGCGAACGACGCCGCCATGCTCGCGCTCGCCGCCGGGGGCGCGGGCTGCCTCAACTTCGCCGACTCCGCCGAGCTACTCGCCGTGCCGGCTGCCTCCTCGTACCGCAGCCTCGACGAGGTCCGCCACGCCGTCGTGGAGGCCGTCGAGGACTTGCTGCGGCGCGAGGCGCACGCAGAGGACGACGCGCTCTCGGTCTCTTGCACCTCCTCGTCCGCGCCCTCCTCCCTCACCGACGACGAGTCGTCCTCTTCCCCGGCGGCCGAGGGCTCGCCGTTCGAGCTGGACGTCCTGAGCGACATGGGATGGGACCTTTACTACGCGAGCCTGGCGCAGGGGATGCTCATGGCGCCGCCTGCTTCCTTGGCCGCGGCGCTCGGCGATTACGGCGAGGCTCACCTCGCCGACGTGCCACTCTGGAGCTACCAGAGCTAG

>CDS

ATGTGTCAGATCAAGAAGGAGATGAGCGGCGAGTCGGGCTCTCCCTGCAGCGGGGAGAACTACTACTACTCGCCCTCGACGTCGCCGGAGCATCAGCAGGCGAAGCAGCAGGCGGCGTGGACGTCGGCGCCGGCGAAGCGGCCGGCGGGGCGGACCAAGTTCAGGGAGACGCGCCACCCGGTGTACCGCGGCGTGCGGCGCAGGGGCAATGCCGGGCGGTGGGTGTGCGAGGTGCGCGTGCCCGGCAGGCGCGGGAGCAGGCTCTGGCTCGGTACCTTCGACACCGCCGAGGCCGCCGCGCGCGCGAACGACGCCGCCATGCTCGCGCTCGCCGCCGGGGGCGCGGGCTGCCTCAACTTCGCCGACTCCGCCGAGCTACTCGCCGTGCCGGCTGCCTCCTCGTACCGCAGCCTCGACGAGGTCCGCCACGCCGTCGTGGAGGCCGTCGAGGACTTGCTGCGGCGCGAGGCGCACGCAGAGGACGACGCGCTCTCGGTCTCTTGCACCTCCTCGTCCGCGCCCTCCTCCCTCACCGACGACGAGTCGTCCTCTTCCCCGGCGGCCGAGGGCTCGCCGTTCGAGCTGGACGTCCTGAGCGACATGGGATGGGACCTTTACTACGCGAGCCTGGCGCAGGGGATGCTCATGGCGCCGCCTGCTTCCTTGGCCGCGGCGCTCGGCGATTACGGCGAGGCTCACCTCGCCGACGTGCCACTCTGGAGCTACCAGAGCTAG

>DNA

AAGTTGAAGCGGACCTAGTCTAACCCACTTGAACTCGACTGGTTTGTAGCCGTATTTCAGGCTGCTGGTCTTTAGGAGCACGTGCACAAAGATTTTCCGTTTGTCATCGATAATATCAAGCCCTCTCAGGTAGGGAGAGATGACAACGCCACCTAAATGCCTCATTTGGACGATGGTGGTGATCGTTTAGTGCCAATGAACCTTGATATAATTTTCATTAATGAATATAAAATCTTTGCGCGACCATTTTCCACCCTCATTTATAAGAATGTGAAATCAATGCATTGAATGTGAATCCAACGTATTCATGTAATTTTTGGATTACTGATGAAGAATATGTGGCCCAATATCAGGTGGATCCTATGTGTTCCGAAGGTCACAGACATGACAACTTTACTATGGTGTTGGTCTTTTGTAGGCTAGTATTAGAACATTTTTAGTAGAATCCGTATAATGTCCCGACACATAGAACAATTGTCAAAATACAGGTCAGCGTGAAAAAACCGCCCAACCAGACCCCGTATCGGACTTTGACCTGTAATTTTTTTGTGGGCGCGGCAAAAGTTTATCCTCAACCTTTAGATTCATGGATGGAAGGCCGATCGAGTTTGCCCCTTATCCGCAGCGAGATTTGACATGAGAGAAATTTTCGCACGGCCGTTCCCGCTTTGCCGCCATTGCCCCGCCTCCATCCGTTTCTGTTCATCTTCCCAGGTCATTCTTCGTCGTCATGGAAGCCTCACAGCCGTCCCACATCACCGTGGTTGTGGCGCACGTGTAATTCTCTCCGACAGATCTCGTCTTCGGCCATGTCGCCCTCGTCGTCGCTCAAGAAACCACCCTACCTGTCCGCAAGCGACAGGGTAGCGTCATCGTGCCAGACGGAAATCTTCGTCATGTTCATGGTAACCGTCTGGATGCCCGCAATGCCCCCTTGATTTGCATTCGTTTTACGAAAGAACGGTTCGTGGATAGATGGTACCGCGTTGGATAATAAAATGAGCTTGAACAATCCAATCAAGATGGAGCGGACGGTTTGAGGGTCTAGTTAGAGAAACACACACGCACAAAAAAAAACTAGCCCTTTCTCACAGAAAAAGAAAAAAAATACTGCAGGCGAAGTGAAAAAAGAGTCTAAGGTGGAGTACAAAACAGACAGGGATTCCAGGTTTGCATCTGCTCATCCACGTCAGAGATGACAATTGAAGCCGGTCACCAAAACCCCCACCCCACGTGCTTCACCTAGGAGCAAGAACTCCACTTCCACTCTTGGTCTGCAACTGCAACGAACCAACACGAATTCCGATTTTTGGTCTGCAGCCCCAATCGCGAGGAAACAATCAGCTTCGACGTTAAGAATCAGACGTCAAACAACACTGACCATCTAGACCCCATCAAAAGAAGAATCGTACCACACAAGCACCTGATACCCTCGGCAAAACCAAAACCCGAATTCGCAAACTGCGGCTCTAGAAAACCATTAACAATGACGAGGCAAAGTAAAACGGCCGCCGGACGGGGCGCAAGGCAAAAGGGCGAAAGACAAAGAGAGCAGGAGCTAGGCGGAAAACGATAAGGGCAAAGCGAAAGCATGAATGTACTAATATATAATAAAGATAATAAAGAAAAGAAATCTTGGGGTCACTCTCGAAGTCTCTCACTTCTCCCCCCCACTTGCGCGCGCCTTCGTACGGTGCATCCCCCCTTCGCCGTATCCAGCCTGTGTCCTCTCCTCTCAGCCGCTGGCTCGCTCGCCGCGCTTTGCCCCCTGCCTTCAGCCGCGAGCCACCGCCACCGCCACCGCCGCCACCACCACCTATAAGTACGCGCTCCCCAAACCACCCTGCTCAAATTTCTCATCGCACAGCAAAACCACCACTTCACACTCGACTCGAGCAACCATAACAAGGACCTCAAGCCGGCCAAAGAAGAAAGAAGAGAGCCATCCAGAGCAGAGCAAGACTACTCTCTCTTGTTGGACTTTGATCGGAGCAAAGAAGATGTGTCAGATCAAGAAGGAGATGAGCGGCGAGTCGGGCTCTCCCTGCAGCGGGGAGAACTACTACTACTCGCCCTCGACGTCGCCGGAGCATCAGCAGGCGAAGCAGCAGGCGGCGTGGACGTCGGCGCCGGCGAAGCGGCCGGCGGGGCGGACCAAGTTCAGGGAGACGCGCCACCCGGTGTACCGCGGCGTGCGGCGCAGGGGCAATGCCGGGCGGTGGGTGTGCGAGGTGCGCGTGCCCGGCAGGCGCGGGAGCAGGCTCTGGCTCGGTACCTTCGACACCGCCGAGGCCGCCGCGCGCGCGAACGACGCCGCCATGCTCGCGCTCGCCGCCGGGGGCGCGGGCTGCCTCAACTTCGCCGACTCCGCCGAGCTACTCGCCGTGCCGGCTGCCTCCTCGTACCGCAGCCTCGACGAGGTCCGCCACGCCGTCGTGGAGGCCGTCGAGGACTTGCTGCGGCGCGAGGCGCACGCAGAGGACGACGCGCTCTCGGTCTCTTGCACCTCCTCGTCCGCGCCCTCCTCCCTCACCGACGACGAGTCGTCCTCTTCCCCGGCGGCCGAGGGCTCGCCGTTCGAGCTGGACGTCCTGAGCGACATGGGATGGGACCTTTACTACGCGAGCCTGGCGCAGGGGATGCTCATGGCGCCGCCTGCTTCCTTGGCCGCGGCGCTCGGCGATTACGGCGAGGCTCACCTCGCCGACGTGCCACTCTGGAGCTACCAGAGCTAG

>HvDREB1.9

>Protein

MDTVAAWPQFEEQDYMTVWPEEQEYRTVWSEPPKRRAGRIKLQETRHPVYRGVRRRGKVGQWVCELRVPVSRGYSRLWLGTFANPEMAARAHDSAALALSGHDACLNFADSAWRMMPVHATGSFRLAPAQEIKDAVAVALEVFQGQHPADACTAEESTTPITSSDLSGLDDEHWIGGMDAGSYYASLAQGMLMEPPAAGGWREDDGEHDDGFNTSTSLWSY

>cDNA

CCATGGACACAGTTGCCGCCTGGCCGCAGTTTGAGGAGCAAGACTACATGACGGTGTGGCCGGAGGAGCAGGAGTACCGGACGGTTTGGTCGGAGCCGCCGAAGCGGCGGGCCGGCCGGATCAAGTTGCAGGAGACGCGCCACCCGGTGTACCGCGGCGTGCGACGCCGTGGCAAGGTCGGGCAGTGGGTGTGCGAGCTGCGCGTCCCCGTAAGCCGGGGTTACTCCAGGCTCTGGCTCGGCACCTTCGCCAACCCCGAGATGGCGGCGCGCGCGCACGACTCCGCCGCGCTCGCCCTCTCCGGCCATGATGCGTGCCTCAACTTCGCCGACTCCGCCTGGCGGATGATGCCCGTCCACGCGACTGGGTCATTTAGGCTCGCCCCCGCGCAAGAGATCAAGGACGCCGTCGCCGTCGCCCTCGAGGTGTTCCAGGGGCAGCACCCAGCCGACGCGTGCACGGCCGAGGAGAGCACGACCCCCATCACCTCAAGCGACCTATCGGGGCTGGACGACGAGCACTGGATCGGCGGCATGGACGCCGGGTCCTACTACGCGAGCTTGGCGCAGGGGATGCTCATGGAGCCGCCGGCCGCCGGAGGGTGGCGGGAGGACGACGGCGAACACGACGACGGCTTCAACACGTCCACGTCGCTGTGGAGCTACTAGTTCGACTGATCAAGCAGTGTAAATTATTAGAGTTGTAGTATCAGTAGCTAGTACTAGTAGCTGTGTTCTTCCACCAGGCGTCAGGCCTGGCAAGCAATCTCAAACCATGAATATGCAAAAAAAGCTGTGTTCTTCCAGATATGGAAAGAAGAGAGAGTAAGCGTCGGGGGCTATGTTTTGCCCAAATGCGAAAGCTCCCGGTTTCTACTTCC

>CDS

ATGACGGTGTGGCCGGAGGAGCAGGAGTACCGGACGGTTTGGTCGGAGCCGCCGAAGCGGCGGGCCGGCCGGATCAAGTTGCAGGAGACGCGCCACCCGGTGTACCGCGGCGTGCGACGCCGTGGCAAGGTCGGGCAGTGGGTGTGCGAGCTGCGCGTCCCCGTAAGCCGGGGTTACTCCAGGCTCTGGCTCGGCACCTTCGCCAACCCCGAGATGGCGGCGCGCGCGCACGACTCCGCCGCGCTCGCCCTCTCCGGCCATGATGCGTGCCTCAACTTCGCCGACTCCGCCTGGCGGATGATGCCCGTCCACGCGACTGGGTCATTTAGGCTCGCCCCCGCGCAAGAGATCAAGGACGCCGTCGCCGTCGCCCTCGAGGTGTTCCAGGGGCAGCACCCAGCCGACGCGTGCACGGCCGAGGAGAGCACGACCCCCATCACCTCAAGCGACCTATCGGGGCTGGACGACGAGCACTGGATCGGCGGCATGGACGCCGGGTCCTACTACGCGAGCTTGGCGCAGGGGATGCTCATGGAGCCGCCGGCCGCCGGAGGGTGGCGGGAGGACGACGGCGAACACGACGACGGCTTCAACACGTCCACGTCGCTGTGGAGCTACTAG

>DNA

AGTTATACGAATAGGCCCTGTCCCCACTAGTGCAAATCTAGCGCGCGGACGTCCGCTTTCTCCCTCGCGCGTGCTAATTTCCCGCCTCCGCTGTAGCGCGATTTTCCATCCCGTGCGTGCTAAGAACGCAGTTTACCGCGCGCATGTGTTTTTTGGCGTCACTTTTGGAGATGCTTTAAGTATTGCATATCTAAGTCCTACTCTATTTGGTCCTAAATATAAGTATTTCTAAAGATTTTATAAAAAAAAACTATGTACAAAGCAAAATGAGTGAATTTACATTTTAAAATATATTTATATACATTTATAATTTTTAGTGGAACCTTTTAAAAAATTTATATTTAAAAACGAAAGGAGTATATAATTAATCTTACGTAAAGATTCGTGTAGGTATTCTTTTTATGTTTAAAATATGCAATAACTATGTCACATATTGTATCCTCCTTCGTCTCGTTCTCATCCATTCCGAGCTCCAGCTCTCCAACTCACTAGCTCTTAACATACTACTGCCTCGCTACACGCCTACACAGCTGCACCAACGCCACAACGCCCTCTCGACGCTAGCTGCGAGCCATGGACACAGTTGCCGCCTGGCCGCAGTTTGAGGAGCAAGACTACATGACGGTGTGGCCGGAGGAGCAGGAGTACCGGACGGTTTGGTCGGAGCCGCCGAAGCGGCGGGCCGGCCGGATCAAGTTGCAGGAGACGCGCCACCCGGTGTACCGCGGCGTGCGACGCCGTGGCAAGGTCGGGCAGTGGGTGTGCGAGCTGCGCGTCCCCGTAAGCCGGGGTTACTCCAGGCTCTGGCTCGGCACCTTCGCCAACCCCGAGATGGCGGCGCGCGCGCACGACTCCGCCGCGCTCGCCCTCTCCGGCCATGATGCGTGCCTCAACTTCGCCGACTCCGCCTGGCGGATGATGCCCGTCCACGCGACTGGGTCGTTCAGGCTCGCCCCCGCGCAAGAGATCAAGGACGCCGTCGCCGTCGCCCTCGAGGTGTTCCAGGGGCAGCACCCAGCCGACGCGTGCACGGCCGAGGAGAGCACGACCCCCATCACCTCAAGCGACCTATCGGGGCTGGACGACGAGCACTGGATCGGCGGCATGGACGCCGGGTCCTACTACGCGAGCTTGGCGCAGGGGATGCTCATGGAGCCGCCGGCCGCCGGAGGGGGGCGGGAGGACGACGGCGAACACGACGACGGCTTCAACACGTCCACGTCGCTGTGGAGCTACTAG

>HvDREB1.10

>Protein

MDAADAASPCDGHRTVWSEPPKRPAGRTKFKETRHPLYRGVRRRGPAGRWVCEVRVLGMRGSRLWLGTFTTAEMAARAHDAAVLALSGRAACLNFADSAWRMLPLLAGPFSTAKEIKDAVAVAVLAFQRQHPVASTAPMSPARTAVDEKEVDGSPAPSALFMSSELLNEHWFGGMDAGSCYSEGMFIESPDTRPWREDLELGGVQTPPWSYLFD

>cDNA

CGGGTATATATGCTCCGCGGCCTGCTCATCCAACCAAACTCCACCGCTCCACTCTCCAGCATCCATCTCTCCCAAGTCTCAGCGCAGCAGCTAATAAAACACGCTGCTTACTCCACAGTCGACCGGCTCCCGGCGACACTGCGATCGATCGATGGACGCCGCAGACGCCGCCTCCCCGTGTGATGGGCACAGGACGGTGTGGTCGGAGCCGCCGAAGCGGCCTGCCGGCCGGACCAAGTTCAAGGAGACGCGCCACCCGCTGTACCGCGGCGTGCGGCGCCGGGGCCCCGCAGGCCGGTGGGTGTGCGAGGTGCGCGTGCTCGGGATGAGGGGCTCCAGGCTCTGGCTCGGCACCTTCACCACCGCCGAGATGGCAGCGCGCGCGCACGACGCCGCCGTTCTCGCGCTCTCTGGCCGCGCCGCCTGTCTGAACTTCGCCGACTCTGCTTGGCGGATGCTCCCCCTCCTCGCCGGCCCGTTCAGCACCGCTAAGGAGATCAAGGATGCCGTCGCCGTCGCCGTCCTGGCGTTCCAAAGACAGCACCCGGTCGCGTCCACGGCGCCAATGTCCCCGGCACGGACAGCCGTTGACGAGAAGGAAGTCGATGGCTCGCCGGCGCCGAGCGCTCTGTTCATGTCCAGCGAGCTGTTGAATGAGCACTGGTTTGGCGGCATGGATGCCGGATCATGCTACTCGGAGGGCATGTTCATAGAGTCGCCGGATACCAGACCGTGGCGGGAAGACCTCGAGCTCGGTGGCGTCCAGACACCGCCATGGAGCTACTTGTTCGACTAAGCAGTTAAAGTATTTGGATGGGTTTTCTGCTTCTGTTCCACCAAATATGGGAGGAAACAGAGGGGAAATTTTTTCCCATATGTCCATACCTGCTCGTTCATTGTGAAAGCCTCTGCTGTCTATTTTCTGGGAGGCTGCATTTGTACTTGATTTTTTTCCTTTCTAGGACATCACTTTGAGTGCTTTTATGTTGTTGTTGTTGAGTTGAATCACTTGAGTGTTCTAGGGAATACAGAATGAGTACTTCTCAGCTATTATGGACCTCAAGCACTCCATGGTCGACATAAGATATTACTTATGAGAAAGAGAACAAGAAAATACACATTAAAGAACTTTCGGCGTCACAGCTTGACAATGATGGA

>CDS

ATGGACGCCGCAGACGCCGCCTCCCCGTGTGATGGGCACAGGACGGTGTGGTCGGAGCCGCCGAAGCGGCCTGCCGGCCGGACCAAGTTCAAGGAGACGCGCCACCCGCTGTACCGCGGCGTGCGGCGCCGGGGCCCCGCAGGCCGGTGGGTGTGCGAGGTGCGCGTGCTCGGGATGAGGGGCTCCAGGCTCTGGCTCGGCACCTTCACCACCGCCGAGATGGCAGCGCGCGCGCACGACGCCGCCGTTCTCGCGCTCTCTGGCCGCGCCGCCTGTCTGAACTTCGCCGACTCTGCTTGGCGGATGCTCCCCCTCCTCGCCGGCCCGTTCAGCACCGCTAAGGAGATCAAGGATGCCGTCGCCGTCGCCGTCCTGGCGTTCCAAAGACAGCACCCGGTCGCGTCCACGGCGCCAATGTCCCCGGCACGGACAGCCGTTGACGAGAAGGAAGTCGATGGCTCGCCGGCGCCGAGCGCTCTGTTCATGTCCAGCGAGCTGTTGAATGAGCACTGGTTTGGCGGCATGGATGCCGGATCATGCTACTCGGAGGGCATGTTCATAGAGTCGCCGGATACCAGACCGTGGCGGGAAGACCTCGAGCTCGGTGGCGTCCAGACACCGCCATGGAGCTACTTGTTCGACTAA

>DNA

TTCCCTTCTCTCTCCTCTTCTCTTTCATCCAACTCAGCAAAAATACAGTATTTTAATCCTTACAGCCTGCTGACTGCCTGCTAACTGTACCTTGAAGCTACCGTTGCCCTGGATCTTTTGCCGATTTCTTTGTCACAAAATTCTATGCCGCTATAAGCGAGTCCAGCTGTCCTTTTCATTCGCTCACTCACTGAACAGTGGGTGAGTGTGAGTGTGAGGAAGCAGGCGCTGCGCTCTCATGCCACGCGCCTGGTGGCACGTTACGTCGCGCTCTGGGCCTCCTTCGTTGATGCTGTGGCCAGCACAGCAGGGCTCCGAAAGATATGCCAAGATGCTTATTTTATTCATTAATTTCCCACAAAAGATGCTTGGATGTGAAGTGTGGCCTCAACTAAGATACTTGTTGTTCGACGGAAGGAAAGAAAAAGATCTAGTGCTAGTATACTATGCTTGTCCTTGCATGTCGTGCAAATTATGTGGCCAGATGTTGCTCCACGTAGTCGTTCAAGCTAATGTCACTGAGCGCGTGACCGCACCACCCCGCGCTAATCTTCTTCCGCGCCACGGCGGGCGCCGGGTATATATGCTCCGCGGCCTGCTCATCCAACCAAACTCCACCGCTCCACTCTCCAGCATCCATCTCTCCCAAGTCTCAGCGCAGCAGCTAATAAAACACGCTGCTTACTCCACAGTCGACCGGCTCCCGGCGACACTGCGATCGATCGATGGACGCCGCAGACGCCGCCTCCCCGTGTGATGGGCACAGGACGGTGTGGTCGGAGCCGCCGAAGCGGCCTGCCGGCCGGACCAAGTTCAAGGAGACGCGCCACCCGCTGTACCGCGGCGTGCGGCGCCGGGGCCCCGCAGGCCGGTGGGTGTGCGAGGTGCGCGTGCTCGGGATGAGGGGCTCCAGGCTCTGGCTCGGCACCTTCACCACCGCCGAGATGGCAGCGCGCGCGCACGACGCCGCCGTTCTCGCGCTCTCTGGCCGCGCCGCCTGTCTGAACTTCGCCGACTCTGCTTGGCGGATGCTCCCCCTCCTCGCCGGCCCGTTCAGCACCGCTAAGGAGATCAAGGATGCCGTCGCCGTCGCCGTCCTGGCGTTCCAAAGACAGCACCCGGTCGCGTCCACGGCGCCAATGTCCCCGGCACGGACAGCCGTTGACGAGAAGGAAGTCGATGGCTCGCCGGCGCCGAGCGCTCTGTTCATGTCCAGCGAGCTGTTGAATGAGCACTGGTTTGGCGGCATGGATGCCGGATCATGCTACTCGGAGGGCATGTTCATAGAGTCGCCGGATACCAGACCGTGGCGGGAAGACCTCGAGCTCGGTGGCGTCCAGACACCGCCATGGAGCTACTTGTTCGACTAAGCAGTTAAAGTATTTGGATGGGTTTTCTGCTTCTGTTCCACCAAATATGGGAGGAAACAGAGGGGAAATTTTTTCCCATATGTCCATACCTGCTCGTTCATTGTGAAAGCCTCTGCTGTCTATTTTCTGGGAGGCTGCATTTGTACTTGATTTTTTTCCTTTCTAGGACATCACTTTGAGTGCTTTTATGTTGTTGTTGTTGAGTTGAATCACTTGAGTGTTCTAGGGAATACAGAATGAGTACTTCTCAGCTATTATGGACCTCAAGCACTCCATGGTCGACATAAGATATTACTTATGAGAAAGAGAACAAGAAAATACACATTAAAGAACTTTCGGCGTCACAGCTTGACAATGATGGA

>HvDREB1.11

>Protein

MDVADIASPSGQQKQQGHRTVSSEPPKRPAGRTKFHETRHPLYRGVRRRGRVGQWVCEVRVPGIKGSRLWLGTFTNPEMAARAHDAAVLALSGRAACLNFADSAWRMRPVLATTGSFGFSSTREIKLAVAVAVVAFQQQQIILPVACPSPEAPASPSAALFYISSGDLLELDEEQWFGGMDAGSYYASLAQGMLVAPPDERARPENREHSGVETPIPLWSYLFDC

>cDNA

AGTTAAACAACACCACCGCTAAGTCCTCCGGCGCTTCTCCACACACAGCAACTTACTCAACCACGCACTCCAGTCAAGCGTCTCCACTAACACTAGCTAGCTCTAGAAATGGACGTCGCCGACATCGCCTCCCCGTCCGGCCAGCAGAAGCAGCAGGGGCACAGGACGGTGTCGTCGGAGCCGCCGAAGCGGCCCGCGGGGCGGACCAAGTTCCACGAGACGCGCCACCCGCTGTACCGCGGCGTGCGGCGCCGGGGCCGGGTCGGGCAGTGGGTGTGCGAGGTGCGCGTGCCCGGGATCAAGGGCTCCAGGCTCTGGCTCGGGACCTTCACCAACCCCGAGATGGCCGCGCGTGCCCACGACGCCGCGGTGCTCGCGCTCTCCGGCCGCGCCGCCTGCCTCAACTTCGCCGACTCCGCGTGGCGGATGCGGCCAGTGCTCGCGACCACCGGGTCGTTTGGCTTCAGCAGCACGCGGGAGATCAAGCTTGCCGTCGCCGTAGCCGTCGTCGCGTTCCAGCAGCAGCAGATTATTCTTCCAGTAGCGTGTCCATCGCCGGAGGCGCCCGCCAGCCCGAGCGCCGCTCTGTTTTACATCTCGTCCGGCGACCTGTTGGAGCTCGACGAGGAGCAGTGGTTTGGCGGCATGGACGCCGGGTCGTACTACGCGAGCTTGGCGCAGGGGATGCTCGTGGCGCCGCCGGACGAAAGAGCGAGGCCAGAGAACCGCGAGCACAGCGGCGTCGAGACACCAATACCACTATGGAGCTATTTGTTCGACTGCTAA

>CDS

ATGGACGTCGCCGACATCGCCTCCCCGTCCGGCCAGCAGAAGCAGCAGGGGCACAGGACGGTGTCGTCGGAGCCGCCGAAGCGGCCCGCGGGGCGGACCAAGTTCCACGAGACGCGCCACCCGCTGTACCGCGGCGTGCGGCGCCGGGGCCGGGTCGGGCAGTGGGTGTGCGAGGTGCGCGTGCCCGGGATCAAGGGCTCCAGGCTCTGGCTCGGGACCTTCACCAACCCCGAGATGGCCGCGCGTGCCCACGACGCCGCGGTGCTCGCGCTCTCCGGCCGCGCCGCCTGCCTCAACTTCGCCGACTCCGCGTGGCGGATGCGGCCAGTGCTCGCGACCACCGGGTCGTTTGGCTTCAGCAGCACGCGGGAGATCAAGCTTGCCGTCGCCGTAGCCGTCGTCGCGTTCCAGCAGCAGCAGATTATTCTTCCAGTAGCGTGTCCATCGCCGGAGGCGCCCGCCAGCCCGAGCGCCGCTCTGTTTTACATCTCGTCCGGCGACCTGTTGGAGCTCGACGAGGAGCAGTGGTTTGGCGGCATGGACGCCGGGTCGTACTACGCGAGCTTGGCGCAGGGGATGCTCGTGGCGCCGCCGGACGAAAGAGCGAGGCCAGAGAACCGCGAGCACAGCGGCGTCGAGACACCAATACCACTATGGAGCTATTTGTTCGACTGCTAA

>DNA

TTCCCTTCTCTCTCCTCTTCTCTCACATCCAACTCAGCAAAATATAATATTTTAATTCTTACAGCCTGCTGGCTGTACTTTATTGTACTTGCTCTTAGAAGAGTGTACTACTTTGTTATTATATGACCCACATTTTATTTTCATAAAGTGTCTAGAAGCATGTGCTAGAGCTGACTAATAACTAAGAGTCCGCTTACCTTCTCTTCTTTTCTCTTTCCTACAACTAAACAAAAATACAATACTTTATTTTTTATAACCAACTGACTAAACTCTATTGTATTTGATGATATGGTACATGCTTTCAGTTTCTTCCGGCCAATAGTGCTGGCGGTTGGCGTGTCCTTTTCGTGACCATAAGAGGCAAGCATAGACACAACACTGAGGTGGTGTTTCTTTTCAAAAGACTAGACTTTTTTTTAGTCCCAAGGACTAAAGAAAAAAGTCCTTTTAATAAAGTGTTTTTCTAGTTCATTAAGGAAAAGTTTCTCCTGTTTCTTTACTCATGGACTAAAAGAGACTTTTTAGTCTATTACTTAGGTAAAGAAACACTACTTGACTGGGCGATACATCTGTGGTCTGCTCGAGGTAAAGCTGCCGATCTCATGTCAAAATCCTTGTAGCTACGCGAGGGGGTCGCTCACCCAGTGGACGCCGCTGAGTGTGATGAAACAGGTCGCTCTCTGGGCCTCCTTTCTGAATCCTATGGCCCACAGGTCTCTCTCTGGGCCTCCTTCCTGAATCCTATGGCCAAGTGGGCATGACGCCCCCACGATATTTCTTCCTCTTCTTGTGGCAACCAAGCTGCCAACACGCCAAGAATGAGTGAGATATTTCAGTGGCCAGTGCTCACTGTGCTTCGGAAGGCGCAGTGATGTTTGCCCAAAAATAGAATGGAAGCCCCCTAAAATACACACCCGGGTAGTCGCCGCGCTCTCGCGCTATATATGCTTTGCCCCGCCGCAGCCCCTTCTTCTCAGTTAAACAACACCACCGCTAAGTCCTCCGGCGCTTCTCCACACACAGCAACTTACTCAACCACGCACTCCAGTCAAGCGTCTCCACTAACACTAGCTAGCTCTAGAAATGGACGTCGCCGACATCGCCTCCCCGTCCGGCCAGCAGAAGCAGCAGGGGCACAGGACGGTGTCGTCGGAGCCGCCGAAGCGGCCCGCGGGGCGGACCAAGTTCCACGAGACGCGCCACCCGCTGTACCGCGGCGTGCGGCGCCGGGGCCGGGTCGGGCAGTGGGTGTGCGAGGTGCGCGTGCCCGGGATCAAGGGCTCCAGGCTCTGGCTCGGGACCTTCACCAACCCCGAGATGGCCGCGCGTGCCCACGACGCCGCGGTGCTCGCGCTCTCCGGCCGCGCCGCCTGCCTCAACTTCGCCGACTCCGCGTGGCGGATGCGGCCAGTGCTCGCGACCACCGGGTCGTTTGGCTTCAGCAGCACGCGGGAGATCAAGCTTGCCGTCGCCGTAGCCGTCGTCGCGTTCCAGCAGCAGCAGATTATTCTTCCAGTAGCGTGTCCATCGCCGGAGGCGCCCGCCAGCCCGAGCGCCGCTCTGTTTTACATCTCGTCCGGCGACCTGTTGGAGCTCGACGAGGAGCAGTGGTTTGGCGGCATGGACGCCGGGTCGTACTACGCGAGCTTGGCGCAGGGGATGCTCGTGGCGCCGCCGGACGAAAGAGCGAGGCCAGAGAACCGCGAGCACAGCGGCGTCGAGACACCAATACCACTATGGAGCTATTTGTTCGACTGCTAATTTAGCACGCAGTGTAAAGTTGTTTACATAGTTGTGTTGTGTTCCTCTTTTTTTTTTTTTAGAAAAGGAGGATCTCATCCGATTCAATTGTGCTAAGTTAAACTTTGAGTTTGTGTTATGCTCAAAAATGCTACACCTACAAACACTTCTATCGTAAGCTCACTTAAAACAGTTATTTCCTTCCCT

>HvDREB1.12

>Protein

MSNPIQTDVAGIASPSGQQEQQGHRTVSSEPPKRPAGRTKFHETRHPLYRGVRRRGRVGQWVCEVRVPGIKGSRLWLGTFNTAEMAARAHDAAALALSGRAACLNFADSAWRMLPVLAAGSFGFDSAREVKAAVAVAVVAFQRRQIIPVAVAVVALQKQQVPVAVAVVTLQQKQQQVPVAVAVVALQQQQVPVAVAVVALQQLQVPVAVAVVALQEQQIILPVACLAPEFYMSSGDLLELDEEQWFGGMDAGSYYASLAQGMLVAPPDERARPEHGEQTGVQTPLWSCLFD

>cDNA

ATGTCGAATCCGATTCAGACGGACGTCGCCGGCATCGCCTCCCCGTCCGGCCAGCAGGAGCAGCAGGGGCACCGGACGGTGTCGTCGGAGCCGCCGAAGCGGCCCGCGGGGCGCACCAAGTTCCACGAGACGCGCCACCCTCTGTACCGCGGCGTGCGCCGCCGCGGCCGCGTCGGGCAGTGGGTGTGCGAGGTGCGCGTGCCCGGGATCAAGGGCTCCAGGCTCTGGCTCGGCACCTTCAACACGGCCGAGATGGCCGCCCGCGCGCACGACGCCGCCGCGCTCGCGCTCTCCGGCCGCGCCGCCTGCCTCAACTTCGCCGACTCCGCCTGGCGGATGCTGCCCGTGCTCGCGGCCGGGTCGTTCGGCTTCGACAGCGCGCGGGAGGTCAAGGCCGCCGTCGCCGTCGCCGTCGTGGCGTTCCAGCGGAGGCAGATTATTCCAGTCGCCGTCGCCGTCGTGGCGCTCCAGAAGCAGCAGGTTCCGGTCGCTGTCGCCGTCGTGACGCTCCAGCAGAAGCAGCAGCAGGTCCCGGTCGCCGTCGCCGTCGTGGCGCTCCAGCAGCAGCAGGTCCCGGTCGCCGTCGCCGTCGTGGCGCTCCAGCAGCTGCAGGTTCCGGTCGCCGTCGCCGTCGTGGCGCTCCAGGAGCAGCAGATTATTCTACCAGTCGCGTGCCTGGCGCCGGAGTTCTACATGTCTTCAGGCGACCTGCTGGAGCTCGACGAGGAGCAGTGGTTCGGCGGAATGGACGCCGGGTCGTACTACGCGAGCTTGGCGCAGGGGATGCTCGTGGCGCCGCCGGACGAAAGAGCGAGGCCGGAGCACGGCGAGCAGACCGGCGTCCAGACGCCGCTATGGAGCTGCTTGTTCGACTAATTTAGCACTACTGTCAACATGTAGATAGTTGCGTTCTTCCAGATTTGGGAGGAAAGAGAGTAGGCAGTTGGTACTACTTTTGGGGAAAAGGGGCTAGATTGCTTAATTCAACAATTGGTACTGAACTCGATTGTGTATCGTGCACTCCATGAATCGAAATCAAGGATTTTTGGGAGAAG

>CDS
ATGTCGAATCCGATTCAGACGGACGTCGCCGGCATCGCCTCCCCGTCCGGCCAGCAGGAGCAGCAGGGGCACCGGACGGTGTCGTCGGAGCCGCCGAAGCGGCCCGCGGGGCGCACCAAGTTCCACGAGACGCGCCACCCTCTGTACCGCGGCGTGCGCCGCCGCGGCCGCGTCGGGCAGTGGGTGTGCGAGGTGCGCGTGCCCGGGATCAAGGGCTCCAGGCTCTGGCTCGGCACCTTCAACACGGCCGAGATGGCCGCCCGCGCGCACGACGCCGCCGCGCTCGCGCTCTCCGGCCGCGCCGCCTGCCTCAACTTCGCCGACTCCGCCTGGCGGATGCTGCCCGTGCTCGCGGCCGGGTCGTTCGGCTTCGACAGCGCGCGGGAGGTCAAGGCCGCCGTCGCCGTCGCCGTCGTGGCGTTCCAGCGGAGGCAGATTATTCCAGTCGCCGTCGCCGTCGTGGCGCTCCAGAAGCAGCAGGTTCCGGTCGCTGTCGCCGTCGTGACGCTCCAGCAGAAGCAGCAGCAGGTCCCGGTCGCCGTCGCCGTCGTGGCGCTCCAGCAGCAGCAGGTCCCGGTCGCCGTCGCCGTCGTGGCGCTCCAGCAGCTGCAGGTTCCGGTCGCCGTCGCCGTCGTGGCGCTCCAGGAGCAGCAGATTATTCTACCAGTCGCGTGCCTGGCGCCGGAGTTCTACATGTCTTCAGGCGACCTGCTGGAGCTCGACGAGGAGCAGTGGTTCGGCGGAATGGACGCCGGGTCGTACTACGCGAGCTTGGCGCAGGGGATGCTCGTGGCGCCGCCGGACGAAAGAGCGAGGCCGGAGCACGGCGAGCAGACCGGCGTCCAGACGCCGCTATGGAGCTGCTTGTTCGACTAA

>DNA

GACGCATGTCAAAATCCTCGTCGCGAGCTCACTCAAGTGGAGGCAGGTGAGTGTGAGGAGGAAGCAGCTGCCTGTTTTGTCTGTGCCTGTTTCCTCAATTGTTGATCATATGGCCAAGTGGCAAGCTGCTAGGATATTTCTGCCTTCTTTGGGCAACAATGATGACGGAATGACGGGCCACTTTAGTTATGTGTATGTATATTTAATTACGTTGTCGATTTGTTCCGGGGCCTGGTGATTATTTCTTCCCCGGTGCCTTTTCCTCTATAAAATCACTGATGCCAGGATGACAAAGAGGTGACGTGTGCACTTTAGACACACGTATTTGGTTCCATGGACGATCGTTCACCTCGAAAATGATCTTGTCCTGGTCTTCGATGTGACTTGTATACTTGTATGTCGTGCGGCACGCAAAACACTACGCAGCTATGTGCCTGGGAACACTTAGAAAATGTATTTTTCTAGATGCGAAAGTGTGCATAAAACACAAAATGAACTCGTAACATAAATTGAAACAAATATGGGAACTGTTAGCATCCATAGATGGATTTATAATAATCATCCACCACCTTTTTAGCCGTTCGATGTATACGCGCGTACCGTCCGATCACCAGCTTGATCGTCTTCGTCCACCACCTAAAATCCCGAAACATCGCTTCAGTTGCAGAAAAAAGCACGCTGCTATGCGTCCGACTGACGGATTAAAAAGTGACTGACGGATCTTTTGCAACATAGATTATGTTGCAATTTTTTTTTGCAACAAAGAGCATGTTGCGAAAATGTCCGTATATTTTTTCGCGATAGGATTTGTTATAGAATATTTTCTGCAACAAGGATCGTGTTGCANNNNNTTTTGTAACAAAGGTCATGTTGCAAAAAACTTTTGCAACGTAGGTCAGGCCGTAGAAAACTTTTGCAACAAAAGATATTTGTTTTAATTTTTTGAAACATAGACCAAGTTGCGGAAACTCGTCATTACGTCTATTTGCACTAGCTACGTCGCTGTTAATCGCAACAACGTCGTTGTTGCAGGAACTCTGCGGATTTGCTCGAGGGATGGGGCTCGAGCTCGGGGCCAAACCAAAGAGCAGACATAGGGCGTCGGAGTCTAGGACGTTTGCATTGGCACCACCGAAGTTGGTTTTGTCCAACACAGAACACGCATCTGGTGGGAGAGGCGGTGGACGCGGTCGTTGAAATCCACCGGTTGATGAGAATAGTTTTCCAACAAATATGCATCTTAAATATGTCTTGTGCACACTTTTTTCGCTTCATCTGAATCGGAAAGACCAAAAAAAAAAAGGCATTGTTACTTCTTCGAACCATAATTTGGTTGACTGCATGAAACCGTGACTTGCTTGACTAGTTTAACATCTGGATCATGGTTATCAGGAACGCGATTCTGTTACACGATTCTACGATTTTTCAAACTAACCTTCGAATTATACAATTTTGGTTAGCTTCAAGCCTTCAATGTCACCACCAAAAGTCGTCAGTCACGGTCCGCCATCACCCACGACACCTAGCCCCGCCAGTGAACACCACACGACGATAAACCACCAAAAGTTGTGTTCATTCGCGGATGGCATGCTTTGTTTCCCCCCTTTGCACAACAAAGAAAAATAAAAGGACACTAGTTCGTTGTGGTGAATACGAGTATTTGTGAGCTACTAGTATGTTCAAACAAAGGCCACCACCCATCACATATGTCATCCAGCCAAAGCTTGAAAGGTAACCAAGGGATGCGCGCAGCGGAAACGATAGTAAAACAAAGGGTGGCGCGCCATGAAGCTGCCGTGTGTTCCGGCGCAAGTGTCACCGTGACTGTGACATACACGTACTACCTCTCCGCCACTTGCCATTTCCAGGAAGCCACCGCCTGCCTTTGCCTACTTATGCTCCCCCGTCTCCTCAAACAAGCTCCACCGATCTCGATCGAAACCTCTCACCACAGCCGCTGATTCTTCCAGTACAAAAACTACTCCACACCTCTCACGAGCATGTCGAATCCGATTCAGACGGACGTCGCCGGCATCGCCTCCCCGTCCGGCCAGCAGGAGCAGCAGGGGCACCGGACGGTGTCGTCGGAGCCGCCGAAGCGGCCCGCGGGGCGCACCAAGTTCCACGAGACGCGCCACCCTCTGTACCGCGGCGTGCGCCGCCGCGGCCGCGTCGGGCAGTGGGTGTGCGAGGTGCGCGTGCCCGGGATCAAGGGCTCCAGGCTCTGGCTCGGCACCTTCAACACGGCCGAGATGGCCGCCCGCGCGCACGACGCCGCCGCGCTCGCGCTCTCCGGCCGCGCCGCCTGCCTCAACTTCGCCGACTCCGCCTGGCGGATGCTGCCCGTGCTCGCGGCCGGGTCGTTCGGCTTCGACAGCGCGCGGGAGGTCAAGGCCGCCGTCGCCGTCGCCGTCGTGGCGTTCCAGCGGAGGCAGATTATTCCAGTCGCCGTCGCCGTCGTGGCGCTCCAGAAGCAGCAGGTTCCGGTCGCTGTCGCCGTCGTGACGCTCCAGCAGAAGCAGCAGCAGGTCCCGGTCGCCGTCGCCGTCTCGGCGCTCCAGCAGCAGCAGGTCCCGGTCGCCGTCGCCGTCGTGGCGCTCCAGCAGCTGCAGGTTCCGGTCGCCGTCGCCGTCGTGGCGCTCCAGGAGCAGCAGATTATTCTACCAGTCGCGTGCCTGGCGCCGGAGTTCTACATGTCTTCAGGCGACCTGCTGGAGCTCGACGAGGAGCAGTGGTTCGGCGGAATGGACGCCGGGTCGTACTACGCGAGCTTGGCGCAGGGGATGCTCGTGGCGCCGCCGGACGAAAGAGCGAGGCCGGAGCACGGCGAGCAGACCGGCGTCCAGACGCCGCTATGGAGCTGCTTGTTCAACTAATTTAGCACTACTGTCAACATGTAGATAGTTGCGTTCTTCCAGATTTGGGAGGAAAGAGAGTAGGCAGTTGGTACTACTTTTGGGGAAAAGGGGCTAGATTGCTTAATTCAACAATTGGTACTGAACTCGATTGTGTATCGTGCACTCCATGAATCGAAATCAAGGATTTTTGGGAGAAGAAAAACAGACACATCAAAGGACTTCAGTGTTGGTTGACAACCTGTGAAAATGATTGATTTGGACAGTTTTGCAAATCGTTCTCTTGCGACGATCCGCTAACGCTCCCTCGGATGCACCATTCCCATCACTTGCGTCAGACAAAGTGGTTTTTCGTGGGCGACGGTGTTGTGGATCTTGCGGTGAACTTGGTATGGGTAGTTCGTCGTTTTTGGCTTTTCCTCCTTGACGATTGTGCCGTCTCTTTCGTTTAGAGCGTTTTATACCTTTAGGTGCGTGTCGTCGCGTTTTAAATAATGTATTCTATAGTCAGATATTTTAAATTAAAAACTTTAAACTCATATTATTATATGTAGCATAAACTTAATGTAAATGAAGCTCGTCCAGCTCCATCGTGTCCATGTACGTCGGCCGACAGCGTCCCCTGTAGTGTCGGTCCGGTCGCCGGCAAGGTATAATAGGATACGGGACACTGGTCGGTTCACGGAAGTCAAGCTCCCGACGTCTCGCATGCCCTACTCTTCCTCGTCGGAGCCCGTAACCCGGACGGTGCCGGTAGACGTCAGCTCGATGATGGATCCTTCCTGAAAGGAGATGATGTCCACCACGATGCGGTCGCGGCGCCCGCACCCGTGCATTATACGGGGCGCTGGAGAGTGTGACTCCATCTCTCGAACGTCCACTGCCGCGAGGGCCTTCGCCGCCTCCCGCGCGGGATGAGCTCGTGGTCGATGGATCTGGAGGTAAAGGACTCGGATTCGTTGCCCGCCATGCTAGAGAAGGCTGGAGATCGCCGGAGGCGAGCTTTGATGGCGGATGGGAGTGGAGTGAAGTGGCTAGGGATTTGGTCCGATGAGCGGATGAAGACGGATATATGTAGGGTCGATACGGGCCATGCCGACGTGGCGGGCGTGCTCGGGCGCGCCCGGGCTCCCCCATATCCATTTCATATTTGGGGTTGGATATGAGTGGTGTCGGTCAGCCCGGGCGTTTGAGTCCCGTTTGAGGCGCCCGGTTAGGTTAAAATTTTGCTTTTCTGCTTGGCTCCTAGGGGAAGTTAGATTGA

>HvDREB2.1

>Protein

MVQPKKKFRGVRQRHWGSWVSEIRHPLLKRRVWLGTFETAEEAARAYDEAAVLMSGRNAKTNFPVQRSSTGDPAPAATRDVRGGSSSSSTSNLSQVLSAKLRKCCKAPSPSLTCLRLDTEKSHIGVWQKRAGARADSNWVMTVELNKGAGPSGDAVAQSTVSATTASSPASTMDDEERLTLQMIEELLSRSGPASPSHGEEGSFVV

>cDNA

GCGTAGGGACGTCCACGACCTAGTTAGTACTACTCTTGCCGTGCATTGTCAGGGCATCTATCCCGTTTGGGCAAAATTAATTTATGGACAGTAAAGTTAAGAGCGGCAAGATGTTCACAAATCTGAAGTTACTGCCATGAACTTTCAGGCTGCATGAAAAAGATCGAAAAAAGGCATAATTTATATGTGGAATACTTTGATGGAATTTGGAAAGTCTTGCAACCAAGCATGACGATCATAACACAGCAGTCAAGAGTCAACAATATGTCAGGAGATATAACTTTTAAATTTTCCATCAAGCAAAGGAAACATGAGCACGCGCCATCGTCGTCACCAGGCTGTACATGGAGCTAACCTTGGACAAGCTTAAATGCGAACCAGCTCCAAGCAAGCGATCCCAGAGCCAAAAATATCGGAGCAAAGGGAAGCTTGACAACTCCCCAGCAAGTCTTACTCAAAACCGCTCTATCAAGCCTAAAAGCAATCTATCTACACTCGCTTAAAAGCAACACTTTGTTGCACACTTGAACCCCCACAGTCCACGGCTCAAACAAGACTGGGTGTTGAAAGATGAAGAGGGGTTCCAAATGAAGCAAAAGAGCACTCAAAGAGGCCATCGCACACAAACCACAAAATACCCCGAAAACGCTAGTTTCCCAACACAAGTCTTACCATATTAACGATCCAAACAGAACAGAAGTCTTGACATATCAGTCTTTGCAGTTAAGTGCCAAGGGCCATGCGTCCCATGGCACTCAAACACATGATTGGATTGGCCATCTGGACCACGGCCATTAGCTGCAAAAGAACCAAGATGGCGAGCTCTCTTTCTGTATCTGGCTCGGTTTTACTTGCAACTTTGTCCTGGTTTGTAACATTATTGTAACTTTCATTAATTCGTTTAGCTCAAGGCTTCTGGTGAACAGCTGTTGGTACCTGACCTGTTGGGCCTTTAACATAATAATAAAAATAATAATAATAATAATAATCTCTGGATCTGATCTTTGAGAAGAAGGGGAAAACATGGCTGGTTGTAGGAGCTTAGGTGAATCAACTCTGCAAGTGCCAGGGCTGCCCAGCCCCACAATTAAATGTGGCTGGCCACGAGTGTAGGTGAGCGTGTTTATTACCGCTGTACCAGCAGGGAAGGTGGTTATGCCATGCATCTACTCAGTTCACCGACAGTCCACCCCTGCGGACCCCCTTCAGACTTCCGTTGCGCCTCCCCCGCTCTTCCGCCTCGGCACACGACTGCCATACTGGTGGTCGTACTAAGTGACTCCAGCAGCAAGGCACTCACGCTCTCCGGTGCATACCTTCCAATGCTGACCAGTGAAAATTAGACCTAGCACACTCTCTTCTGTGAGGGGGTGCGTGTGACACTGTGCCGCGTACTATTTGACTACCTACGCATCTAACCACATTTAACACGGCAGTGCAGAGGCAAGTACAGGCTGGGCAGCTTATATTCATCACTCGGACCAGAGCTCTCCTCCCCACCACCCACAACACCTTGGCGGACTCAGAGGCTCGTTCCCGCGTCTGTGGACGACAGACGGAACAAATGGTGCAACCCAAGAAGAAGTTCCGTGGAGTCAGGCAGCGCCACTGGGGCTCCTGGGTCTCCGAGATCAGACACCCCCTCCTTAAGAGGAGGGTGTGGCTTGGTACCTTTGAAACCGCTGAAGAGGCCGCACGGGCCTATGACGAGGCTGCCGTTCTGATGAGCGGACGCAATGCCAAGACCAACTTCCCCGTGCAGAGGAGCAGCACCGGCGATCCTGCCCCAGCTGCAACCCGGGACGTCCGTGGTGGCAGCTCCTCATCCTCGACAAGCAACCTGTCCCAGGTCCTCAGTGCCAAGCTTCGCAAGTGCTGCAAGGCGCCGTCTCCGTCCCTGACCTGCCTTCGCCTCGACACCGAGAAGTCCCACATTGGCGTCTGGCAGAAGCGCGCAGGGGCCCGCGCCGACTCCAACTGGGTCATGACCGTGGAGCTCAACAAAGGGGCCGGGCCATCTGGCGATGCAGTGGCGCAGTCCACAGTGTCAGCAACCACGGCTTCTTCACCGGCGAGTACAATGGATGACGAGGAGAGGCTCACGCTGCAGATGATCGAGGAGCTGCTGAGCAGGAGCGGTCCAGCTTCGCCTTCACATGGAGAAGAAGGTAGCTTCGTTGTCTGAAAAAGCATGGATGAAACAGCATCGCAACGTCGCCATCCGAGATCAGGATCCCGTAGACTGGTTGGTACGCAAGCAGTTCAGGTTGCTTCAGTAAGATAGTTATATGTCTATAACTCAGTCCAAGTTACAAAAACCTTAAGTTTGCAGTTGTGTGCAGCTATATGTACTTATATTTGTTATTACATACACAGTCACCGTCAAGATACTTTATTACACATCAATGTCAGCTATATAGTATATATAAAGGAAATAAATAGAATGTGTTTTGTGTTACCTCATGCCTTCATTCAACTTCAGACTCCAAACTTGGACTCTTAAGAGAAATCTAAATAACATGATGAAGGTGATATTTGTGAAGAAGACCTCAGCTCTATATGACAACCATTTCTAAGGACCATGGGCTCTAGAGGTTTATGTATACAGAAGAAAGCACCAACTATTTTGTTGTGTTGATGCAATGTGTGTGTGCATTTATTGTGAGGAAAAACATTTATAAGGGATATAAGACAAACCAGCAAACAGCCAAGCACCAATACACCTCAGTAAAAAAAAAATAGATGCAAATTTGTCCACGGAAAAATAAGGTATCAACCGGAGGCTACAACTTGGCATTGTGCCTCAGCTAGCACACTTTTCCATCATCTGCAGTCGTCACGAATTTCCTTCAAAAAAAGGGAGGTGAAATATTTCTGAATATGAGAAGGGTATGTATTTTCAGGAAGCTCTCTTCTGCCAGTTTATGTTAGTAATGTTGTCAACAACTCCACATCATGCAATCATATGCTCAACAACGTAGATGTATGAGTACGCATTTTTCATAATCCGAACTAAATCCTAAGAAATCCACTTAGAACTCTCCCAGGCCAAAGAAGTCTTAAGGGTGTGAGTGATCAACCCAACAATGGACCACAGAATAATTATTGGAGGACAGGTGTACAAAAAGACCTTAAGAGTCGTATTTCTCCTCATTCAATGACGAAATGCAATGCACAATAAGTGACAATTAATCATGTTAGGTTAGCTAGGATGAGCCCATGCTATAATGACCAGCAGATCCTTTGCCTTCAGACTAGGAACAACTGATAGCAGCCTTGGAGCTTCAACTAACAATCAAACAAATAGAACACAATCGCTAGACTGATTCAGCTCATGTCAATTATGCACTAAAAAGAAGGCCATCATATAGGGTGAGTTTGTGAAAATAGTTTATGTACCAGTGATGATGACCATTCATCATGGTAAGCCCAAAATGAGCAACTCTTGTCGACTTAGCAAATACAGAACTAGAAGAAACTTAATGGTCAGGGCTTGTTAGATCGGATGCTAACTCAACATTGGCAAGTAAAAGAGTTTATGGGTGTAAAAAGAACAGAAAGACACATCAAGAATGACGAGCAAGGTCCCCACATTCCTTCTTTGTCTTGCCAGACTTCAGAACATGGAGAAATGAGAAGGAATTTATCAGCTTCATGGTTGAGTTTAGAGGTTGGTACAGAGCAGGCATGCTTAAGGGCCCGGTCAGTTAGTTGGTCAGTCATTATAAACAATTGAATCAGAAAAGGAGAAAACAATAACACATATGGAAAAATACTGTCAACCATAGTTTCTGAATACTGAGTTACTAGGTCAGGGAATAAATTGCATTACACAAGTTTGGGTTCTTAAGATAGCTAAGTTGCTTACTTTCTTTATCATATCCCTGCCCGTACCTATATAAAGATGGAAATGTTTGAATGGAGACTGGCTGTCTTTTCACCTACCTTGTCTTACTCGGACCATCAGCAGATGTGTAATACTGGCCTTCAAAATTCCCGAACTCGACCGCCATTGCTGCCACAGTTTCCATCCTCACGGGGATCCTTCAAGCAACAACCAAAAGCTTTCTCCACCTGCTTCTTCAGACTTCAGCCAACTTTTGCAGTCATGTGCGTGTAGTATCATTTCAAAGTCATTCTTCGACCCTTTGCCGCAAGCTACCATGTAGCATCACTACACACCCCTCTTCACTCTTGACGCACAATAACAGAATAAACAACATCAAAGGATCCTGCTCTACAGGGACAGCTCTTAAGAGATTTATATCCTAAGTTGGTCTTTTCCATCTTCTATTTATACAAGGTTGCGAAGTGTCATATACTAATTGCTACTTGCTAAGGTATACTGTATATATTTACAGTCATGGACTTTAACATGCATGTGTATTGGGTAAGTTTGGGGACGCGAATTTGGTGAACATGGTTTCATGCATCCCTTATGAATAGTAAATTTGAAAAAATGCTAGAAAAAAAAATCTGAATTTATTTTTGTCATATACATAGTCAAACAGTATACTCGGGTATGAAGTTTCATGAAAAAATCACATCCGTGGTAATCTGCACAAAAATGGCAAAATCAAAGCTATATTAAAAAACATTGTTTGATGAATAGTATGGTCACAATTGTATTTTCTTCACTAAGAATACCATGGGTGTCAATACATCACAAAACTTCTCACGTGAGTAGAATGGTTGACTAAGTTTCTTACCGCCAAATTTCAATTTAAAAAAATCAGTATTTTTATGAATTTACTATTCACGTGGGTGCGCGTGATACCGTGTTCACCTTTGCATTTTCGGTGAATTTGGTGGCTAATTTCGACCACTCCGTCGTCC

>CDS

ATGGTGCAACCCAAGAAGAAGTTCCGTGGAGTCAGGCAGCGCCACTGGGGCTCCTGGGTCTCCGAGATCAGACACCCCCTCCTTAAGAGGAGGGTGTGGCTTGGTACCTTTGAAACCGCTGAAGAGGCCGCACGGGCCTATGACGAGGCTGCCGTTCTGATGAGCGGACGCAATGCCAAGACCAACTTCCCCGTGCAGAGGAGCAGCACCGGCGATCCTGCCCCAGCTGCAACCCGGGACGTCCGTGGTGGCAGCTCCTCATCCTCGACAAGCAACCTGTCCCAGGTCCTCAGTGCCAAGCTTCGCAAGTGCTGCAAGGCGCCGTCTCCGTCCCTGACCTGCCTTCGCCTCGACACCGAGAAGTCCCACATTGGCGTCTGGCAGAAGCGCGCAGGGGCCCGCGCCGACTCCAACTGGGTCATGACCGTGGAGCTCAACAAAGGGGCCGGGCCATCTGGCGATGCAGTGGCGCAGTCCACAGTGTCAGCAACCACGGCTTCTTCACCGGCGAGTACAATGGATGACGAGGAGAGGCTCACGCTGCAGATGATCGAGGAGCTGCTGAGCAGGAGCGGTCCAGCTTCGCCTTCACATGGAGAAGAAGGTAGCTTCGTTGTCTGA

>DNA

CAACACTCGAATTTAAGGAGATTTGGTTTGACCAGCTGAGATCCAAAACTCCAAACATTTCTAAAATGAGGTACTACTGCTACTACCACCACATTGTGAAATGGTCCTATTTCCTGGCTAGATGTACTGCTGCCAGTTTGAACTGGTGTGCTGCATGTACTTGCCATGAGGAAAGGGTTGCGTGCGGTGCAGACAGTGTCAGAAGAATAGATCGGTCACAGAAGCTTCCTATCCAGCAAAACCTGACACAGATGTCTGTGGAGCCTGCACATACCTGCTCCTCATTAACTGGGTCGACAGGGGTAACGGACTCCAGCAAGCAAATCCGATTCACTTCCACGCACAAAATAAAACGAAATCTGGGCAGTTGCTTGGTGAAATATGACAATTATTGCAGATCAACTGTTGGGCACTTAAATGTCGATATCACCAGACTGCGTAGGGACGTCCACGACCTAGTTAGTACTACTCTTGCCGTGCATTGTCAGGGCATCTATCCCGTTTGGGCAAAATTAATTTATGGACAGTAAAGTTAAGAGCGGCAAGATGTTCACAAATCTGAAGTTACTGCCATGAACTTTCAGGCTGCATGAAAAAGATCGAAAAAAGGCATAATTTATATGTGGAATACTTTGATGGAATTTGGAAAGTCTTGCAACCAAGCATGACGATCATAACACAGCAGTCAAGAGTCAACAATATGTCAGGAGATATAACTTTTAAATTTTCCATCAAGCAAAGGAAACATGAGCACGCGCCATCGTCGTCACCAGGCTGTACATGGAGCTAACCTTGGACAAGCTTAAATGCGAACCAGCTCCAAGCAAGCGATCCCAGAGCCAAAAATATCGGAGCAAAGGGAAGCTTGACAACTCCCCAGCAAGTCTTACTCAAAACCGCTCTATCAAGCCTAAAAGCAATCTATCTACACTCGCTTAAAAGCAACACTTTGTTGCACACTTGAACCCCCACAGTCCACGGCTCAAACAAGACTGGGTGTTGAAAGATGAAGAGGGGTTCCAAATGAAGCAAAAGAGCACTCAAAGAGGCCATCGCACACAAACCACAAAATACCCCGAAAACGCTAGTTTCCCAACACAAGTCTTACCATATTAACGATCCAAACAGAACAGAAGTCTTGACATATCAGTCTTTGCAGTTAAGTGCCAAGGGCCATGCGTCCCATGGCACTCAAACACATGATTGGATTGGCCATCTGGACCACGGCCATTAGCTGCAAAAGAACCAAGATGGCGAGCTCTCTTTCTGTATCTGGCTCGGTTTTACTTGCAACTTTGTCCTGGTTTGTAACATTATTGTAACTTTCATTAATTCGTTTAGCTCAAGGCTTCTGGTGAACAGCTGTTGGTACCTGACCTGTTGGGCCTTTAACATAATAATAAAAATAATAATAATAATAATAATCTCTGGATCTGATCTTTGAGAAGAAGGGGAAAACATGGCTGGTTGTAGGAGCTTAGGTGAATCAACTCTGCAAGTGCCAGGGCTGCCCAGCCCCACAATTAAATGTGGCTGGCCACGAGTGTAGGTGAGCGTGTTTATTACCGCTGTACCAGCAGGGAAGGTGGTTATGCCATGCATCTACTCAGTTCACCGACAGTCCACCCCTGCGGACCCCCTTCAGACTTCCGTTGCGCCTCCCCCGCTCTTCCGCCTCGGCACACGACTGCCATACTGGTGGTCGTACTAAGTGACTCCAGCAGCAAGGCACTCACGCTCTCCGGTGCATACCTTCCAATGCTGACCAGTGAAAATTAGACCTAGCACACTCTCTTCTGTGAGGGGGTGCGTGTGACACTGTGCCGCGTACTATTTGACTACCTACGCATCTAACCACATTTAACACGGCAGTGCAGAGGCAAGTACAGGCTGGGCAGCTTATATTCATCACTCGGACCAGAGCTCTCCTCCCCACCACCCACAACACCTTGGCGGACTCAGAGGCTCGTTCCCGCGTCTGTGGACGACAGACGGAACAAATGGTGCAACCCAAGAAGAAGTTCCGTGGAGTCAGGCAGCGCCACTGGGGCTCCTGGGTCTCCGAGATCAGACACCCCCTCCTGTAAGCCCCTCCCCTCCTTTCTAGCCTCCTTCCATTCCCGCCAAGTTAGTGGTTTCTTCGCCGGTTTACAAGATTCAAACTAACAGCATGCGTGTCTTTTGTGAAGTAAGAGGAGGGTGTGGCTTGGTACCTTTGAAACCGCTGAAGAGGCCGCACGGGCCTATGACGAGGCTGCCGTTCTGATGAGCGGACGCAATGCCAAGACCAACTTCCCCGTGCAGAGGAGCAGCACCGGCGATCCTGCCCCAGCTGCAACCCGGGACGTCCGTGGTGGCAGCTCCTCATCCTCGACAAGCAACCTGTCCCAGGTCCTCAGTGCCAAGCTTCGCAAGTGCTGCAAGGCGCCGTCTCCGTCCCTGACCTGCCTTCGCCTCGACACCGAGAAGTCCCACATTGGCGTCTGGCAGAAGCGCGCAGGGGCCCGCGCCGACTCCAACTGGGTCATGACCGTGGAGCTCAACAAAGGGGCCGGGCCATCTGGCGATGCAGTGGCGCAGTCCACAGTGTCAGCAACCACGGCTTCTTCACCGGCGAGTACAATGGATGACGAGGAGAGGCTCACGCTGCAGATGATCGAGGAGCTGCTGAGCAGGAGCGGTCCAGCTTCGCCTTCACATGGAGAAGAAGGTAGCTTCGTTGTCTGAAAAAGCATGGATGAAACAGCATCGCAACGTCGCCATCCGAGATCAGGATCCCGTAGACTGGTTGGTACGCAAGCAGTTCAGGTTGCTTCAGTAAGATAGTTATATGTCTATAACTCAGTCCAAGTTACAAAAACCTTAAGTTTGCAGTTGTGTGCAGCTATATGTACTTATATTTGTTATTACATACACAGTCACCGTCAAGATACTTTATTACACATCAATGTCAGCTATATAGTATATATAAAGGAAATAAATAGAATGTGTTTTGTGTTACCTCATGCCTTCATTCAACTTCAGACTCCAAACTTGGACTCTTAAGAGAAATCTAAATAACATGATGAAGGTGATATTTGTGAAGAAGACCTCAGCTCTATATGACAACCATTTCTAAGGACCATGGGCTCTAGAGGTTTATGTATACAGAAGAAAGCACCAACTATTTTGTTGTGTTGATGCAATGTGTGTGTGCATTTATTGTGAGGAAAAACATTTATAAGGGATATAAGACAAACCAGCAAACAGCCAAGCACCAATACACCTCAGTAAAAAAAAAATAGATGCAAATTTGTCCACGGAAAAATAAGGTATCAACCGGAGGCTACAACTTGGCATTGTGCCTCAGCTAGCACACTTTTCCATCATCTGCAGTCGTCACGAATTTCCTTCAAAAAAAGGGAGGTGAAATATTTCTGAATATGAGAAGGGTATGTATTTTCAGGAAGCTCTCTTCTGCCAGTTTATGTTAGTAATGTTGTCAACAACTCCACATCATGCAATCATATGCTCAACAACGTAGATGTATGAGTACGCATTTTTCATAATCCGAACTAAATCCTAAGAAATCCACTTAGAACTCTCCCAGGCCAAAGAAGTCTTAAGGGTGTGAGTGATCAACCCAACAATGGACCACAGAATAATTATTGGAGGACAGGTGTACAAAAAGACCTTAAGAGTCGTATTTCTCCTCATTCAATGACGAAATGCAATGCACAATAAGTGACAATTAATCATGTTAGGTTAGCTAGGATGAGCCCATGCTATAATGACCAGCAGATCCTTTGCCTTCAGACTAGGAACAACTGATAGCAGCCTTGGAGCTTCAACTAACAATCAAACAAATAGAACACAATCGCTAGACTGATTCAGCTCATGTCAATTATGCACTAAAAAGAAGGCCATCATATAGGGTGAGTTTGTGAAAATAGTTTATGTACCAGTGATGATGACCATTCATCATGGTAAGCCCAAAATGAGCAACTCTTGTCGACTTAGCAAATACAGAACTAGAAGAAACTTAATGGTCAGGGCTTGTTAGATCGGATGCTAACTCAACATTGGCAAGTAAAAGAGTTTATGGGTGTAAAAAGAACAGAAAGACACATCAAGAATGACGAGCAAGGTCCCCACATTCCTTCTTTGTCTTGCCAGACTTCAGAACATGGAGAAATGAGAAGGAATTTATCAGCTTCATGGTTGAGTTTAGAGGTTGGTACAGAGCAGGCATGCTTAAGGGCCCGGTCAGTTAGTTGGTCAGTCATTATAAACAATTGAATCAGAAAAGGAGAAAACAATAACACATATGGAAAAATACTGTCAACCATAGTTTCTGAATACTGAGTTACTAGGTCAGGGAATAAATTGCATTACACAAGTTTGGGTTCTTAAGATAGCTAAGTTGCTTACTTTCTTTATCATATCCCTGCCCGTACCTATATAAAGATGGAAATGTTTGAATGGAGACTGGCTGTCTTTTCACCTACCTTGTCTTACTCGGACCATCAGCAGATGTGTAATACTGGCCTTCAAAATTCCCGAACTCGACCGCCATTGCTGCCACAGTTTCCATCCTCACGGGGATCCTTCAAGCAACAACCAAAAGCTTTCTCCACCTGCTTCTTCAGACTTCAGCCAACTTTTGCAGTCATGTGCGTGTAGTATCATTTCAAAGTCATTCTTCGACCCTTTGCCGCAAGCTACCATGTAGCATCACTACACACCCCTCTTCACTCTTGACGCACAATAACAGAATAAACAACATCAAAGGATCCTGCTCTACAGGGACAGCTCTTAAGAGATTTATATCCTAAGTTGGTCTTTTCCATCTTCTATTTATACAAGGTTGCGAAGTGTCATATACTAATTGCTACTTGCTAAGGTATACTGTATATATTTACAGTCATGGACTTTAACATGCATGTGTATTGGGTAAGTTTGGGGACGCGAATTTGGTGAACATGGTTTCATGCATCCCTTATGAATAGTAAATTTGAAAAAATGCTAGAAAAAAAAATCTGAATTTATTTTTGTCATATACATAGTCAAACAGTATACTCGGGTATGAAGTTTCATGAAAAAATCACATCCGTGGTAATCTGCACAAAAATGGCAAAATCAAAGCTATATTAAAAAACATTGTTTGATGAATAGTATGGTCACAATTGTATTTTCTTCACTAAGAATACCATGGGTGTCAATACATCACAAAACTTCTCACGTGAGTAGAATGGTTGACTAAGTTTCTTACCGCCAAATTTCAATTTAAAAAAATCAGTATTTTTATGAATTTACTATTCACGTGGGTGCGCGTGATACCGTGTTCACCTTTGCATTTTCGGTGAATTTGGTGGCTAATTTCGACCACTCCGTCGTCC

>HvDREB2.2

>Protein

MVQSKKKFRGVRQRHWGSWVSEIRHPLLKRRVWLGTFETAEEAARAYDEAAILMSGRNAKTNFPVPRSANGEIIVAPAAAARDIRGGVGSSSSGAAGASSLSQILSAKLRKCCKTPSPSLTCLRLDTEKSHIGVWQKRAGARADSSWVMTVELNKEPAAAAPPTPSDSTVSATPSSSTSTSTTGSPPEAMEDEERIALQMIEELLSRSSPASPSHGLLHGEEGSLLI

>cDNA

CAGTAGCTTATATTCACTGGGAGCAGCAGGGCCTTCGCTCCTCCCCACACTAGACTGCTCCGCCTCTCTTCCGTGGAGGAGGCCGGGCTCACACTCACTCACTCACTCACTCGCACTGCGCCACACTGCCTGCTGATCATTCCTTCCCTCCGCTCTGCCCGTCCTCCTCCCTGTCAGAGAGAGAATCTCGCTCGCTCTGCATTCCGCAGAGAAACATCATGGTACAGTCCAAGAAGAAGTTTCGCGGCGTCAGGCAGCGCCACTGGGGCTCCTGGGTCTCCGAGATCAGGCATCCTCTCCTGAAGAGGAGGGTGTGGTTGGGCACCTTTGAGACGGCGGAGGAGGCTGCGCGGGCGTACGATGAGGCTGCCATCCTGATGAGCGGGCGCAACGCCAAGACCAACTTCCCCGTACCGAGGAGTGCCAACGGGGAGATCATCGTCGCCCCAGCAGCAGCAGCACGGGACATTCGCGGTGGCGTTGGCTCGTCGTCCTCCGGGGCCGCCGGCGCCAGCAGCCTGTCACAGATCCTCAGCGCCAAGCTCCGCAAGTGCTGCAAGACACCGTCCCCGTCCCTCACCTGCCTCCGCCTCGACACCGAGAAGTCCCACATTGGCGTCTGGCAGAAGCGCGCGGGTGCCCGTGCCGACTCCAGCTGGGTCATGACCGTCGAGCTCAACAAGGAGCCGGCCGCAGCGGCACCACCAACGCCCAGCGACAGCACGGTGTCGGCGACTCCTTCCTCGTCCACGTCCACGTCCACAACGGGCTCCCCACCGGAGGCAATGGAGGACGAAGAGAGGATCGCGCTGCAGATGATAGAGGAGCTGCTGAGCAGGAGCAGCCCGGCTTCGCCGTCACATGGGCTGCTGCACGGTGAAGAAGGCAGCCTCCTCATCTGAAGAAAAATATTGCACGGTTAAGAAAGTGTGATCAGGTCACCATCCCAGATCAAGGATCTGGTAGGGTGGTTGGCGCACAAGCAGTTAAGATCATTGCTCCACATCGTAGGTACCAGCCGAGTATCTCTCCATTACGCACTACGTAAAATCAAGCTTAGGAAACGATTAATTACTACTGTGTATGTACGCGTGTGTGTGAAGCCCTGTGTATTTATAAATTAATCAAAGGCTTACTTGTATGTAACTAAGTATATGCCGTCACCGTCATGATAGTCACACTATGTATCAACATCATCAGACTATATAGTACTACATGATTACGTATATATCAATGATATCGCTTTCTAATAAATGTAATAAACACCTTGTAATGCAGTACTCTTGTGCATTTGATTTCCTTTGTTAGAG

>CDS

ATGGTACAGTCCAAGAAGAAGTTTCGCGGCGTCAGGCAGCGCCACTGGGGCTCCTGGGTCTCCGAGATCAGGCATCCTCTCCTGAAGAGGAGGGTGTGGTTGGGCACCTTTGAGACGGCGGAGGAGGCTGCGCGGGCGTACGATGAGGCTGCCATCCTGATGAGCGGGCGCAACGCCAAGACCAACTTCCCCGTACCGAGGAGTGCCAACGGGGAGATCATCGTCGCCCCAGCAGCAGCAGCACGGGACATTCGCGGTGGCGTTGGCTCGTCGTCCTCCGGGGCCGCCGGCGCCAGCAGCCTGTCACAGATCCTCAGCGCCAAGCTCCGCAAGTGCTGCAAGACACCGTCCCCGTCCCTCACCTGCCTCCGCCTCGACACCGAGAAGTCCCACATTGGCGTCTGGCAGAAGCGCGCGGGTGCCCGTGCCGACTCCAGCTGGGTCATGACCGTCGAGCTCAACAAGGAGCCGGCCGCAGCGGCACCACCAACGCCCAGCGACAGCACGGTGTCGGCGACTCCTTCCTCGTCCACGTCCACGTCCACAACGGGCTCCCCACCGGAGGCAATGGAGGACGAAGAGAGGATCGCGCTGCAGATGATAGAGGAGCTGCTGAGCAGGAGCAGCCCGGCTTCGCCGTCACATGGGCTGCTGCACGGTGAAGAAGGCAGCCTCCTCATCTGA

>DNA

CGGGTGTCCTGCATGGACGGAGTGGCATTGGAACTGGCGTAGTTAAACCCGCAGTGCGTGTGTCAGTGCATGGCACAGATTTGTTACTCGTCCTGGATGTAGCTCGCACTTGACTGCCTGATGAGTAACCACGTACGTCCATCGATCATGGGCACGTCGTATCGTGCATTCACGCATGCTTTATTGTTGTGGAGTAGGACCGGTACTGTACTCCACTCCTCTTCTCCCTTGGGGAGTCTTTTCCTACTGCACTAGCTTGAGTGTCAGAAATCAGCCTTGATCATCAATTAATTTCTTAGTATGGTGAGTTATTTGTGATACTATAAAATTGGCAAATACAGTTTGATCCATGTTCAAACAATTTCATATGCTTGTCATTTCATCCATGTACTACAAGGAATTTATAGGAGTAGCCCTTAGCGACAAATGACAACCAGCTGAGTATATAGGAATAGCACTGAGCACTTAGGACATCTTCAACGTTAACCACTAAAACAGACACACAATGAGAGGAAATGTCCGGTCCTAAATATAAGTCTTTTTAAAGATTTCACTAAGAGACTACATACGGAACAAAATGAGCGAATCTACACTCTATAATATGTCTATATACATCCATATGTAGTTTCCTAGTAAAATCTCTTAAAAGACTTNNNTTTAGAAACGGAGGGAGTAGTTCGTTGGAACTTGAATGTCTAGACCAGCGTGCGGAAGTTTGAGAGATAGCATTAGATGGATGGCAAAAAGTGTCAAGATGCCCTTAGCAATAAGAGTGATTTCGAAAACCAAATGGGATTACCACAAAGAGCTGGACGGGTTGGATCCATGGCTCCGGCTCGTCTCTTGCAAATGAGGTCTCCCACGCCATAAATGAGGTCGCCAAGCGTGTAGGTGGGGGAGTTAATTACCGGGGTTACGTACTGGCAGGCAGCGGGGAGGGAGTGGTTACGTTGCCACTCGGCCACAGTTCACCGACAGCCCACCCCAGCAGCGCAACGGGGGGCCCCTCTTCCCTTCCCCCAGCAAGCTCTCGCCGGAGAGATCTTTGATCTTTCTTCTCTCCTTTGGTCTTTGTCTTTCCCTCTGGCTGGGCTGAGCTTTATCACTTGCTACACGCATGCTGTTACAGCTTCCAACCCCACTGCTCCAACCCCCTCCATGGATTACAGCCTTATACAGACAGAGTAGGGGATTGCTAGCTGAATTGCCTGCCCTTTAGGCACCACTACATATGTTGTATACTTGTATTCATATATGGTAGTCAGGTAGTCACTAATGTCACAACTTCCATAGGGAGTGGTGAATTTCAACCTGAAACTGGAGTTGTAACTTGTAGGTCCCGGAAGTAACCATGTCCCAGTAAGATGATGGATAGATTCGGTGTGTGTACTGCATGGTAGTGACACGAAATCTCTGAGAAGCTTAGAGTGGGTGACGCACATGACAGATTGACAGTACCCCAATGTCCTAGCGGAGTACAAGTGAAACAAGGACGGGCATGCATGGAAGCAGCCCAACCATCAACAGATACGTGGAACGCAAGGCACACGTACTCCCAGTCAGTTGAAGTTACTGCCACCCCGGCAAAATCCAATCGATCTCTCTCCCAGTCACACACATGCATACGCCATGTGGCCCTATGTGTATGTATGTATGTATGTATNNNNNNNNNTATGTATGTATGTATGTATGTATGCAAGCGTAGGTGTATACGCATCCGCCTATCCATCTTTCTATCTAACCACATTTAACGCTCTGCAAGTACAGGGGGGGTCCGAGGTGCAGTAGCTTATATTCACTGGGAGCAGCAGGGCCTTCGCTCCTCCCCACACTAGACTGCTCCGCCTCTCTTCCGTGGAGGAGGCCGGGCTCACACTCACTCACTCACTCACTCGCACTGCGCCACACTGCCTGCTGATCATTCCTTCCCTCCGCTCTGCCCGTCCTCCTCCCTGTCAGAGAGAGAATCTCGCTCGCTCTGCATTCCGCAGAGAAACATCATGGTACAGTCCAAGAAGAAGTTTCGCGGCGTCAGGCAGCGCCACTGGGGCTCCTGGGTCTCCGAGATCAGGCATCCTCTCCTGTAAGCCTCTCGCTAGCTCTCTCTCTCTCTATAGCTGCTACCCCTACTCTCCGGCCTACGACTCGTGTGGCTATCCCACCGTGTCCTGAGAGATAGATACATTGACTCTAGTTTGAATTGCCAACTGCTTTAAGGCTGCTTTCGTTGATTTTCTCTTGATGTGTTCAAAATGCATGCATGTATGTGTGTATGGTTTACAGGAAGAGGAGGGTGTGGTTGGGCACCTTTGAGACGGCGGAGGAGGCTGCGCGGGCGTACGATGAGGCTGCCATCCTGATGAGCGGGCGCAACGCCAAGACCAACTTCCCCGTACCGAGGAGTGCCAACGGGGAGATCATCGTCGCCCCAGCAGCAGCAGCACGGGACATTCGCGGTGGCGTTGGCTCGTCGTCCTCCGGGGCCGCCGGCGCCAGCAGCCTGTCACAGATCCTCAGCGCCAAGCTCCGCAAGTGCTGCAAGACACCGTCCCCGTCCCTCACCTGCCTCCGCCTCGACACCGAGAAGTCCCACATTGGCGTCTGGCAGAAGCGCGCGGGTGCCCGTGCCGACTCCAGCTGGGTCATGACCGTCGAGCTCAACAAGGAGCCGGCCGCAGCGGCACCACCAACGCCCAGCGACAGCACGGTGTCGGCGACTCCTTCCTCGTCCACGTCCACGTCCACAACGGGCTCCCCACCGGAGGCAATGGAGGACGAAGAGAGGATCGCGCTGCAGATGATAGAGGAGCTGCTGAGCAGGAGCAGCCCGGCTTCGCCGTCACATGGGCTGCTGCACGGTGAAGAAGGCAGCCTCCTCATCTGAAGAAAAATATTGCACGGTTAAGAAAGTGTGATCAGGTCACCATCCCAGATCAAGGATCTGGTAGGGTGGTTGGCGCACAAGCAGTTAAGATCATTGCTCCACATCGTAGGTACCAGCCGAGTATCTCTCCATTACGCACTACGTAAAATCAAGCTTAGGAAACGATTAATTACTACTGTGTATGTACGCGTGTGTGTGAAGCCCTGTGTATTTATAAATTAATCAAAGGCTTACTTGTATGTAACTAAGTATATGCCGTCACCGTCATGATAGTCACACTATGTATCAACATCATCAGACTATATAGTACTACATGATTACGTATATATCAATGATATCGCTTTCTAATAAATGTAATAAACACCTTGTAATGCAGTACTCTTGTGCATTTGATTTCCTTTGTTAGAG

>HvDREB2.3

>Protein

MARPQQRYRGVRQRHWGSWVSEIRHPLLKTRIWLGTFETAEDAARAYDEAARIMCGPRVRTNFPDNDAAPSSSFLSPALVAKLHRFNVACGPQAAQQGDKGASASSVGVEPRTSPTPFAGYTGNGASAPSAAAGWSGGFLEEQYVEQMIEELLDSNFSMEISY

>cDNA

CACCACACTAATCACCATCACCAGCGCCATCGTCTTCTCCGAGCCCCGAGACACAGTGCCGAGACACGCTAGCGGGCAGCACGGAGGCAGCTGGTTCCACTCTCACAGAGCAAGCGAAGAGCTCCTTCTCCAATGGCGCGGCCACAGCAGCGGTACCGCGGCGTCCGGCAGCGCCACTGGGGCTCCTGGGTCTCCGAGATCCGCCACCCCCTCCTCAAGACGAGGATCTGGCTGGGCACCTTCGAGACGGCGGAGGACGCGGCGCGCGCCTACGACGAGGCGGCGCGCATCATGTGCGGCCCGCGCGTGCGCACCAACTTCCCCGACAACGACGCCGCCCCGTCGTCGTCGTTCCTCTCCCCGGCCCTGGTCGCCAAGCTCCACCGCTTCAACGTGGCGTGCGGCCCGCAGGCCGCGCAGCAGGGGGACAAGGGCGCCTCGGCGTCGTCCGTGGGCGTGGAGCCGCGCACGTCGCCGACGCCGTTCGCCGGCTACACGGGCAATGGCGCCAGCGCGCCGTCGGCGGCGGCCGGGTGGAGCGGGGGGTTCCTGGAGGAGCAGTACGTGGAGCAGATGATCGAGGAGCTGCTGGACTCCAACTTCTCCATGGAGATCTCCTACTAGCTCCGCTCCGCCACCTCCGCGCATGCTCTGCTCCCGCGTCTTCCGTCCGCTTGCTCTGTTTCCGCTCCATTTCCCTTTCTCTTCCTCTTTCTCTCTCTCTCTCTGTGTGGGTGGGTTTGTTTTTAGCTTAGCATAAGCCATGTCGGGAAGAGCTCGAGGTTAGTATGGTCATCACCCGAGCTCTCCAGCGGCTTTGTGTATACAGTAGCTTCCTTAGAGCACATTTCTTCATTCTTGTATTACTATTCATGTCTTGATGATGCCCTAATTACTGTTTCGATCTTCGTGTTAAGCTTTGATGACAC

>CDS

ATGGCGCGGCCACAGCAGCGGTACCGCGGCGTCCGGCAGCGCCACTGGGGCTCCTGGGTCTCCGAGATCCGCCACCCCCTCCTCAAGACGAGGATCTGGCTGGGCACCTTCGAGACGGCGGAGGACGCGGCGCGCGCCTACGACGAGGCGGCGCGCATCATGTGCGGCCCGCGCGTGCGCACCAACTTCCCCGACAACGACGCCGCCCCGTCGTCGTCGTTCCTCTCCCCGGCCCTGGTCGCCAAGCTCCACCGCTTCAACGTGGCGTGCGGCCCGCAGGCCGCGCAGCAGGGGGACAAGGGCGCCTCGGCGTCGTCCGTGGGCGTGGAGCCGCGCACGTCGCCGACGCCGTTCGCCGGCTACACGGGCAATGGCGCCAGCGCGCCGTCGGCGGCGGCCGGGTGGAGCGGGGGGTTCCTGGAGGAGCAGTACGTGGAGCAGATGATCGAGGAGCTGCTGGACTCCAACTTCTCCATGGAGATCTCCTACTAG

>DNA

TAGTCTAAGGGAATATTATTAACACTAGTTCCTGACTTCTGGGTATAGTCTAGCTCACATGGTGTTCTCTAGCTTACATGGAATATATTGATTTCTTACTCATTTGTCCAGAGAAATATGTGTCCTCGTAGAACATGCGATCACATCTTTTTTATATCTTTATGGCTTCAGAAAATATTTACATTTGCCACTTGTTCTTTTTCAAAGAAAACCACAGGGAGAACCCTCCAGGATGAACCGAAATTCGCATCCTATGAGACATCCACTCCTCAACTAAGCGAGATAGGATCCTTATTGATCACTTGAAAATGATACCATTAAATATGTCTACATATCTTCATGACAGTAGGGACAACCCTGTGCACATAGCTCCCGGTTAGGGAAAAGGTCTGACCAATTTGGGTCTTTGTACACAACCTTCCCCTCTGTAGTTATGCAAGAGGTTGTTTCTAGGGCTCAAACCAGTGATCTGCTTGTCACAAGGCAACATGTTTAGTATTGCGCCGAAGCTTCCCTTCATCTTCATGACAGTAGTTATGTAAAAAATAAAGTGCAAGCAAAGATGAAAAGGATGACAATAATGCACATTGTTTGAAATGTTGCTTGTGGTGGAACAATTTGGTAACAAAGACAAAATGGACGAAAAACTATTTAATAAGTAGGATACCACGAGTATTTCTTCCATCATACTCTCAATTTATAGCTAATGTGTTTTCTCAATTGTTCTAAAATATATTACCATCTCGAAAACTTAAATTAAATGGTTACAAATTTTGTTACCCAAACTACAACCATCTTGCCAATTATCCCAAAGCACACACAATTCATCGGTAACATCACAATCAATCCCAATGCCAAATAAGGCTTAGAAAACCTAAATTTAAATAAATGACTCTTCACATTTTAAACAAATGTTTTTGTTAATTGCTCGCCCAATATAGCACATGCATGATTTTATTACTATCTCTGCTAAAATGGACTATACAATTGGAAACTCACAATCAAACTACTCTAATATCAATAACTAACTAGTAGAGTATTTCAATTGGATTAAGATATAATATTTATTTTCATGACATAATTTCGTAATTAAAAATAATGGTCTTTTGCTATCATTCCTACAAACAATAATGGTCAAATTTGTGTAGGGAATTCGTAAATGTTTTAAAACTCATCAATTTTTTAGTCAAAAGGAGTTCTATACTCATTTTTTAATGATCAATGTGTGGTTACTGAATCTTGATATCTGTAAATTTAATAGCCATAGAAAAAATTGTGAAACATATAGTACTAGTATGTAAACACATAAAATCTTATTAACCAAACATGCTTATCATAATTAACCAAACTGATGCAGAGGTTAAGAGGTAAAACCTTCATTTTCAAAAATATTTAGGTGCTAGCCAGCCAACCGAGATCACTAGTGCTTCTAAGAGTTTCGAGCATTTTTCCTTGGATCCTGATAAATTCTAAATGTTCGGACTTAAGCATGGTACCTCAAACATTTCAATGCAATTTCAGTTGATGTGAGGTGGTAACTTTAATTTGCAAACATGGTAATTTTGTCTTGTCAGAAAATTGTCATGTTTGCCAACTAAACGTGTCACCTCACGCCAACTAAATTTGCCATAAAAAATGTTTGGGCTGTCATTCTTAAAATCCAAACGTTCGGGGTTTATAATTTCCTTTCCCTTTTGGTGTTTCTGAGTTTTTTGTGTGTGCAATTTCTAACTTGATCAAGGTGAGCCAGGGGTGACATAAACACAGGCTGGGACCCACCGAGAGGCCGTTCAAAACAGGCCGGGAACTGTATTTCCTCCTTCCTCCCTTGTGTCTATTTATAGCGCTCAAAAGCTCGCAGTCGCACACACCACACTAATCACCATCACCAGCGCCATCGTCTTCTCCGAGCCCCGAGACACAGTGCCGAGACACGCTAGCGGGCAGCACGGAGGCAGCTGGTTCCACTCTCACAGAGCAAGCGAAGAGCTCCTTCTCCAATGGCGCGGCCACAGCAGCGGTACCGCGGCGTCCGGCAGCGCCACTGGGGCTCCTGGGTCTCCGAGATCCGCCACCCCCTCCTGTACGTCGCCGTCGTCGAAAGGCTCTGCGATTGCTCCGGATCGACCGGTTCTCTTGCGTGGCCTGACCATGGCGGTTTTCTTTCCTTGCAGCAAGACGAGGATCTGGCTGGGCACCTTCGAGACGGCGGAGGACGCGGCGCGCGCCTACGACGAGGCGGCGCGCATCATGTGCGGCCCGCGCGTGCGCACCAACTTCCCCGACAACGACGCCGCCCCGTCGTCGTCGTTCCTCTCCCCGGCCCTGGTCGCCAAGCTCCACCGCTTCAACGTGGCGTGCGGCCCGCAGGCCGCGCAGCAGGGGGACAAGGGCGCCTCGGCGTCGTCCGTGGGCGTGGAGCCGCGCACGTCGCCGACGCCGTTCGCCGGCTACACGGGCAATGGCGCCAGCGCGCCGTCGGCGGCGGCCGGGTGGAGCGGGGGGTTCCTGGAGGAGCAGTACGTGGAGCAGATGATCGAGGAGCTGCTGGACTCCAACTTCTCCATGGAGATCTCCTACTAGCTCCGCTCCGCCACCTCCGCGCATGCTCTGCTCCCGCGTCTTCCGTCCGCTTGCTCTGTTTCCGCTCCATTTCCCTTTCTCTTCCTCTTTCTCTCTCTCTCTCTGTGTGGGTGGGTTTGTTTTTAGCTTAGCATAAGCCATGTCGGGAAGAGCTCGAGGTTAGTATGGTCATCACCCGAGCTCTCCAGCGGCTTTGTGTATACAGTAGCTTCCTTAGAGCACATTTCTTCATTCTTGTATTACTATTCATGTCTTGATGATGCCCTAATTACTGTTTCGATCTTCGTGTTAAGCTTTGATGACAC

>HvDREB2.4

>Protein

MELHFQVQPAVLQLQDYCYYYQHQQQEAAAVQAKPTKPRGRKKGSTSHSKFVGVRQRPSGRWVAEIKDTTQKIRMWLGTFETADAAARAYDEAARLLRGAEARTNFAPRISPDCPLAVRIRGLLHHKKLKKARLPAASANIPGPSSTPAAPATYAPSNSNSNSNSNSNSNSMDGGACGGASSSSSSSAVSCDGAMKQGGGEVYRPDFAPVAGAEELESWMFESSFGQFPALDGFAAVDACTLPAASPEETSASGMVEFERMKVERRISASLYAMNGLQEYFDKVFEASAGDPLWDLSPLCQ

>cDNA

CTGAAAGTAACCCCACACGAGAGGGCAGGATCACTGCCCATACATTCCTCCTACTCCTATCTGAAAATTTCAAACCGTTCCCAAAGCCCCTATAAATCCTCCCATTTGCCAAGCAACCAGGGCAAACACTCTGCAGACAGACACCTGTCTCCTGTCCTCCTCTCACACCTCCTCCAACTTCCACTTAAGCCAGAGACATGGAGCTCCACTTCCAGGTGCAGCCGGCGGTGCTCCAGCTGCAGGACTACTGTTACTACTACCAGCATCAGCAGCAGGAGGCCGCCGCCGTGCAGGCCAAGCCCACCAAGCCGCGGGGCCGGAAGAAGGGCAGCACCAGCCACAGCAAGTTCGTCGGCGTCCGGCAGCGCCCGTCGGGCCGCTGGGTGGCCGAGATCAAGGACACCACGCAGAAGATCCGCATGTGGCTCGGCACCTTCGAGACCGCCGACGCCGCCGCGCGCGCCTACGACGAGGCCGCCCGCCTCCTCCGCGGCGCCGAGGCGCGCACCAACTTCGCCCCGCGCATCTCCCCGGACTGCCCGCTCGCCGTCCGCATCCGGGGCCTCCTCCACCACAAGAAGCTCAAGAAGGCCAGGCTGCCCGCCGCCTCCGCCAACATCCCCGGCCCCTCCTCCACGCCGGCCGCTCCCGCCACATACGCCCCAAGCAACAGCAACAGCAATAGCAATAGCAACAGCAACAGCAATAGCATGGATGGTGGCGCTTGTGGGGGCGCCAGCAGCAGTAGCAGCAGCAGCGCGGTCAGCTGCGACGGCGCCATGAAGCAAGGCGGCGGGGAGGTGTACAGGCCGGACTTTGCCCCCGTCGCCGGTGCCGAGGAGCTGGAGTCTTGGATGTTCGAGTCGTCGTTCGGCCAGTTCCCGGCGCTGGACGGGTTCGCCGCCGTCGATGCCTGCACGCTACCAGCCGCGTCTCCGGAGGAGACCAGCGCGTCCGGGATGGTGGAGTTCGAGCGGATGAAGGTTGAGCGGCGGATCTCGGCGTCCCTGTACGCCATGAACGGCCTGCAGGAGTACTTCGACAAGGTGTTCGAGGCGTCCGCCGGCGACCCGCTGTGGGATCTCTCGCCGCTCTGCCAGTAGCCTGGCTTCTTCGCTCTTCACCCCGTCGTCGTTCTTGCTCTTTTGCCTCCTGGGTGTTGGGGGAAATGGCCCCGGATGTATGTGTGTATGCATGTCAACTTGGTTTTTGGTGTCGCCAGGCAGTGCAGTGTAAAGAGGAAGGGGGTAGTGTTCTAGATTTCAAATATGTTCTCCATTCGATGCATTTTTGTGTGCAAAATGCTCCAAATTTGTCGTGTGGTTTAGCAGACCACTCTGAATTTCATGACGTAGCTAGTTCAGCCATGGTGTAGTTTCAGTTCTCGAGATTTGTTCAGCCATGGTGTAGTTTCAGTTCTCGAGATTTGTTCAGCCATGGTGTAGTTTCAGTTCTCGAGATTTGTTCAGCCATAGATGAGAGAACGGCATGTTTTGCTACAGTTTTCTGCTAGTGTCAATGTGGTTGTCCATCAGATGGCTGTGTTGCTTGCCGGAAACCTC

>CDS

ATGGAGCTCCACTTCCAGGTGCAGCCGGCGGTGCTCCAGCTGCAGGACTACTGTTACTACTACCAGCATCAGCAGCAGGAGGCCGCCGCCGTGCAGGCCAAGCCCACCAAGCCGCGGGGCCGGAAGAAGGGCAGCACCAGCCACAGCAAGTTCGTCGGCGTCCGGCAGCGCCCGTCGGGCCGCTGGGTGGCCGAGATCAAGGACACCACGCAGAAGATCCGCATGTGGCTCGGCACCTTCGAGACCGCCGACGCCGCCGCGCGCGCCTACGACGAGGCCGCCCGCCTCCTCCGCGGCGCCGAGGCGCGCACCAACTTCGCCCCGCGCATCTCCCCGGACTGCCCGCTCGCCGTCCGCATCCGGGGCCTCCTCCACCACAAGAAGCTCAAGAAGGCCAGGCTGCCCGCCGCCTCCGCCAACATCCCCGGCCCCTCCTCCACGCCGGCCGCTCCCGCCACATACGCCCCAAGCAACAGCAACAGCAATAGCAATAGCAACAGCAACAGCAATAGCATGGATGGTGGCGCTTGTGGGGGCGCCAGCAGCAGTAGCAGCAGCAGCGCGGTCAGCTGCGACGGCGCCATGAAGCAAGGCGGCGGGGAGGTGTACAGGCCGGACTTTGCCCCCGTCGCCGGTGCCGAGGAGCTGGAGTCTTGGATGTTCGAGTCGTCGTTCGGCCAGTTCCCGGCGCTGGACGGGTTCGCCGCCGTCGATGCCTGCACGCTACCAGCCGCGTCTCCGGAGGAGACCAGCGCGTCCGGGATGGTGGAGTTCGAGCGGATGAAGGTTGAGCGGCGGATCTCGGCGTCCCTGTACGCCATGAACGGCCTGCAGGAGTACTTCGACAAGGTGTTCGAGGCGTCCGCCGGCGACCCGCTGTGGGATCTCTCGCCGCTCTGCCAGTAG

>DNA

ATCCGTATGTAGTCCATTAGTGAAAATTCTAAAAAGACTTATATTTATAAACGGAGTGAGTAAGAGGGTATTTCTTTCTTTATGGAGGGAGTACTGTCTAAACTCCAAGTGAAATGAAATTACTCGCTGTGGTACAATTAGTATTAAACTAATTTGAGTATTGGGGTCAATTATTTAAATATAAAGGGCAAGTACACGTACCATTAACCGAACAAGTATTTTAAGAACCAAAATTCACAGAAAATACAAGAGGTCGTTTATAGTGGTGGTGCACTAACTTTGTCTTGAAAGGTGTGGTCATACACCGCGGCCACCAATCTCTCAACCTAGTGATTCCACAACAAGAGGACGTGACTAAAATGGACGTGGCTAAGGTCTTGTTTGAAACCATTCAGATTATATAATTCANNNNNNNNNATCTATTATGTCTTCAAACAGGACAAATCATAATATAGATTATAAAAAACTAGATGAACAGATTATTAAAAACTCATAATCTATTTTATCCCAGCTAAAATTAGATTATGGATTGCTAATGATCCATTACTATTGTAAAGTTGAAGATAATTACATTTCTACCACCGCCACCCTCCTCTTTAGAAAAAAACAAAGATCAGACAGGTCATTAAGCAATGTAAAATCTGGATTACAGTTTATATAATTTAGCCTCCAAGCATGTCCACCTAAATTATTTTTATAAACCAGATTATATAATCTATCTTCGTAATCCAGATTATCATAATCTATTATTGTTCCAAACAGAACCTAAGTCTAAGTCGCTTGAAAAAAAAACATTGAGTCAGTCGATTTGTTATGAGCCGTCGGATGCAAGATGAATGGCCAGATCGATTTCTTAAGCTTCCCGCCCTCTTGTTCATCCCCGAAACCTTCCCATGTACCACCGGCCCAATCGCATGCCTCCGTCGCCCGCGGCCGATGATGCGCCATCCTCGCTGCAACGGCCTTCACATTCATCGCCGCCTACCGTCGTGCACATGTGGTCTTGGCCATCCCTTTAAATTGGCGACCACAAGTTCTTTTGGATGAGCCCGCACCCGTCAATCTTCGGACTCACCTCCGCCGTCTCATTGCCGGCTGAGGCTCGTTCCTGTCCAAGGCCTTGCCGGCGTCTCCGGTCCTCGCTTTGCTCGAACTGGCGCCGTCGTCTATAGCCTCGGCGCTCCACGCGACATTCCCCCTAAAAAAAGATTGATCCTTCCCGCAAAAATAAAATAAAATACAACTGATCCAAATTTGAATCCATGCGAGTGACGTGCAGCTAAACTGAAGTAAAATACAGAAAGAGAAACCACTGCATTTGCTACTTTCCTTGCTGACAATATATGGAACAAACCAAACCGAAGAGACAAACAAACATGACATCCCAAGGCAGAGTAGAGTAACATAATAACATACATATGCCACGATCAATAACTCCACTTTGACCATGATTGACTGACCAGCCCTGAAAGTAACCCCACACGAGAGGGCAGGATCACTGCCCATACATTCCTCCTACTCCTATCTGAAAATTTCAAACCGTTCCCAAAGCCCCTATAAATCCTCCCATTTGCCAAGCAACCAGGGCAAACACTCTGCAGACAGACACCTGTCTCCTGTCCTCCTCTCACACCTCCTCCAACTTCCACTTAAGCCAGAGACATGGAGCTCCACTTCCAGGTGCAGCCGGCGGTGCTCCAGCTGCAGGACTACTGTTACTACTACCAGCATCAGCAGCAGGAGGCCGCCGCCGTGCAGGCCAAGCCCACCAAGCCGCGGGGCCGGAAGAAGGGCAGCACCAGCCACAGCAAGTTCGTCGGCGTCCGGCAGCGCCCGTCGGGCCGCTGGGTGGCCGAGATCAAGGACACCACGCAGAAGATCCGCATGTGGCTCGGCACCTTCGAGACCGCCGACGCCGCCGCGCGCGCCTACGACGAGGCCGCCCGCCTCCTCCGCGGCGCCGAGGCGCGCACCAACTTCGCCCCGCGCATCTCCCCGGACTGCCCGCTCGCCGTCCGCATCCGGGGCCTCCTCCACCACAAGAAGCTCAAGAAGGCCAGGCTGCCCGCCGCCTCCGCCAACATCCCCGGCCCCTCCTCCACGCCGGCCGCTCCCGCCACATACGCCCCAAGCAACAGCAACAGCAATAGCAATAGCAACAGCAACAGCAATAGCATGGATGGTGGCGCTTGTGGGGGCGCCAGCAGCAGTAGCAGCAGCAGCGCGGTCAGCTGCGACGGCGCCATGAAGCAAGGCGGCGGGGAGGTGTACAGGCCGGACTTTGCCCCCGTCGCCGGTGCCGAGGAGCTGGAGTCTTGGATGTTCGAGTCGTCGTTCGGCCAGTTCCCGGCGCTGGACGGGTTCGCCGCCGTCGATGCCTGCACGCTACCAGCCGCGTCTCCGGAGGAGACCAGCGCGTCCGGGATGGTGGAGTTCGAGCGGATGAAGGTTGAGCGGCGGATCTCGGCGTCCCTGTACGCCATGAACGGCCTGCAGGAGTACTTCGACAAGGTGTTCGAGGCGTCCGCCGGCGACCCGCTGTGGGATCTCTCGCCGCTCTGCCAGTAGCCTGGCTTCTTCGCTCTTCACCCCGTCGTCGTTCTTGCTCTTTTGCCTCCTGGGTGTTGGGGGAAATGGCCCCGGATGTATGTGTGTATGCATGTCAACTTGGTTTTTGGTGTCGCCAGGCAGTGCAGTGTAAAGAGGAAGGGGGTAGTGTTCTAGATTTCAAATATGTTCTCCATTCGATGCATTTTTGTGTGCAAAATGCTCCAAATTTGTCGTGTGGTTTAGCAGACCACTCTGAATTTCATGACGTAGCTAGTTCAGCCATGGTGTAGTTTCAGTTCTCGAGATTTGTTCAGCCATGGTGTAGTTTCAGTTCTCGAGATTTGTTCAGCCATGGTGTAGTTTCAGTTCTCGAGATTTGTTCAGCCATAGATGAGAGAACGGCATGTTTTGCTACAGTTTTCTGCTAGTGTCAATGTGGTTGTCCATCAGATGGCTGTGTTGCTTGCCGGAAACCTC

>HvDREB2.5

>Protein

MELQFQQQQQQRCQYEAAVGKAAAKGRGSKCKFVGVRQRPSGRWVAEIKDTTHKIRVWLGTFETAEDAARAYDEAACLLRGSNTRTNFATAAPSAAASSPPDSPLASRIRTLLTHKKLKKSASPPPRASSHSQPPALAIGPATAASNASAGSTSSTISFAMSAGGAAAAHHTPTPLSNHMTYQQWINNGGEHLHQHLEHHPWPATLSAAVPTLAARRNVGDCRVITDGTRPEKQQEDSASPGAALSGVVQEQDDGFNIGSDPCDSLWDLPPICQLSCRSLMY

>cDNA

GGGACATGCGCGCCATGACAAACCCTCCACCAACTCGCCCCGCCGGCGCCCTCCCTTAAATCCATGCGCAGCCTCCAAAAGTTCAGACCAGATACAGAGAGAGAGAATTGGTCAATCAATGGAGCTCCAGTTCCAGCAGCAGCAGCAGCAGCAATGCCAGTACGAGGCGGCGGTGGGCAAGGCGGCGGCGAAGGGGAGGGGTAGCAAGTGCAAGTTCGTCGGGGTGAGGCAGCGGCCGTCGGGGAGGTGGGTGGCGGAGATCAAGGACACCACGCACAAGATACGGGTGTGGCTCGGCACGTTCGAGACCGCCGAGGATGCCGCGCGCGCCTACGACGAGGCCGCATGCCTCCTCCGGGGCTCCAACACGCGGACCAACTTCGCCACCGCCGCCCCTTCCGCCGCCGCCTCCTCGCCGCCGGACTCGCCGCTCGCGTCCAGGATCCGCACCCTGCTCACCCACAAGAAGCTCAAGAAGAGCGCCTCGCCGCCGCCGCGGGCGTCGTCCCACTCCCAGCCGCCCGCTCTCGCCATCGGCCCCGCCACGGCAGCCAGTAATGCCAGCGCCGGCAGCACGAGCTCGACCATCAGCTTCGCCATGAGCGCCGGTGGAGCCGCCGCCGCCCACCACACTCCCACTCCCCTCTCCAACCACATGACATACCAGCAGTGGATCAACAACGGAGGCGAGCACCTCCATCAACACCTAGAGCACCACCCATGGCCTGCAACGCTCAGCGCGGCCGTCCCGACGCTCGCCGCCCGGCGCAACGTGGGCGACTGCCGGGTGATCACGGACGGTACGAGGCCGGAGAAGCAACAGGAAGACTCCGCGTCGCCCGGCGCCGCCATGAGCGGGGTAGTGCAGGAGCAGGACGACGGGTTCGACATCGGGAGCGACCCCTGCGACTCGCTGTGGGATCTGCCGCCGATCTGCCAGCTCTCCTGCAGGTCCCTCATGTACTAGACTACCGGCCGACCAAGGTTCCGCCGGCCGGCCGCGAGATCATCTTGAGGTCCATTTCCTGCCGTCGGGAGCCAATTCCATACAGGAAGATTGTATGAGGAATGATGGCCAAGCAAGCAGGGAAATGTGACGGCGGGTTGCACCTACGTGTTCGTACGTGGGTCAGGGATGAGATATGGGTGGATTTTGAGGGCACGCACGCGGTGTTGCTCTTTCTATTCTTTTTTGTCTACCAACGAATTTTCTCTCTAGGCGAAGTACGTGGAGTGGAGGTGCCGTAAAGATGTGTCACCCTTGTGCACAACAAATAATGTAATTCTTGTGGTGGATAATGATGGGGTTTTGAAGTAGTTATGGTATAGGCCGGGTGTGTGTGGCTAGAGAGACTTGGCTAGCATGTTGTTATTTTTCCGTTTTGTCTACGTTTCTCTCTTGGGTTTATGTTTTATTAACATGAGTTTAGTTTTTGTCGATGTTCTAATCGAGGATAAAGTGC

>CDS

ATGGAGCTCCAGTTCCAGCAGCAGCAGCAGCAGCAATGCCAGTACGAGGCGGCGGTGGGCAAGGCGGCGGCGAAGGGGAGGGGTAGCAAGTGCAAGTTCGTCGGGGTGAGGCAGCGGCCGTCGGGGAGGTGGGTGGCGGAGATCAAGGACACCACGCACAAGATACGGGTGTGGCTCGGCACGTTCGAGACCGCCGAGGATGCCGCGCGCGCCTACGACGAGGCCGCATGCCTCCTCCGGGGCTCCAACACGCGGACCAACTTCGCCACCGCCGCCCCTTCCGCCGCCGCCTCCTCGCCGCCGGACTCGCCGCTCGCGTCCAGGATCCGCACCCTGCTCACCCACAAGAAGCTCAAGAAGAGCGCCTCGCCGCCGCCGCGGGCGTCGTCCCACTCCCAGCCGCCCGCTCTCGCCATCGGCCCCGCCACGGCAGCCAGTAATGCCAGCGCCGGCAGCACGAGCTCGACCATCAGCTTCGCCATGAGCGCCGGTGGAGCCGCCGCCGCCCACCACACTCCCACTCCCCTCTCCAACCACATGACATACCAGCAGTGGATCAACAACGGAGGCGAGCACCTCCATCAACACCTAGAGCACCACCCATGGCCTGCAACGCTCAGCGCGGCCGTCCCGACGCTCGCCGCCCGGCGCAACGTGGGCGACTGCCGGGTGATCACGGACGGTACGAGGCCGGAGAAGCAACAGGAAGACTCCGCGTCGCCCGGCGCCGCCATGAGCGGGGTAGTGCAGGAGCAGGACGACGGGTTCGACATCGGGAGCGACCCCTGCGACTCGCTGTGGGATCTGCCGCCGATCTGCCAGCTCTCCTGCAGGTCCCTCATGTACTAG

>DNA

CCGGCGTAGCTCTCAGCCTGGCTGAGAGGGTACTTTTTGCATCAGTTGGGCCAGGCCCAGATGGCCATACGGTTTACCAAACAAGCTGAAAATTGCATCTCGGATGCCATGCAGGTGCAGCGCGGGCAACCAAACACGCCCTTAGTGTCTTGGTTGGGGTGGCAAGGTGGAGGTCTCAATCCCATGTAGGAATAGTGTCATCTCCTATTCCTGTTTCGTTGGTGCACTTAGCATTGATAGAGGTTTGTCTCATGTGGATCTTCATGTATCCGATTGGTTTCAGTCTTCCAAGGATCCATCTGGATTCGGTCAATGCTCATGGTTTTTGTAGTTTTTACTATGCCGTCAGCGTCTTTTGGTCTACTGTAACAACTTTTCGGCTGCCGCTTCTACAAGCTCATGAATTTAAACAAGTTTGATCTGTTTAGACAAAGGTTGAGAGGTGGCTACGAGCTTTGCTCACAGCGCGACGCCGGTGCATCAAGAAGGAAGAAGACTTCGGCATCCCAAAAGATTGGATGCATTTCCCATTTTATACAAAGATGTATCTCCAAGGGCCTGCTACTTAATATATGGTGTTTGACCTCACTGAAAGAAAAGTTTAAGCATGTGTTCCAAAGAAAAAAAGTTTAAGCATCGTTCTCTTTTCCTTATGCGTGCCATGTTGCACACGAAGAACCACTAATGAATCGGGCAGGCGCATATATACATGACATTGACCATCCAAATTCATATTTCAGCTCGGCGAAGACTGCAGCCCGATTGCAACTGCCAATCATGAAGACACCTGACAAATGGCGCTCCGAGGAGAAGCATCCATAAACAAAAACTCGTTGGTCATCAGTACTACTCAGTAGTGCGCGGGCTGTAACACCCTTCTTCTTCTTCATCCGCCCCACGTCGTCGATCGGCCTCGGCGAGGGCATACATGGCGGCATCGAAGCTCGATCGGGTTGACACGTACGTACGTAGCGCTCGTCGCCGGGCCTGAAAACAAAGGGCGCTGTCGTCAACCTTGGGGAGAGGACAAAGACAAACATAACATGCCAGCCACGACGTGCGACCACATCAAACATCGAGATAAGCTACGGAGTTCGGCACGTAGCAGCGCCCCTAGAAAACTCAGCCTCCCGCTAGCCCACTAACAAAGATCTCGCCGTTACGGGACATGCGCGCCATGACAAACCCTCCACCAACTCGCCCCGCCGGCGCCCTCCCTTAAATCCATGCGCAGCCTCCAAAAGTTCAGACCAGATACAGAGAGAGAGAATTGGTCAATCAATGGAGCTCCAGTTCCAGCAGCAGCAGCAGCAGCAATGCCAGTACGAGGCGGCGGTGGGCAAGGCGGCGGCGAAGGGGAGGGGTAGCAAGTGCAAGTTCGTCGGGGTGAGGCAGCGGCCGTCGGGGAGGTGGGTGGCGGAGATCAAGGACACCACGCACAAGATACGGGTGTGGCTCGGCACGTTCGAGACCGCCGAGGATGCCGCGCGCGCCTACGACGAGGCCGCATGCCTCCTCCGGGGCTCCAACACGCGGACCAACTTCGCCACCGCCGCCCCTTCCGCCGCCGCCTCCTCGCCGCCGGACTCGCCGCTCGCGTCCAGGATCCGCACCCTGCTCACCCACAAGAAGCTCAAGAAGAGCGCCTCGCCGCCGCCGCGGGCGTCGTCCCACTCCCAGCCGCCCGCTCTCGCCATCGGCCCCGCCACGGCAGCCAGTAATGCCAGCGCCGGCAGCACGAGCTCGACCATCAGCTTCGCCATGAGCGCCGGTGGAGCCGCCGCCGCCCACCACACTCCCACTCCCCTCTCCAACCACATGACATACCAGCAGTGGATCAACAACGGAGGCGAGCACCTCCATCAACACCTAGAGCACCACCCATGGCCTGCAACGCTCAGCGCGGCCGTCCCGACGCTCGCCGCCCGGCGCAACGTGGGCGACTGCCGGGTGATCACGGACGGTACGAGGCCGGAGAAGCAACAGGAAGACTCCGCGTCGCCCGGCGCCGCCATGAGCGGGGTAGTGCAGGAGCAGGACGACGGGTTCGACATCGGGAGCGACCCCTGCGACTCGCTGTGGGATCTGCCGCCGATCTGCCAGCTCTCCTGCAGGTCCCTCATGTACTAGACTACCGGCCGACCAAGGTTCCGCCGGCCGGCCGCGAGATCATCTTGAGGTCCATTTCCTGCCGTCGGGAGCCAATTCCATACAGGAAGATTGTATGAGGAATGATGGCCAAGCAAGCAGGGAAATGTGACGGCGGGTTGCACCTACGTGTTCGTACGTGGGTCAGGGATGAGATATGGGTGGATTTTGAGGGCACGCACGCGGTGTTGCTCTTTCTATTCTTTTTTGTCTACCAACGAATTTTCTCTCTAGGCGAAGTACGTGGAGTGGAGGTGCCGTAAAGATGTGTCACCCTTGTGCACAACAAATAATGTAATTCTTGTGGTGGATAATGATGGGGTTTTGAAGTAGTTATGGTATAGGCCGGGTGTGTGTGGCTAGAGAGACTTGGCTAGCATGTTGTTATTTTTCCGTTTTGTCTACGTTTCTCTCTTGGGTTTATGTTTTATTAACATGAGTTTAGTTTTTGTCGATGTTCTAATCGAGGATAAAGTGC

>HvDREB2.6

>Protein

MVPSMARKRKASDDASDADIDVGIPLSLASTDAWTTSTVAAATAARQQQRGRKRFVGVRQRPSGRWVAEIKDTIQKIRVWLGTFDTAEEAARAYDEAACLLRGANTRTNFWPRPSPPAALQPPPAMLSMQAPALPSKVSNLLLLRLKARNQQLLRDDAAAPQEAALLQQHMSSSTSSCQESYVHGRERDDEYCFQVDDFLSDECNNSPEMEEEEEEVEEEEEEEEEMDFQFMDKSAAAGDEDAGLCSPFEMVAAELGGSAPVEANDADGEPATAVQEAMRRMDYERKVSASLYALSGVSECLRMRLGGGGASAARDQLTGLREACRKKQRVAVQQPASEQSSEPAAAAGADHDAEDGKASVQEECSGSSSSGLTEAGSSPSPEAANGGSDGDVLLWSSLDLAPICYMA

>cDNA

CACACTCCAGGCCGATCGAGCCGGATCGATGGTTCCATCCATGGCGAGGAAGCGCAAGGCATCGGACGACGCCTCCGACGCCGACATCGACGTCGGCATACCTCTATCGCTAGCTTCGACGGACGCATGGACGACGTCCACCGTTGCGGCCGCGACGGCGGCGAGGCAGCAGCAGCGTGGCAGGAAGCGGTTCGTGGGCGTGCGGCAGCGGCCGTCCGGCCGGTGGGTGGCGGAGATCAAGGACACGATCCAGAAGATCCGCGTGTGGCTCGGCACCTTCGACACCGCCGAGGAGGCCGCCCGCGCCTACGACGAGGCCGCCTGCCTCCTCCGCGGCGCCAACACCCGCACCAACTTCTGGCCCCGCCCCTCGCCGCCCGCCGCTTTGCAGCCGCCGCCGGCCATGCTGTCGATGCAGGCGCCGGCGTTGCCTTCCAAGGTCTCCAACCTCCTCCTCCTCCGCCTCAAGGCCCGCAACCAGCAGCTCCTCCGCGACGACGCCGCGGCGCCGCAAGAGGCCGCGCTGCTGCAGCAGCACATGTCCTCGTCGACGTCGTCGTGCCAAGAATCGTACGTCCATGGCCGTGAGCGTGACGACGAGTACTGCTTCCAGGTGGACGACTTTTTGAGCGACGAGTGCAACAACTCGCCAGAAATGGAGGAGGAAGAAGAAGAGGTGGAAGAGGAGGAGGAGGAGGAGGAGGAGATGGACTTCCAGTTCATGGACAAGTCGGCGGCGGCGGGGGACGAGGACGCCGGGCTCTGCTCCCCGTTCGAGATGGTCGCGGCCGAGCTCGGCGGCTCGGCGCCGGTGGAGGCGAACGACGCGGACGGCGAGCCGGCGACGGCGGTGCAGGAGGCGATGAGGAGGATGGACTACGAGAGGAAGGTGTCCGCGTCGCTCTACGCGCTCAGCGGCGTGTCCGAGTGCCTCAGGATGCGCCTCGGCGGCGGCGGCGCCTCCGCCGCGCGCGATCAGCTCACCGGCCTGCGCGAGGCGTGCCGCAAGAAGCAGAGGGTGGCGGTGCAGCAGCCGGCATCGGAGCAGTCCTCGGAACCAGCCGCCGCTGCCGGTGCTGACCACGACGCGGAGGACGGCAAAGCCTCCGTGCAAGAGGAGTGTTCAGGCAGCAGCAGCAGCGGCCTCACGGAGGCGGGGAGCTCGCCGTCACCGGAGGCGGCGAACGGCGGCAGCGACGGCGACGTGCTGCTGTGGAGCTCCCTGGACCTGGCGCCCATCTGCTACATGGCATAGGCTGATCAAGTTTTGCTGTTGTGTTCCATCTTCCATGGTTGCGACTTGCAGTTAGGTTGTGTTGTGTTGGAGTGGAATTCGTTTTGTTCCATGCATGGCGGATGGCTCGTATGAGAAGCCCTGGCCATGAGACTTGCTTAATTACCAATTTATTTCGAGAAACTGTGGTACGTAAT

>CDS

ATGGTTCCATCCATGGCGAGGAAGCGCAAGGCATCGGACGACGCCTCCGACGCCGACATCGACGTCGGCATACCTCTATCGCTAGCTTCGACGGACGCATGGACGACGTCCACCGTTGCGGCCGCGACGGCGGCGAGGCAGCAGCAGCGTGGCAGGAAGCGGTTCGTGGGCGTGCGGCAGCGGCCGTCCGGCCGGTGGGTGGCGGAGATCAAGGACACGATCCAGAAGATCCGCGTGTGGCTCGGCACCTTCGACACCGCCGAGGAGGCCGCCCGCGCCTACGACGAGGCCGCCTGCCTCCTCCGCGGCGCCAACACCCGCACCAACTTCTGGCCCCGCCCCTCGCCGCCCGCCGCTTTGCAGCCGCCGCCGGCCATGCTGTCGATGCAGGCGCCGGCGTTGCCTTCCAAGGTCTCCAACCTCCTCCTCCTCCGCCTCAAGGCCCGCAACCAGCAGCTCCTCCGCGACGACGCCGCGGCGCCGCAAGAGGCCGCGCTGCTGCAGCAGCACATGTCCTCGTCGACGTCGTCGTGCCAAGAATCGTACGTCCATGGCCGTGAGCGTGACGACGAGTACTGCTTCCAGGTGGACGACTTTTTGAGCGACGAGTGCAACAACTCGCCAGAAATGGAGGAGGAAGAAGAAGAGGTGGAAGAGGAGGAGGAGGAGGAGGAGGAGATGGACTTCCAGTTCATGGACAAGTCGGCGGCGGCGGGGGACGAGGACGCCGGGCTCTGCTCCCCGTTCGAGATGGTCGCGGCCGAGCTCGGCGGCTCGGCGCCGGTGGAGGCGAACGACGCGGACGGCGAGCCGGCGACGGCGGTGCAGGAGGCGATGAGGAGGATGGACTACGAGAGGAAGGTGTCCGCGTCGCTCTACGCGCTCAGCGGCGTGTCCGAGTGCCTCAGGATGCGCCTCGGCGGCGGCGGCGCCTCCGCCGCGCGCGATCAGCTCACCGGCCTGCGCGAGGCGTGCCGCAAGAAGCAGAGGGTGGCGGTGCAGCAGCCGGCATCGGAGCAGTCCTCGGAACCAGCCGCCGCTGCCGGTGCTGACCACGACGCGGAGGACGGCAAAGCCTCCGTGCAAGAGGAGTGTTCAGGCAGCAGCAGCAGCGGCCTCACGGAGGCGGGGAGCTCGCCGTCACCGGAGGCGGCGAACGGCGGCAGCGACGGCGACGTGCTGCTGTGGAGCTCCCTGGACCTGGCGCCCATCTGCTACATGGCATAG

>DNA

ACTAATACTAATACTTCCTAACACCACCCCCCCCCCCCCCCCCCCCCAACAATCGACAGAACATGAAGTATGTAGTGTATATTTGACTGATATGAACAAATACATATATATTTGTCTCTTCCTTCATCATCGGACATATTCAAATTCTGGCTCCGCCACTAACACCTTACCACCAGAATTCATGAGTTTGAATGAAAAGTGGGGAAGGGGTCATTGCTACCGATGGCTGGCTACGTCTATACCCCGGCTAAGTAGAATGGATGATGATGCAACATTCATGACAAGGTGGTGTTAATGAACGACACGAGGAGGATGTGTAAGAGGAGTAGTACAAAAATAGCCCATGGTGGCATTTCGATCATTTTCTCGTCCGGGTTTAGACTTATTAGATCATTTCGATCACTTCCTCCCTTGCCCTATTAGACTTCAATCATTTTCTGGGGCGGATTGTGCAAAAACCTGAAAATGCTAGACAATCCCATATGACGAGAGGGACGGGAAAAAATTAATTCGGTACTACGAAATGAATTTTTAGCTCCAAAAATTATGTTGCTTCTATTTTTTTAACACTGTATGTTTTTTAAAGAATTTTGGGCTTTTGGGTCAAGCTAAAAGTTGAAGCTCAAGCTGTCATGACTTGACAACTGGGCTTTGGGCCAACCCGCTGAGCTGAGCTGCCCGTGCTTAGATCTACCCTATACAAACTTTTAGCCTTAAGGCCTTGTCTGAGACTGCTCTACTTCATAAAATTCAGAGTTGTTTGACTGAATCCACTATGAAACAGTTTTTTTTATAGAAGTTGTAACACTTTTAAAGAGAGTGTTCGCCTTTTATCTAGCTCTAGCTTCAAGAATGAGGAATTTGATGGAAATGATATATCTGATTGAATGAGTGAAAAAAACAAAGGGGTATCCACTTACTGGTGGCGGTGGTGGATAATTTTCTTCCAATTTCAGCTTGTACTTTTTTGGTACACCTCCTAAAGAGCTTCACAAAAAACATAAAGTTGTACCCTAGATTCTAGTGTTTTTTTGCGGAGTGACATTATCGTGGAGCTATCCTGTTTAGCTTGCTTTTTTTAGAGCGGAACTAAATTTGAAGGAGCGGAGTAGTCTCTAACATGCCGAGTATAGTGGTTTAATCGTCTTTCTTGCATACACATACCATTTCACACACAAGAGATTACCCGATGAGGAATCAAAGAGGTCTTGAACTCAAAACTTCTCGACTCTCATTACATATCGAGTATCCATGCAACAATGCACTCAAACAAAAAGGGTGAGCAATATGTACACTCATACTTTGAAGTCTAAAAAGGATCATGTGTACCTAGCTAGTTTTTCACTAACTAAATAATATATGTGTAGCCATATGAACCTATAAAAAAATGGCCGTATGCACCGATTGATGCAGAGGCCGGGGGTTTAAACTTTTTTTTTAAAGAAATAAAAAATGAACCTATCAATGACTAGGCCAAATACGACCACACCCACAACCTTAGCTAAGCAGATTCAAAAGACAACTTTTCACAGCATGAACACATGAATTAACTAGAGATCCCATCTCTCTCACTAGATCTCCTTGTCCCCCCATTTGTTGAACACCAAAACTAAACAAATGCAATGCAATTTTTGTACTCCCAATCAGTGATACCAGTCCGTCACCATTAACAACATAAATGCAGCAGATTGCATACAAATACTTCGTAAACTTATGCGTATGAATTAATATGAATACACAAGTGAGCTAGACTAGCACAAACACAAGCAACAAAACTGAGGCATCTTCCTCCTCTCCTCCCTTATATATACTCCTTACCCGATCATCTCTTTTCAGCCTCCCAAGTACACACTCCAGGCCGATCGAGCCGGATCGATGGTTCCATCCATGGCGAGGAAGCGCAAGGCATCGGACGACGCCTCCGACGCCGACATCGACGTCGGCATACCTCTATCGCTAGCTTCGACGGACGCATGGACGACGTCCACCGTTGCGGCCGCGACGGCGGCGAGGCAGCAGCAGCGTGGCAGGAAGCGGTTCGTGGGCGTGCGGCAGCGGCCGTCCGGCCGGTGGGTGGCGGAGATCAAGGACACGATCCAGAAGATCCGCGTGTGGCTCGGCACCTTCGACACCGCCGAGGAGGCCGCCCGCGCCTACGACGAGGCCGCCTGCCTCCTCCGCGGCGCCAACACCCGCACCAACTTCTGGCCCCGCCCCTCGCCGCCCGCCGCTTTGCAGCCGCCGCCGGCCATGCTGTCGATGCAGGCGCCGGCGTTGCCTTCCAAGGTCTCCAACCTCCTCCTCCTCCGCCTCAAGGCCCGCAACCAGCAGCTCCTCCGCGACGACGCCGCGGCGCCGCAAGAGGCCGCGCTGCTGCAGCAGCACATGTCCTCGTCGACGTCGTCGTGCCAAGAATCGTACGTCCATGGCCGTGAGCGTGACGACGAGTACTGCTTCCAGGTGGACGACTTTTTGAGCGACGAGTGCAACAACTCGCCAGAAATGGAGGAGGAAGAAGAAGAGGTGGAAGAGGAGGAGGAGGAGGAGGAGGAGATGGACTTCCAGTTCATGGACAAGTCGGCGGCGGCGGGGGACGAGGACGCCGGGCTCTGCTCCCCGTTCGAGATGGTCGCGGCCGAGCTCGGCGGCTCGGCGCCGGTGGAGGCGAACGACGCGGACGGCGAGCCGGCGACGGCGGTGCAGGAGGCGATGAGGAGGATGGACTACGAGAGGAAGGTGTCCGCGTCGCTCTACGCGCTCAGCGGCGTGTCCGAGTGCCTCAGGATGCGCCTCGGCGGCGGCGGCGCCTCCGCCGCGCGCGATCAGCTCACCGGCCTGCGCGAGGCGTGCCGCAAGAAGCAGAGGGTGGCGGTGCAGCAGCCGGCATCGGAGCAGTCCTCGGAACCAGCCGCCGCTGCCGGTGCTGACCACGACGCGGAGGACGGCAAAGCCTCCGTGCAAGAGGAGTGTTCAGGCAGCAGCAGCAGCGGCCTCACGGAGGCGGGGAGCTCGCCGTCACCGGAGGCGGCGAACGGCGGCAGCGACGGCGACGTGCTGCTGTGGAGCTCCCTGGACCTGGCGCCCATCTGCTACATGGCATAG

>HvDREB2.7

>Protein

MEEVPAPVTEEQNKRRCCPLRRSRKGCMKGKGGPENQRCPFRGVRQRTWGRWVAEIREPNRGARIWLGTFATALDAARTYDAAARALYGDCARLNLATAPLPSMTAEVPLLHEQAQQPSTHGTNTNNTNVPCCSSSNSTPSLTTRTNSSTLDSELSNYNMAGTSELEDFDEYVAGLPKPEDYGLDGFEEVMDE

>cDNA

TCTCCATCCACCTACCAAAGTCATATCAACAATGGAAGAGGTGCCGGCGCCGGTGACTGAAGAACAGAACAAGCGACGGTGTTGCCCACTCCGGCGGTCGCGCAAGGGGTGCATGAAGGGCAAGGGCGGGCCAGAAAACCAGCGGTGCCCCTTCCGCGGCGTCCGCCAGCGCACGTGGGGCCGGTGGGTGGCCGAGATCCGTGAGCCCAATCGTGGTGCCCGCATATGGCTTGGTACCTTTGCCACCGCGCTCGACGCCGCACGCACATACGACGCTGCGGCAAGGGCGCTCTATGGTGACTGCGCACGCCTCAATCTCGCAACAGCTCCACTTCCATCCATGACTGCGGAAGTTCCTCTCCTTCACGAACAAGCACAACAGCCTTCGACTCATGGTACCAACACCAATAATACCAATGTGCCATGCTGCTCCTCCTCCAACTCAACACCGTCGCTGACAACCCGCACCAACTCAAGCACCTTGGACAGTGAGTTGTCAAATTACAACATGGCAGGGACGTCGGAGTTGGAGGACTTCGATGAGTATGTGGCGGGGCTTCCCAAGCCAGAAGACTATGGCTTGGATGGGTTTGAGGAAGTGATGGACGAATAAGGCAGAGGAGGGATCAACATCTAGGACCCCACCGTCGCATCTGAGTGACATACCATGCATGGCTAGCAACCGCTTCTTCCCGCAGCAACCCAACTCGTGGTGATTATAGATCTCGCCGTCAACATTAAAAGAAGAAAGTACATGGAAAATTATATACACCGTTGGTGCTCTCTTTCTACTTAGTATGTTTTGTATATGTTGGTCGATGTAAATCTAGGATGGATCATGTTGTGTTGTTGGTCCATGAATGAAGCATGCATGCATCACCACATCTTGGCATAAATGATTCCGCATACATTCTGGCGCGCATAGTACATTGAACATATATAACACATGATCCTACATGGTAACATAGCA

>CDS

ATGGAAGAGGTGCCGGCGCCGGTGACTGAAGAACAGAACAAGCGACGGTGTTGCCCACTCCGGCGGTCGCGCAAGGGGTGCATGAAGGGCAAGGGCGGGCCAGAAAACCAGCGGTGCCCCTTCCGCGGCGTCCGCCAGCGCACGTGGGGCCGGTGGGTGGCCGAGATCCGTGAGCCCAATCGTGGTGCCCGCATATGGCTTGGTACCTTTGCCACCGCGCTCGACGCCGCACGCACATACGACGCTGCGGCAAGGGCGCTCTATGGTGACTGCGCACGCCTCAATCTCGCAACAGCTCCACTTCCATCCATGACTGCGGAAGTTCCTCTCCTTCACGAACAAGCACAACAGCCTTCGACTCATGGTACCAACACCAATAATACCAATGTGCCATGCTGCTCCTCCTCCAACTCAACACCGTCGCTGACAACCCGCACCAACTCAAGCACCTTGGACAGTGAGTTGTCAAATTACAACATGGCAGGGACGTCGGAGTTGGAGGACTTCGATGAGTATGTGGCGGGGCTTCCCAAGCCAGAAGACTATGGCTTGGATGGGTTTGAGGAAGTGATGGACGAATAA

>DNA

AGGGCATATTTCCAACAGTCATCACTTTCCACTGCCCACTTTCTATACTTGCTTGTGTAGAGTGTTTATTGACTTGAGTGAATTGCTAAAACTTGCAAGAACAAAGATTGGGAAAAGGCTAAGTTTAATTTGTTCAAGTAGTCTAATCACCCCCTCTACACCTACTTATGATCCTACAAGAGCGAGCCACATAGAGGCTAAGGGCAGGGAGAGCATGAAGTGTCATTCTGAACATCAGTGATAGAAAGGTTTAGTACAATAACACGGATCTTATCTCTAGGGTTTCAAGAGGTTTAGGCATTAAAAGCCTAATGCATTTATTTGATTATTCCAATCTTAGTTATTGAGGCAACCCCTTGAGACGATAGGGCGTTCAATTGGACAACTGACTCTTGATGCACGACGTGTGTTGGTCAAGATATAATTTGTACACATCCCCCACTTCGGTTCGTAACAAGCACATCTCGATGGGTGACTTCCGGCGCACAAATTAATATCATATTTGGTCCCAACACAAATATATGGTTGTAAGCATTAAGACTTCATACCCATGCCTATTTTTATTATAAGTGTTCTCCTTCAGTTGTTTGGTATGATCCGGTCAAAAGCTGTAATTGTTACTATAACAAAAGCGTGTATGAGACCCTAGCTTAAAGGGCATCATCTGTCGTGGCTTCAATAACTCAGGGTCACCTCTGGGGAAAAAGGCCACAATCCTTTCTGCTCCATTTCCGGATTACCAAAAGAAGTAATCAGTTTTCTTGATTTGTCATAATCACGATTAAAACTAAATAACACAGTCATCAATGCAACATTATAGTTTTTCGAAGTTTATGTGGCAGGTATTAGGAAGTTTTCAAAAGGCGGTAGTTTTTGCAATCATAGCCCCAATAATGGTAGGTTTATGCTATTTACTCTAGCTAGAGCCATGAATTAAATGTCCAAAGAGACAGGTATGTGCGTGTTGTGATGTGCCAGCCAGCCATGACATGTGTACGCAAGAGATGGAGACCAGGTGTGAGTTGGGAAGGCATGACACTTGCACACATGCACACCTACACGCGCCACTTCACCTGCACACACAGACAACACATGTAGATACCCGTCCAAATCCAAATCAATCAAAGCGACTGGCTCGCTGACCGAGGTAGGTAGCGCAGTCCCTCCAGATGCCTCGTCGTTCCCACCACACGTTGCTCCGTCCCTACACGTACGCACCTCATCTCACCATTGTCTTCCATGCCATCACCGCATCTATAAATATCAATGGTTATGGACCCTCTCCCGAGCAGAGTCAATCTCCATCCACCTACCAAAGTCATATCAACAATGGAAGAGGTGCCGGCGCCGGTGACTGAAGAACAGAACAAGCGACGGTGTTGCCCACTCCGGCGGTCGCGCAAGGGGTGCATGAAGGGCAAGGGCGGGCCAGAAAACCAGCGGTGCCCCTTCCGCGGCGTCCGCCAGCGCACGTGGGGCCGGTGGGTGGCCGAGATCCGTGAGCCCAATCGTGGTGCCCGCATATGGCTTGGTACCTTTGCCACCGCGCTCGACGCCGCACGCACATACGACGCTGCGGCAAGGGCGCTCTATGGTGACTGCGCACGCCTCAATCTCGCAACAGCTCCACTTCCATCCATGACTGCGGAAGTTCCTCTCCTTCACGAACAAGCACAACAGCCTTCGACTCATGGTACCAACACCAATAATACCAATGTGCCATGCTGCTCCTCCTCCAACTCAACACCGTCGCTGACAACCCGCACCAACTCAAGCACCTTGGACAGTGAGTTGTCAAATTACAACATGGCAGGGACGTCGGAGTTGGAGGACTTCGATGAGTATGTGGCGGGGCTTCCCAAGCCAGAAGACTATGGCTTGGATGGGTTTGAGGAAGTGATGGACGAATAAGGCAGAGGAGGGATCAACATCTAGGACCCCACCGTCGCATCTGAGTGACATACCATGCATGGCTAGCAACCGCTTCTTCCCGCAGCAACCCAACTCGTGGTGATTATAGATCTCGCCGTCAACATTAAAAGAAGAAAGTACATGGAAAATTATATACACCGTTGGTGCTCTCTTTCTACTTAGTATGTTTTGTATATGTTGGTCGATGTAAATCTAGGATGGATCATGTTGTGTTGTTGGTCCATGAATGAAGCATGCATGCATCACCACATCTTGGCATAAATGATTCCGCATACATTCTGGCGCGCATAGTACATTGAACATATATAACACATGATCCTACATGGTAACATAGCA

>HvDREB2.8

>Protein

MESMEPEVVAGMKQKKCCPLRRSRKGCMKGKGGPDNQQCPFRGVRQRTWGKWVAEIREPNRGARLWLGTFATALDAARAYDAAARALYGDCARLNLSASPSQVQVQVQVQLPPAQGSGANGNSPPGTPCCSSNNSNSNSSASTPTGTPTDVDCSNWMQPSYCYGTAEAPEDFEAYVTRLPKAEDFGLEGFQEVPLEVLAEAGGGVSIWDLSIGPDMMAAAADSSAAACTVPQQRLQQPTC

>cDNA

GAGATATATACTCCATGCCAACACACTCGCTCGTCATCAGTCCATGGAATCAATGGAGCCGGAGGTGGTTGCGGGGATGAAGCAGAAGAAGTGCTGCCCGCTCCGGCGGTCGCGCAAGGGCTGCATGAAGGGCAAGGGCGGCCCCGACAACCAGCAGTGCCCCTTCCGCGGCGTCCGCCAGCGCACCTGGGGCAAGTGGGTCGCCGAGATCCGCGAGCCCAACCGCGGCGCCCGCCTCTGGCTCGGCACCTTCGCCACCGCGCTCGACGCCGCGCGCGCCTACGACGCCGCCGCCAGGGCGCTCTACGGCGACTGCGCCCGCCTCAACCTCTCAGCGTCGCCGTCCCAGGTGCAGGTGCAGGTGCAGGTGCAGCTCCCGCCAGCTCAAGGCAGTGGAGCCAACGGTAACTCGCCGCCGGGGACGCCGTGTTGCTCCTCCAACAACTCCAACTCCAACTCCAGCGCGTCCACCCCGACCGGGACCCCCACGGACGTGGACTGCAGCAACTGGATGCAGCCGTCTTACTGTTACGGCACGGCGGAGGCGCCGGAGGACTTCGAGGCGTACGTGACGCGGCTGCCCAAGGCGGAGGACTTCGGACTGGAGGGGTTCCAGGAGGTGCCTCTGGAGGTGCTGGCGGAAGCCGGAGGAGGGGTCAGCATCTGGGACCTCTCCATCGGCCCCGACATGATGGCAGCGGCAGCCGATTCTTCAGCTGCTGCCTGCACCGTCCCGCAACAGCGGCTGCAGCAACCCACCTGCTGAGGTGCAGATCCACATAGAACGGAGTGGCGATGATACATACTCCTATAATATATACTCCGGAGTGACATGGAACGGAGTATATATAGTACATATACCGCATGCGGCATGCATGCTAGTTAACGGCGGTGGTGCATGCAGCTGGATAGCTAGTTTAGTTGAGTGTTTCCTTTTTCTCCATCGTTGCAGTAGCATACTATAGCTTTATATCGGCGGCAGGTGGGCGTGCAAGTGTGTGTGTGATATATTCGTGCATGGGAGAAGGGGTAATGAAATGATCAGTCTAGATGTTGTGCCTGGTGCTATTGGAGATATAACAGGATTAGAAGCATCTACAACCACGAGCCATATTTGGCCTAGCCCTTGGAGCATGATCTAGAGTCACACCTTCGCTTTGGCCGCTGCTTCCGTCGTCGCCTCGCGTTTCGACTACTCGATGGCGAGTCCGAGGGTCATGGAGGCGGCGGCTCCCAGCAACTCGATAGCGAGTCAAGGGTCGTGGTGGCGCGCATCAGCCTCCTTCGGTTCGGCCTAACAGGAAGGGCGCTCAAGAACGGACGCGGCAGGCTGGCAACGGTGTTCGCATTGTGTCGATGGGCCATGGGGGACGGCGTGAAGTCAGAGCTTCTTCTACCACCCTGGTCTCCTCTACGTCGCGCATCGTGTGGCACGACAATGATTCCCGCTCGTCATTGGCGCCGGATTCATCACCAGACGCTGGACGCTCGCCATAGATCCAAAAGGATGGGACGTGGAGAGGAGGAGACAGAGAGAAGTAGAGTGGATAATGAGT

>CDS

ATGGAATCAATGGAGCCGGAGGTGGTTGCGGGGATGAAGCAGAAGAAGTGCTGCCCGCTCCGGCGGTCGCGCAAGGGCTGCATGAAGGGCAAGGGCGGCCCCGACAACCAGCAGTGCCCCTTCCGCGGCGTCCGCCAGCGCACCTGGGGCAAGTGGGTCGCCGAGATCCGCGAGCCCAACCGCGGCGCCCGCCTCTGGCTCGGCACCTTCGCCACCGCGCTCGACGCCGCGCGCGCCTACGACGCCGCCGCCAGGGCGCTCTACGGCGACTGCGCCCGCCTCAACCTCTCAGCGTCGCCGTCCCAGGTGCAGGTGCAGGTGCAGGTGCAGCTCCCGCCAGCTCAAGGCAGTGGAGCCAACGGTAACTCGCCGCCGGGGACGCCGTGTTGCTCCTCCAACAACTCCAACTCCAACTCCAGCGCGTCCACCCCGACCGGGACCCCCACGGACGTGGACTGCAGCAACTGGATGCAGCCGTCTTACTGTTACGGCACGGCGGAGGCGCCGGAGGACTTCGAGGCGTACGTGACGCGGCTGCCCAAGGCGGAGGACTTCGGACTGGAGGGGTTCCAGGAGGTGCCTCTGGAGGTGCTGGCGGAAGCCGGAGGAGGGGTCAGCATCTGGGACCTCTCCATCGGCCCCGACATGATGGCAGCGGCAGCCGATTCTTCAGCTGCTGCCTGCACCGTCCCGCAACAGCGGCTGCAGCAACCCACCTGCTGA

>DNA

GGACCATGGCGGGTTGGGAATCTCGGCCTCCCGCGTTATGAATACCTCCCTCATGTTGCGGTGGGTATGGCTTATCCTCCGCAATGAGGTGGGACTATGGCTCCAACTCCTTCATGCAAAGTACCTAAGGGGTGAGCCGCTACTCGCTTGCTCCCGTGCGGGTTGATCCCAATTCTGACGCGCTATCCAAAAGATATAGGAGGAGATTTGCCTAGGAATATCCTTTAATATAGGTAATGAGGAGGGGACCCACTTCTCGTTGGACCCATGGGTTAGGGATGAGCCACTTAGGGTGTGGTTTCCTATCCTATTCTTCACCTGCATGTACCCATCCCTTCTAGTCTCTAACACCCTCCTCGAGGGGCAATGGGTTATTAGTTCACGCGGCCATTCAGATCGATAGAAACGGACGAGTGGAACCGCGTGCTTGACGTCTTGCCACAGGTGCTCCCGGATCACCCGGATACGGTGGCTTGGCATCTATCCCCTTCGCGGGAATTTTCGATTACTTCGGCATTTCAGGCCATATGTCACTCCCTCATTTTACCATGGCTCGCGTCGTTAGGGAGTGCCCCTGCCCCTAAAGATCAAAATCTTTGTGTGGCAGTTACTCCGTAACCGCATCCCTTCGGGGGCGGAGGTGCGAAAGCGGCACAGATCGGGAGACGAACGTGCCCCTTATGCGCGACACCAGATACGACAACACACATTATCCTCATGTGCATAGCGGCGAGGGCCATGTAGGAATTCATTTGTGAGGCTCTTGGACCCGACTGGGAGGTCCAGGACTTTGCGGAGTTCCTCCAATTAAGGGCCAACCACATCGGACGTCGCCGCCGCATATTCGGGTTGATCTTTACCGCAATGGCTTATACCCTTTGGACAACTCAAAATAAAATGATCTCATTTCTTTTATTTTTTTTAGGTTAGATTGTGCCGTGGGCCCGTCGGCCCGGTTTTTATGTATGATTTTACTCGTATGTGAACTTTATGAATAGTTGTTTTATCTATAAAGCAAAGCCAAAAACTATTTCAAGAAGTCTTTTGTATAAGAATTAAGAAAAACAAAGTAACCCTGTCTATGTTCATCTTGGGTCCGAGAAGCACGTGATAAATGCCACCGAAGGCATGGAGATTCAAAGGAAGAGGCATATCTGATGATTTTGCCCTGCCACAAAACACGTGGCATGAGTGGTGATCCCGACGTCATCAAGGAGAATAGCGCGCCGAGCCGCGGGCCGGAGAAGAGGACCCTTTCTGCACGCTGCGTATCCACCGAGACCGCTGCCACAGCATGAGCAAGACACGGACAGCTGTCCGTTCAGGAGCTGCGACACTTGCGTGCATGCATGCGCGAATACACGCGCCCACTGCACGTGCGAGCGCTCAACACCTAGAGCTCTTTCTTCCGATGTCCGATGGTCGCTGCCAAATCTTAGCTGCCATGGAACAAGCCAGGCCACGTAGCACGGTTCGTACGACCCCTGCACAAAAAACTAACGCCATGCCCAATTGCTTGCTCATGCCATGATGTCGTAGGGAGAGATGGATAGAGAGAACGTACGCACTGCTGCACTGCTGCACACACACACACATTGACGCATTGGATGGATATATAGTCACACGTGTTGCCACTGAAAAAAAGGAAACATGGAGAAGAAGCTCGTCCAAATCCAAACCCCCGGGCGGCTCGCTGACCGAGGCGTCGTCGCTTGAACCTGTGCGCGTGTGCCGGCGTGTAGCCGCCACGTGGGCCTGGCGCGGAGACTCGTGGAGCCCACGGCGTCGCCCATGTGTCCTGCTCGCCCGGCCCCACCCCTTCGTGTCGAGCGGCCTCCTTACTTGGCGCACGCCTGGGATACCTCGGACGCGCTCCCTACACGTCGCCTCGCCGCCGTCTTTCCCGCCCTTCGCTTTTCCACTTCACCCACCTCTATAAATACCCGCCTCCCAGTCCGAGATATATACTCCATGCCAACACACTCGCTCGTCATCAGTCCATGGAATCAATGGAGCCGGAGGTGGTTGCGGGGATGAAGCAGAAGAAGTGCTGCCCGCTCCGGCGGTCGCGCAAGGGCTGCATGAAGGGCAAGGGCGGCCCCGACAACCAGCAGTGCCCCTTCCGCGGCGTCCGCCAGCGCACCTGGGGCAAGTGGGTCGCCGAGATCCGCGAGCCCAACCGCGGCGCCCGCCTCTGGCTCGGCACCTTCGCCACCGCGCTCGACGCCGCGCGCGCCTACGACGCCGCCGCCAGGGCGCTCTACGGCGACTGCGCCCGCCTCAACCTCTCAGCGTCGCCGTCCCAGGTGCAGGTGCAGGTGCAGGTGCAGCTCCCGCCAGCTCAAGGCAGTGGAGCCAACGGTAACTCGCCGCCGGGGACGCCGTGTTGCTCCTCCAACAACTCCAACTCCAACTCCAGCGCGTCCACCCCGACCGGGACCCCCACGGACGTGGACTGCAGCAACTGGATGCAGCCGTCTTACTGTTACGGCACGGCGGAGGCGCCGGAGGACTTCGAGGCGTACGTGACGCGGCTGCCCAAGGCGGAGGACTTCGGACTGGAGGGGTTCCAGGAGGTGCCTCTGGAGGTGCTGGCGGAAGCCGGAGGAGGGGTCAGCATCTGGGACCTCTCCATCGGCCCCGACATGATGGCAGCGGCAGCCGATTCTTCAGCTGCTGCCTGCACCGTCCCGCAACAGCGGCTGCAGCAACCCACCTGCTGAGGTGCAGATCCACATAGAACGGAGTGGCGATGATACATACTCCTATAATATATACTCCGGAGTGACATGGAACGGAGTATATATAGTACATATACCGCATGCGGCATGCATGCTAGTTAACGGCGGTGGTGCATGCAGCTGGATAGCTAGTTTAGTTGAGTGTTTCCTTTTTCTCCATCGTTGCAGTAGCATACTATAGCTTTATATCGGCGGCAGGTGGGCGTGCAAGTGTGTGTGTGATATATTCGTGCATGGGAGAAGGGGTAATGAAATGATCAGTCTAGATGTTGTGCCTGGTGCTATTGGAGATATAACAGGATTAGAAGCATCTACAACCACGAGCCATATTTGGCCTAGCCCTTGGAGCATGATCTAGAGTCACACCTTCGCTTTGGCCGCTGCTTCCGTCGTCGCCTCGCGTTTCGACTACTCGATGGCGAGTCCGAGGGTCATGGAGGCGGCGGCTCCCAGCAACTCGATAGCGAGTCAAGGGTCGTGGTGGCGCGCATCAGCCTCCTTCGGTTCGGCCTAACAGGAAGGGCGCTCAAGAACGGACGCGGCAGGCTGGCAACGGTGTTCGCATTGTGTCGATGGGCCATGGGGGACGGCGTGAAGTCAGAGCTTCTTCTACCACCCTGGTCTCCTCTACGTCGCGCATCGTGTGGCACGACAATGATTCCCGCTCGTCATTGGCGCCGGATTCATCACCAGACGCTGGACGCTCGCCATAGATCCAAAAGGATGGGACGTGGAGAGGAGGAGACAGAGAGAAGTAGAGTGGATAATGAGT

>HvDREB2.9

>Protein

METGGSKREGDCPGQERTKKVRRRTTGPDSVAETIKKWKEQNQKLQQENGSRKAPAKGSKKGCMAGKGGPENSNCAYRGVRQRTWGKWVAEIREPNRGNRLWLGSFPTAVEAARAYDDAARAMYGATARVNFPEHSPDANSGCTMAPSLLTSNGATAVSHPSDGKDESESPPSLVSNAPTAALHRSDAKDEFESAGTVAHKVKTEVSNDLGSTHEEHKALEVFQPKGKALHKEANVSYDYFNVEEVVDMIIVELSADVKMEAHEEYQEGDDGFSLFSY

>cDNA

ATTCACACGAAAGTCGCTGAGTTCAGCCCTCTCCCGCTCCCTCTTTCGAAAGGCGGAAAACGAATTTTGAAACCGGCGCCCCACGGTGATAGATACCATGATCGCGGCGAACCCAGAGTGAATCGGGCCGGCGCGTCCGTCCGATTGGAGGGGCTGCCTACAAGTTACGACTTGCCTTTACCGGTCGTGGTGGCACGGGAGCGCTGTGGAGGCGGCCAGCACAGCACCGCACAGGCACCGCCCCAGTTAAAACGGAGAGGAGGAGGGGGGAGAAAGCGGGTGACGGCCGTGCCACTCGTGTTGACCCACCCCTGCCTCCTGCCCCCGCGCTCCACGTCAAAACCAAGGCGGCGGCAGCGGGGTGGGAGAGCCGGGAGCACCGACCGACACCGGGGGCTGCATGCGGAGCTGAGGCGAGGCGAGGAGAGATCCGGCGCGGGTGCCACCGCCGCCCGCCCGCGGGAGATCTGGTTGGCGGCGCCGCCGCCCGGACAAGGAAGCGGCCGCGGAGGCGGCGTGGGGCGAGCTGCCGGGGAGGCCGACGAAGCTAGAGGAGATCTCTCTCTCCCTTCCTCCCTCCTCTTCCGCCTCGATGGAGACCGGGGGTAGCAAGCGGGAAGGAGACTGCCCCGGGCAGGAAAGGACGAAGAAAGTGCGCAGGAGAACCACTGGTCCAGATTCGGTTGCTGAAACTATCAAGAAGTGGAAGGAGCAAAACCAGAAGCTCCAGCAAGAGAATGGATCCCGGAAAGCGCCCGCCAAGGGTTCCAAGAAAGGGTGCATGGCAGGGAAAGGAGGTCCAGAGAATTCAAACTGCGCTTACCGCGGTGTGAGGCAGCGCACGTGGGGCAAATGGGTGGCTGAGATCCGTGAGCCCAACCGTGGCAACCGGCTGTGGCTTGGTTCATTCCCTACCGCAGTCGAAGCTGCACGTGCATATGATGATGCCGCAAGGGCAATGTATGGCGCCACAGCGCGTGTCAACTTCCCAGAGCATTCCCCAGATGCCAACTCTGGTTGCACGATGGCACCTTCACTGCTGACGTCTAATGGGGCAACCGCTGTGTCACATCCGTCTGATGGGAAGGATGAATCAGAATCTCCTCCTTCTCTTGTCTCAAATGCGCCGACAGCTGCGCTGCATCGGTCTGATGCCAAGGATGAGTTTGAGTCTGCAGGGACTGTGGCGCATAAGGTGAAAACAGAAGTGAGCAATGATTTGGGAAGTACCCATGAGGAGCACAAGGCCCTGGAAGTATTCCAACCAAAAGGGAAGGCTTTACATAAAGAAGCGAACGTAAGTTATGATTACTTCAACGTTGAAGAAGTTGTCGACATGATAATTGTGGAATTGAGTGCTGATGTAAAAATGGAAGCACATGAAGAGTACCAAGAGGGCGATGACGGGTTTAGTCTTTTCTCATATTAGGGTTCTAGCTATGAGGGTTGTAGTCATGCGGAGCAATAGGGATTACTTCATTCTAGCTGCTAGGAAATACTTCAAATTATCTGCAACCTGAAGCTTTGTAGTCACTTATGGTTTTAATCTTACTGGAGAGAATAGCTTTATACCATAAGTCAACGGGTACAAGAAGTTGTCCTGTGTGTCGAGTTCATGTACTGTGGTAAAAATTGAGTCCATATTTAATGAGCTTACTCTGTTGATATCTTTTCTTATTTGTTTGGCGGAACTCAAGTTAGCTCTTGAAAATGGCTGGTAGAAACTCGGGGTAGCGCTTAGGGTTGGCATTAAATTGAGGCATATTAGGAAGTCATATTTCTGCAGGTGTTACTATATAGTATTGAATTATTGTAGAAAGGTCAGATTATTTATAAACTGTTAGTAACATACCACTGCTACTGCCATTCAATCATTGGAAGTAGTACATGATTGTTACGGAAATTCGCAACGATAAGTTGCATGCACTCTGCATTCAAGCTGTCTTGTTTTACTGTAAGAGGTCCTTTAGGTTGATAAAATCTAGACTGGGATATCCCAGTGCATAGTCATTCTGAGTGCTTCATGGCAGACCTTTCATCAGAAAATGTCTATGTGCACATCGCGGACAAACATGACGCCATCCTGTTTGGTGAATGGGTGGTACACATTTGAGTACCTGTCATGGAATCTGCAGGAAGCAGCAAAAGCTTGGAAGGCTATAGCGGCACTGCAACAGCCGCTACACAGCAGTGCAGCCACACAAACCTACTGATTCGTGTCCACAAAACAGAGTGGCTGAAAGCAGCCTTCCGATGGAAGACAGAGGTCGATCCACTGGCCATACAAGACGAAGATGTTCATCATCGTTAAGGAGTGAGCCCATTCCAACTATTTATCCCGAACAGGTATCACTCTGACCATTCCAACTATTTATCCTGAACAGGTATCACTCTGAAGTATATATAGTTAGATGTTGTTTCCTTCAGCAACTGAAACTTACATTTGCTACCTTTGTCCCCTATCCCTTGTCCACCATTTGCATTCTTATTTTTATGCGTCAATGGGAGTTGGG

>CDS

ATGGAGACCGGGGGTAGCAAGCGGGAAGGAGACTGCCCCGGGCAGGAAAGGACGAAGAAAGTGCGCAGGAGAACCACTGGTCCAGATTCGGTTGCTGAAACTATCAAGAAGTGGAAGGAGCAAAACCAGAAGCTCCAGCAAGAGAATGGATCCCGGAAAGCGCCCGCCAAGGGTTCCAAGAAAGGGTGCATGGCAGGGAAAGGAGGTCCAGAGAATTCAAACTGCGCTTACCGCGGTGTGAGGCAGCGCACGTGGGGCAAATGGGTGGCTGAGATCCGTGAGCCCAACCGTGGCAACCGGCTGTGGCTTGGTTCATTCCCTACCGCAGTCGAAGCTGCACGTGCATATGATGATGCCGCAAGGGCAATGTATGGCGCCACAGCGCGTGTCAACTTCCCAGAGCATTCCCCAGATGCCAACTCTGGTTGCACGATGGCACCTTCACTGCTGACGTCTAATGGGGCAACCGCTGTGTCACATCCGTCTGATGGGAAGGATGAATCAGAATCTCCTCCTTCTCTTGTCTCAAATGCGCCGACAGCTGCGCTGCATCGGTCTGATGCCAAGGATGAGTTTGAGTCTGCAGGGACTGTGGCGCATAAGGTGAAAACAGAAGTGAGCAATGATTTGGGAAGTACCCATGAGGAGCACAAGGCCCTGGAAGTATTCCAACCAAAAGGGAAGGCTTTACATAAAGAAGCGAACGTAAGTTATGATTACTTCAACGTTGAAGAAGTTGTCGACATGATAATTGTGGAATTGAGTGCTGATGTAAAAATGGAAGCACATGAAGAGTACCAAGAGGGCGATGACGGGTTTAGTCTTTTCTCATATTAG

>DNA

GTATGTTAGAGGTTAGACATGCAAAAGGAACTCTAACATTGGTTGAAGACTCAACATATACCATGTAGGTTCACTTGGAATTAAAACGCCAAACTAAGATGTTCCACGAAGTGATGTGCGCCGAGTTCATAATTTGCATCTACTTTCAACTTTGCCAAGGCTTCAAAATGACTTTTTGAGATAGCAGGATGGTGGAAATATTCCCCACTAAAAGCATAACTTATGCAAGTGCACTATGAATTGATGGGGAAATAAATTCTTAATGATGACAGTGTTTTGAACCTCTCGAGCCGAATAAGCTATTGGCGTCATTGTTTAAACTACAAATGGTTATGTTTGAAGGCCAAACATATCCATGCGACGCAAAACTGTGTGATGTAACGAAGTTCCAATACATGATGTGACGTGTTCGTGTGACATATGTGCCTACACATTAGCTGATTCTACGTGAACATCGAGAAAGGCTCAAGATCTTAAACCGATAAAACGGCCACATCTTTTAAGTATCAGTGAGGAGGCACCTAATTTCATCATGAGGTCACTTTGACGATGGTACCAAACATGCGAAGATAAATCATCCGAACACTATACCTACATACGCTTACGTCTACCACGAGATCTCACATGAGAGCCTCACGTGTCGCCCACCACGGGATCTCAGACGAGAGCCTAATTGCATATCTCTCGACAACAAAACAATCACCACCGATGCTCTTCTCCACTTTAGCACCACCTTTCTTTCGCCCGCTTCCTCACGACAAGAAGAGGGGATACAAGTATCTTTGGATGAGTCTGACACCAAGCCTGTCAAACTCGAATGCGCCAAAAATTTGAAAAAGGAATTCAAATCCTCGGTTTCACCAACACACGGGGTTTAGAAACTGAACCGATGAACGAAAAGCTGGCGAAGGTCCATACGTGCGTGGATGCTTACAGTCCATGCCAAAAAATTATTGAGGAGACACCTAATTTTATCATGATACCAACATCGCTAAGATAGGTTGTCTAACACTAAACCTATGTCACATCAGGAGATTTCAAACAAGGGCCACACGTGGCATTCATCACGAGATCTCAAACGAGGGCCTTGTGTGTCTATCTCTTGATAACAAGACAACCGTCACCGATGCTCTTCCCCATCGTCAACGTCACCTTTTCCCGTCTGTCTCCTTACGTCGGAAAGAGTATACATGTTTGTTTGGACTAATGCCCACGCCGCCCCCTTCAAACTTGGCCAATAATTTGACGAAGAAATTTAAATCCTTGGTTTCACCAAAACGCGTGATGCAAAAGGAAAAGGAACCACTACCAGAAAGTTGGCGGAGGTTCAAACGCACATCGATGGCGAAAAATTGACAGGGCAGCATTCCAAACAAACGGTGCATCTCGTTTAGTACTACCAGCAGACATTCACACGAAAGTCGCTGAGTTCAGCCCTCTCCCGCTCCCTCTTTCGAAAGGCGGAAAACGAATTTTGAAACCGGCGCCCCACGGTGATAGATACCATGATCGCGGCGAACCCAGAGTGAATCGGGCCGGCGCGTCCGTCCGATTGGAGGGGCTGCCTACAAGTTACGACTTGCCTTTACCGGTCGTGGTGGCACGGGAGCGCTGTGGAGGCGGCCAGCACAGCACCGCACAGGCACCGCCCCAGTTAAAACGGAGAGGAGGAGGGGGGAGAAAGCGGGTGACGGCCGTGCCACTCGTGTTGACCCACCCCTGCCTCCTGCCCCCGCGCTCCACGTCAAAACCAAGGCGGCGGCAGCGGGGTGGGAGAGCCGGGAGCACCGACCGACACCGGGGGCTGCATGCGGAGCTGAGGCGAGGCGAGGAGAGATCCGGCGCGGGTGCCACCGCCGCCCGCCCGCGGGAGATCTGGTTGGCGGCGCCGCCGCCCGGACAAGGAAGCGGCCGCGGAGGCGGCGTGGGGCGAGCTGCCGGGGAGGCCGACGAAGCTAGAGGAGATCTCTCTCTCCCTTCCTCCCTCCTCTTCCGCCTCGATGGAGACCGGGGGTAGCAAGCGGGAAGGAGACTGCCCCGGGCAGGAAAGGTCAGCGTCGGAACCACCCTCCTCCACCTCCGCCTCCGTCCCGCCGCGCTTCGCTCGAATCTATGGGCATTGCTGAGGCGGCGGTGGCGCGCCGTCGCCGTGGCATGGAATTTGCGCGTGTTTCCGCGCTCGGTCGCTACCGTGGATTGCCCGTCTGAAGCTAGAGCTAACGCGCGTGTGTGTCGGATTAGTTTTGGGCCGCGCTACGTCGTCCTAGGTCGGCGGCTGGCCTCCCGTTCGGTTCGCCGGGTTTCCGCCGGCTCTAGAATTATGGTTTCCTCCGCCTTCGCGGGTGTGGATTGGAGTTCGGCGAGATTTAGGGCGGCGCGCAGGGAACTGTCGGTGTTGGGTCGGGTGAGGCGGCGCCGTCCTCGGAATTGGATGCGGAAGTTCCCCATGCCCATGGCGGTGGTCGGGGATTGCTCATGCCATGGCGGTTCGATTGTTGGTGCATAGCATCATAAACTATATCTTCGGGGGCTCGATTGCAGTTGGTACCCAACCCAAGTGATAATGATCTCCTTGATTTTTTTTCCAGCAAGGAAACGAGCATAGCTCTGCTTCGTTTTGTTTGAATGCAAGGGAAAGGAGGCCCTGAGAGATTTGAGATATATATATAATATGTATGACTTTTTTTTTCTGCGAGGAAGATGTAGTATATGACTCTGATAGTGTATGTTTTTTGTTTCAATGTTTTAGCCTTGTGACATGAATTGCCTTGATGACCAGGACGAAGAAAGTGCGCAGGAGAACCACTGGTCCAGATTCGGTTGCTGAAACTATCAAGAAGTGGAAGGAGCAAAACCAGAAGCTCCAGCAAGAGAATGGATCCCGGAAAGCGCCCGCCAAGGGTTCCAAGAAAGGGTGCATGGCAGGGAAAGGAGGTCCAGAGAATTCAAACTGCGCTTACCGCGGTGTGAGGCAGCGCACGTGGGGCAAATGGGTGGCTGAGATCCGTGAGCCCAACCGTGGCAACCGGCTGTGGCTTGGTTCATTCCCTACCGCAGTCGAAGCTGCACGTGCATATGATGATGCCGCAAGGGCAATGTATGGCGCCACAGCGCGTGTCAACTTCCCAGAGCATTCCCCAGATGCCAACTCTGGTTGCACGATGGCACCTTCACTGCTGACGTCTAATGGGGCAACCGCTGTGTCACATCCGTCTGATGGGAAGGATGAATCAGAATCTCCTCCTTCTCTTGTCTCAAATGCGCCGACAGCTGCGCTGCATCGGTCTGATGCCAAGGATGAGTTTGAGTCTGCAGGGACTGTGGCGCATAAGGTGAAAACAGAAGTGAGCAATGATTTGGGAAGTACCCATGAGGAGCACAAGGCCCTGGAAGTATTCCAACCAAAAGGGAAGGCTTTACATAAAGAAGCGAACGTAAGTTATGATTACTTCAACGTTGAAGAAGTTGTCGACATGATAATTGTGGAATTGAGTGCTGATGTAAAAATGGAAGCACATGAAGAGTACCAAGAGGGCGATGACGGGTTTAGTCTTTTCTCATATTAGGGTTCTAGCTATGAGGGTTGTAGTCATGCGGAGCAATAGGGATTACTTCATTCTAGCTGCTAGGAAATACTTCAAATTATCTGCAACCTGAAGCTTTGTAGTCACTTATGGTTTTAATCTTACTGGAGAGAATAGCTTTATACCATAAGTCAACGGGTACAAGAAGTTGTCCTGTGTGTCGAGTTCATGTACTGTGGTAAAAATTGAGTCCATATTTAATGAGCTTACTCTGTTGATATCTTTTCTTATTTGTTTGGCGGAACTCAAGTTAGCTCTTGAAAATGGCTGGTAGAAACTCGGGGTAGCGCTTAGGGTTGGCATTAAATTGAGGCATATTAGGAAGTCATATTTCTGCAGGTGTTACTATATAGTATTGAATTATTGTAGAAAGGTCAGATTATTTATAAACTGTTAGTAACATACCACTGCTACTGCCATTCAATCATTGGAAGTAGTACATGATTGTTACGGAAATTCGCAACGATAAGTTGCATGCACTCTGCATTCAAGCTGTCTTGTTTTACTGTAAGAGGTCCTTTAGGTTGATAAAATCTAGACTGGGATATCCCAGTGCATAGTCATTCTGAGTGCTTCATGGCAGACCTTTCATCAGAAAATGTCTATGTGCACATCGCGGACAAACATGACGCCATCCTGTTTGGTGAATGGGTGGTACACATTTGAGTACCTGTCATGGAATCTGCAGGAAGCAGCAAAAGCTTGGAAGGCTATAGCGGCACTGCAACAGCCGCTACACAGCAGTCAGTGCCCTTGTCCTCTAGGACCATTTAAAAACAACGCCAAGACTAACTTCTTTCTTGTTGATTGTTGCAGGTGCAGCCACACAAACCTACTGATTCGTGTCCACAAAACAGAGTGGCTGAAAGCAGCCTTCCGATGGAAGACAGAGGTCGATCCACTGGCCATACAAGGTGCTTTATTTGTCACTGTCTTGCTGTTTATTTGATATTGGACACGCACTTGGAATACATGTTGACTTTGTGTTAATCTTGTTTTGTAGACGAAGATGTTCATCATCGTTAAGGAGTGAGCCCATTCCAACTATTTATCCCGAACAGGTATCACTCTGACCATTCCAACTATTTATCCTGAACAGGTATCACTCTGAAGTATATATAGTTAGATGTTGTTTCCTTCAGCAACTGAAACTTACATTTGCTACCTTTGTCCCCTATCCCTTGTCCACCATTTGCATTCTTATTTTTATGCGTCAATGGGAGTTGGG

>HvDREB2.10

>Protein

MQGKGGPENTQCGFRGVRQRTWGKWVAEIRESNRVSRLWLGTFPTAEVAAQAYDEAAKAMYGPLARTNFPVQDAQAAPTVVVQVATEGVVRGSSASCESTTTSNHSDVASSSHNKQLQIQAPEISSQSDLLESTQSVEYIQQQSVPDAVSSIAMSTSEEDVYEPLEPISNLPDGEAHCAISRCFGPRTWPETWTRRRRGCPAASSTPSSGVPAPALAPQRAEAVPSPRGGNGRRCRG

>cDNA

ATGCAAGGGAAAGGAGGCCCTGAGAATACACAATGTGGATTCCGTGGGGTAAGGCAACGAACTTGGGGGAAGTGGGTTGCTGAAATTCGGGAGTCAAATCGGGTCAGCAGGCTCTGGTTGGGGACGTTCCCTACTGCTGAAGTTGCTGCTCAAGCTTATGATGAAGCAGCCAAAGCAATGTATGGCCCGCTGGCTCGCACCAACTTCCCTGTGCAGGATGCACAAGCTGCTCCTACTGTGGTTGTACAAGTGGCAACCGAAGGTGTTGTACGTGGTTCTTCAGCATCATGCGAGTCGACTACAACATCCAACCACTCCGACGTTGCTTCTTCCTCGCATAATAAGCAACTACAAATTCAAGCTCCTGAGATTTCCTCTCAGTCAGATTTGCTGGAGTCCACCCAGTCAGTTGAGTACATCCAACAACAGTCTGTTCCTGATGCTGTCTCAAGCATTGCAATGAGCACATCTGAAGAGGATGTCTATGAGCCATTGGAGCCTATCTCCAATTTGCCAGATGGGGAAGCACACTGCGCGATCAGCAGGTGTTTCGGACCAAGGACGTGGCCTGAGACATGGACCCGCCGTCGCCGAGGGTGTCCGGCGGCATCCTCTACGCCATCTTCGGGGGTTCCGGCTCCGGCTCTGGCCCCGCAGCGCGCCGAGGCCGTGCCAAGCCCAAGAGGAGGCAACGGTCGGCGGTGTAGGGGTTGA

>CDS

ATGCAAGGGAAAGGAGGCCCTGAGAATACACAATGTGGATTCCGTGGGGTAAGGCAACGAACTTGGGGGAAGTGGGTTGCTGAAATTCGGGAGTCAAATCGGGTCAGCAGGCTCTGGTTGGGGACGTTCCCTACTGCTGAAGTTGCTGCTCAAGCTTATGATGAAGCAGCCAAAGCAATGTATGGCCCGCTGGCTCGCACCAACTTCCCTGTGCAGGATGCACAAGCTGCTCCTACTGTGGTTGTACAAGTGGCAACCGAAGGTGTTGTACGTGGTTCTTCAGCATCATGCGAGTCGACTACAACATCCAACCACTCCGACGTTGCTTCTTCCTCGCATAATAAGCAACTACAAATTCAAGCTCCTGAGATTTCCTCTCAGTCAGATTTGCTGGAGTCCACCCAGTCAGTTGAGTACATCCAACAACAGTCTGTTCCTGATGCTGTCTCAAGCATTGCAATGAGCACATCTGAAGAGGATGTCTATGAGCCATTGGAGCCTATCTCCAATTTGCCAGATGGGGAAGCACACTGCGCGATCAGCAGGTGTTTCGGACCAAGGACGTGGCCTGAGACATGGACCCGCCGTCGCCGAGGGTGTCCGGCGGCATCCTCTACGCCATCTTCGGGGGTTCCGGCTCCGGCTCTGGCCCCGCAGCGCGCCGAGGCCGTGCCAAGCCCAAGAGGAGGCAACGGTCGGCGGTGTAGGGGTTGA
>DNA

CAGCATGCTGGATGCGTCCTTGCCGAAATAGTCGAGCAGAGGTTCTTCCTCGCGGTTAGGATGTACGGAGTTTATGATACAGAGACCTATTTTGATTTTCTACTGATCTCGTTTTTTCTCCTTTCTATTTGTGAACTCTTTTGATATGGTATTTACTCCTTATTTCAACTTTCCACGGTTTTTTGTTCTATGATTTCAGGGAGTATGGACCAATGTATGGCCGAACAAGATGCGGTCGTTGTTCTTGGGGTGTTGCTGCCATTTTGCTGGACATAATCAATGCACTGTTGAGACCTCAGTCTATCCAGTGTAGGTCATACCTTCGGATCAATTGTTCACAGTTGTTGGCATATTTTTCTCCATGCTTGTTTTGTTAATCAAATTTCTGCTTTTATTTAGGTACAAGTATTTAATGAATTTACACTTCTCCATGGCGCGTCCATGAGGACAAAAAAATTATGCTCCAAATCCGCCTGGCGAGGAGTAGGTTATCTGATGATTGCTTATTATATGCTTGTATTTTCAGCTTGTTCAGGCAGGAGATGTTCTTTTGAATTTGTCAGTGATTTCGAAAAGTGGAGCTGCACTCAAGGTTCGAGATGTAGTATTTGTCGATTGGATGGTCTGTTTGACTCTTTACCCTGTTCTTTTAGATGGTTGTTTTTAAAACTCTTGCCGCCATCAGCAACACCTGAGGCTCCTCTTCCTCAGCACAAAAGTTGCAGGAAGCACAATTTGAGAGGTAAACCAAATATTTAATTTTCATTAGGAGCTAATCTGAACCATGTACAAAGTCTGCCATAGTTCAAACAAGATCTATGGACTGAAGGTTCGTTTGAGTTCCACTATTCGAGTTAACATGTACATAAATTTGCGTTTTTAACAGTTCTCGGGTGCATGCTTGGAGAAGAGGATAGGAAAAGAGGCTTGCATCTAGAGATGACAAGGGTAGTCACCTGATGCAACTAAAAGTTCAGTTTACGTAATTATTCAGGTAGTACTCCCTGGTTGGCTGGAGTATGGTGTGATGTGTTTCAAAAGGCAATACCCTTTAGCTCGTCACACCCACCGCCTCATGTTCCCCCTTTGCTGAGGTGGTGCAGGGTCGAAGCTTTGATGTTCTCCACCATGCCAGCATATCGAAATTTCCGTTATTTTGAAATATGTTAGTATGAAATATTATGTATTAATTTTTTTTATCACACACAAGTTATAATAAATTTGGATGCTTGATTTGATTCATGATTTCTATTTGCTCTCTTGCAGCAGCGACACACGCGCGCGTGTGTGTGACCGGTGAGATGCCATTTGAAAAGGTAACAAAGCACGTTCTTGGGGGTCAGGCATAATGCCTAGCAGGGACGTAGATCATACGTTTAGAAAGGTACCATAACACGTTCGTATGAAAACTACTTTGAAGCTACACAAATTTCTCTTTCGAAAGGTACCATAACAAGTTTGTTAAATATTGCAGATTATTGGAGAGACAGGCCCTGGTAGCACCGACTGTTGACTACTCTCCTGTCTGTACGTTAATGGTCGTCCCTTTCCGCTCACATATATACACACACGTACAAGAGAAGGCAAAGGCAAAGCACCAACCCCTCAAGAGGCACATCCTTGCTTGCTTGCTTGCCTCTCGTGTTCTCATCTCCATAGCTTGCAGATAAGTAAAGCTTGTCCGTTGTGCAGAGAGGGGAGGTGGATAGGTGCAGATGGAAGGGCTGCAGAGATCGTCCTCCACTTTCAGAAGGTCCGGCTCGTCGGGCCTGGTGTGGGACGAGCGGTTCCTGACCGAGGGCGCCGAGAGAATACGGTACTAACTGCTACTCTGCTATTTGTTCTACAGGAAAAAGCGACCTCGAAGGTCACGTGATGGGCCTAATTCAGTCTCTGAGACGATCAAGCAATGGAAAGAAGTGAAACAACAACTCGAGCATGATCCACAGGGTGCAAAGCGGGCGAGGAAGCCACCAGCAAAGGGTTCAAAGAAGGGCTGCATGCAAGGGAAAGGAGGCCCTGAGAATACACAATGTGGATTCCGTGGGGTAAGGCAACGAACTTGGGGGAAGTGGGTTGCTGAAATTCGGGAGTCAAATCGGGTCAGCAGGCTCTGGTTGGGGACGTTCCCTACTGCTGAAGTTGCTGCTCAAGCTTATGATGAAGCAGCCAAAGCAATGTATGGCCCGCTGGCTCGCACCAACTTCCCTGTGCAGGATGCACAAGCTGCTCCTACTGTGGTTGTACAAGTGGCAACCGAAGGTGTTGTACGTGGTTCTTCAGCATCATGCGAGTCGACTACAACATCCAACCACTCCGACGTTGCTTCTTCCTCGCATAATAAGCAACTACAAATTCAAGCTCCTGAGATTTCCTCTCAGTCAGATTTGCTGGAGTCCACCCAGTCAGTTGAGTACATCCAACAACAGTCTGTTCCTGATGCTGTCTCAAGCATTGCAATGAGCACATCTGAAGAGGATGTCTATGAGCCATTGGAGCCTATCTCCAATTTGCCAGATGGGGAAGCACACTGCGCGATCAGCAGGTGTTTCGGACCAAGGACGTGGCCTGAGACATGGACCCGCCGTCGCCGAGGGTGTCCGGCGGCATCCTCTACGCCATCTTCGGGGGTTCCGGCTCCGGCTCTGGCCCCGCAGCGCGCCGAGGCCGTGCCAAGCCCAAGAGGAGGCAACGGTCGGCGGTGTAGGGGTTGA

>HvDREB2.11

>Protein

MQGKGGPENTQCGFRGVRQRTWGKWVAEIREPNRVSRLWLGTFPTAEVAAQAYDEAARAMYGPLARTNFPVQDAQAAPAVAVPVATEGVVRGSSASCESTTTSNHSDVASSSHNKQLQIQAPEISSRSDLLESTQSVEYSQQQSVPDAVSSIAMSTSEEDVYEPLEPISNLPDGEADCFDIEELLKLIEADPVEVDPVTVGSWNEFQDAGANARGSWNEFQCGVSWNANAGMEMGQQEPLYLDGLDQGMLEGMLHSDYPFPVWISEDRPMHNPAFHDAEMSEFFEGL

>cDNA

AAGCGACCTCGGAGGTCACGTGATGGCCCTAATTCCGTCTCTGAGACGATCAAGCGATGGAAAGAAGTGAACCAACAACTTGAGCATGATCCACAGGGTGCAAAGCGGGCCAGGAAGCCACCTGCAAAGGGTTCAAAGAAGGGCTGCATGCAAGGGAAAGGAGGCCCTGAGAATACACAATGTGGATTCCGTGGGGTAAGGCAACGAACTTGGGGGAAGTGGGTTGCTGAAATTCGGGAGCCAAATCGGGTCAGCAGGCTCTGGTTGGGGACGTTCCCTACTGCTGAAGTTGCTGCTCAAGCTTATGATGAAGCAGCCAGAGCAATGTATGGCCCGCTGGCTCGTACCAACTTCCCTGTGCAGGATGCACAAGCTGCTCCTGCTGTGGCTGTACCAGTGGCAACCGAAGGTGTAGTACGTGGTTCTTCAGCATCATGCGAGTCGACTACAACATCCAACCACTCCGACGTTGCTTCTTCCTCGCATAATAAGCAACTACAAATTCAAGCTCCTGAGATTTCCTCTCGGTCAGATTTGCTGGAGTCCACCCAGTCAGTTGAGTACAGCCAACAACAGTCTGTTCCTGATGCTGTCTCAAGCATTGCAATGAGCACATCTGAAGAGGATGTTTATGAGCCATTGGAGCCTATCTCCAATTTGCCGGATGGGGAAGCAGACTGTTTTGATATTGAAGAACTATTGAAACTGATAGAAGCCGACCCAGTGGAAGTTGACCCGGTGACTGTGGGCTCCTGGAACGAATTCCAGGATGCTGGGGCCAACGCTAGGGGCTCTTGGAACGAATTCCAGTGTGGGGTCTCCTGGAACGCCAACGCTGGCATGGAGATGGGCCAACAGGAACCTCTGTACCTGGATGGCTTGGACCAAGGCATGTTGGAGGGCATGCTGCATTCTGATTATCCTTTCCCAGTGTGGATATCAGAGGATCGGCCAATGCACAACCCTGCCTTCCATGATGCTGAGATGAGCGAGTTCTTTGAGGGGTTGTGATTCCTACTGGCCGCCTTGCTCATGGCGTTTGGTCGGCTTCCCCCTCGGCGTCCGCTGCTGCGTTTCAATGAAGAAGAGGTCTTCCAATGAAGAAGAGGTGGACCGGATTGGATTCCTTTGCAGAACTAATAAGCTCCTAAGCTAGTTTTTTGTGCTTCGTTTGTAGTTCTGTTGGCATGGGGACCCTTCTCTGTTTGGTGTTTCTTTTGATAAGAAACCTTATTGTGCATCACGATCTTTGGAAGTGGAATAAGAAAAATGTGAAATGCATTGATGTCT

>CDS

ATGCAAGGGAAAGGAGGCCCTGAGAATACACAATGTGGATTCCGTGGGGTAAGGCAACGAACTTGGGGGAAGTGGGTTGCTGAAATTCGGGAGCCAAATCGGGTCAGCAGGCTCTGGTTGGGGACGTTCCCTACTGCTGAAGTTGCTGCTCAAGCTTATGATGAAGCAGCCAGAGCAATGTATGGCCCGCTGGCTCGTACCAACTTCCCTGTGCAGGATGCACAAGCTGCTCCTGCTGTGGCTGTACCAGTGGCAACCGAAGGTGTAGTACGTGGTTCTTCAGCATCATGCGAGTCGACTACAACATCCAACCACTCCGACGTTGCTTCTTCCTCGCATAATAAGCAACTACAAATTCAAGCTCCTGAGATTTCCTCTCGGTCAGATTTGCTGGAGTCCACCCAGTCAGTTGAGTACAGCCAACAACAGTCTGTTCCTGATGCTGTCTCAAGCATTGCAATGAGCACATCTGAAGAGGATGTTTATGAGCCATTGGAGCCTATCTCCAATTTGCCGGATGGGGAAGCAGACTGTTTTGATATTGAAGAACTATTGAAACTGATAGAAGCCGACCCAGTGGAAGTTGACCCGGTGACTGTGGGCTCCTGGAACGAATTCCAGGATGCTGGGGCCAACGCTAGGGGCTCTTGGAACGAATTCCAGTGTGGGGTCTCCTGGAACGCCAACGCTGGCATGGAGATGGGCCAACAGGAACCTCTGTACCTGGATGGCTTGGACCAAGGCATGTTGGAGGGCATGCTGCATTCTGATTATCCTTTCCCAGTGTGGATATCAGAGGATCGGCCAATGCACAACCCTGCCTTCCATGATGCTGAGATGAGCGAGTTCTTTGAGGGGTTGTGA

>DNA

AACTTGTGGAGCAGAGGAAAGTACCCGGAGTCATGTTCTCGTCGAACCAATAGGTATGATGCTTTGATCACTACTGTAAACAAGCATGAACTATGACTGAGATGGGTACCTCGCGATTGACTCTGAAGAAGCCAGCAGAAATAACTGTTTGTGTTTCAGCAGGAAGCAGTGATCTTCTCTGTAATGAATATGCACTCTTGGCGCAGCAAAACCCCAAGGGAGATGCGCTGCCTGTGGCATCTATTCTGCGGTGTGTTTAACCGTTCTATGTCTGGCTGCTCTATCTATTATAGAGCATGTGAGCTATGACTTGAGTTCCAGCCAACCTTTGGACCGTTTTGCCTTGTCAATATAATAGTTGGTTTTATAAGTGGCTGCTATTGAACCGAGGGCTTGCTGTTGAACTGTGAGCTTGCTGTTGGTTGATGGTATCACTTTCTGTGGATGTGGTCTCATTTAAACTGACTCTACGTGCTGTTGAACAGTTCCGTAGTGGCGAATTTTGCCATGCCCTTTCTTTATTTATTAGTCTTCAGTGTCAGGGTCGAAGTTAGGTCTCTTGTCTTGTTTGTGTTTTTGTAACTTGTTACCTAACATGTTTAGGGTTAGAAGCCGATTAGTAGTAGTTTGTCTGGAGATGGTTTGTCTATAAATGAATATTATATGCCCCTCTTGTGTGTTCATCAGTTACAGTGGAGATGGTACTGTTTATACACAGTTTGTAACAGTTCGGTGTTTGAACTAAATGTTTCTGTGTGGAGATGATCACTTTTATGTGTTCCGATTGTAGTTCTTTTGGTTGATTGTACCTTGTACCTGAATTGGGTGAGGAAAAACTAGAGAGAATATGGTACTAACTGCTACTCTGCTATTTGTTCTACAGGAAAAAGCGACCTCGGAGGTCACGTGATGGCCCTAATTCCGTCTCTGAGACGATCAAGCGATGGAAAGAAGTGAACCAACAACTTGAGCATGATCCACAGGGTGCAAAGCGGGCCAGGAAGCCACCTGCAAAGGGTTCAAAGAAGGGCTGCATGCAAGGGAAAGGAGGCCCTGAGAATACACAATGTGGATTCCGTGGGGTAAGGCAACGAACTTGGGGGAAGTGGGTTGCTGAAATTCGGGAGCCAAATCGGGTCAGCAGGCTCTGGTTGGGGACGTTCCCTACTGCTGAAGTTGCTGCTCAAGCTTATGATGAAGCAGCCAGAGCAATGTATGGCCCGCTGGCTCGTACCAACTTCCCTGTGCAGGATGCACAAGCTGCTCCTGCTGTGGCTGTACCAGTGGCAACCGAAGGTGTAGTACGTGGTTCTTCAGCATCATGCGAGTCGACTACAACATCCAACCACTCCGACGTTGCTTCTTCCTCGCATAATAAGCAACTACAAATTCAAGCTCCTGAGATTTCCTCTCGGTCAGATTTGCTGGAGTCCACCCAGTCAGTTGAGTACAGCCAACAACAGTCTGTTCCTGATGCTGTCTCAAGCATTGCAATGAGCACATCTGAAGAGGATGTTTATGAGCCATTGGAGCCTATCTCCAATTTGCCGGATGGGGAAGCAGACTGTTTTGATATTGAAGAACTATTGAAACTGATAGAAGCCGACCCAGTGGAAGTTGACCCGGTGACTGTGGGCTCCTGGAACGAATTCCAGGATGCTGGGGCCAACGCTAGGGGCTCTTGGAACGAATTCCAGTGTGGGGTCTCCTGGAACGCCAACGCTGGCATGGAGATGGGCCAACAGGAACCTCTGTACCTGGATGGCTTGGACCAAGGCATGTTGGAGGGCATGCTGCATTCTGATTATCCTTTCCCAGTGTGGATATCAGAGGATCGGCCAATGCACAACCCTGCCTTCCATGATGCTGAGATGAGCGAGTTCTTTGAGGGGTTGTGATTCCTACTGGCCGCCTTGCTCATGGCGTTTGGTCGGCTTCCCCCTCGGCGTCCGCTGCTGCGTTTCAATGAAGAAGAGGTCTTCCAATGAAGAAGAGGTGGACCGGATTGGATTCCTTTGCAGAACTAATAAGCTCCTAAGCTAGTTTTTTGTGCTTCGTTTGTAGTTCTGTTGGCATGGGGACCCTTCTCTGTTTGGTGTTTCTTTTGATAAGAAACCTTATTGTGCATCACGATCTTTGGAAGTGGAATAAGAAAAATGTGAAATGCATTGATGTCTCCAATTTTTTTATTTTTTTGCTGCCTTAGTAAATTCCTGTCTGATTATAATCTACCAACTTAACGTCGTTAATACTGTTTAGCATCCTCTGTTGAGTATTTTGAAGACCTTTCAGGTCAAAGCCCCAAATTTTGTATACAAACCGTGCCGTACAATGTCGTAACTTAAAAACAAGGACTACACGTGATAAGTCCTGATACCAACTGAACTTCTCAAAAAAAGAAAAAACAACAACGGCTGATATTTCCATGGTCAGCAAATATAAATTCGTATTCAGGAC

>HvDREB4.1

>Protein

MESDTIHAPATSSSSSSHSSGSTVINGVQDVSAKHLKRKRQTATSPAPGTGSQGTSEAADGARGEESSSCAAAADERTAGRHPSYRGVRRRSWGKWVSEIREPRKKSRIWLGTFPTAEMAARAHDVAAVAIKGRRAHLNFPHLAHALPRPASTSPADIQAAAALAAAQCESSSSVAETTETSTTSTEKENSTSAASSGEAGSGEENALFDLPDLLLDLRDGLWCSPTWASAADDYDGGEAAMHEPLLWVEQCWT

>cDNA

CGTCCATGTAAAAGAAGACAATTATCATTTGGCCGGGCTGCATATATGCTGCTCATACAACTGGAGTTGTGAACTGGCCAGTATTTGGACGGTCGCTGTTTGGTTATGTCTACTACCCAACTATATATCGTTGCCAATGCAGGCCATCCATGTGTTGGAGGCCACACCAGCTTGCAAATATCTAACTAGCCGCAGAATTCCCCACGTATTTGTTAATTCCGGAGATCCTAACAACAACATCAGCTTTATTTCTTTTCTAGCTCCGCATGGTGGCGATATCTTTTGTTTATGCAGAGAAGGAAAATAAACGTAGGCCTCCACTAATCAATCCACTATTTATAGCCGCGCGCACCACCTTCCCTCCCCAGCCTTGAGCCTTGTCTGGTGCTTGTAGTTGTAGAAAGGAAGGACTCGAGCCATGGAGTCTGACACGATCCATGCACCCGCCACCTCCTCTTCCTCCTCCTCCCACTCTTCCGGCTCGACTGTCATCAATGGCGTACAGGATGTGTCCGCCAAGCATCTGAAGCGCAAGAGACAGACCGCCACCTCTCCTGCTCCCGGCACCGGAAGCCAAGGGACGTCGGAGGCCGCCGACGGCGCCCGGGGGGAGGAGAGCAGCAGCTGCGCCGCCGCGGCGGACGAGCGCACGGCCGGTAGGCACCCGTCGTACCGCGGCGTGCGGCGCCGGAGCTGGGGCAAGTGGGTGTCGGAGATCCGAGAGCCGCGCAAGAAGTCGCGCATCTGGCTCGGCACGTTCCCGACCGCCGAGATGGCCGCGCGCGCGCACGACGTGGCCGCGGTCGCCATCAAGGGCCGCCGCGCGCACCTCAACTTCCCGCACCTCGCCCACGCGCTCCCCCGTCCGGCTTCCACCTCGCCCGCCGACATCCAGGCCGCGGCCGCCTTGGCCGCCGCCCAATGCGAGTCTTCCTCCTCCGTCGCCGAGACCACGGAGACGTCGACGACATCGACGGAGAAGGAGAACTCGACATCGGCCGCGAGCTCCGGTGAGGCCGGCAGCGGCGAGGAGAACGCTCTGTTCGACCTGCCCGACCTTCTGCTGGACCTGAGGGACGGCCTCTGGTGCTCGCCGACCTGGGCGTCGGCCGCCGACGACTACGACGGCGGCGAAGCCGCCATGCACGAGCCGCTCCTGTGGGTGGAGCAGTGCTGGACCTAGCCGCTCCGTTCGAACGCGCGGACCATGACTGGCCAGAAGAGAACACCGGTGGACACTGGCATGTAGCCATGTACTAGCAGTATTTCATTTTTCCAATTAGGTTAAGCTTTGCTTCGCGCCATGCCTCGGTTGCCATCCTGGGGTGGCAAGTCGTTCTAGCTAGACGTTTCACCGTTTTCCCCACGGTGTGTGTTGTATCCGGAATGTGAAATACAATACCCTCACAAAAGAAAAAGAAAAAAAGATGAAATACAATTTACCTCTTCAGCCTCAAAATCTCAA

>CDS

ATGGAGTCTGACACGATCCATGCACCCGCCACCTCCTCTTCCTCCTCCTCCCACTCTTCCGGCTCGACTGTCATCAATGGCGTACAGGATGTGTCCGCCAAGCATCTGAAGCGCAAGAGACAGACCGCCACCTCTCCTGCTCCCGGCACCGGAAGCCAAGGGACGTCGGAGGCCGCCGACGGCGCCCGGGGGGAGGAGAGCAGCAGCTGCGCCGCCGCGGCGGACGAGCGCACGGCCGGTAGGCACCCGTCGTACCGCGGCGTGCGGCGCCGGAGCTGGGGCAAGTGGGTGTCGGAGATCCGAGAGCCGCGCAAGAAGTCGCGCATCTGGCTCGGCACGTTCCCGACCGCCGAGATGGCCGCGCGCGCGCACGACGTGGCCGCGGTCGCCATCAAGGGCCGCCGCGCGCACCTCAACTTCCCGCACCTCGCCCACGCGCTCCCCCGTCCGGCTTCCACCTCGCCCGCCGACATCCAGGCCGCGGCCGCCTTGGCCGCCGCCCAATGCGAGTCTTCCTCCTCCGTCGCCGAGACCACGGAGACGTCGACGACATCGACGGAGAAGGAGAACTCGACATCGGCCGCGAGCTCCGGTGAGGCCGGCAGCGGCGAGGAGAACGCTCTGTTCGACCTGCCCGACCTTCTGCTGGACCTGAGGGACGGCCTCTGGTGCTCGCCGACCTGGGCGTCGGCCGCCGACGACTACGACGGCGGCGAAGCCGCCATGCACGAGCCGCTCCTGTGGGTGGAGCAGTGCTGGACCTAG

>DNA

TTTTACCCACACAAGTGTCAAATGCACACATTTTTACATTTGGTTCAAGCACTCAACACTTACATGCACATAGTATCTAGGTGTATCCTTCTAATGACGCCAATTCATGCTTTTGCAGCCTGATACAGTAACAGGAGACATAGTTGTTGTCTTGCAACTTAAAGAGCACCCAAAATTCAAGAGGAAGTCGGATGACCTGTTCGTTGAGCATGCGATCTCTCTGACTGAGGCTCTCTGTGGCTTCCAGTTTGTTCTGACCCATCTTGATGGTCGACAGCTTCTGATCAAATCCAATCCTGGGGAGATCATAAAGCCTGGTACATGAATTTCCTCTTGTATTTCTGTTTTTCCTTGTTCCTTCCGAGTTCCTCTATCCTGCCGCGATAGAGTTTCCCCCTGTTTGGAACATAGTGATATAATCTTTTGTTCTGGAAAACAGGTCAGCACAAGGCCATAAATGACGAGGGCATGCCTCGGCATGGCCGGCCTTTCATGAAGGGTCGCCTGTTCGTCGAGTTCAGCGTGGAGTTCCCTGAGCCTGGAGTACTGACCCCTAGCCAATGCAAGTCACTTGAGAAGATCCTGCCGCCAAGACCAGGGAGCCAGTCGTCGGACATGGACGTGGATCAGTGCGAGGAAACGACCATGCACGACGTGAACATAGAAGAGGAGATGAGACGCAGGCAGCATCAAAGGCGGCAAGAAGCATACGATGAAGAAGATGAGGATGAAGGCGGTGCCCCAAGGGGGGTGCAGTGCGCCCAGCAGTAGGAAACAAGGATGTCGATCAACTACATTCTACTATTGCTCCTTTCCCGTTTAATTAAAGTACTCCTAGCTGATCTGATGCGCGGTATTATTATTGTTGGCGTCCTAAGATTATGAGGTTGTTAGCCATAAGTATCATTCATCCTTTTGTTGGGCCTATTGCCTGCTGTGTCGTGTACCGGTATTATTTTGGAGCCCATACTTTGATATTTCAGACTTGGTTGGGTGAATTCGTCTTTGTGTGTGCTGGTGCTGCATTATTATTATTATTATATTTGTAATCTTCGTGCTGCTCAGCAACTTGAGGCCTTGCTACTATTTGTATCAGATCTGTCAGTGAGGGTCATTAGTATGAGTGGAACGTGTTGATGGAATTTACTCTTACCAATGCCATCCTCGATGGCATCTCGGCACCAATAGTGAAGCGAAGACTTTGCCGGTTGAAGCAGATGCAATCGCCAGGCGACGCGCCACCGTTCCTTCCAGGCTCTTGCTTGAACCACGGCCGGTGTGGCACCGCATTGCCGTTCGCACATCGTTGTTAACGAAGTCCTTGAGTTGTACCAGCAAGCAGTCCGGTCCAGCACAAGTTGGCGCACGAAATAGTAACCACCTTCCTGCAGGAAGGAAGGTGCTGGCTGGTTAAACCAAGCGGCCTGATTGATTGAAGGGGTCTTGTTCTCAGTGACATCTACTTAACGGCTAGTTGTTGAGGTGTAGTCTTCGTCTCTGAGGACATCAGCCTCTCCTCCAAGCTCGCGTGACCGGGCCGGTCGTCCATGTAAAAGAAGACAATTATCATTTGGCCGGGCTGCATATATGCTGCTCATACAACTGGAGTTGTGAACTGGCCAGTATTTGGACGGTCGCTGTTTGGTTATGTCTACTACCCAACTATATATCGTTGCCAATGCAGGCCATCCATGTGTTGGAGGCCACACCAGCTTGCAAATATCTAACTAGCCGCAGAATTCCCCACGTATTTGTTAATTCCGGAGATCCTAACAACAACATCAGCTTTATTTCTTTTCTAGCTCCGCATGGTGGCGATATCTTTTGTTTATGCAGAGAAGGAAAATAAACGTAGGCCTCCACTAATCAATCCACTATTTATAGCCGCGCGCACCACCTTCCCTCCCCAGCCTTGAGCCTTGTCTGGTGCTTGTAGTTGTAGAAAGGAAGGACTCGAGCCATGGAGTCTGACACGATCCATGCACCCGCCACCTCCTCTTCCTCCTCCTCCCACTCTTCCGGCTCGACTGTCATCAATGGCGTACAGGATGTGTCCGCCAAGCATCTGAAGCGCAAGAGACAGACCGCCACCTCTCCTGCTCCCGGCACCGGAAGCCAAGGGACGTCGGAGGCCGCCGACGGCGCCCGGGGGGAGGAGAGCAGCAGCTGCGCCGCCGCGGCGGACGAGCGCACGGCCGGTAGGCACCCGTCGTACCGCGGCGTGCGGCGCCGGAGCTGGGGCAAGTGGGTGTCGGAGATCCGAGAGCCGCGCAAGAAGTCGCGCATCTGGCTCGGCACGTTCCCGACCGCCGAGATGGCCGCGCGCGCGCACGACGTGGCCGCGGTCGCCATCAAGGGCCGCCGCGCGCACCTCAACTTCCCGCACCTCGCCCACGCGCTCCCCCGTCCGGCTTCCACCTCGCCCGCCGACATCCAGGCCGCGGCCGCCTTGGCCGCCGCCCAATGCGAGTCTTCCTCCTCCGTCGCCGAGACCACGGAGACGTCGACGACATCGACGGAGAAGGAGAACTCGACATCGGCCGCGAGCTCCGGTGAGGCCGGCAGCGGCGAGGAGAACGCTCTGTTCGACCTGCCCGACCTTCTGCTGGACCTGAGGGACGGCCTCTGGTGCTCGCCGACCTGGGCGTCGGCCGCCGACGACTACGACGGCGGCGAAGCCGCCATGCACGAGCCGCTCCTGTGGGTGGAGCAGTGCTGGACCTAGCCGCTCCGTTCGAACGCGCGGACCATGACTGGCCAGAAGAGAACACCGGTGGACACTGGCATGTAGCCATGTACTAGCAGTATTTCATTTTTCCAATTAGGTTAAGCTTTGCTTCGCGCCATGCCTCGGTTGCCATCCTGGGGTGGCAAGTCGTTCTAGCTAGACGTTTCACCGTTTTCCCCACGGTGTGTGTTGTATCCGGAATGTGAAATACAATACCCTCACAAAAGAAAAAGAAAAAAAGATGAAATACAATTTACCTCTTCAGCCTCAAAATCTCAA

>HvDREB4.2

>Protein

MEADASHAPTTSSSSVSSSTLSTSSSSCSLANSAQEPPKNPKPKHPKKRKRAAADQETDAAATNGARGDESSCCSTDEDNAASVKAAVSKSGFKHPSYRGVRRRSWGKWVSEIREPRKKSRIWLGTFPTAEMAARAHDVAALAIKGRSAHLNFPDLAHELPRPDSTSPADIQAAAAKAAATAAVQCEPEPEHEHETPSSSGAVSESPPEPAAACPEAAVPADSGEVDNALFDLPDLLLDLRDGLFWSPVWPVALAAEEYDGGCCVGLSEPLLWAE

>cDNA

GAAGTCCCCACGCTTCTCGATCGTAGTCCACTTAGCTACAGGCCTTCAGATCCCTGCTACCGAGCCATGGAGGCCGACGCTAGCCATGCACCCACCACCTCCTCCTCCTCCGTCTCCTCGTCGACATTGTCCACTTCCTCCTCCTCCTGCTCCCTCGCCAACAGCGCGCAGGAGCCTCCCAAGAACCCCAAGCCCAAGCACCCGAAGAAGCGCAAGAGAGCCGCCGCCGACCAAGAAACGGACGCCGCGGCTACCAATGGCGCCCGTGGGGACGAGAGCAGCTGCTGCAGCACCGACGAGGACAACGCCGCGAGCGTCAAGGCGGCCGTGTCCAAGAGCGGCTTCAAGCACCCGTCGTACCGCGGCGTGCGGCGCCGGAGCTGGGGCAAGTGGGTGTCCGAGATCCGCGAGCCGCGCAAGAAGTCCCGCATCTGGCTCGGCACCTTCCCCACCGCGGAGATGGCGGCGCGCGCCCACGACGTGGCCGCGCTCGCCATCAAGGGCCGCTCCGCGCACCTCAACTTCCCGGACCTCGCCCACGAGCTGCCCCGCCCGGACTCCACGTCGCCCGCCGACATCCAGGCCGCCGCCGCCAAGGCCGCCGCCACCGCCGCCGTGCAGTGCGAGCCCGAGCCCGAGCACGAGCACGAGACGCCGTCGTCCTCCGGCGCCGTTTCGGAGTCACCGCCGGAGCCTGCGGCAGCCTGCCCCGAAGCAGCGGTGCCAGCGGACAGCGGCGAGGTCGACAATGCGCTGTTCGACCTGCCCGACCTTCTTCTCGACCTGAGGGACGGGCTCTTCTGGTCGCCGGTCTGGCCGGTGGCGCTGGCCGCCGAGGAGTACGACGGCGGCTGCTGCGTCGGGCTCAGCGAGCCACTCCTGTGGGCCGAGTAGGGGTATATTTTGTTTTTCGCCGCATGAATTGCTTTGCCACTTGCCAGTAGTAGTACTATAGTAGTATCGTACGTAGCAAGGATAAACCATTTAGTGGTAGCACTGTCATTGGAAAAAGTTATTATTCTCAGCTTTTTGTGAGTCTTCTTCACTCGGCC

>CDS

ATGGAGGCCGACGCTAGCCATGCACCCACCACCTCCTCCTCCTCCGTCTCCTCGTCGACATTGTCCACTTCCTCCTCCTCCTGCTCCCTCGCCAACAGCGCGCAGGAGCCTCCCAAGAACCCCAAGCCCAAGCACCCGAAGAAGCGCAAGAGAGCCGCCGCCGACCAAGAAACGGACGCCGCGGCTACCAATGGCGCCCGTGGGGACGAGAGCAGCTGCTGCAGCACCGACGAGGACAACGCCGCGAGCGTCAAGGCGGCCGTGTCCAAGAGCGGCTTCAAGCACCCGTCGTACCGCGGCGTGCGGCGCCGGAGCTGGGGCAAGTGGGTGTCCGAGATCCGCGAGCCGCGCAAGAAGTCCCGCATCTGGCTCGGCACCTTCCCCACCGCGGAGATGGCGGCGCGCGCCCACGACGTGGCCGCGCTCGCCATCAAGGGCCGCTCCGCGCACCTCAACTTCCCGGACCTCGCCCACGAGCTGCCCCGCCCGGACTCCACGTCGCCCGCCGACATCCAGGCCGCCGCCGCCAAGGCCGCCGCCACCGCCGCCGTGCAGTGCGAGCCCGAGCCCGAGCACGAGCACGAGACGCCGTCGTCCTCCGGCGCCGTTTCGGAGTCACCGCCGGAGCCTGCGGCAGCCTGCCCCGAAGCAGCGGTGCCAGCGGACAGCGGCGAGGTCGACAATGCGCTGTTCGACCTGCCCGACCTTCTTCTCGACCTGAGGGACGGGCTCTTCTGGTCGCCGGTCTGGCCGGTGGCGCTGGCCGCCGAGGAGTACGACGGCGGCTGCTGCGTCGGGCTCAGCGAGCCACTCCTGTGGGCCGAGTAG

>DNA

GAAGTCCCCACGCTTCTCGATCGTAGTCCACTTAGCTACAGGCCTTCAGATCCCTGCTACCGAGCCATGGAGGCCGACGCTAGCCATGCACCCACCACCTCCTCCTCCTCCGTCTCCTCGTCGACATTGTCCACTTCCTCCTCCTCCTGCTCCCTCGCCAACAGCGCGCAGGAGCCTCCCAAGAACCCCAAGCCCAAGCACCCGAAGAAGCGCAAGAGAGCCGCCGCCGACCAAGAAACGGACGCCGCGGCTACCAATGGCGCCCGTGGGGACGAGAGCAGCTGCTGCAGCACCGACGAGGACAACGCCGCGAGCGTCAAGGCGGCCGTGTCCAAGAGCGGCTTCAAGCACCCGTCGTACCGCGGCGTGCGGCGCCGGAGCTGGGGCAAGTGGGTGTCCGAGATCCGCGAGCCGCGCAAGAAGTCCCGCATCTGGCTCGGTACCTTCCCCACCGCGGAGATGGCGGCGCGCGCCCACGACGTGGCCGCGCTCGCCATCAAGGGCCGCTCCGCGCACCTCAACTTCCCGGACCTCGCCCACGAGCTGCCCCGCCCGGACTCCACGTCGCCCGCCGACATCCAGGCCGCCGCCGCCAAGGCCGCCGCCACCGCCGCCGTGCAGTGCGAGCCCGAGCCCGAGCACGAGCACGAGACGCCGTCGTCCTCCGGCGCCGTTTCGGAGTCACCGCCGGAGCCTGCGGCAGCCTGCCCCGAAGCAGCGGTGCCAGCGGACAGCGGCGAGGTCGACAATGCGCTGTTCGACCTGCCCGACCTTCTTCTCGACCTGAGGGACGGGCTCTTCTGGTCGCCGGTCTGGCCGGTGGCGCTGGCCGCCGAGGAGTACGACGGCGGCTGCTGCGTCGGGCTCAGCGAGCCACTCCTGTGGGCCGAGTAGGAGTATATTTTGTTTTTCGCCGCATGAATTGCTTTGCTACTTGCCAGTAGTAGTACTATAGTAGTATCGTACGTAGCAAGGATAAACCATTTAGTGGTAGCACTGTCATTGGAAAAGTTATTATTCTCAGCTTTCTGTGAGTCTTCTTCACTCGGCCATGAAACATGTGTGCTTTTTTATTTGTATTGTGCTATCAACAAAAAGATGCTTTGCTAGACACGATGGACTTCCCTCAGAAAGAAGAGGACACGGGTTTGCTCTACAAGTTTGCAAATGCTTTAGATGATTGCTCAGTTGATAGATACTTCTACTGTTTTACATCTACATGGTGGGCATTAACTCGATGCGTTCGCAATTATTTTGTTATTTTGATTGCCGGCCGTTTGTAATTACCTCGTTTTTATGGTATACGGTGAAACAGTGCTATTACACAAGCAAAAAAAGCAAGCCAGAATTAATGAACCGCGCAGTGTTTCCTTACCGATTGCACAAACGAGTTTGGCACCAAGGAATGGACTCTTGTGTTGGCGTGTTATACTGCCCCGCTCTCCAACAGAAGTGCAGTTTTAACAAACAAAACCCCAAATTTCCACAGAGTTCTGACCTTGCTGCTGCTTCGTCGTCGTTATGAGAGGATCTCTAGAAACACGGAGGGGAAAATCTTGTAGTAATATAACAATGCCAACATGGCTTTTAATTACCTGCGGCTTAGTATAGTTAACTAGTCTGTAGGTGAGGTAGTGAGCTGTAGTCATATCTTTGCTGGGTTTGGTACAATTCCCACGTTATCCTAGGTTACAATAAAGGGAGTCGATCGAGTCCAGAGGCGGAAAGACACGGCCGGAGCTAGGACGAAAAAAAGAAACAAGCAAAGCAGACGGAAGGATGAGAGAAAGTTCCAGCCCTGACCGCGCAATATTCCCTTGTCTCGTAGCAGCAGAGCTTGTTAAAAACGCAAAGCCAATGACCACCTGGTGTAGGTTAGGTACACACCACAAGCACGCCAGTTACCGGCTCCCATATGATACGAGTCCAGCTGCAAGCCGTATGTACTCCACCGCCAGCAAAGCAGCAACGCCAAGTTTGATAAGTTTTAATTAAAATCAGTCTGCTTGTTCTTTTCAGGTATAAGAAACTCCAGGTTTAGTCACCTCCACATGTAATCATCGATAAATTAGTAGGGAAAAACCAAGGATCTTGATGAGTACGCACGTACACGTAGCGGAGGCCATGAGTGGCCGCGCGCGTACGTCGTCGACCGCCTCACATGCCCAACACATGGCCAGGCGATCATGCACTGCATATGGTTGCGTACATACATACATGCCGCGACGTAGTACGACGCATGCAGGCAAGCAGGCAGGCGGCATCGCCAGTGTTAGCATAACAAGCCGGGCTGGCTGATTGCGTCCTGACTGATTAACAATAACAAACGCGTGCGTTCGTATCGTACGTACGTGATCACCGGCATTAACCAATGGAGATGGAGATCGGGGGTGTCGCTGATTCGCGGGGTGCATGCCGACGGGAGGATCCGATGCCGCCGTCCGTGCGGTGCTCCGGCAGCTGGGGCCGGTCGACGGCTGCCCCGGCCGGCCGGGCGGGCGGCGGCTCCGGTGGGGGCGGTAGGTAGGGGTAGGGGTGGTACCGGGCTGGAGGCTCGTACTACGTACGTTTGCCGCTTCAATTGCTGGGATGGGGATATCTGTGCGTGGATTCGGACTGGGCAATGAGGCACCTAACCGGTTAGTTAGCACTCAACAGAATGTTGGTGCAACCGGCCGATCCATCGACACAGTCGGGGTCTAAGCATTTAATGCTTTGATTTTATGATGCGTGCGCGCCTTAAGTAATATAAGTATGGACGGGTATGTACCATATCGTGACTGATTTGATGCCACGGGATGATACTACGGTCCAAGGAAGCTACTGTGCTACTGTTGTTGTTGTGGGAGTCGTCAGTTAACACAAAGTAAACAGTATAGGAACACACACAAACAGGACTAGTCTCAAACTATGGCAAAGAAATAGATCATGGTTAATCCGGAGATTTCTATGACGATTTTATCAGACATGAATCGCGACTGGGCAATGCTTTTTTCTGACCAACCAAAAGTGAAAAGGTTTACCTCTGACCAGCAAAATGAAGGCCAGGCTGAGGGTCCTATGAGAAGGGCGATCTATAGACGACGGCCACCCATAATTGGACAAAAGTCATTTCTTATTAGATTCAAACACCCACGAATAATTGTAACCGGGCACTACCACACAATGGAGATGACGATCGCTGATCGTCCTTGCTCCTGCATGTGACAAGGATGCCTAGCTCGCGCCCATCAAGGTGTTAGTTTTCACTCCAAACGCATGTGGCCACTGCT

>HvDREB4.3

>Protein

MGATEDSSSGSETTTSSSVEALASPPSPTATTASSKKKRARNDGRHPTYRGVRMRSWGKWVSEIREPRKKSRIWLGTFATAEMAARAHDVAALAIKGRAAHLNFPDLAHELPRPATAAPKDVQAAAALAASADFPASATATANAGAKNPDGPEPNAASASTPPDTAEDALFDLPDLLFDLRHGPPSCQVSCASTWDDDVAFAGPGAGVFRLEEPLQWEY

>cDNA

GGCCAACACGCTGGCCTCACGCCTTCACCATTCGATTCCCCGCTTTGTTTTTGCTTTACACGTTGCTCCCTCCGCCTCATCTGCTCATCATAAAAACTCCCACTTCCTTCCCTCGTAACGCACCACTCACCTCACCTCCTCACGCTCTCGCTCGCTCATCACTCCCCAACTACTAACTCCACCACCAGCAGCAGGCAGCAGCTAGCGCGCGGCAGCCATGGGCGCCACGGAGGATTCCTCCTCCGGCTCCGAGACGACCACCTCGTCCTCGGTGGAGGCCCTGGCCTCGCCGCCGTCCCCGACGGCCACCACCGCGTCGTCCAAGAAGAAGCGGGCGCGCAACGACGGGCGGCACCCGACGTACCGCGGGGTGCGCATGCGGAGCTGGGGCAAGTGGGTGTCGGAGATTCGGGAGCCCCGCAAGAAGTCGCGCATCTGGCTCGGCACCTTCGCCACCGCGGAGATGGCGGCGCGCGCGCACGACGTGGCCGCTCTCGCCATCAAGGGCCGTGCCGCGCACCTCAACTTCCCCGACCTCGCCCACGAGCTCCCGCGCCCGGCCACCGCCGCGCCCAAAGACGTCCAGGCGGCCGCCGCGCTAGCCGCCTCCGCTGACTTCCCGGCCTCTGCCACTGCCACTGCCAATGCCGGCGCCAAGAACCCCGACGGCCCGGAACCCAACGCCGCCTCTGCGTCGACACCGCCGGACACAGCCGAGGACGCGCTGTTCGACCTCCCCGACCTCCTCTTCGACCTCAGACACGGCCCTCCGTCCTGCCAGGTCTCGTGCGCTTCCACGTGGGACGACGACGTGGCCTTCGCCGGCCCCGGCGCCGGCGTGTTCCGCCTGGAGGAGCCGCTGCAGTGGGAGTATTGAAAACCCGCCCGGTCGCCGCCGCCGCCGCTGCATGCATGTGTGGCTCGTAGATGGTCGTTACCACATCGGACCAGAAATCATGTCGAAACGATCGGTGAACCAGCGCTGCGTTTGCCTTTACTAAATTTAGGAGTCGGGCCATGTACAAATTGGGGTTAGCCCTGTGTTCTTTATCGTATTTGCTTTGCGTGGATTGTGGGGCAAGAATATGTTTGTTTGTGCAATTAACAACGCATCTCTTCCTTCCTTCTTTCTTTCTTTCCTTCTTTCTTTTAGGGCGCGCTAGTGGTAAAGGCGATCGGTGGAGCGGGATTTGTTGGACTAATGCGGCACGAAATGCTTTAAGGATGGATGCAGCTTTTAAATTACGGCGATATTCGACTGGCTAGCCCTGTCCTTTCACTCCACTGATCCGGCACAAAGCGCCGGCAGCAATCATGCATTCAACCTCGATCCGATATTCCCGCAGATTAAATTTATCTTCTCTTTTCATATGATATGAGGCTGGAGCTACATGTTCTTATTCGCCCTTGGGAAAATTACGTGTCCTTTTACAATTTCAG

>CDS

ATGGGCGCCACGGAGGATTCCTCCTCCGGCTCCGAGACGACCACCTCGTCCTCGGTGGAGGCCCTGGCCTCGCCGCCGTCCCCGACGGCCACCACCGCGTCGTCCAAGAAGAAGCGGGCGCGCAACGACGGGCGGCACCCGACGTACCGCGGGGTGCGCATGCGGAGCTGGGGCAAGTGGGTGTCGGAGATTCGGGAGCCCCGCAAGAAGTCGCGCATCTGGCTCGGCACCTTCGCCACCGCGGAGATGGCGGCGCGCGCGCACGACGTGGCCGCTCTCGCCATCAAGGGCCGTGCCGCGCACCTCAACTTCCCCGACCTCGCCCACGAGCTCCCGCGCCCGGCCACCGCCGCGCCCAAAGACGTCCAGGCGGCCGCCGCGCTAGCCGCCTCCGCTGACTTCCCGGCCTCTGCCACTGCCACTGCCAATGCCGGCGCCAAGAACCCCGACGGCCCGGAACCCAACGCCGCCTCTGCGTCGACACCGCCGGACACAGCCGAGGACGCGCTGTTCGACCTCCCCGACCTCCTCTTCGACCTCAGACACGGCCCTCCGTCCTGCCAGGTCTCGTGCGCTTCCACGTGGGACGACGACGTGGCCTTCGCCGGCCCCGGCGCCGGCGTGTTCCGCCTGGAGGAGCCGCTGCAGTGGGAGTATTGA

>DNA

CCATGCAACGTTCCCCAGCAGTGCATGCGGTGGATAAGATGGGTTCGCTAGAAGCAGCAACACCGAGTGGAAGAAGGGTGGGTTGTGCACGAGGATGTAGAGGTGATGTGATGTGATCTGGTGACAGCTAGGTTTACCGACGCCTCCAATATATAGGCTTTCGTAGTAGGTAGATATCGGCTTGACCGACGCTAGAGTCACAAAGATCCCATGGATCCGACGTCTTGGATACCTAGCGCGCCACGAAAGACGGGGATTCGAAGGCCTGCTCTCTCGTACAGTCATGCATGCGGGGAATAAGATAGGCTTGCGAGAAGCATCAACAACGAGCGGAAAGTCGGTGGATTGTGCGCGAAGAGGTAGAGCAGATATGATGTGGTCTGGTGACAGCTAGGGTTAGCAACACCTTAAATATATAGGTGATTGTAGTAGGTAGACGTGGGCTGGACTAACGCCCGAATCGGCAACAACCCTTGGGTACCTAGCGCGTCCATTAATGAATCGTATTGTGAATGAATTATCTATTTCTGATCGGCTAAAATTAATCACACGCATGACATAGGTCTAAACTCGACTTGTCGAGGAAACCCGACACAAGTGTGCGAGGCGGGCGGGAAATGAGGATCACATGGGAATCACTCCTCTTGCCATACTCCAATAACATGCGAAAGGGGATTCCTTTAAGGGGGCTAAAAATTTATTTACTTGGGCATGGTACTAAAGTTTCCACCACCTTCCTTCCATGACACCCATTTAGCCTTTTGAGATTTTTCTAAGGATCTAATTCCATCTATATTTCAGTAGGATGTCCATTTACTTTGCACCATAGTGTATTAAGTAATAACTTACAAGTTACAGTATTTTGGAGGTACTTTACAACATAGGTCTATAGGTGTGATTTTATGTGTTCAAACCAAATATCTAAGTTGATATTGCTGGTCAAACTTTTCAAAGTTTGGCCGAAATAATGTCCAGCAGTATGACCTTTTTCTGAAGCTGGAGGGAGTTTTTTTAAGGGTGAAACTGGGCGGAGTTCTTTTTAAGGGTGAAACTGGAGGGAGTTGAATTGGTTGTAATTCATTTTTTGGAAGATTTTGTAAAGCTATATGGTGCAAAGACAGCTGCATGCTGCGACAGGACTAATAAAGCTTGTTTAATCGCTCAAAAACAATGCTCGTGAGTTAGTATTCAGTTCCGAAAGGCCAACACGCTGGCCTCACGCCTTCACCATTCGATTCCCCGCTTTGTTTTTGCTTTACACGTTGCTCCCTCCGCCTCATCTGCTCATCATAAAAACTCCCACTTCCTTCCCTCGTAACGCACCACTCACCTCACCTCCTCACGCTCTCGCTCGCTCATCACTCCCCAACTACTAACTCCACCACCAGCAGCAGGCAGCAGCTAGCGCGCGGCAGCCATGGGCGCCACGGAGGATTCCTCCTCCGGCTCCGAGACGACCACCTCGTCCTCGGTGGAGGCCCTGGCCTCGCCGCCGTCCCCGACGGCCACCACCGCGTCGTCCAAGAAGAAGCGGGCGCGCAACGACGGGCGGCACCCGACGTACCGCGGGGTGCGCATGCGGAGCTGGGGCAAGTGGGTGTCGGAGATTCGGGAGCCCCGCAAGAAGTCGCGCATCTGGCTCGGCACCTTCGCCACCGCGGAGATGGCGGCGCGCGCGCACGACGTGGCCGCTCTCGCCATCAAGGGCCGTGCCGCGCACCTCAACTTCCCCGACCTCGCCCACGAGCTCCCGCGCCCGGCCACCGCCGCGCCCAAAGACGTCCAGGCGGCCGCCGCGCTAGCCGCCTCCGCTGACTTCCCGGCCTCTGCCACTGCCACTGCCAATGCCGGCGCCAAGAACCCCGACGGCCCGGAACCCAACGCCGCCTCTGCGTCGACACCGCCGGACACAGCCGAGGACGCGCTGTTCGACCTCCCCGACCTCCTCTTCGACCTCAGACACGGCCCTCCGTCCTGCCAGGTCTCGTGCGCTTCCACGTGGGACGACGACGTGGCCTTCGCCGGCCCCGGCGCCGGCGTGTTCCGCCTGGAGGAGCCGCTGCAGTGGGAGTATTGAAAACCCGCCCGGTCGCCGCCGCCGCCGCTGCATGCATGTGTGGCTCGTAGATGGTCGTTACCACATCGGACCAGAAATCATGTCGAAACGATCGGTGAACCAGCGCTGCGTTTGCCTTTACTAAATTTAGGAGTCGGGCCATGTACAAATTGGGGTTAGCCCTGTGTTCTTTATCGTATTTGCTTTGCGTGGATTGTGGGGCAAGAATATGTTTGTTTGTGCAATTAACAACGCATCTCTTCCTTCCTTCTTTCTTTCTTTCCTTCTTTCTTTTAGGGCGCGCTAGTGGTAAAGGCGATCGGTGGAGCGGGATTTGTTGGACTAATGCGGCACGAAATGCTTTAAGGATGGATGCAGCTTTTAAATTACGGCGATATTCGACTGGCTAGCCCTGTCCTTTCACTCCACTGATCCGGCACAAAGCGCCGGCAGCAATCATGCATTCAACCTCGATCCGATATTCCCGCAGATTAAATTTATCTTCTCTTTTCATATGATATGAGGCTGGAGCTACATGTTCTTATTCGCCCTTGGGAAAATTACGTGTCCTTTTACAATTTCAG

>HvDREB4.4

>Protein

MPDSELNSPASSSASPPPSPTTARRGVLEKRGRDAGGNGAGGRHPAYRGVRMRAWGKWVSEIREPRKKSRIWLGTFPTPEMAARAHDAAAIVVKGPAAVLNFPELASSLPRPASAAPHDVQAAAARAAAMDPAPVREGMSAEPMPAAATSPLWCRGQMLSQVDDELEEIIELPSIDEDIAAAVEVAFGGTFCQDPVAEPWYEAAWLGEHAGIGSHDELAVSALGLEPSQFWGQPDGIAASGFGALLWNL

>cDNA

CCCCGCTAGCTTCAAAACACCCATCGTCGGGAGCGTGCACTCGCATTGGAAATGCCTGACTCTGAGCTGAACAGTCCGGCGTCATCGTCCGCCTCGCCGCCGCCATCCCCGACCACGGCGAGACGCGGAGTTCTGGAGAAGAGGGGGAGGGATGCCGGTGGCAATGGCGCGGGCGGCCGGCACCCGGCGTACCGCGGCGTTCGGATGAGGGCGTGGGGGAAGTGGGTGTCCGAGATCAGGGAGCCGCGCAAGAAGTCGCGCATCTGGCTGGGCACCTTCCCGACGCCAGAGATGGCGGCACGCGCGCACGACGCCGCGGCGATCGTCGTGAAGGGCCCTGCAGCCGTGCTCAACTTCCCCGAGCTGGCCTCGTCCCTGCCGCGCCCGGCCTCCGCCGCGCCCCACGACGTGCAGGCCGCGGCAGCCCGCGCCGCTGCCATGGACCCTGCCCCCGTGCGCGAAGGCATGAGCGCGGAGCCAATGCCGGCTGCTGCGACGTCGCCCCTGTGGTGCCGCGGGCAGATGTTGAGCCAGGTCGACGACGAGCTTGAGGAAATCATCGAGCTGCCATCCATCGACGAGGACATCGCGGCCGCTGTTGAGGTCGCGTTCGGGGGCACGTTCTGCCAAGACCCCGTCGCCGAGCCGTGGTACGAAGCAGCGTGGCTGGGAGAGCACGCGGGCATTGGCTCGCACGACGAGCTGGCCGTGTCCGCGCTCGGTCTGGAGCCGTCTCAGTTCTGGGGGCAACCCGATGGCATCGCCGCGTCCGGGTTCGGTGCGCTCTTGTGGAACCTGTAATAATAGCAAATAATCCGTCACTCTCCCTCTTTTTTGACTTGTGAATATGGAATTTCTTTCTCTCTTGTCAGCTTCTAAATTTTCCGTCCTTTAATTTGACTATTTGTGGCTACCCGTGACTGTGATCTAGGTGGTTGATACTGATGCACATTAATAATAATCGATGCCTCAGTGCACGTTTGTAAATAAGTTTTAGATACGTACATGTACATGCAATCATGCAGTGCAGTGCTAATTGTACGTACCTGAAATGTATACATAAAATCTCTTTCAAATTTTACATGTCGTATCCAGAC

>CDS

ATGCCTGACTCTGAGCTGAACAGTCCGGCGTCATCGTCCGCCTCGCCGCCGCCATCCCCGACCACGGCGAGACGCGGAGTTCTGGAGAAGAGGGGGAGGGATGCCGGTGGCAATGGCGCGGGCGGCCGGCACCCGGCGTACCGCGGCGTTCGGATGAGGGCGTGGGGGAAGTGGGTGTCCGAGATCAGGGAGCCGCGCAAGAAGTCGCGCATCTGGCTGGGCACCTTCCCGACGCCAGAGATGGCGGCACGCGCGCACGACGCCGCGGCGATCGTCGTGAAGGGCCCTGCAGCCGTGCTCAACTTCCCCGAGCTGGCCTCGTCCCTGCCGCGCCCGGCCTCCGCCGCGCCCCACGACGTGCAGGCCGCGGCAGCCCGCGCCGCTGCCATGGACCCTGCCCCCGTGCGCGAAGGCATGAGCGCGGAGCCAATGCCGGCTGCTGCGACGTCGCCCCTGTGGTGCCGCGGGCAGATGTTGAGCCAGGTCGACGACGAGCTTGAGGAAATCATCGAGCTGCCATCCATCGACGAGGACATCGCGGCCGCTGTTGAGGTCGCGTTCGGGGGCACGTTCTGCCAAGACCCCGTCGCCGAGCCGTGGTACGAAGCAGCGTGGCTGGGAGAGCACGCGGGCATTGGCTCGCACGACGAGCTGGCCGTGTCCGCGCTCGGTCTGGAGCCGTCTCAGTTCTGGGGGCAACCCGATGGCATCGCCGCGTCCGGGTTCGGTGCGCTCTTGTGGAACCTGTAA

>DNA

CCGGGGGAGGGGGAGGGGGGGGGGCGCATACAGTATAACTTAGAAAAAGAGAAAATGTACTCCAATTATCATTGCAACGTCAAACTGAAAACCCTCATCATTGTGAAATTAATGGGGTGGCAGCAAGTTACTAACGCACCCATCTCTCCACAAACAATATGTATAAATGTAAATTGCGCAGCACGTACACATCACTAGCTTGATGTTCGTAAGCAATTTTATCCTCTTTGCAACGGTCTCTTGGAAAATTTATCCGTTATTAGAGGGCACAACTCAGAGGTCGAGACTCGAGAGGTAGATCAATCTAGAAAAATGAACAGAGAGATCTGGAGAGATAGAGAGAGAGGGGGGAGCGTTGCTGACTGGACGGCCGCGACAGGGACGTCCGCTGGTGAGACGTCGACATCATGGCGCTCCCAACAGCCCCTACAAAACACGCCCTGAGCTTCCCCGCTAGCTTCAAAACACCCATCGTCGGGAGCGTGCACTCGCATTGGAAATGCCTGACTCTGAGCTGAACAGTCCGGCGTCATCGTCCGCCTCGCCGCCGCCATCCCCGACCACGGCGAGACGCGGAGTTCTGGAGAAGAGGGGGAGGGATGCCGGTGGCAATGGCGCGGGCGGCCGGCACCCGGCGTACCGCGGCGTTCGGATGAGGGCGTGGGGGAAGTGGGTGTCCGAGATCAGGGAGCCGCGCAAGAAGTCGCGCATCTGGCTGGGCACCTTCCCGACGCCAGAGATGGCGGCACGCGCGCACGACGCCGCGGCGATCGTCGTGAAGGGCCCTGCAGCCGTGCTCAACTTCCCCGAGCTGGCCTCGTCCCTGCCGCGCCCGGCCTCCGCCGCGCCCCACGACGTGCAGGCCGCGGCAGCCCGCGCCGCTGCCATGGACCCTGCCCCCGTGCGCGAAGGCATGAGCGCGGAGCCAATGCCGGCTGCTGCGACGTCGCCCCTGTGGTGCCGCGGGCAGATGTTGAGCCAGGTCGACGACGAGCTTGAGGAAATCATCGAGCTGCCATCCATCGACGAGGACATCGCGGCCGCTGTTGAGGTCGCGTTCGGGGGCACGTTCTGCCAAGACCCCGTCGCCGAGCCGTGGTACGAAGCAGCGTGGCTGGGAGAGCACGCGGGCATTGGCTCGCACGACGAGCTGGCCGTGTCCGCGCTCGGTCTGGAGCCGTCTCAGTTCTGGGGGCAACCCGATGGCATCGCCGCGTCCGGGTTCGGTGCGCTCTTGTGGAACCTGTAATAATAGCAAATAATCCGTCACTCTCCCTCTTTTTTGACTTGTGAATATGGAATTTCTTTCTCTCTTGTCAGCTTCTAAATTTTCCGTCCTTTAATTTGACTATTTGTGGCTACCCGTGACTGTGATCTAGGTGGTTGATACTGATGCACATTAATAATAATCGATGCCTCAGTGCACGTTTGTAAATAAGTTTTAGATACGTACATGTACATGCAATCATGCAGTGCAGTGCTAATTGTACGTACCTGAAATGTATACATAAAATCTCTTTCAAATTTTACATGTCGTATCCAGAC

>HvDREB4.5

>Protein

MAEKPCATSSSSAAADPLQVQVQAAAAGPATELSPRNPTSPNSSPAHLGDTVGTTTALAATSSGEPSPRSTGKHPFYRGIRCRNGKWVSEIREPRKARRIWLGTYPTAEMAAAAYDVAARALRGSDAVLNFPGAASSRPVPASTSPEDIRAAAAAAAAAAQLYRPHGEEAPDGTASIAATAEEQRHQGMMAREGAADGRTPYHHQMGNEDFMDEEAIFEMPQMLRNMAAGMMMSPPRLSPTASDEWPEPPGAGESLWSYHDP

>cDNA

GGCGACAGCTCATCGCAAGCCATACACACACTCTCCCGCTCAAACAACGCGGTCTGGCTAGCTGCCTGCATGCGCGCGGCGCCGCTCCTCTCTTCTCCCATGGAGAGAGAGATCAGCTTTTCACCATGGCGGACCTCACGCTTCTCCCCCGCTTCTTTACTAGCGCATATATACCTCCCGCCCTCCTGGCCTCATGCCGGATCTCACGCTTCCAGTTCTCCTCGTGCGTTTCTTGACACCTACACCTACCTAGCTCTCCTCTTCCAGACTTCCAATCCACTACTCTCAGTCTCTGCCCACAGCACACACATCGAGACGACGGGTACTTAACCGCTTACCCGCCGCCCCGCATACACCTGCGCTGCGTGCACCATAGATTAGCTAGTTCCATAGGATAGGCGCACTGCAGCTCTGGCATGCGTGTAGGTATATAGAGAGGGGCGTGCCCTAATGGTAGTACATGGCTGAGAAGCCTTGTGCAACCAGCTCCTCGTCTGCTGCTGCTGATCCTCTTCAGGTGCAAGTGCAAGCTGCTGCTGCTGGCCCGGCGACCGAACTTTCCCCGCGCAATCCTACCTCGCCCAATTCCTCGCCTGCTCACCTAGGCGACACGGTGGGGACAACAACGGCGTTGGCGGCGACGAGCTCCGGGGAGCCGTCGCCGCGGTCCACCGGGAAGCACCCCTTCTACCGCGGCATCCGGTGCAGAAACGGCAAGTGGGTCTCGGAGATCCGCGAGCCGCGCAAGGCGCGCCGCATATGGCTCGGAACTTATCCGACTGCAGAGATGGCTGCCGCGGCCTATGACGTGGCCGCCCGCGCGCTGCGCGGCTCCGACGCCGTGCTCAACTTCCCCGGCGCCGCCTCCTCGCGCCCGGTCCCCGCGTCTACCTCTCCTGAGGACATACGCGCGGCCGCAGCTGCAGCCGCGGCAGCTGCCCAGCTTTACAGACCGCACGGCGAGGAGGCGCCTGATGGCACTGCTTCGATTGCCGCGACAGCGGAGGAACAGAGACATCAAGGCATGATGGCAAGGGAAGGCGCCGCCGATGGCCGCACGCCGTACCATCATCAGATGGGCAATGAGGATTTCATGGACGAGGAGGCCATCTTCGAGATGCCGCAGATGCTGCGCAACATGGCGGCGGGCATGATGATGAGCCCGCCAAGGCTGAGCCCCACCGCCTCCGACGAGTGGCCAGAACCGCCGGGGGCCGGGGAGAGCCTGTGGAGCTACCACGATCCCTAGTATATTACATTTGCTGAAGCCGGTGAGCTGGTTATTGGTACGTACGTACTCCACTACTACTATACCAGTTACTAAACCTAATTAATCCGGAGTGGATACATGGACCCCGGCCGTTGCGGGGCATCGTCCTCTATCTGGTATATATGTCTGTGCCATAACAGTGGCATCACTAGGCGCTGGCCTGCGATATTATAGCTGCTACCTAGATAGTACTCGCCAGTTACGTCAGATTCGATATTATGTTCTTGCTAGCTACGTACGGTCATTTTCGCACATAAAGTCCGCTTGTTGATTCGCTTGGCTTATTGCTCGTTTTACTATGAGAGCAAGCAGGCACCACGTACCTTTTTCTTCCTTTTTTTCTTCTATAAAAAGTGCCGGGTGGATCGCACATCACCGCGAGACTAATGCATTGTAACTTTTGTAACTGTAGACATAAAAATATTTGGCGGTGGATAAACTAATGTCGATGCGGACGTGCATATGGATCCAATTTTCCTTTTCTTTAGTGGTGTCCTTTGGCTCTACTAGTTTATATATATACAAACTGGGGAAGTTAGCCGTTGGGAAGAGCAAAAGAGGTAAGAGTTGGTGGGCAATCTGTTATCTGTTGGATACTGTGATCGATGAAAGATCC

>CDS

ATGGCTGAGAAGCCTTGTGCAACCAGCTCCTCGTCTGCTGCTGCTGATCCTCTTCAGGTGCAAGTGCAAGCTGCTGCTGCTGGCCCGGCGACCGAACTTTCCCCGCGCAATCCTACCTCGCCCAATTCCTCGCCTGCTCACCTAGGCGACACGGTGGGGACAACAACGGCGTTGGCGGCGACGAGCTCCGGGGAGCCGTCGCCGCGGTCCACCGGGAAGCACCCCTTCTACCGCGGCATCCGGTGCAGAAACGGCAAGTGGGTCTCGGAGATCCGCGAGCCGCGCAAGGCGCGCCGCATATGGCTCGGAACTTATCCGACTGCAGAGATGGCTGCCGCGGCCTATGACGTGGCCGCCCGCGCGCTGCGCGGCTCCGACGCCGTGCTCAACTTCCCCGGCGCCGCCTCCTCGCGCCCGGTCCCCGCGTCTACCTCTCCTGAGGACATACGCGCGGCCGCAGCTGCAGCCGCGGCAGCTGCCCAGCTTTACAGACCGCACGGCGAGGAGGCGCCTGATGGCACTGCTTCGATTGCCGCGACAGCGGAGGAACAGAGACATCAAGGCATGATGGCAAGGGAAGGCGCCGCCGATGGCCGCACGCCGTACCATCATCAGATGGGCAATGAGGATTTCATGGACGAGGAGGCCATCTTCGAGATGCCGCAGATGCTGCGCAACATGGCGGCGGGCATGATGATGAGCCCGCCAAGGCTGAGCCCCACCGCCTCCGACGAGTGGCCAGAACCGCCGGGGGCCGGGGAGAGCCTGTGGAGCTACCACGATCCCTAG

>DNA

TTGTTCTGTAAAACGATTGGAATTCCTTCACTTCAATTAACGACATTGTATGGATTGATTTTTATCAAATTTAACTTCCATGCTTCAGTCTTTCTTATATGATATGAAAATTCAAACCGTTCTTTTCAAAAAATGGACGGGTGCGACAGTGTGCCCCGTTCATGGTCTAGTAATAATATTGTGATCCCAAATGTTTACTACCATACAGTAGTAGTTACCTTCATGTGAGTTTTGTATGTTATACGTAGTATCTGTCAAAATCGAGAGTATGTACATGTAGTATGTAGTATATAAGAATATTAGAGTAGTATACATCCTTTTTTGGTAGTATGTACTATTTTTCTAGTATATTTTTTGTACATACAAACATGAAGATATTTTAAATATACAAAAAAATATGGATTTACATGCAATTTTTTTGTATAGTTTGCTTTTAAATGTCTTAATATAGTATGAACACAATTAAAATAATAATCTATTATATATACGACCATGCGGTTGTATATGAGACATGAGGGTAACTATCCTCGGGTGGTAAGATATATTTTCCTACATACTTTTACGTGACATAAAATTATGAATATAAAAACACAATAGAAACTATACATATAAATGTTAATTTTATGGTTTTATTACTCATATCTTACTTTTTATGCGCAATTTTGTGTAGCGGATTAATATGAATGTAACAATTATATACCAAATGTATTTTTTATATGAAACAGTCACAAAATTACCTCAAGTGAAAAATAAGCTATTCTTCATCTGGGTGATAAATAGTTCCTTCTTGACATACATGCTTGCTTTCGGTTGCGAACAGAGTATTTTAGTGACTTCTAGTTTGGGAAAACGTAGAAGTACCCGATCGAACTGTTTATTCAATGGAAAAAGGCGCACAAGTAAGGTTCTTTTTTTCCTTGTAGGACACAACTAAAGTTGGATGTGCATAGATTTAATTTATCACTTTACAGAATATATAGCTCAATTTGTACCCGAAAAAGAAAACTATATGGGCACGGCACCAACCCCAGGTGCATGCATGTGTTGTCCCGTCTAACACAAACACAATCGGCAGGATACTTGCTTTGTCAAAGGAGCCCGGTCTGAAATAAGCCGGGCGCCGATATATGCCGAAACGTAGCAGGAGTACGTAGCTTAGCTGCGGCCACGACTAGTTTGTGAGAAGCGGCCAAATGGCCCAAAAGGTTGACAAGAAAAGCGAGCAAATCAAAAGCCCCCACCTGGCAGGCACCGGGAGGCCACCACCTGTCAGTCACGGTCACACGGAGCTGCCGTATCTTTCGCTAGCCCCAAAAGAAATTATCAGCAATCACACTGAGCGGCCCGATCTTAATTTCTCAATTAACCCCCACAAAAGCCGGGCCATCAATTAGGACAGCGACGTCGCCATCGTCTCCCTCTGTTTCGGCCCCGCGAAGGTAAAACGAAGGAGGTGAACTCGATCAGCCGCTGCAACTAATACCACTACTGATATTAGCATGCTTAATTAGTTAGGTGGAGTCTTGTTGTTTGCTCCTCAGGCGACAGCTCATCGCAAGCCATACACACACTCTCCCGCTCAAACAACGCGGTCTGGCTAGCTGCCTGCATGCGCGCGGCGCCGCTCCTCTCTTCTCCCATGGAGAGAGAGATCAGCTTTTCACCATGGCGGACCTCACGCTTCTCCCCCGCTTCTTTACTAGCGCATATATACCTCCCGCCCTCCTGGCCTCATGCCGGATCTCACGCTTCCAGTTCTCCTCGTGCGTTTCTTGACACCTACACCTACCTAGCTCTCCTCTTCCAGACTTCCAATCCACTACTCTCAGTCTCTGCCCACAGCACACACATCGAGACGACGGGTACTTAACCGCTTACCCGCCGCCCCGCATACACCTGCGCTGCGTGCACCATAGATTAGCTAGTTCCATAGGATAGGCGCACTGCAGCTCTGGCATGCGTGTAGGTATATAGAGAGGGGCGTGCCCTAATGGTAGTACATGGCTGAGAAGCCTTGTGCAACCAGCTCCTCGTCTGCTGCTGCTGATCCTCTTCAGGTGCAAGTGCAAGCTGCTGCTGCTGGCCCGGCGACCGAACTTTCCCCGCGCAATCCTACCTCGCCCAATTCCTCGCCTGCTCACCTAGGCGACACGGTGGGGACAACAACGGCGTTGGCGGCGACGAGCTCCGGGGAGCCGTCGCCGCGGTCCACCGGGAAGCACCCCTTCTACCGCGGCATCCGGTGCAGAAACGGCAAGTGGGTCTCGGAGATCCGCGAGCCGCGCAAGGCGCGCCGCATATGGCTCGGAACTTATCCGACTGCAGAGATGGCTGCCGCGGCCTATGACGTGGCCGCCCGCGCGCTGCGCGGCTCCGACGCCGTGCTCAACTTCCCCGGCGCCGCCTCCTCGCGCCCGGTCCCCGCGTCTACCTCTCCTGAGGACATACGCGCGGCCGCAGCTGCAGCCGCGGCAGCTGCCCAGCTTTACAGACCGCACGGCGAGGAGGCGCCTGATGGCACTGCTTCGATTGCCGCGACAGCGGAGGAACAGAGACATCAAGGCATGATGGCAAGGGAAGGCGCCGCCGATGGCCGCACGCCGTACCATCATCAGATGGGCAATGAGGATTTCATGGACGAGGAGGCCATCTTCGAGATGCCGCAGATGCTGCGCAACATGGCGGCGGGCATGATGATGAGCCCGCCAAGGCTGAGCCCCACCGCCTCCGACGAGTGGCCAGAACCGCCGGGGGCCGGGGAGAGCCTGTGGAGCTACCACGATCCCTAGTATATTACATTTGCTGAAGCCGGTGAGCTGGTTATTGGTACGTACGTACTCCACTACTACTATACCAGTTACTAAACCTAATTAATCCGGAGTGGATACATGGACCCCGGCCGTTGCGGGGCATCGTCCTCTATCTGGTATATATGTCTGTGCCATAACAGTGGCATCACTAGGCGCTGGCCTGCGATATTATAGCTGCTACCTAGATAGTACTCGCCAGTTACGTCAGATTCGATATTATGTTCTTGCTAGCTACGTACGGTCATTTTCGCACATAAAGTCCGCTTGTTGATTCGCTTGGCTTATTGCTCGTTTTACTATGAGAGCAAGCAGGCACCACGTACCTTTTTCTTCCTTTTTTTCTTCTATAAAAAGTGCCGGGTGGATCGCACATCACCGCGAGACTAATGCATTGTAACTTTTGTAACTGTAGACATAAAAATATTTGGCGGTGGATAAACTAATGTCGATGCGGACGTGCATATGGATCCAATTTTCCTTTTCTTTAGTGGTGTCCTTTGGCTCTACTAGTTTATATATATACAAACTGGGGAAGTTAGCCGTTGGGAAGAGCAAAAGAGGTAAGAGTTGGTGGGCAATCTGTTATCTGTTGGATACTGTGATCGATGAAAGATCC

>HvDREB4.6

>Protein

MEQGREGLMGSMCGRRPRAETRHPVYRGVRFRAGKWVSEIRELRKHTRIWLGTYPTPEMAAAAYDAAALALRGAGTALNFPDAARSRPAPASMSADDVRAAAAAAAAAAMGSTWTSHRGDQCYDDQPRGGESHRRELDDTVGVVDEDDVFEMPRLMVSMAEGLMINPPVLGTAAADGCSAASYYAEAEDEGAVSLWDHS

>cDNA

GAAAGAGCCACTCCTTTTTGCTCCCTGTGCTTCACAGCTAATCTCTAGCCCGGCATCGATCGAGCGAGCTCGCAAAGCAGAAAACCGCGTATGATCTCACCAACTCCGGCCGCTTCCGAGCTTTTAAACCCTCCTGAAACCCCCGCCTGGATTAGAGTACCCCAGCGCTATATATCGTGGTCAAAGTGTCTGCTAATTGGCATTGCGAATACTGTTACAAGCTGGTTTGTTCACTGGCTCCTCGCTCGCTTTGCTACGTTCCTCGTGCAGCTCGGTTGAGTACGTGTTGGTTGGTGCAATGTACGTAGTCCGTTGCATGTGTTGGTAGGGAGAGTGTTGCGCTGCACGGGTGTGAAACTCTGAGTTGGGTTCGTTGGCTGGTACTCCTACTTGAATGGAGCAAGGAAGGGAGGGGCTGATGGGGAGCATGTGCGGGAGGCGGCCGAGAGCGGAGACGCGCCACCCTGTGTACCGCGGCGTGCGGTTCCGGGCGGGGAAGTGGGTGTCGGAGATCCGGGAGCTGCGCAAGCACACCAGGATCTGGCTCGGCACCTACCCGACGCCCGAGATGGCCGCGGCGGCGTACGACGCGGCCGCATTGGCCCTGCGTGGCGCGGGGACAGCGCTCAACTTCCCGGACGCGGCGAGGTCGCGCCCGGCGCCGGCGTCCATGTCCGCCGACGATGTCCGTGCCGCGGCGGCCGCTGCCGCCGCGGCCGCGATGGGCAGCACCTGGACTTCTCATCGGGGCGACCAATGCTATGATGATCAGCCGCGTGGAGGCGAGTCGCACAGGCGTGAGCTTGACGACACGGTCGGCGTCGTGGACGAGGACGACGTGTTCGAGATGCCGCGGCTGATGGTGAGCATGGCGGAGGGGCTGATGATCAACCCGCCGGTGCTGGGCACAGCGGCGGCAGACGGTTGCTCGGCGGCGTCGTACTACGCGGAAGCTGAGGATGAGGGCGCCGTGAGCTTGTGGGATCACTCTTGATCGATCACGTAATGGAGATAGTGGAGGAGGGTGTGACTAAGACCGATAGGGACGCGCGCCATGAGCCCATGACCTGCTGGTGGCGATGTGGGATCGCCCGTGATGTGGACGGACGAATGTTTGTTGGCCAAACGTAGTATATATGTACGCTCTGTAACTGTAATGCGCCATGTAGTACGTAGCATGGATGTGAAATGGATGGTTGGGACACAACATGGATACAGGCATGTTAGTTATGGTGTCATCTTCTTCTTTAAATCATATATTTATATTTACTAACAAAATATATGTACGCTCTGTAATGCGCCGTGTACGTAGCATGGATGTGAAATGGATGGTGG

>CDS

ATGGAGCAAGGAAGGGAGGGGCTGATGGGGAGCATGTGCGGGAGGCGGCCGAGAGCGGAGACGCGCCACCCTGTGTACCGCGGCGTGCGGTTCCGGGCGGGGAAGTGGGTGTCGGAGATCCGGGAGCTGCGCAAGCACACCAGGATCTGGCTCGGCACCTACCCGACGCCCGAGATGGCCGCGGCGGCGTACGACGCGGCCGCATTGGCCCTGCGTGGCGCGGGGACAGCGCTCAACTTCCCGGACGCGGCGAGGTCGCGCCCGGCGCCGGCGTCCATGTCCGCCGACGATGTCCGTGCCGCGGCGGCCGCTGCCGCCGCGGCCGCGATGGGCAGCACCTGGACTTCTCATCGGGGCGACCAATGCTATGATGATCAGCCGCGTGGAGGCGAGTCGCACAGGCGTGAGCTTGACGACACGGTCGGCGTCGTGGACGAGGACGACGTGTTCGAGATGCCGCGGCTGATGGTGAGCATGGCGGAGGGGCTGATGATCAACCCGCCGGTGCTGGGCACAGCGGCGGCAGACGGTTGCTCGGCGGCGTCGTACTACGCGGAAGCTGAGGATGAGGGCGCCGTGAGCTTGTGGGATCACTCTTGA

>DNA

GTGCTCATCAAACGCCACCGTGTGAAAAAGATCTTTGGAGATGGTGAGGTAGAACCAGCGGATGATGGTGGCGTCGAGGATCATCCAGTCCTCGTCGTTCACCATGAGGGACGAGTCCACGGTGCCATCGATGTGGCCTTGAAGGAGGTACTCACGGAACACCAGGGAGAAGTACCTCTTCCAGGCCGACTAGGAGCCGGTGGACTGGTCAAGGACCACCCGGACGCGGTCAAAGATGTTGAGATCGCGAACGAGGGTGACGTCGGGGACGGCAAACGGATTGCTGCCGGAGGAGATTGAGGAACGCGGAGAGACCATGGTGGTGTCAGGGGATGAAGGGTTGTGGGGGGCGAAAGCGATGGAGAGGTGATGTGGCGGAGGTGGCGGAGGCGCTCAAGATGGCGCGGTGGCGGCGGCGGTTGCTGAGGCGGCGACGGCGAGGGATGCTGAGGTGGCGATGGTGGCGGCTAGGTCGGGATCGTGGAGGAATGATACCATGTAGATTAGGTTTAAGGCTGTGCAACACACAATATTGATTGGATGCACTAGACACGTATATGTAGGTACAAGATGAGCCTCTATCTCAACTATACAAAGGACTAGGAGGAAGATCCAATAACGCAATGCACATATATATTCAACACACAGAAGAGCTTATTGAAAAAAAAAGATGCAGAGGAGAGCTGCGAATTTTATGAAGAGGGAAGCTGCGTTGATACTGACAATACTATTTGGAGGATAGCATCAAGAGCCCCCTTTCCCAAATATTGCAAAAGGTAGACAAGAATGCATGCTTGGATTGAGCTTTACTCCAAGCTAGCCAAATGGACATATGGGGTCGCCACATATATTCCCCAGGCCCATGCCTGTAATCTTATATGCATACGTGTCCACTTAGAATTTGGAAATAATTCTCTAGATGGAGCCATGCGTGTTGCATGGCCATTAGTGGCGCGAGAAGACAAAGGCACTGATAATAACAACTGTATGCGCGAGTTACACCTGCTGCCGGTGGTGACACGCCGATCTCCATTCCTTGTTCCCTACAAAACCAAAACCATGTTCATCTGTGGACATGCATGCACGCATGTACCCATATAACGGCTGACATTCTTTCACCGCTACGCCACCGCGTCCGAACGACCTCAAATCCCTACCACACACGCACTGCTAAGCCCAAGCATGAACATGTCACGCGGGGAGAAAATATCTGACCGCCCACCGGCGGCACGTCCCTACTCCGTAATCTAGTACTGGATATGTTAGGTGGCACGCAAGAGAACAAAGGAATCATGCATCATCATCATCGCCTACTCATCGCTGGTTCCATCTCTCACTTTCGCTTTTCCCATAAAACCCCTATTATATTGTCCTTGAACGGGTCAACACAGGTGTTGGACCATCGGCGATGTCTCCGGCAGTCAACTCCGGTGGCCACTCCGGCAGCCCGGCGTGCCGCCACCTGGATTTTGATTCGTAACAACCCGGTAATGATCTTATGATGTCGTACTAAACATCGCCGCCCACTAACCCAAAAGCGGCTTTCTAAGCCTAAGATTGCACCCCTATGTGGCGGGCCTCCATTGGGCGGCCCGGGCCAAAGCCTGAAAGAGCCACTCCTTTTTGCTCCCTGTGCTTCACAGCTAATCTCTAGCCCGGCATCGATCGAGCGAGCTCGCAAAGCAGAAAACCGCGTATGATCTCACCAACTCCGGCCGCTTCCGAGCTTTTAAACCCTCCTGAAACCCCCGCCTGGATTAGAGTACCCCAGCGCTATATATCGTGGTCAAAGTGTCTGCTAATTGGCATTGCGAATACTGTTACAAGCTGGTTTGTTCACTGGCTCCTCGCTCGCTTTGCTACGTTCCTCGTGCAGCTCGGTTGAGTACGTGTTGGTTGGTGCAATGTACGTAGTCCGTTGCATGTGTTGGTAGGGAGAGTGTTGCGCTGCACGGGTGTGAAACTCTGAGTTGGGTTCGTTGGCTGGTACTCCTACTTGAATGGAGCAAGGAAGGGAGGGGCTGATGGGGAGCATGTGCGGGAGGCGGCCGAGAGCGGAGACGCGCCACCCTGTGTACCGCGGCGTGCGGTTCCGGGCGGGGAAGTGGGTGTCGGAGATCCGGGAGCTGCGCAAGCACACCAGGATCTGGCTCGGCACCTACCCGACGCCCGAGATGGCCGCGGCGGCGTACGACGCGGCCGCATTGGCCCTGCGTGGCGCGGGGACAGCGCTCAACTTCCCGGACGCGGCGAGGTCGCGCCCGGCGCCGGCGTCCATGTCCGCCGACGATGTCCGTGCCGCGGCGGCCGCTGCCGCCGCGGCCGCGATGGGCAGCACCTGGACTTCTCATCGGGGCGACCAATGCTATGATGATCAGCCGCGTGGAGGCGAGTCGCACAGGCGTGAGCTTGACGACACGGTCGGCGTCGTGGACGAGGACGACGTGTTCGAGATGCCGCGGCTGATGGTGAGCATGGCGGAGGGGCTGATGATCAACCCGCCGGTGCTGGGCACAGCGGCGGCAGACGGTTGCTCGGCGGCGTCGTACTACGCGGAAGCTGAGGATGAGGGCGCCGTGAGCTTGTGGGATCACTCTTGATCGATCACGTAATGGAGATAGTGGAGGAGGGTGTGACTAAGACCGATAGGGACGCGCGCCATGAGCCCATGACCTGCTGGTGGCGATGTGGGATCGCCCGTGATGTGGACGGACGAATGTTTGTTGGCCAAACGTAGTATATATGTACGCTCTGTAACTGTAATGCGCCATGTAGTACGTAGCATGGATGTGAAATGGATGGTTGGGACACAACATGGATACAGGCATGTTAGTTATGGTGTCATCTTCTTCTTTAAATCATATATTTATATTTACTAACAAAATATATGTACGCTCTGTAATGCGCCGTGTACGTAGCATGGATGTGAAATGGATGGTGG

>HvDREB5.1

>Protein

MQQGEYRSSSSSEGSAGSAAAAAAAAAAAAMAPLAAAAAAVAAKEEHNVTVAVAPPMPMAMAMPLQQQQPRKQYRGVRMRKWGKWVAEIREPHKRTRIWLGSYATPVAAARAYDTAVFYLRGRSARLNFPDEISALALSSPEAAEAGGGEMAGELADGGALSAASIRKKAIEVGSRVDALQTGMTTMVAAPAHHRERQRLHHHHHAEPHGEELHRHVKQQRTAWNGRAKNPDLNQAPSPDTSDAE

>cDNA

CCTTTGGGGTGCGGCTGGCTCGGCTTGGCTTGGCTTGGAACGGGTCAGGCCAGGCTCCCTTCCAAACAGCACAGTAGAGTACACTGCTGCTGCCGTCGATGTCCTCTTCCCTTCTCTCCCTCCCGAGCGGAGCTGCGCTCCAGTTTATGATTATCCCAACCAAACTCTAATCATACCCCCCACCCACAAAATCCCAATACAAATACCGCCCAGCAAATAGATAGCCGGGACTGGAGCCACGGCAAGAAAGAGAAGCCAGCTCCGCTGCTCCAACCCAAAAGCTACTCCTCCTTCCGGCACGCGGCAGGGAGGTGAGAGAGAGAGAGAGAGAGACCAGCCGAGGCGGCGGCCGGCCACCATAGCAGCTTAGGTTAGGCAGGCATCTTCCCTCATGCAGCAGGGCGAGTACCGCTCGTCGTCTTCGAGCGAGGGCTCGGCGGGTTCTGCGGCGGCTGCGGCTGCGGCTGCGGCGGCCATGGCGCCCCTGGCGGCTGCGGCCGCGGCGGTGGCGGCCAAGGAGGAGCACAACGTGACGGTGGCCGTGGCGCCGCCCATGCCCATGGCCATGGCGATGCCGCTGCAGCAGCAGCAGCCGCGGAAGCAGTACCGCGGCGTGCGCATGCGCAAGTGGGGCAAGTGGGTGGCGGAGATCCGCGAGCCGCACAAGCGGACGCGCATCTGGCTGGGGTCCTACGCCACGCCCGTCGCCGCCGCGCGCGCCTACGACACGGCCGTCTTCTACCTCCGCGGCAGGTCGGCGCGCCTCAACTTCCCCGACGAGATCTCCGCGCTCGCGCTGTCGTCGCCCGAGGCCGCCGAGGCCGGTGGAGGGGAGATGGCGGGCGAGCTGGCCGACGGCGGCGCGCTGTCGGCGGCTTCGATCCGGAAGAAGGCCATCGAGGTCGGGTCCCGCGTGGACGCGCTCCAGACCGGCATGACCACCATGGTCGCCGCGCCCGCGCACCACCGGGAGCGGCAGCGGCTCCACCACCACCACCACGCGGAGCCGCACGGCGAGGAGCTGCACCGCCACGTGAAGCAGCAGCGGACGGCGTGGAACGGGCGCGCCAAGAACCCGGATCTCAACCAGGCGCCCAGCCCGGACACCTCCGACGCCGAGTGAAGCAGCCGGAAGAAGCGGCGGCTTCCAGTCATCTACAACCAACCAGTCGACCATGCAGCCAGTCTCCGGAGAGCAGCCAGCAGCGTATGTTGATGAAGCAGCACAAGCAACTCAATCCAGTCCACCACCCAACGGTCTCCCTAGCAAGCTCCGCCGCGTCGGCGGGCGAGGTGCCGGTGGTCGGTGGGTCGGTGGCGAGATTATTTTTCAATACGTTGGTCTGGATAAGAACAAGGGAGGGGGAGGGGAGGCGGGGGACGCCACAAGTGGCGGTCTTTCCAAATGTCAAAAAAGACAGCTGTAACAGTGATAAAACAATCGCCACCATCTTCTTCTTTTTTCTCTCTGTTTCTGTTATCGAGTAGATAAGCAGCGATTAGCAGTGCAACTTCTGTTAATTAATCGTCCGTCGCCCCCTTCGTTGTTAATTAACTGCTGAGTGCATCGCCTCTGGATTTTGATATCGTATTATCTGAGTAGTGTAATTGAAATGATCCAGTGGTTGGCTTGGCGGCTTAATCCGGCTGTCTGCTGGTGGGAGCAAAGCAAAGCAAAGCAAGCGAGCATATCCGCGTTAGGTTAGGTTAGGCATCAGCAGGCACTAATCAGTTGGGGTGAGACTTTTCTCTATCCATGTCTCTGCTGCTGCATGCGTGTGCCTCAATCCGAATGTGCCGGTGCAATTATGGATCGGGCAGCGGCGGGCCGTGCTGGACTGGCACTGCTGGCTAATCTAATCTGATCTCATCTCATCTCATCTCCCCAGCGGACGAAGTGGAGCTAGCTAGCTACAGGGATATTTGATGGATCACCGATCACGTGGTGGCATGGCGATTAGGC

>CDS

ATGCAGCAGGGCGAGTACCGCTCGTCGTCTTCGAGCGAGGGCTCGGCGGGTTCTGCGGCGGCTGCGGCTGCGGCTGCGGCGGCCATGGCGCCCCTGGCGGCTGCGGCCGCGGCGGTGGCGGCCAAGGAGGAGCACAACGTGACGGTGGCCGTGGCGCCGCCCATGCCCATGGCCATGGCGATGCCGCTGCAGCAGCAGCAGCCGCGGAAGCAGTACCGCGGCGTGCGCATGCGCAAGTGGGGCAAGTGGGTGGCGGAGATCCGCGAGCCGCACAAGCGGACGCGCATCTGGCTGGGGTCCTACGCCACGCCCGTCGCCGCCGCGCGCGCCTACGACACGGCCGTCTTCTACCTCCGCGGCAGGTCGGCGCGCCTCAACTTCCCCGACGAGATCTCCGCGCTCGCGCTGTCGTCGCCCGAGGCCGCCGAGGCCGGTGGAGGGGAGATGGCGGGCGAGCTGGCCGACGGCGGCGCGCTGTCGGCGGCTTCGATCCGGAAGAAGGCCATCGAGGTCGGGTCCCGCGTGGACGCGCTCCAGACCGGCATGACCACCATGGTCGCCGCGCCCGCGCACCACCGGGAGCGGCAGCGGCTCCACCACCACCACCACGCGGAGCCGCACGGCGAGGAGCTGCACCGCCACGTGAAGCAGCAGCGGACGGCGTGGAACGGGCGCGCCAAGAACCCGGATCTCAACCAGGCGCCCAGCCCGGACACCTCCGACGCCGAGTGA

>DNA

TTCGTGTAAGGTGATAGCCAAATCATAATTAAGGGCCATATCAAGATTAAAAATGCAATGACTATCAAACAAACAAGTTTTGTTGTGAAAAACAATTTAAATCATGGACCATTGGACCTGGTTATGGGTGGATAGGATCAATCGTGTCAAAATAAAGTGGTGCTTTTAATGTTGGTTCAATCATTGTTGCAAGAGCTTGGTATTATATTTAACCTAAAGCAGCAATATTACGGCGTGATAATCTTGGTGAAACTTATCAGTGAGTCAATCTTGTGTTTCATGCAAGAACTAAACATATTGAAGTGGATGGCCACTTTGTCACAAAAAGAGTAGCAAACAAGTTCCTCAACATTCATTTTATAACCACAAGAGATCAAGTTGCACATGGGTTTACAAAACCATTGACAGTACGGCAACATGAAGCATTTAGTCGCATTCTCAACATAGATAAGTTATGATTGACAGTGGATGTTCAGATACTTTGTATAGTTTGTATTTCGTACATGTGATTGTTGTGTAATCCTGGCAATTGGTTTGTGTATGATATGTATGTGTCTCATGTATAGTTATCAGAAAAGCCAAGTCGAGATCAAAACCGACCCAGAACCCATGTAATCTTCCACAAACGAGTCCCTGCTGATCGATATACAACAAGCTCTTCCATCTAGTTTTACATATATAAATAGTCAATTCGATACTTGCTCTCCAAATATGTGAGCGCACTTGTGATACTCTCACAGCAAAGGAGCACACTGTTTTGGCAGTTAAAGAAATTATAGTACCTTGTGGCCGTTGATGTAACTATTAGGATTTATTTTCCTTTTCTCTTCTTAGGATGATTTTACTAGGAGTTCTAGTTTGGTTTCCAAAGTTTAGGCCACACAATTTTACTTGTGGCCGATATGAGCACTAGTCGCCAATCCATTTGCTTGATGTGATTGTCCTAAGCGACTTTGCATGTCACATGAAAAAAATGGCATATCATGTGCATCACATGATGAAGAAAACGTAAGGACGATCTTTTTCGTTTCCTCTTGCGCGGGTAAGGATAATCTTTTTCATTGCCTAGTCATATGTCTATTTTGTAAGACATGTAGTAGTACATTTCTTCTAGAATTTGCCACTTATTGTATACTCAAGCGATCACTTGTTGACCCCGAAAATGTGAACGGAACTTGGACAGGAAGAAACCGGAGCGGCAACGGAGGTCAACAAAATTACAGCGTGGCGACAAGTGCGCACGAGGGTTGGTGAGCAACAGAGAAGGGTACATGGATTGTGACGTTGCGGTCATCTTATCATCGTAGTATAATATAAACTTACCTTACCACAAGAAAGAACAGTTCGTGTTTTTTCTCCTGCCTGGACCAGCAGCAACAACGATGGTGTTGTTTCCACAGCCAGAGCAAGGAATTGCACGCCAAATACAAATTCCAATCCTACAAGGTTGACGCTAGCTGTACCCAAGTACGAGTACAATACAAATACAACCGGCCGACGTTGTTGCTACGTATGGAGTACTGGTCCAAATACGTACGTGGGGATGGATATGCATGGACCTGTGGGGTGTCGTGTCCGGACGCAAGTTAGACGGTGGACCTAGCGATTTCCTTTGGGGTGCGGCTGGCTCGGCTTGGCTTGGCTTGGAACGGGTCAGGCCAGGCTCCCTTCCAAACAGCACAGTAGAGTACACTGCTGCTGCCGTCGATGTCCTCTTCCCTTCTCTCCCTCCCGAGCGGAGCTGCGCTCCAGTTTATGATTATCCCAACCAAACTCTAATCATACCCCCCACCCACAAAATCCCAATACAAATACCGCCCAGCAAATAGATAGCCGGGACTGGAGCCACGGCAAGAAAGAGAAGCCAGCTCCGCTGCTCCAACCCAAAAGCTACTCCTCCTTCCGGCACGCGGCAGGGAGGTGAGAGAGAGAGAGAGAGAGACCAGCCGAGGCGGCGGCCGGCCACCATAGCAGCTTAGGTTAGGCAGGCATCTTCCCTCATGCAGCAGGGCGAGTACCGCTCGTCGTCTTCGAGCGAGGGCTCGGCGGGTTCTGCGGCGGCTGCGGCTGCGGCTGCGGCGGCCATGGCGCCCCTGGCGGCTGCGGCCGCGGCGGTGGCGGCCAAGGAGGAGCACAACGTGACGGTGGCCGTGGCGCCGCCCATGCCCATGGCCATGGCGATGCCGCTGCAGCAGCAGCAGCCGCGGAAGCAGTACCGCGGCGTGCGCATGCGCAAGTGGGGCAAGTGGGTGGCGGAGATCCGCGAGCCGCACAAGCGGACGCGCATCTGGCTGGGGTCCTACGCCACGCCCGTCGCCGCCGCGCGCGCCTACGACACGGCCGTCTTCTACCTCCGCGGCAGGTCGGCGCGCCTCAACTTCCCCGACGAGATCTCCGCGCTCGCGCTGTCGTCGCCCGAGGCCGCCGAGGCCGGTGGAGGGGAGATGGCGGGCGAGCTGGCCGACGGCGGCGCGCTGTCGGCGGCTTCGATCCGGAAGAAGGCCATCGAGGTCGGGTCCCGCGTGGACGCGCTCCAGACCGGCATGACCACCATGGTCGCCGCGCCCGCGCACCACCGGGAGCGGCAGCGGCTCCACCACCACCACCACGCGGAGCCGCACGGCGAGGAGCTGCACCGCCACGTGAAGCAGCAGCGGACGGCGTGGAACGGGCGCGCCAAGAACCCGGATCTCAACCAGGCGCCCAGCCCGGACACCTCCGACGCCGAGTGAAGCAGCCGGAAGAAGCGGCGGCTTCCAGTCATCTACAACCAACCAGTCGACCATGCAGCCAGTCTCCGGAGAGCAGCCAGCAGCGTATGTTGATGAAGCAGCACAAGCAACTCAATCCAGTCCACCACCCAACGGTCTCCCTAGCAAGCTCCGCCGCGTCGGCGGGCGAGGTGCCGGTGGTCGGTGGGTCGGTGGCGAGATTATTTTTCAATACGTTGGTCTGGATAAGAACAAGGGAGGGGGAGGGGAGGCGGGGGACGCCACAAGTGGCGGTCTTTCCAAATGTCAAAAAAGACAGCTGTAACAGTGATAAAACAATCGCCACCATCTTCTTCTTTTTTCTCTCTGTTTCTGTTATCGAGTAGATAAGCAGCGATTAGCAGTGCAACTTCTGTTAATTAATCGTCCGTCGCCCCCTTCGTTGTTAATTAACTGCTGAGTGCATCGCCTCTGGATTTTGATATCGTATTATCTGAGTAGTGTAATTGAAATGATCCAGTGGTTGGCTTGGCGGCTTAATCCGGCTGTCTGCTGGTGGGAGCAAAGCAAAGCAAAGCAAGCGAGCATATCCGCGTTAGGTTAGGTTAGGCATCAGCAGGCACTAATCAGTTGGGGTGAGACTTTTCTCTATCCATGTCTCTGCTGCTGCATGCGTGTGCCTCAATCCGAATGTGCCGGTGCAATTATGGATCGGGCAGCGGCGGGCCGTGCTGGACTGGCACTGCTGGCTAATCTAATCTGATCTCATCTCATCTCATCTCCCCAGCGGACGAAGTGGAGCTAGCTAGCTACAGGGATATTTGATGGATCACCGATCACGTGGTGGCATGGCGATTAGGC

>HvDREB5.2

>Protein

MEREATAFYPPAPAPPQRPTLAQPLSSRAPPSGVTVASAGRGGGGGAGRQYRGVRMRKWGKWVAEIREPNKRSRIWLGSYATAVAAARAYDTAVFYLRGRSARLNFPDQLLDGAPAAPGDLTAAAIRKKAAEVGARVDALHSGGILVPGAPLPAPPSSPSQRRRPKNPDLNREPTPDTDDDE

>cDNA

CGGCACGAGGCAGCGGCAGAGCGCCCACGCCGCAGGCAGGCGCGCACCGCAGCGGCAACCAACCAGCCCCCACACCCCCACCCCCGCCGAATCGGCTGCGCGGGCGCCGCCATGGAGAGGGAAGCCACGGCCTTCTACCCGCCGGCGCCGGCGCCGCCGCAGCGGCCGACGCTCGCGCAGCCGCTCTCGTCCCGCGCGCCGCCGTCCGGCGTCACGGTCGCCAGCGCGGGGCGGGGAGGAGGGGGAGGGGCGGGCAGGCAGTACCGCGGGGTGCGCATGCGCAAGTGGGGCAAGTGGGTGGCCGAGATCCGGGAGCCCAACAAGCGGTCGCGGATCTGGCTCGGCTCCTACGCCACCGCCGTCGCCGCCGCCCGCGCCTACGACACCGCCGTCTTCTACCTCCGCGGCCGCTCCGCGCGCCTCAACTTCCCCGACCAGCTCCTCGACGGCGCCCCCGCGGCGCCGGGCGACCTCACCGCCGCCGCCATCCGCAAGAAGGCCGCCGAGGTCGGCGCGCGCGTCGACGCGCTCCACTCCGGCGGGATCCTTGTTCCCGGCGCCCCTCTCCCGGCCCCGCCGTCGTCGCCGTCCCAGCGCCGCCGGCCCAAGAACCCCGACCTCAACCGGGAGCCCACCCCCGACACCGACGACGACGAGTGAACCTCGCCCGCCCGATTCTTCACTTCTCCTCCCACCACCGCCTACATACATACCTAGCACCAGCGCCTCTTAATTAGTCCTTAGCAGCAATCAGCAGCCAGCCAGCCAGCGAGCTAGCTAGCAGTAGCAGCAGATCAGTCATCTCAGTGAGCAAGCAATCAGATACTAGTACTAGTTCAGTTCAGTGCACAAGAAGAAGAAAGGCATGGGGAAGCAAGCAAGCAACCGAGCGTGGATCCGACACGCAAGGTGTTCGGTGAAATGCCGGGCGAGCTCGGGGGCCGACCCGACCAATAATGGAGCCGCGACGTCGCAGTCAGCTCGGCCAGGAGGTTAGTAGTAACTAAGTAGGGACCGGCCTAGGCTACCACCGGCGTCCCCCTCCCGTGGGATGTCTGTCCGGCCGAGGCGGCGACGGCGACTGCGACTGCGACGGTGAGCGAGCATGGTAGCAGTAGTGGACACAGGGCGGGCATGCTGCTAAGCGAGGCGCATGCATGGCCATGTGTGTAGCTCCCTTGAGAAGTTGAGATATAAAAGAAAAGAGTGTTGTAAAGGAAAGATGCCGGC

>CDS

ATGGAGAGGGAAGCCACGGCCTTCTACCCGCCGGCGCCGGCGCCGCCGCAGCGGCCGACGCTCGCGCAGCCGCTCTCGTCCCGCGCGCCGCCGTCCGGCGTCACGGTCGCCAGCGCGGGGCGGGGAGGAGGGGGAGGGGCGGGCAGGCAGTACCGCGGGGTGCGCATGCGCAAGTGGGGCAAGTGGGTGGCCGAGATCCGGGAGCCCAACAAGCGGTCGCGGATCTGGCTCGGCTCCTACGCCACCGCCGTCGCCGCCGCCCGCGCCTACGACACCGCCGTCTTCTACCTCCGCGGCCGCTCCGCGCGCCTCAACTTCCCCGACCAGCTCCTCGACGGCGCCCCCGCGGCGCCGGGCGACCTCACCGCCGCCGCCATCCGCAAGAAGGCCGCCGAGGTCGGCGCGCGCGTCGACGCGCTCCACTCCGGCGGGATCCTTGTTCCCGGCGCCCCTCTCCCGGCCCCGCCGTCGTCGCCGTCCCAGCGCCGCCGGCCCAAGAACCCCGACCTCAACCGGGAGCCCACCCCCGACAC**CGACGACGACGAGTGA**

>DNA

CGGCACGAGGCAGCGGCAGAGCGCCCACGCCGCAGGCAGGCGCGCACCGCAGCGGCAACCAACCAGCCCCCACACCCCCACCCCCGCCGAATCGGCTGCGCGGGCGCCGCCATGGAGAGGGAAGCCACGGCCTTCTACCCGCCGGCGCCGGCGCCGCCGCAGCGGCCGACGCTCGCGCAGCCGCTCTCGTCCCGCGCGCCGCCGTCCGGCGTCACGGTCGCCAGCGCGGGGCGGGGAGGAGGGGGAGGGGCGGGCAGGCAGTACCGCGGGGTGCGCATGCGCAAGTGGGGCAAGTGGGTGGCCGAGATCCGGGAGCCCAACAAGCGGTCGCGGATCTGGCTCGGCTCCTACGCCACCGCCGTCGCCGCCGCCCGCGCCTACGACACCGCCGTCTTCTACCTCCGCGGCCGCTCCGCGCGCCTCAACTTCCCCGACCAGCTCCTCGACGGCGCCCCCGCGGCGCCGGGCGACCTCACCGCCGCCGCCATCCGCAAGAAGGCCGCCGAGGTCGGCGCGCGCGTCGACGCGCTCCACTCCGGCGGGATCCTTGTTCCCGGCGCCCCTCTCCCGGCCCCGCCGTCGTCGCCGTCCCAGCGCCGCCGGCCCAAGAACCCCGACCTCAACCGGGAGCCCACCCCCGACACCGACGACGACGAGTGAACCTCGCCCGCCCGATTCTTCACTTCTCCTCCCACCACCGCCTACATACATACCTAGCACCAGCGCCTCTTAATTAGTCCTTAGCAGCAATCAGCAGCCAGCCAGCCAGCGAGCTAGCTAGCAGTAGCAGCAGATCAGTCATCTCAGTGAGCAAGCAATCAGATACTAGTACTAGTTCAGTTCAGTGCACAAGAAGAAGAAAGGCATGGGGAAGCAAGCAAGCAACCGAGCGTGGATCCGACACGCAAGGTGTTCGGTGAAATGCCGGGCGAGCTCGGGGGCCGACCCGACCAATAATGGAGCCGCGACGTCGCAGTCAGCTCGGCCAGGAGGTTAGTAGTAACTAAGTAGGGACCGGCCTAGGCTACCACCGGCGTCCCCCTCCCGTGGGATGTCTGTCCGGCCGAGGCGGCGACGGCGACTGCGACTGCGACGGTGAGCGAGCATGGTAGCAGTAGTGGACACAGGGCGGGCATGCTGCTAAGCGAGGCGCATGCATGGCCATGTGTGTAGCTCCCTTGAGAAGTTGAGATATAAAAGAAAAGAGTGTTGTAAAGGAAAGATGCCGGC

>HvDREB5.3

>Protein

MVKNHPGSGTSSRCVDAAAVPESGGRRPAAMAVRQYKGVRMRSWGSWVSEIRAPHQKRRIWLGSYATPEAAARAYDAALLCLKGSDAVLNFPSSSSASPPSPPHSVPADDLSPRSIQRAAAAAAAAFEATRIVVDDSCSSSAEATTPRTSVSVSTLGSADVQEHATSSMSRAASAGSPVGDHEELWTDLDAFASPKLMDLIAAGHATPFSSTWEEPEEDGEMMRLWSFC

>cDNA

ATGGTCAAGAACCACCCGGGCAGTGGCACTAGCAGCAGGTGCGTCGACGCTGCGGCGGTACCGGAGAGCGGCGGTAGGCGGCCGGCGGCAATGGCGGTGAGGCAGTACAAGGGCGTGCGGATGCGGAGCTGGGGGTCGTGGGTGTCCGAGATCAGGGCGCCGCACCAGAAGCGCCGGATCTGGCTCGGCTCCTACGCCACCCCGGAGGCCGCCGCGCGCGCCTACGATGCCGCCCTCCTCTGCCTCAAGGGCTCCGACGCCGTCCTCAACTTCCCGTCCTCGTCCTCCGCCTCACCACCCTCGCCGCCGCATTCCGTTCCCGCCGACGACCTGTCCCCGAGGTCCATCCAGCGCGCGGCCGCCGCCGCCGCCGCGGCCTTCGAAGCCACCAGGATCGTCGTGGACGACAGCTGCTCTTCCAGCGCCGAGGCGACGACGCCGCGGACCTCGGTCTCGGTGTCGACGCTGGGGAGCGCCGACGTCCAGGAGCACGCCACGTCCTCGATGTCCCGCGCGGCCAGCGCTGGCTCGCCGGTGGGAGATCACGAGGAGCTGTGGACGGACCTGGACGCCTTCGCGTCCCCCAAGCTCATGGATCTCATCGCCGCCGGTCACGCCACGCCCTTCTCGTCGACCTGGGAGGAGCCCGAGGAGGACGGCGAGATGATGAGGCTGTGGAGCTTCTGCTAGAGCGATGATGCACGTACGCACCGTTTGCAATGGATCGACTTCGACACGCGCATCCATCCATGAGTCGATCGCCATCCATTGATCATCGATTTTTCTCTTCTCCCATGGAAGCTTAGCTAGCTAGGTGCATGCATGGATCCTCTCTAGGCTAGCTCCAGCTAGGTCTCAGTCCAAGTCTGCTTCCACAGATTCTTTCGTTCGTTTTTAGTGTAAGAACATAATACTTATACTACCAC

>CDS

ATGGTCAAGAACCACCCGGGCAGTGGCACTAGCAGCAGGTGCGTCGACGCTGCGGCGGTACCGGAGAGCGGCGGTAGGCGGCCGGCGGCAATGGCGGTGAGGCAGTACAAGGGCGTGCGGATGCGGAGCTGGGGGTCGTGGGTGTCCGAGATCAGGGCGCCGCACCAGAAGCGCCGGATCTGGCTCGGCTCCTACGCCACCCCGGAGGCCGCCGCGCGCGCCTACGATGCCGCCCTCCTCTGCCTCAAGGGCTCCGACGCCGTCCTCAACTTCCCGTCCTCGTCCTCCGCCTCACCACCCTCGCCGCCGCATTCCGTTCCCGCCGACGACCTGTCCCCGAGGTCCATCCAGCGCGCGGCCGCCGCCGCCGCCGCGGCCTTCGAAGCCACCAGGATCGTCGTGGACGACAGCTGCTCTTCCAGCGCCGAGGCGACGACGCCGCGGACCTCGGTCTCGGTGTCGACGCTGGGGAGCGCCGACGTCCAGGAGCACGCCACGTCCTCGATGTCCCGCGCGGCCAGCGCTGGCTCGCCGGTGGGAGATCACGAGGAGCTGTGGACGGACCTGGACGCCTTCGCGTCCCCCAAGCTCATGGATCTCATCGCCGCCGGTCACGCCACGCCCTTCTCGTCGACCTGGGAGGAGCCCGAGGAGGACGGCGAGATGATGAGGCTGTGGAGCTTCTGCTAG

>DNA

ATGGTCAAGAACCACCCGGGCAGTGGCACTAGCAGCAGGTGCGTCGACGCTGCGGCGGTACCGGAGAGCGGCGGTAGGCGGCCGGCGGCAATGGCGGTGAGGCAGTACAAGGGCGTGCGGATGCGGAGCTGGGGGTCGTGGGTGTCCGAGATCAGGGCGCCGCACCAGAAGCGCCGGATCTGGCTCGGCTCCTACGCCACCCCGGAGGCCGCCGCGCGCGCCTACGATGCCGCCCTCCTCTGCCTCAAGGGCTCCGACGCCGTCCTCAACTTCCCGTCCTCGTCCTCCGCCTCACCACCCTCGCCGCCGCATTCCGTTCCCGCCGACGACCTGTCCCCGAGGTCCATCCAGCGCGCGGCCGCCGCCGCCGCCGCGGCCTTCGAAGCCACCAGGATCGTCGTGGACGACAGCTGCTCTTCCAGCGCCGAGGCGACGACGCCGCGGACCTCGGTCTCGGTGTCGACGCTGGGGAGCGCCGACGTCCAGGAGCACGCCACGTCCTCGATGTCCCGCGCGGCCAGCGCTGGCTCGCCGGTGGGAGATCACGAGGAGCTGTGGACGGACCTGGACGCCTTCGCGTCCCCCAAGCTCATGGATCTCATCGCCGCCGGTCACGCCACGCCCTTCTCGTCGACCTGGGAGGAGCCCGAGGAGGACGGCGAGATGATGAGGCTGTGGAGCTTCTGCTAGAGCGATGATGCACGTACGCACCGTTTGCAATGGATCGACTTCGACACGCGCATCCATCCATGAGTCGATCGCCATCCATTGATCATCGATTTTTCTCTTCTCCCATGGAAGCTTAGCTAGCTAGGTGCATGCATGGATCCTCTCTAGGCTAGCTCCAGCTAGGTCTCAGTCCAAGTCTGCTTCCACAGATTCTTTCGTTCGTTTTTAGTGTAAGAACATAATACTTATACTACCAC

>HvDREB5.4

>Protein

MVKTAAAGSNGNGVAVKQQQQQQLGGKMRTYKGVRMRSWGAWVSEIRAPGQKTRIWLGSHSTAEAAARAYDAALLCLKGGAAAADLNFPVRFPFDLPAAAMSPKSIQRVAAAAAAAGAGASVVDFACADDSASADAVDFAGADDAITPDYCSSSSNASPVSSPETASSGGAADLDGVDLLGYGYSQCSLAEIEAFFQSPKCMEYAMMDPCSAFFAPAPTAMAMEDECSWEEEGDIALWSFSMD

>cDNA

GTTTTTGGGCCCTATATAAGTAGCCCGTTCGGACCATCGACCAGACCAGTGAGCCCAGCACACAGATCATCAGCAGCATTCAGCAACAAGCCTTGCGTCGCAAACCAAAACCATCGACCGTCGCATCGGAGGAGAGGAACCGACTGAGCAGTCTGAGCTAGGAGGAGGTTCGCCGTAGCAATGGTGAAGACGGCTGCAGCAGGGAGCAACGGCAATGGCGTCGCGGTGAAGCAGCAGCAGCAGCAGCAGCTTGGAGGCAAGATGAGGACGTACAAGGGGGTGCGGATGAGGAGCTGGGGCGCGTGGGTGTCGGAGATCCGGGCGCCGGGGCAGAAGACGAGGATATGGCTCGGCTCCCACTCCACCGCCGAGGCCGCCGCGCGCGCCTACGACGCCGCGCTGCTCTGCCTCAAGGGGGGCGCCGCCGCCGCCGACCTCAACTTCCCCGTGCGCTTCCCCTTCGACCTCCCCGCCGCCGCCATGTCGCCCAAGTCCATCCAGCGCGTCGCCGCCGCAGCCGCAGCCGCCGGTGCCGGCGCCAGTGTCGTCGACTTCGCCTGTGCCGACGACAGTGCCAGCGCTGATGCCGTCGACTTTGCCGGTGCCGACGACGCCATCACCCCGGACTACTGCAGCTCCTCCAGCAATGCCTCGCCGGTGAGCTCCCCGGAAACGGCGAGCAGCGGCGGCGCCGCCGACCTCGACGGCGTGGACCTCCTGGGCTACGGTTACAGCCAGTGCTCGCTCGCGGAGATCGAGGCGTTCTTCCAGTCGCCCAAGTGCATGGAGTACGCGATGATGGACCCGTGCAGCGCGTTCTTCGCTCCGGCGCCGACGGCCATGGCCATGGAGGACGAGTGCAGCTGGGAGGAAGAAGGCGACATTGCGCTCTGGAGCTTCTCAATGGACTGAAAACTGCAGCCAAGGAAGCCGGAGCACCCAAGAAACTGACAAATCGTTACTCAAGCGAAGACCACAACTGAACTGGTCATGCATGGTGATAACTATACTTGATTTCCTATACTTAATTTGATTCATTGTACACAGCAACAATAAGGTAGTTGAGCGTTGTGGGGGGAGCTCTCATGGAGCTACATGTTTTAAGGCAGCTTCCATGGAGTTAACCTCCCTTCAATTGCTTGTTACATCCATGTTAAAAAAAGCTGCTTTGCAATTGCAAGCATATATTTCTTTTGAAACAACGGACCCTCTCATCAATTGTGTGAGTATCTGGACTATTCACTTCGAATGGTAAG

>CDS

ATGGTGAAGACGGCTGCAGCAGGGAGCAACGGCAATGGCGTCGCGGTGAAGCAGCAGCAGCAGCAGCAGCTTGGAGGCAAGATGAGGACGTACAAGGGGGTGCGGATGAGGAGCTGGGGCGCGTGGGTGTCGGAGATCCGGGCGCCGGGGCAGAAGACGAGGATATGGCTCGGCTCCCACTCCACCGCCGAGGCCGCCGCGCGCGCCTACGACGCCGCGCTGCTCTGCCTCAAGGGGGGCGCCGCCGCCGCCGACCTCAACTTCCCCGTGCGCTTCCCCTTCGACCTCCCCGCCGCCGCCATGTCGCCCAAGTCCATCCAGCGCGTCGCCGCCGCAGCCGCAGCCGCCGGTGCCGGCGCCAGTGTCGTCGACTTCGCCTGTGCCGACGACAGTGCCAGCGCTGATGCCGTCGACTTTGCCGGTGCCGACGACGCCATCACCCCGGACTACTGCAGCTCCTCCAGCAATGCCTCGCCGGTGAGCTCCCCGGAAACGGCGAGCAGCGGCGGCGCCGCCGACCTCGACGGCGTGGACCTCCTGGGCTACGGTTACAGCCAGTGCTCGCTCGCGGAGATCGAGGCGTTCTTCCAGTCGCCCAAGTGCATGGAGTACGCGATGATGGACCCGTGCAGCGCGTTCTTCGCTCCGGCGCCGACGGCCATGGCCATGGAGGACGAGTGCAGCTGGGAGGAAGAAGGCGACATTGCGCTCTGGAGCTTCTCAATGGACTGA

>DNA

GGGGGGGGGGGGGGGGTACACAGAGTGCGACCGCGCTAGGTGCACCAAGGGGGGCGGTGCCTGGCCACCACCGAAGAACTCCGGCGACGACATCGGTCGGGAGGAGACGTACGACAGACCGGCTTCCGCAACTTTGACAGAGAGAGAGAGAGGAAGAGACAAAGAGAAGGAATAAAGAGTTATGAGCTGTGGGACAAATTAACTCAGATGCTTCCCCTCTGCCGACGGTGGCTGGATAATTAGGCAGATCCGGAGGGAGCTAGGTGCAAAGTGTCACGAGCTGGCCTGCCCTGTCCACTTGACGCGGTGCTATTGGCCGATGGTGAGTTTGGACTGTGCTTGCACATTACCTGCAAGTTTTGAGAGTGAGTAGGGTCAGTTTTCAACTTATAGGACTCAACTATCACATTGGTGTAAGTTTTAAGACCCCTAATGCTATTACCTCAACAATCAATGGCGAACAAAAAGCTCGCTAGCTCCTCCGGCGCGTGCGTGCATGCATGTTACCCTGCCGTTGTCGTGCGCGTGGATTCCCACTGCCGCGCTGCGTGGGAGGAGGAGAGGGCGGACATTAGTCTCGCTTCGCCAGGGGCGGTACGTACGTACGTACGTCTGTAAACTCTAATCTAATCTAATCATGTGCAGTCAACTCGTCGGCAGGGTGTGGTGTGGTGTGGTGTACTGGTTGGTTGATTAGTTAACAGTTAACCCACAGCTGGAATCAACCGGGCGACTAATTACACATGCAGCGGAGTCCGGCGGATCGAGACGTCTGGAACCTTTTGCTTTGATGTCCTGTTTTTTTGCTTTGATGCTCACTACACGTCGTACGATCCTAGCCCTGGATTTTCACTCCGACGCCACGTGCCTCATCACCCTCATGTGGAAGGAGAGAACGCAGTGCCGGTAGTATTTGACAGCGCCTTAATGTCAAACGTTCATGGTGTTTCTGCTTAGATACTCGCACAATCTAGCTCTGGATTCTTAGCTTTTTACCACGACGCCACATGCCGGGCCATGTTCTGATGTTCAGCAGTAGTCCTAACTACTAACTGACGAACGTGCCACATGTGAAAGCGCCACGGAAGTCCATCGACCACCAGCAGCCCGGTGGCGACTTGCAATCTCACACGACCCCAAGGGCGGCGTTTACCATGGCCCGCCGCGGGACACCAATCACCACCGGGTCTTTCTTCACCTACGCAGCCAGCAGCCTCTGCTCCAAGTGACAGGTAGGTGCTGTTGACACTCGATGCACTCTGCAGTCTCCACTCGGTTTGGCCAGGCAGGCAGGCAGGCAAAGGCAACAAGTGCATCATGCCGTTGCCGTGACCGGGCAAGCCACACCCGAATCGAATCAGGGCACTGTTTTTTATGCTAGCACAGTACTGCTACAACTACAAGCTGCTAATGGACATGGCATGGCACCGGTAGCAGTAGTAGCACACAGGATGCTTCCTCCCTTGGATTTTGTTACGATATTATTTCCTCCGCATGCATTATTGCCAAGATGTACGACCGCGGGTTTGTATATGCGCACTGTTTTGTCAGAGTATCGCTGTGATGTGAACGTAATGGTTTGTTAGTTGTGCTCAGCCAGTAAACAAATCCGTGGACGAGGCACACGCAGTTTTTGGGCCCTATATAAGTAGCCCGTTCGGACCATCGACCAGACCAGTGAGCCCAGCACACAGATCATCAGCAGCATTCAGCAACAAGCCTTGCGTCGCAAACCAAAACCATCGACCGTCGCATCGGAGGAGAGGAACCGACTGAGCAGTCTGAGCTAGGAGGAGGTTCGCCGTAGCAATGGTGAAGACGGCTGCAGCAGGGAGCAACGGCAATGGCGTCGCGGTGAAGCAGCAGCAGCAGCAGCAGCTTGGAGGCAAGATGAGGACGTACAAGGGGGTGCGGATGAGGAGCTGGGGCGCGTGGGTGTCGGAGATCCGGGCGCCGGGGCAGAAGACGAGGATATGGCTCGGCTCCCACTCCACCGCCGAGGCCGCCGCGCGCGCCTACGACGCCGCGCTGCTCTGCCTCAAGGGGGGCGCCGCCGCCGCCGACCTCAACTTCCCCGTGCGCTTCCCCTTCGACCTCCCCGCCGCCGCCATGTCGCCCAAGTCCATCCAGCGCGTCGCCGCCGCAGCCGCAGCCGCCGGTGCCGGCGCCAGTGTCGTCGACTTCGCCTGTGCCGACGACAGTGCCAGCGCTGATGCCGTCGACTTTGCCGGTGCCGACGACGCCATCACCCCGGACTACTGCAGCTCCTCCAGCAATGCCTCGCCGGTGAGCTCCCCGGAAACGGCGAGCAGCGGCGGCGCCGCCGACCTCGACGGCGTGGACCTCCTGGGCTACGGTTACAGCCAGTGCTCGCTCGCGGAGATCGAGGCGTTCTTCCAGTCGCCCAAGTGCATGGAGTACGCGATGATGGACCCGTGCAGCGCGTTCTTCGCTCCGGCGCCGACGGCCATGGCCATGGAGGACGAGTGCAGCTGGGAGGAAGAAGGCGACATTGCGCTCTGGAGCTTCTCAATGGACTGAAAACTGCAGCCAAGGAAGCCGGAGCACCCAAGAAACTGACAAATCGTTACTCAAGCGAAGACCACAACTGAACTGGTCATGCATGGTGATAACTATACTTGATTTCCTATACTTAATTTGATTCATTGTACACAGCAACAATAAGGTAGTTGAGCGTTGTGGGGGGAGCTCTCATGGAGCTACATGTTTTAAGGCAGCTTCCATGGAGTTAACCTCCCTTCAATTGCTTGTTACATCCATGTTAAAAAAAGCTGCTTTGCAATTGCAAGCATATATTTCTTTTGAAACAACGGACCCTCTCATCAATTGTGTGAGTATCTGGACTATTCACTTCGAATGGTAAG

>HvDREB5.5

>Protein

MVKSAQQQQQQVALGGDGGNAAAARQGGGGGGRQQQYKGVRMRSWGSWVSEIRAPNQKTRIWLGSYSTAEAAARAYDAALLCLRAPPPTSTSPSTSPSTSPPPPCRPSPSSASPPQQQQQPAAAPCSLTPCPAPPQPGPAPAPPRRAAPPSAPRRTTSRPATATPPTTTTRPWRGATTAWTTTRWRTSTPSSSRPSAWTTP

>cDNA

CACCAGCCACACACACCCAACCACCCCCCGCTACCACTTCAACCCGCACTGGTTGGTCTTCGCACACTCTCGGAGTTCAGCATCGCTCGCGCATCATCAGAATAGTCAAGCCCGTATCACGGCGAGGAGCCGAGGAGCATCGTCGGCGAGCGTGATCGGCCGGGAAATGGTGAAGAGCGCGCAGCAGCAGCAGCAGCAGGTGGCGCTCGGCGGCGACGGCGGCAATGCGGCGGCGGCGAGGCAGGGCGGCGGCGGCGGGGGGAGGCAGCAGCAGTACAAGGGCGTGCGGATGCGGAGCTGGGGGTCGTGGGTGTCGGAGATCCGGGCGCCCAACCAGAAGACGCGCATATGGCTCGGCTCCTACTCCACCGCCGAGGCCGCCGCGCGCGCCTACGACGCCGCGCTGCTCTGCCTCCGGGCTCCGCCGCCGACCTCAACTTCCCCGTCCACCTCCCCTTCCACGTCCCCGCCGCCGCCATGTCGCCCAAGTCCATCCAGCGCGTCGCCGCCGCAGCAGCAGCAGCAGCCGGCAGCAGCCCCCTGCAGCCTCACGCCGTGCCCAGCTCCCCCGCAGCCTGGCCCGGCCCCGGCGCCGCCCCGCCGTGCGGCTCCTCCTTCGGCTCCCCGGAGGACGACCAGTCGGCCCGCCACGGCAACGCCGCCGACGACGACGACGAGGCCATGGCGCGGGGCGACGACGGCGTGGACTACGACGCGCTGGCGGACATCGACGCCTTCTTCCAGTCGCCCAAGTGCATGGACTACTCCATGATGATGGACCCCTGCAGCACCTTCTTCGCGCCGGCGCCCGTCGAGTGGGAGGAGGAAGCCGAGATCAGCCTCTGGAGCTTCTCCTCCTACAACTGATAAATGCCAGCCCCGACGACGGCCATGGAGGACAATTCAAGCTTCGACCGGTCAAATCGCCGCTAAGCAATTCCATCAACACTGACAGATTAATTACCCAACTACCACTACGAGTGACCAAGAATTGGTCACGGTAAAAATAATCGCAAGCCATGATCAATTCTTATTTCATGGTCTTGCGGAAAATGATGATTAATTATTATTTCATTGATATTCCGGCCGTGGCTAAATATACTAATAGTACCACTGACGTACTAGTCGTACGTATCCGTACTAGACCCATTATGCGTTTGTGGGGGAGCTCTGATGCAGCTACATGTTTTAAGGCAGCTTCCATGGAGCCAACTCCCCTCAATTAGTTACATGTGTACTATGTGTAAAAGCTGTTTGTGCAAGTGTGCAAGCAATGGGTTATGATGATGAACTGCCTCTTCAATTCATTTTTAGTTAGTG

>CDS

ATGGTGAAGAGCGCGCAGCAGCAGCAGCAGCAGGTGGCGCTCGGCGGCGACGGCGGCAATGCGGCGGCGGCGAGGCAGGGCGGCGGCGGCGGGGGGAGGCAGCAGCAGTACAAGGGCGTGCGGATGCGGAGCTGGGGGTCGTGGGTGTCGGAGATCCGGGCGCCCAACCAGAAGACGCGCATATGGCTCGGCTCCTACTCCACCGCCGAGGCCGCCGCGCGCGCCTACGACGCCGCGCTGCTCTGCCTCCGGGCTCCGCCGCCGACCTCAACTTCCCCGTCCACCTCCCCTTCCACGTCCCCGCCGCCGCCATGTCGCCCAAGTCCATCCAGCGCGTCGCCGCCGCAGCAGCAGCAGCAGCCGGCAGCAGCCCCCTGCAGCCTCACGCCGTGCCCAGCTCCCCCGCAGCCTGGCCCGGCCCCGGCGCCGCCCCGCCGTGCGGCTCCTCCTTCGGCTCCCCGGAGGACGACCAGTCGGCCCGCCACGGCAACGCCGCCGACGACGACGACGAGGCCATGGCGCGGGGCGACGACGGCGTGGACTACGACGCGCTGGCGGACATCGACGCCTTCTTCCAGTCGCCCAAGTGCATGGACTACTCCATGA

>DNA

AGTCTTCTAAGCATAATGCCATCAACACCAAAAACTCCTTGGAAGCAATATGTCCTTTCAACCATCACTTGAGTTGGAGCTACTCCTAGTCAAGCTTACATATCTGATTTCTTCCTTCTCTCTTTGGTCTTGCTCTAGGCCTCTAGCACATACCCCCGGAGAAGTAATCAAGGAAGCAACTCCACTCTAGTGGTCTCTAGTGTCCACCCCTAGTTCTTGGATTGGTAGCCCCTTTTCTCCATTTTCTGGTGTCATTGATGCCCATGTGGAAGAAGAAAAGCGTATCATAAAACTAGGTTGGAAAGTGGTTGAGAGTTACAACTTACAAGATTTGATTTTGTTTATTTGTGATCTTGCGATCCTTTTTTTCATTTGGTTAACTATTCCCTCCGCTCCAAAAATTCCATAACATCTTGAAGTTAAATATCTTGAAGTCTGACTAAACCTGTAAAAAAGTCTACATCCATAACATCAACTATATACTCCATCCGTCTTAAAATAAGTGTCTCGACTTTATACCAGCTCTAGTATAAAGTTATACTAAGCTTAAGACACTNNTTTTGAGACGGAGGGAGTATATATATTATAAATATGTATTTTATGATGAATATAATAAAACAAATTGGATGTTGCAGATGTCGATGTATTTTTTTGCGGACTTGTTCAAACTTAAAGATGTTTGAATCAAGACAATCTAGAGCTTCAAATATTTTGGAATAGAGGAAGTAGCTATTATGTTGTGTGTGACACTTGCTACTCCCTCCGTTCCTAAATNTAAGTCTTTTTAGAGATTCTACTAGGGGACTACATACGTAGCAAAATAAGTGAATCTAAACTTTAATGTATGTCTATATACATCCGTATGTAGTTCCCTAGTGGAACTCTAAAAAGTTATATTTAAAAACGGGGGGAGTACTTTTTAACGCCAAAATCCAACACAGAAAACAATACGAAACATAACAACCATGATTACATATTGCATATATATAGCAAGCCTAGCAGCGGATTTGCACAGGCCAGCCAGCTAGTAGTTTAGAGTGCTAAGGCGATAAACCTTCAAAATGGTGTTTCTAGATTCCTCGCTTCTGACTTCTGTATAGGTGTACATGTCGGAACGATCGATGGTTTATGCCCAGCGCTCACCAAACAAGATAAGCCACGGCATGCACGCCAGCACTTAGACCAATCATAATGAGCGAAGCATGCATGCATGCGGCTAACTCATCCTTGCTTAGTTTTGAGTAAACTTTAAGCATAAAACGTGTTGCTGCCGAGGCGCTGACATGCTGCGTACGTACTATATACTTTCTTTGTTTTTAAAATATAAGTTTTTTAGAAATTTTAATATAAACTACGTACGAATGTATATCGTTATATTTTAAAGCATAAATTCATTAATTTTGCTTTTTATGCAGTGTCTATTATAATTTTTAAAAAGATCTTATATATAGAAACGAAGGGAGTATGATCGAATTTGGAGATCTTTTCATTACTTTCAGTTAGGTTATTTCCCTTAGTGTTTCCTCTGCATTATCGCAGATGCAGCGGTACGGCACCGGGGTGGCAAAATAAATAAATAAATAAGGTACCAATAAGGTTATACTCTCTCCGTCTTAAAAATTTTGTCTTATGTTCATTTAAAAATGAATGTATCTAGATACTAGAAAGTATCAGCGAGATGTGAGCGAAATGGTTATACTACTAGAGACTAGGACACAGAACCAATTTCAGGTGAGACAAAGGAAAGCAGAAATGGCCGAGTTTATAAAAAAAAATGCACGCTTTGATGCTCGTGGTTCCTATAAATAAGCCATGGGACGCATCACTCTGCCTGCACCAGCCACACACACCCAACCACCCCCCGCTACCACTTCAACCCGCACTGGTTGGTCTTCGCACACTCTCGGAGTTCAGCATCGCTCGCGCATCATCAGAATAGTCAAGCCCGTATCACGGCGAGGAGCCGAGGAGCATCGTCGGCGAGCGTGATCGGCCGGGAAATGGTGAAGAGCGCGCAGCAGCAGCAGCAGCAGGTGGCGCTCGGCGGCGACGGCGGCAATGCGGCGGCGGCGAGGCAGGGCGGCGGCGGCGGGGGGAGGCAGCAGCAGTACAAGGGCGTGCGGATGCGGAGCTGGGGGTCGTGGGTGTCGGAGATCCGGGCGCCCAACCAGAAGACGCGCATATGGCTCGGCTCCTACTCCACCGCCGAGGCCGCCGCGCGCGCCTACGACGCCGCGCTGCTCTGCCTCCGGGCTCCGCCGCCGACCTCAACTTCCCCGTCCACCTCCCCTTCCACGTCCCCGCCGCCGCCATGTCGCCCAAGTCCATCCAGCGCGTCGCCGCCGCAGCAGCAGCAGCAGCCGGCAGCAGCCCCCTGCAGCCTCACGCCGTGCCCAGCTCCCCCGCAGCCTGGCCCGGCCCCGGCGCCGCCCCGCCGTGCGGCTCCTCCTTCGGCTCCCCGGAGGACGACCAGTCGGCCCGCCACGGCAACGCCGCCGACGACGACGACGAGGCCATGGCGCGGGGCGACGACGGCGTGGACTACGACGCGCTGGCGGACATCGACGCCTTCTTCCAGTCGCCCAAGTGCATGGACTACTCCATGATGATGGACCCCTGCAGCACCTTCTTCGCGCCGGCGCCCGTCGAGTGGGAGGAGGAAGCCGAGATCAGCCTCTGGAGCTTCTCCTCCTACAACTGATAAATGCCAGCCCCGACGACGGCCATGGAGGACAAGCTTCGACCGGTCAAATCGCCGCTAAGCAATTCCATCAACACTGACAGATTAATTACCCAACTACCACTACGAGTGACCAAGAATTGGTCACGGTAAAAATAATCGCAAGCCATGATCAATTCTTATTTCATGGTCTTGCGGAAAATGATGATTAATTATTATTTCATTGATATTCCGGCCGTGGCTAAATATACTAATAGTACCACTGACGTACTAGTCGTACGTATCCGTACTAGACCCATTATGCGTTTGTGGGGGAGCTCTGATGCAGCTACATGTTTTAAGGCAGCTTCCATGGAGCCAACTCCCCTCAATTAGTTACATGTGTACTATGTGTAAAAGCTGTTTGTGCAAGTGTGCAAGCAATGGGTTATGATGATGAACTGCCTCTTCAATTCATTTTTAGTTAGTG

>HvDREB6.1

>Protein

MDASLRTLSPSSFTGEVRSAVSSLLLSPGGASALDTVFSHLPPPVTIPPLGSSVYYRQSELLRTFAATHHQASAAAATSSSSGYAVPFPFPGAAAHDAAAAAAHDAAAAAAAGRKMYRGVRQRQWGKWVAEIRLPQNRVRVWLGTYDSPETAAHAYDRAAYRLRGEYARLNFPGVMDGADDARFPDALRHLRDAVDAKIQAIRVRMARKRARARRLREESKQQQQQNNTQRAEAAPVAPTAPRPVVSESATTSETTTTTTSSYGSPEGVLSAADFECSLQQMPSFDPELIWEMLNF

>cDNA

CTCCATTTCATTCCCGGTTGCCAGGGGCGTCATGGTGACCACGGCGCCCGGCGCCCGGCGCCCATGGGTTATATAAAGCCACACGACGCGCACCTGGCTTGGACAGACAGACAAACCTCTCACACTCCACCGCTAATCGCCACCACACCAAGACTCGCCTTTTGCTCCGGCCAAGGATGCCATTCCCGCCCCGCCCGTGAACGCCCGAACCAACTCGCTCCAGCTCCCGTCCATGGACGCCAGCCTCCGGACGCTGTCGCCGTCCAGCTTCACCGGGGAGGTGCGCTCCGCGGTGTCCTCCCTCCTGCTCTCCCCCGGCGGCGCCAGCGCGCTCGACACCGTCTTCTCCCACCTGCCCCCGCCCGTCACCATCCCGCCGCTCGGCTCCAGCGTCTACTACCGCCAGAGCGAGCTCCTCCGCACCTTCGCCGCCACCCACCATCAAGCCAGCGCTGCGGCGGCTACCAGCTCCTCGTCGGGCTACGCTGTTCCTTTTCCTTTTCCTGGTGCGGCGGCGCACGACGCGGCGGCAGCGGCGGCGGCGGGCAGGAAGATGTACCGCGGCGTGCGGCAGCGGCAGTGGGGCAAGTGGGTGGCGGAGATCCGGCTGCCGCAGAACCGGGTGCGCGTCTGGCTCGGCACCTACGACTCTCCCGAGACCGCCGCGCACGCCTACGACCGCGCCGCCTACCGGCTCCGCGGCGAGTACGCGCGCCTCAACTTCCCCGGCGTCATGGACGGCGCCGACGACGCCCGCTTCCCCGACGCCCTCCGCCATCTCCGCGACGCGGTGGACGCCAAGATCCAGGCCATCCGCGTCCGCATGGCCCGCAAGCGCGCGCGCGCCAGGCGGCTGCGCGAGGAGAGCAAGCAGCAGCAGCAGCAGAACAACACCCAACGCGCCGAGGCCGCCCCGGTGGCGCCGACGGCCCCGCGCCCCGTCGTGTCCGAGAGCGCCACGACGTCCGAGACGACGACGACGACGACGTCGTCGTACGGGTCGCCGGAGGGGGTGCTGTCCGCGGCCGACTTCGAGTGCTCGCTGCAGCAGATGCCGTCCTTCGACCCGGAGCTCATCTGGGAGATGCTCAACTTCTAGCCGCCGCCGGCGGCCAGTAGGACTAGACCGATTCGACGGACGCAAAGCCAACCAGCATCAAGGTCCATCCCATCCCATTCATGGCGATGGCGTGGACATGGTTGCGGCGTCTGTCGGAGCCGAAGCTGGTATCGTAGGATTGATTCGATCTCCATCTGTGAGGAGGGAGGATTACAGTTAACCTGTCACTATATGAGCAATAAAATGCCCGGGAAAAATTGGGCGTCTCCTAGAGACTATACTCCTAAATCCCAATTACTACTAGCAACTGCAACTGCATTCTGCATCTGTCGTGTCCTGAGCATTCGCATCACATCACATCGACAAAGTTGTAAAGCTGATCCCTGAATTCATAGCATCTCTAATGTCAAC

>CDS

TGGACGCCAGCCTCCGGACGCTGTCGCCGTCCAGCTTCACCGGGGAGGTGCGCTCCGCGGTGTCCTCCCTCCTGCTCTCCCCCGGCGGCGCCAGCGCGCTCGACACCGTCTTCTCCCACCTGCCCCCGCCCGTCACCATCCCGCCGCTCGGCTCCAGCGTCTACTACCGCCAGAGCGAGCTCCTCCGCACCTTCGCCGCCACCCACCATCAAGCCAGCGCTGCGGCGGCTACCAGCTCCTCGTCGGGCTACGCTGTTCCTTTTCCTTTTCCTGGTGCGGCGGCGCACGACGCGGCGGCAGCGGCGGCGGCGGGCAGGAAGATGTACCGCGGCGTGCGGCAGCGGCAGTGGGGCAAGTGGGTGGCGGAGATCCGGCTGCCGCAGAACCGGGTGCGCGTCTGGCTCGGCACCTACGACTCTCCCGAGACCGCCGCGCACGCCTACGACCGCGCCGCCTACCGGCTCCGCGGCGAGTACGCGCGCCTCAACTTCCCCGGCGTCATGGACGGCGCCGACGACGCCCGCTTCCCCGACGCCCTCCGCCATCTCCGCGACGCGGTGGACGCCAAGATCCAGGCCATCCGCGTCCGCATGGCCCGCAAGCGCGCGCGCGCCAGGCGGCTGCGCGAGGAGAGCAAGCAGCAGCAGCAGCAGAACAACACCCAACGCGCCGAGGCCGCCCCGGTGGCGCCGACGGCCCCGCGCCCCGTCGTGTCCGAGAGCGCCACGACGTCCGAGACGACGACGACGACGACGTCGTCGTACGGGTCGCCGGAGGGGGTGCTGTCCGCGGCCGACTTCGAGTGCTCGCTGCAGCAGATGCCGTCCTTCGACCCGGAGCTCATCTGGGAGATGCTCAACTTCTAG

>DNA

CGGCCTGTTTAGCCCGGCCCATTTGGGCAGCTATATCCAGTGGTGTACAGAATGATCGATGGAATTCTTTGGTGTTTTGACATGGGAGTGAACATGAACCCGTCAACCATGGCGATATCTCTGCGTGGGTGCATCTTCTCCGTTGGGCGGTCGATCACATGAGCGATTACGGGTGTAAACATGCCGAGAAGTTGACCGAGTGGCGTCACATTAACTCCGTGCGTCGCAGCTAAATTGACACACATCACACACACACCCGGTTTGCAGTCTGAACGGCGCCGGGCTCAGTTCATACGTAGGACTCCATTTCATTCCCGGTTGCCAGGGGCGTCATGGTGACCACGGCGCCCGGCGCCCGGCGCCCATGGGTTATATAAAGCCACACGACGCGCACCTGGCTTGGACAGACAGACAAACCTCTCACACTCCACCGCTAATCGCCACCACACCAAGACTCGCCTTTTGCTCCGGCCAAGGATGCCATTCCCGCCCCGCCCGTGAACGCCCGAACCAACTCGCTCCAGCTCCCGTCCATGGACGCCAGCCTCCGGACGCTGTCGCCGTCCAGCTTCACCGGGGAGGTGCGCTCCGCGGTGTCCTCCCTCCTGCTCTCCCCCGGCGGCGCCAGCGCGCTCGACACCGTCTTCTCCCACCTGCCCCCGCCCGTCACCATCCCGCCGCTCGGCTCCAGCGTCTACTACCGCCAGAGCGAGCTCCTCCGCACCTTCGCCGCCACCCACCATCAAGCCAGCGCTGCGGCGGCTACCAGCTCCTCGTCGGGCTACGCTGTTCCTTTTCCTTTTCCTGGTGCGGCGGCGCACGACGCGGCGGCAGCGGCGGCGGCGGGCAGGAAGATGTACCGCGGCGTGCGGCAGCGGCAGTGGGGCAAGTGGGTGGCGGAGATCCGGCTGCCGCAGAACCGGGTGCGCGTCTGGCTCGGCACCTACGACTCTCCCGAGACCGCCGCGCACGCCTACGACCGCGCCGCCTACCGGCTCCGCGGCGAGTACGCGCGCCTCAACTTCCCCGGCGTCATGGACGGCGCCGACGACGCCCGCTTCCCCGACGCCCTCCGCCATCTCCGCGACGCGGTGGACGCCAAGATCCAGGCCATCCGCGTCCGCATGGCCCGCAAGCGCGCGCGCGCCAGGCGGCTGCGCGAGGAGAGCAAGCAGCAGCAGCAGCAGAACAACACCCAACGCGCCGAGGCCGCCCCGGTGGCGCCGACGGCCCCGCGCCCCGTCGTGTCCGAGAGCGCCACGACGTCCGAGACGACGACGACGACGACGTCGTCGTACGGGTCGCCGGAGGGGGTGCTGTCCGCGGCCGACTTCGAGTGCTCGCTGCAGCAGATGCCGTCCTTCGACCCGGAGCTCATCTGGGAGATGCTCAACTTCTAGCCGCCGCCGGCGGCCAGTAGGACTAGACCGATTCGACGGACGCAAAGCCAACCAGCATCAAGGTCCATCCCATCCCATTCATGGCGATGGCGTGGACATGGTTGCGGCGTCTGTCGGAGCCGAAGCTGGTATCGTAGGATTGATTCGATCTCCATCTGTGAGGAGGGAGGATTACAGTTAACCTGTCACTATATGAGCAATAAAATGCCCGGGAAAAATTGGGCGTCTCCTAGAGACTATACTCCTAAATCCCAATTACTACTAGCAACTGCAACTGCATTCTGCATCTGTCGTGTCCTGAGCATTCGCATCACATCACATCGACAAAGTTGTAAAGCTGATCCCTGAATTCATAGCATCTCTAATGTCAAC

>HvDREB6.2

>Protein

MDAADSGGGVRGRERRWKGKAASSAAEKQQQQPLAPVLEDAPAAALLPPLKKMRSPDCRLRRSVSSLSSAPASPDSSSVSNPLSPPATSLPPYASSTRQIFPFAYDPSPAAAPRLLQLLQYSSSLYQQPMLPQQQQHTRSQHPHMISFGDAQQQQQQQFEAAAALVPPQYMSPEALRYWSAALNLSPRGVLGGVVPPALYQHLLRPPGPAKLYRGVRQRHWGKWVAEIRLPRNRTRLWLGTFDTAEDAAMAYDREAFKLRGENARLNFPDLFLGKGRSGGSGRTSASAAASASSSSSKSAPPTPEETHAQQAQLLLQREQKQHMDEQANAVGPKPLLSAAEQDGLPEPEQNPQLQNAEQQCSDGSTAMMQQAPATPGGVWGPADDAWFSAWGPGSSVWDYDMDSAHGLLLQSRFAGEQAGMDYVPSAPEAHMAPAAGTGTACAAPPSPLPPRPPFMWKD

>cDNA

CTCCCCCGCGCACGCGCGCCCCGCAGCTTCTCTCGCAGCCTTGTACGCTTCGTACGTGAGTTGCTTGGCCTGGTTTGGTATTGTCTGCGGCGGGCGGCGGCGGCCGGGTTAAAATTCAGCCGGAGGGTTCGCGCGATTGGGCGAGTTGCTGGAATGGACGCGGCGGATAGTGGTGGCGGCGTCCGCGGGCGCGAGCGGAGATGGAAGGGGAAGGCCGCGAGCTCGGCGGCGGAGAAGCAGCAGCAGCAGCCGCTGGCGCCGGTTTTAGAAGACGCGCCGGCGGCCGCATTGCTCCCGCCGCTGAAGAAGATGCGGAGCCCCGACTGCCGCCTCCGCCGCTCCGTGTCCTCGCTGTCGTCGGCCCCTGCTTCCCCGGACTCCTCCTCTGTTTCCAACCCCCTCTCTCCGCCCGCGACGTCCTTGCCGCCTTATGCGTCGTCGACGCGGCAGATATTCCCGTTCGCGTACGATCCGTCCCCGGCGGCGGCCCCGAGGCTCCTGCAGCTGTTGCAATACTCCTCCAGCTTGTACCAACAGCCGATGCTGCCGCAGCAGCAGCAACACACACGTTCGCAGCATCCGCACATGATATCCTTCGGCGATGCCCAGCAGCAGCAGCAGCAGCAGTTCGAGGCGGCGGCCGCCTTGGTTCCGCCGCAGTACATGTCGCCCGAGGCGCTGCGCTACTGGAGCGCGGCCCTGAACCTGAGCCCGCGTGGCGTGCTCGGCGGGGTCGTGCCGCCGGCGCTGTACCAGCACCTGCTGCGGCCGCCTGGCCCGGCCAAGCTGTACCGCGGCGTGCGCCAGCGTCACTGGGGGAAGTGGGTGGCGGAGATCCGCCTGCCGCGGAACCGAACGCGCCTGTGGCTCGGCACCTTCGACACCGCCGAGGACGCCGCCATGGCGTACGATCGCGAGGCCTTCAAGCTGCGCGGCGAGAACGCGCGGCTCAATTTCCCCGACCTCTTTCTCGGCAAAGGCCGCTCCGGCGGGAGCGGCCGCACCAGCGCCAGCGCCGCGGCGTCGGCCTCCTCCTCCTCCTCCAAGTCCGCTCCGCCGACGCCGGAAGAGACCCATGCGCAGCAAGCTCAGCTGCTGCTCCAGCGTGAACAGAAGCAGCACATGGACGAGCAAGCCAACGCTGTTGGACCGAAACCTCTGCTCTCCGCAGCAGAGCAGGACGGCCTCCCGGAACCAGAGCAAAATCCTCAGCTCCAGAATGCAGAGCAACAATGCAGCGACGGCAGCACGGCCATGATGCAGCAGGCTCCAGCAACCCCCGGCGGCGTCTGGGGCCCCGCCGACGACGCATGGTTCAGCGCGTGGGGTCCGGGCAGCTCTGTCTGGGACTACGACATGGACAGCGCCCATGGCCTCCTACTCCAGTCTCGCTTCGCCGGTGAGCAGGCCGGCATGGACTACGTCCCCAGTGCGCCCGAAGCCCACATGGCACCGGCGGCAGGGACAGGCACGGCCTGTGCCGCTCCCCCTTCTCCCCTTCCTCCCCGTCCTCCCTTCATGTGGAAGGACTAAGACACGCATTCCCATCTCTGAATATCTCACACACAGAAGACAGAACCTACCTACGCTGTGAGATGCACACAGGCTGCAGATTTTTAACAGACAGCTTGTGGCATCATCACATCACAGGAACATCTCATAACCTTTTTCTTTTCTCTCTTGATCGATTTTCTCCTGAAAATCGCATGGAAGGCCCCGGCTGCATTTTATAGGCTGGGACAAAGATGGGCTATATTTGATTCTCCATCCTTGTACATCCTTCCGTGATGATTCTAATGAGATTTAGACATTAGAT

>CDS

ATGGACGCGGCGGATAGTGGTGGCGGCGTCCGCGGGCGCGAGCGGAGATGGAAGGGGAAGGCCGCGAGCTCGGCGGCGGAGAAGCAGCAGCAGCAGCCGCTGGCGCCGGTTTTAGAAGACGCGCCGGCGGCCGCATTGCTCCCGCCGCTGAAGAAGATGCGGAGCCCCGACTGCCGCCTCCGCCGCTCCGTGTCCTCGCTGTCGTCGGCCCCTGCTTCCCCGGACTCCTCCTCTGTTTCCAACCCCCTCTCTCCGCCCGCGACGTCCTTGCCGCCTTATGCGTCGTCGACGCGGCAGATATTCCCGTTCGCGTACGATCCGTCCCCGGCGGCGGCCCCGAGGCTCCTGCAGCTGTTGCAATACTCCTCCAGCTTGTACCAACAGCCGATGCTGCCGCAGCAGCAGCAACACACACGTTCGCAGCATCCGCACATGATATCCTTCGGCGATGCCCAGCAGCAGCAGCAGCAGCAGTTCGAGGCGGCGGCCGCCTTGGTTCCGCCGCAGTACATGTCGCCCGAGGCGCTGCGCTACTGGAGCGCGGCCCTGAACCTGAGCCCGCGTGGCGTGCTCGGCGGGGTCGTGCCGCCGGCGCTGTACCAGCACCTGCTGCGGCCGCCTGGCCCGGCCAAGCTGTACCGCGGCGTGCGCCAGCGTCACTGGGGGAAGTGGGTGGCGGAGATCCGCCTGCCGCGGAACCGAACGCGCCTGTGGCTCGGCACCTTCGACACCGCCGAGGACGCCGCCATGGCGTACGATCGCGAGGCCTTCAAGCTGCGCGGCGAGAACGCGCGGCTCAATTTCCCCGACCTCTTTCTCGGCAAAGGCCGCTCCGGCGGGAGCGGCCGCACCAGCGCCAGCGCCGCGGCGTCGGCCTCCTCCTCCTCCTCCAAGTCCGCTCCGCCGACGCCGGAAGAGACCCATGCGCAGCAAGCTCAGCTGCTGCTCCAGCGTGAACAGAAGCAGCACATGGACGAGCAAGCCAACGCTGTTGGACCGAAACCTCTGCTCTCCGCAGCAGAGCAGGACGGCCTCCCGGAACCAGAGCAAAATCCTCAGCTCCAGAATGCAGAGCAACAATGCAGCGACGGCAGCACGGCCATGATGCAGCAGGCTCCAGCAACCCCCGGCGGCGTCTGGGGCCCCGCCGACGACGCATGGTTCAGCGCGTGGGGTCCGGGCAGCTCTGTCTGGGACTACGACATGGACAGCGCCCATGGCCTCCTACTCCAGTCTCGCTTCGCCGGTGAGCAGGCCGGCATGGACTACGTCCCCAGTGCGCCCGAAGCCCACATGGCACCGGCGGCAGGGACAGGCACGGCCTGTGCCGCTCCCCCTTCTCCCCTTCCTCCCCGTCCTCCCTTCATGTGGAAGGACTAA

>DNA

GTAAGTGAAACAATGTGGCAAATATGAATGAAAAAAAAGTGTCAAAACATGTCAATATGGGATCTAGTTTCGAAGATCTCATCGCGACAAAGACAACTGTGAAAACAGATCCTTGATCAAATTAACCGTTTTAGAGATAAGACTTTTTAANNNTCAAACATTGAAGGGAATCTAGCTGACATTATTACATGCATGCCTGGTAAAGAAAGAAGGTTTGTCGCTGCATGTCCAAACAATTTGAGCACTCTAGCACATTGATCGCATGTGCTTGCATGACTTTTGTAGTGATTAGTAAAAATACTGTAGCACGATAGCGACACATGCAGAAGTATAATACATGCGAGACAGAGTGTCCATGCATTGCACCCGCCGCATGAAAAATCCGCTGTGCGGCGCTTCTAATTAGATTTTCCCTTTATTGAATCAGCCAGCCATGACGTTGGAATATTGGAAGTACTGCCAGCATAACAACTGGAGGTGATAGGCATTCGCCAACTCGAACCCGGTCAGCCCACCAACAAAACCCGATTTTTTCGCAGTCGAGAAGAATAATAACGGGGCCGAAACGAGATTGTAGTGCAAGGAAAGCAACAGACGGATCACTCCAAGGCTTCTTTCCCGGCCAGTGCCGTCCGCGCAAAGCCGCGACACCAAACCGAAAGCTACGTCGGGTCTCCAGCGCATGGCTCGCGATTCTTGCGGCGCTCCTAGGCCGTCGCACGAATCGCGAGGTCCGACGTGGCGCCGGGCGGGGCTCGCTGACGGGTCAATGCGCGTGGGGCCCGACCACCGTCCACGACCCCGTTGGCGCCACACCGCGACAACTGCCTTTCTCGTTTTCGCCCGCGTGGAACATCCCCGAGCTCCCGAAAAATATCTGGCCCGCCCACCTTTATAAGCCCGTCGCGGCCGGCCGATCACTCCAAATCAGATCAGAACCTCGCGCCCCATTGGCTCACCTCCTCCCCCGCGCACGCGCGCCCCGCAGCTTCTCTCGCAGCCTTGTACGCTTCGTACGTGAGTTGCTTGGCCTGGTTTGGTATTGTCTGCGGCGGGCGGCGGCGGCCGGGTTAAAATTCAGCCGGAGGGTTCGCGCGATTGGGCGAGTTGCTGGAATGGACGCGGCGGATAGTGGTGGCGGCGTCCGCGGGCGCGAGCGGAGATGGAAGGGGAAGGCCGCGAGCTCGGCGGCGGAGAAGCAGCAGCAGCAGCCGCTGGCGCCGGTTTTAGAAGACGCGCCGGCGGCCGCATTGCTCCCGCCGCTGAAGAAGATGCGGAGCCCCGACTGCCGCCTCCGCCGCTCCGTGTCCTCGCTGTCGTCGGCCCCTGCTTCCCCGGACTCCTCCTCTGTTTCCAACCCCCTCTCTCCGCCCGCGACGTCCTTGCCGCCTTATGCGTCGTCGACGCGGCAGATATTCCCGTTCGCGTACGATCCGTCCCCGGCGGCGGCCCCGAGGCTCCTGCAGCTGTTGCAATACTCCTCCAGCTTGTACCAACAGCCGATGCTGCCGCAGCAGCAGCAACACACACCTTCGCAGCATCCGCAGATGATATCCTTCGGCGATGCCCAGCAGCAGCAGCAGTTCGAGGCGGCGGCCGCCTTGGTTCCGCCGCAGTACATGTCGCCCGAGGCGCTGCGCTACTGGAGCGCGGCCCTGAACCTGAGCCCGCGAGGCGTGCTCGGCGGGGTCGTGCCGCCGGCGCTGTACCAGCACCTGCTGCGGCCGCCTGGCCCGGCCAAGCTGTACCGCGGCGTGCGCCAGCGTCACTGGGGGAAGTGGGTGGCGGAGATCCGCCTGCCGCGGAACCGAACGCGCCTGTGGCTCGGCACCTTCGACACCGCCGAGGACGCCGCCATGGCGTACGATCGCGAGGCCTTCAAGCTGCGCGGCGAGAACGCGCGGCTCAATTTCCCCGACCTCTTTCTCGGCAAAGGCCGCTCCGGCGGGAGCGGCCGCACCAGCGCCAGCGCCGCGGCGTCGGCCTCCTCCTCCTCCTCCAAGTCCGCTCCGCCGACGCCGGAAGAGACCCATGCGCAGCAAGCTCAGCTGCTGCTCCAGCGTGAACAGAAGCAGCACATGGACGAGCAAGCCAACGCTGTTGGACCGAAACCTCTGCTCTCCGCAGCAGAGCAGGACGGCCTCCCGGAACCAGAGCAAAATCCTCAGCTCCAGAATGCAGAGCAACAATGCAGCGACGGCAGCACGGCCATGATGCAGCAGGCTCCAGCAACCCCCGGCGGCGTCTGGGGCCCCGCCGACGACGCATGGTTCAGCGCGTGGGGTCCGGGCAGCTCTGTCTGGGACTACGACATGGACAGCGCCCATGGCCTCCTACTCCAGTCTCGCTTCGCCGGTGAGCAGGCCGGCATGGACTACGTCCCCAGTGCGCCCGAAGCCCACATGGCACCGGCGGCAGGGACAGGCACGGCCTGTGCCGCTCCCCCTTCTCCCCTTCCTCCCCGTCCTCCCTTCATGTGGAAGGACTAAGACACGCATTCCCATCTCTGAATATCTCACACACAGAAGACAGAACCTACCTACGCTGTGAGATGCACACAGGCTGCAGATTTTTAACAGACAGCTTGTGGCATCATCACATCACAGGAACATCTCATAACCTTTTTCTTTTCTCTCTTGATCGATTTTCTCCTGAAAATCGCATGGAAGGCCCCGGCTGCATTTTATAGGCTGGGACAAAGATGGGCTATATTTGATTCTCCATCCTTGTACATCCTTCCGTGATGATTCTAATGAGATTTAGACATTAGAT

>HvDREB6.3

>Protein

MDASSASGESGSGGGRGTGRRWKGKGVTPSARHIFPFAYEPSATTARESPQLLRQYSSMSQPAASSPQQQPPLRHQQMISFGGSPPCATHSFFMPAESAQQQQHLVRYWSEALNLSPRGGLAGMPPSLYQQLLRAPPPPQKLYRGVRQRHWGKWVAEIRLPRNRTRLWLGTFDTAEDAAMAYDREAFKLRGENARLNFPDRFLGKGRAGGSGRTSAASSAVTSVATGTGTASCSSSSSSPPQTSDEAAANTQQAPRQREQQHAEEWSTLGNQPQHPPPTTIPQDGGSRDAATPYSAEMFHSSAPSGGMWVQADESWFNAWGPGSSFWDYEMDDSARGLFIHHPRFSGDDAGMSHSGAQETPPATAAAGTSDTPCDDVLVTSSAPPPETYQAPNFM

>cDNA

ACCCCACCACGCGCACGCTCGATCGTTAGCTCTCCGACTCCCCGCGCTCCCCCGCGACTACGCCAAGCGATCATTTTCCTAGCGCCGCGAAGTCTTCTCCGCGCGCATGGACGCGTCGAGCGCGAGCGGCGAGAGTGGAAGCGGAGGCGGTCGCGGGACTGGCAGGAGGTGGAAGGGGAAGGGGGTGACGCCGATACAGGCGCGGCGGCAGCAGCTGCTGGCGCCGGTCTTCGAGGACGCGTCCGCGGCATTGCTGCGCCCGCTCAGGAAGATCGGGAGGAGCCCCGACCGCCTCCACCGGACCACGTCGTCGCTCTCCACTTCCTCGTCGTCGGCCCCGGCCTCGCCTCGGTCTTTCCCGGCTTCCGACGCCGCCGCGCCTTCCGCGCGGCACATCTTCCCCTTCGCGTATGAGCCCTCGGCGACGACGGCGCGCGAGAGCCCGCAGCTTCTCCGGCAGTACTCCAGCATGTCCCAACCTGCAGCGTCGTCGCCGCAGCAGCAGCCGCCTTTGCGGCACCAGCAGATGATCTCGTTCGGCGGGTCGCCTCCGTGCGCGACCCATTCGTTCTTCATGCCAGCCGAGAGCGCGCAGCAGCAACAGCATCTGGTGCGGTACTGGAGCGAGGCGCTGAACCTGAGCCCCCGCGGCGGCCTGGCCGGCATGCCGCCTTCGCTGTACCAGCAGCTGCTGCGGGCGCCGCCACCGCCGCAGAAACTGTACCGCGGCGTGCGGCAGCGGCACTGGGGCAAGTGGGTGGCGGAGATCCGCCTGCCACGGAATCGCACGCGGCTGTGGCTCGGCACGTTCGACACCGCCGAGGACGCCGCCATGGCCTACGACCGCGAGGCCTTCAAGCTCCGTGGCGAGAACGCGCGGCTCAACTTCCCCGATCGGTTCCTCGGGAAGGGCCGCGCAGGAGGGAGCGGCCGCACCAGCGCCGCCAGCTCCGCTGTCACCTCAGTCGCCACCGGCACCGGCACCGCTTCCTGCTCGTCGTCATCGTCGTCGCCTCCCCAAACTTCTGACGAGGCGGCGGCCAACACGCAGCAAGCTCCGCGGCAGCGGGAACAGCAGCACGCAGAAGAATGGTCCACGCTTGGAAATCAACCACAGCATCCACCCCCAACGACTATTCCCCAGGACGGCGGCTCTCGCGACGCGGCTACGCCCTACTCGGCCGAAATGTTTCACTCGTCGGCGCCATCCGGCGGCATGTGGGTTCAGGCCGACGAGTCATGGTTCAACGCATGGGGCCCTGGCAGCTCCTTCTGGGACTACGAGATGGACGACAGCGCCCGCGGCCTCTTCATTCACCATCCTCGCTTCTCCGGTGACGATGCCGGCATGTCGCATTCTGGCGCACAAGAAACACCACCGGCGACGGCAGCAGCAGGGACGTCCGACACACCATGCGATGACGTCCTGGTAACCTCTTCAGCTCCTCCTCCAGAAACATACCAGGCTCCAAACTTCATGTGAAAGGTCTAAACGTTCAAAGTTTGAACACCTTGGTGATCGATCCCGAGTAGAATTAGGACAGAGATGCACGCAGAGGTTGCGGTTTTTAAGACAACTTCTCCGGCATCACGCACTTACACATCACAGCATTGTTTCAGATTCAGAAGCCATCTGGCCACACATTTATTTCCCATACTTTTCTTCTCGAAACCACTAGGGAATCCTGTATATTGGCTGCAAATTTTTGGCCAGCTTGACGTATGGGGTGACCTGTCAATCTCCTCCATTTACACATTTTAGCATATGTTGAATGTCTTAGATAGTTGGTGTAGTCTTCGAAGTTTGTTTGCTGCAAACCTCATCGTTTCATACTTCTATGTGATGATGGCTCATATGATTAGGTATCTATGCGTGTACGATTTGTCATTGTACTTTGCTTTGAAACATTCCAGCTATTAATTGTTCAGCTATGCTAAGTTGCTTGTTCATCTTCAACCTTAGGTACGTTTTCTTTGCTTCTG

>CDS

ATGGACGCGTCGAGCGCGAGCGGCGAGAGTGGAAGCGGAGGCGGTCGCGGGACTGGCAGGAGGTGGAAGGGGAAGGGGGTGACGCCGATACAGGCGCGGCGGCAGCAGCTGCTGGCGCCGGTCTTCGAGGACGCGTCCGCGGCATTGCTGCGCCCGCTCAGGAAGATCGGGAGGAGCCCCGACCGCCTCCACCGGACCACGTCGTCGCTCTCCACTTCCTCGTCGTCGGCCCCGGCCTCGCCTCGGTCTTTCCCGGCTTCCGACGCCGCCGCGCCTTCCGCGCGGCACATCTTCCCCTTCGCGTATGAGCCCTCGGCGACGACGGCGCGCGAGAGCCCGCAGCTTCTCCGGCAGTACTCCAGCATGTCCCAACCTGCAGCGTCGTCGCCGCAGCAGCAGCCGCCTTTGCGGCACCAGCAGATGATCTCGTTCGGCGGGTCGCCTCCGTGCGCGACCCATTCGTTCTTCATGCCAGCCGAGAGCGCGCAGCAGCAACAGCATCTGGTGCGGTACTGGAGCGAGGCGCTGAACCTGAGCCCCCGCGGCGGCCTGGCCGGCATGCCGCCTTCGCTGTACCAGCAGCTGCTGCGGGCGCCGCCACCGCCGCAGAAACTGTACCGCGGCGTGCGGCAGCGGCACTGGGGCAAGTGGGTGGCGGAGATCCGCCTGCCACGGAATCGCACGCGGCTGTGGCTCGGCACGTTCGACACCGCCGAGGACGCCGCCATGGCCTACGACCGCGAGGCCTTCAAGCTCCGTGGCGAGAACGCGCGGCTCAACTTCCCCGATCGGTTCCTCGGGAAGGGCCGCGCAGGAGGGAGCGGCCGCACCAGCGCCGCCAGCTCCGCTGTCACCTCAGTCGCCACCGGCACCGGCACCGCTTCCTGCTCGTCGTCATCGTCGTCGCCTCCCCAAACTTCTGACGAGGCGGCGGCCAACACGCAGCAAGCTCCGCGGCAGCGGGAACAGCAGCACGCAGAAGAATGGTCCACGCTTGGAAATCAACCACAGCATCCACCCCCAACGACTATTCCCCAGGACGGCGGCTCTCGCGACGCGGCTACGCCCTACTCGGCCGAAATGTTTCACTCGTCGGCGCCATCCGGCGGCATGTGGGTTCAGGCCGACGAGTCATGGTTCAACGCATGGGGCCCTGGCAGCTCCTTCTGGGACTACGAGATGGACGACAGCGCCCGCGGCCTCTTCATTCACCATCCTCGCTTCTCCGGTGACGATGCCGGCATGTCGCATTCTGGCGCACAAGAAACACCACCGGCGACGGCAGCAGCAGGGACGTCCGACACACCATGCGATGACGTCCTGGTAACCTCTTCAGCTCCTCCTCCAGAAACATACCAGGCTCCAAACTTCATGTGA

>DNA

TGATGCATACAATCATGTACTCCCACCATGTTATATTTGTTGTTAAGTTAAATTTTGTATAGGTTAACAAAATTCACAAAATAAATATTTATAATATCAAATGAGTACGGCATAAAATATATTTCATGGTAAATTTAACCATAGTGATTTGATAGTGTAGATGTTGACAAAATTTTGCATATACTTAGTTGGAGTTTACAAAGTTTGACTTAAGCCAAAACTAATATGGCTGACTCCGTTCTGGTTTATTGGTCCCATTCGTATATTATGCCCAATTTTAACCGTGGATTTGACTAACAAAATATAAGTTGTATGTCATAAAACTTATATTGTTGGATTCATATTTGAAAGAGGTTTTCAATTTTAGTATTTTTGTGGTACATAACTTTTTAAGATAAATCAAAGGTCAAATTTTAACCCAAAATATAAGGAGGACTAATAAATCCGGACGGAGGTAGTATGCACTAAATTTTGACATAGATGGAGTATTATTTATCATGAAAAATGATAACAAGCATTGGTATTGGATAAATTGGCTTACCAAAGAGGGGGGAAAATGCGGCTATCCACAATGAGATCAGAAAAGAGCCATTTCCCAACCAAATAAAACAGAAAAGCATGAGGTTCGAAGCCCTTTCTCATTTCCAATTAGTATCGGTAATGCCCCGTCTAAAATTATTTCATCACAATCACACGTTAGCTTGGTTGGAAAAGTTCGTTTGCACCCACTAACTAATAACCTGTCTCATTGCAACGTCTCCACAAAACCCTTGACATGGACTATAAGCTAACAACAGGTGCATGACTTAAGACAGTGCCTTCAAGAAGACATCGCCTCTCGCACGCCGAAGTCATGGGGGTTTCATCCCTAGAGAATTCAACCACGATATTAACCGCCAATGGACTGATCCACTTCTTCGCGATCTACTCACATATAACTTGCCAGCAAAAGAAGAGATCACCGGAAGATCTAATCACTGTCAACTACCATCTTGCCAATGATTCTTATGCACGTCATGGTAGCACATAATATCGTCTGCCACCACATACATCGCCCCGAATGTCATGCCCCGTAAGGGTCTAGATCCGACCTCGGCGGCACCCCTCGCCCCTCCCATGGTGGTGAGGGAGAGTAGCACCGCTTCACCCATCGGCTCCAACCTACCTGCGGCATTGAGCTACCGTCAGGCGCACCTCCGCACTAGCAGCTGCAAAGTGCACCCGATCCTAATAGGCGCGCATGGCATCGGATCCGTGTGACCTCACCACCACACCATCGAGCAAACCAGCGTCGCTCCTAATGACCGAGGCGACCACCGTATCAACAAAAGAGGAAATCGCGCGCCGAAACCTTCAATGTATGGCCCACTGTCATAACGGTGTTGGCCGGTGGTGGCGGCAATAATGGTAAACATTCAAAGATTTACATGCTTTTACACGCTAACTAGGATCTTTTCAATCTAACTAATCTCTCTCCTCCCTGATTTTTACGGTGGGCTCCACCTCCCCGTTAATCTCCAATCAAGATTTATCTTTCTATAAAAGTTCGTATTTTTTAATACGTGTAGCATTACTGATGGTAACAATGACGAGGAAGAGAAGAAGAGGGCTTGTGGCGCTAGGGTTCTTCCTTGTATCGCCTCGGAGCGAAACGAGGGGGTCGAGTCGAGCTTTGCTCTACACGGGAGTGGAGTTACTTTTTCTTTAGAACATGGAGTGGAGTTCCTTGGCGAGTCCATGCTCGTGGGGTCCTTACCACCGACCACTATCTTTTGCCCCTGGCGCGCCGCGCAGCACCATGATCCCATTGCAACGAAGCATTTCACTTTTCCCTACCCTACCCTACCCTTATAAGCTTCGGTCCGGTCGCACTCGTCTCGGAATCAGAACCAAAACCCCACCACGCGCACGCTCGATCGTTAGCTCTCCGACTCCCCGCGCTCCCCCGCGACTACGCCAAGCGATCATTTTCCTAGCGCCGCGAAGTCTTCTCCGCGCGCATGGACGCGTCGAGCGCGAGCGGCGAGAGTGGAAGCGGAGGCGGTCGCGGGACTGGCAGGAGGTGGAAGGGGAAGGGGGTGACGCCGATACAGGCGCGGCGGCAGCAGCTGCTGGCGCCGGTCTTCGAGGACGCGTCCGCGGCATTGCTGCGCCCGCTCAGGAAGATCGGGAGGAGCCCCGACCGCCTCCACCGGACCACGTCGTCGCTCTCCACTTCCTCGTCGTCGGCCCCGGCCTCGCCTCGGTCTTTCCCGGCTTCCGACGCCGCCGCGCCTTCCGCGCGGCACATCTTCCCCTTCGCGTATGAGCCCTCGGCGACGACGGCGCGCGAGAGCCCGCAGCTTCTCCGGCAGTACTCCAGCATGTCCCAACCTGCAGCGTCGTCGCCGCAGCAGCAGCCGCCTTTGCGGCACCAGCAGATGATCTCGTTCGGCGGGTCGCCTCCGTGCGCGACCCATTCGTTCTTCATGCCAGCCGAGAGCGCGCAGCAGCAACAGCATCTGGTGCGGTACTGGAGCGAGGCGCTGAACCTGAGCCCCCGCGGCGGCCTGGCCGGCATGCCGCCTTCGCTGTACCAGCAGCTGCTGCGGGCGCCGCCACCGCCGCAGAAACTGTACCGCGGCGTGCGGCAGCGGCACTGGGGCAAGTGGGTGGCGGAGATCCGCCTGCCACGGAATCGCACGCGGCTGTGGCTCGGCACGTTCGACACCGCCGAGGACGCCGCCATGGCCTACGACCGCGAGGCCTTCAAGCTCCGTGGCGAGAACGCGCGGCTCAACTTCCCCGATCGGTTCCTCGGGAAGGGCCGCGCAGGAGGGAGCGGCCGCACCAGCGCCGCCAGCTCCGCTGTCACCTCAGTCGCCACCGGCACCGGCACCGCTTCCTGCTCGTCGTCATCGTCGTCGCCTCCCCAAACTTCTGACGAGGCGGCGGCCAACACGCAGCAAGCTCCGCGGCAGCGGGAACAGCAGCACGCAGAAGAATGGTCCACGCTTGGAAATCAACCACAGCATCCACCCCCAACGACTATTCCCCAGGACGGCGGCTCTCGCGACGCGGCTACGCCCTACTCGGCCGAAATGTTTCACTCGTCGGCGCCATCCGGCGGCATGTGGGTTCAGGCCGACGAGTCATGGTTCAACGCATGGGGCCCTGGCAGCTCCTTCTGGGACTACGAGATGGACGACAGCGCCCGCGGCCTCTTCATTCACCATCCTCGCTTCTCCGGTGACGATGCCGGCATGTCGCATTCTGGCGCACAAGAAACACCACCGGCGACGGCAGCAGCAGGGACGTCCGACACACCATGCGATGACGTCCTGGTAACCTCTTCAGCTCCTCCTCCAGAAACATACCAGGCTCCAAACTTCATGTGA

>HvDREB6.4

>Protein

MAAIDLYGAANQLSSSSSSSSSDQELMRALEPFIRSASSPTSSTSTSTSPFSYHAASTSTSTSSPFSYYSCYSTQPQESCYLPASSSYSYTTLQAPFAPATSSFSQLPPLPPTSQYSTSPSATYQSPSAGDAVGLASLGPEQIHQIQAQLYLQQQQQQQQRGLSASFLGPRAQPMKQAGAPSASAAGKLYRGVRQRHWGKWVAEIRLPKNRTRLWLGTFDTAEDAALAYDKAAFRLRGDAARLNFPNLRRGGAHLAGPLHASVNAKLDSICQNIATAPSSKSAPPDSPKASTSTTSTEGDYGSVLSAGSTPLPPPPQSSQHQHQQPAAPLHEMANLDFSEAPWDESDAFHLHQDLHKCPSWEIDWDSILS

>cDNA

GAGGAGAGACACGCCACCCGCCCCCACCCCACACCACACCATCACCACCGTCACCAAGCACGCACGCCCTCCCACCCACGACTCACGACCACGACCAGACACCCCACCGCTTCCCCGGCGAGGCTGAGCGATCTTTCCCCAAACCCAAACACAGCAAGCACGACGGAGCACCCACGCCACCCCGGCGGCGAACGCAGGTAAGATCTCCTCCCCGACCTCCCCTCCCCCGGGGCCTCTCTTGGTTGCTATTAGATCAGGCAAGAAGGGGTTCCTGGAAACCATTTTTTCTTCATCATCTTCTTCTTCTTCCCTCGCTTTGCTCGCATGCAAAGTTTTTTATCTTCTTGTTCTTGCTAGGACGAGCGGAGATCCGGAGAACCTTTTTTCTCCTCTGGATTCTCGGTGCGTCTTCTTCTTCTTTTTAGCCTTTCTTGCGGGCCGGCAGAGGGGCTGTTTTTACAGAGCCCCTTCAGGTTGCTCGCACAATTGAATTTGATCTCGATCTCTCCCCTGTTCTTCTTCGCTTTTATTGAAGAAAACAGGGCTTTATTATCCAAGATTCTTTCGGTTTCGTTTCTACCTTTTGGTTTTAGTTCGGTTTCTGCACAAAGGGTTTCGGTTTCTTTTTTCATCTCTTTCTTGGTTGGCAAAAATGGCGGCCATAGATCTGTATGGCGCCGCAAACCAGCTCAGCTCCTCCTCGTCGTCGTCCTCCTCGGACCAGGAGCTCATGAGAGCGCTCGAACCTTTTATCAGGAGCGCTTCCTCGCCCACCTCCTCCACCTCCACCTCCACCTCGCCATTTTCATACCACGCCGCCTCCACCTCCACCTCCACCTCGTCGCCATTTTCGTATTACAGCTGCTACTCTACGCAGCCTCAAGAATCCTGCTACCTCCCTGCCTCCTCCTCTTACAGTTACACCACGCTTCAAGCTCCGTTTGCTCCCGCCACCTCGTCCTTCTCGCAGCTCCCGCCTCTGCCGCCGACCTCGCAGTACAGCACCTCGCCGTCGGCCACGTACCAGTCGCCGTCGGCGGGTGACGCGGTGGGGCTGGCCAGCCTGGGCCCAGAACAGATCCATCAGATCCAGGCGCAGCTCTACCTCCAGCAGCAGCAGCAGCAGCAGCAGAGGGGCCTGTCGGCGTCGTTCCTCGGCCCGCGGGCGCAGCCCATGAAGCAGGCCGGGGCGCCGTCGGCTTCGGCCGCCGGGAAGCTGTACCGCGGCGTGCGGCAGCGCCACTGGGGCAAGTGGGTGGCGGAGATCCGCCTCCCCAAGAACCGGACGCGGCTGTGGCTCGGCACCTTCGACACCGCCGAGGACGCGGCGCTCGCCTACGACAAGGCGGCCTTCCGCCTCCGCGGAGACGCGGCGCGCCTCAACTTCCCCAACCTCCGCCGCGGCGGCGCGCACCTCGCCGGCCCGCTCCACGCCTCCGTCAACGCCAAGCTCGACTCCATCTGCCAGAACATCGCCACCGCACCGTCGTCCAAGTCGGCTCCGCCGGACTCGCCCAAGGCCTCCACGTCCACGACGTCGACGGAGGGGGACTACGGGTCGGTGCTCTCCGCCGGCTCGACGCCCCTCCCTCCGCCGCCGCAGTCGTCGCAGCACCAGCACCAGCAGCCCGCGGCGCCGCTCCACGAGATGGCCAACCTGGACTTCTCGGAGGCGCCGTGGGAYGAGTCGGACGCCTTCCACCTCCACCAGGACCTCCACAAGTGCCCGTCATGGGAGATCGACTGGGACTCCATCCTCTCGTGATCGATCGTACGCCAGAGCAAACGGAAGCTCCATTAATCAATTGTTAAGCAGTGTAATTAATCCCGCAGCGGCCAAGGCTCGATGGAATATTAGACATCGGCCGCCGCCGCCGGAGTTAGGGGTTAAGGAGTTCGCCATCGTCGTCATAGGAGCAGCAGTCGTTTATACAGGCTTAAGACTAAGATTATTTAGCTCACAAACTACCTGTAA

>CDS

ATGGCGGCCATAGATCTGTATGGCGCCGCAAACCAGCTCAGCTCCTCCTCGTCGTCGTCCTCCTCGGACCAGGAGCTCATGAGAGCGCTCGAACCTTTTATCAGGAGCGCTTCCTCGCCCACCTCCTCCACCTCCACCTCCACCTCGCCATTTTCATACCACGCCGCCTCCACCTCCACCTCCACCTCGTCGCCATTTTCGTATTACAGCTGCTACTCTACGCAGCCTCAAGAATCCTGCTACCTCCCTGCCTCCTCCTCTTACAGTTACACCACGCTTCAAGCTCCGTTTGCTCCCGCCACCTCGTCCTTCTCGCAGCTCCCGCCTCTGCCGCCGACCTCGCAGTACAGCACCTCGCCGTCGGCCACGTACCAGTCGCCGTCGGCGGGTGACGCGGTGGGGCTGGCCAGCCTGGGCCCAGAACAGATCCATCAGATCCAGGCGCAGCTCTACCTCCAGCAGCAGCAGCAGCAGCAGCAGAGGGGCCTGTCGGCGTCGTTCCTCGGCCCGCGGGCGCAGCCCATGAAGCAGGCCGGGGCGCCGTCGGCTTCGGCCGCCGGGAAGCTGTACCGCGGCGTGCGGCAGCGCCACTGGGGCAAGTGGGTGGCGGAGATCCGCCTCCCCAAGAACCGGACGCGGCTGTGGCTCGGCACCTTCGACACCGCCGAGGACGCGGCGCTCGCCTACGACAAGGCGGCCTTCCGCCTCCGCGGAGACGCGGCGCGCCTCAACTTCCCCAACCTCCGCCGCGGCGGCGCGCACCTCGCCGGCCCGCTCCACGCCTCCGTCAACGCCAAGCTCGACTCCATCTGCCAGAACATCGCCACCGCACCGTCGTCCAAGTCGGCTCCGCCGGACTCGCCCAAGGCCTCCACGTCCACGACGTCGACGGAGGGGGACTACGGGTCGGTGCTCTCCGCCGGCTCGACGCCCCTCCCTCCGCCGCCGCAGTCGTCGCAGCACCAGCACCAGCAGCCCGCGGCGCCGCTCCACGAGATGGCCAACCTGGACTTCTCGGAGGCGCCGTGGGACGAGTCGGACGCCTTCCACCTCCACCAGGACCTCCACAAGTGCCCGTCATGGGAGATCGACTGGGACTCCATCCTCTCGTGA

>DNA

GAGGAGAGACACGCCACCCGCCCCCACCCCACACCACACCATCACCACCGTCACCAAGCACGCACGCCCTCCCACCCACGACTCACGACCACGACCAGACACCCCACCGCTTCCCCGGCGAGGCTGAGCGATCTTTCCCCAAACCCAAACACAGCAAGCACGACGGAGCACCCACGCCACCCCGGCGGCGAACGCAGGTAAGATCTCCTCCCCGACCTCCCCTCCCCCGGGGCCTCTCTTGGTTGCTATTAGATCAGGCAAGAAGGGGTTCCTGGAAACCATTTTTTCTTCATCATCTTCTTCTTCTTCCCTCGCTTTGCTCGCATGCAAAGTTTTTTATCTTCTTGTTCTTGCTAGGACGAGCGGAGATCCGGAGAACCTTTTTTCTCCTCTGGATTCTCGGTGCGTCTTCTTCTTCTTTTTAGCCTTTCTTGCGGGCCGGCAGAGGGGCTGTTTTTACAGAGCCCCTTCAGGTTGCTCGCACAATTGAATTTGATCTCGATCTCTCCCCTGTTCTTCTTCGCTTTTATTGAAGAAAACAGGGCTTTATTATCCAAGATTCTTTCGGTTTCGTTTCTACCTTTTGGTTTTAGTTCGGTTTCTGCACAAAGGGTTTCGGTTTCTTTTTTCATCTCTTTCTTGGTTGGCAAAAATGGCGGCCATAGATCTGTATGGCGCCGCAAACCAGCTCAGCTCCTCCTCGTCGTCGTCCTCCTCGGACCAGGAGCTCATGAGAGCGCTCGAACCTTTTATCAGGAGCGCTTCCTCGCCCACCTCCTCCACCTCCACCTCCACCTCGCCATTTTCATACCACGCCGCCTCCACCTCCACCTCCACCTCGTCGCCATTTTCGTATTACAGCTGCTACTCTACGCAGCCTCAAGAATCCTGCTACCTCCCTGCCTCCTCCTCTTACAGTTACACCACGCTTCAAGCTCCGTTTGCTCCCGCCACCTCGTCCTTCTCGCAGCTCCCGCCTCTGCCGCCGACCTCGCAGTACAGCACCTCGCCGTCGGCCACGTACCAGTCGCCGTCGGCGGGTGACGCGGTGGGGCTGGCCAGCCTGGGCCCAGAACAGATCCATCAGATCCAGGCGCAGCTCTACCTCCAGCAGCAGCAGCAGCAGCAGCAGAGGGGCCTGTCGGCGTCGTTCCTCGGCCCGCGGGCGCAGCCCATGAAGCAGGCCGGGGCGCCGTCGGCTTCGGCCGCCGGGAAGCTGTACCGCGGCGTGCGGCAGCGCCACTGGGGCAAGTGGGTGGCGGAGATCCGCCTCCCCAAGAACCGGACGCGGCTGTGGCTCGGCACCTTCGACACCGCCGAGGACGCGGCGCTCGCCTACGACAAGGCGGCCTTCCGCCTCCGCGGAGACGCGGCGCGCCTCAACTTCCCCAACCTCCGCCGCGGCGGCGCGCACCTCGCCGGCCCGCTCCACGCCTCCGTCAACGCCAAGCTCGACTCCATCTGCCAGAACATCGCCACCGCACCGTCGTCCAAGTCGGCTCCGCCGGACTCGCCCAAGGCCTCCACGTCCACGACGTCGACGGAGGGGGACTACGGGTCGGTGCTCTCCGCCGGCTCGACGCCCCTCCCTCCGCCGCCGCAGTCGTCGCAGCACCAGCACCAGCAGCCCGCGGCGCCGCTCCACGAGATGGCCAACCTGGACTTCTCGGAGGCGCCGTGGGACGAGTCGGACGCCTTCCACCTCCACCAGGACCTCCACAAGTGCCCGTCATGGGAGATCGACTGGGACTCCATCCTCTCGTGATCGATCGTACGCCAGAGCAAACGGAAGCTCCATTAATCAATTGTTAAGCAGTGTAATTAATCCCGCAGCGGCCAAGGCTCGATGGAATATTAGACATCGGCCGCCGCCGCCGGAGTTAGGGGTTAAGGAGTTCGCCATCGTCGTCATAGGAGCAGCAGTCGTTTATACAGGCTTAAGACTAAGATTATTTAGCTCACAAACTACCTGTAA

>HvDREB6.5

>Protein

MAAAIDMYKYNPATHQIGSASDQELMKALEPFITGASSSSYPYPYQYQYYSSPSMTQDSYTATPSSSSSYASFAAPPLPTTAPFSQLPPLYSSSSQYAVNNGSMGLAQLGPAQIQQIQAQFFVQQQQQRGLAGGSFLGPRAQPMKQSGSPPPRASAAALALAGVAPAQSKLYRGVRQRHWGKWVAEIRLPKNRTRLWLGTFDTAEDAALRYDKAVFRLRGDLARLNFPSLRRGGAHLAGPLHASVDAKLTAICQSLAAPSSKNSAEAEPEPESPKCSASTEGEDSVSAGSPPPVPEMEKLDFTEAPWDESETFHLRKYPSVEIDWDSILS

>cDNA

ATGGCCGCAGCCATAGACATGTACAAGTACAACCCCGCCACGCACCAGATCGGCTCTGCCTCGGATCAGGAGCTCATGAAAGCACTCGAACCTTTTATCACGGGTGCTTCCTCCTCTTCCTACCCCTACCCCTACCAGTACCAGTATTACTCTTCTCCTTCCATGACCCAAGATTCATACACGGCCACCCCATCATCGTCGTCATCCTACGCCTCGTTCGCAGCCCCTCCTCTACCCACCACCGCGCCCTTCTCGCAGCTTCCGCCGCTCTACTCCTCCTCTTCGCAGTATGCCGTGAACAACGGATCCATGGGGCTGGCCCAGCTCGGCCCGGCCCAGATCCAGCAGATCCAGGCCCAGTTCTTCGTCCAGCAGCAGCAGCAGAGGGGCCTGGCTGGCGGCTCCTTCCTTGGGCCGCGCGCGCAGCCGATGAAGCAGTCCGGGTCGCCGCCGCCGCGCGCGTCCGCCGCGGCGCTGGCGCTGGCCGGAGTGGCGCCCGCGCAGTCCAAGCTGTACCGCGGAGTGCGGCAGCGCCACTGGGGCAAGTGGGTGGCGGAGATCCGCCTCCCCAAGAACCGTACGAGGCTGTGGCTCGGCACCTTCGACACCGCCGAGGACGCCGCGCTTCGCTACGACAAGGCCGTCTTCCGCCTCCGCGGCGACCTGGCCCGCCTCAACTTCCCGTCGCTCCGACGCGGCGGCGCCCACCTGGCCGGCCCGCTCCACGCCTCCGTCGACGCCAAGCTCACCGCCATCTGCCAGTCGCTCGCCGCGCCCTCGTCCAAGAACTCCGCCGAGGCGGAGCCGGAGCCGGAGTCCCCCAAGTGCTCCGCGTCCACGGAGGGAGAGGACTCGGTGTCCGCCGGGTCCCCGCCGCCGGTCCCGGAGATGGAGAAGCTGGACTTCACGGAGGCGCCGTGGGACGAGTCGGAGACCTTCCACCTGCGCAAGTACCCGTCCGTGGAGATCGACTGGGACTCCATCCTGTCGTGAACAGCAAGCAGCTACTACCACTACCAGTACAGTCTTCGTTAAGCTCCGTAGCTATGATGTAATTTCTCCTTGGATCGAATCGGCGGCTGCTCTGGCCCGACGGCATTTTAGACGTCGGCCATGGCTGCTGCGAGTAGCTAGCAGTAGCAGTAGCAGTAAGAAGCCAGTGGTGTTTAGTGCTGTAGTAAGGTCGTCGCTACTACGTGGTGTAACTGATCTCCTGGTTGACCCGCCGGCAGTTTTTTCACGGCAAAGGCGGCCAGTCGAGAGGTGTAATCGTGTTTACCCGTTGGAAATTGCACTAGCTTAATT

>CDS

ATGGCCGCAGCCATAGACATGTACAAGTACAACCCCGCCACGCACCAGATCGGCTCTGCCTCGGATCAGGAGCTCATGAAAGCACTCGAACCTTTTATCACGGGTGCTTCCTCCTCTTCCTACCCCTACCCCTACCAGTACCAGTATTACTCTTCTCCTTCCATGACCCAAGATTCATACACGGCCACCCCATCATCGTCGTCATCCTACGCCTCGTTCGCAGCCCCTCCTCTACCCACCACCGCGCCCTTCTCGCAGCTTCCGCCGCTCTACTCCTCCTCTTCGCAGTATGCCGTGAACAACGGATCCATGGGGCTGGCCCAGCTCGGCCCGGCCCAGATCCAGCAGATCCAGGCCCAGTTCTTCGTCCAGCAGCAGCAGCAGAGGGGCCTGGCTGGCGGCTCCTTCCTTGGGCCGCGCGCGCAGCCGATGAAGCAGTCCGGGTCGCCGCCGCCGCGCGCGTCCGCCGCGGCGCTGGCGCTGGCCGGAGTGGCGCCCGCGCAGTCCAAGCTGTACCGCGGAGTGCGGCAGCGCCACTGGGGCAAGTGGGTGGCGGAGATCCGCCTCCCCAAGAACCGTACGAGGCTGTGGCTCGGCACCTTCGACACCGCCGAGGACGCCGCGCTTCGCTACGACAAGGCCGTCTTCCGCCTCCGCGGCGACCTGGCCCGCCTCAACTTCCCGTCGCTCCGACGCGGCGGCGCCCACCTGGCCGGCCCGCTCCACGCCTCCGTCGACGCCAAGCTCACCGCCATCTGCCAGTCGCTCGCCGCGCCCTCGTCCAAGAACTCCGCCGAGGCGGAGCCGGAGCCGGAGTCCCCCAAGTGCTCCGCGTCCACGGAGGGAGAGGACTCGGTGTCCGCCGGGTCCCCGCCGCCGGTCCCGGAGATGGAGAAGCTGGACTTCACGGAGGCGCCGTGGGACGAGTCGGAGACCTTCCACCTGCGCAAGTACCCGTCCGTGGAGATCGACTGGGACTCCATCCTGTCGTGA

>DNA

ATTAGTAATATATAACACTATATGGGATAGGAAACAAAAATGGCAATACGGTATTACTGGGATAAGTCGGATCGGCATCGTTGATCACCTGATCTAGCGCTCGCCGATGATGAGATGTAGCTTGGCGTGGCTTGTGACCGGAAATTCTTGGTGATGTGTGTATGCATGCAACGCCTTCAAATTAGAAAACAGAATCGGTGTAGTTCGTCGCGTCGCAACTTGCAAACATGCGTCAGGGCCATCCGTCCGCCCACCATGGCCACATCCACTTTGACCCCAGCATGGACGCCACCCGCCGTCTTGCCGTTGTCGCCGGCCGCGTCGATCTAGGAGGAAGAAAATGCAACGAAGAGAAAAAGGAACAGACTAACTGCCCGGTTTTTCTGGTCAAGAAATAGCGGAGTTTGAGGAATGGCGGAACATATCACGATCATAGAAGAGTTACATGCTAATTTTCAGGACGTATTTTTTTTTGGATAAATTGTATCTGGCTAGGGAACACGTATTTTCTCAAAAGGGAAGGTTAGGAAACACGTACAGAACGAGGAACAAACGAGTACTACAGCCGTCAACATTTTGGGCCTCTCGGGGCCCCTCGATTTTGGGGGCCCGGGGCGGTCGCCCCCCTGCCCCCCTCAGGGTCGACCCTGATTGGAGTCGAGCTCTCGCTGTCTCGCGTGTCCTACTCTTTCTCGTTGGAGCCCGCAACCCGGACGGCGCCGATGAATGTGAGACCGAATGGATCCCTCCCGGGAGGAGCTGACGTCCACTACGATGAGGCGCCCGCACTGGTTCACCATACGGGACGCCGGAGAATGCAACTCCGCCTCATCAACGTCCACCGCCGCAAGGCATGCCACTGCCTCCCGTGCTTGCTGCCTCGCATGGCGGGCACACCGTGCCATGTTTGTGTGTGAGGACGCCCATGTTGCGTCCTCCAGCTCGGCATAATAATGGATGGGTTGTGCGTCCGCCCCCGTTGTCGGATGCGAGACAGACATCACCAAATATGGTGAGGGGACGACAGCACCGCTACCGGCGGATTCTCGACCAAAGGAGTCTATGCACTGCCGTCGGGCGGCCTCCTCCCGAGAGCGGTGGAGCGCAAGCCGAACGATCATCTCCTCCTCGGAGCCCCGTGAGATGAGCTCATGGTGGACGAATGTGGAGGTGTAGGACTCGGATCCACTAGCCGCCATGCCGAAGAAGGCCAGAGATCGCCGAACGAGAGCTTGGGTGGCGAAGTGATGGAGGGGACTGAAGTGGCTAGGGTTTGGTCCGTACAGAGGATCGAGATGAATATATATGAGGTCGGATGGGCCAGCAGGGGCCGGACTCGTCGTGGCGGACGCGCGTGGTCCTCCCCATATCCGCCTCATATTTGGGCTGGATATAAGGGGTGTCGGTCAGCCTGACTGTTTGAGGCGTATGTCTCGATCATATTTTGCGAATGGTCACTGATTAGGCTCTCCGTCTGGACGTTTTAGGCGGGTTTAGGACGTCCGATTATAGATGCTGTAACGAGGAATTTTGTTTTTGTTTGCCATTTTTTTTTTGAAATATAGAACGACATTCGTGGGAAACGTGGGAAAAAATGATGCTCCAAAATATGTTTGAAAATAACATCTTGGAGCATCGATATTTTTGTTTGCCATATTTTTCACGATTAACAACTGGTGATAAAATTTTGCAAGCAATTGAAACATTTATCAATATTTCACACATAAAAGAATCATTTCTTGTGTTTTTTTTTTTTGATTTTCCTGTTTGCTCGAGCTCATATGAGCTCGGGATAAGAACAACCGTGTCTAAACAAACAGACTAAACAGAAGATTACGAAACTAATGTAGAAAAAGATATGTACATGTGAAAAATGATTTAGCACCTTATTTTAAGCAGTGGGTTCGGAGCTCTTGGCGCAGCTCAGAAGCCGAATTCCGGAGCAGAGCACAAAGGGCAGGGGCAGCACATTGTGAGGTCAAACGAGCGCACCACCACCCAATGAGACGAGGCCACTTGCACAGAACTACAGCGTGGAGATGATCACCCGCGTGTCCGGAGCACAAGTGGCGGATCCTCATTGGCCGCAACTTGCGGTTCCCGCGTGCTGCCCGGCGCGTGCTGACGCCATGGGCGAAAGCAACGTCTCATTTTTCAAAACCGCAGTGCAGCTCCCAACAGAACAAGAAACGATTAACGAAATTAACCCCAATCACATCGCTTTGTGGCAGCAAATTGCACGGCGGGTGGGCGTTGCTTGGCTTGGCGTGCACTGCCGGCGGGAGCCTGGCGCTATAAAACGCCCGGGCGCGACGCCCCTTCTTCCTCAGAGAGACGCCTCAACCCCCACCACCCACCGACTGCAAACGCGCAGCCAGCCACATCCCATCCCATTCCACTCCACGGTGAGGTTGAGCGATCTGACGAGCCCTTTGAGCAGCGACGACGACGGCAGGTTTGTTCTCTGAACTTCTCTGGTCTTTTTAGCAGCAGCCAGCAGGACCACGCGAGAAACAGAGGCCCTGCTCAAGGGGTCCTTCTTCTGGGATTATTAGATCGGAAGAAGCGCTCTCCTCTTGCTCGGGGCTCCTCTTGATTTCTTGGCGACCTCACCACTCTCCTAGAGGAACAGAGATCCAGAGAACCTTTTTTCTCTCCTGGATTCTTGCCTTTCTTTTTCCTTTTCTTTCTGCCTTCCTTAATTTGCCTCGGCACGGCCGGAAAGGGTTCTTTTGCAATACCCTTCGAGTTCGTCCGGATTTATTCCCTGTTTTCCCCTCGTTTCTCTAATTTTATTGAGGAACAGGGCCCTTTTTTTTTCCCTTCCTTTCTGCTTGCACGAGAAAGCTCCTTTTTTTCCGTCCCGTTTTTGGTTCGCCCTTCTCTGCATCACCATCTTCTTCTTCCTCTGTCCAAAGCCCCCATGGCCGCAGCCATAGACATGTACAAGTACAACCCCGCCACGCACCAGATCGGCTCTGCCTCGGATCAGGAGCTCATGAAAGCACTCGAACCTTTTATCACGGGTGCTTCCTCCTCTTCCTACCCCTACCCCTACCAGTACCAGTATTACTCTTCTCCTTCCATGACCCAAGATTCATACACGGCCACCCCATCATCGTCGTCATCCTACGCCTCGTTCGCAGCCCCTCCTCTACCCACCACCGCGCCCTTCTCGCAGCTTCCGCCGCTCTACTCCTCCTCTTCGCAGTATGCCGTGAACAACGGATCCATGGGGCTGGCCCAGCTCGGCCCGGCCCAGATCCAGCAGATCCAGGCCCAGTTCTTCGTCCAGCAGCAGCAGCAGAGGGGCCTGGCTGGCGGCTCCTTCCTTGGGCCGCGCGCGCAGCCGATGAAGCAGTCCGGGTCGCCGCCGCCGCGCGCGTCCGCCGCGGCGCTGGCGCTGGCCGGAGTGGCGCCCGCGCAGTCCAAGCTGTACCGCGGAGTGCGGCAGCGCCACTGGGGCAAGTGGGTGGCGGAGATCCGCCTCCCCAAGAACCGTACGAGGCTGTGGCTCGGCACCTTCGACACCGCCGAGGACGCCGCGCTTCGCTACGACAAGGCCGTCTTCCGCCTCCGCGGCGACCTGGCCCGCCTCAACTTCCCGTCGCTCCGACGCGGCGGCGCCCACCTGGCCGGCCCGCTCCACGCCTCCGTCGACGCCAAGCTCACCGCCATCTGCCAGTCGCTCGCCGCGCCCTCGTCCAAGAACTCCGCCGAGGCGGAGCCGGAGCCGGAGTCCCCCAAGTGCTCCGCGTCCACGGAGGGAGAGGACTCGGTGTCCGCCGGGTCCCCGCCGCCGGTCCCGGAGATGGAGAAGCTGGACTTCACGGAGGCGCCGTGGGACGAGTCGGAGACCTTCCACCTGCGCAAGTACCCGTCCGTGGAGATCGACTGGGACTCCATCCTGTCGTGAACAGCAAGCAGCTACTACCACTACCAGTACAGTCTTCGTTAAGCTCCGTAGCTATGATGTAATTTCTCCTTGGATCGAATCGGCGGCTGCTCTGGCCCGACGGCATTTTAGACGTCGGCCATGGCTGCTGCGAGTAGCTAGCAGTAGCAGTAGCAGTAAGAAGCCAGTGGTGTTTAGTGCTGTAGTAAGGTCGTCGCTACTACGTGGTGTAACTGATCTCCTGGTTGACCCGCCGGCAGTTTTTTCACGGCAAAGGCGGCCAGTCGAGAGGTGTAATCGTGTTTACCCGTTGGAAATTGCACTAGCTTAATT

>HvDREB6.6

>Protein

MYRMAESRDQVMHAFAPPAAHGAAPTISFSFPCPGAEQGGGAGLLRGASYLTPAQILQLQSQLHHARRAPPGAAMAMAAVGQPMKRHGVAALPARPAAKLYRGVRQRHWGKWVAEIRLPRNRTRLWLGTFDAAEEAALAYDAAAFRLRGESARLNFPELRRGGQHHGPPLDAAIDAKLHSICHGEDPPQSQSNATPTPTSFPDVKSEPVCSVSESSSSADGEVSSCSDDVPEMQLLDFSEAPWDEHLLSKYPSLEIDWDAILS

>cDNA

ACCAAATCAAGCTCTTCCTCCGCCGCTGCCCCCCCACAGCTCTCATTCCCTCACCAAATCGGCAGGTTCATCCTTGATCACCATTTCCTTCCTCTGCTTCTCCGTCTGTGTGGGTCTGTCGGTTTCTTGATCGACCAAGGGCATGGCGACGACGGCGGACTGGCGCGGCTATATGCCCGAACTTCCTGCGGCGATGTACCGCATGGCCGAGAGCAGGGACCAGGTGATGCACGCGTTCGCCCCGCCGGCGGCGCACGGCGCGGCGCCGACCATCTCCTTCTCCTTCCCCTGCCCCGGCGCGGAGCAGGGCGGGGGCGCCGGCCTGCTCCGTGGCGCCAGCTACCTCACCCCCGCGCAAATCCTCCAGCTCCAGTCGCAGCTGCACCACGCGCGCCGGGCGCCGCCCGGCGCGGCCATGGCCATGGCCGCCGTGGGGCAGCCGATGAAGCGGCACGGCGTGGCGGCGCTCCCGGCGCGGCCGGCCGCCAAGCTGTACCGCGGCGTGCGGCAGCGGCATTGGGGGAAGTGGGTGGCCGAGATCCGCCTGCCCCGCAACCGCACCCGCCTCTGGCTCGGCACCTTCGACGCCGCCGAGGAGGCCGCGCTGGCCTACGACGCCGCCGCCTTCCGGCTCCGCGGCGAGTCCGCCAGGCTCAACTTCCCCGAGCTCAGGCGCGGCGGCCAGCACCACGGCCCGCCGCTCGACGCCGCCATCGACGCCAAGCTCCACTCCATCTGCCACGGGGAGGACCCGCCACAGAGCCAGAGCAATGCAACGCCGACGCCGACTTCTTTCCCGGACGTCAAGAGCGAGCCAGTCTGCTCCGTCTCCGAGAGCTCGTCGTCGGCCGACGGCGAGGTGTCCTCGTGCTCCGACGACGTCCCGGAGATGCAGCTTCTTGATTTCTCGGAGGCTCCGTGGGACGAGCACCTGCTGAGCAAGTACCCGTCGCTCGAGATCGACTGGGACGCCATCCTTTCCTGAATCAAGCTGCTAATGCTTGGTTCATAGCTCGATCAAAGTGATTGCTTCTGCTTTTATTAGGCTCTTGGATAGGTTATGGGTTTGATCAAGTATTCATGTGTACCACCATCGACCCCTGCTCTGCTTGGTTTTTGAATGGCATTGACAGGAAGGAGGTTCTATGTAG

>CDS

ATGTACCGCATGGCCGAGAGCAGGGACCAGGTGATGCACGCGTTCGCCCCGCCGGCGGCGCACGGCGCGGCGCCGACCATCTCCTTCTCCTTCCCCTGCCCCGGCGCGGAGCAGGGCGGGGGCGCCGGCCTGCTCCGTGGCGCCAGCTACCTCACCCCCGCGCAAATCCTCCAGCTCCAGTCGCAGCTGCACCACGCGCGCCGGGCGCCGCCCGGCGCGGCCATGGCCATGGCCGCCGTGGGGCAGCCGATGAAGCGGCACGGCGTGGCGGCGCTCCCGGCGCGGCCGGCCGCCAAGCTGTACCGCGGCGTGCGGCAGCGGCATTGGGGGAAGTGGGTGGCCGAGATCCGCCTGCCCCGCAACCGCACCCGCCTCTGGCTCGGCACCTTCGACGCCGCCGAGGAGGCCGCGCTGGCCTACGACGCCGCCGCCTTCCGGCTCCGCGGCGAGTCCGCCAGGCTCAACTTCCCCGAGCTCAGGCGCGGCGGCCAGCACCACGGCCCGCCGCTCGACGCCGCCATCGACGCCAAGCTCCACTCCATCTGCCACGGGGAGGACCCGCCACAGAGCCAGAGCAATGCAACGCCGACGCCGACTTCTTTCCCGGACGTCAAGAGCGAGCCAGTCTGCTCCGTCTCCGAGAGCTCGTCGTCGGCCGACGGCGAGGTGTCCTCGTGCTCCGACGACGTCCCGGAGATGCAGCTTCTTGATTTCTCGGAGGCTCCGTGGGACGAGCACCTGCTGAGCAAGTACCCGTCGCTCGAGATCGACTGGGACGCCATCCTTTCCTGA

>DNA

ACCAAATCAAGCTCTTCCTCCGCCGCTGCCCCCCCACAGCTCTCATTCCCTCACCAAATCGGCAGGTTCATCCTTGATCACCATTTCCTTCCTCTGCTTCTCCGTCTGTGTGGGTCTGTCGGTTTCTTGATCGACCAAGGGCATGGCGACGACGGCGGACTGGCGCGGCTATATGCCCGAACTTCCTGCGGCGATGTACCGCATGGCCGAGAGCAGGGACCAGGTGATGCACGCGTTCGCCCCGCCGGCGGCGCACGGCGCGGCGCCGACCATCTCCTTCTCCTTCCCCTGCCCCGGCGCGGAGCAGGGCGGGGGCGCCGGCCTGCTCCGTGGCGCCAGCTACCTCACCCCCGCGCAAATCCTCCAGCTCCAGTCGCAGCTGCACCACGCGCGCCGGGCGCCGCCCGGCGCGGCCATGGCCATGGCCGCCGTGGGGCAGCCGATGAAGCGGCACGGCGTGGCGGCGCTCCCGGCGCGGCCGGCCGCCAAGCTGTACCGCGGCGTGCGGCAGCGGCATTGGGGGAAGTGGGTGGCCGAGATCCGCCTGCCCCGCAACCGCACCCGCCTCTGGCTCGGCACCTTCGACGCCGCCGAGGAGGCCGCGCTGGCCTACGACGCCGCCGCCTTCCGGCTCCGCGGCGAGTCCGCCAGGCTCAACTTCCCCGAGCTCAGGCGCGGCGGCCAGCACCACGGCCCGCCGCTCGACGCCGCCATCGACGCCAAGCTCCACTCCATCTGCCACGGGGAGGACCCGCCACAGAGCCAGAGCAATGCAACGCCGACGCCGACTTCTTTCCCGGACGTCAAGAGCGAGCCAGTCTGCTCCGTCTCCGAGAGCTCGTCGTCGGCCGACGGCGAGGTGTCCTCGTGCTCCGACGACGTCCCGGAGATGCAGCTTCTTGATTTCTCGGAGGCTCCGTGGGACGAGCACCTGCTGAGCAAGTACCCGTCGCTCGAGATCGACTGGGACGCCATCCTTTCCTGAATCAAGCTGCTAATGCTTGGTTCATAGCTCGATCAAAGTGATTGCTTCTGCTTTTATTAGGCTCTTGGATAGGTTATGGGTTTGATCAAGTATTCATGTGTACCACCATCGACCCCTGCTCTGCTTGGTTTTTGAATGGCATTGACAGGAAGGAGGTTCTATGTAG

>HvDREB6.7

>Protein

MAAAIDLSGEDLVRALEPFIREASAPPPLHSHPSPTSPFSFPHAAYSGYPYGVQAQAQTELSPAQMHYIQARLHLQRQTGQPGHLGPRPQPMKPASAAAATPPRPQKLYRGVRQRHWGKWVAEIRLPRNRTRLWLGTFDTAEEAALAYDQAAYRLRGDAARLNFPDNAASRGPLHASVDAKLQTLCQNITASKNAKKSASVSASTAAATSSTPTSNCSSPSSDEASSSLESAESSPSPTTTAAEVPEMQQLDFSEAPWDEAAGFALTKYPSYEIDWDSLLATN

>cDNA

AATCCCTAGATGGCTGCAGCTATAGATCTGTCCGGGGAGGATCTGGTGAGAGCACTCGAGCCTTTTATCCGAGAGGCCTCTGCCCCCCCTCCGCTCCACTCCCATCCTAGTCCCACCTCGCCATTCTCCTTCCCCCACGCCGCCTACAGTGGTTACCCGTACGGGGTGCAGGCACAGGCCCAGACCGAGCTCAGCCCGGCCCAGATGCACTACATCCAGGCACGCCTCCACCTCCAGCGCCAGACCGGCCAGCCGGGCCACCTCGGCCCGCGGCCCCAGCCCATGAAGCCCGCTTCGGCGGCAGCGGCCACACCGCCGCGGCCGCAGAAGCTCTACCGCGGCGTTCGGCAGCGCCACTGGGGCAAGTGGGTGGCGGAGATCCGCCTCCCCCGCAACCGCACCCGCCTCTGGCTCGGCACCTTCGACACCGCCGAGGAGGCGGCTCTCGCCTACGACCAGGCCGCCTACCGCCTCCGTGGCGACGCAGCGCGCCTCAACTTCCCCGACAACGCCGCCTCCCGCGGCCCGCTCCATGCCTCTGTTGACGCCAAGCTCCAGACCCTCTGCCAGAACATCACCGCTTCCAAGAACGCCAAGAAGTCCGCCTCCGTCTCCGCGTCCACCGCCGCAGCCACGTCGTCCACCCCCACCAGCAACTGCTCCTCGCCGTCCTCCGACGAGGCGTCGTCCTCGCTCGAGTCCGCCGAGTCGTCACCATCACCCACCACCACCGCAGCAGAGGTTCCTGAGATGCAGCAGCTCGACTTCAGCGAGGCACCATGGGACGAGGCAGCCGGCTTCGCCCTCACCAAGTACCCGTCCTATGAGATCGACTGGGACTCGCTCCTCGCCACCAATTAGCACCCAGTTCATCTTCGTCAGCTACTACTACCAGTACCGTCTTTTAGCGTGTCATGATGCTAGGTTAATGGGTCGCCGCGATGCAGATGGCATTTTAGACATTCTGCGCCGGCCTTTAGCGGATTAGCTCTAAGTCTCTAATCCTTGTTCATTGTGTAGACCTATGATTCG

>CDS

ATGGCTGCAGCTATAGATCTGTCCGGGGAGGATCTGGTGAGAGCACTCGAGCCTTTTATCCGAGAGGCCTCTGCCCCCCCTCCGCTCCACTCCCATCCTAGTCCCACCTCGCCATTCTCCTTCCCCCACGCCGCCTACAGTGGTTACCCGTACGGGGTGCAGGCACAGGCCCAGACCGAGCTCAGCCCGGCCCAGATGCACTACATCCAGGCACGCCTCCACCTCCAGCGCCAGACCGGCCAGCCGGGCCACCTCGGCCCGCGGCCCCAGCCCATGAAGCCCGCTTCGGCGGCAGCGGCCACACCGCCGCGGCCGCAGAAGCTCTACCGCGGCGTTCGGCAGCGCCACTGGGGCAAGTGGGTGGCGGAGATCCGCCTCCCCCGCAACCGCACCCGCCTCTGGCTCGGCACCTTCGACACCGCCGAGGAGGCGGCTCTCGCCTACGACCAGGCCGCCTACCGCCTCCGTGGCGACGCAGCGCGCCTCAACTTCCCCGACAACGCCGCCTCCCGCGGCCCGCTCCATGCCTCTGTTGACGCCAAGCTCCAGACCCTCTGCCAGAACATCACCGCTTCCAAGAACGCCAAGAAGTCCGCCTCCGTCTCCGCGTCCACCGCCGCAGCCACGTCGTCCACCCCCACCAGCAACTGCTCCTCGCCGTCCTCCGACGAGGCGTCGTCCTCGCTCGAGTCCGCCGAGTCGTCACCATCACCCACCACCACCGCAGCAGAGGTTCCTGAGATGCAGCAGCTCGACTTCAGCGAGGCACCATGGGACGAGGCAGCCGGCTTCGCCCTCACCAAGTACCCGTCCTATGAGATCGACTGGGACTCGCTCCTCGCCACCAATTAG

>DNA

TCTCGAGCTTTGGAGTAGACGACTATTACAAAAACAAGGGAAAAGCTATTCCGCGCAGAATGGAACCAACCTTTCACCAAAATGCATGTTTATTCCGTAATGCCAATTCTAATGAAGCTTGCCGACACATTTACTGTATTGACCCTTGTGGTTGCAATGTATCATGTTACCAACTTATCGTGTGGAAGGGTAGATATGAAGTAGGGTTACGACACTATACTCAATTCTTGACACTCAGTGAATGGATGCCATAATACTAGAATATTTTCTCTGTTGCATGATTGTATACTAAGCTAGCCCATAAGCTAAGCATATGTGTAGTGTAAAAGCAGTGGATCTAGGATGTAATACTAGGACATATGATGTATTCTCGGTATTTCATCGTCGACTGTACGTGTCAGCTATTGATCCACAGACTCACATAAAAAGCGCAAACATTCGAGCCCCTTCTCGGGAGGAGGTCATGACACTCCTTTTCATAATTCTATTTTTCTTTTATTTTTCATTGAACAAAACTAGATAATGACCTACGCCCCGCCAAGTAGTGATATGTGTGACGTCCTCCCACAAGGACGACACGAGGCGGTGGAGGGGCTGCTCGTGAAAGACGGTGTGGTGAGGAGGGGTGATTTGATGGGGACAACATTTCGCTGGGGCGATGACAAGGGGCGGGTGTTGCGATTACACGCGAAAGTGTTAGGTGATCTTTAGGATGCCAATAGTTTAGGCGAAGGAAAACTTTCCCTAAACCTGAGAAAAGTCGGGGGCAAATTTATAAGATAATGTAAATTTGTTTCATGTTTATGGATCTATTGGAGAGATTTTTCGGCCCAAACTTTTCTTAAACGGTATGCTTTTGTACGGATTGATCTGTTGGAGTTGCTATAAAGCAGTTCACGAAAAGCATGAACCAAATTAGGAAAATTCTAATGACCAGAAATAACCGTCGCCTCGCCGCGTTCAAAAAGAAAAAGGAAAAGAAATAACCGTTACCTCCGAGCGACCGCGCAACCTCCGCGCGAGCCGTTTGCCACTTGCAGCGGCTGAGGCAGCGATTCCTGCGGAATATTCGCGGCACCGGCGAAGCTGATCGGCGTCGACCAGCAGACACCAGGAAACACCAAACAGCGCCTCGCCACGGCGGCGACACCGGCGACACCCGGTTCCTCACCCGCACACGTGTCGGTGCGCAGGACACCCGGCGCCCTCCCAGTGGCGGAACTCGCGCGCGCTGCTCGACGCGGTTCACCCACACCACATCCGATCGAGCATTTAATGCCTGTGCCTCTGCCCGCTGTCCCACATGACAGTCGCGCAGAGGGGGAGGAGTGGGAGCGAAGCAAACCACTAATCACAGCCCTAAACAACCGCGAGAGGACGGAAAAGGTGCAAATTATTATTCGGGAGAGGATAAAGATAAACTATGGCCATCAAGTCGTGGGCTGCCCAGCCCAGGGGCCGCCCGCGCTATAAAGGAGCTGCTTGGCCCTCCAGTCCTCCTCCCCGCCTCCATTTCGCCCACTCCCACCTTCGTCCACACACACACACACGCATTCCACAGCCACCAGCCACCGAAAAAGCCCCAATCCCAGCAACCACAAACACTCACGTGAGTTCGAGCGAGGTAAGCATAGATCCGAGCGAGTTCTTGCGTCGCCAAGGTAAGGTTCTCTGAGTCTCAACCCCTCTTCCTCCCTCCCCTGTTCGTCCCCTTGTTCACCCAGCAGACAGTGAGGTCTCCAGGACGTTTTAGATCCCGGGCTCCAGATCTGCTCTTTTTTTCTTCTGTTTTAGTTGTAGTAATCGGCCAGGCGAGGTTCTTTTTCCACACCTCCTTGCTCGTCGTGTCTAGATCTACCACCAACGCCATCTCTTTTTGCTGTGGCCGTTGTTGATTTTTCTCTGCCTGTTCTAGGTTTTGGTTTGGAAACCAGGCAGCTTTCTTTCTTGTTCATCTTTCCAAGAAAACAAAACAAAAAAACACCAAAAAAAATCCCTAGATGGCTGCAGCTATAGATCTGTCCGGGGAGGATCTGGTGAGAGCACTCGAGCCTTTTATCCGAGAGGCCTCTGCCCCCCCTCCGCTCCACTCCCATCCTAGTCCCACCTCGCCATTCTCCTTCCCCCACGCCGCCTACAGTGGTTACCCGTACGGGGTGCAGGCACAGGCCCAGACCGAGCTCAGCCCGGCCCAGATGCACTACATCCAGGCACGCCTCCACCTCCAGCGCCAGACCGGCCAGCCGGGCCACCTCGGCCCGCGGCCCCAGCCCATGAAGCCCGCTTCGGCGGCAGCGGCCACACCGCCGCGGCCGCAGAAGCTCTACCGCGGCGTTCGGCAGCGCCACTGGGGCAAGTGGGTGGCGGAGATCCGCCTCCCCCGCAACCGCACCCGCCTCTGGCTCGGCACCTTCGACACCGCCGAGGAGGCGGCTCTCGCCTACGACCAGGCCGCCTACCGCCTCCGTGGCGACGCAGCGCGCCTCAACTTCCCCGACAACGCCGCCTCCCGCGGCCCGCTCCATGCCTCTGTTGACGCCAAGCTCCAGACCCTCTGCCAGAACATCACCGCTTCCAAGAACGCCAAGAAGTCCGCCTCCGTCTCCGCGTCCACCGCCGCAGCCACGTCGTCCACCCCCACCAGCAACTGCTCCTCGCCGTCCTCCGACGAGGCGTCGTCCTCGCTCGAGTCCGCCGAGTCGTCACCATCACCCACCACCACCGCAGCAGAGGTTCCTGAGATGCAGCAGCTCGACTTCAGCGAGGCACCATGGGACGAGGCAGCCGGCTTCGCCCTCACCAAGTACCCGTCCTATGAGATCGACTGGGACTCGCTCCTCGCCACCAATTAGCACCCAGTTCATCTTCGTCAGCTACTACTACCAGTACCGTCTTTTAGCGTGTCATGATGCTAGGTTAATGGGTCGCCGCGATGCAGATGGCATTTTAGACATTCTGCGCCGGCCTTTAGCGGATTAGCTCTAAGTCTCTAATCCTTGTTCATTGTGTAGACCTATGATTCG

>HvERF1.1

>Protein

MAPRAAEKAPVSPPTGLGLGVGGGVGVVAGGAHYRGVRKRPWGRFAAEIRDPAKKSRVWLGTYDTAEEAARAYDTAAREFRGAKAKTNFPFPSSSSPSPLAAGGGSPSSNSTLDSSGGGSGGCAQAPMQAIPLPPALDLDLFHRAAAVTAGGMRFPFNGYPVAPRQPLHPYFFYEQAAAAAAASSGYRTLKMAQPVTVAAVAQSDSDSSSVVDLSPSPPAVTAHKAVAFDLDLNRPPPSED

>cDNA

GCGAGGACAGTCAAACCGCATGCTGACTGCGCCGCGTTTTATTCCCTCTCGCCGCTCCCTCCACCGCTCACCGCTCGCTCCCCTTTTCCCCTACTCACTTCCCTTTTTTAACCCCCCGTCCATCTATAAATGCACGCCCCCCTCCTCGCCTTATCCTGGGCGCTACACACCCAACCCACCCCACCTCACCAAAACGCCCGCATAACCAAAAAGATACGCGCACGCCACTTGTACACCTGCTGCACCCATGGCGCCTAGAGCGGCGGAGAAGGCGCCTGTCTCCCCGCCCACCGGGCTCGGCCTTGGCGTTGGCGGCGGCGTCGGGGTCGTAGCCGGCGGCGCCCACTACCGTGGCGTTCGGAAGCGCCCCTGGGGACGTTTCGCCGCAGAGATCCGCGACCCGGCCAAGAAGAGCAGGGTGTGGCTCGGCACGTACGACACGGCGGAGGAGGCCGCGCGCGCCTACGACACCGCCGCGCGCGAGTTCCGCGGCGCCAAGGCCAAAACGAACTTCCCGTTCCCTTCGTCGTCGTCGCCGTCTCCTCTCGCCGCCGGCGGCGGCAGCCCGAGCAGCAACAGCACCTTGGACTCGAGCGGTGGTGGGAGCGGCGGCTGCGCCCAGGCGCCTATGCAGGCCATCCCGCTGCCGCCCGCCCTCGACCTGGACCTCTTCCACCGCGCGGCGGCCGTGACCGCCGGCGGCATGCGCTTTCCATTCAACGGTTACCCGGTGGCGCCGCGCCAGCCCCTGCACCCGTACTTCTTCTACGAACAGGCCGCGGCCGCCGCGGCGGCTTCGTCAGGTTACCGCACGCTGAAGATGGCGCAGCCGGTCACCGTGGCGGCCGTTGCCCAGAGCGACTCCGACTCCTCGTCGGTCGTTGATCTGTCCCCGTCGCCCCCAGCGGTGACAGCGCATAAGGCGGTCGCGTTTGATCTGGATCTGAACCGGCCGCCGCCTTCGGAGGACTAGACAAAGGACAAATTTTAGATGATGACTGTAGCTTGTCATTTTTCCTGCAGGGAACGTTTTTTTCCCTCCTTCCTTCCTCGTTTTGGTCCTCTTGTATTTTTGTTTAGTTGTCTGAGACAGACCGAGGAGCCCTGTAAATAGTTTTTCCGCCGAGAACGTAGCAGAACCGATCTGAGATCTGTTCGTCTAAACGGATCAAACCGGCGTGGAGGTGAACCGAGACTTGAGTATATGTGTATCTCGTAAGATGAGTGTAGTATGATGATGAGTTTTTCAGGACAGGAGTAGTACTTCTTTTCTTTTTGTTCAGAAAGGCAAGATGAATGGTTGGGAAACGTCATCAGTTTTGCTGCCATCTCTGCGACACGACAGTTAATGGTTTGGTGAATTGCACGTCTCACTCTGCATGGTTGCTTCTGGACTCGACTGCCTTGAGGTTGCGCAGGAGTAATTGAGCTGGAAGGAGTAGCAGTATTAACGTAATTTTGCATGCCATTATTGCGCACTCAGCTGTCAAATTGCGGCTTTCAAACCCTTCACACGCTGACAGCCTCGGAGAAAAAGAAAGATGGGTAGATCTAGGACGGAAAGGGCCAAGGTGCAGTGGGCACGAGAGACAAGTGTGGACGGGAGAGAAAGAGAGGGATGGCGTGCGAGCGGAGCGGTGCGGTGGACGGACCGACGATCCGTAGGCGCGATGGAATCGCGACCGCGTTGTTCTACTCGTGACCTTGAGAATAACGTGTGCGTGTGGGCGACGTCTGTACG

>CDS

ATGGCGCCTAGAGCGGCGGAGAAGGCGCCTGTCTCCCCGCCCACCGGGCTCGGCCTTGGCGTTGGCGGCGGCGTCGGGGTCGTAGCCGGCGGCGCCCACTACCGTGGCGTTCGGAAGCGCCCCTGGGGACGTTTCGCCGCAGAGATCCGCGACCCGGCCAAGAAGAGCAGGGTGTGGCTCGGCACGTACGACACGGCGGAGGAGGCCGCGCGCGCCTACGACACCGCCGCGCGCGAGTTCCGCGGCGCCAAGGCCAAAACGAACTTCCCGTTCCCTTCGTCGTCGTCGCCGTCTCCTCTCGCCGCCGGCGGCGGCAGCCCGAGCAGCAACAGCACCTTGGACTCGAGCGGTGGTGGGAGCGGCGGCTGCGCCCAGGCGCCTATGCAGGCCATCCCGCTGCCGCCCGCCCTCGACCTGGACCTCTTCCACCGCGCGGCGGCCGTGACCGCCGGCGGCATGCGCTTTCCATTCAACGGTTACCCGGTGGCGCCGCGCCAGCCCCTGCACCCGTACTTCTTCTACGAACAGGCCGCGGCCGCCGCGGCGGCTTCGTCAGGTTACCGCACGCTGAAGATGGCGCAGCCGGTCACCGTGGCGGCCGTTGCCCAGAGCGACTCCGACTCCTCGTCGGTCGTTGATCTGTCCCCGTCGCCCCCAGCGGTGACAGCGCATAAGGCGGTCGCGTTTGATCTGGATCTGAACCGGCCGCCGCCTTCGGAGGACTAG

>DNA

ATCACTTAGTTGTTTATCTTGCATAACTTGTTGGTGCACATAGGTGAACCTAGTTGTTTGAGGTTTGTGCTTGACATATTAAACGTTAGTTTTATTTCGTATTTGTTCAAGTCTAAACCTTAATTATTTTAAAGCGTCTATTCACCCCCCTCTAAACGACATCCACGTCCTTTCATAAACAACACGCCGCCAAAAAACCCTTTCTTGAGGTTTGAGACTTTTTTGATACTTTTTTAATAGTGCCCCTACTTTTAGCATGTCATTTAATAACTTCTAATACTAGGGGTAACATGATCTTTTTGCATGTCATTTAATGACCTCTATTTTCTGTTTAATCCCTGAAAAAAAACTTATAGGGACTAGGGATTTTAAAGAAACATGGGCTAAATATTACTTCGTGTGACGCTAGAATGGGATTGAGCGACAAACTTGGGGGTATGGCGACGCAAAATACAAATATCATCATCAGGGCTTGTTCGGTTAATCCCCATCACAAGTGGATTGAAGGGGATTGGAGGGGTTTGAGATGAATTTTGACTTGCTGGGGATTTAATCCCTTCAAATCCCTCGAAGAAGGCCTCAAGCGGAACCGTTCCCTGGCATACGGCATATATTGTGCAAAATTGATGTCTGGCGGTGGGCTAAACAGAAAAAGGCGCCGCTGGCCTAATTTAATGCCGCCATGCTAAAGACGAGTGGGGGGCGGCGGCGAGGACAGTCAAACCGCATGCTGACTGCGCCGCGTTTTATTCCCTCTCGCCGCTCCCTCCACCGCTCACCGCTCGCTCCCCTTTTCCCCTACTCACTTCCCTTTTTTAACCCCCCGTCCATCTATAAATGCACGCCCCCCTCCTCGCCTTATCCTGGGCGCTACACACCCAACCCACCCCACCTCACCAAAACGCCCGCATAACCAAAAAGATACGCGCACGCCACTTGTACACCTGCTGCACCCATGGCGCCTAGAGCGGCGGAGAAGGCGCCTGTCTCCCCGCCCACCGGGCTCGGCCTTGGCGTTGGCGGCGGCGTCGGGGTCGTAGCCGGCGGCGCCCACTACCGTGGCGTTCGGAAGCGCCCCTGGGGACGTTTCGCCGCAGAGATCCGCGACCCGGCCAAGAAGAGCAGGGTGTGGCTCGGCACGTACGACACGGCGGAGGAGGCCGCGCGCGCCTACGACACCGCCGCGCGCGAGTTCCGCGGCGCCAAGGCCAAAACGAACTTCCCGTTCCCTTCGTCGTCGTCGCCGTCTCCTCTCGCCGCCGGCGGCGGCAGCCCGAGCAGCAACAGCACCTTGGACTCGAGCGGTGGTGGGAGCGGCGGCTGCGCCCAGGCGCCTATGCAGGCCATCCCGCTGCCGCCCGCCCTCGACCTGGACCTCTTCCACCGCGCGGCGGCCGTGACCGCCGGCGGCATGCGCTTTCCATTCAACGGTTACCCGGTGGCGCCGCGCCAGCCCCTGCACCCGTACTTCTTCTACGAACAGGCCGCGGCCGCCGCGGCGGCTTCGTCAGGTTACCGCACGCTGAAGATGGCGCAGCCGGTCACCGTGGCGGCCGTTGCCCAGAGCGACTCCGACTCCTCGTCGGTCGTTGATCTGTCCCCGTCGCCCCCAGCGGTGACAGCGCATAAGGCGGTCGCGTTTGATCTGGATCTGAACCGGCCGCCGCCTTCGGAGGACTAGACAAAGGACAAATTTTAGATGATGACTGTAGCTTGTCATTTTTCCTGCAGGGAACGTTTTTTTCCCTCCTTCCTTCCTCGTTTTGGTCCTCTTGTATTTTTGTTTAGTTGTCTGAGACAGACCGAGGAGCCCTGTAAATAGTTTTTCCGCCGAGAACGTAGCAGAACCGATCTGAGATCTGTTCGTCTAAACGGATCAAACCGGCGTGGAGGTGAACCGAGACTTGAGTATATGTGTATCTCGTAAGATGAGTGTAGTATGATGATGAGTTTTTCAGGACAGGAGTAGTACTTCTTTTCTTTTTGTTCAGAAAGGCAAGATGAATGGTTGGGAAACGTCATCAGTTTTGCTGCCATCTCTGCGACACGACAGTTAATGGTTTGGTGAATTGCACGTCTCACTCTGCATGGTTGCTTCTGGACTCGACTGCCTTGAGGTTGCGCAGGAGTAATTGAGCTGGAAGGAGTAGCAGTATTAACGTAATTTTGCATGCCATTATTGCGCACTCAGCTGTCAAATTGCGGCTTTCAAACCCTTCACACGCTGACAGCCTCGGAGAAAAAGAAAGATGGGTAGATCTAGGACGGAAAGGGCCAAGGTGCAGTGGGCACGAGAGACAAGTGTGGACGGGAGAGAAAGAGAGGGATGGCGTGCGAGCGGAGCGGTGCGGTGGACGGACCGACGATCCGTAGGCGCGATGGAATCGCGACCGCGTTGTTCTACTCGTGACCTTGAGAATAACGTGTGCGTGTGGGCGACGTCTGTACG

>HvERF1.2

>Protein

MAPRTSDKTATPPAAAVAATGLALGVGGGANGGGVGPHYRGVRKRPWGRYAAEIRDPAKKSRVWLGTYDTAEEAARAYDAAAREYRGNKAKTNFPFASAPPAAALTGDGSRSSNSSTVDSFGGDVQAPMQAMPLPPSVELDLFHRAASTAGAGMRSPFSGYPVSHPYYFFGQAAAAAAAGCHMYNLAPKVTVASVSPSDSDSSSIVDLAPSPPARKPVPFDLDLNCPPPAEH

>cDNA

CTGGTGTGCTTCTTTTGTGTGTTTTATTCATTTTATTTAGTTGCTTTTATGCATTTTCTAGTGCACACAAACTTCATGCCCAACGCGTGACCGAGAAGGGACGGAACCACAAAATTTGTAACGGATCCAGGGTTGGGGCAATTCCGCGGATCCAGAGGGTCCGGATGAAAAAGCGGGCGTTACAATTGTAATGCCGCCACGCTAAAAGACGAGGGGCGGCGGCGCGGACAGTCAAACCGCTCCCGACTGCGCCGAGTTTTATTCCCTCCTCTTGCCGCTCCACCACTCGCTCCCCATTTCCTCCCTTTTTAACCAGCTGCTCCCCCCCACCCCCTATAATAAGGAGCGTCCCTCCTCCCTCTCTCCCACCTCCCAGATCCTGCACGCCATCCATCAGCTTGATACGCACACACCCATCCGCAACAAGAAAAAGAAAGGGAAGCCATGGCGCCCAGGACGTCCGACAAGACGGCGACGCCGCCTGCTGCCGCGGTCGCCGCGACCGGCCTGGCGCTCGGCGTCGGCGGCGGCGCCAACGGTGGAGGCGTCGGCCCGCACTACAGGGGCGTGAGGAAGCGCCCGTGGGGCCGGTACGCGGCGGAGATCCGCGACCCGGCCAAGAAGAGCCGGGTGTGGCTCGGCACGTACGACACGGCCGAGGAGGCCGCGCGGGCCTACGACGCCGCCGCGCGCGAGTACCGCGGCAACAAGGCCAAGACCAACTTCCCCTTCGCCTCCGCGCCGCCCGCCGCAGCCCTCACCGGCGACGGCAGCCGGAGCAGCAACAGCAGCACCGTGGACTCCTTCGGCGGCGACGTGCAGGCACCCATGCAGGCCATGCCGCTCCCTCCCTCCGTCGAGCTCGACCTGTTCCACCGCGCGGCCAGCACCGCCGGGGCCGGCATGCGGTTCCCTTTCAGCGGCTACCCCGTTTCGCACCCGTACTACTTCTTCGGACAGGCCGCGGCGGCCGCCGCCGCCGGCTGCCACATGTACAACCTGGCCCCGAAGGTCACCGTGGCGTCCGTGTCCCCGAGCGACTCCGACTCCTCGTCGATCGTGGATCTGGCGCCGTCGCCGCCCGCAAGGAAGCCCGTCCCTTTTGATCTTGACCTGAACTGCCCGCCGCCGGCCGAGCACTGATCGGTGGCCCGAGTTTATTTAGATGATATGACTGCTAGTTTTCTTTCGTCTCCTTTTTCTTTTTCTCTCCCTAGAAGGAAGAAAAAACTCCATGTACCTCCATGATGCTTAGTTGAGGCCCCCTTGCATAACAACGGCAGGGGCAGATCTGTAAATACGGTTTCTTTTTTTCGCCGGAGAGTAGTTGGCTTGAGCAAGAGAGCCAGAGAGATGGATGTATTGTCGCCCAAGACAGATCCTGCCGGCGTTGTTATGGACCGAATTATAATGAGTTACTTCTACTTTGACAGCCCAAGCGACCACTCGTATTTACTCTCTACTAGTACTCAGTTATTGAAGCATTTTATAGCAGATCTTTTGCTGAAACGGAAGATACCCCGAGTAGTAGATGGAGCAGAGTAAGTGCGTTTGGGTGGGTTTGCGCCCGTC

>CDS

ATGGCGCCCAGGACGTCCGACAAGACGGCGACGCCGCCTGCTGCCGCGGTCGCCGCGACCGGCCTGGCGCTCGGCGTCGGCGGCGGCGCCAACGGTGGAGGCGTCGGCCCGCACTACAGGGGCGTGAGGAAGCGCCCGTGGGGCCGGTACGCGGCGGAGATCCGCGACCCGGCCAAGAAGAGCCGGGTGTGGCTCGGCACGTACGACACGGCCGAGGAGGCCGCGCGGGCCTACGACGCCGCCGCGCGCGAGTACCGCGGCAACAAGGCCAAGACCAACTTCCCCTTCGCCTCCGCGCCGCCCGCCGCAGCCCTCACCGGCGACGGCAGCCGGAGCAGCAACAGCAGCACCGTGGACTCCTTCGGCGGCGACGTGCAGGCACCCATGCAGGCCATGCCGCTCCCTCCCTCCGTCGAGCTCGACCTGTTCCACCGCGCGGCCAGCACCGCCGGGGCCGGCATGCGGTTCCCTTTCAGCGGCTACCCCGTTTCGCACCCGTACTACTTCTTCGGACAGGCCGCGGCGGCCGCCGCCGCCGGCTGCCACATGTACAACCTGGCCCCGAAGGTCACCGTGGCGTCCGTGTCCCCGAGCGACTCCGACTCCTCGTCGATCGTGGATCTGGCGCCGTCGCCGCCCGCAAGGAAGCCCGTCCCTTTTGATCTTGACCTGAACTGCCCGCCGCCGGCCGAGCACTGA

>DNA

TACTATTAGAACAATAGCTAAAATTGAGTGAGCAAACACATATGGCATAACCAACATTCATATATGCCTAGTATAGTAAAATCCGCATATGTAAAAAGAGATAACAGGAAGCATCTTCATTGCACTTAATATGTCGCTACAAATTACTATCTTGATATAAATGTATCGTCCAACATTAATTAAAATTTGAGAGCATTTTATGTCGCAAAAAAATATTTCCATTTTAGAATGAAGTGTAGCTTAGTCATCATCCTACACAATCATGGCTAGCTATTACTTTCACAAACATTTACGACAAAACATTAGCTAGAACCGAGTGAGCATACCCTTATTGATATGGACATGACCCCTATAGAGATGACCATACTTATTATGGACAAAGAGAATTATCAGGTGAATTCTAATATAAATTATATATACTAGAATTCACGTGATAATTGTCTTTGCACGTAATAATTATGGTTATATCTATAGGGTCATGTCCAAATCACTTATGACACAACCGACGACATTTGCTTTGTTTATGGTTACAGAAAAATGTAGAGCTATCGTCCAGTCACACACTCAAATCTACGCGATATTATTAAATATGCATACGACATATAAAAAATAACGATAAGTATTTCATTGCACTTAAAAATGTTGCTTCAATTACCACATGATACCCAATTTTGTTTAGATTGTAGTGAATTGTAACGCTATCATTGTCGTACACAATCATATCTCATCACGCACACTCAGAGCGAGCTACTACCATCACAGTTACAAAACAAATGAATACATTTTGTTATAACTTATATGCTACCATAAGTTTGTAAACGTTTTTTAGATACTCCTATGTTGATACCATTGGCTTAGACATGTTACTATTCACACTTCATTTATCACAATTGAACGACCACAATGAGAAGTCATGGATAACAAAAAGATAAATAGAACAAATAAAGTAATACTATAGATTGGATAGCATCTTTCTCCAAGGAAGAGTTTGCTGGAGCTAACCTAAACCACTACCATCTTCCTCGACTCACTACAAGTGAGGCAGTTAGTTCCCCTTCCTTTTGCCGGATCGTAGGTTGTGTTAGGGTGAGCAAACCCCAAATAAGCGATTATGACTTGTAGTCCGTTGGTGTTAGTCTCTTTTGGCGGCACTTTGGTGCTGAAGATGAAGCCATGGAGGATAGTTCTATTGCAGCTTTGTCCCTACCATGGTAGTGTTGATTTGAACGACGTTGACGCGTTGGCAACCACGTGATCTTATCGACCGACGCGGTTCACAATTGATTATCAGCCGTGTTTATTTGGGAGGTGTCAGCGCCTTGATGCCTTTTTTGTGGTGACCTTTGTCATGTAATCCAGCATCATCCTCTTCTCTAGGCCCTTGCTCGTCCCGGATGGACGTCACTTCCGTTTCATGGCAGGGTTCGATGATTTCTCAGTTGCATGTGTGGATGAAACTTTATTGTTACAACAATGGCGCGAAACTATAGTTGTTGCTACAATGGACGTGTCATTGGTATAGGTTCGCGAAGAAGACACATCCCATAATTCAATTGCTGGTGTGCTTCTTTTGTGTGTTTTATTCATTTTATTTAGTTGCTTTTATGCATTTTCTAGTGCACACAAACTTCATGCCCAACGCGTGACCGAGAAGGGACGGAACCACAAAATTTGTAACGGATCCAGGGTTGGGGCAATTCCGCGGATCCAGAGGGTCCGGATGAAAAAGCGGGCGTTACAATTGTAATGCCGCCACGCTAAAAGACGAGGGGCGGCGGCGCGGACAGTCAAACCGCTCCCGACTGCGCCGAGTTTTATTCCCTCCTCTTGCCGCTCCACCACTCGCTCCCCATTTCCTCCCTTTTTAACCAGCTGCTCCCCCCCACCCCCTATAATAAGGAGCGTCCCTCCTCCCTCTCTCCCACCTCCCAGATCCTGCACGCCATCCATCAGCTTGATACGCACACACCCATCCGCAACAAGAAAAAGAAAGGGAAGCCATGGCGCCCAGGACGTCCGACAAGACGGCGACGCCGCCTGCTGCCGCGGTCGCCGCGACCGGCCTGGCGCTCGGCGTCGGCGGCGGCGCCAACGGTGGAGGCGTCGGCCCGCACTACAGGGGCGTGAGGAAGCGCCCGTGGGGCCGGTACGCGGCGGAGATCCGCGACCCGGCCAAGAAGAGCCGGGTGTGGCTCGGCACGTACGACACGGCCGAGGAGGCCGCGCGGGCCTACGACGCCGCCGCGCGCGAGTACCGCGGCAACAAGGCCAAGACCAACTTCCCCTTCGCCTCCGCGCCGCCCGCCGCAGCCCTCACCGGCGACGGCAGCCGGAGCAGCAACAGCAGCACCGTGGACTCCTTCGGCGGCGACGTGCAGGCACCCATGCAGGCCATGCCGCTCCCTCCCTCCGTCGAGCTCGACCTGTTCCACCGCGCGGCCAGCACCGCCGGGGCCGGCATGCGGTTCCCTTTCAGCGGCTACCCCGTTTCGCACCCGTACTACTTCTTCGGACAGGCCGCGGCGGCCGCCGCCGCCGGCTGCCACATGTACAACCTGGCCCCGAAGGTCACCGTGGCGTCCGTGTCCCCGAGCGACTCCGACTCCTCGTCGATCGTGGATCTGGCGCCGTCGCCGCCCGCAAGGAAGCCCGTCCCTTTTGATCTTGACCTGAACTGCCCGCCGCCGGCCGAGCACTGATCGGTGGCCCGAGTTTATTTAGATGATATGACTGCTAGTTTTCTTTCGTCTCCTTTTTCTTTTTCTCTCCCTAGAAGGAAGAAAAAACTCCATGTACCTCCATGATGCTTAGTTGAGGCCCCCTTGCATAACAACGGCAGGGGCAGATCTGTAAATACGGTTTCTTTTTTTCGCCGGAGAGTAGTTGGCTTGAGCAAGAGAGCCAGAGAGATGGATGTATTGTCGCCCAAGACAGATCCTGCCGGCGTTGTTATGGACCGAATTATAATGAGTTACTTCTACTTTGACAGCCCAAGCGACCACTCGTATTTACTCTCTACTAGTACTCAGTTATTGAAGCATTTTATAGCAGATCTTTTGCTGAAACGGAAGATACCCCGAGTAGTAGATGGAGCAGAGTAAGTGCGTTTGGGTGGGTTTGCGCCCGTC

>HvERF1.3

>Protein

MDPVLRGAASPGGGQGGAGGGGGGGEAHYRGVRKRPWGRYAAEIRDPWKKTRVWLGTFDTPVEAAFAYDRAARTLRGAKAKTNFPDHAHRHHHHRPPLQQQPVPFGGIDLNLNFPSPWHFVYFSPPAAAPAPRLPQEASPAASVPPSTALELGMAPRPAGLPFDLNEPPSLLFGS

>cDNA
[truncated: 275,437 more chars]
